# Supplementary material for: Enantioselective Ugi and Ugi-azide reactions catalyzed by anionic stereogenic-at-cobalt(III) complexes
Source: Nat Commun. 2022 Nov 18;13:7065. doi: 10.1038/s41467-022-34887-1 (PMC9674650; doi:10.1038/s41467-022-34887-1)
Supplement: Supplementary file 2 — Supplementary Information [file 41467_2022_34887_MOESM2_ESM.pdf]

Supplementary Information for

Enantioselective Ugi and Ugi-azide reactions catalyzed by anionic stereogenic-at-cobalt(III) complexes

Bing-Bing Sun[1,3], Kun Liu[1,3], Quan Gao[2,3], Wei Fang[1], Shuang Lu[1], Chun-Ru Wang[1], Chuan-Zhi Yao[1], Hai-Qun Cao[2,*] and Jie Yu[1,2,*]

[1] Department of Applied Chemistry, Anhui Agricultural University, Hefei 230036, China.

[2] School of Plant Protection, Anhui Province Engineering Laboratory for Green Pesticide Development and Application, and Anhui Province Key Laboratory of Crop Integrated Pest Management, Anhui Agricultural University, Hefei 230036, China.

[3] These authors contributed equally: Bing-Bing Sun, Kun Liu and Quan Gao.

[*] Email: jieyu@ustc.edu.cn (J.Y.); haiquncao@163.com (H.-Q. C.).

Table of Contents

1. Supplementary Methods S-2
2. Supplementary Discussion S-3
   2.1 Optimization studies for asymmetric Ugi-4CRs S-3
   2.2 Optimization studies for asymmetric Ugi-azide reactions S-5
   2.3 Preliminary bioassay results S-7
   2.4 Comparison of the ratio of NaN_3:CH_3COOH in the Ugi-azide reactions S-9
   2.5. Crystallography data S-10
   2.6 The non-linear effect studies S-14
   2.7 The kinetic studies of partial reaction orders S-18
3. Supplementary Notes S-27
   3.1. Preparation of anionic stereogenic-at-cobalt(III) complexes S-27
   3.2. Experimental Procedures of Asymmetric Ugi-4CRs and Characteristic Data S-28
   3.3. Experimental Procedures of Asymmetric Ugi-azide reactions and Characteristic Data S-48
   3.4. Asymmetric Ugi-4CRs and Ugi-azide Reactions in Gram-Scale S-63
   3.5. Control experiments S-64
   3.6. NMR spectra S-67
4. Supplementary References S-255

1. Supplementary Methods

Analytic grade solvents for the column chromatography and commercially available reagents were used as received. Toluene was dried over Na and distilled prior to use.

^{1}H-NMR, ^{13}C-NMR and ^{19}F-NMR spectrums were recorded on an Agilent 600 NMR spectrometer at 600 MHz, 151 MHz and 564 MHz using $CDCl_3$ as solvent, respectively. ^{1}H-NMR data are reported as follows: δ, chemical shift; coupling constants (*J* are given in Hertz, Hz) and integration. Abbreviations to denote the multiplicity of a particular signal were s (singlet), d (doublet), t (triplet), q (quartet) and m (multiplet). Chemical shifts were reported in ppm from the tetramethylsilane with the solvent resonance as internal standard.

Melting points were measured on a digital melting point apparatus and the temperature was uncorrected. High resolution mass spectrometric measurements (HRMS) were performed by the Waters Xevo G2-XS TOF (ESI Source). Optical rotations were recorded on an Anton Paar MCP-100 polarimeter.

HPLC analysis was performed on Waters-Breeze (2487 Dual λ Absorbance Detector and 1525 Binary HPLC Pump, UV detection monitored at 254 nm). Chiralpak IA, IB, IC, ID, IE and IF columns were purchased from Daicel Chemical Industries, Ltd.

Single crystal structures of the compounds were determined by measuring X-ray intensity data on a 'Bruker APEX-II CCD' diffractometer. The crystal was kept at low and room temperature (172-298 K) during data collection. Using Olex2, the structure was solved with the ShelXT structure solution program using Intrinsic Phasing and refined with the ShelXL refinement package using Least Squares minimization. The non-hydrogen atoms were refined anisotropically and all the hydrogen atoms were assigned in idealized locations.

2. Supplementary Discussion

2.1 Optimization studies for asymmetric Ugi-4CRs

Supplementary Table 1. Optimization studies for asymmetric Ugi-4CRs

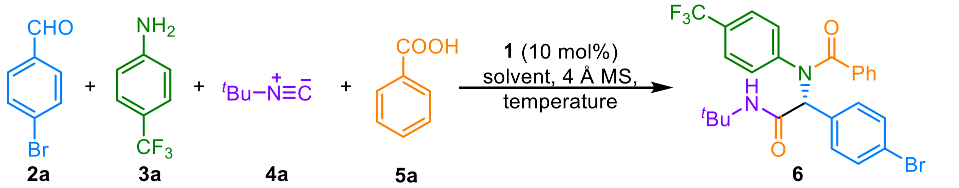

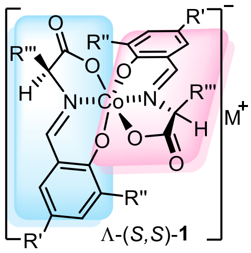

1a: R' = R'' = ^{t}Bu, R''' = ^{i}Pr, M = H;
1b: R' = R'' = ^{t}Bu, R''' = ^{i}Pr, M = Na;
1c: R' = R'' = ^{t}Amyl, R''' = ^{i}Pr , M = H;
1d: R' = R'' = ^{t}Amyl, R''' = ^{i}Pr , M = Na;
1e: R' = R'' = ^{t}Amyl, R''' = ^{t}Bu , M = H;
1f: R' = R'' = ^{t}Amyl, R''' = ^{t}Bu, M = Na;
1g: R' = R'' = R''' = ^{t}Bu, M = H;
1h: R' = R'' = R''' = ^{t}Bu, M = Na;
1i: R' = R'' = ^{t}Bu, R''' = Bn, M = Na;
1j: R' = R'' = ^{t}Bu, R''' = ^{i}Bu, M = Na;
1k: R' = R'' = ^{t}Bu, R''' = ^{s}Bu, M = Na;
1l: R' = R'' = ^{t}Bu, R''' = Cy, M = Na;
1m: R' = R'' = R''' = ^{t}Bu, M = Li;
1n: R' = R'' = R''' = ^{t}Bu, M = K;
1o: R' = R''' = ^{t}Bu, R'' = TMS, M = Na;
1p: R' = R''' = ^{t}Bu, R'' = TES, M = Na.

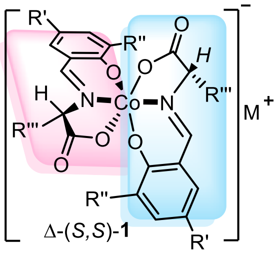

Entry	1	solvent	ratio of 2a:3a:4a:5a	temperature (°C)	yield (%)[a]	e.r.[b]
1	Λ-(*S*,*S*)-**1a**	toluene	1.2:1:1.2:1	-20	54	80.5:19.5
2	Λ-(*S*,*S*)-**1b**	toluene	1.2:1:1.2:1	-20	33	85.5:14.5
3	Λ-(*S*,*S*)-**1c**	toluene	1.2:1:1.2:1	-20	63	80.5:19.5
4	Λ-(*S*,*S*)-**1d**	toluene	1.2:1:1.2:1	-20	61	84.5:14.5
5	Λ-(*S*,*S*)-**1e**	toluene	1.2:1:1.2:1	-20	77	88.5:11.5
6	Λ-(*S*,*S*)-**1f**	toluene	1.2:1:1.2:1	-20	64	93:7
7	Λ-(*S*,*S*)-**1g**	toluene	1.2:1:1.2:1	-20	89	92:8
8	Λ-(*S*,*S*)-**1h**	toluene	1.2:1:1.2:1	-20	72	95:5
9	Λ-(*S*,*S*)-**1i**	toluene	1.2:1:1.2:1	-20	trace	N.D.
10	Λ-(*S*,*S*)-**1j**	toluene	1.2:1:1.2:1	-20	27	71:29
11	Λ-(*S*,*S*)-**1k**	toluene	1.2:1:1.2:1	-20	18	69:31
12	Λ-(*S*,*S*)-**1l**	toluene	1.2:1:1.2:1	-20	39	65.5:34.5
13	Λ-(*S*,*S*)-**1m**	toluene	1.2:1:1.2:1	-20	70	94.5:5.5
14	Λ-(*S*,*S*)-**1n**	toluene	1.2:1:1.2:1	-20	83	93.5:6.5
15	Λ-(*S*,*S*)-**1o**	toluene	1.2:1:1.2:1	-20	96	93:7
16	Λ-(*S*,*S*)-**1p**	toluene	1.2:1:1.2:1	-20	40	95:5
17	Δ-(*S*,*S*)-**1b**	toluene	1.2:1:1.2:1	-20	39	28:72
18	Λ-(*S*,*S*)-**1h**	toluene	1.2:1:1.2:1	25	79	82.5:17.5
19	Λ-(*S*,*S*)-**1h**	toluene	1.2:1:1.2:1	0	75	92.5:7.5
20	Λ-(*S*,*S*)-**1h**	toluene	1.5:1:1.2:1	-20	82	95.5:4.5
21	Λ-(*S*,*S*)-**1h**	toluene	2:1:1.2:1	-20	80	95.5:4.5

22	Λ-(*S*,*S*)-**1h**	toluene	1.5:1:2:1	-20	84	95.5:4.5
23	Λ-(*S*,*S*)-**1h**	toluene	1.5:1:3:1	-20	92	95.5:4.5
24	Λ-(*S*,*S*)-**1h**	toluene	1.5:1:3:3	-20	99	96:4
25	Λ-(*S*,*S*)-**1h**	toluene	1.5:1:3:5	-20	99	96.5:3.5
26	Λ-(*S*,*S*)-**1h**	toluene	1.5:1:3:5	-30	99	97:3
27	Λ-(*S*,*S*)-**1h**	toluene	1.5:1:3:5	-40	99	97.5:2.5
28	Λ-(*S*,*S*)-**1h**	$CHCl_3$	1.5:1:3:5	-40	15	76.5:23.5
29	Λ-(*S*,*S*)-**1h**	EtOAc	1.5:1:3:5	-40	N.R.	-
30	Λ-(*S*,*S*)-**1h**	$(CH_2Cl)_2$	1.5:1:3:5	-40	37	73:27
31	Λ-(*S*,*S*)-**1h**	MeCN	1.5:1:3:5	-40	N.R.	-
32	Λ-(*S*,*S*)-**1h**	^{n}hexane	1.5:1:3:5	-40	38	94.5:5.5
33	Λ-(*S*,*S*)-**1h**	Et_2O	1.5:1:3:5	-40	15	95:5
34	Λ-(*S*,*S*)-**1h**	CH_2Cl_2	1.5:1:3:5	-40	19	69.5:30.5
35	Λ-(*S*,*S*)-**1h**	CH_3OH	1.5:1:3:5	-40	N.R.	-
36[c]	Λ-(*S*,*S*)-**1h**	toluene	1.5:1:3:5	-40	99	97:3
37[d]	Λ-(*S*,*S*)-**1h**	toluene	1.5:1:3:5	-40	76	97:3

Asymmetric Ugi-4CRs were performed by using **2a** (0.12 mmol), **3a** (0.10 mmol), **4a** (0.12 mmol), **5a** (0.10 mmol), 4 Å MS (100 mg) and **1** (0.01 mmol) in toluene (2.0 mL) at -20 °C for 48 h. [a] Isolated yields were based on **3a**. [b] e.r. values were determined by chiral stationary HPLC. [c] Λ-(*S*,*S*)-**1h** (0.005 mmol) was employed. [c] Λ-(*S*,*S*)-**1h** (0.002 mmol) was employed. N.R. = no reaction.

2.2 Optimization studies for asymmetric Ugi-azide reactions

Supplementary Table 2. Optimization studies for asymmetric Ugi-azide reactions

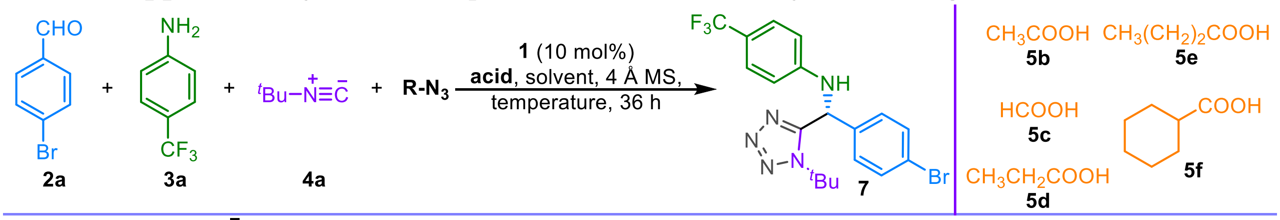

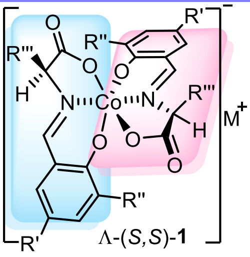

1a: R' = R'' = ^{t}Bu, R''' = ^{i}Pr, M = H;
1b: R' = R'' = ^{t}Bu, R''' = ^{i}Pr, M = Na;
1d: R' = R'' = ^{t}Amyl, R''' = ^{i}Pr , M = Na;
1f: R' = R'' = ^{t}Amyl, R''' = ^{t}Bu, M = Na;
1g: R' = R'' = R''' = ^{t}Bu, M = H;
1h: R' = R'' = R''' = ^{t}Bu, M = Na;
1l: R' = R'' = ^{t}Bu, R''' = Cy, M = Na;
1m: R' = R'' = R''' = ^{t}Bu, M = Li;
1n: R' = R'' = R''' = ^{t}Bu, M = K;
1o: R' = R''' = ^{t}Bu, R'' = TMS, M = Na;
1p: R' = R''' = ^{t}Bu, R'' = TES, M = Na.

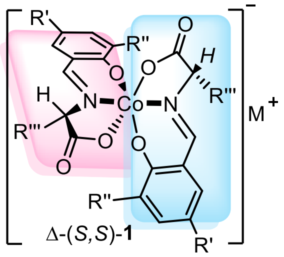

Entry	1	R-N3	solvent	acid	temperature (°C)	yield (%)[a]	e.r.[b]
1	Λ-(*S*,*S*)-**1a**	TMSN$_3$	toluene	-	25	46	80.5:19.5
2	Λ-(*S*,*S*)-**1b**	TMSN$_3$	toluene	-	25	36	91:9
3	Λ-(*S*,*S*)-**1d**	TMSN$_3$	toluene	-	25	16	73.5:26.5
4	Λ-(*S*,*S*)-**1f**	TMSN$_3$	toluene	-	25	76	69.5:30.5
5	Λ-(*S*,*S*)-**1g**	TMSN$_3$	toluene	-	25	68	90.5:9.5
6	Λ-(*S*,*S*)-**1h**	TMSN$_3$	toluene	-	25	96	94:6
7	Λ-(*S*,*S*)-**1l**	TMSN$_3$	toluene	-	25	65	72:28
8	Λ-(*S*,*S*)-**1m**	TMSN$_3$	toluene	-	25	77	91.5:8.5
9	Λ-(*S*,*S*)-**1n**	TMSN$_3$	toluene	-	25	56	56.5:43.5
10	Λ-(*S*,*S*)-**1o**	TMSN$_3$	toluene	-	25	51	91.5:8.5
11	Λ-(*S*,*S*)-**1p**	TMSN$_3$	toluene	-	25	34	83:17
12	Δ-(*S*,*S*)-**1b**	TMSN$_3$	toluene	-	25	34	29:71
13	Λ-(*S*,*S*)-**1h**	TMSN$_3$	CCl_4	-	25	18	89.5:10.5
14	Λ-(*S*,*S*)-**1h**	TMSN$_3$	CH_2Cl_2	-	25	22	63.5:36.5
15	Λ-(*S*,*S*)-**1h**	TMSN$_3$	^{n}hexane	-	25	17	89.5:10.5
16	Λ-(*S*,*S*)-**1h**	TMSN$_3$	$(CH_2Cl)_2$	-	25	39	61:39
17	Λ-(*S*,*S*)-**1h**	TMSN$_3$	toluene	-	-20	76	94.5:5.5
18	Λ-(*S*,*S*)-**1h**	TsN$_3$	toluene	-	-20	trace	-
19	Λ-(*S*,*S*)-**1h**	NaN$_3$	toluene	-	-20	trace	-
20	Λ-(*S*,*S*)-**1h**	NaN$_3$	toluene	$MeNH_3^+Cl^-$	-20	79	93:7
21	Λ-(*S*,*S*)-**1h**	NaN$_3$	toluene	**5b**	-20	65	95:5
22	Λ-(*S*,*S*)-**1h**	NaN$_3$	toluene	$Et_3NH^+Cl^-$	-20	71	90.5:9.5

23[c]	Λ-(*S*,*S*)-**1h**	NaN_3	toluene	**5b**	-30	82	95.5:4.5
24[c]	Λ-(*S*,*S*)-**1h**	NaN_3	toluene	**5c**	-30	77	95:5
25[c]	Λ-(*S*,*S*)-**1h**	NaN_3	toluene	**5d**	-30	69	93:7
26[c]	Λ-(*S*,*S*)-**1h**	NaN_3	toluene	**5e**	-30	65	92:8
27[c]	Λ-(*S*,*S*)-**1h**	NaN_3	toluene	**5f**	-30	72	82:18
28[c]	Λ-(*S*,*S*)-**1h**	NaN_3	toluene	CF_3COOH	-30	-[d]	N.D.
29[c]	Λ-(*S*,*S*)-**1h**	NaN_3	toluene	CF_3SO_3H	-30	-[d]	N.D.
30[c]	Λ-(*S*,*S*)-**1h**	NaN_3	toluene	$TsOH\cdot H_2O$	-30	-[d]	N.D.

Asymmetric Ugi-azide reactions were performed by using **2a** (0.15 mmol), **3a** (0.10 mmol), **4a** (0.30 mmol), R-N_3 (0.30 mmol), acid (0.30 mmol), 4 Å MS (100 mg) and **1** (0.01 mmol) in toluene (2.0 mL) for 36 h. [a] Isolated yields were based on **3a**. [b] e.r. values were determined by chiral stationary HPLC. [c] acid (0.40 mmol) was employed. [d] A messy reaction was observed. N.D. = not detected.

2.3 Preliminary bioassay results

Supplementary Table 3. *In vitro* antifungal activities of selected compounds

compounds	Inhibitory rate/%		
	C. gloeosporioides Penz.	*B. cinerea*	*F. oxysporum* (Schl.) F. sp. *cucumerinum* Owen
**6**	42.67±1.00	11.14±0.12	14.91±0.21
**7**	21.70±0.15	22.60±0.30	24.30±0.40
**16**	30.67±0.25	9.30±0.20	15.42±0.60
**33**	14.50±0.22	9.20±0.18	10.60±0.50
**42**	22.90±0.18	18.00±0.30	12.80±0.70
**54**	30.67±1.10	14.36±0.32	17.42±0.26
**60**	23.60±0.40	19.40±0.28	30.60±0.40
**61**	16.40±0.80	14.00±0.60	20.40±0.28
**62**	10.00±0.60	12.90±0.80	9.00±0.50
**67**	26.20±0.50	25.00±0.90	28.90±0.70
**75**	58.45±0.95	61.97±1.50	51.48±0.72
**77**	36.71±0.85	60.56±0.80	33.22±1.28
**82**	49.28±0.90	38.03±0.28	29.22±0.32
**84**	18.20±0.35	10.60±0.45	6.40±0.80
Prochloraz	85.30±1.36	92.80±1.30	100.00±0.00

The antifungal activities of selected products were tested at the concentration of 100 μg/mL. All data are the average values of three replications.

Selected trifluoromethyl group-containing α-acylamino amides and tetrazoles were tested against three phytopathogenic fungi (Colletotrichum gloeosporioides Penz., Botrytis cinerea and Fusarium oxysporum (Schl.) F. sp. cucumerinum Owen) in vitro with the hyphal growth rate method at a concentration of 100 μg/mL, with Prochloraz used as the positive control (see Supplementary Table S3 for details). As shown in Table 2, α-acylamino amide 6 showed good antifungal activity against C. gloeosporioides Penz. (42.67%) and tetrazole 77 showed good antifungal activity against B. cinerea (60.56%). 75 also exhibited significant inhibitory to C. gloeosporioides Penz. (58.45%), B. cinerea (61.97%) and F. oxysporum (Schl.) F. sp. cucumerinum Owen (51.48%). These chiral products afforded from classical enantioselective Ugi-4CR and Ugi-azide reactions have shown potential antifungal activities and can be regarded as promising candidates in the search for new

pesticide scaffolds. Further investigations into bioactivity study for novel pesticide development are in progress.

Anti-fungal activity assays. The anti-fungal activities of selected compounds were tested *in vitro* using the hyphal growth rate method. The phytopathogenic fungi *Colletotrichum gloeosporioides* Penz., *Botrytis cinerea* and *Fusarium oxysporum* (Schl.) F. sp. *cucumerinum* Owen were obtained from Plant Pathology Laboratory of Anhui Agricultural University. All of these phytopathogens inoculated at the centre of the plate and incubated in the dark at 28℃. Subsequently, 0.010 g of filter-sterilized compound dissolved in acetone (1.0 mL). The solution was added to potato dextrose agar medium (PDA medium) (99.0 mL), and then the medium was sub-packed in 6 sterilized petri dishes to obtaining drug-containing medium. Several fungal disks were made on the plate of phytopathogens with a hole punch with a 0.60 cm in diameter. The disk was picked up by an inoculation needle and placed lightly on drug-containing medium plates. This process was repeated 3 times per treatment, with reproducible results.

Determination of the inhibitory rate. The plates were placed in the dark at 28 ℃ and photographed after 48 h or 72 h. The calculation formula was as follows:

$$Inhibitory\,rate\,(\%) = \frac{Diameter\,of\ control - Diameter\,of\ treatment}{Diameter\,of\ control - 0.6\ cm} \times 100$$

In addition, the compounds which showed better antifungal activity were selected to evaluate the half maximal effective concentration (EC_{50}) against the three phytopathogenic fungi (Supplementary Table 4). Firstly, these compounds were dissolved in acetone to formulate as 2000 μg/mL according to the pre-test results, respectively. Then diluted the test solutions at concentrations of 1000, 500, 200, 100, 50, and 20 μg/mL for the subsequent analyses.

Supplementary Table 4. EC_{50} of the selected compounds to tested phytopathogenic fungi

Compounds	*C. gloeosporioides* Penz.		*B. cinerea*		*F. oxysporum* (Schl.) F. sp. *cucumerinum* Owen	
	EC_{50} (μg/mL)	95% CI (μg/mL)	EC_{50} (μg/mL)	95% CI (μg/mL)	EC_{50} (μg/mL)	95% CI (μg/mL)
**75**	87.23	57.99-129.90	62.19	54.73-70.31	88.98	80.20-98.60
**77**	182.60	126.50-269.40	71.84	54.82-92.85	232.10	173.70-316.00
**82**	125.70	76.83-225.10	199.20	143.50-282.80	236.00	178.80-317.30

2.4 Comparison of the ratio of NaN3:CH3COOH in the Ugi-azide reactions

Supplementary Table 5. Comparison of the ratio of NaN3:CH3COOH in the Ugi-azide reactions

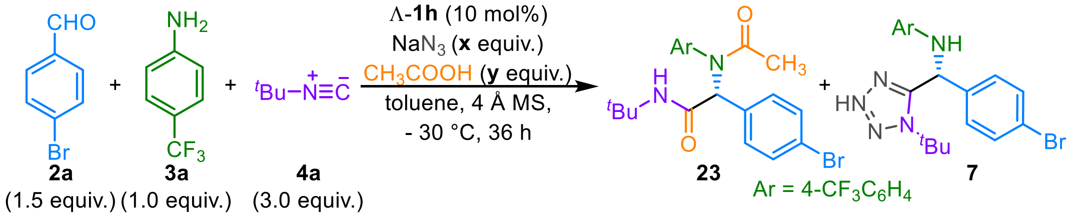

Entry	x	y	23 yield (%)[a]	23 ee[b]	7 yield (%)[a]	7 ee[b]
1	1	2	38	85	12	83
2	1	3	66	88	15	82
3	1	4	69	89	19	83
4	3	2	15	87	31	90
5	3	3	11	88	65	91
6	3	4	13	87	82	91
7	3	5	21	89	71	91
8	5	2	12	89	42	87
9	5	3	11	88	69	88
10	5	4	15	88	84	89
11	5	5	15	91	81	89
12	7	2	9	87	39	82
13	7	3	5	89	62	82
14	7	4	5	90	89	80
15	7	5	11	88	84	82

Asymmetric Ugi-azide reactions were performed by using **2a** (0.15 mmol), **3a** (0.10 mmol), **4a** (0.30 mmol), CH_3COOH (**x** equiv.), NaN_3 (**y** equiv.), 4 Å MS (100 mg) and Λ-(*S*,*S*)-**1h** (0.01 mmol) in toluene (2.0 mL) at -30 °C for 36 h. [a] Isolated yields were based on **3a**. [b] ee values were determined by chiral stationary HPLC.

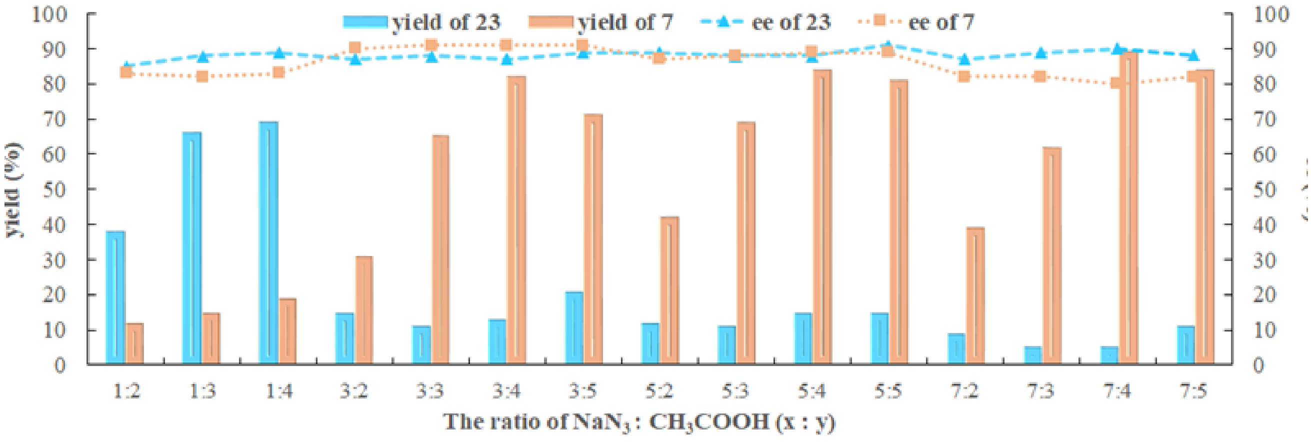

2.5. Crystallography data

Supplementary Table 6. Crystal data and structure refinement for **7** (CCDC 2103292)

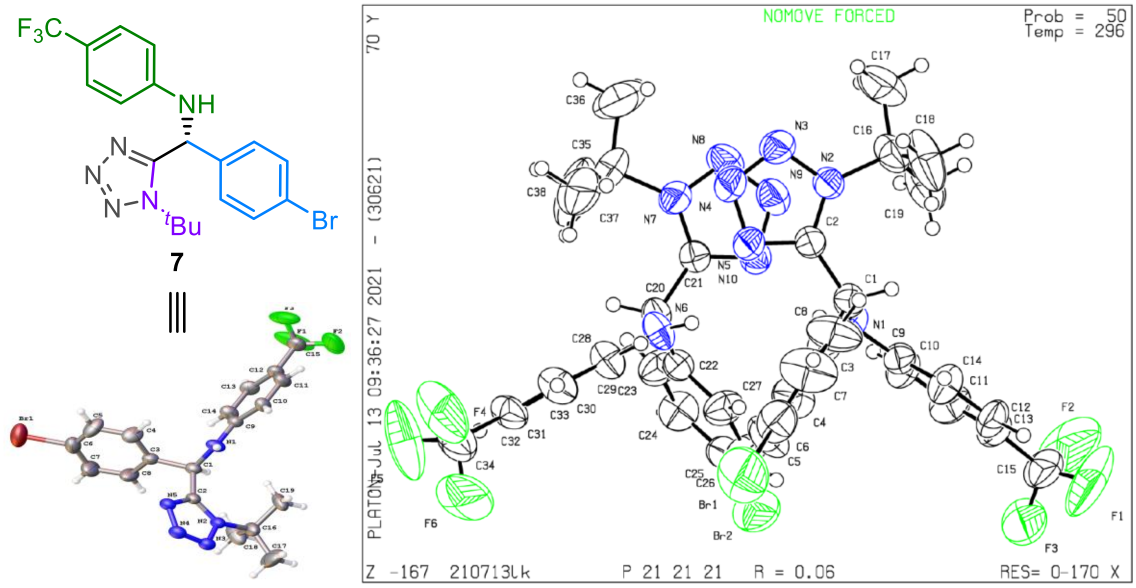

Empirical formula	$C_{19}H_{19}BrF_3N_5$
Formula weight	454.30
Temperature/K	296.15
Crystal system	orthorhombic
Space group	$P2_12_12_1$
a/Å	12.3667(14)
b/Å	14.9515(18)
c/Å	22.783(3)
α/°	90
β/°	90
γ/°	90
Volume/Å^3	4212.5(8)
Z	8
ρ_{calc}g/cm^3	1.433
μ/mm^{-1}	1.991
F(000)	1840.0
Crystal size/mm^3	0.09 × 0.06 × 0.05
Radiation	MoKα (λ = 0.71073)
2Θ range for data collection/°	3.576 to 54.872
Index ranges	$-15 \leq h \leq 15$, $-19 \leq k \leq 16$, $-26 \leq l \leq 29$
Reflections collected	34398

Independent reflections	9541 [R_{int} = 0.0636, R_{sigma} = 0.0910]
Data/restraints/parameters	9541/0/511
Goodness-of-fit on F^2	1.000
Final R indexes [I>=2σ (I)]	R_1 = 0.0572, wR_2 = 0.1118
Final R indexes [all data]	R_1 = 0.1512, wR_2 = 0.1417
Largest diff. peak/hole / e $Å^{-3}$	0.45/-0.52
Flack parameter	0.004(6)

Sample preparation for crystal growth: Compound **7** (20.0 mg) was dissolved in *n*-hexane (5.0 mL), while slow evaporation of solvent at room temperature white crystals were grown. Thermal ellipsoids are shown at 50% probability.

Supplementary Table 7. Crystal data and structure refinement for **31** (CCDC 2103289)

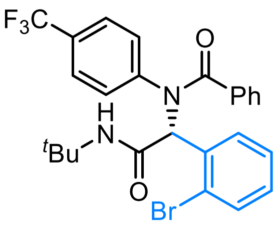

31

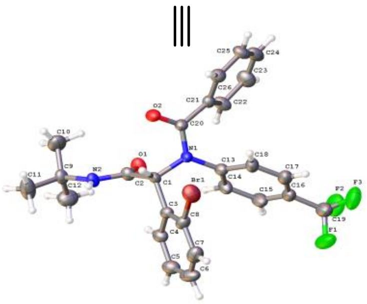

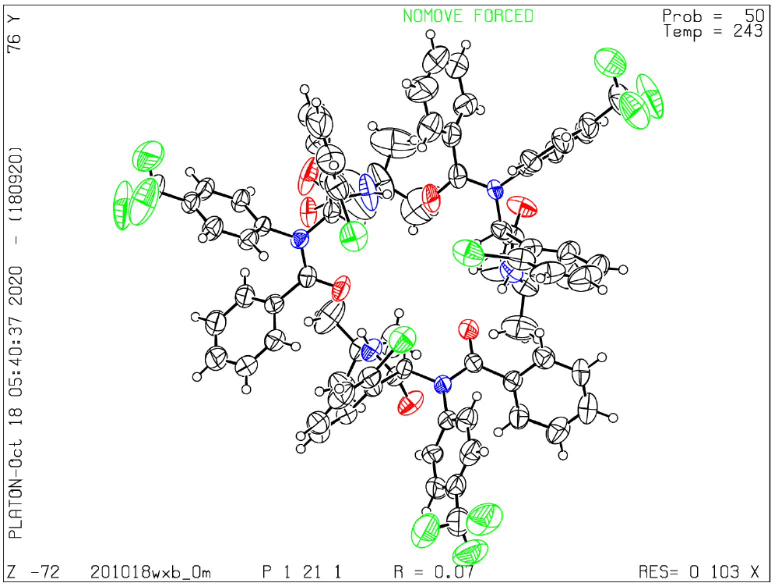

Empirical formula	$C_{26}H_{24}BrF_3N_2O_2$
Formula weight	533.38
Temperature/K	242.89
Crystal system	monoclinic
Space group	P21
a/Å	10.8684(4)
b/Å	26.3933(11)
c/Å	14.2914(6)
α/°	90
β/°	103.855(2)
γ/°	90
Volume/Å^3	3980.3(3)
Z	6
ρ_{calc}g/cm^3	1.335
μ/mm^{-1}	1.699
F(000)	1632.0
Crystal size/mm^3	0.08 × 0.08 × 0.05
Radiation	GaKα (λ = 1.34139)
2Θ range for data collection/°	6.26 to 110.414
Index ranges	$-13 \le h \le 10$, $-32 \le k \le 31$, $-17 \le l \le 17$
Reflections collected	43236
Independent reflections	14953 [Rint = 0.0737, Rsigma = 0.0770]

Data/restraints/parameters	14953/63/937
Goodness-of-fit on F^2	1.032
Final R indexes [I>=2σ (I)]	R_1 = 0.0677, wR_2 =0.1742
Final R indexes [all data]	R_1 = 0.1013, wR_2 = 0.2055
Largest diff. peak/hole / e Å^{-3}	0.73/-0.84
Flack parameter	0.088(10)

Sample preparation for crystal growth: Compound **31** (20.0 mg) was dissolved in *n*-hexane/ isopropanol (v/v = 3/7, 5.0 mL), while slow evaporation of solvent at room temperature white crystals were grown. Thermal ellipsoids are shown at 50% probability.

2.6 The non-linear effect studies

2.6.1 The positive NLE of Asymmetric Ugi-4CRs of 5a:

Supplementary Table 8. The NLE of asymmetric Ugi-4CRs of **5a**[a]

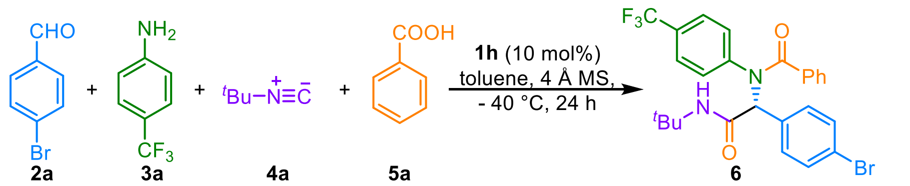

entry	Δ-**1h** (mg)	Λ-**1h** (mg)	*ee*$_1$ of **1h** (%)[b]	c (g/100 mL)[c]	$[\alpha]_{20}$	*ee*$_2$ of **1h** (%)[d]	yield of **6** (%)[e]	*ee* of **6** (%)[f]
1	2.6	47.6	89.64	0.0080	-3050.00	82.90	86	92
2	5.1	44.9	79.60	0.0080	-2633.33	71.57	88	90
3	7.6	42.5	69.66	0.0080	-2462.50	66.93	90	85
4	10.1	40.2	59.84	0.0084	-1928.57	52.42	86	73
5	15.0	35.0	40.00	0.0080	-1408.33	38.28	92	57
6	20.1	30.2	20.08	0.0080	-675.00	18.35	90	29
7	--	2.0	100	0.0080	-3575.00	100	99	95
8	2.0	--	--	0.0080	+3783.33	--	99	95

[a] The scalemic catalyst **1h** was prepared by mixing the two enantiopure catalysts Δ-(*R*,*R*)-**1h** and Λ-(*S*,*S*)-**1h**, which were completely dissolved in MeOH and then the solvent was evaporated in vacuo before used. [b] The *ee*$_1$ of **1h** were calculated by the quality of Δ-(*R*,*R*)-**1h** and Λ-(*S*,*S*)-**1h**. [c] The samples were dissolved in MeOH. [d] The *ee*$_2$ of **1h** were calculated according to the optical rotation values of different scalemic catalysts **1h** divided by the average optical rotation values of Δ-(*R*,*R*)-**1h** and Λ-(*S*,*S*)-**1h**. The *ee*$_2$ values were used as the horizontal coordinate below. [e] Isolated yield. [f] Determined by HPLC analysis.

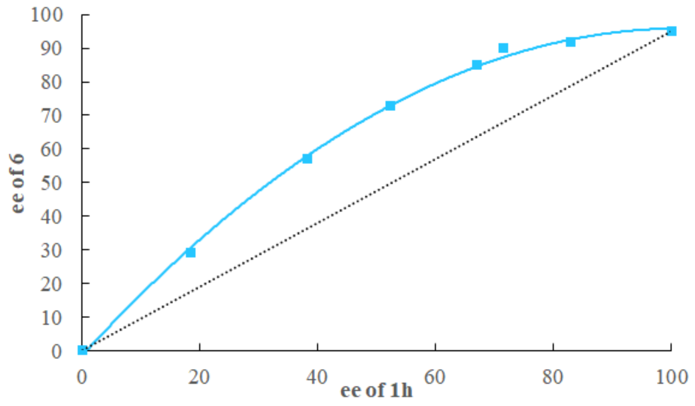

Procedure: A 10-mL oven-dried tube was charged with 4-bromobenzaldehyde **2a** (0.15 mmol), 4-trifluroaniline **3a** (0.10 mmol), scalemic catalyst **1h** (0.01 mmol), 4 Å molecular sieves (100 mg), and toluene (2.0 mL) at room temperature and stirred for 30 min. Then benzoic acid **5a** (0.50 mmol) was added in one portion. The mixture was cooled to -40 °C and stirred for another 30 min. The *tert*-butyl isocyanide **4a** (0.30 mmol) was added in one portion and the resulting solution was stirred vigorously for 24 h. The reaction was then quenched with pre-cooled NEt_3 (-40 °C, 1.0 mmol). The mixture was purified by flash column chromatography (silica gel, petroleum ether/EtOAc/CH_2Cl_2 = 6:1:1) to give the α-acylamino amide **6**.

2.6.2 The general procedure of kinetic studies on Ugi-4CR.

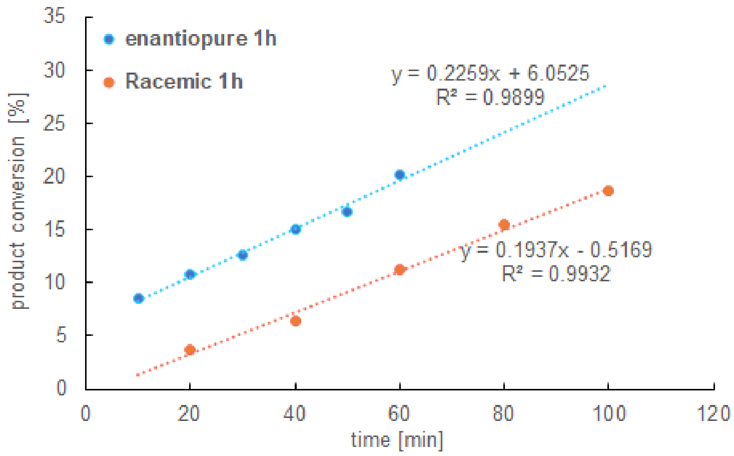

Supplementary Figure 1 | Kinetic studies on Ugi-4CR. The kinetic studies on Ugi-4CR of enantiopure Λ-(*S*,*S*)-**1h** and racemic catalyst [Equivalent mixture of Λ-(*S*,*S*)-**1h** and Δ-(*R*,*R*)-**1h**] were performed. It is revealed that the optically pure Λ-(*S*,*S*)-**1h** showed relatively higher catalytic activity than the racemic catalyst, which might be the possible reason for the positive nonlinear effect.

Procedure: A 10-mL oven-dried tube was charged with 4-bromobenzaldehyde **2a** (0.15 mmol), 4-trifluroaniline **3a** (0.10 mmol), catalyst **1h** (0.01 mmol, either enantiopure or racemic catalyst), 4 Å molecular sieves (100 mg), and toluene (2.0 mL) at room temperature and stirred for 30 min. Then benzoic acid **5a** (0.50 mmol) was added in one portion. The mixture was cooled to -40 °C and stirred for another 30 min. The *tert*-butyl isocyanide **4a** (0.30 mmol) was added in one portion and the resulting solution was stirred for a specific time (10 min, 20 min, 30 min, 40 min, 50 min, 60 min, 80 min, 100 min). The reaction was then quenched with pre-cooled NEt_3 (-40 °C, 1.0 mmol) and saturated aqueous $NaHCO_3$ (1.0 mL). Then the mixture was diluted by water (10 mL) and DCM (10 mL). The organic phase was separated and the aqueous phase was extracted by DCM (10 mL × 2). The organic phase was combined, dried over anhydrous Na_2SO_4, filtrated and evaporated under reduced pressure. The residue was resolved in $CDCl_3$ (0.6 mL) and diphenylmethane (16.8 mg, 0.10 mmol) was added as internal standard for ^{1}H-NMR analysis.

2.6.3 The positive NLE of Asymmetric Ugi and Ugi-azide reactions.

Supplementary Table 9. The NLE of asymmetric Ugi and Ugi-azide reactions[a]

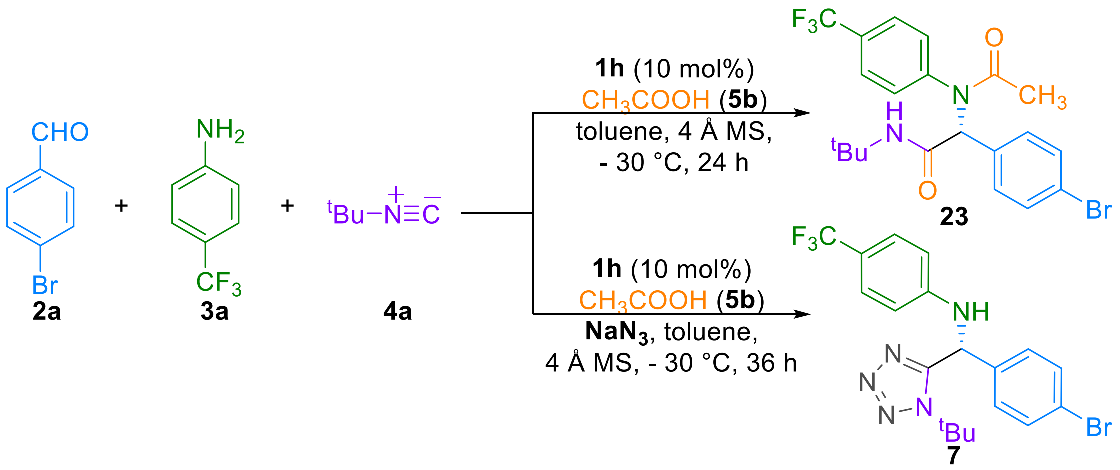

entry	Δ-**1h** (mg)	Λ-**1h** (mg)	*ee*$_1$ of **1h** (%)[b]	c (g/100 mL)[c]	$[\alpha]_{20}$	*ee*$_2$ of **1h** (%)[d]	yield of **23** (%)[e]	*ee* of **23** (%)[f]	yield of **7** (%)[e]	*ee* of **7** (%)[f]
1	2.5	47.5	90.00	0.0088	-3284.09	98.31	75	86	78	82
2	5.3	44.9	78.88	0.0088	-2340.91	70.08	71	82	82	77
3	7.5	42.5	70.06	0.0084	-2250.00	67.36	71	76	81	64
4	9.9	40.3	60.56	0.0088	-1882.58	56.36	71	65	76	58
5	14.9	34.9	40.16	0.0084	-1333.33	39.92	76	47	79	40
6	20.1	30.3	20.24	0.0080	-787.50	23.58	75	37	61	20
7	--	2.2	100	0.0088	-3367.42	100	78	89	79	91
8	2.1	--	--	0.0084	+3313.19	--	80	89	82	91

[a] The scalemic catalyst **1h** was prepared by mixing the two enantiopure catalysts Δ-(*R*,*R*)-**1h** and Λ-(*S*,*S*)-**1h**, which were completely dissolved in MeOH and then the solvent was evaporated in vacuo before used. [b] The *ee*$_1$ of **1h** were calculated by the quality of Δ-(*R*,*R*)-**1h** and Λ-(*S*,*S*)-**1h**. [c] The samples were dissolved in MeOH. [d] The *ee*$_2$ of **1h** were calculated according to the optical rotation values of different scalemic catalysts **1h** divided by the average optical rotation values of Δ-(*R*,*R*)-**1h** and Λ-(*S*,*S*)-**1h**. The *ee*$_2$ values were used as the horizontal coordinate below. [e] Isolated yield. [f] Determined by HPLC analysis.

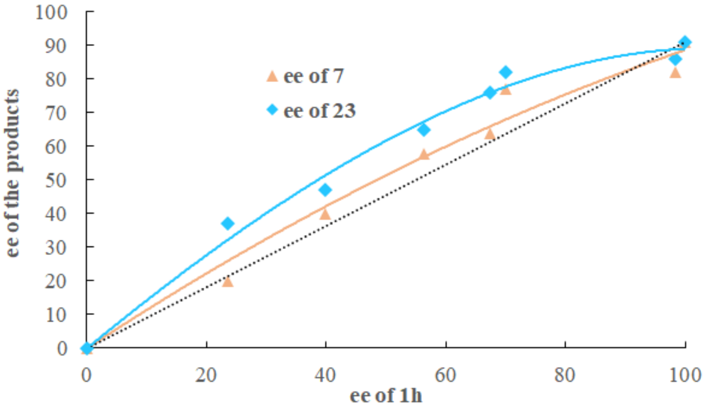

Procedure of asymmetric Ugi reaction of 5b: A 10-mL oven-dried tube was charged with 4-bromobenzaldehyde **2a** (0.15 mmol), 4-trifluroaniline **3a** (0.10 mmol), scalemic catalyst **1h** (0.01 mmol), 4 Å molecular sieves (100 mg), and toluene (2.0 mL) at room temperature and stirred for 30 min. Then acetic acid **5b** (0.50 mmol) was added in one portion. The mixture was cooled to -30 °C and stirred for another 30 min. The *tert*-butyl isocyanide **4a** (0.30 mmol) was added in one portion and the resulting solution was stirred vigorously for 24 h. The reaction was then quenched with pre-cooled NEt_3 (-40 °C, 1.0 mmol). The mixture was purified by flash column chromatography (silica gel, petroleum ether/EtOAc/CH_2Cl_2 = 6:1:1) to give the α-acylamino amide **23**

Procedure of asymmetric Ugi-azide reaction: A 10-mL oven-dried tube was charged with 4-bromobenzaldehyde **2a** (0.15 mmol), 4-trifluroaniline **3a** (0.10 mmol), scalemic catalyst **1h** (0.01 mmol), NaN_3 (0.30 mmol), 4 Å molecular sieves (100 mg), and toluene (2.0 mL) at room temperature and stirred for 30 min. Then acetic acid **5b** (0.40 mmol) was added in one portion. The mixture was cooled to -30 °C and stirred for another 30 min. The *tert*-butyl isocyanide **4a** (0.30 mmol) was added in one portion and the resulting solution was stirred vigorously for 36 h. The reaction was then quenched with pre-cooled NEt_3 (-30 °C, 1.0 mmol). The mixture was purified by flash column chromatography (silica gel, petroleum ether/EtOAc/CH_2Cl_2 = 6:1:1) to give the α-aminotetrazole **7**.

2.7 The kinetic studies of partial reaction orders

General procedure for the preparation of the samples for kinetic measurements on Ugi-4CR: A 10-mL oven-dried tube was charged with 4-bromobenzaldehyde **2a** (0.15 mmol), 4-trifluroaniline **3a** (0.10 mmol), catalyst **1h** (0.01 mmol), 4 Å molecular sieves (100 mg), and toluene (2.0 mL) at room temperature and stirred for 30 min. Then acetic acid **5b** (0.50 mmol) was added in one portion. The mixture was cooled to -30 °C and stirred for another 30 min. The *tert*-butyl isocyanide **4a** (0.30 mmol) was added in one portion and the resulting solution was stirred for a specific time (3 min, 6 min, 9 min, 12 min, 15 min, 18 min). The reaction was then quenched with pre-cooled NEt_3 (-30 °C, 1.0 mmol) and saturated aqueous $NaHCO_3$ (1.0 mL). Then the mixture was diluted by water (10 mL) and DCM (10 mL). The organic phase was separated and the aqueous phase was extracted by DCM (10 mL × 2). The organic phase was combined, dried over anhydrous Na_2SO_4, filtrated and evaporated under reduced pressure. The residue was resolved in $CDCl_3$ (0.6 mL) and diphenylmethane (16.8 mg, 0.10 mmol) was added as internal standard for ^{1}H-NMR analysis.

General procedure for the preparation of the samples for kinetic measurements on Ugi-azide reactions: A 10-mL oven-dried tube was charged with 4-bromobenzaldehyde **2a** (0.15 mmol), 4-trifluroaniline **3a** (0.10 mmol), catalyst **1h** (0.01 mmol), 4 Å molecular sieves (100 mg), and toluene (2.0 mL) at room temperature and stirred for 30 min. Then acetic acid **5b** (0.50 mmol) was added in one portion. The mixture was cooled to -30 °C and stirred for another 30 min. The *tert*-butyl isocyanide **4a** (0.30 mmol) was added in one portion and the resulting solution was stirred for a specific time (3 min, 6 min, 9 min, 12 min, 15 min, 18 min). The reaction was then quenched with pre-cooled NEt_3 (-30 °C, 1.0 mmol) and saturated aqueous $NaHCO_3$ (1.0 mL). Then the mixture was diluted by water (10 mL) and DCM (10 mL). The organic phase was separated and the aqueous phase was extracted by DCM (10 mL × 2). The organic phase was combined, dried over anhydrous Na_2SO_4, filtrated and evaporated under reduced pressure. The residue was resolved in $CDCl_3$ (0.6 mL) and diphenylmethane (16.8 mg, 0.10 mmol) was added as internal standard for ^{1}H-NMR analysis.

2.7.1 Determination of the statistical error of the used method on Ugi-4CR

The statistical error of the used method was determined by reproducing the standard Ugi-4CR 4 times. The relative standard deviation was only 2.58%.

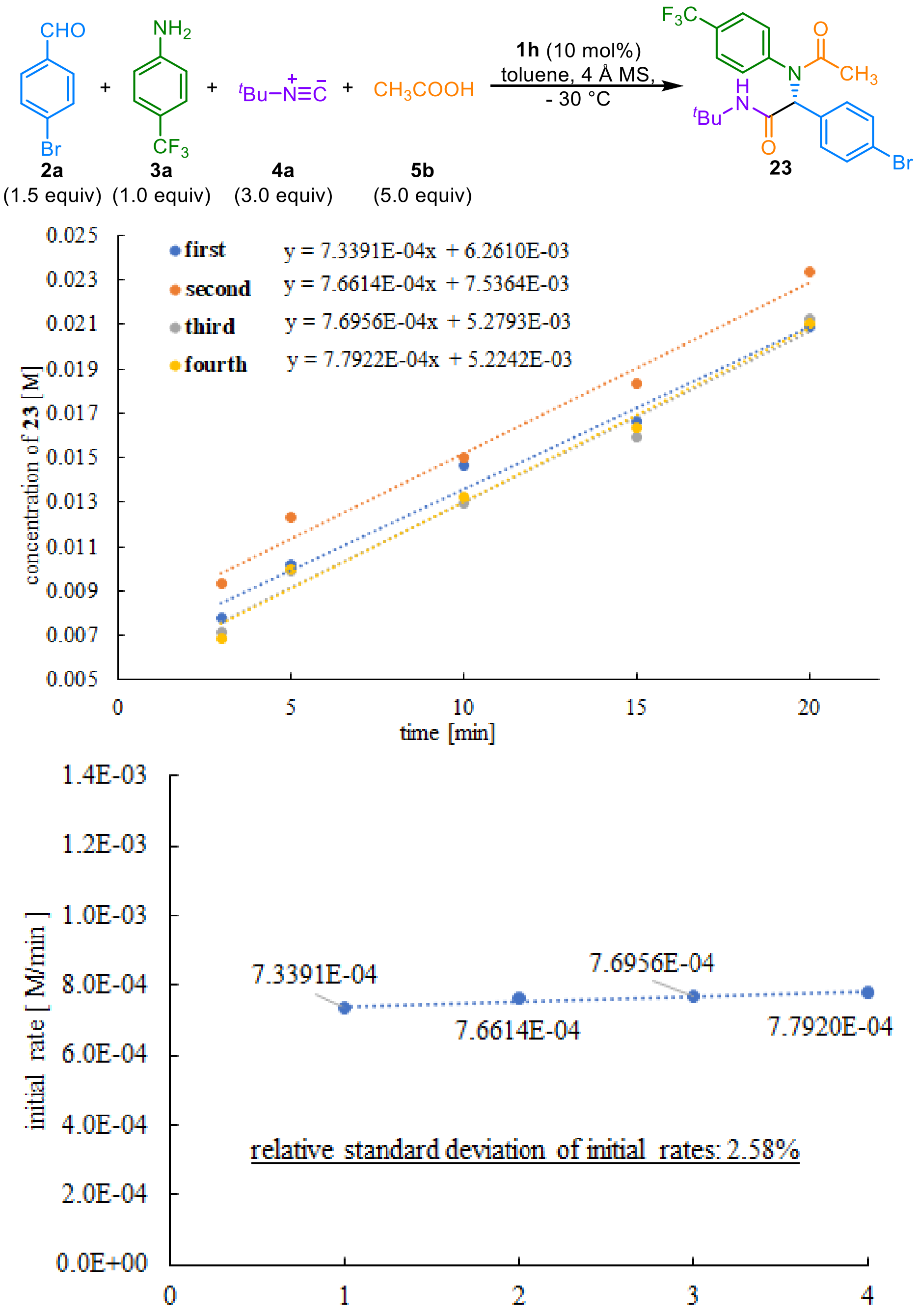

Supplementary Figure 2 | Determination of rates of the used method on Ugi-4CR.

2.7.2 Catalyst 1h rate order on Ugi-4CRs

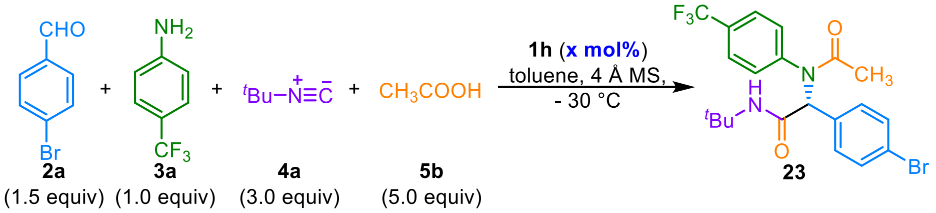

The rate order of catalyst **1h** was evaluated following the general procedure, varying the starting-concentration of **1h** between 2 mol% and 15 mol%. The quantitative results and graghs of these experiments are provided below, which demonstrated first-order dependence with repect to the catalyst **1h**.

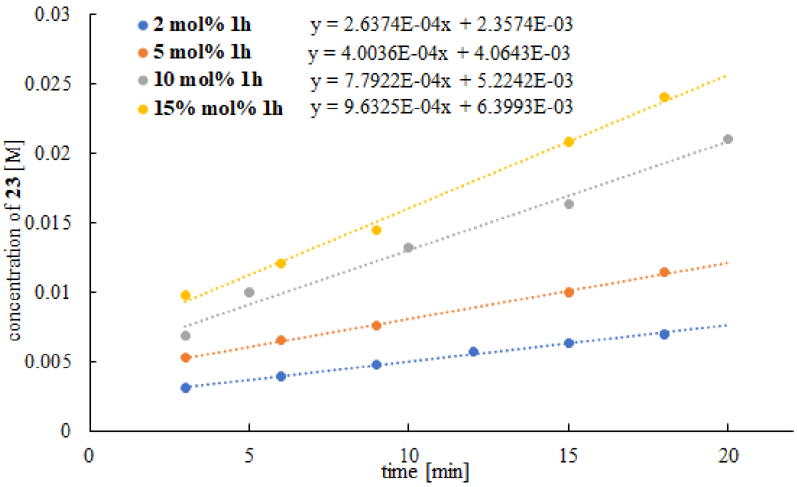

Supplementary Figure 3 | Rates determined from varying [catalyst **1h**].

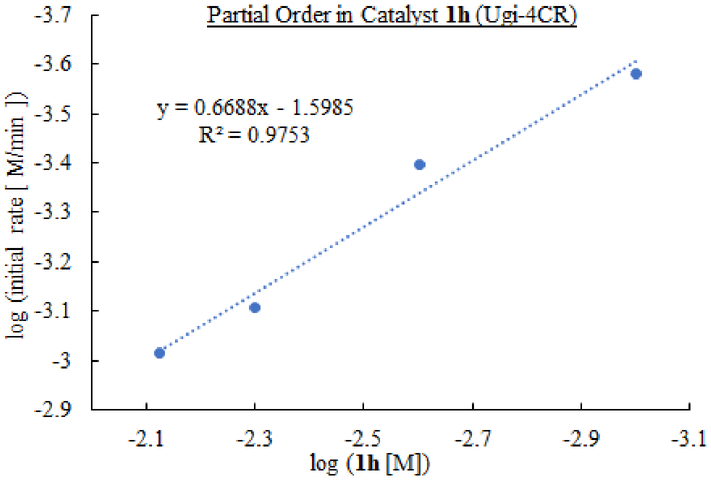

**1h** [M]	**2a** [M]	**3a** [M]	**4a** [M]	**5b** [M]	Initial rate [M/min]
0.001	0.075	0.050	0.150	0.250	2.6374×10^{-4}
0.0025	0.075	0.050	0.150	0.250	4.0036×10^{-4}
0.005	0.075	0.050	0.150	0.250	7.7922×10^{-4}
0.0075	0.075	0.050	0.150	0.250	9.6325×10^{-4}

Supplementary Figure 4 | Plot of initial rates *vs.* [catalyst **1h**] and associated tabulated data.

2.7.3 Aldimine 99a rate order on Ugi-3CRs

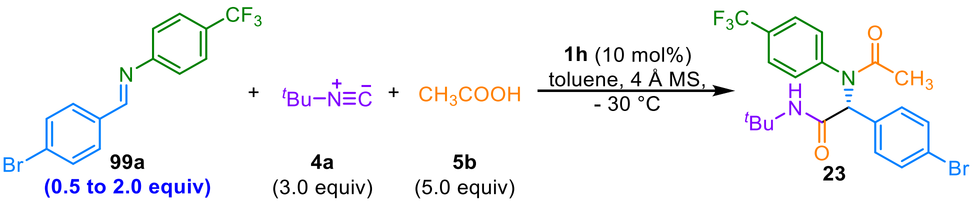

The rate order of aldimine **99a** was evaluated instead of 4-bromobenzaldehyde and 4-trifluroaniline following the general procedure, varying the starting-concentration of aldimine **99a** between 0.5 equiv. and 2.0 equiv. The quantitative results and graghs of these experiments are provided below, which demonstrated first-order dependence with repect to the aldimine **99a**.

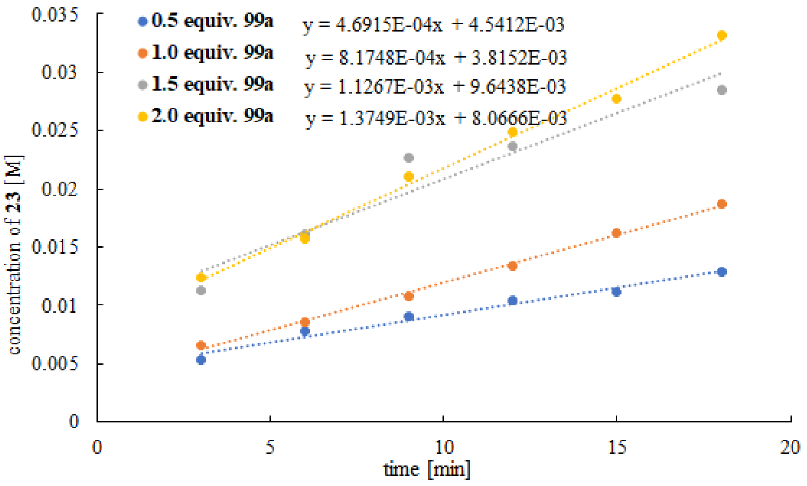

Supplementary Figure 5 | Rates determined from varying [aldimine **99a**].

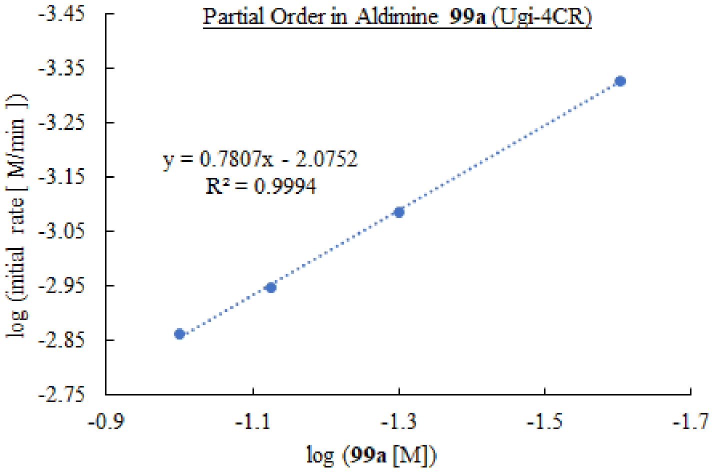

**1h** [M]	**99a** [M]	**4a** [M]	**5b** [M]	Initial rate [M/min]
0.005	0.025	0.150	0.250	4.6915×10^{-4}
0.005	0.050	0.150	0.250	8.1748×10^{-4}
0.005	0.075	0.150	0.250	1.1267×10^{-3}
0.005	0.100	0.150	0.250	1.3749×10^{-3}

Supplementary Figure 6 | Plot of initial rates *vs.* [aldimine **99a**] and associated tabulated data.

2.7.4 Isocyanide 4a rate order on Ugi-4CRs

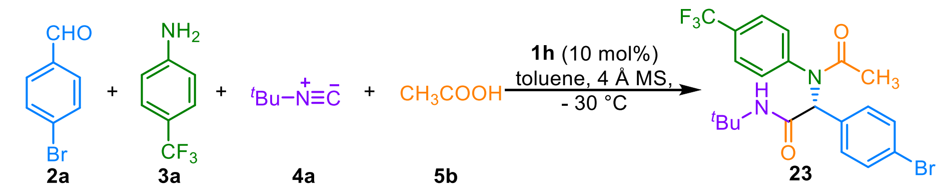

2a (1.5 equiv) **3a** (1.0 equiv) **4a (0.5 to 5.0 equiv)** **5b** (5.0 equiv) **23**

The rate order of *tert*-butyl isocyanide **4a** was evaluated following the general procedure, varying the starting-concentration of *tert*-butyl isocyanide **4a** between 0.5 eq and 5.0 eq. The quantitative results and graghs of these experiments are provided below, which demonstrated first-order dependence with repect to the isocyanide **4a**.

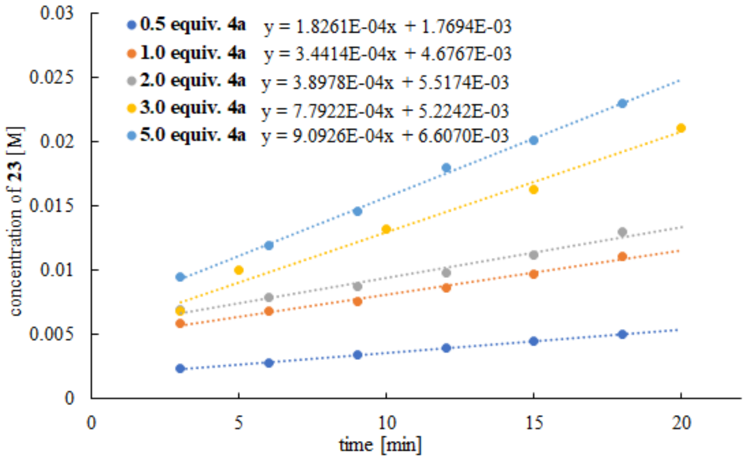

Supplementary Figure 7 | Rates determined from varying [isocyanide **4a**].

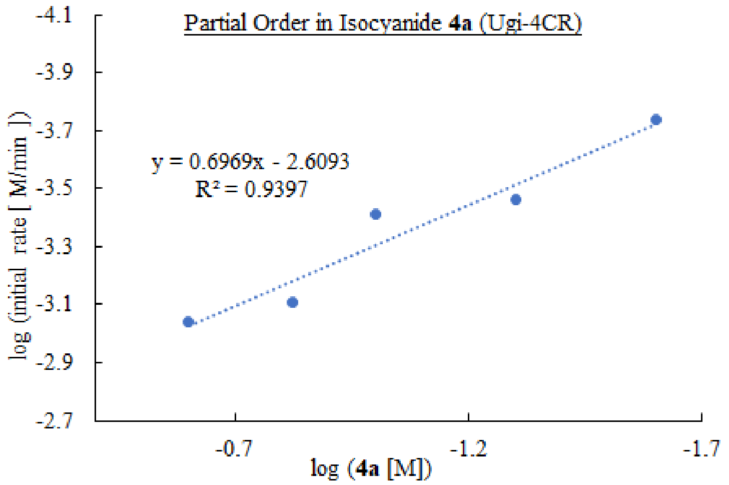

**1h** [M]	**2a** [M]	**3a** [M]	**4a** [M]	**5b** [M]	Initial rate [M/min]
0.005	0.075	0.050	0.025	0.250	1.8261×10^{-4}
0.005	0.075	0.050	0.050	0.250	3.4414×10^{-4}
0.005	0.075	0.050	0.100	0.250	3.8978×10^{-4}
0.005	0.075	0.050	0.150	0.250	7.7922×10^{-4}
0.005	0.075	0.050	0.250	0.250	9.0926×10^{-4}

Supplementary Figure 8 | Plot of initial rates *vs.* [isocyanide **4a**] and associated tabulated data.

2.7.5 Acetic acid 5b rate order on Ugi-4CRs

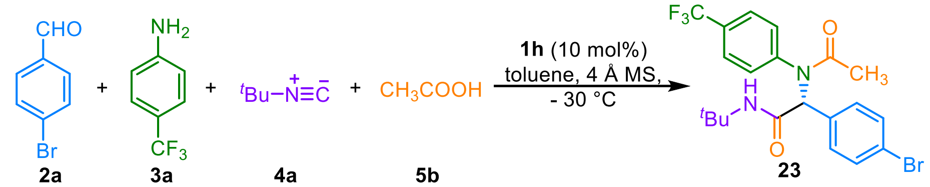

2a (1.5 equiv) 3a (1.0 equiv) 4a (3.0 equiv) 5b (1.5 to 5.0 equiv) 23

The rate order of acetic acid **5b** was evaluated following the general procedure, varying the starting-concentration of acetic acid **5b** between 1.5 eq and 5.0 eq. The quantitative results and graghs of these experiments are provided below, which demonstrated first-order dependence with repect to the acetic acid **5b**.

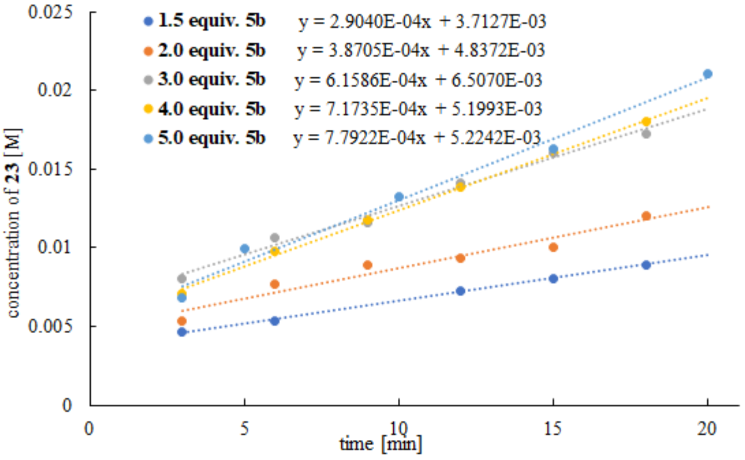

Supplementary Figure 9 | Rates determined from varying [acetic acid **5b**].

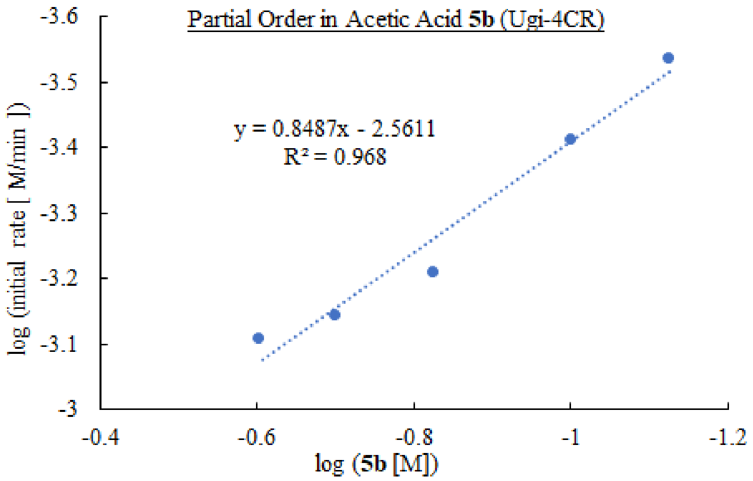

**1h** [M]	**2a** [M]	**3a** [M]	**4a** [M]	**5b** [M]	Initial rate [M/min]
0.005	0.075	0.050	0.150	0.075	2.9040×10^{-4}
0.005	0.075	0.050	0.150	0.100	3.8705×10^{-4}
0.005	0.075	0.050	0.150	0.150	6.1586×10^{-4}
0.005	0.075	0.050	0.150	0.200	7.1735×10^{-4}
0.005	0.075	0.050	0.150	0.250	7.7922×10^{-4}

Supplementary Figure 10 | Plot of initial rates *vs.* [acetic acid **5b**] and associated tabulated data.

2.7.6 Acetic acid 5b rate order on Ugi-azide reactions

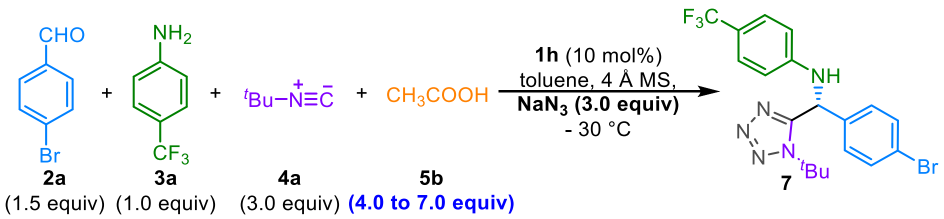

The rate order of acetic acid **5b** was evaluated following the general procedure, varying the starting-concentration of acetic acid **5b** between 4.0 eq and 7.0 eq. The quantitative results and graghs of these experiments are provided below, which also demonstrated first-order dependence with repect to the acetic acid **5b**.

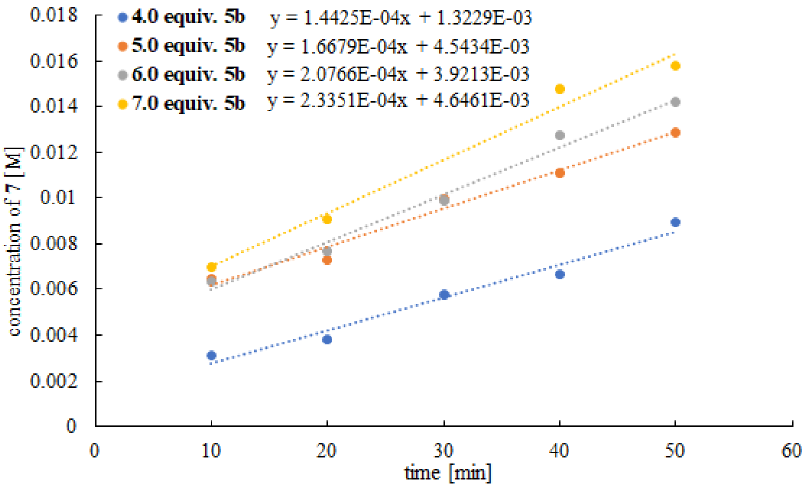

Supplementary Figure 11 | Rates determined from varying [acetic acid **5b**].

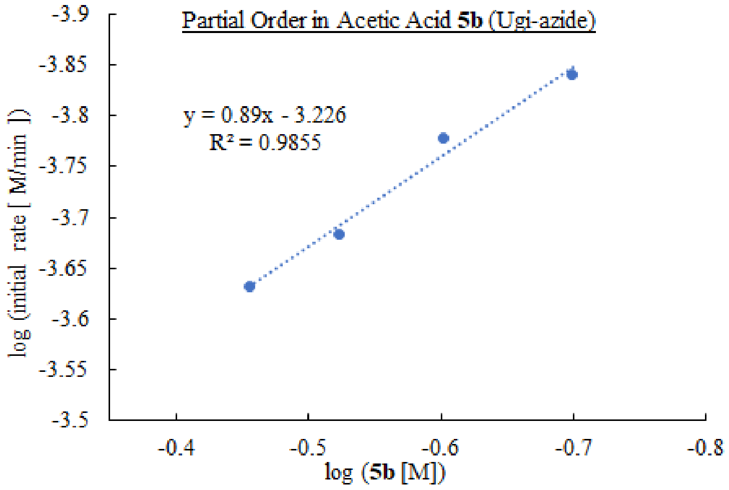

**1h** [M]	**2a** [M]	**3a** [M]	**4a** [M]	**5b** [M]	NaN3	Initial rate [M/min]
0.005	0.075	0.050	0.150	0.200	0.150	1.4425×10^{-4}
0.005	0.075	0.050	0.150	0.250	0.150	1.6679×10^{-4}
0.005	0.075	0.050	0.150	0.300	0.150	2.0766×10^{-4}
0.005	0.075	0.050	0.150	0.350	0.150	2.3351×10^{-4}

Supplementary Figure 12 | Plot of initial rates *vs.* [acetic acid **5b**] and associated tabulated data.

2.7.7 NaN_3 rate order on Ugi-azide reactions

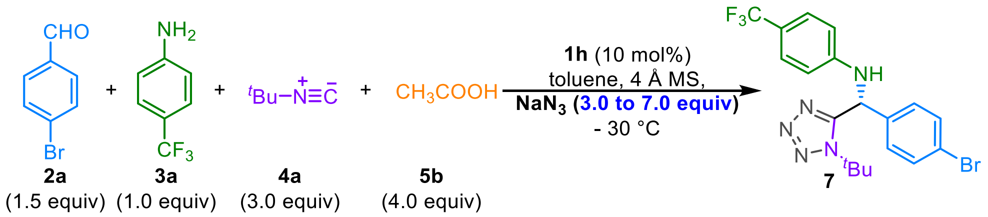

The rate order of NaN_3 was evaluated following the general procedure, varying the starting-concentration of NaN_3 between 3.0 eq and 7.0 eq. The quantitative results and graphs of these experiments are provided below. From the plot it is evident that the overall reaction rate under these conditions is independent on the NaN_3 concentration (NaN_3 is insoluble in toluene), i.e., the reaction is zero-order with respect to NaN_3.

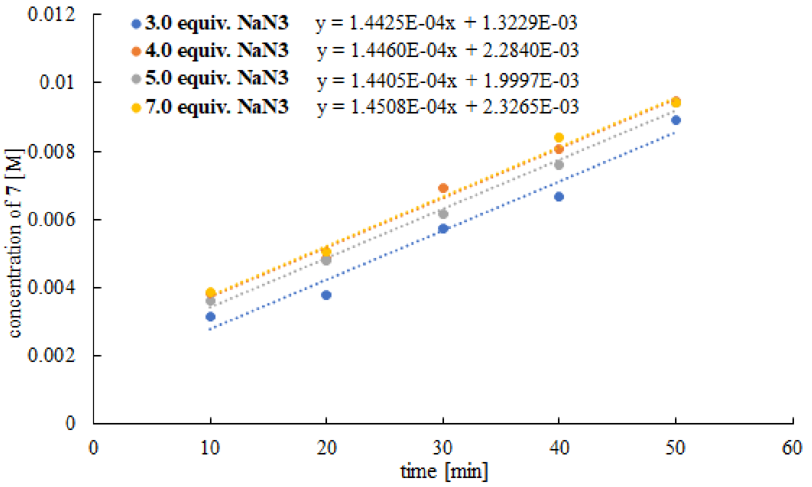

Supplementary Figure 13 | Rates determined from varying [NaN_3].

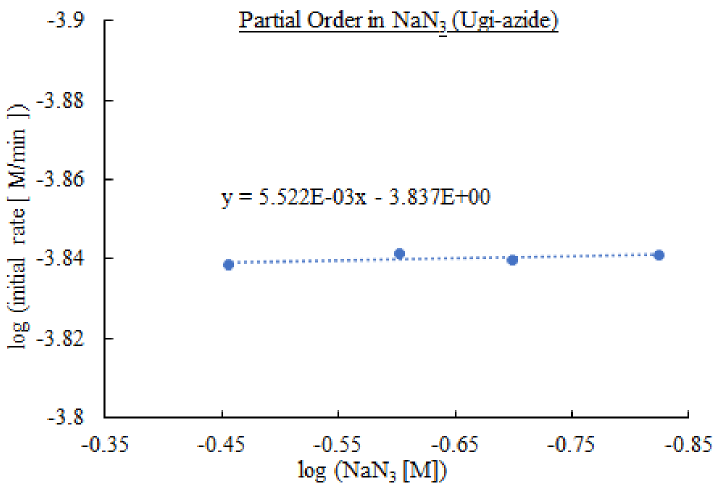

**1h** [M]	**2a** [M]	**3a** [M]	**4a** [M]	**5b** [M]	**NaN_3**	Initial rate [M/min]
0.005	0.075	0.050	0.150	0.200	0.150	1.4425×10^{-4}
0.005	0.075	0.050	0.150	0.200	0.200	1.4460×10^{-4}
0.005	0.075	0.050	0.150	0.200	0.250	1.4405×10^{-4}
0.005	0.075	0.050	0.150	0.200	0.350	1.4508×10^{-4}

Supplementary Figure 14 | Plot of initial rates *vs.* [NaN_3] and associated tabulated data.

2.7.8 $TMSN_3$ rate order on Ugi-azide reactions

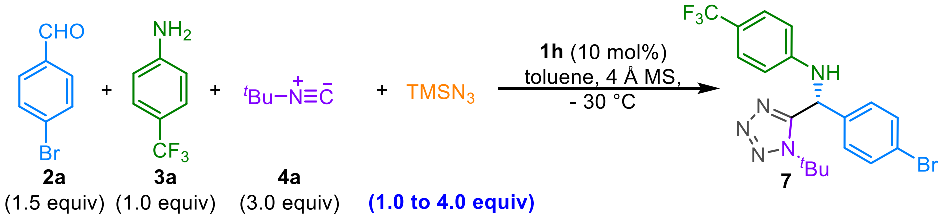

The rate order of $TMSN_3$ was evaluated following the general procedure, varying the starting-concentration of $TMSN_3$ between 3.0 eq and 7.0 eq. The quantitative results and graphs of these experiments are provided below, which also demonstrated first-order dependence with repect to $TMSN_3$.

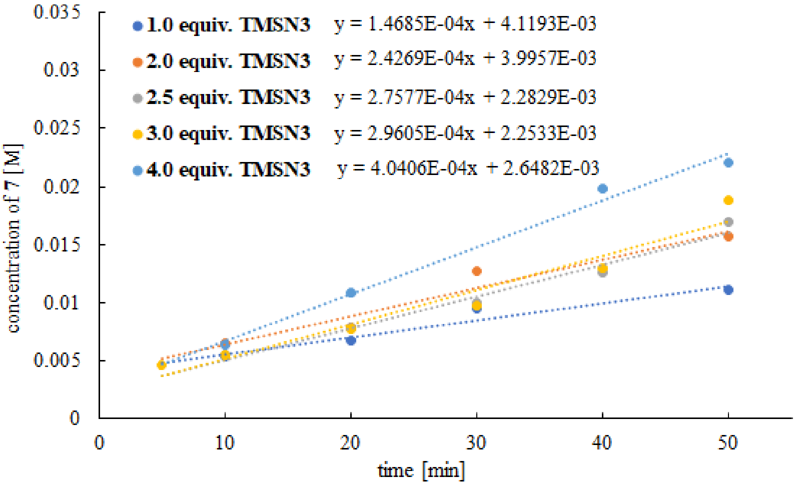

Supplementary Figure 15 | Rates determined from varying [$TMSN_3$].

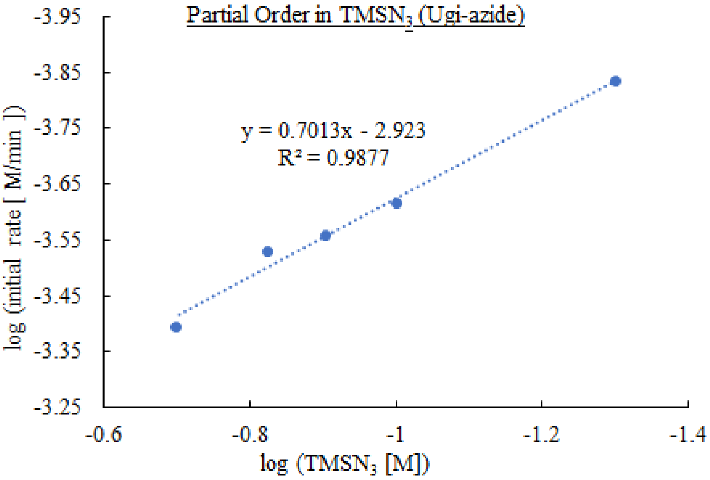

**1h** [M]	**2a** [M]	**3a** [M]	**4a** [M]	**$TMSN_3$** [M]	Initial rate [M/min]
0.005	0.075	0.050	0.150	0.050	1.4685×10^{-4}
0.005	0.075	0.050	0.150	0.100	2.4269×10^{-4}
0.005	0.075	0.050	0.150	0.125	2.7577×10^{-4}
0.005	0.075	0.050	0.150	0.150	2.9605×10^{-4}
0.005	0.075	0.050	0.150	0.200	4.0406×10^{-4}

Supplementary Figure 16 | Plot of initial rates *vs.* [$TMSN_3$] and associated tabulated data.

3. Supplementary Notes

3.1. Preparation of anionic stereogenic-at-cobalt(III) complexes

The catalysts (Λ-**1a**-Λ-**1p**) were known compounds and synthesized according to the literatures[1-4].

Procedures for alkali salts of anionic stereogenic-at-cobalt(III) complexes: In a 50 mL round-bottom flask was placed the 3,5-*di*-substituted salicylaldehyde (5 mmol), *L*-amino acid (5 mmol) and Na_2CO_3 (2.5 mmol) in EtOH (25 mL). The reaction mixture became homogeneous by heating at 90 °C for 6 h and then $Co(NO_3)_2{\cdot}6H_2O$ (2.7 mmol) was added. The resultant mixture was refluxed 42 h, and then filtered. The filtrate was concentrated in vacuo, and the residue was purified by column chromatography on neutral Al_2O_3 (EtOH as eluent, the Λ-(*S*,*S*)-complexes were always observed to have higher R_f values than the Δ-(*S*,*S*)-complexes). An additional purification was carried out by flash column chromatography on silica gel (Silica gel 60 extra pure was purchased from Merck KgaA, eluent: CH_2Cl_2 / MeOH = 10/1) to afford the major product Λ-**1b**, Λ-**1d**, Λ-**1f**, Λ-**1h**-Λ-**1p**.

Preparation of the acidic form: In a 50 mL round-bottom flask, H_2SO_4 (0.55 mmol, 18 M) was added to the sodium salt **1** (0.5 mmol) in CH_2Cl_2 (25 mL). Then the solution was allowed to warm to room temperature. The resultant mixture was vigorously stirred overnight and then filtered. The filtrate was washed with 3 M HCl (20 mL), H_2O (20 mL × 2) and the organic layers were dried over Na_2SO_4, and concentrated to afford the catalyst Λ-**1a**, Λ-**1c**, Λ-**1e**, and Λ-**1g**. It has been identified that the activity of the catalyst is the same as the Co(III)-templated Brønsted acids prepared by passing through a column filled with an ion-exchange resin DOWEX® 50WX8 in H^+-form (100-200 mesh).

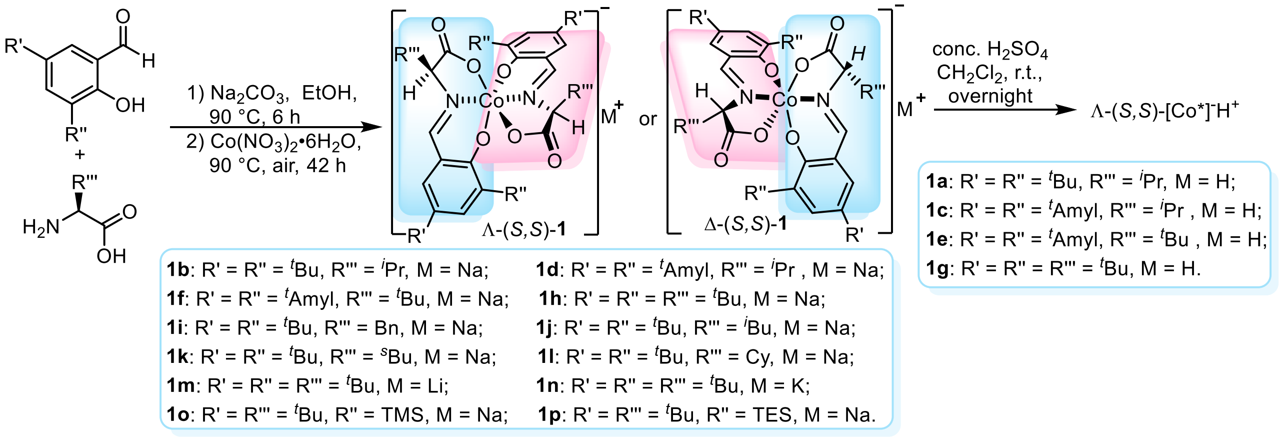

3.2. Experimental Procedures of Asymmetric Ugi-4CRs and Characteristic Data

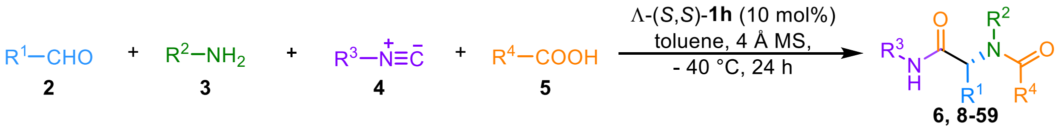

General Procedure A: Synthesis of racemic α-acylamino amides via Ugi-4CRs

To a 10-mL oven-dried tube was added aldehyde **2** (0.1 mmol), amine **3** (0.1 mmol), isocyanide **4** (0.1 mmol), acid **5** (0.1 mmol) and MeOH (1.0 mL). The solution was stirred overnight and then the solvent was removed under reduced pressure. The residue was purified by flash column chromatography to give the racemic α-acylamino amide.

General Procedure B: Synthesis of chiral α-acylamino amides via asymmetric Ugi-4CRs

A 10-mL oven-dried tube was charged with aldehyde **2** (0.15 mmol), amine **3** (0.10 mmol), catalyst Λ-**1h** (0.01 mmol), 4 Å molecular sieves (100 mg), and toluene (2.0 mL) at room temperature and stirred for 30 min. Then carboxylic acid **5** (0.50 mmol) was added. The mixture was cooled to -40 °C and stirred for another 30 min. The isocyanide **4** (0.30 mmol) was added in one portion and the resulting solution was stirred vigorously for 24 h. The reaction was then quenched with pre-cooled NEt_3 (-40 °C, 1.0 mmol). The mixture was purified by flash column chromatography (silica gel, petroleum ether/EtOAc/CH_2Cl_2 = 6:1:1) to give the enantioenriched α-acylamino amide.

(*R*)-*N*-(1-(4-Bromophenyl)-2-(tert-butylamino)-2-oxoethyl)-*N*-(4-(trifluoromethyl)phenyl)benzamide (6): yield: 52.8 mg (99%); (Flash column chromatography eluent, petroleum ether/ethyl acetate/dichloromethane = 6/1/1); slight yellow foam; $[\alpha]_D^{25}$ = -50.4 (c 0.21, MeOH); **^{1}H-NMR** (600 MHz, $CDCl_3$) δ 7.38 (d, *J* = 8.3 Hz, 2H), 7.30 – 7.25 (m, 4H), 7.22 (t, *J* = 7.3 Hz, 1H), 7.17 – 7.08 (m, 6H), 6.10 (s, 1H), 5.72 (s, 1H), 1.36 (s, 9H); **^{13}C-NMR** (151 MHz, $CDCl_3$) δ 171.1, 168.1, 144.4, 135.4, 133.7, 131.9, 131.7, 130.6, 130.1, 129.3 (q, *J* = 32.8 Hz), 128.6, 128.0, 125.6 (q, *J* = 2.7 Hz), 123.7 (q, *J* = 272.0 Hz), 123.0, 65.9, 52.0, 28.7; **^{19}F-NMR** (564 MHz, $CDCl_3$) δ -62.7; **HRMS** (ESI) calculated for $C_{26}H_{25}{}^{79}BrF_3N_2O_2$ $[M+H]^+$: 533.1046, found: 533.1048; **HRMS** (ESI) calculated for $C_{26}H_{25}{}^{81}BrF_3N_2O_2$ $[M+H]^+$: 535.1026, found: 535.1033; **Enantiomeric ratio**: 97.5:2.5, determined by HPLC (Daicel Chirapak IF, isopropanol / hexanel = 30/70, flow rate 1.0 mL/min, T = 30 ºC, λ = 254 nm): t_R = 4.53 min (major), t_R = 4.05 min (minor).

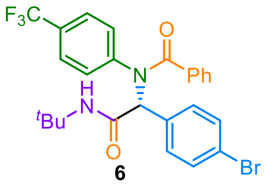

(*R*)-*N*-(1-(4-Bromophenyl)-2-(tert-butylamino)-2-oxoethyl)-*N*-(4-cyanophenyl)benzamide (8): yield: 48.6 mg (99%); (Flash column chromatography eluent, petroleum ether/ethyl acetate/dichloromethane = 6/1/1); slight yellow foam; $[\alpha]_D^{25}$ = -60.9 (c 0.20, MeOH); **^{1}H-NMR** (600 MHz, $CDCl_3$) δ 7.37 (d, *J* = 8.4 Hz, 2H), 7.28

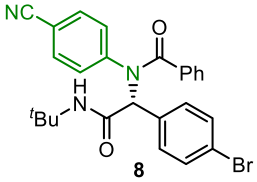

(d, J = 8.7 Hz, 2H), 7.27 – 7.25 (m, 1H), 7.25 – 7.23 (m, 2H), 7.18 – 7.07 (m, 6H), 6.16 (s, 1H), 5.64 (s, 1H), 1.36 (s, 9H); **^{13}C-NMR** (151 MHz, $CDCl_3$) δ 170.9, 168.0, 145.2, 135.2, 133.4, 132.3, 132.0, 131.7, 131.1, 130.2, 128.6, 128.1, 123.1, 118.1, 110.9, 65.3, 52.1, 28.7; **HRMS** (ESI) calculated for $C_{26}H_{25}{}^{79}BrN_3O_2$ $[M+H]^+$: 490.1125, found: 490.1129; **HRMS** (ESI) calculated for $C_{26}H_{25}{}^{81}BrN_3O_2$ $[M+H]^+$: 492.1104, found: 490.1113; **Enantiomeric ratio**: 97:3, determined by HPLC (Daicel Chirapak IF, isopropanol / hexanel = 30/70, flow rate 1.0 mL/min, T = 30 ºC, λ = 254 nm): t_R = 6.17 min (major), t_R = 5.37 min (minor).

(*R*)-methyl-4-(*N*-(1-(4-Bromophenyl)-2-(tert-butylamino)-2-oxoethyl)benzamido)benzoate (9):

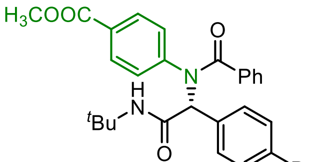

9

yield: 48.6 mg (93%); (Flash column chromatography eluent, petroleum ether/ethyl acetate/dichloromethane = 4/1/1); slight yellow foam; $[\alpha]_D^{25}$ = -49.0 (c 0.22, MeOH); **^{1}H-NMR** (600 MHz, $CDCl_3$) δ 7.68 (d, J = 7.5 Hz, 2H), 7.35 (d, J = 6.6 Hz, 2H), 7.27 (d, J = 7.4 Hz, 2H), 7.21 – 7.10 (m, 5H), 7.06 (d, J = 6.6 Hz, 2H), 6.12 (s, 1H), 5.76 (s, 1H), 3.82 (s, 3H), 1.37 (s, 9H); ^{13}**C-NMR** (151 MHz, $CDCl_3$) δ 171.1, 168.1, 166.3, 145.4, 135.6, 133.8, 131.9, 131.8, 130.1, 130.0, 129.9, 128.8, 128.6, 127.9, 122.9, 66.0, 52.2, 52.0, 28.8; **HRMS** (ESI) calculated for $C_{27}H_{28}{}^{79}BrN_2O_4$ $[M+H]^+$: 523.1227, found: 523.1230; **HRMS** (ESI) calculated for $C_{27}H_{28}{}^{81}BrN_2O_4$ $[M+H]^+$: 525.1206, found: 523.1215; **Enantiomeric ratio**: 91:9, determined by HPLC (Daicel Chirapak IF, isopropanol / hexanel = 30/70, flow rate 1.0 mL/min, T = 30 ºC, λ = 254 nm): t_R = 6.15 min (major), t_R = 5.30 min (minor).

(*R*)-*N*-(1-(4-Bromophenyl)-2-(tert-butylamino)-2-oxoethyl)-*N*-(4-nitrophenyl)benzamide (10):

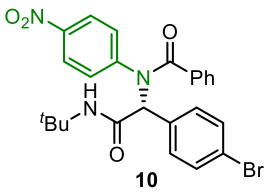

10

yield: 47.8 mg (94%); (Flash column chromatography eluent, petroleum ether/ethyl acetate/dichloromethane = 4/1/1); yellow foam; $[\alpha]_D^{25}$ = -79.0 (c 0.17, MeOH); **^{1}H-NMR** (600 MHz, $CDCl_3$) δ 7.85 (d, J = 9.1 Hz, 2H), 7.37 (d, J = 8.4 Hz, 2H), 7.27 (d, J = 7.2 Hz, 2H), 7.23 (d, J = 7.4 Hz, 1H), 7.18 – 7.14 (m, 4H), 7.12 (d, J = 8.4 Hz, 2H), 6.20 (s, 1H), 5.64 (s, 1H), 1.37 (s, 9H); **^{13}C-NMR** (151 MHz, $CDCl_3$) δ 171.0, 168.0, 147.0, 146.0, 135.1, 133.4, 132.1, 131.7, 131.1, 130.4, 128.6, 128.1, 123.7, 123.2, 65.3, 52.2, 28.7; **HRMS** (ESI) calculated for $C_{25}H_{25}{}^{79}BrN_3O_4$ $[M+H]^+$: 510.1023, found:510.1026; **HRMS** (ESI) calculated for $C_{25}H_{25}{}^{81}BrN_3O_4$ $[M+H]^+$: 512.1002, found: 512.1010; **Enantiomeric ratio**: 97.5:2.5, determined by HPLC (Daicel Chirapak ID, isopropanol / hexanel = 20/80, flow rate 1.0 mL/min, T = 30 ºC, λ = 254 nm): t_R = 8.09 min (major), t_R = 7.23 min (minor).

(*R*)-*N*-(1-(4-Bromophenyl)-2-(tert-butylamino)-2-oxoethyl)-*N*-(4-(methylsulfonyl)phenyl)ben-

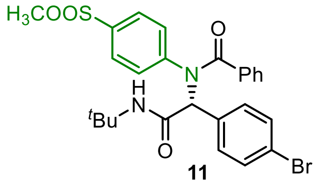

11

zamide (11): yield: 37.0 mg (68%); (Flash column chromatography eluent, petroleum ether/ethyl acetate/dichloromethane = 3/1/1); white foam; $[\alpha]_D^{25}$ = -38.8 (c 0.06, MeOH); **^{1}H-NMR** (600 MHz, $CDCl_3$) δ 7.57 (d, J = 8.7

Hz, 2H), 7.37 (d, *J* = 8.4 Hz, 2H), 7.27 (s, 2H), 7.24 – 7.17 (m, 3H), 7.15 – 7.12 (m, 4H), 6.11 (s, 1H), 5.72 (s, 1H), 2.90 (s, 3H), 1.36 (s, 9H); ^{13}**C-NMR** (151 MHz, $CDCl_3$) δ 171.1, 168.0, 146.3, 138.8, 135.1, 133.5, 132.0, 131.7, 131.1, 130.4, 128.6, 128.1, 127.7, 123.2, 65.9, 52.1, 44.5, 28.7; **HRMS** (ESI) calculated for $C_{26}H_{28}{}^{79}BrN_2O_4S$ [M+H]$^+$: 543.0948, found: 543.0953; **HRMS** (ESI) calculated for $C_{26}H_{28}{}^{81}BrN_2O_4S$ [M+H]$^+$: 545.0927, found: 545.0936; **Enantiomeric ratio**: 96.5:3.5, determined by HPLC (Daicel Chirapak ID, isopropanol / hexanel = 30/70, flow rate 1.0 mL/min, T = 30 ºC, λ = 254 nm): t_R = 10.36 min (major), t_R = 9.56 min (minor).

(*R*)-*N*-(4-Bromophenyl)-*N*-(1-(4-bromophenyl)-2-(tert-butylamino)-2-oxoethyl)benzamide (12):

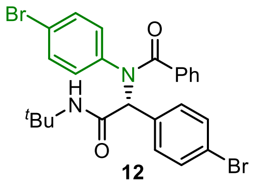

12

yield: 29.2 mg (54%); (Flash column chromatography eluent, petroleum ether/ethyl acetate/dichloromethane = 6/1/1); white foam; $[\alpha]_D^{25}$ = -22.8 (c 0.11, MeOH); ^{1}**H-NMR** (600 MHz, $CDCl_3$) δ 7.37 (d, *J* = 8.4 Hz, 2H), 7.26 (d, *J* = 7.6 Hz, 2H), 7.22 (t, *J* = 7.4 Hz, 1H), 7.17 – 7.10 (m, 6H), 6.86 (s, 2H), 6.08 (s, 1H), 5.69 (s, 1H), 1.36 (s, 9H); ^{13}**C-NMR** (151 MHz, $CDCl_3$) δ 171.2, 168.2, 140.0, 135.6, 133.8, 132.0, 131.9, 131.7, 129.9, 128.5, 127.9, 123.0, 121.3, 65.7, 52.0, 28.8; **HRMS** (ESI) calculated for $C_{25}H_{25}{}^{79}Br_2N_2O_2$ [M+H]$^+$: 543.0277, found: 543.0286; **HRMS** (ESI) calculated for $C_{25}H_{25}{}^{79}Br^{81}BrN_2O_2$ [M+H]$^+$: 545.0257, found: 545.0265; **HRMS** (ESI) calculated for $C_{25}H_{25}{}^{81}Br_2N_2O_2$ [M+H]$^+$: 547.0242, found: 547.0251; **Enantiomeric ratio**: 96:4, determined by HPLC (Daicel Chirapak IF, isopropanol / hexanel = 30/70, flow rate 1.0 mL/min, T = 30 ºC, λ = 254 nm): t_R = 4.99 min (major), t_R = 4.45 min (minor).

(*R*)-*N*-(1-(4-Bromophenyl)-2-(tert-butylamino)-2-oxoethyl)-*N*-(4-chlorophenyl)benzamide (13):

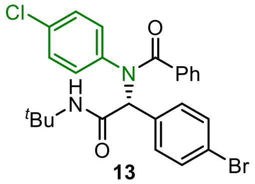

13

yield: 26.1 mg (52%); (Flash column chromatography eluent, petroleum ether/ethyl acetate/dichloromethane = 6/1/1); white foam; $[\alpha]_D^{25}$ = -110.2 (c 0.12, MeOH); ^{1}**H-NMR** (600 MHz, $CDCl_3$) δ 7.36 (d, *J* = 8.2 Hz, 2H), 7.27 – 7.25 (m, 2H), 7.20 (t, *J* = 7.3 Hz, 1H), 7.14 (d, *J* = 7.5 Hz, 2H), 7.11 (d, *J* = 8.5 Hz, 2H), 6.97 (d, *J* = 8.3 Hz, 2H), 6.92 (s, 2H), 6.09 (s, 1H), 5.76 (s, 1H), 1.35 (s, 9H); ^{13}**C-NMR** (151 MHz, $CDCl_3$) δ 171.2, 168.2, 139.4, 135.6, 133.8, 133.2, 131.9, 131.8, 131.7, 129.8, 128.7, 128.5, 127.9, 122.9, 65.6, 51.9, 28.7; **HRMS** (ESI) calculated for $C_{25}H_{25}{}^{79}Br^{35}ClN_2O_2$ [M+H]$^+$: 499.0782, found: 499.0788; **HRMS** (ESI) calculated for $C_{25}H_{25}{}^{81}Br^{35}ClN_2O_2$ [M+H]$^+$: 501.0762, found: 501.0769; **HRMS** (ESI) calculated for $C_{25}H_{25}{}^{81}Br^{37}ClN_2O_2$ [M+H]$^+$: 503.0738, found: 503.0749; **Enantiomeric ratio**: 96.5:3.5, determined by HPLC (Daicel Chirapak IF, isopropanol / hexanel = 10/90, flow rate 1.0 mL/min, T = 30 ºC, λ = 254 nm): t_R = 8.73 min (major), t_R = 7.59 min (minor).

(*R*)-*N*-(1-(4-Bromophenyl)-2-(tert-butylamino)-2-oxoethyl)-*N*-(3-(trifluoromethyl)phenyl)benzamide (14): yield: 40.4 mg (76%); (Flash column chromatography eluent, petroleum ether/ethyl acetate/dichloromethane = 6/1/1); slight yellow foam; $[\alpha]_D^{25}$ = -31.6 (c 0.05, MeOH); **^{1}H-NMR** (600 MHz, $CDCl_3$) δ 7.35 (d, *J* = 8.0 Hz, 2H), 7.24 (s, 2H), 7.22 – 7.16 (m, 3H), 7.16 – 7.05 (m, 6H), 6.13 (s, 1H), 5.73 (s, 1H), 1.36 (s, 9H); **^{13}C-NMR** (151 MHz, $CDCl_3$) δ 171.3, 168.1, 141.5, 135.4, 134.1, 133.7, 131.9,131.8 , 130.89 (q, *J* = 32.6 Hz), 129.9, 129.0, 128.5, 128.0, 127.37 (q, *J* = 3.7 Hz), 123.92 (q, *J* = 3.5 Hz), 123.3 (q, *J* = 282.2 Hz), 123.1, 65.6, 52.0, 28.7; **^{19}F-NMR** (564 MHz, $CDCl_3$) δ -63.0; **HRMS** (ESI) calculated for $C_{26}H_{25}{}^{79}BrF_3N_2O_2$ $[M+H]^+$: 533.1046, found: 533.1049; **HRMS** (ESI) calculated for $C_{26}H_{25}{}^{81}BrF_3N_2O_2$ $[M+H]^+$: 533.1026, found: 535.1031; **Enantiomeric ratio**: 91.5:8.5, determined by HPLC (Daicel Chirapak IF, isopropanol / hexanel = 30/70, flow rate 1.0 mL/min, T = 30 ºC, λ = 254 nm): t_R = 6.22 min (major), t_R = 5.89 min (minor).

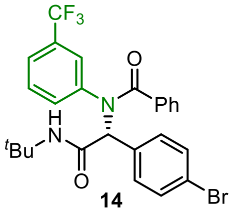

14

(*R*)-*N*-(1-(4-Bromophenyl)-2-(tert-butylamino)-2-oxoethyl)-4-(trifluoromethyl)-*N*-(4-(trifluoromethyl)phenyl)benzamide (15): yield: 32.2 mg (54%); (Flash column chromatography eluent, petroleum ether/ethyl acetate/dichloromethane = 6/1/1); slight yellow foam; $[\alpha]_D^{25}$ = -43.6 (c 0.08, MeOH); **^{1}H-NMR** (600 MHz, $CDCl_3$) δ 7.44 – 7.36 (m, 6H), 7.30 (d, *J* = 8.4 Hz, 2H), 7.13 – 7.12 (m, 2H), 7.10 (d, *J* = 8.4 Hz, 2H), 6.08 (s, 1H), 5.53 (s, 1H), 1.36 (s, 9H); **^{13}C-NMR** (151 MHz, $CDCl_3$) δ 169.7, 167.8, 143.6, 139.0, 133.3, 132.1, 131.9, 131.8 (q, *J* = 32.1 Hz) 130.9, 129.8 (q, *J* = 31.7 Hz), 128.9, 125.9 (q, *J* = 3.6 Hz), 125.1 (q, *J* = 3.6 Hz), 123.6 (q, *J* = 273.1 Hz), 123.5 (q, *J* = 272.1 Hz), 123.4, 65.9, 52.2, 28.8; **^{19}F-NMR** (564 MHz, $CDCl_3$) δ -63.1, -62.7; **HRMS** (ESI) calculated for $C_{27}H_{24}{}^{79}BrF_6N_2O_2$ $[M+H]^+$: 601.0925, found: 601.0928; **HRMS** (ESI) calculated for $C_{27}H_{24}{}^{81}BrF_6N_2O_2$ $[M+H]^+$: 603.0905, found: 603.0912; **Enantiomeric ratio**: 93:7, determined by HPLC (Daicel Chirapak IF, isopropanol / hexanel = 10/90, flow rate 1.0 mL/min, T = 30 ºC, λ = 254 nm): t_R = 5.54 min (major), t_{min} = 4.81 min (minor).

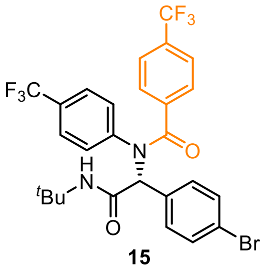

15

(*R*)-4-bromo-*N*-(1-(4-Bromophenyl)-2-(tert-butylamino)-2-oxoethyl)-*N*-(4-(trifluoromethyl)-phenyl)benzamide (16): yield: 36.5 mg (60%); (Flash column chromatography eluent, petroleum ether/ethyl acetate/dichloromethane = 6/1/1); slight yellow foam; $[\alpha]_D^{25}$ = -33.5 (c 0.12, MeOH); **^{1}H-NMR** (600 MHz, $CDCl_3$) δ 7.37 (d, *J* = 8.3 Hz, 2H), 7.33 – 7.26 (m, 4H), 7.14 (d, *J* = 8.4 Hz, 2H), 7.12 – 7.05 (m, 4H), 6.06 (s, 1H), 5.57 (s, 1H), 1.35 (s, 9H); **^{13}C-NMR** (151 MHz, $CDCl_3$) δ 170.0, 167.9, 144.1, 134.3, 133.5, 132.0, 131.8, 131.3, 130.7, 130.3, 129.6 (q, *J* = 32.9 Hz), 125.8 (q, *J* = 3.6 Hz), 124.7, 123.6 (q, *J* = 272.6 Hz), 123.2, 66.0, 52.1, 28.7; **^{19}F-NMR** (564 MHz, $CDCl_3$) δ -62.6; **HRMS** (ESI) calculated for $C_{26}H_{24}{}^{79}Br_2F_3N_2O_2$ $[M+H]^+$: 611.0151, found:

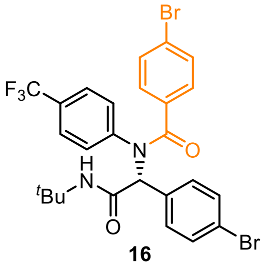

16

611.0154; $C_{26}H_{24}{}^{79}Br^{81}BrF_3N_2O_2$ $[M+H]^+$: 613.0131, found: 613.0137; $C_{26}H_{24}{}^{81}Br_2F_3N_2O_2$ $[M+H]^+$: 615.0116, found: 615.0120; **Enantiomeric ratio**: 91.5:8.5, determined by HPLC (Daicel Chirapak IF, isopropanol / hexanel = 30/70, flow rate 1.0 mL/min, T = 30 ºC, λ = 254 nm): t_R = 5.64 min (major), t_R = 5.11 min (minor).

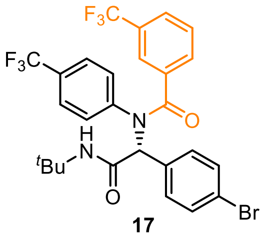

17

(R)-N-(1-(4-bromophenyl)-2-(tert-butylamino)-2-oxoethyl)-3-(trifluoromethyl)-N-(4-(trifluoromethyl)phenyl)benzamide (17): yield: 37.0 mg (61%); (Flash column chromatography eluent, petroleum ether/ethyl acetate/dichloromethane = 6/1/1); slight yellow foam; $[\alpha]_D^{25}$ = -47.9 (c 0.11, MeOH); **^{1}H-NMR** (600 MHz, $CDCl_3$) δ 7.52 (s, 1H), 7.46 (dd, *J* = 15.8, 7.8 Hz, 2H), 7.38 (d, *J* = 8.3 Hz, 2H), 7.32 – 7.26 (m, 3H), 7.18 – 7.09 (m, 4H), 6.09 (s, 1H), 5.53 (s, 1H), 1.37 (s, 9H); **^{13}C-NMR** (151 MHz, $CDCl_3$) δ 169.4, 167.8, 143.7, 136.2, 133.3, 132.1, 131.9, 131.8, 131.0, 130.6 (q, *J* = 33.0 Hz), 129.9 (q, *J* = 33.3 Hz), 128.6, 126.7 (q, *J* = 3.7 Hz), 125.84 – 125.71 (m), 123.55 (q, *J* = 272.3 Hz), 123.51 (q, *J* = 272.6 Hz), 123.4, 65.9, 52.2, 28.8; **^{19}F-NMR** (564 MHz, $CDCl_3$) δ -63.1, -62.8; **HRMS** (ESI) calculated for $C_{27}H_{24}{}^{79}BrF_6N_2O_2$ $[M+H]^+$: 601.0920, found: 601.0923; **HRMS** (ESI) calculated for $C_{27}H_{24}{}^{81}BrF_6N_2O_2$ $[M+H]^+$: 603.0899, found: 603.0908; **Enantiomeric ratio**: 96:4, determined by HPLC (Daicel Chirapak IF, isopropanol / hexanel = 10/90, flow rate 1.0 mL/min, T = 30 ºC, λ = 254 nm): t_R = 5.41 min (major), t_R = 4.92 min (minor).

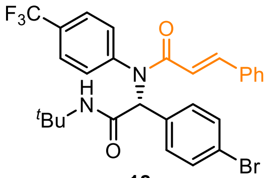

18

(*R*)-*N*-(1-(4-Bromophenyl)-2-(tert-butylamino)-2-oxoethyl)-*N*-(4-(trifluoromethyl)phenyl)cinnamamide (18): yield: 43.0 mg (77%); (Flash column chromatography eluent, petroleum ether/ethyl acetate/dichloromethane = 8/1/1); slight yellow foam; $[\alpha]_D^{25}$ = -33.3 (c 0.01, MeOH); **^{1}H-NMR** (600 MHz, $CDCl_3$) δ 7.72 (d, *J* = 15.5 Hz, 1H), 7.52 (d, *J* = 8.0 Hz, 2H), 7.34 (d, *J* = 8.4 Hz, 2H), 7.33 – 7.21 (m, 7H), 7.06 (d, *J* = 8.4 Hz, 2H), 6.10 (d, *J* = 15.5 Hz, 1H), 6.06 (s, 1H), 5.75 (s, 1H), 1.35 (s, 9H); **^{13}C-NMR** (151 MHz, $CDCl_3$) δ 168.2, 166.4, 143.8, 142.9, 134.8, 133.7, 131.9, 131.3, 130.5 (q, *J* = 20.1 Hz), 130.1, 128.8, 128.1, 126.2 (q, *J* = 3.1 Hz), 123.8 (q, *J* = 272.2 Hz), 123.1, 118.1, 64.9, 52.0, 28.8; **^{19}F-NMR** (564 MHz, $CDCl_3$) δ -62.6; **HRMS** (ESI) calculated for $C_{28}H_{27}{}^{79}BrF_3N_2O_2$ $[M+H]^+$: 559.1208, found: 559.1209; **HRMS** (ESI) calculated for $C_{28}H_{27}{}^{81}BrF_3N_2O_2$ $[M+H]^+$: 561.1188, found: 561.1194; **Enantiomeric ratio**: 93.5:6.5, determined by HPLC (Daicel Chirapak IF, isopropanol / hexanel = 20/80, flow rate 1.0 mL/min, T = 30 ºC, λ = 254 nm): t_R = 5.76 min (major), t_R = 5.33 min (minor).

(*R*)-*N*-(1-(4-Bromophenyl)-2-(tert-butylamino)-2-oxoethyl)-3-phenyl-*N*-(4-(trifluoromethyl)-phenyl)propanamide (19): yield: 50.0 mg (89%); (Flash column chromatography eluent, petroleum ether/ethyl acetate/dichloromethane = 8/1/1); slight yellow oil; $[\alpha]_D^{25}$ = -19.4 (c 0.23, MeOH); **^{1}H-**

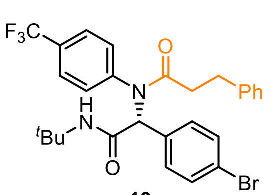

19

NMR (600 MHz, $CDCl_3$) δ 7.41 (s, 2H), 7.29 (d, *J* = 8.4 Hz, 2H), 7.22 (t, *J* = 7.3 Hz, 2H), 7.16 (t, *J* = 7.3 Hz, 1H), 7.03 (d, *J* = 7.1 Hz, 2H), 6.95 – 6.43 (m, 4H), 5.90 (s, 1H), 5.57 (d, *J* = 16.1 Hz, 1H), 2.90 (t, *J* = 7.6 Hz, 2H), 2.36 – 2.23 (m, 2H), 1.33 (s, 9H); **^{13}C-NMR** (151 MHz, $CDCl_3$) δ 172.4, 168.3, 142.9, 140.8, 133.5, 131.8, 131.7, 131.2, 130.4 (q, *J* = 33.7 Hz), 128.50, 128.47, 126.3, 126.1 (q, *J* = 6.7 Hz), 124.6, 123.7 (q, *J* = 272.4 Hz), 122.8, 64.5, 51.9, 36.8, 31.5, 28.7; **^{19}F-NMR** (564 MHz, $CDCl_3$) δ -62.7; **HRMS** (ESI) calculated for $C_{28}H_{29}{}^{79}BrF_3N_2O_2$ $[M+H]^+$: 561.1359, found: 561.1364; **HRMS** (ESI) calculated for $C_{28}H_{29}{}^{81}BrF_3N_2O_2$ $[M+H]^+$: 563.1339, found: 563.1348; **Enantiomeric ratio**: 94.5:5.5, determined by HPLC (Daicel Chirapak IB, isopropanol / hexanel = 20/80, flow rate 1.0 mL/min, T = 30 ºC, λ = 254 nm): t_R = 5.85 min (major), t_R = 6.41 min (minor).

(*R*)-*N*-(1-(4-Bromophenyl)-2-(tert-butylamino)-2-oxoethyl)-*N*-(4-(trifluoromethyl)phenyl)-cyclobutanecarboxamide (20): yield: 36.6 mg (72%); (Flash column chromatography eluent, petroleum ether/ethyl acetate/dichloromethane = 8/1/1); slight yellow oil; $[\alpha]_D^{25}$ = -30.2 (c 0.22, MeOH); **^{1}H-NMR** (600 MHz, $CDCl_3$) δ 7.44 (d, *J* = 8.0 Hz, 2H), 7.29 (d, *J* = 8.4 Hz, 2H), 7.22 – 7.01 (m, 2H), 6.97 (d, *J* = 8.4 Hz, 2H), 5.93 (s, 1H), 5.70 (s, 1H), 2.89 – 2.80 (m, 1H), 2.33 – 2.23 (m, 2H), 1.75 – 1.65 (m, 4H), 1.32 (s, 9H); **^{13}C-NMR** (151 MHz, $CDCl_3$) δ 174.9 , 168.4 , 142.8 , 133.7 , 131.9 , 131.7 , 131.4 , 130.4 (q, *J* = 32.6 Hz), 125.8 (q, *J* = 6.5 Hz), 123.78 (q, *J* = 272.3 Hz), 122.9 , 64.4 , 51.8 , 38.7 , 28.7 , 25.9 , 25.3 , 17.8; **^{19}F-NMR** (564 MHz, $CDCl_3$) δ -62.6; **HRMS** (ESI) calculated for $C_{24}H_{27}{}^{79}BrF_3N_2O_2$ $[M+H]^+$: 511.1208, found: 511.1211; **HRMS** (ESI) calculated for $C_{24}H_{27}{}^{81}BrF_3N_2O_2$ $[M+H]^+$: 513.1188, found: 513.1194; **Enantiomeric ratio**: 96:4, determined by HPLC (Daicel Chirapak IB, isopropanol / hexanel = 30/70, flow rate 1.0 mL/min, T = 30 ºC, λ = 254 nm): t_R = 3.90 min (major), t_R = 5.01 min (minor).

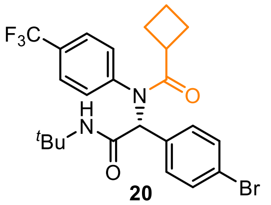

20

(*R*)-*N*-(1-(4-Bromophenyl)-2-(tert-butylamino)-2-oxoethyl)-*N*-(4-(trifluoromethyl)phenyl)-cyclopentanecarboxamide (21): yield: 37.5 mg (71%); (Flash column chromatography eluent, petroleum ether/ethyl acetate/dichloromethane = 8/1/1); slight yellow oil; $[\alpha]_D^{25}$ = -30.6 (c 0.19, MeOH); **^{1}H-NMR** (600 MHz, $CDCl_3$) δ 7.47 (s, 2H), 7.30 (d, *J* = 8.4 Hz, 2H), 7.27 – 7.04 (m, 2H), 6.98 (d, *J* = 8.4 Hz, 2H), 5.92 (s, 1H), 5.70 (s, 1H), 2.43 – 2.32 (m, 1H), 1.86 – 1.75 (m, 2H), 1.69 – 1.65 (m, 2H), 1.61 – 1.51 (m, 2H), 1.44 – 1.35 (m, 2H), 1.32 (s, 9H); **^{13}C-NMR** (151 MHz, $CDCl_3$) δ 177.1, 168.4, 143.4, 133.7, 131.9, 131.7, 131.4, 130.4 (q, *J* = 32.4 Hz), 126.0 (q, *J* = 6.7 Hz), 123.7 (q, *J* = 272.3 Hz), 122.9, 64.5, 51.8, 42.8, 31.3, 30.9, 28.7, 26.4, 26.3; **^{19}F-NMR** (564 MHz, $CDCl_3$) δ -62.6; **HRMS** (ESI) calculated for $C_{25}H_{29}{}^{79}BrF_3N_2O_2$ $[M+H]^+$: 525.1359, found: 525.1363; **HRMS** (ESI) calculated for $C_{25}H_{29}{}^{81}BrF_3N_2O_2$ $[M+H]^+$: 527.1339, found: 527.1345; **Enantiomeric ratio**: 94.5:5.5,

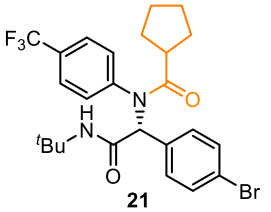

21

determined by HPLC (Daicel Chirapak IB, isopropanol / hexanel = 30/70, flow rate 1.0 mL/min, T = 30 ºC, λ = 254 nm): t_R = 3.61 min (major), t_R = 4.34 min (minor).

(*R*)-*N*-(1-(4-Bromophenyl)-2-(tert-butylamino)-2-oxoethyl)-*N*-(4-(trifluoromethyl)phenyl)cyclohexanecarboxamide (22): yield: 40.2 mg (75%); (Flash column chromatography eluent, petroleum ether/ethyl acetate/dichloromethane = 8/1/1); slight yellow oil; $[\alpha]_D^{25}$ = -28.2 (c 0.18, MeOH); **^{1}H-NMR** (600 MHz, $CDCl_3$) δ 7.48 (s, 2H), 7.30 (d, *J* = 8.4 Hz, 2H), 7.26 – 7.23 (m, 2H), 6.98 (d, *J* = 8.3 Hz, 2H), 5.91 (s, 1H), 5.69 (s, 1H), 2.01 – 1.92 (m, 1H), 1.69 – 1.45 (m, 7H), 1.32 (s, 9H), 1.20 – 1.11 (m, 1H), 0.98 – 0.84 (m, 2H); **^{13}C-NMR** (151 MHz, $CDCl_3$) δ 173.2, 168.4, 143.3, 133.6, 131.9, 131.8, 131.3, 130.4 (q, *J* = 32.9 Hz), 126.1 (q, *J* = 3.4 Hz), 123.7 (q, *J* = 274.3 Hz), 123.0, 64.4, 51.9, 36.9, 28.7, 18.7, 13.7; **^{19}F-NMR** (564 MHz, $CDCl_3$) δ -62.6; **HRMS** (ESI) calculated for $C_{26}H_{31}{}^{79}BrF_3N_2O_2$ $[M+H]^+$: 539.1516, found: 539.1519; **HRMS** (ESI) calculated for $C_{26}H_{31}{}^{81}BrF_3N_2O_2$ $[M+H]^+$: 541.1495, found: 541.1504; **Enantiomeric ratio**: 97:3, determined by HPLC (Daicel Chirapak IB, isopropanol / hexanel = 20/80, flow rate 1.0 mL/min, T = 30 ºC, λ = 254 nm): t_R = 4.54 min (major), t_R = 5.78 min (minor).

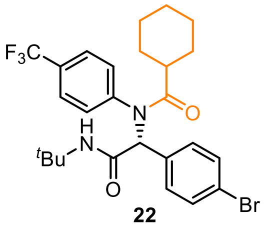

22

(*R*)-2-(4-Bromophenyl)-*N*-(tert-butyl)-2-(*N*-(4-(trifluoromethyl)phenyl)acetamido)acetamide (23): yield: 41.0 mg (87%); (Flash column chromatography eluent, petroleum ether/ethyl acetate/dichloromethane = 6/1/1); white foam; $[\alpha]_D^{25}$ = -37.0 (c 0.08, MeOH); **^{1}H-NMR** (600 MHz, $CDCl_3$) δ 7.48 (d, *J* = 8.1 Hz, 2H), 7.32 (d, *J* = 8.2 Hz, 2H), 7.26 - 7.24 (m, 2H), 6.98 (d, *J* = 8.2 Hz, 2H), 5.93 (s, 1H), 5.49 (s, 1H), 1.84 (s, 3H), 1.33 (s, 9H); **^{13}C-NMR** (151 MHz, $CDCl_3$) δ 170.7, 168.3, 143.7, 133.6, 131.90, 131.86, 131.2, 130.5 (q, *J* = 33.0 Hz), 126.2 (q, *J* = 3.5 Hz), 123.7 (q, *J* = 272.3 Hz), 123.1, 64.3, 52.0, 28.7, 23.4; **^{19}F-NMR** (564 MHz, $CDCl_3$) δ -62.7; **HRMS** (ESI) calculated for $C_{21}H_{23}{}^{79}BrF_3N_2O_2$ $[M+H]^+$: 471.0890, found: 471.0897; **HRMS** (ESI) calculated for $C_{21}H_{23}{}^{81}BrF_3N_2O_2$ $[M+H]^+$: 473.0869, found: 473.0878; **Enantiomeric ratio**: 95.5:4.5, determined by HPLC (Daicel Chirapak IC, isopropanol / hexanel = 20/80, flow rate 1.0 mL/min, T = 30 ºC, λ = 254 nm): t_R = 4.59 min (major), t_R = 4.16 min (minor).

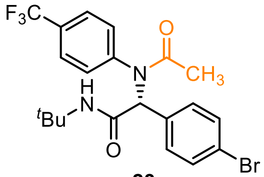

23

(*R*)-*N*-(1-(4-Bromophenyl)-2-(tert-butylamino)-2-oxoethyl)-*N*-(4-(trifluoromethyl)phenyl)propionamide (24): yield: 47.0 mg (97%); (Flash column chromatography eluent, petroleum ether/ethyl acetate/dichloromethane = 6/1/1); white foam; $[\alpha]_D^{25}$ = -92.6 (c 0.04, MeOH); **^{1}H-NMR** (600 MHz, $CDCl_3$) δ 7.53 – 7.40 (m, 2H),7.35 – 7.28 (m, 2H), 7.27 – 7.06 (m, 2H), 7.01 – 6.92 (m, 2H), 5.94 (d, *J* = 11.0 Hz, 1H), 5.63 (s, 1H), 2.05 – 1.91 (m, 2H), 1.31 (s, 9H), 1.02 (t, *J* = 7.4 Hz, 3H); **^{13}C-NMR** (151 MHz,

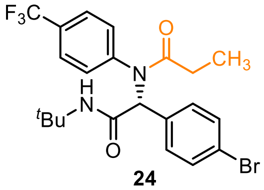

24

$CDCl_3$) δ 174.0, 168.4, 143.2, 133.7, 131.89, 131.77, 131.3, 130.4 (q, *J* = 32.6 Hz), 126.1 (q, *J* = 3.2 Hz), 123.72 (q, *J* = 272.3 Hz), 123.0, 64.3, 51.8, 28.7, 28.6, 9.4; **^{19}F-NMR** (564 MHz, $CDCl_3$) δ -62.7; **HRMS** (ESI) calculated for $C_{22}H_{25}{}^{79}BrF_3N_2O_2$ $[M+H]^+$: 485.1046, found: 485.1050; **HRMS** (ESI) calculated for $C_{22}H_{25}{}^{81}BrF_3N_2O_2$ $[M+H]^+$: 487.1026, found: 487.1033; **Enantiomeric ratio**: 93.5:6.5, determined by HPLC (Daicel Chirapak IB, isopropanol / hexanel = 30/70, flow rate 1.0 mL/min, T = 30 ºC, λ = 254 nm): t_R = 4.40 min (major), t_R = 5.00 min (minor).

(*R*)-*N*-(1-(4-Bromophenyl)-2-(tert-butylamino)-2-oxoethyl)-*N*-(4-(trifluoromethyl)phenyl)-butyramide (25): yield: 42.1 mg (84%); (Flash column chromatography eluent, petroleum ether/ethyl acetate/dichloromethane = 6/1/1); slight yellow oil; $[\alpha]_D^{25}$ = -15.5 (c 0.10, MeOH); **^{1}H-NMR** (600 MHz, $CDCl_3$) δ 7.47 (d, *J* = 7.2 Hz, 2H), 7.31 (d, *J* = 8.4 Hz, 2H), 7.25 – 7.10 (m, 2H), 6.98 (d, *J* = 8.4 Hz, 2H), 5.92 (s, 1H), 5.60 (s, 1H), 2.03 – 1.90 (m, 2H), 1.61 – 1.52 (m, 2H), 1.32 (s, 9H), 0.81 (t, *J* = 7.4 Hz, 3H); **^{13}C-NMR** (151 MHz, $CDCl_3$) δ 173.2, 168.4, 143.3, 133.7, 131.9, 131.8, 131.3, 130.4 (q, *J* = 32.8 Hz), 126.1 (q, *J* = 6.5 Hz), 123.8 (q, *J* = 272.4 Hz), 123.0, 64.4, 51.9, 36.9, 28.7, 18.7, 13.7; **^{19}F-NMR** (564 MHz, $CDCl_3$) δ -62.7; **HRMS** (ESI) calculated for $C_{23}H_{27}{}^{79}BrF_3N_2O_2$ $[M+H]^+$: 499.1208, found: 499.1209; **HRMS** (ESI) calculated for $C_{23}H_{27}{}^{79}BrF_3N_2O_2$ $[M+H]^+$: 501.1188, found: 501.1192; **Enantiomeric ratio**: 94:6, determined by HPLC (Daicel Chirapak IB, isopropanol / hexanel = 20/80, flow rate 1.0 mL/min, T = 30 ºC, λ = 254 nm): t_R = 4.86 min (major), t_R = 5.56 min (minor).

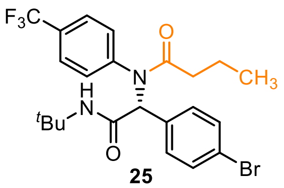
25

(*R*)-*N*-(1-(4-Bromophenyl)-2-(tert-butylamino)-2-oxoethyl)-*N*-(4-(trifluoromethyl)phenyl)pentanamide (26): yield: 49.8 mg (97%); (Flash column chromatography eluent, petroleum ether/ethyl acetate/dichloromethane = 6/1/1); slight yellow oil; $[\alpha]_D^{25}$ = -29.3 (c 0.04, MeOH); **^{1}H-NMR** (600 MHz, $CDCl_3$) δ 7.47 (d, *J* = 7.3 Hz, 2H), 7.30 (d, *J* = 8.4 Hz, 2H), 7.27 – 7.03 (m, 2H), 6.98 (d, *J* = 8.4 Hz, 2H), 5.93 (s, 1H), 5.66 (s, 1H), 2.06 – 1.91 (m, 2H), 1.59 – 1.46 (m, 2H), 1.32 (s, 9H), 1.22 – 1.14 (m, 2H), 0.78 (t, *J* = 7.4 Hz, 3H); **^{13}C-NMR** (151 MHz, $CDCl_3$) δ 173.4, 168.4, 143.3, 133.7, 131.9, 131.8, 131.3, 130.4 (q, *J* = 32.8 Hz), 126.1 (q, *J* = 6.8 Hz), 123.7 (q, *J* = 272.3 Hz), 123.0, 64.4, 51.8, 34.7, 28.7, 27.4, 22.3, 13.8; **^{19}F-NMR** (564 MHz, $CDCl_3$) δ -62.7; **HRMS** (ESI) calculated for $C_{24}H_{29}{}^{79}BrF_3N_2O_2$ $[M+H]^+$: 513.1359, found: 513.1360; **HRMS** (ESI) calculated for $C_{24}H_{29}{}^{81}BrF_3N_2O_2$ $[M+H]^+$: 515.1339, found: 515.1344; **Enantiomeric ratio**: 95:5, determined by HPLC (Daicel Chirapak IB, isopropanol / hexanel = 20/80, flow rate 1.0 mL/min, T = 30 ºC, λ = 254 nm): t_R = 5.45 min (major), t_R = 5.01 min (minor).

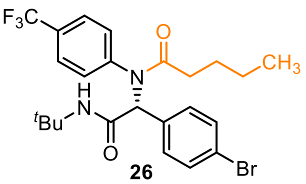
26

(*R*)-*N*-(1-(4-Bromophenyl)-2-(tert-butylamino)-2-oxoethyl)-*N*-(4-(trifluoromethyl)phenyl)pivalamide (27): yield: 29.7 mg (58%); (Flash column chromatography eluent, petroleum ether/ethyl acetate/dichloromethane = 6/1/1); slight yellow oil; $[\alpha]_D^{25}$ = -16.8 (c 0.06, MeOH); **^{1}H-NMR** (600 MHz, $CDCl_3$) δ 7.44 (d, *J* = 7.8 Hz, 2H), 7.31 – 7.26 (m, 4H), 6.96 (d, *J* = 8.4 Hz, 2H), 5.74 (s, 1H), 5.64 (s, 1H), 1.33 (s, 9H), 1.01 (s, 9H); **^{13}C-NMR** (151 MHz, $CDCl_3$) δ 178.1, 168.6, 143.9, 133.8, 132.7, 132.0, 131.6, 130.4 (q, *J* = 32.4 Hz), 125.3 (q, *J* = 7.0 Hz), 123.7 (q, *J* = 272.8 Hz), 122.9, 67.2, 51.7, 41.4, 29.5, 28.7; **^{19}F-NMR** (564 MHz, $CDCl_3$) δ -62.6; **HRMS** (ESI) calculated for $C_{24}H_{28}{}^{79}BrF_3N_2NaO_2$ $[M+Na]^+$: 535.1184, found: 535.1184; **HRMS** (ESI) calculated for $C_{24}H_{28}{}^{81}BrF_3N_2NaO_2$ $[M+Na]^+$: 537.1163, found: 537.1169; **Enantiomeric ratio**: 94:6, determined by HPLC (Daicel Chirapak IB, isopropanol / hexanel = 30/70, flow rate 1.0 mL/min, T = 30 ºC, λ = 254 nm): t_R = 7.62 min (major), t_R = 6.16 min (minor).

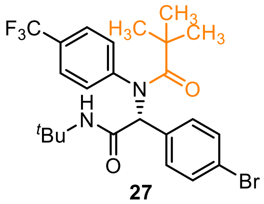

27

(*R*)-*N*-(1-(4-bromophenyl)-2-(tert-butylamino)-2-oxoethyl)-*N*-(4-(trifluoromethyl)phenyl)furan-2-carboxamide (28): yield: 42.7 mg (82%); (Flash column chromatography eluent, petroleum ether/ethyl acetate/dichloromethane = 5/1/1); colorless oil; $[\alpha]_D^{25}$ = -33.5 (c 0.20, MeOH); **^{1}H-NMR** (600 MHz, $CDCl_3$) δ 7.48 (d, *J* = 8.4 Hz, 2H), 7.36 (d, *J* = 8.4 Hz, 2H), 7.32 – 7.24 (m, 3H), 7.09 (d, *J* = 8.4 Hz, 2H), 6.21 (m, *J* = 3.6, 1.6 Hz, 1H), 6.04 (s, 1H), 5.83 (d, *J* = 3.5 Hz, 1H), 5.70 (s, 1H), 1.35 (s, 9H); **^{13}C-NMR** (151 MHz, $CDCl_3$) δ 167.93, 159.35, 146.48, 145.06, 143.29, 133.26, 132.01, 131.89, 131.37, 130.56 (q, *J* = 32.7 Hz), 125.90 (q, *J* = 3.6 Hz), 123.77 (q, *J* = 272.5 Hz), 123.22, 117.67, 111.34, 65.55, 52.00, 28.74; **^{19}F-NMR** (564 MHz, $CDCl_3$) δ -62.6; **HRMS** (ESI) calculated for $C_{24}H_{22}{}^{79}BrF_3N_2NaO_3$ $[M+Na]^+$: 545.0664, found: 545.0673; **HRMS** (ESI) calculated for $C_{24}H_{22}{}^{81}BrF_3N_2NaO_3$ $[M+Na]^+$: 547.0643, found:547.0656; **Enantiomeric ratio**: 90:10, determined by HPLC (Daicel Chirapak IF, isopropanol / hexanel = 30/70, flow rate 1.0 mL/min, T = 30 ºC, λ = 254 nm): t_R = 4.41 min (major), t_R = 4.07 min (minor).

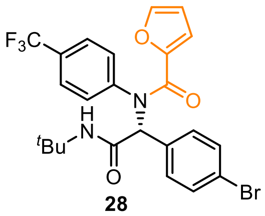

28

(2*E*,4*E*)-*N*-((*R*)-1-(4-bromophenyl)-2-(tert-butylamino)-2-oxoethyl)-*N*-(4-(trifluoromethyl)phenyl)hexa-2,4-dienamide (29): yield: 19.3 mg (37%); (Flash column chromatography eluent, petroleum ether/ethyl acetate/dichloromethane = 5/1/1); slight yellow oil; $[\alpha]_D^{25}$ = -33.2 (c 0.14, MeOH); **^{1}H-NMR** (600 MHz, $CDCl_3$) δ 7.49 (d, *J* = 8.2 Hz, 2H), 7.40 – 7.27 (m, 4H), 7.13 (m, *J* = 28.1, 8.5 Hz, 1H), 7.04 (d, *J* = 8.4 Hz, 2H), 6.09 (m, *J* = 14.9, 6.9 Hz, 1H), 6.02 (s, 1H), 6.01 – 5.96 (m, 1H), 5.75 (s, 1H), 5.44 (d, *J* = 14.8 Hz, 1H), 1.78 (d, *J* = 6.7 Hz, 3H), 1.34 (s, 9H); **^{13}C-NMR** (151 MHz, $CDCl_3$) δ 168.31, 166.79, 144.18, 143.04, 139.10, 133.78, 131.84, 131.78, 130.37 (q, *J* = 34.7 Hz), 131.27, 130.12, 126.05 (q, *J* = 3.0 Hz), 123.80 (q, *J* = 271.8 Hz), 122.95,

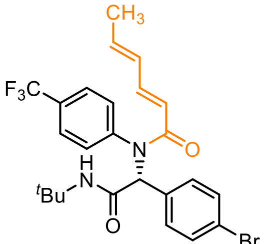

29

118.96, 64.67, 51.89, 28.76, 18.62; **^{19}F-NMR** (564 MHz, $CDCl_3$) δ -62.6; **HRMS** (ESI) calculated for $C_{25}H_{26}{}^{79}BrF_3N_2NaO_2$ $[M+Na]^+$: 545.1027, found: 545.1022; **HRMS** (ESI) calculated for $C_{25}H_{26}{}^{81}BrF_3N_2NaO_2$ $[M+Na]^+$: 547.1007, found: 547.1005; **Enantiomeric ratio**: 79:21, determined by HPLC (Daicel Chirapak IF, isopropanol / hexanel = 10/90, flow rate 1.0 mL/min, T = 30 ºC, λ = 254 nm): t_R = 5.76 min (major), t_{min} = 5.06 min (minor).

(*R*)-*N*-(2-(Tert-butylamino)-1-(2-chlorophenyl)-2-oxoethyl)-*N*-(4-(trifluoromethyl)phenyl)benzamide (30): yield: 47.2 mg (97%); (Flash column chromatography eluent, petroleum ether/ethyl acetate/dichloromethane = 6/1/1); white foam; $[\alpha]_D^{25}$ = -114.8 (c 0.16, MeOH); **^{1}H-NMR** (600 MHz, $CDCl_3$) δ 7.31 – 7.26 (m, 4H), 7.24 – 7.18 (m, 5H), 7.15 – 7.09 (m, 3H), 7.06 (t, *J* = 7.6 Hz, 1H), 6.57 (s, 1H), 5.80 (s, 1H), 1.40 (s, 9H); **^{13}C-NMR** (151 MHz, $CDCl_3$) δ 170.9, 168.4, 143.6, 135.7, 135.4, 132.5, 131.6, 130.6, 130.2, 129.8, 129.7, 129.0 (q, *J* = 32.0 Hz), 128.5, 127.9, 127.0, 125.1 (q, *J* = 6.8 Hz), 123.7 (q, *J* = 272.7 Hz), 62.8, 52.1, 28.7; **^{19}F-NMR** (564 MHz, $CDCl_3$) δ -62.7; **HRMS** (ESI) calculated for $C_{26}H_{25}{}^{35}ClF_3N_2O_2$ $[M+H]^+$: 489.1557, found: 489.1554; **HRMS** (ESI) calculated for $C_{26}H_{25}{}^{37}ClF_3N_2O_2$ $[M+H]^+$: 491.1527, found: 491.1538; **Enantiomeric ratio**: 93:7, determined by HPLC (Daicel Chirapak IF, isopropanol / hexanel = 10/90, flow rate 1.0 mL/min, T = 30 ºC, λ = 254 nm): t_R = 5.41 min (major), t_R = 5.91 min (minor).

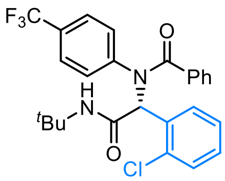

30

(*R*)-*N*-(1-(2-Bromophenyl)-2-(tert-butylamino)-2-oxoethyl)-*N*-(4-(trifluoromethyl)phenyl)benzamide (31): yield: 52.5 mg (98%); (Flash column chromatography eluent, petroleum ether/ethyl acetate/dichloromethane = 6/1/1); white solid; m.p.: 170.5 – 171.2 ˚C; $[\alpha]_D^{25}$ = -91.5 (c 0.18, MeOH); **^{1}H-NMR** (600 MHz, $CDCl_3$) δ 7.48 (d, *J* = 7.7 Hz, 1H), 7.31 – 7.24 (m, 4H), 7.20 – 7.16 (m, 3H), 7.15 – 7.03 (m, 5H), 6.49 (s, 1H), 5.66 (s, 1H), 1.39 (s, 9H); **^{13}C-NMR** (151 MHz, $CDCl_3$) δ 170.8, 168.4, 143.6, 135.7, 134.2, 133.2, 131.9, 130.7, 130.4, 129.8, 129.0 (q, *J* = 32.2 Hz), 128.5, 127.9, 127.6, 126.3, 125.1 (q, *J* = 6.8 Hz), 123.7 (q, *J* = 272.3 Hz), 65.2, 52.1, 28.7; **^{19}F-NMR** (564 MHz, $CDCl_3$) δ -62.7; **HRMS** (ESI) calculated for $C_{26}H_{25}{}^{79}BrF_3N_2O_2$ $[M+H]^+$: 533.1052, found: 533.1055; **HRMS** (ESI) calculated for $C_{26}H_{25}{}^{81}BrF_3N_2O_2$ $[M+H]^+$: 535.1031, found: 535.1043; **Enantiomeric ratio**: 91.5:8.5, determined by HPLC (Daicel Chirapak IF, isopropanol / hexanel = 20/80, flow rate 1.0 mL/min, T = 30 ºC, λ = 254 nm): t_R = 4.42 min (major), t_R = 4.76 min (minor).

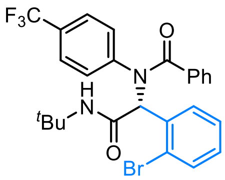

31

(*R*)-*N*-(2-(Tert-butylamino)-1-(3-chlorophenyl)-2-oxoethyl)-*N*-(4-(trifluoromethyl)phenyl)benzamide (32): yield: 48.4 mg (99%); (Flash column chromatography eluent, petroleum ether/ethyl acetate/dichloromethane = 6/1/1); slight yellow oil; $[\alpha]_D^{25}$ = -62.6 (c 0.22, MeOH); **^{1}H-NMR** (600 MHz, $CDCl_3$) δ 7.30 – 7.26 (m,

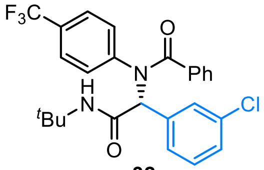

32

5H), 7.23 – 7.19 (m, 2H), 7.16 – 7.09 (m, 6H), 6.14 (s, 1H), 5.84 (s, 1H), 1.36 (s, 9H); **^{13}C-NMR** (151 MHz, $CDCl_3$) δ 171.1, 168.0, 144.3, 136.7, 135.4, 134.6, 130.7, 130.2, 130.1, 129.9, 129.3 (q, *J* = 32.8 Hz), 128.9, 128.6, 128.1, 128.0, 125.5 (q, *J* = 7.2 Hz), 123.7 (q, *J* = 272.2 Hz), 66.0, 52.0, 28.7; **^{19}F-NMR** (564 MHz, $CDCl_3$) δ -62.7; **HRMS** (ESI) calculated for $C_{26}H_{25}{}^{35}ClF_3N_2O_2$ $[M+H]^+$: 489.1557, found: 489.1554; **HRMS** (ESI) calculated for $C_{26}H_{25}{}^{37}ClF_3N_2O_2$ $[M+H]^+$: 491.1527, found: 491.1538; **Enantiomeric ratio**: 95:5, determined by HPLC (Daicel Chirapak IF, isopropanol / hexanel = 10/90, flow rate 1.0 mL/min, T = 30 ºC, λ = 254 nm): t_R = 6.60 min (major), t_R = 5.70 min (minor).

(*R*)-*N*-(2-(Tert-butylamino)-2-oxo-1-(3-(trifluoromethyl)phenyl)ethyl)-*N*-(4-(trifluoromethyl)-phenyl)benzamide (33): yield: 47.3 mg (91%); (Flash column chromatography eluent, petroleum ether/ethyl acetate/dichloromethane = 6/1/1); colorless oil; $[\alpha]_D^{25}$ = -84.8 (c 0.02, MeOH); **^{1}H-NMR** (600 MHz, $CDCl_3$) δ 7.50 – 7.40 (m, 3H), 7.34 (t, *J* = 7.7 Hz, 1H), 7.29 – 7.18 (m, 5H), 7.17 – 7.07 (m, 4H), 6.31 (s, 1H), 5.98 (s, 1H), 1.37 (s, 9H); **^{13}C-NMR** (151 MHz, $CDCl_3$) δ 171.2, 168.0, 144.0, 135.7, 135.3, 133.4, 131.1 (q, *J* = 33.8 Hz), 130.8, 130.2, 129.4 (q, *J* = 33.7 Hz), 129.1, 128.6, 128.0, 127.1 (q, *J* = 33.2 Hz), 125.6 (q, *J* = 3.7 Hz), 125.4 (q, *J* = 3.7 Hz), 123.7 (q, *J* = 272.4 Hz), 123.6 (q, *J* = 272.3 Hz), 65.6, 52.1, 28.7; **^{19}F-NMR** (564 MHz, $CDCl_3$) δ -63.0, -62.8; **HRMS** (ESI) calculated for $C_{27}H_{25}F_6N_2O_2$ $[M+H]^+$: 523.1815, found: 523.1815; **Enantiomeric ratio**: 95.5:4.5, determined by HPLC (Daicel Chirapak IF, isopropanol / hexanel = 10/90, flow rate 1.0 mL/min, T = 30 ºC, λ = 254 nm): t_R = 5.51 min (major), t_R = 5.16 min (minor).

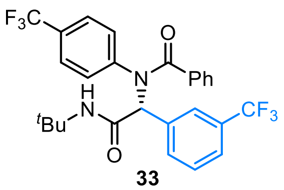

33

(*R*)-*N*-(2-(Tert-butylamino)-1-(4-fluorophenyl)-2-oxoethyl)-*N*-(4-(trifluoromethyl)phenyl)benzamide (34): yield: 46.7 mg (99%); (Flash column chromatography eluent, petroleum ether/ethyl acetate/dichloromethane = 6/1/1); white foam; $[\alpha]_D^{25}$ = -83.2 (c 0.19, MeOH); **^{1}H-NMR** (600 MHz, $CDCl_3$) δ 7.25 (t, *J* = 7.9 Hz, 4H), 7.22 – 7.18 (m, 3H), 7.14 – 7.09 (m, 4H), 6.90 (t, *J* = 8.5 Hz, 2H), 6.16 (s, 1H), 5.75 (s, 1H), 1.36 (s, 9H); **^{13}C-NMR** (151 MHz, $CDCl_3$) δ 171.1, 168.4, 163.6, 161.9, 144.3, 135.5, 132.0 (d, *J* = 8.3 Hz), 130.8, 130.0, 129.2 (q, *J* = 32.7 Hz), 128.6, 127.9, 125.5 (q, *J* = 6.7 Hz), 123.68 (q, *J* = 272.3 Hz), 115.7 (d, *J* = 21.5 Hz), 65.6, 51.9, 28.8; **^{19}F-NMR** (564 MHz, $CDCl_3$) δ -112.3, -62.7; **HRMS** (ESI) calculated for $C_{26}H_{25}F_4N_2O_2$ $[M+H]^+$: 473.1847, found: 473.1851; **Enantiomeric ratio**: 95:5, determined by HPLC (Daicel Chirapak IF, isopropanol / hexanel = 10/90, flow rate 1.0 mL/min, T = 30 ºC, λ = 254 nm): t_R = 6.85 min (najor), t_R = 6.34 min (minor).

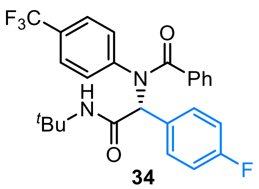

34

(*R*)-*N*-(2-(Tert-butylamino)-1-(4-cyanophenyl)-2-oxoethyl)-*N*-(4-(trifluoromethyl)phenyl)benzamide (35): yield: 47.5 mg (99%); (Flash column chromatography eluent, petroleum ether/ethyl

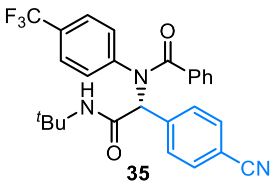
35

acetate/dichloromethane = 4/1/1); white foam; $[\alpha]_D^{25}$ = -53.4 (c 0.08, MeOH); **^{1}H-NMR** (600 MHz, $CDCl_3$) δ 7.53 (d, *J* = 8.1 Hz, 2H), 7.41 (d, *J* = 8.1 Hz, 2H), 7.30 – 7.22 (m, 5H), 7.15 (t, *J* = 7.6 Hz, 2H), 7.11 (d, *J* = 7.8 Hz, 2H), 6.18 (s, 1H), 5.95 (s, 1H), 1.36 (s, 9H); **^{13}C-NMR** (151 MHz, $CDCl_3$) δ 171.2, 167.5, 144.2, 139.9, 134.9, 132.3, 130.6, 130.4, 130.3, 129.5 (q, *J* = 33.7 Hz), 128.6, 128.1, 125.8 (q, *J* = 3.5 Hz), 123.53 (q, *J* = 272.2 Hz), 118.2, 112.6, 66.2, 52.2, 28.7; **^{19}F-NMR** (564 MHz, $CDCl_3$) δ -62.7; **HRMS** (ESI) calculated for $C_{27}H_{25}F_3N_3O_2$ $[M+H]^+$: 480.1893, found: 480.1896; **Enantiomeric ratio**: 95:5, determined by HPLC (Daicel Chirapak IB, isopropanol / hexanel = 20/80, flow rate 1.0 mL/min, T = 30 ºC, λ = 254 nm): t_R = 7.54 min (major), t_R = 11.72 min (minor).

(*R*)-*N*-(2-(Tert-butylamino)-1-(4-chlorophenyl)-2-oxoethyl)-*N*-(4-(trifluoromethyl)phenyl)benzamide (36): yield: 48.4 mg (99%); (Flash column chromatography eluent, petroleum ether/ethyl acetate/dichloromethane = 6/1/1); colorless oil; $[\alpha]_D^{25}$ = -86.8 (c 0.18, MeOH); **^{1}H-NMR** (600 MHz, $CDCl_3$) δ 7.29 – 7.25 (m, 4H), 7.22 – 7.16 (m, 5H), 7.15 – 7.10 (m, 4H), 6.15 (s, 1H), 5.77 (s, 1H), 1.37 (s, 9H); **^{13}C-NMR** (151 MHz, $CDCl_3$) δ 171.1, 168.2, 144.4, 135.4, 134.8, 133.2, 131.5, 130.7, 130.1, 129.3 (q, *J* = 32.7 Hz), 128.9, 128.6, 128.0, 125.6 (q, *J* = 3.5 Hz), 123.7 (q, *J* = 272.3 Hz), 65.8, 52.0, 28.7; **^{19}F-NMR** (564 MHz, $CDCl_3$) δ -62.7; **HRMS** (ESI) calculated for $C_{26}H_{25}{}^{35}ClF_3N_2O_2$ $[M+H]^+$: 489.1557, found: 489.1556; **HRMS** (ESI) calculated for $C_{26}H_{25}{}^{37}ClF_3N_2O_2$ $[M+H]^+$: 491.1527, found: 491.1540; **Enantiomeric ratio**: 97:3, determined by HPLC (Daicel Chirapak IF, isopropanol / hexanel = 10/90, flow rate 1.0 mL/min, T = 30 ºC, λ = 254 nm): t_R = 7.34 min (major), t_R = 6.35 min (minor).

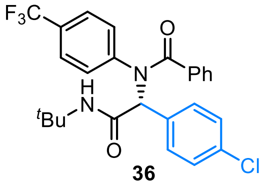
36

(*R*)-*N*-(2-(Tert-butylamino)-2-oxo-1-(p-tolyl)ethyl)-*N*-(4-(trifluoromethyl)phenyl)benzamide (37): yield: 41.0 mg (87%); (Flash column chromatography eluent, petroleum ether/ethyl acetate/dichloromethane = 6/1/1); colorless oil; $[\alpha]_D^{25}$ = -48.9 (c 0.03, MeOH); **^{1}H-NMR** (600 MHz, $CDCl_3$) δ 7.28 (d, *J* = 7.2 Hz, 2H), 7.23 (d, *J* = 8.5 Hz, 2H), 7.19 (t, *J* = 7.4 Hz, 1H), 7.15 – 7.07 (m, 6H), 7.03 (d, *J* = 7.9 Hz, 2H), 6.12 (s, 1H), 5.62 (s, 1H), 2.27 (s, 3H), 1.35 (s, 9H); **^{13}C-NMR** (151 MHz, $CDCl_3$) δ 171.0, 168.7, 144.7, 138.6, 135.8, 131.6, 130.8, 130.1, 129.8, 129.4, 128.9 (q, *J* = 32.6 Hz), 128.6, 127.9, 125.3 (q, *J* = 3.5 Hz), 123.8 (q, *J* = 272.1 Hz), 66.5, 51.8, 28.8, 21.2; **^{19}F-NMR** (564 MHz, $CDCl_3$) δ -62.6; **HRMS** (ESI) calculated for $C_{27}H_{28}F_3N_2O_2$ $[M+H]^+$: 469.2097, found: 469.2104; **Enantiomeric ratio**: 95:5, determined by HPLC (Daicel Chirapak IF, isopropanol / hexanel = 30/70, flow rate 1.0 mL/min, T = 30 ºC, λ = 254 nm): t_R = 4.83 min (major), t_R = 4.17 min (minor).

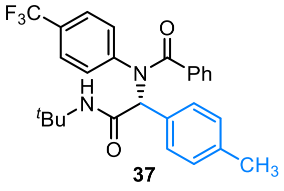
37

(*R*)-*N*-(2-(Tert-butylamino)-1-(4-(methylsulfonyl)phenyl)-2-oxoethyl)-*N*-(4-(trifluoromethyl)-phenyl)benzamide (38): yield: 50.5 mg (95%); (Flash column chromatography eluent, petroleum ether/ethyl acetate/dichloromethane = 4/1/1); white foam; $[\alpha]_D^{25}$ = -47.1 (c 0.17, MeOH); **^{1}H-NMR** (600 MHz, $CDCl_3$) δ 7.81 (d, *J* = 8.1 Hz, 2H), 7.51 (d, *J* = 8.1 Hz, 2H), 7.28 (t, *J* = 7.8 Hz, 4H), 7.26 – 7.24 (m, 1H), 7.18 – 7.10 (m, 4H), 6.21 (s, 1H), 6.01 (s, 1H), 2.97 (s, 3H), 1.37 (s, 9H); **^{13}C-NMR** (151 MHz, $CDCl_3$) δ 171.2, 167.5, 144.4, 140.9, 140.7, 134.9, 130.8, 130.5 ,130.3, 129.5 (q, *J* = 31.8 Hz), 128.6, 128.1, 127.6, 125.8 (q, *J* = 3.7 Hz), 123.53 (q, *J* = 272.1 Hz), 66.2, 52.2, 44.4, 28.7; **^{19}F-NMR** (564 MHz, $CDCl_3$) δ -62.7; **HRMS** (ESI) calculated for $C_{27}H_{28}F_3N_2O_4S$ $[M+H]^+$: 533.1716, found: 533.1721; **Enantiomeric ratio**: 94:6, determined by HPLC (Daicel Chirapak IF, isopropanol / hexanel = 20/80, flow rate 1.0 mL/min, T = 30 ºC, λ = 254 nm): t_R = 13.19 min (major), t_R = 11.18 min (minor).

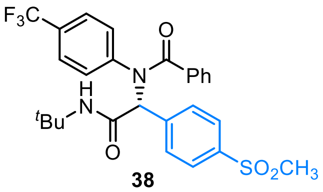

38

Methyl-(*R*)-4-(2-(Tert-butylamino)-2-oxo-1-(*N*-(4-(trifluoromethyl)phenyl)benzamido)ethyl)-benzoate (39): yield: 50.3 mg (98%); (Flash column chromatography eluent, petroleum ether/ethyl acetate/dichloromethane = 4/1/1); colorless oil; $[\alpha]_D^{25}$ = -36.9 (c 0.15, MeOH); **^{1}H-NMR** (600 MHz, $CDCl_3$) δ 7.89 (d, *J* = 8.2 Hz, 2H), 7.33 (d, *J* = 8.1 Hz, 2H), 7.29 – 7.18 (m, 5H), 7.16 – 7.08 (m, 4H), 6.21 (s, 1H), 5.80 (s, 1H), 3.87 (s, 3H), 1.35 (s, 9H); **^{13}C-NMR** (151 MHz, $CDCl_3$) δ 171.2, 168.0, 166.4, 144.4, 139.6, 135.3, 130.4, 130.5, 130.1, 130.0, 129.9, 129.2 (q, *J* = 32.4 Hz), 128.6, 128.0, 125.6 (q, *J* = 3.7 Hz), 123.6 (q, *J* = 272.2 Hz), 66.3, 52.3, 52.0, 28.7; **^{19}F-NMR** (564 MHz, $CDCl_3$) δ -62.7; **HRMS** (ESI) calculated for $C_{28}H_{28}F_3N_2O_4$ $[M+H]^+$: 513.1996, found: 513.2005; **Enantiomeric ratio**: 96:4, determined by HPLC (Daicel Chirapak IF, isopropanol / hexanel = 20/80, flow rate 1.0 mL/min, T = 30 ºC, λ = 254 nm): t_R = 6.94 min (major), t_R = 6.28 min (minor).

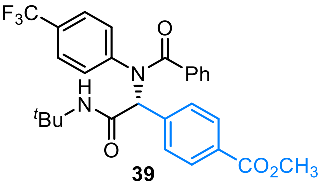

39

(*R*)-*N*-(2-(Tert-butylamino)-1-(4-methoxyphenyl)-2-oxoethyl)-*N*-(4-(trifluoromethyl)phenyl)-benzamide (40): yield: 40.4 mg (83%); (Flash column chromatography eluent, petroleum ether/ethyl acetate/dichloromethane = 6/1/1); colorless oil; $[\alpha]_D^{25}$ = -53.7 (c 0.13, MeOH); **^{1}H-NMR** (600 MHz, $CDCl_3$) δ 7.27 (d, *J* = 7.8 Hz, 2H), 7.24 (d, *J* = 8.5 Hz, 2H), 7.19 (t, *J* = 7.3 Hz, 1H), 7.15 - 7.05 (m, 6H), 6.74 (d, *J* = 8.5 Hz, 2H), 6.12 (s, 1H), 5.60 (s, 1H), 3.74 (s, 3H), 1.35 (s, 9H); **^{13}C-NMR** (151 MHz, $CDCl_3$) δ 171.0, 168.8, 159.8, 144.6 135.8 131.5, 130.9, 129.8, 128.9 (q, *J* = 32.1 Hz), 128.6, 127.9, 126.6, 125.3 (q *J* = 3.6 Hz), 123.8 (q, *J* = 272.3 Hz), 114.1, 65.9, 55.3, 51.8, 28.8; **^{19}F-NMR** (564 MHz, $CDCl_3$) δ -62.6; **HRMS** (ESI) calculated for $C_{27}H_{28}F_3N_2O_3$ $[M+H]^+$: 485.2047, found: 485.2051; **Enantiomeric ratio**: 93:7, determined by HPLC (Daicel Chirapak IF, isopropanol

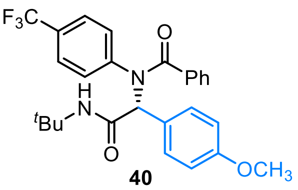

40

/ hexanel = 30/70, flow rate 1.0 mL/min, T = 30 °C, λ = 254 nm): t_R = 6.15 min (major), t_R = 6.56 min (minor).

(*R*)-*N*-(1-(4-Bromo-2-fluorophenyl)-2-(tert-butylamino)-2-oxoethyl)-*N*-(4-(trifluoromethyl)phenyl)benzamide (41): yield: 54.5 mg (99%); (Flash column chromatography eluent, petroleum ether/ethyl acetate/dichloromethane = 6/1/1); white foam; $[\alpha]_D^{25}$ = -104.5 (c 0.15, MeOH); **^{1}H-NMR** (600 MHz, $CDCl_3$) δ 7.27 (d, *J* = 8.4 Hz, 2H), 7.25 – 7.18 (m, 6H), 7.16 – 7.11 (m, 4H), 6.37 (s, 1H), 5.97 (s, 1H), 1.39 (s, 9H); **^{13}C-NMR** (151 MHz, $CDCl_3$) δ 170.9, 167.8, 161.5, 159.8, 143.9, 135.28, 132.44, 130.51, 130.05, 129.4 (q, *J* = 32.6 Hz), 128.53, 127.91, 125.53 (q, *J* = 3.4 Hz), 123.65 (q, *J* = 272.2 Hz), 123.49 (d, *J* = 9.6 Hz), 121.45 (d, *J* = 13.9 Hz), 119.13 (d, *J* = 25.1 Hz), 59.2, 52.0, 28.7; **^{19}F-NMR** (564 MHz, $CDCl_3$) δ -112.4, -62.7; **HRMS** (ESI) calculated for $C_{26}H_{24}{}^{79}BrF_4N_2O_2$ $[M+H]^+$: 551.0957, found: 551.0961; **HRMS** (ESI) calculated for $C_{26}H_{24}{}^{81}BrF_4N_2O_2$ $[M+H]^+$: 553.0937, found: 553.0945; **Enantiomeric ratio**: 94.5:5.5, determined by HPLC (Daicel Chirapak IB, isopropanol / hexanel = 10/90, flow rate 1.0 mL/min, T = 30 °C, λ = 254 nm): t_R = 5.95 min (major), t_R = 6.71 min (minor).

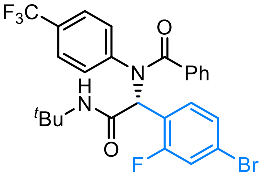

41

(*R*)-*N*-(2-(Tert-butylamino)-2-oxo-1-phenylethyl)-*N*-(4-(trifluoromethyl)phenyl)benzamide (42): yield: 44.9 mg (99%); (Flash column chromatography eluent, petroleum ether/ethyl acetate/dichloromethane = 6/1/1); colorless oil; $[\alpha]_D^{25}$ = -54.6 (c 0.02, MeOH); **^{1}H-NMR** (600 MHz, $CDCl_3$) δ 7.28 (d, *J* = 7.5 Hz, 2H), 7.24 – 7.17 (m, 8H), 7.15 – 7.08 (m, 4H), 6.17 (s, 1H), 5.68 (s, 1H), 1.36 (s, 9H); **^{13}C-NMR** (151 MHz, $CDCl_3$) δ 171.1, 168.5, 144.6, 135.7, 134.7, 130.8, 130.1, 129.9, 129.0 (q, *J* = 32.7 Hz), 128.7, 128.6, 127.9, 125.3 (q, *J* = 3.7 Hz), 123.7 (q, *J* = 272.0 Hz), 66.6, 51.9, 28.7; **^{19}F-NMR** (564 MHz, $CDCl_3$) δ -62.6; **HRMS** (ESI) calculated for $C_{26}H_{26}F_3N_2O_2$ $[M+H]^+$: 455.1941, found: 455.1946; **Enantiomeric ratio**: 96.5:3.5, determined by HPLC (Daicel Chirapak IF, isopropanol / hexanel = 30/70, flow rate 1.0 mL/min, T = 30 °C, λ = 254 nm): t_R = 4.36 min (major), t_R = 3.99 min (minor).

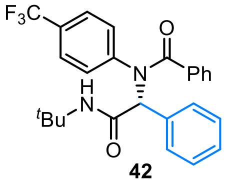

42

(*R*)-*N*-(2-(Tert-butylamino)-1-(naphthalen-2-yl)-2-oxoethyl)-*N*-(4-(trifluoromethyl)phenyl)benzamide (43): yield: 50.0 mg (99%); (Flash column chromatography eluent, petroleum ether/ethyl acetate/dichloromethane = 6/1/1); white foam; $[\alpha]_D^{25}$ = -52.1 (c 0.04, MeOH); **^{1}H-NMR** (600 MHz, $CDCl_3$) δ 7.80 (s, 1H), 7.79 – 7.73 (m, 2H), 7.68 (d, *J* = 8.5 Hz, 1H), 7.51 – 7.45 (m, 2H), 7.31 (d, *J* = 7.5 Hz, 2H), 7.26 – 7.25 (m, 1H), 7.22 – 7.11 (m, 7H), 6.36 (s, 1H), 5.76 (s, 1H), 1.37 (s, 9H); **^{13}C-NMR** (151 MHz, $CDCl_3$) δ 171.2, 168.5, 144.6, 135.7, 133.1, 133.0, 132.2, 130.8, 129.91, 129.87, 129.0 (q, *J* = 32.7 Hz), 128.6, 128.4, 128.1, 127.9, 127.7, 127.1, 126.9, 126.6, 125.4 (q, *J* = 3.5 Hz), 123.7 (q,

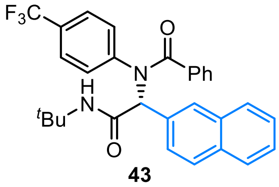

43

J = 272.1 Hz), 66.7, 51.9, 28.8; **^{19}F-NMR** (564 MHz, $CDCl_3$) δ -62.7; **HRMS** (ESI) calculated for $C_{30}H_{28}F_3N_2O_2$ $[M+H]^+$: 505.2097, found: 505.2103; **Enantiomeric ratio**: 95:5, determined by HPLC (Daicel Chirapak IF, isopropanol / hexanel = 30/70, flow rate 1.0 mL/min, T = 30 ºC, λ = 254 nm): t_R = 5.16 min (major), t_R = 4.46 min (minor).

(*R*)-*N*-(2-(Tert-butylamino)-2-oxo-1-(3-(trifluoromethyl)phenyl)ethyl)-*N*-(4-(trifluoromethyl)-phenyl)benzamide (44): yield: 28.4 mg (64%); (Flash column chromatography eluent, petroleum ether/ethyl acetate/dichloromethane = 6/1/1); yellow oil; $[\alpha]_D^{25}$ = -8.2 (c 0.09, MeOH); **^{1}H-NMR** (600 MHz, $CDCl_3$) δ 7.28 (d, J = 8.9 Hz, 2H), 7.27 – 7.17 (m, 6H), 7.17 – 7.07 (m, 4H), 6.48 (s, 1H), 6.30 (s, 1H), 1.39 (s, 9H); **^{13}C-NMR** (151 MHz, $CDCl_3$) δ 170.9, 166.4, 147.9, 144.4, 143.1, 133.7, 130.2, 129.9, 129.2 (q, J = 33.6 Hz), 128.9, 128.7, 128.5, 128.0, 125.5 (q, J = 3.6 Hz), 123.8 (q, J = 272.1 Hz), 112.5, 111.0, 60.3, 52.0, 28.7; **^{19}F-NMR** (564 MHz, $CDCl_3$) δ -62.6; **HRMS** (ESI) calculated for $C_{24}H_{24}F_3N_2O_3$ $[M+H]^+$: 445.1734, found: 445.1739; **Enantiomeric ratio**: 92.5:7.5, determined by HPLC (Daicel Chirapak IF, isopropanol / hexanel = 30/70, flow rate 1.0 mL/min, T = 30 ºC, λ = 254 nm): t_R = 5.93 min (major), t_R = 5.18 min (minor).

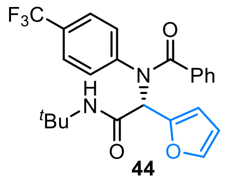

44

(*R*)-*N*-(2-(tert-butylamino)-1-(4-hydroxyphenyl)-2-oxoethyl)-*N*-(4-(trifluoromethyl)phenyl)benzamide (45): yield: 20.7 mg (44%); (Flash column chromatography eluent, petroleum ether/ethyl acetate/dichloromethane = 3/1/1); colorless oil; $[\alpha]_D^{25}$ = -14.8 (c 0.21, MeOH); **^{1}H-NMR** (600 MHz, $CDCl_3$)) δ 7.30 – 7.26 (m, 2H), 7.24 (s, 2H), 7.21 (t, J = 7.4 Hz, 1H), 7.14 (d, J = 7.7 Hz, 2H), 7.12 – 7.08 (m, 2H), 7.07 (d, J = 8.5 Hz, 2H), 6.70 (d, J = 8.6 Hz, 2H), 6.06 (s, 1H), 5.94 (s, 1H), 5.62 (s, 1H), 1.36 (s, 9H); **^{13}C-NMR** (151 MHz, $CDCl_3$) δ 171.23, 168.86, 156.35, 144.60, 135.68, 131.70, 130.86, 129.93, 129.07 (q, J = 32.8 Hz), 128.59, 127.93, 126.42, 125.40 (q, J = 3.7 Hz), 123.78 (q, J = 271.9 Hz), 115.75, 66.22, 51.93, 28.78; **^{19}F-NMR** (564 MHz, $CDCl_3$) δ -62.6; **HRMS** (ESI) calculated for $C_{26}H_{25}F_3N_2NaO_3$ $[M+Na]^+$: 493.1715, found: 493.1714; **Enantiomeric ratio**: 63.5:36.5, determined by HPLC (Daicel Chirapak IB, isopropanol / hexanel = 30/70, flow rate 1.0 mL/min, T = 30 ºC, λ = 254 nm): t_R = 3.80 min (major), t_R = 5.30 min (minor).

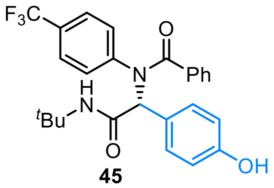

45

(*R*)-*N*-(2-(Tert-butylamino)-1-cyclohexyl-2-oxoethyl)-*N*-(4-(trifluoromethyl)phenyl)benzamide (46): yield: 25.0 mg (54%); (Flash column chromatography eluent, petroleum ether/ethyl acetate/dichloromethane = 10/1/1); colorless oil; $[\alpha]_D^{25}$ = -7.7 (c 0.09, MeOH); **^{1}H-NMR** (600 MHz, $CDCl_3$) δ 7.41 (d, J = 8.4 Hz, 2H), 7.28 – 2.22 (m, 3H), 7.21 – 7.13 (m, 4H), 6.83 (s, 1H), 4.49 (s, 1H), 2.17 (s, 1H), 1.85 (d, J = 11.4 Hz, 2H), 1.73 – 1.62 (m, 3H), 1.34 (s, 9H), 1.27 – 1.21 (m, 1H), 1.18 – 1.10 (m, 2H), 1.07 – 0.97 (m,

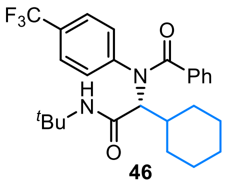

46

2H); **^{13}C-NMR** (151 MHz, $CDCl_3$) δ 172.1, 169.2, 136.0, 130.2, 129.1 (q, *J* = 33.1 Hz), 129.0, 128.5, 128.2, 125.9 (q, *J* = 3.5 Hz), 123.8 (q, *J* = 272.2 Hz), 113.1, 64.9, 51.4, 41.2, 36.3, 30.2, 28.8, 26.4, 25.7, 25.6; **^{19}F-NMR** (564 MHz, $CDCl_3$) δ -62.6; **HRMS** (ESI) calculated for $C_{26}H_{32}F_3N_2O_2$ $[M+H]^+$: 461.2410, found: 461.2415; **Enantiomeric ratio**: 81:19, determined by HPLC (Daicel Chirapak IA, isopropanol / hexanel = 30/70, flow rate 1.0 mL/min, T = 30 ºC, λ = 254 nm): t_R = 4.81 min (major), t_R = 5.59 min (minor).

(*R*)-*N*-(2-(Tert-butylamino)-1-cyclohexyl-2-oxoethyl)-*N*-(4-nitrophenyl)benzamide (47): yield: 32.1 mg (73%); (Flash column chromatography eluent, petroleum ether/ethyl acetate/dichloromethane = 10/1/1); yellow oil; $[\alpha]_D^{25}$ = -11.5 (c 0.06, MeOH); **^{1}H-NMR** (600 MHz, $CDCl_3$) δ 8.02 (d, *J* = 8.7 Hz, 2H), 7.35 (d, *J* = 8.5 Hz, 2H), 7.22 – 7.16 (m, 4H), 6.62 (s, 1H), 4.62 (s, 1H), 2.10 (s, 1H), 1.88 – 1.83 (m, 1H), 1.76 – 1.72 (m, 2H), 1.68 – 1.64 (m, 2H), 1.35 (s, 9H), 1.05 – 1.01 (m, 2H), 0.90 – 0.84 (m, 3H); **^{13}C-NMR** (151 MHz, $CDCl_3$) δ 172.0, 168. 9, 135.7, 130.6, 129.3, 128.5, 128.4, 126.4, 124.1, 111.8, 51.6, 36.2, 30.2, 28.8, 26.3, 25.7, 25.5; **HRMS** (ESI) calculated for $C_{25}H_{32}N_3O_4$ $[M+H]^+$: 438.2387, found: 438.2390; **Enantiomeric ratio**: 90:10, determined by HPLC (Daicel Chirapak ID, isopropanol / hexanel = 30/70, flow rate 1.0 mL/min, T = 30 ºC, λ = 254 nm): t_R = 6.74 min (major), t_R = 5.39 min (minor).

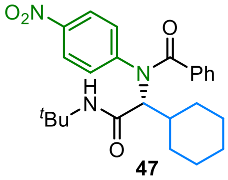

47

(*R*)-*N*-(2-(Tert-butylamino)-1-cyclohexyl-2-oxoethyl)-*N*-(4-cyanophenyl)benzamide (48): yield: 29.2 mg (70%); (Flash column chromatography eluent, petroleum ether/ethyl acetate/dichloromethane = 10/1/1); colorless oil; $[\alpha]_D^{25}$ = -9.9 (c 0.07, MeOH); **^{1}H-NMR** (600 MHz, $CDCl_3$) δ 7.44 (d, *J* = 8.4 Hz, 2H), 7.35 – 7.26 (m, 3H), 7.20 – 7.13 (m, 4H), 6.64 (s, 1H), 4.58 (s, 1H), 2.08 (s, 1H), 1.83 (d, *J* = 12.4 Hz, 1H), 1.73 (d, *J* = 9.0 Hz, 2H), 1.65 (d, *J* = 9.0 Hz, 2H), 1.33 (s, 9H), 1.29 – 1.20 (m, 2H), 1.20 – 1.03 (m, 3H); **^{13}C-NMR** (151 MHz, $CDCl_3$) δ 171.9, 168.9, 135.7, 132.6, 130.4, 129.4, 128.5, 128.3, 118.2, 110.6, 110.1, 51.5, 36.2, 30.1, 28.8, 26.3, 25.7, 25.5; **HRMS** (ESI) calculated for $C_{26}H_{32}N_3O_2$ $[M+H]^+$: 418.2489, found: 418.2494; **Enantiomeric ratio**: 91.5:8.5, determined by HPLC (Daicel Chirapak IB, isopropanol / hexanel = 30/70, flow rate 1.0 mL/min, T = 30 ºC, λ = 254 nm): t_R = 4.68 min (major), t_R = 3.98 min (minor).

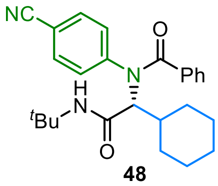

48

(*R*)-*N*-(1-(Tert-butylamino)-1-oxopentan-2-yl)-*N*-(4-cyanophenyl)benzamide (49): yield: 22.4 mg (59%); (Flash column chromatography eluent, petroleum ether/ethyl acetate/dichloromethane = 10/1/1); colorless oil; $[\alpha]_D^{25}$ = -68.1 (c 0.05, MeOH); **^{1}H-NMR** (600 MHz, $CDCl_3$) δ 7.45 (d, *J* = 8.7 Hz, 2H), 7.26 – 7.23 (m, 3H), 7.19 – 7.12 (m, 4H), 6.40 (s, 1H), 5.16 – 5.11 (m, 1H), 1.76 – 1.65 (m, 2H), 1.37

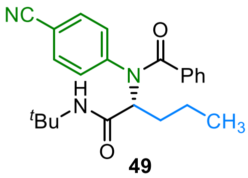

49

(s, 9H), 1.31 – 1.23 (m, 2H), 0.86 (t, J = 7.1 Hz, 3H); **^{13}C-NMR** (151 MHz, $CDCl_3$) δ 171.4, 169.7, 144.6, 135.6, 132.6, 130.2, 130.1, 128.2, 118.1, 111.1, 60.2, 51.6, 31.0, 28.8, 19.8, 13.9; **HRMS** (ESI) calculated for $C_{23}H_{28}N_3O_2$ $[M+H]^+$: 378.2176, found: 378.2177; **Enantiomeric ratio**: 82.5:17.5, determined by HPLC (Daicel Chirapak IF, isopropanol / hexanel = 20/80, flow rate 1.0 mL/min, T = 30 °C, λ = 254 nm): t_R = 7.50 min (major), t_R = 6.54 min (minor).

(*R*)-*N*-(1-(Tert-butylamino)-1-oxononan-2-yl)-*N*-(4-cyanophenyl)benzamide (50): yield: 26.6 mg (61%); (Flash column chromatography eluent, petroleum ether/ethyl acetate/dichloromethane = 10/1/1); colorless oil; $[\alpha]_D^{25}$ = -58.0 (c 0.09, MeOH); **^{1}H-NMR** (600 MHz, $CDCl_3$) δ 7.45 (d, J = 8.6 Hz, 2H), 7.25 – 7.22 (m, 3H), 7.19 – 7.12 (m, 4H), 6.39 (s, 1H), 5.12 (t, J = 7.5 Hz, 1H), 1.73 (d, J = 9.0 Hz, 1H), 1.37 (s, 9H), 1.31 – 1.14 (m, 9H), 0.83 (t, J = 7.1 Hz, 3H); **^{13}C-NMR** (151 MHz, $CDCl_3$) δ 171.4, 169.7, 144.5, 135.6, 132.6, 130.2, 130.1, 128.2, 118.1, 111.1, 110.1, 60.4, 51.6, 31.6, 29.0, 28.9, 28.8, 26.4, 22.5, 14.0; **HRMS** (ESI) calculated for $C_{27}H_{36}N_3O_2$ $[M+H]^+$: 434.2802, found: 434.2811; **Enantiomeric ratio**: 85:15, determined by HPLC (Daicel Chirapak IF, isopropanol / hexanel = 30/70, flow rate 1.0 mL/min, T = 30 °C, λ = 254 nm): t_R = 4.90 min (major), t_R = 4.49 min (minor).

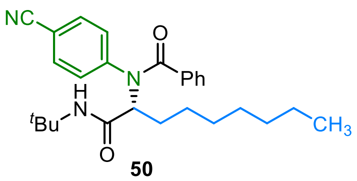

50

(*R*)-*N*-(1-(Tert-butylamino)-1-oxodecan-2-yl)-*N*-(4-cyanophenyl)benzamide (51): yield: 31.3 mg (70%); (Flash column chromatography eluent, petroleum ether/ethyl acetate/dichloromethane = 10/1/1); colorless oil; $[\alpha]_D^{25}$ = -80.0 (c 0.05, MeOH); **^{1}H-NMR** (600 MHz, $CDCl_3$) δ 7.45 (d, J = 8.5 Hz, 2H), 7.27 – 7.21 (m, 3H), 7.19 – 7.11 (m, 4H), 6.39 (s, 1H), 5.12 (t, J = 7.5 Hz, 1H), 1.76 – 1.68 (m, 1H), 1.36 (s, 9H), 1.33 – 1.10 (m, 13H), 0.84 (t, J = 7.1 Hz, 3H); **^{13}C-NMR** (151 MHz, $CDCl_3$) δ 171.4, 169.7, 144.5, 135.6, 132.6, 130.2, 130.1, 128.2, 118.1, 111.1, 60.4, 51.5, 31.8, 29.3, 29.2, 28.9, 28.8, 26.4, 25.4, 22.7, 14.2; **HRMS** (ESI) calculated for $C_{28}H_{38}N_3O_2$ $[M+H]^+$: 448.2959, found: 448.2964; **Enantiomeric ratio**: 85:15, determined by HPLC (Daicel Chirapak IF, isopropanol / hexanel = 30/70, flow rate 1.0 mL/min, T = 30 °C, λ = 254 nm): t_R = 4.77 min (major), t_R = 4.42 min (minor).

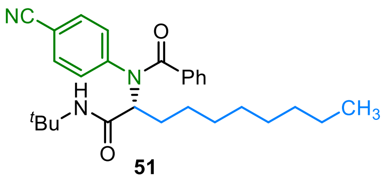

51

(*R*)-*N*-Butyl-*N*-(2-(Tert-butylamino)-1-(4-cyanophenyl)-2-oxoethyl)benzamide (52): yield: 20.1 mg (51%); (Flash column chromatography eluent, petroleum ether/ethyl acetate/dichloromethane = 10/1/1); colorless oil; $[\alpha]_D^{25}$ = -20.6 (c 0.07, MeOH); **^{1}H-NMR** (600 MHz, $CDCl_3$) δ 7.66 (d, J = 8.2 Hz, 2H), 7.56 (s, 2H), 7.47 – 7.38 (m, 5H), 6.74 (s, 1H), 5.54 (s, 1H), 3.41 – 3.26 (m, 2H), 1.38 (s, 9H), 1.22 – 1.18 (m, 2H), 1.07 – 0.91 (m, 2H), 0.64 (s, 3H); **^{13}C-NMR** (151 MHz, $CDCl_3$) δ 173.0,

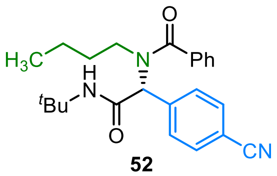

52

168.2, 141.3, 136.0, 132.4, 130.2, 129.2, 128.7, 126.7, 118.5, 112.1, 65.0, 51.8, 31.4, 28.7, 19.9, 13.4; **HRMS** (ESI) calculated for $C_{24}H_{30}N_3O_2$ $[M+H]^+$: 392.2333, found: 392.2342; **Enantiomeric ratio**: 60.5:39.5, determined by HPLC (Daicel Chirapak IF, isopropanol / hexanel = 30/70, flow rate 1.0 mL/min, T = 30 °C, λ = 254 nm): t_R = 7.43 min (major), t_R = 8.21 min (minor).

(*R*)-*N*-(1-(4-Bromophenyl)-2-(cyclohexylamino)-2-oxoethyl)-*N*-(4-(trifluoromethyl)phenyl)-benzamide (53): yield: 38.1 mg (68%); (Flash column chromatography eluent, petroleum ether/ethyl acetate/dichloromethane = 6/1/1); slight yellow foam; $[\alpha]_D^{25}$ = -19.8 (c 0.03, MeOH); **^{1}H-NMR** (600 MHz, $CDCl_3$) δ 7.39 (d, *J* = 8.4 Hz, 2H), 7.31 – 7.26 (m, 4H), 7.22 (t, *J* = 7.4 Hz, 1H), 7.18 – 7.07 (m, 6H), 6.16 (s, 1H), 5.70 (d, *J* = 7.8 Hz, 1H), 3.90 – 3.80 (m, 1H), 2.00 – 1.85 (m, 2H), 1.72 – 1.55 (m, 4H), 1.41 – 1.31 (m, 2H), 1.20 – 1.10 (m, 2H); **^{13}C-NMR** (151 MHz, $CDCl_3$) δ 171.1, 167.9, 144.3, 135.2, 133.5, 131.9, 131.7, 130.5, 130.0, 129.2 (q, *J* = 32.7 Hz), 128.5, 127.9, 125.5 (q, *J* = 3.7 Hz), 123.6 (q, *J* = 273.1 Hz), 123.0, 65.4, 49.0, 32.8, 25.4, 24.7; **^{19}F-NMR** (564 MHz, $CDCl_3$) δ -62.7; **HRMS** (ESI) calculated for $C_{28}H_{27}{}^{79}BrF_3N_2O_2$ $[M+H]^+$: 559.1208, found: 559.1210; **HRMS** (ESI) calculated for $C_{28}H_{27}{}^{81}BrF_3N_2O_2$ $[M+H]^+$: 561.1188, found: 561.1193; **Enantiomeric ratio**: 97:3, determined by HPLC (Daicel Chirapak IF, isopropanol / hexanel = 20/80, flow rate 1.0 mL/min, T = 30 °C, λ = 254 nm): t_R = 4.94 min (major), t_R = 5.65 min (minor).

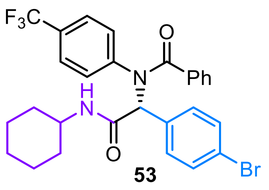

(*R*)-*N*-(2-(cyclohexylamino)-2-oxo-1-(4-(trifluoromethyl)phenyl)ethyl)-*N*-(4-(trifluoromethyl)-phenyl)benzamide (54): yield: 49.3 mg (90%); (Flash column chromatography eluent, petroleum ether/ethyl acetate/dichloromethane = 6/1/1); slight yellow foam; $[\alpha]_D^{25}$ = -67.7 (c 0.15, MeOH); **^{1}H-NMR** (600 MHz, $CDCl_3$) δ 7.52 (d, *J* = 8.2 Hz, 2H), 7.42 (d, *J* = 8.2 Hz, 2H), 7.32 – 7.26 (m, 4H), 7.24 – 7.21 (m, 1H), 7.18 – 7.08 (m, 4H), 6.24 (s, 1H), 5.85 (d, *J* = 7.6 Hz, 1H), 3.92 – 3.81 (m, 1H), 1.97 (d, *J* = 9.6 Hz, 1H), 1.90 (d, *J* = 10.6 Hz, 1H), 1.72 – 1.60 (m, 3H), 1.41 – 1.31 (m, 2H), 1.21 – 1.05 (m, 3H); **^{13}C-NMR** (151 MHz, $CDCl_3$) δ 171.2, 167.5, 144.4, 138.5, 135.0, 130.9 (q, *J* = 32.7 Hz), 130.3, 130.21, 130.17, 129.3 (q, *J* = 32.6 Hz), 128.5, 127.9, 125.6 (q, *J* = 3.8 Hz), 125.5 (q, *J* = 3.7 Hz), 123.5 (q, *J* = 272.3 Hz), 123.4 (q, *J* = 272.3 Hz), 65.9, 49.0, 32.8, 25.4, 24.7; **^{19}F-NMR** (564 MHz, $CDCl_3$) δ -62.9, -62.7; **HRMS** (ESI) calculated for $C_{29}H_{27}F_6N_2O_2$ $[M+H]^+$: 549.1971, found: 549.1976; **Enantiomeric ratio**: 98:2, determined by HPLC (Daicel Chirapak IF, isopropanol / hexanel = 30/70, flow rate 1.0 mL/min, T = 30 °C, λ = 254 nm): t_R = 4.22 min (major), t_R = 5.05 min (minor).

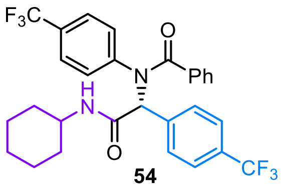

(*R*)-*N*-(1-(4-bromophenyl)-2-(cyclopentylamino)-2-oxoethyl)-*N*-(4-(trifluoromethyl)phenyl)benzamide (55): yield: 51.7 mg (95%); (Flash column chromatography eluent, petroleum ether/ethyl

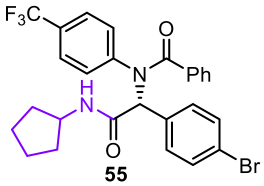

55

acetate/dichloromethane = 4/1/1); colorless oil; $[\alpha]_D^{25}$ = -54.6 (c 0.21, MeOH); **^{1}H-NMR** (600 MHz, $CDCl_3$) δ 7.39 (d, *J* = 8.5 Hz, 2H), 7.29 (d, *J* = 8.5 Hz, 2H), 7.27 – 7.25 (m, 2H), 7.25 – 7.20 (m, 1H), 7.14 (q, *J* = 8.1 Hz, 6H), 6.20 (s, 1H), 5.92 (d, *J* = 7.1 Hz, 1H), 4.27 (m, *J* = 13.6, 6.6 Hz, 1H), 2.04 – 1.95 (m, 2H), 1.64 – 1.56 (m, 4H), 1.44 – 1.39 (m, 1H), 1.34 (m, *J* = 11.2, 6.2 Hz, 1H); **^{13}C-NMR** (151 MHz, $CDCl_3$) δ 171.24, 168.48, 144.41, 135.33, 133.60, 131.97, 131.75, 130.67, 130.14, 129.34 (q, *J* = 32.8 Hz), 128.59, 127.99, 125.63 (q, *J* = 3.6 Hz), 123.67 (q, *J* = 272.2 Hz), 123.14, 65.48, 51.93, 33.04, 32.98, 23.81, 23.80; **^{19}F-NMR** (564 MHz, $CDCl_3$) δ -62.6; **HRMS** (ESI) calculated for $C_{27}H_{24}{}^{79}BrF_3N_2NaO_2$ $[M+Na]^+$: 567.0871, found: 567.0870; **HRMS** (ESI) calculated for $C_{27}H_{24}{}^{81}BrF_3N_2NaO_2$ $[M+Na]^+$: 569.0850, found: 569.0854; **Enantiomeric ratio**: 96:4, determined by HPLC (Daicel Chirapak IF, isopropanol / hexanel = 30/70, flow rate 1.0 mL/min, T = 30 ºC, λ = 254 nm): t_R = 4.81 min (major), t_R = 5.54 min (minor).

(*R*)-*N*-(1-(4-bromophenyl)-2-(isopropylamino)-2-oxoethyl)-*N*-(4-(trifluoromethyl)phenyl)benzamide (56): yield: 44.2 mg (85%); (Flash column chromatography eluent, petroleum ether/ethyl acetate/dichloromethane = 4/1/1); colorless oil; $[\alpha]_D^{25}$ = -37.8 (c 0.20, MeOH); **^{1}H-NMR** (600 MHz, $CDCl_3$) δ 7.40 (d, *J* = 8.5 Hz, 2H), 7.31 – 7.26 (m, 4H), 7.23 (m, *J* = 10.6, 4.3 Hz, 1H), 7.18 – 7.10 (m, 6H), 6.17 (s, 1H), 5.70 (d, *J* = 7.6 Hz, 1H), 4.16 (m, *J* = 11.5, 5.8 Hz, 1H), 1.19 (d, *J* = 6.6 Hz, 3H), 1.13 (d, *J* = 6.5 Hz, 3H); **^{13}C-NMR** (151 MHz, $CDCl_3$) δ 171.23, 168.02, 144.44, 135.35, 133.59, 132.00, 131.79, 130.67, 130.14, 129.35 (q, *J* = 32.8 Hz), 128.59, 128.00, 125.64 (q, *J* = 3.6 Hz), 123.68 (q, *J* = 272.4 Hz), 123.18, 65.55, 42.27, 22.62, 22.57; **^{19}F-NMR** (564 MHz, $CDCl_3$) δ -62.6; **HRMS** (ESI) calculated for $C_{25}H_{22}{}^{79}BrF_3N_2NaO_2$ $[M+Na]^+$: 541.0714, found:541.0716; **HRMS** (ESI) calculated for $C_{25}H_{22}{}^{81}BrF_3N_2NaO_2$ $[M+Na]^+$: 543.0694, found: 543.0698; **Enantiomeric ratio**: 96:4, determined by HPLC (Daicel Chirapak IF, isopropanol / hexanel = 30/70, flow rate 1.0 mL/min, T = 30 ºC, λ = 254 nm): t_R =4.44 min (major), t_R = 4.86 min (minor).

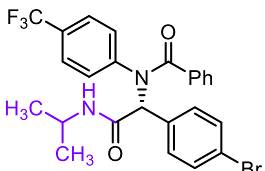

56

(*R*)-*N*-(1-(4-bromophenyl)-2-oxo-2-((2,4,4-trimethylpentan-2-yl)amino)ethyl)-*N*-(4-(trifluoromethyl)phenyl)benzamide (57): yield: 58.7 mg (99%); (Flash column chromatography eluent, petroleum ether/ethyl acetate/dichloromethane = 6/1/1); colorless oil; $[\alpha]_D^{25}$ = -65.3 (c 0.20, MeOH); **^{1}H-NMR** (600 MHz, $CDCl_3$) δ 7.39 (d, *J* = 8.5 Hz, 2H), 7.32 – 7.26 (m, 4H), 7.24 – 7.20 (m, 1H), 7.18 – 7.12 (m, 6H), 6.04 (s, 1H), 5.75 (s, 1H), 1.79 (d, *J* = 14.9 Hz, 1H), 1.61 (d, *J* = 14.9 Hz, 1H), 1.47 (s, 3H), 1.42 (s, 3H), 0.94 (s, 9H); **^{13}C-NMR** (151 MHz, $CDCl_3$) δ 171.07, 167.57, 144.71, 135.43, 133.75, 131.93, 131.76, 130.50, 130.06, 129.27 (q, *J* = 32.8 Hz), 128.56, 127.98, 125.66 (q, *J* = 3.6 Hz), 123.68 (q, *J* = 272.4 Hz), 123.06, 66.54, 56.10, 52.82, 31.53, 28.93, 28.53; **^{19}F-NMR**

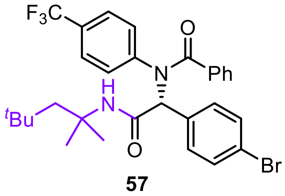

57

(564 MHz, $CDCl_3$) δ -62.6; **HRMS** (ESI) calculated for $C_{30}H_{32}{}^{79}BrF_3N_2NaO_2$ $[M+Na]^+$: 611.1497, found:611.1505; **HRMS** (ESI) calculated for $C_{30}H_{32}{}^{81}BrF_3N_2NaO_2$ $[M+Na]^+$: 613.1476, found: 613.1489; **Enantiomeric ratio**: 98:2., determined by HPLC (Daicel Chirapak IF, isopropanol / hexanel = 30/70, flow rate 1.0 mL/min, T = 30 °C, λ = 254 nm): t_R = 4.55 min (major), t_R = 3.81min (minor).

Ethyl (*R*)-(2-(4-bromophenyl)-2-(*N*-(4-(trifluoromethyl)phenyl)benzamido)acetyl)glycinate

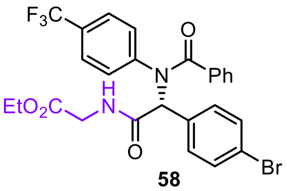

58

(58): yield: 26.4 mg (47%); (Flash column chromatography eluent, petroleum ether/ethyl acetate/dichloromethane = 3/1/1); colorless oil; $[\alpha]_D^{25}$ = -38.5 (c 0.20, MeOH); **¹H-NMR** (600 MHz, $CDCl_3$) δ 7.41 (d, *J* = 8.5 Hz, 2H), 7.35 – 7.27 (m, 4H), 7.24 (t, *J* = 7.4 Hz, 1H), 7.21 – 7.13 (m, 4H), 7.11 (d, *J* = 7.8 Hz, 2H), 6.52 (t, *J* = 4.9 Hz, 1H), 6.30 (s, 1H), 4.21 (q, *J* = 7.1 Hz, 2H), 4.11 (t, *J* = 5.0 Hz, 2H), 1.29 (d, *J* = 7.1 Hz, 3H); **¹³C-NMR** (151 MHz, $CDCl_3$) δ 171.4, 169.6, 169.2, 144.3, 135.2, 133.0, 132.0, 132.0, 130.6, 130.3, 129.5 (q, *J* = 32.8 Hz), 128.7, 128.0, 125.8 (q, *J* = 3.6 Hz), 123.7 (q, *J* = 272.4 Hz), 123.4, 65.4, 61.7, 41.8, 14.2; **¹⁹F-NMR** (564 MHz, $CDCl_3$) δ -62.7; **HRMS** (ESI) calculated for $C_{26}H_{22}{}^{79}BrF_3N_2NaO_4$ $[M+Na]^+$: 585.0613, found: 585.0609; **HRMS** (ESI) calculated for $C_{26}H_{22}{}^{81}BrF_3N_2NaO_4$ $[M+Na]^+$: 587.0592, found: 587.0594; **Enantiomeric ratio**: 78.5:21.5, determined by HPLC (Daicel Chirapak IF, isopropanol / hexanel = 30/70, flow rate 1.0 mL/min, T = 30 °C, λ = 254 nm): t_R = 7.20 min (major), t_R = 10.04 min (minor).

(*R*)-*N*-(1-(4-bromophenyl)-2-(butylamino)-2-oxoethyl)-*N*-(4-(trifluoromethyl)phenyl)benzamide

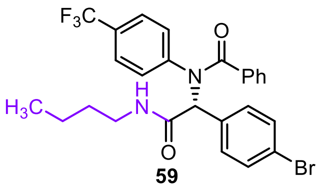

59

(59): yield: 36.7 mg (69%); (Flash column chromatography eluent, petroleum ether/ethyl acetate/dichloromethane = 4/1/1); colorless oil; $[\alpha]_D^{25}$ = -16.5 (c 0.19, MeOH); **¹H-NMR** (600 MHz, $CDCl_3$) δ 7.40 (d, *J* = 8.5 Hz, 2H), 7.33 – 7.27 (m, 4H), 7.25 – 7.21 (m, 1H), 7.18 – 7.11 (m, 6H), 6.17 (s, 1H), 5.92 (s, 1H), 3.37 – 3.30 (m, 2H), 1.49 (m, *J* = 15.0, 7.1 Hz, 2H), 1.33 – 1.29 (m, 2H), 0.90 (t, *J* = 7.4 Hz, 3H); **¹³C-NMR** (151 MHz, $CDCl_3$) δ 171.23, 168.86, 144.53, 135.30, 133.59, 132.01, 131.78, 130.62, 130.17, 129.39 (q, *J* = 32.6 Hz), 128.62, 128.01, 125.69 (q, *J* = 3.6 Hz), 123.68 (q, *J* = 272.3 Hz), 123.22, 65.77, 39.89, 31.57, 20.13, 13.74; **¹⁹F-NMR** (564 MHz, $CDCl_3$) δ -62.7; **HRMS** (ESI) calculated for $C_{26}H_{24}{}^{79}BrF_3N_2NaO_2$ $[M+Na]^+$:555.0871, found: 555.0875; **HRMS** (ESI) calculated for $C_{26}H_{24}{}^{81}BrF_3N_2NaO_2$ $[M+Na]^+$: 557.0850, found: 557.0859; **Enantiomeric ratio**: 96:4, determined by HPLC (Daicel Chirapak IF, isopropanol / hexanel = 30/70, flow rate 1.0 mL/min, T = 30 °C, λ = 254 nm): t_R = 4.96 min (major), t_R = 5.73 min (minor).

3.3. Experimental Procedures of Asymmetric Ugi-azide reactions and Characteristic Data

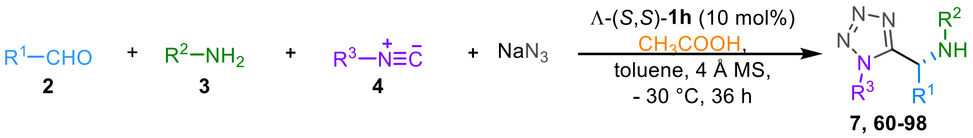

General Procedure C: Synthesis of racemic α-aminotetrazoles via Ugi-azide reactions

To a 10-mL oven-dried tube was added aldehyde **2** (0.1 mmol), amine **3** (0.1 mmol), isocyanide **4** (0.2 mmol), $TMSN_3$ (0.2 mmol) and MeOH (1.0 mL). The solution was stirred overnight and then the solvent was removed under reduced pressure. The residue was purified by flash column chromatography to give the racemic α-aminotetrazole.

General Procedure D: Synthesis of chiral α-aminotetrazoles via asymmetric Ugi-azide reactions

A 10-mL oven-dried tube was charged with aldehyde **2** (0.15 mmol), amine **3** (0.10 mmol), catalyst Λ-**1h** (0.01 mmol), NaN_3 (0.30 mmol), 4 Å molecular sieves (100 mg), and toluene (2.0 mL) at room temperature and stirred for 30 min. Then acetic acid **5b** (0.40 mmol) was added. The mixture was cooled to -30 °C and stirred for another 30 min. The isocyanide **4** (0.30 mmol) was added in one portion and the resulting solution was stirred vigorously for 36 h. The reaction was then quenched with pre-cooled NEt_3 (-30 °C, 1.0 mmol). The mixture was purified by flash column chromatography (silica gel, petroleum ether/EtOAc/CH_2Cl_2 = 6:1:1) to give the enantioenriched α-aminotetrazole.

(*R*)-*N*-((4-bromophenyl)(1-(*tert*-butyl)-1*H*-tetrazol-5-yl)methyl)-4-(trifluoromethyl)aniline (7):

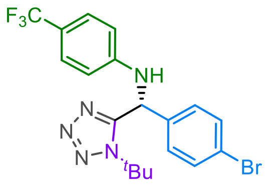

7

yield: 37.2 mg (82%); (Flash column chromatography eluent, petroleum ether/ethyl acetate/dichloromethane = 6/1/1); white solid; m.p.: 143.5 – 145.1 °C; $[\alpha]_D^{25}$ = -80.8 (c 0.38, MeOH); **^{1}H-NMR** (600 MHz, $CDCl_3$) δ 7.50 (d, *J* = 8.2 Hz, 2H), 7.40 (d, *J* = 8.3 Hz, 2H), 7.25 (d, *J* = 10.0 Hz, 2H), 6.66 (d, *J* = 8.4 Hz, 2H), 6.12 (d, *J* = 8.6 Hz, 1H), 5.30 (d, *J* = 8.6 Hz, 1H), 1.73 (s, 9H); **^{13}C-NMR** (151 MHz, $CDCl_3$) δ 154.3, 147.9, 136.5, 132.4, 129.29, 129.27, 126.9 (q, *J* = 3.8 Hz), 124.5 (q, *J* = 270.4 Hz), 123.1, 121.0 (q, *J* = 32.8 Hz), 114.2, 113.13, 113.10, 62.0, 53.3, 30.2; **^{19}F-NMR** (564 MHz, $CDCl_3$) δ -61.5; **HRMS** (ESI) calculated for $C_{19}H_{20}{}^{79}BrF_3N_5$ $[M+H]^+$: 454.0854, found: 454.0860; **HRMS** (ESI) calculated for $C_{19}H_{20}{}^{81}BrF_3N_5$ $[M+H]^+$: 456.0834, found: 456.0843; **Enantiomeric ratio**: 95.5:4.5, determined by HPLC (Daicel Chirapak IF, isopropanol / hexanel = 30/70, flow rate 1.0 mL/min, T = 30 ºC, λ = 254 nm): t_R = 5.26 min (major), t_R = 6.45 min (minor).

(*R*)-*N*-((1-(*tert*-butyl)-1H-tetrazol-5-yl)(4-fluorophenyl)methyl)-4-(trifluoromethyl)aniline (60): yield: 30.6 mg (78%); (Flash column chromatography eluent, petroleum ether/ethyl acetate/dichloromethane = 6/1/1); white foam; $[\alpha]_D^{25}$ = -108.4 (c 0.31, MeOH); **^{1}H-NMR** (600 MHz,

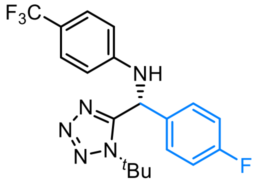

60

$CDCl_3$) δ 7.40 (d, *J* = 8.3 Hz, 2H), 7.37 – 7.32 (m, 2H), 7.06 (t, *J* = 8.4 Hz, 2H), 6.66 (d, *J* = 8.3 Hz, 2H), 6.15 (d, *J* = 8.6 Hz, 1H), 5.24 (d, *J* = 8.4 Hz, 1H), 1.72 (s, 9H); **^{13}C-NMR** (151 MHz, $CDCl_3$) δ 163.6, 162.0, 154.6, 148.0, 133.3, 129.5 (d, *J* = 8.4 Hz), 126.8 (q, *J* = 3.8 Hz), 124.5 (q, *J* = 270.9 Hz), 120.9 (q, *J* = 32.3 Hz), 116.3 (d, *J* = 21.9 Hz), 113.1, 61.9, 53.2, 30.1; **^{19}F-NMR** (564 MHz, $CDCl_3$) δ -112.2, -61.5; **HRMS** (ESI) calculated for $C_{19}H_{20}F_4N_5$ $[M+H]^+$: 394.1655, found: 394.1658; **Enantiomeric ratio**: 95:5, determined by HPLC (Daicel Chirapak IF, isopropanol / hexanel = 30/70, flow rate 1.0 mL/min, T = 30 °C, λ = 254 nm): t_R = 4.83 min (major), t_R = 5.97 min (minor).

(*R*)-*N*-((1-(*tert*-butyl)-1*H*-tetrazol-5-yl)(4-chlorophenyl)methyl)-4-(trifluoromethyl)aniline (61):

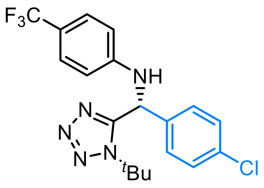

61

yield: 28.2 mg (69%); (Flash column chromatography eluent, petroleum ether/ethyl acetate/dichloromethane = 6/1/1); slight yellow foam; $[\alpha]_D^{25}$ = -92.8 (c 0.28, MeOH); **^{1}H-NMR** (600 MHz, $CDCl_3$) δ 7.40 (d, *J* = 8.4 Hz, 2H), 7.37 – 7.33 (m, 2H), 7.32 – 7.29 (m, 2H), 6.65 (d, *J* = 8.4 Hz, 2H), 6.14 (d, *J* = 8.6 Hz, 1H), 5.25 (d, *J* = 8.6 Hz, 1H), 1.73 (s, 9H); **^{13}C-NMR** (151 MHz, $CDCl_3$) δ 154.3, 147.9, 136.0, 135.0, 129.5, 129.0, 126.8 (q, *J* = 3.6 Hz), 124.5 (q, *J* = 270.5 Hz), 121.0 (q, *J* = 33.8 Hz), 113.1, 62.0, 53.2, 30.1; **^{19}F-NMR** (564 MHz, $CDCl_3$) δ -61.5; **HRMS** (ESI) calculated for $C_{19}H_{20}{}^{35}ClF_3N_5$ $[M+H]^+$: 410.1354, found: 410.1362; **HRMS** (ESI) calculated for $C_{19}H_{20}{}^{37}ClF_3N_5$ $[M+H]^+$: 412.1324, found: 412.1304; **Enantiomeric ratio**: 95:5, determined by HPLC (Daicel Chirapak IF, isopropanol / hexanel = 30/70, flow rate 1.0 mL/min, T = 30 °C, λ = 254 nm): t_R = 5.10 min (major), t_R = 6.18 min (minor).

(*R*)-4-((1-(*tert*-butyl)-1*H*-tetrazol-5-yl)((4(trifluoromethyl)phenyl)amino)methyl)benzonitrile

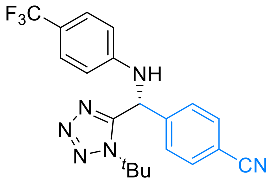

62

(62): yield: 30.1 mg (75%); (Flash column chromatography eluent, petroleum ether/ethyl acetate/dichloromethane = 3/1/1); white foam; $[\alpha]_D^{25}$ = -43.5 (c 0.30, MeOH); **^{1}H-NMR** (600 MHz, $CDCl_3$) δ 7.68 (d, *J* = 8.3 Hz, 2H), 7.53 (d, *J* = 8.2 Hz, 2H), 7.42 (d, *J* = 8.4 Hz, 2H), 6.65 (d, *J* = 8.4 Hz, 2H), 6.21 (d, *J* = 8.6 Hz, 1H), 5.31 (d, *J* = 8.5 Hz, 1H), 1.77 (s, 9H); **^{13}C-NMR** (151 MHz, $CDCl_3$) δ 153.7, 147.5, 142.6, 132.9, 128.3, 127.0 (q, *J* = 3.7 Hz), 124.4 (q, *J* = 270.9 Hz), 121.5 (q, *J* = 32.9 Hz), 117.9, 113.0, 62.2, 53.2, 30.2; **^{19}F-NMR** (564 MHz, $CDCl_3$) δ -61.5; **HRMS** (ESI) calculated for $C_{20}H_{20}F_3N_6$ $[M+H]^+$: 401.1702, found: 401.1707; **Enantiomeric ratio**: 97.5:2.5, determined by HPLC (Daicel Chirapak IB, isopropanol / hexanel = 20/80, flow rate 1.0 mL/min, T = 30 °C, λ = 254 nm): t_R = 10.88 min (major), t_R = 10.10 min (minor).

(*R*)-*N*-((1-(*tert*-butyl)-1*H*-tetrazol-5-yl)(4-nitrophenyl)methyl)-4-(trifluoromethyl)aniline (63): yield: 34.4 mg (82%); (Flash column chromatography eluent, petroleum ether/ethyl

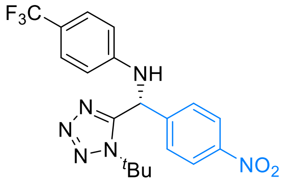

63

acetate/dichloromethane = 2/1/1); slight yellow foam; $[\alpha]_D^{25}$ = -70.1 (c 0.28, MeOH); **^{1}H-NMR** (600 MHz, $CDCl_3$) δ 8.24 (d, *J* = 8.7 Hz, 2H), 7.60 (d, *J* = 8.6 Hz, 2H), 7.42 (d, *J* = 8.4 Hz, 2H), 6.67 (d, *J* = 8.4 Hz, 2H), 6.26 (d, *J* = 8.7 Hz, 1H), 5.28 (d, *J* = 8.6 Hz, 1H), 1.78 (s, 9H); **^{13}C-NMR** (151 MHz, $CDCl_3$) δ 153.7, 148.1, 147.4, 144.4, 128.5, 127.0 (q, *J* = 3.8 Hz), 125.3 (q, *J* = 276.0 Hz), 124.4, 121.6 (q, *J* = 37.1 Hz), 113.1, 62.2, 53.0, 30.2; **^{19}F-NMR** (564 MHz, $CDCl_3$) δ -61.6; **HRMS** (ESI) calculated for $C_{19}H_{20}F_3N_6O_2$ $[M+H]^+$: 421.1594, found: 421.1597; **Enantiomeric ratio**: 97:3, determined by HPLC (Daicel Chirapak IF, isopropanol / hexanel = 30/70, flow rate 1.0 mL/min, T = 30 ºC, λ = 254 nm): t_R = 9.20 min (major), t_R = 6.77 min (minor).

(*R*)-*N*-((1-(*tert*-butyl)-1*H*-tetrazol-5-yl)(*p*-tolyl)methyl)-4-(trifluoromethyl)aniline (64): yield: 28.2 mg (72%); (Flash column chromatography eluent, petroleum ether/ethyl acetate/dichloromethane = 5/1/1); white foam; $[\alpha]_D^{25}$ = -157.7 (c 0.28, MeOH); **^{1}H-NMR** (600 MHz, $CDCl_3$) δ 7.38 (d, *J* = 8.4 Hz, 2H), 7.22 (d, *J* = 7.9 Hz, 2H), 7.17 (d, *J* = 7.9 Hz, 2H), 6.65 (d, *J* = 8.3 Hz, 2H), 6.11 (d, *J* = 8.5 Hz, 1H), 5.23 (d, *J* = 8.3 Hz, 1H), 2.33 (s, 3H), 1.70 (s, 9H); **^{13}C-NMR** (151 MHz, $CDCl_3$) δ 154.8, 148.3, 138.9, 134.4, 129.9, 127.6, 126.7 (q, *J* = 3.5 Hz), 124.6 (q, *J* = 269.5 Hz), 120.6 (q, *J* = 32.4 Hz), 113.0, 61.8, 53.7, 30.1, 21.1; **^{19}F-NMR** (564 MHz, $CDCl_3$) δ -61.4; **HRMS** (ESI) calculated for $C_{20}H_{23}F_3N_5$ $[M+H]^+$: 390.1900, found: 390.1907; **Enantiomeric ratio**: 93:7, determined by HPLC (Daicel Chirapak IF, isopropanol / hexanel = 30/70, flow rate 1.0 mL/min, T = 30 ºC, λ = 254 nm): t_R = 5.19 min (major), t_R = 9.33 min (minor).

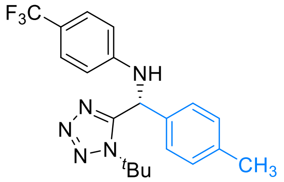

64

(*R*)-*N*-((1-(*tert*-butyl)-1*H*-tetrazol-5-yl)(4-methoxyphenyl)methyl)-4-(trifluoromethyl)aniline (65): yield: 31.8 mg (78%); (Flash column chromatography eluent, petroleum ether/ethyl acetate/dichloromethane = 4/1/1); colorless oil; $[\alpha]_D^{25}$ = -97.7 (c 0.32, MeOH); **^{1}H-NMR** (600 MHz, $CDCl_3$) δ 7.38 (d, *J* = 8.3 Hz, 2H), 7.26 (d, *J* = 8.5 Hz, 2H), 6.88 (d, *J* = 8.5 Hz, 2H), 6.65 (d, *J* = 8.3 Hz, 2H), 6.10 (d, *J* = 8.5 Hz, 1H), 5.24 (d, *J* = 8.4 Hz, 1H), 3.78 (s, 3H), 1.70 (s, 9H); **^{13}C-NMR** (151 MHz, $CDCl_3$) δ 159.9, 154.9, 148.3, 129.3, 129.0, 126.7 (q, *J* = 3.8 Hz), 123.7 (q, *J* = 270.1 Hz), 120.5 (q, *J* = 32.5 Hz), 114.6, 113.1, 61.8, 55.3, 53.4, 30.0; **^{19}F-NMR** (564 MHz, $CDCl_3$) δ -61.4; **HRMS** (ESI) calculated for $C_{20}H_{22}F_3N_5NaO$ $[M+Na]^+$: 428.1674, found: 428.1670; **Enantiomeric ratio**: 92.5:7.5, determined by HPLC (Daicel Chirapak IF, isopropanol / hexanel = 30/70, flow rate 1.0 mL/min, T = 30 ºC, λ = 254 nm): t_R = 6.36 min (major), t_R = 11.67 min (minor).

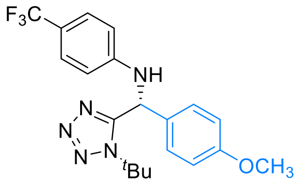

65

(*R*)-*N*-((1-(*tert*-butyl)-1*H*-tetrazol-5-yl)(4-(methylsulfonyl)phenyl)methyl)-4-(trifluoromethyl)-aniline (66): yield: 31.7 mg (70%); (Flash column chromatography eluent, petroleum ether/ethyl

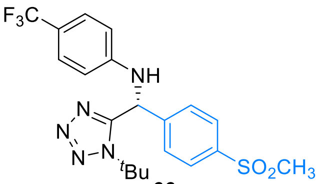
66

acetate/dichloromethane = 2/1/1); white foam; $[\alpha]_D^{25}$ = -67.9 (c 0.32, MeOH); **^{1}H-NMR** (600 MHz, $CDCl_3$) δ 7.93 (d, *J* = 8.3 Hz, 2H), 7.62 (d, *J* = 8.2 Hz, 2H), 7.40 (d, *J* = 8.4 Hz, 2H), 6.68 (d, *J* = 8.4 Hz, 2H), 6.25 (d, *J* = 8.6 Hz, 1H), 5.51 (d, *J* = 8.4 Hz, 1H), 3.03 (s, 3H), 1.78 (s, 9H); **^{13}C-NMR** (151 MHz, $CDCl_3$) δ 153.8, 147.6, 143.6, 141.0, 128.5, 128.2, 127.0 (q, *J* = 3.8 Hz), 124.4 (q, *J* = 270.8 Hz), 121.2 (q, *J* = 32.8 Hz), 113.0, 62.3, 53.0, 44.3, 30.2; **^{19}F-NMR** (564 MHz, $CDCl_3$) δ -61.5; **HRMS** (ESI) calculated for $C_{20}H_{22}F_3N_5NaO_2S$ $[M+Na]^+$: 476.1344, found: 476.1338; **Enantiomeric ratio**: 99:1, determined by HPLC (Daicel Chirapak IF, isopropanol / hexanel = 30/70, flow rate 1.0 mL/min, T = 30 ºC, λ = 254 nm): t_R = 6.07 min (major), t_R = 7.83 min (minor).

(*R*)-*N*-((1-(*tert*-butyl)-1*H*-tetrazol-5-yl)(phenyl)methyl)-4-(trifluoromethyl)aniline (67): yield:

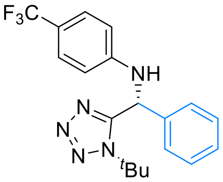
67

23.6 mg (63%); (Flash column chromatography eluent, petroleum ether/ethyl acetate/dichloromethane = 6/1/1); slight yellow oil; $[\alpha]_D^{25}$ = -77.2 (c 0.24, MeOH); **^{1}H-NMR** (600 MHz, $CDCl_3$) δ 7.2-7.32 (m, 7H), 6.67 (d, *J* = 8.4 Hz, 2H), 6.16 (d, *J* = 8.8 Hz, 1H), 5.22 (d, *J* = 8.7 Hz, 1H), 1.71 (s, 9H); **^{13}C-NMR** (151 MHz, $CDCl_3$) δ 154.7, 148.2, 137.4, 129.3, 129.0, 127.7, 126.8 (q, *J* = 3.8 Hz), 124.6 (q, *J* = 270.9 Hz), 120.7 (q, *J* = 32.7 Hz), 113.1, 61.9, 54.0, 30.1; **^{19}F-NMR** (564 MHz, $CDCl_3$) δ -61.4; **HRMS** (ESI) calculated for $C_{19}H_{20}F_3N_5Na$ $[M+Na]^+$: 398.1568, found: 398.1568; **Enantiomeric ratio**: 91:9, determined by HPLC (Daicel Chirapak IF, isopropanol / hexanel = 30/70, flow rate 1.0 mL/min, T = 30 ºC, λ = 254 nm): t_R = 4.87 min (major), t_R = 6.13 min (minor).

(*R*)-*N*-((2-bromophenyl)(1-(*tert*-butyl)-1*H*-tetrazol-5-yl)methyl)-4-(trifluoromethyl)aniline (68):

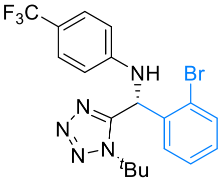
68

yield: 34.0 mg (75%); (Flash column chromatography eluent, petroleum ether/ethyl acetate/dichloromethane = 6/1/1); white foam; $[\alpha]_D^{25}$ = -49.1 (c 0.34, MeOH); **^{1}H-NMR** (600 MHz, $CDCl_3$) δ 7.64 (d, *J* = 7.8 Hz, 1H), 7.41 (d, *J* = 8.4 Hz, 2H), 7.38 – 7.31 (m, 2H), 7.26-7.22 (m, 1H), 6.67 (d, *J* = 8.4 Hz, 2H), 6.48 (d, *J* = 9.2 Hz, 1H), 5.18 (d, *J* = 9.2 Hz, 1H), 1.74 (s, 9H); **^{13}C-NMR** (151 MHz, $CDCl_3$) δ 153.6, 148.0, 136.4, 133.4, 130.4, 129.2, 128.3, 126.9 (q, *J* = 3.6 Hz), 124.5 (q, *J* = 270.9 Hz), 123.7, 121.1 (q, *J* = 32.6 Hz), 113.2, 62.5, 53.2, 29.9; **^{19}F-NMR** (564 MHz, $CDCl_3$) δ -61.5; **HRMS** (ESI) calculated for $C_{19}H_{20}{}^{79}BrF_3N_5$ $[M+H]^+$: 454.0854, found: 454.0859; **HRMS** (ESI) calculated for $C_{19}H_{20}{}^{81}BrF_3N_5$ $[M+H]^+$: 456.0834, found: 456.0840; **Enantiomeric ratio**: 93:7, determined by HPLC (Daicel Chirapak IE, isopropanol / hexanel = 10/90, flow rate 1.0 mL/min, T = 30 ºC, λ = 254 nm): t_R = 8.24 min (major), t_R = 8.75 min (minor).

(*R*)-*N*-((1-(*tert*-butyl)-1*H*-tetrazol-5-yl)(2-chlorophenyl)methyl)-4-(trifluoromethyl)aniline (69): yield: 25.3 mg (62%); (Flash column chromatography eluent, petroleum ether/ethyl

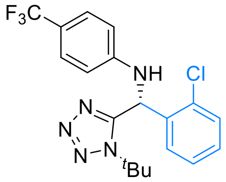

69

acetate/dichloromethane = 6/1/1); colorless oil; $[\alpha]_D^{25}$ = -76.3 (c 0.25, MeOH); **^{1}H-NMR** (600 MHz, $CDCl_3$) δ 7.47 – 7.43 (m, 1H), 7.43 – 7.36 (m, 3H), 7.35 – 7.28 (m, 2H), 6.67 (d, *J* = 8.4 Hz, 2H), 6.53 (d, *J* = 9.1 Hz, 1H), 5.13 (d, *J* = 9.1 Hz, 1H), 1.75 (s, 9H); **^{13}C-NMR** (151 MHz, $CDCl_3$) δ 153.7, 147.9, 134.9, 133.1, 130.2, 130.0, 128.9, 127.7, 126.9 (q, *J* = 3.8 Hz), 122.7 (q, *J* = 272.2 Hz), 121.1 (q, *J* = 32.8 Hz), 113.1, 62.4, 50.6, 29.9; **^{19}F-NMR** (564 MHz, $CDCl_3$) δ -61.5; **HRMS** (ESI) calculated for $C_{19}H_{20}{}^{35}ClF_3N_5$ $[M+H]^+$: 410.1359, found: 410.1366; **HRMS** (ESI) calculated for $C_{19}H_{20}{}^{37}ClF_3N_5$ $[M+H]^+$: 412.1324, found: 412.1284; **Enantiomeric ratio**: 94:6, determined by HPLC (Daicel Chirapak IA, isopropanol / hexanel = 30/70, flow rate 1.0 mL/min, T = 30 °C, λ = 254 nm): t_R = 7.00 min (major), t_R = 5.36 min (minor).

(*R*)-*N*-((1-(*tert*-butyl)-1*H*-tetrazol-5-yl)(2-(trifluoromethyl)phenyl)methyl)-4-(trifluoromethyl)-aniline (70): yield: 34.5 mg (78%); (Flash column chromatography eluent, petroleum ether/ethyl acetate/dichloromethane = 6/1/1); slight yellow oil; $[\alpha]_D^{25}$ = -38.5 (c 0.34, MeOH); **^{1}H-NMR** (600 MHz, $CDCl_3$) δ 7.75 (d, *J* = 7.9 Hz, 2H), 7.61 (t, *J* = 7.6 Hz, 1H), 7.50 (t, *J* = 7.7 Hz, 1H), 7.39 (d, *J* = 8.5 Hz, 2H), 6.64 (d, *J* = 8.5 Hz, 2H), 6.58 (d, *J* = 8.9 Hz, 1H), 5.22 (d, *J* = 8.8 Hz, 1H), 1.77 (s, 9H); **^{13}C-NMR** (151 MHz, $CDCl_3$) δ 153.7, 147.3, 135.6, 132.8, 129.0, 128.8, 127.8 (q, *J* = 30.7 Hz), 127.0 (q, *J* = 3.6 Hz), 126.6 (q, *J* = 5.8 Hz), 124.4 (q, *J* = 266.1 Hz), 123.5 (q, *J* = 272.4 Hz), 121.2 (q, *J* = 31.0 Hz), 112.9, 63.0, 49.3, 29.8; **^{19}F-NMR** (564 MHz, $CDCl_3$) δ -61.5, -59.3; **HRMS** (ESI) calculated for $C_{20}H_{19}F_6N_5Na$ $[M+Na]^+$: 466.1442, found: 466.1446; **Enantiomeric ratio**: 95:5, determined by HPLC (Daicel Chirapak IF, isopropanol / hexanel = 30/70, flow rate 1.0 mL/min, T = 30 °C, λ = 254 nm): t_R = 4.34 min (major), t_R = 3.89 min (minor).

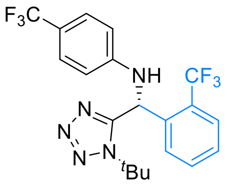

70

(*R*)-*N*-((1-(*tert*-butyl)-1*H*-tetrazol-5-yl)(2-fluorophenyl)methyl)-4-(trifluoromethyl)aniline (71): yield: 30.2 mg (77%); (Flash column chromatography eluent, petroleum ether/ethyl acetate/dichloromethane = 5/1/1); white foam; $[\alpha]_D^{25}$ = -54.6 (c 0.30, MeOH); **^{1}H-NMR** (600 MHz, $CDCl_3$) δ 7.53 – 7.47 (m, 1H), 7.41 (d, *J* = 8.5 Hz, 2H), 7.36 – 7.31 (m, 1H), 7.19 – 7.15 (m, 1H), 7.13 – 7.07 (m, 1H), 6.70 (d, *J* = 8.5 Hz, 2H), 6.52 – 6.47 (m, 1H), 5.20 (d, *J* = 8.9 Hz, 1H), 1.74 (s, 9H); **^{13}C-NMR** (151 MHz, $CDCl_3$) δ 160.7, 159.0, 154.0, 147.7, 130.6 (d, *J* = 8.5 Hz), 128.8 (d, *J* = 2.9 Hz), 126.9 (q, *J* = 3.6 Hz), 125.2 (d, *J* = 3.4 Hz), 123.9 (q, *J* = 273.4 Hz), 121.0 (q, *J* = 31.6 Hz), 115.7 (d, *J* = 21.8 Hz), 112.9, 62.1, 46.5, 29.9; **^{19}F-NMR** (564 MHz, $CDCl_3$) δ -118.2, -61.5; **HRMS** (ESI) calculated for $C_{19}H_{20}F_4N_5$ $[M+H]^+$: 394.1649, found: 394.1655; **Enantiomeric ratio**: 92.5:7.5, determined by HPLC (Daicel Chirapak IB, isopropanol / hexanel = 20/80, flow rate 1.0 mL/min, T = 30 °C, λ = 254 nm): t_R = 5.60 min (major), t_R = 5.23 min (minor).

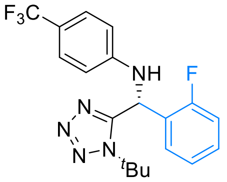

71

(*R*)-*N*-((1-(*tert*-butyl)-1*H*-tetrazol-5-yl)(3-chlorophenyl)methyl)-4-(trifluoromethyl)aniline (72):

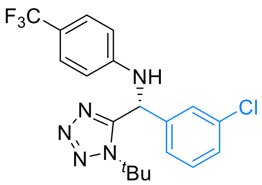

72

yield: 33.1 mg (81%); (Flash column chromatography eluent, petroleum ether/ethyl acetate/dichloromethane = 6/1/1); colorless oil; $[\alpha]_D^{25}$ = -29.7 (c 0.33, MeOH); **^{1}H-NMR** (600 MHz, $CDCl_3$) δ 7.45 – 7.36 (m, 3H), 7.35 – 7.28 (m, 2H), 7.26 – 7.22 (m, 1H), 6.66 (d, *J* = 8.5 Hz, 2H), 6.13 (d, *J* = 8.7 Hz, 1H), 5.28 (d, *J* = 8.6 Hz, 1H), 1.74 (s, 9H); **^{13}C-NMR** (151 MHz, $CDCl_3$) δ 154.2, 147.9, 139.5, 135.3, 130.5, 129.2, 127.8, 126.9 (q, *J* = 3.7 Hz), 126.3 (q, *J* = 270.2 Hz), 125.7, 121.1 (q, *J* = 33.0 Hz), 113.1, 62.0, 53.3, 30.2; **^{19}F-NMR** (564 MHz, $CDCl_3$) δ -61.5; **HRMS** (ESI) calculated for $C_{19}H_{20}{}^{35}ClF_3N_5$ $[M+H]^+$: 410.1354, found: 410.1351; **HRMS** (ESI) calculated for $C_{19}H_{20}{}^{37}ClF_3N_5$ $[M+H]^+$: 412.1324, found: 412.1320; **Enantiomeric ratio**: 94:6, determined by HPLC (Daicel Chirapak IF, isopropanol / hexanel = 30/70, flow rate 1.0 mL/min, T = 30 °C, λ = 254 nm): t_R = 4.52 min (major), t_R = 5.28 min (minor).

(*R*)-*N*-((1-(*tert*-butyl)-1*H*-tetrazol-5-yl)(3-(trifluoromethyl)phenyl)methyl)-4-(trifluoromethyl)-aniline (73):

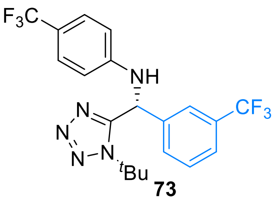

73

yield: 32.3 mg (73%); (Flash column chromatography eluent, petroleum ether/ethyl acetate/dichloromethane = 6/1/1); slight yellow oil; $[\alpha]_D^{25}$ = -54.6 (c 0.32, MeOH); **^{1}H-NMR** (600 MHz, $CDCl_3$) δ 7.66 – 7.57 (m, 3H), 7.54 – 7.50 (m, 1H), 7.44 – 7.39 (m, 2H), 6.66 (d, *J* = 8.5 Hz, 2H), 6.22 (d, *J* = 8.7 Hz, 1H), 5.32 (d, *J* = 8.6 Hz, 1H), 1.75 (s, 9H); **^{13}C-NMR** (151 MHz, $CDCl_3$) δ 154.1, 147.8, 138.6, 131.7 (q, *J* = 32.5 Hz), 130.8, 129.8, 126.9 (q, *J* = 3.7 Hz), 125.8 (q, *J* = 3.5 Hz), 124.4 (q, *J* = 3.8 Hz), 123.5 (q, *J* = 271.6 Hz), 121.2 (q, *J* = 32.1 Hz), 113.1, 62.1, 53.4, 30.2; **^{19}F-NMR** (564 MHz, $CDCl_3$) δ -62.7, -61.5; **HRMS** (ESI) calculated for $C_{20}H_{20}F_6N_5$ $[M+H]^+$: 444.1617, found: 444.1614; **Enantiomeric ratio**: 91:9, determined by HPLC (Daicel Chirapak IF, isopropanol / hexanel = 30/70, flow rate 1.0 mL/min, T = 30 °C, λ = 254 nm): t_R = 3.90 min (major), t_R = 4.20 min (minor).

(*R*)-*N*-((4-bromo-2-fluorophenyl)(1-(*tert*-butyl)-1*H*-tetrazol-5-yl)methyl)-4-(trifluoromethyl)-aniline (74):

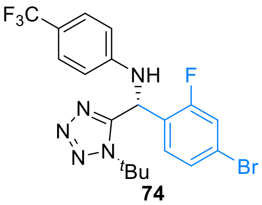

74

yield: 36.2 mg (77%); (Flash column chromatography eluent, petroleum ether/ethyl acetate/dichloromethane = 6/1/1); slight yellow oil; $[\alpha]_D^{25}$ = -32.1 (c 0.36, MeOH); **^{1}H-NMR** (600 MHz, $CDCl_3$) δ 7.49 – 7.44 (m, 1H), 7.44 – 7.40 (m, 2H), 7.34 – 7.27 (m, 2H), 6.66 (d, *J* = 8.4 Hz, 2H), 6.40 (d, *J* = 8.7 Hz, 1H), 5.28 (d, *J* = 8.6 Hz, 1H), 1.76 (s, 9H); **^{13}C-NMR** (151 MHz, $CDCl_3$) δ 160.5, 158.8, 153.5, 147.4, 130.0 (d, *J* = 3.8 Hz), 128.6 (d, *J* = 3.4 Hz), 127.0 (q, *J* = 3.7 Hz), 124.5 (q, *J* = 270.9 Hz), 124.0 (d, *J* = 13.8 Hz), 123.3 (d, *J* = 9.7 Hz), 121.3 (q, *J* = 32.9 Hz), 119.3 (d, *J* = 24.9 Hz), 112.8, 62.2, 46.3 (d, *J* = 3.1 Hz), 29.9; **^{19}F-NMR** (564 MHz, $CDCl_3$) δ -115.6, -61.5; **HRMS** (ESI) calculated for $C_{19}H_{19}{}^{79}BrF_4N_5$ $[M+H]^+$: 472.0754, found: 472.0753; **HRMS** (ESI) calculated

for $C_{19}H_{19}{}^{81}BrF_4N_5$ [M+H]$^+$: 474.0734, found: 474.0742; **Enantiomeric ratio**: 95:5, determined by HPLC (Daicel Chirapak IF, isopropanol / hexanel = 30/70, flow rate 1.0 mL/min, T = 30 ºC, λ = 254 nm): t_R = 4.87 min (major), t_R = 4.35 min (minor).

(*S*)-*N*-((1-(*tert*-butyl)-1*H*-tetrazol-5-yl)(furan-2-yl)methyl)-4-(trifluoromethyl)aniline (75): yield: 30.4 mg (83%); (Flash column chromatography eluent, petroleum ether/ethyl acetate/dichloromethane = 5/1/1); colorless oil; $[\alpha]_D^{25}$ = -26.2 (c 0.30, MeOH); **^{1}H-NMR** (600 MHz, $CDCl_3$) δ 7.44 (d, *J* = 8.4 Hz, 2H), 7.40 – 7.36 (m, 1H), 6.76 (d, *J* = 8.4 Hz, 2H), 6.38 – 6.31 (m, 1H), 6.30 – 6.22 (m, 2H), 5.36 (d, *J* = 9.0 Hz, 1H), 1.74 (s, 9H); **^{13}C-NMR** (151 MHz, $CDCl_3$) δ 153.0, 150.4, 148.0, 143.0, 126.8 (q, *J* = 3.7 Hz), 124.5 (q, *J* = 270.2 Hz), 121.1 (q, *J* = 32.2 Hz), 113.2, 111.0, 109.1, 62.1, 48.2, 29.9; **^{19}F-NMR** (564 MHz, $CDCl_3$) δ -61.5; **HRMS** (ESI) calculated for $C_{17}H_{19}F_3N_5O$ [M+H]$^+$: 366.1536, found: 366.1539; **Enantiomeric ratio**: 92.5:7.5, determined by HPLC (Daicel Chirapak IF, isopropanol / hexanel = 30/70, flow rate 1.0 mL/min, T = 30 ºC, λ = 254 nm): t_R = 5.42 min (major), t_R = 6.75 min (minor).

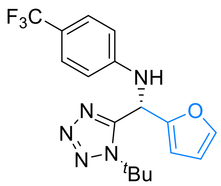

75

(*R*)-*N*-((1-(*tert*-butyl)-1*H*-tetrazol-5-yl)(naphthalen-2-yl)methyl)-4-(trifluoromethyl)aniline (76): yield: 26.3 mg (62%); (Flash column chromatography eluent, petroleum ether/ethyl acetate/dichloromethane = 6/1/1); slight yellow oil; $[\alpha]_D^{25}$ = -49.6 (c 0.26, MeOH); **^{1}H-NMR** (600 MHz, $CDCl_3$) δ 7.87 (d, *J* = 8.5 Hz, 1H), 7.84 – 7.81 (m, 1H), 7.79 – 7.76 (m, 1H), 7.73 – 7.70 (m, 1H), 7.54 – 7.48 (m, 3H), 7.40 – 7.36 (m, 2H), 6.71 – 6.68 (m, 2H), 6.32 (d, *J* = 8.7 Hz, 1H), 5.34 (d, *J* = 8.7 Hz, 1H), 1.73 (s, 9H); **^{13}C-NMR** (151 MHz, $CDCl_3$) δ 154.6, 148.3, 134.7, 133.2, 133.1, 129.5, 128.1, 127.7, 126.9, 126.8 (q, *J* = 3.7 Hz), 125.0 (q, *J* = 271.1 Hz), 124.97, 120.8 (q, *J* = 32.6 Hz), 113.1, 62.0, 54.1, 30.1; **^{19}F-NMR** (564 MHz, $CDCl_3$) δ -61.5; **HRMS** (ESI) calculated for $C_{23}H_{23}F_3N_5$ [M+H]$^+$: 426.1900, found: 426.1907; **Enantiomeric ratio**: 88.5:11.5, determined by HPLC (Daicel Chirapak IF, isopropanol / hexanel = 30/70, flow rate 1.0 mL/min, T = 30 ºC, λ = 254 nm): t_R = 5.77 min (major), t_R = 9.40 min (minor).

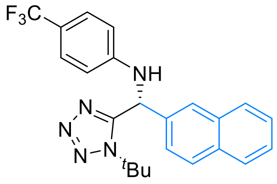

76

(*R*)-*N*-((1-(*tert*-butyl)-1*H*-tetrazol-5-yl)(pyridin-2-yl)methyl)-4-(trifluoromethyl)aniline (77): yield: 25.1 mg (67%); (Flash column chromatography eluent, petroleum ether/ethyl acetate/dichloromethane = 3/1/1); slight yellow oil; $[\alpha]_D^{25}$ = -65.5 (c 0.25, MeOH); **^{1}H-NMR** (600 MHz, $CDCl_3$) δ 8.57 – 8.52 (m, 1H), 7.72 – 7.65 (m, 1H), 7.52 (d, *J* = 7.9 Hz, 1H), 7.40 (d, *J* = 8.5 Hz, 2H), 7.26 – 7.22 (m, 1H), 6.77 (d, *J* = 8.5 Hz, 2H), 6.38 (d, *J* = 6.8 Hz, 1H), 5.89 (d, *J* = 6.6 Hz, 1H), 1.73 (s, 9H); **^{13}C-NMR** (151 MHz, $CDCl_3$) δ 157.0, 154.4, 148.8, 148.2, 137.6, 126.8 (q, *J* = 3.7 Hz), 124.6 (q, *J* = 270.6 Hz), 123.4, 121.8, 120.5 (q, *J* = 32.6 Hz), 113.0, 62.4, 55.3, 30.0; **^{19}F-NMR** (564 MHz, $CDCl_3$) δ -61.4;

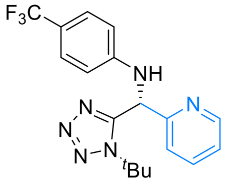

77

HRMS (ESI) calculated for $C_{18}H_{20}F_3N_6$ $[M+H]^+$: 377.1702, found: 377.1710; **Enantiomeric ratio**: 72.5:27.5, determined by HPLC (Daicel Chirapak IF, isopropanol / hexanel = 30/70, flow rate 1.0 mL/min, T = 30 ºC, λ = 254 nm): t_R = 6.48 min (major), t_R = 5.42 min (minor).

(*R*)-4-((1-(tert-butyl)-1*H*-tetrazol-5-yl)((4-(trifluoromethyl)phenyl)amino)methyl)phenol (78):

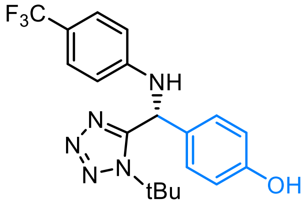

78

yield: 29.7 mg (76%); (Flash column chromatography eluent, petroleum ether/ethyl acetate/dichloromethane = 3/1/1); white foam; $[\alpha]_D^{25}$ = -72.6 (c 0.30, MeOH); **^{1}H-NMR** (600 MHz, $CDCl_3$) δ 7.38 (d, *J* = 8.5 Hz, 2H), 7.17 – 7.12 (m, 2H), 6.80 – 6.76 (m, 2H), 6.64 (d, *J* = 8.5 Hz, 2H), 6.10 (d, *J* = 8.6 Hz, 1H), 6.01 – 5.96 (m, 1H), 5.17 (d, *J* = 8.5 Hz, 1H), 1.70 (s, 9H); **^{13}C-NMR** (151 MHz, $CDCl_3$) δ 156.3, 155.1, 148.2, 129.1, 126.7 (q, *J* = 3.8 Hz), 124.6 (q, *J* = 270.8 Hz), 120.7 (q, *J* = 32.3 Hz), 116.2, 113.0, 62.0, 53.4, 30.0; **^{19}F-NMR** (564 MHz, $CDCl_3$) δ -61.4; **HRMS** (ESI) calculated for $C_{19}H_{20}F_3N_5NaO$ $[M+Na]^+$: 414.1518, found: 414.1519; **Enantiomeric ratio**: 78.5:21.5, determined by HPLC (Daicel Chirapak IF, isopropanol / hexanel = 30/70, flow rate 1.0 mL/min, T = 30 ºC, λ = 254 nm): t_R = 4.07 min (major), t_R = 7.09 min (minor).

(*R*)-*N*-((1-(*tert*-butyl)-1*H*-tetrazol-5-yl)(cyclohexyl)methyl)-4-(trifluoromethyl)aniline (79):

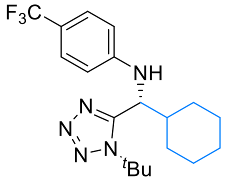

79

yield: 22.1 mg (58%); (Flash column chromatography eluent, petroleum ether/ethyl acetate/dichloromethane = 6/1/1); colorless oil; $[\alpha]_D^{25}$ = -48.9 (c 0.22, MeOH); **^{1}H-NMR** (600 MHz, $CDCl_3$) δ 7.40 (d, *J* = 8.4 Hz, 2H), 6.67 (d, *J* = 8.4 Hz, 2H), 4.94 – 4.88 (m, 1H), 4.56 (d, *J* = 10.0 Hz, 1H), 2.16 – 2.09 (m, 1H), 2.04 – 2.00 (m, 1H), 1.81 – 1.77 (m, 1H), 1.73 (s, 9H), 1.44 – 1.39 (m, 1H), 1.34 – 1.07 (m, 7H); **^{13}C-NMR** (151 MHz, $CDCl_3$) δ 155.3, 149.4, 126.8 (q, *J* = 3.7 Hz), 124.6 (q, *J* = 270.5 Hz), 119.8 (q, *J* = 33.1 Hz), 112.5, 61.5, 54.4, 44.8, 30.7, 30.5, 29.2, 26.1, 26.0, 25.9; **^{19}F-NMR** (564 MHz, $CDCl_3$) δ -61.3; **HRMS** (ESI) calculated for $C_{19}H_{27}F_3N_5$ $[M+H]^+$: 382.2219, found: 382.2223; **Enantiomeric ratio**: 85.5:14.5, determined by HPLC (Daicel Chirapak IF, isopropanol / hexanel = 10/90, flow rate 1.0 mL/min, T = 30 ºC, λ = 254 nm): t_R = 7.63 min (major), t_R = 8.26 min (minor).

(*R*)-*N*-(1-(1-(*tert*-butyl)-1*H*-tetrazol-5-yl)butyl)-4-(trifluoromethyl)aniline (80): yield: 21.2 mg

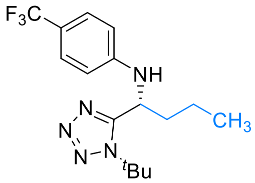

80

(62%); (Flash column chromatography eluent, petroleum ether/ethyl acetate/dichloromethane = 6/1/1); colorless oil; $[\alpha]_D^{25}$ = -42.8 (c 0.21, MeOH); **^{1}H-NMR** (600 MHz, $CDCl_3$) δ 7.43 (d, *J* = 8.4 Hz, 2H), 6.68 (d, *J* = 8.4 Hz, 2H), 5.11 – 5.03 (m, 1H), 4.64 (d, *J* = 9.9 Hz, 1H), 2.17 – 2.08 (m, 1H), 2.07 – 1.98 (m, 1H), 1.76 (s, 9H), 1.51 – 1.43 (m, 1H), 1.40 – 1.32 (m, 1H), 0.96 (t, *J* = 7.3 Hz, 3H); **^{13}C-NMR** (151 MHz, $CDCl_3$) δ 155.5, 148.7, 126.9 (q, *J* = 3.8 Hz), 124.6 (q, *J* = 270.7 Hz), 120.4 (q, *J* = 32.9 Hz), 112.7, 61.5, 49.4, 37.2, 30.2, 19.3, 13.7; **^{19}F-NMR** (564 MHz, $CDCl_3$) δ -61.4; **HRMS**

(ESI) calculated for $C_{16}H_{23}F_3N_5$ $[M+H]^+$: 342.1906, found: 342.1902; **Enantiomeric ratio**: 87.5:12.5, determined by HPLC (Daicel Chirapak IF, isopropanol / hexanel = 30/70, flow rate 1.0 mL/min, T = 30 ºC, λ = 254 nm): t_R = 5.03 min (major), t_R = 4.52 min (minor).

(*R*)-4-((1-(*tert*-butyl)-1*H*-tetrazol-5-yl)(p-tolylamino)methyl)benzonitrile (81): yield: 24.9 mg

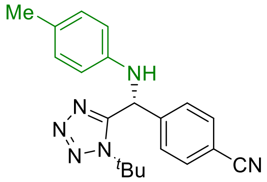

81

(72%); (Flash column chromatography eluent, petroleum ether/ethyl acetate/dichloromethane = 6/1/1); white foam; $[\alpha]_D^{25}$ = -65.5 (c 0.25, MeOH); **^{1}H-NMR** (600 MHz, $CDCl_3$) δ 7.65 (d, *J* = 8.0 Hz, 2H), 7.51 (d, *J* = 8.0 Hz, 2H), 6.98 (d, *J* = 7.8 Hz, 2H), 6.57 (d, *J* = 8.0 Hz, 2H), 6.14 (s, 1H), 4.63 (s, 1H), 2.22 (s, 3H), 1.75 (s, 9H); **^{13}C-NMR** (151 MHz, $CDCl_3$) δ 154.3, 143.5, 142.6, 132.7, 130.1, 129.4, 128.4, 118.2, 114.6, 112.6, 61.9, 54.3, 30.2, 20.4; **HRMS** (ESI) calculated for $C_{20}H_{23}N_6$ $[M+H]^+$: 347.1984, found: 347.1987; **Enantiomeric ratio**: 95.5:4.5, determined by HPLC (Daicel Chirapak IB, isopropanol / hexanel = 20/80, flow rate 1.0 mL/min, T = 30 ºC, λ = 254 nm): t_R = 5.20 min (major), t_R = 6.31 min (minor).

(*R*)-4-((1-(*tert*-butyl)-1*H*-tetrazol-5-yl)((4-fluorophenyl)amino)methyl)benzonitrile (82): yield:

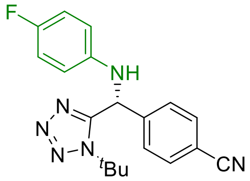

82

24.9 mg (71%); (Flash column chromatography eluent, petroleum ether/ethyl acetate/dichloromethane = 4/1/1); white foam; $[\alpha]_D^{25}$ = -78.1 (c 0.25, MeOH); **^{1}H-NMR** (600 MHz, $CDCl_3$) δ 7.66 (d, *J* = 8.0 Hz, 2H), 7.50 (d, *J* = 8.0 Hz, 2H), 6.92 – 6.83 (m, 2H), 6.65 – 6.57 (m, 2H), 6.08 (d, *J* = 9.4 Hz, 1H), 4.72 (d, *J* = 9.4 Hz, 1H), 1.73 (s, 9H); **^{13}C-NMR** (151 MHz, $CDCl_3$) δ 157.9, 156.3, 154.1, 143.1, 141.3, 132.8, 128.4, 118.0, 116.2 (d, *J* = 22.7 Hz), 116.0 (d, *J* = 7.7 Hz), 112.8, 62.0, 55.1, 30.2; **^{19}F-NMR** (564 MHz, $CDCl_3$) δ -124.2; **HRMS** (ESI) calculated for $C_{19}H_{20}FN_6$ $[M+H]^+$: 351.1728, found: 351.1733; **Enantiomeric ratio**: 98:2, determined by HPLC (Daicel Chirapak IA, isopropanol / hexanel = 30/70, flow rate 1.0 mL/min, T = 30 ºC, λ = 254 nm): t_R = 12.70 min (major), t_R = 9.29 min (minor).

(*R*)-4-((1-(*tert*-butyl)-1*H*-tetrazol-5-yl)((4-chlorophenyl)amino)methyl)benzonitrile (83): yield:

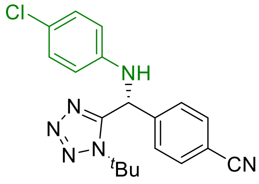

83

22.7 mg (62%); (Flash column chromatography eluent, petroleum ether/ethyl acetate/dichloromethane = 4/1/1); white foam; $[\alpha]_D^{25}$ = -77.7 (c 0.23, MeOH); **^{1}H-NMR** (600 MHz, $CDCl_3$) δ 7.66 (d, *J* = 7.8 Hz, 2H), 7.51 (d, *J* = 7.8 Hz, 2H), 7.12 (d, *J* = 8.2 Hz, 2H), 6.57 (d, *J* = 8.2 Hz, 2H), 6.11 (d, *J* = 9.1 Hz, 1H), 4.94 (d, *J* = 9.0 Hz, 1H), 1.75 (s, 9H); **^{13}C-NMR** (151 MHz, $CDCl_3$) δ 154.0, 143.6, 142.9, 132.8, 129.5, 128.3, 124.7, 118.0, 115.3, 112.8, 62.1, 54.0, 30.2; **HRMS** (ESI) calculated for $C_{19}H_{20}{}^{35}ClN_6$ $[M+H]^+$: 367.1438, found: 367.1435; **HRMS** (ESI) calculated for $C_{19}H_{20}{}^{37}ClN_6$ $[M+H]^+$: 369.1408, found: 369.1451; **Enantiomeric ratio**: 96:4, determined by HPLC (Daicel Chirapak IA, isopropanol

/ hexanel = 30/70, flow rate 1.0 mL/min, T = 30 ºC, λ = 254 nm): t_R = 14.23 min (major), t_R = 9.47 min (minor).

(*R*)-4-(((4-bromophenyl)amino)(1-(*tert*-butyl)-1*H*-tetrazol-5-yl)methyl)benzonitrile (84): yield: 29.6 mg (72%); (Flash column chromatography eluent, petroleum ether/ethyl acetate/dichloromethane = 6/1/1); white foam; $[\alpha]_D^{25}$ = -47.4 (c 0.29, MeOH); **^{1}H-NMR** (600 MHz, $CDCl_3$) δ 7.66 (d, *J* = 8.3 Hz, 2H), 7.51 (d, *J* = 8.2 Hz, 2H), 7.26 (d, *J* = 9.7 Hz, 2H), 6.53 (d, *J* = 8.7 Hz, 2H), 6.11 (d, *J* = 9.1 Hz, 1H), 4.98 (d, *J* = 9.1 Hz, 1H), 1.75 (s, 9H); **^{13}C-NMR** (151 MHz, $CDCl_3$) δ 153.9, 144.0, 142.9, 132.8, 132.4, 128.3, 118.0, 115.7, 112.8, 111.7, 62.1, 53.9, 30.2; **HRMS** (ESI) calculated for $C_{19}H_{20}{}^{79}BrN_6$ $[M+H]^+$: 411.0927, found: 411.0934; **HRMS** (ESI) calculated for $C_{19}H_{20}{}^{81}BrN_6$ $[M+H]^+$: 413.0907, found: 413.0912; **Enantiomeric ratio**: 97:3, determined by HPLC (Daicel Chirapak IA, isopropanol / hexanel = 30/70, flow rate 1.0 mL/min, T = 30 ºC, λ = 254 nm): t_R = 9.42 min (major), t_R = 14.50 min (minor).

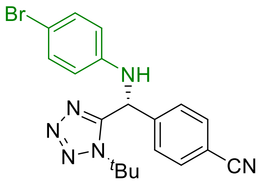

84

Methyl (*R*)-4-(((1-(*tert*-butyl)-1*H*-tetrazol-5-yl)(4-cyanophenyl)methyl)amino)benzoate (85): yield: 29.3 mg (75%); (Flash column chromatography eluent, petroleum ether/ethyl acetate/dichloromethane = 3/1/1); colorless oil; $[\alpha]_D^{25}$ = -133.1 (c 0.29, MeOH); **^{1}H-NMR** (600 MHz, $CDCl_3$) δ 7.86 (d, *J* = 8.7 Hz, 2H), 7.67 (d, *J* = 8.3 Hz, 2H), 7.53 (d, *J* = 8.2 Hz, 2H), 6.62 (d, *J* = 8.7 Hz, 2H), 6.24 (d, *J* = 8.5 Hz, 1H), 5.43 (d, *J* = 8.5 Hz, 1H), 3.84 (s, 3H), 1.77 (s, 9H); **^{13}C-NMR** (151 MHz, $CDCl_3$) δ 166.7, 153.7, 148.7, 142.6, 132.9, 131.7, 128.3, 121.1, 117.9, 113.0, 112.6, 62.2, 53.0, 51.7, 30.2; **HRMS** (ESI) calculated for $C_{21}H_{23}N_6O_2$ $[M+H]^+$: 391.1877, found: 391.1882; **Enantiomeric ratio**: 95.5:4.5, determined by HPLC (Daicel Chirapak IA, isopropanol / hexanel = 20/80, flow rate 1.0 mL/min, T = 30 ºC, λ = 254 nm): t_R = 5.69 min (major), t_R = 8.43 min (minor).

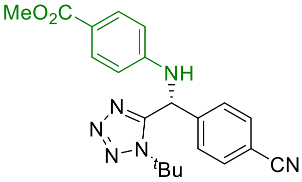

85

(*R*)-4-((1-(*tert*-butyl)-1*H*-tetrazol-5-yl)((4-cyanophenyl)amino)methyl)benzonitrile (86): yield: 23.3 mg (65%); (Flash column chromatography eluent, petroleum ether/ethyl acetate/dichloromethane = 2/1/1); white foam; $[\alpha]_D^{25}$ = -89.8 (c 0.23, MeOH); **^{1}H-NMR** (600 MHz, $CDCl_3$) δ 7.69 (d, *J* = 7.9 Hz, 2H), 7.52 (d, *J* = 7.9 Hz, 2H), 7.45 (d, *J* = 8.3 Hz, 2H), 6.64 (d, *J* = 8.3 Hz, 2H), 6.19 (d, *J* = 8.3 Hz, 1H), 5.59 (d, *J* = 8.1 Hz, 1H), 1.76 (s, 9H); **^{13}C-NMR** (151 MHz, $CDCl_3$) δ 153.4, 148.2, 142.1, 134.0, 133.0, 128.3, 119.3, 117.8, 113.3, 102.0, 62.3, 52.9, 30.2; **HRMS** (ESI) calculated for $C_{20}H_{20}N_7$ $[M+H]^+$: 358.1780, found: 358.1788; **Enantiomeric ratio**: 97:3, determined by HPLC (Daicel Chirapak IA, isopropanol / hexanel = 30/70, flow rate 1.0 mL/min, T = 30 ºC, λ = 254 nm): t_R = 5.65 min (major), t_R = 5.03 min (minor).

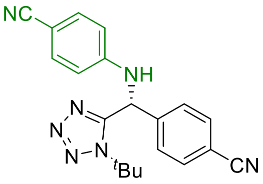

86

(*R*)-4-((1-(*tert*-butyl)-1*H*-tetrazol-5-yl)((4-fluoro-3-(trifluoromethyl)phenyl)amino)methyl)-bezonitrile (87): yield: 30.1 mg (72%); (Flash column chromatography eluent, petroleum ether/ethyl acetate/dichloromethane = 6/1/1); white foam; $[\alpha]_D^{25}$ = -78.3 (c 0.30, MeOH); **^{1}H-NMR** (600 MHz, $CDCl_3$) δ 7.68 (d, *J* = 7.8 Hz, 2H), 7.50 (d, *J* = 7.9 Hz, 2H), 7.01 (t, *J* = 9.2 Hz, 1H), 6.85 – 6.80 (m, 1H), 6.80 – 6.75 (m, 1H), 6.10 (d, *J* = 8.4 Hz, 1H), 5.11 (d, *J* = 8.8 Hz, 1H), 1.73 (s, 9H); **^{13}C-NMR** (151 MHz, $CDCl_3$) δ 153.8, 142.5, 141.5 (d, *J* = 2.3 Hz), 133.1, 128.6, 122.5 (q, *J* = 272.3 Hz), 119.2 (d, *J* = 7.4 Hz), 118.9 (q, *J* = 13.6 Hz), 118.2 (d, *J* = 22.0 Hz), 118.0, 113.3, 111.9 (q, *J* = 4.5 Hz), 62.3, 54.7, 30.3; **^{19}F-NMR** (564 MHz, $CDCl_3$) δ -126.8, -61.7; **HRMS** (ESI) calculated for $C_{20}H_{19}F_4N_6$ $[M+H]^+$: 419.1607, found: 419.1601; **Enantiomeric ratio**: 95:5, determined by HPLC (Daicel Chirapak IF, isopropanol / hexanel = 20/80, flow rate 1.0 mL/min, T = 30 ºC, λ = 254 nm): t_R = 4.16 min (major), t_R = 4.67 min (minor).

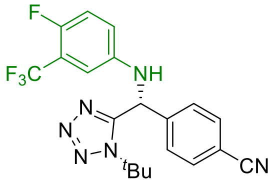

87

(*R*)-4-(((3-bromo-4-(trifluoromethyl)phenyl)amino)(1-(*tert*-butyl)-1*H*-tetrazol-5-yl)methyl)-benzonitrile (88): yield: 32.6 mg (68%); (Flash column chromatography eluent, petroleum ether/ethyl acetate/dichloromethane = 6/1/1); white foam; $[\alpha]_D^{25}$ = -47.9 (c 0.33, MeOH); **^{1}H-NMR** (600 MHz, $CDCl_3$) δ 7.70 (d, *J* = 8.2 Hz, 2H), 7.51 (d, *J* = 8.2 Hz, 2H), 7.45 (d, *J* = 8.6 Hz, 1H), 6.95 – 6.89 (m, 1H), 6.59 – 6.54 (m, 1H), 6.16 (d, *J* = 8.6 Hz, 1H), 5.32 (d, *J* = 8.5 Hz, 1H), 1.76 (s, 9H); **^{13}C-NMR** (151 MHz, $CDCl_3$) δ 153.6, 148.3, 142.1, 133.2, 129.3 (q, *J* = 4.5 Hz), 128.5, 123.3 (q, *J* = 271.8 Hz), 119.1, 117.9, 113.5, 111.4, 62.4, 53.3, 30.4; **^{19}F-NMR** (564 MHz, $CDCl_3$) δ -61.2; **HRMS** (ESI) calculated for $C_{20}H_{19}{}^{79}BrF_3N_6$ $[M+H]^+$: 479.0807, found: 479.0800; **HRMS** (ESI) calculated for $C_{20}H_{19}{}^{81}BrF_3N_6$ $[M+H]^+$: 481.0786, found: 481.0780; **Enantiomeric ratio**: 97.5:2.5, determined by HPLC (Daicel Chirapak IF, isopropanol / hexanel = 20/80, flow rate 1.0 mL/min, T = 30 ºC, λ = 254 nm): t_R = 6.72 min (major), t_R = 5.70 min (minor).

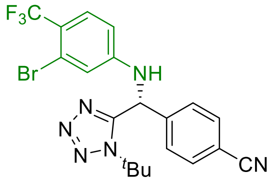

88

(*R*)-4-((1-(tert-butyl)-1*H*-tetrazol-5-yl)((3-(trifluoromethyl)phenyl)amino)methyl)benzonitrile (89): yield: 26.1 mg (65%); (Flash column chromatography eluent, petroleum ether/ethyl acetate/dichloromethane = 6/1/1); slight yellow oil; $[\alpha]_D^{25}$ = -43.2 (c 0.26, MeOH); **^{1}H-NMR** (600 MHz, $CDCl_3$) δ 7.68 (d, *J* = 8.3 Hz, 2H), 7.52 (d, *J* = 8.3 Hz, 2H), 7.29 – 7.25 (m, 1H), 7.04 (d, *J* = 7.6 Hz, 1H), 6.84 (s, 1H), 6.81 – 6.77 (m, 1H), 6.19 (d, *J* = 9.1 Hz, 1H), 5.20 (d, *J* = 9.0 Hz, 1H), 1.75 (s, 9H); **^{13}C-NMR** (151 MHz, $CDCl_3$) δ 153.8, 145.3, 142.6, 132.9, 131.9 (q, *J* = 31.9 Hz), 130.1, 128.4, 123.9 (q, *J* = 272.4 Hz), 117.9, 117.2, 116.3 (q, *J* = 3.7 Hz), 113.0, 110.1 (q, *J* = 3.6 Hz), 62.1, 53.8, 30.2; **^{19}F-NMR** (564 MHz, $CDCl_3$) δ -63.0; **HRMS** (ESI) calculated for $C_{20}H_{20}F_3N_6$ $[M+H]^+$: 401.1696, found: 401.1703;

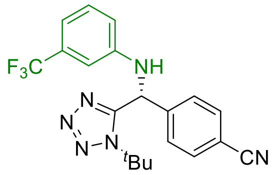

89

Enantiomeric ratio: 91:9, determined by HPLC (Daicel Chirapak IF, isopropanol / hexanel = 30/70, flow rate 1.0 mL/min, T = 30 ºC, λ = 254 nm): t_R = 5.26 min (major), t_R = 8.48 min (minor).

(*R*)-4-(((3,5-bis(trifluoromethyl)phenyl)amino)(1-(*tert*-butyl)-1*H*-tetrazol-5-yl)methyl)benzo-

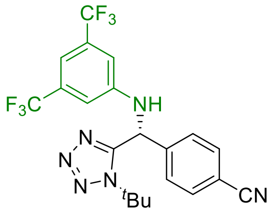

90

nitrile (90): yield: 34.2 mg (73%); (Flash column chromatography eluent, petroleum ether/ethyl acetate/dichloromethane = 6/1/1); slight yellow oil; $[\alpha]_D^{25}$ = -75.5 (c 0.34, MeOH); **^{1}H-NMR** (600 MHz, $CDCl_3$) δ 7.70 (d, *J* = 8.3 Hz, 2H), 7.53 (d, *J* = 8.2 Hz, 2H), 7.26 – 7.25 (m, 1H), 6.99 (s, 2H), 6.19 (d, *J* = 8.7 Hz, 1H), 5.65 (d, *J* = 8.7 Hz, 1H), 1.75 (s, 9H); **^{13}C-NMR** (151 MHz, $CDCl_3$) δ 153.4, 146.0, 141.7, 133.1, 132.9 (q, *J* = 33.3 Hz), 128.5, 123.1 (q, *J* = 272.7 Hz), 117.7, 113.4, 113.0, 112.7 (q, *J* = 3.7 Hz), 62.3, 53.7, 30.2; **^{19}F-NMR** (564 MHz, $CDCl_3$) δ -63.3; **HRMS** (ESI) calculated for $C_{21}H_{19}F_6N_6$ $[M+H]^+$: 469.1575, found: 469.1570; **Enantiomeric ratio**: 94:6, determined by HPLC (Daicel Chirapak IF, isopropanol / hexanel = 30/70, flow rate 1.0 mL/min, T = 30 ºC, λ = 254 nm): t_R = 5.61 min (major), t_R = 4.98 min (minor).

(*R*)-4-((1-cyclohexyl-1*H*-tetrazol-5-yl)((4-(trifluoromethyl)phenyl)amino)methyl)benzonitrile

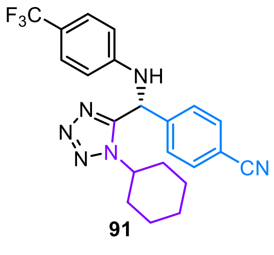

91

(91): yield: 27.3 mg (64%); (Flash column chromatography eluent, petroleum ether/ethyl acetate/dichloromethane = 6/1/1); white foam; $[\alpha]_D^{25}$ = -49.3 (c 0.27, MeOH); **^{1}H-NMR** (600 MHz, $CDCl_3$) δ 7.68 (d, *J* = 8.0 Hz, 2H), 7.57 (d, *J* = 8.0 Hz, 2H), 7.40 (d, *J* = 8.3 Hz, 2H), 6.69 (d, *J* = 8.3 Hz, 2H), 5.94 (d, *J* = 6.8 Hz, 1H), 5.63 (d, *J* = 6.7 Hz, 1H), 4.27 – 4.18 (m, 1H), 2.07 – 2.01 (m, 1H), 1.98 – 1.92 (m, 2H), 1.90 – 1.84 (m, 2H), 1.78 – 1.73 (m, 1H), 1.32 – 1.22 (m, 4H); **^{13}C-NMR** (151 MHz, $CDCl_3$) δ 153.2, 147.6, 142.4, 133.2, 128.2, 127.0 (q, *J* = 3.8 Hz), 124.5 (q, *J* = 270.9 Hz), 121.4 (q, *J* = 32.9 Hz), 117.9, 113.2, 58.9, 52.7, 33.0, 25.4, 24.7; **^{19}F-NMR** (564 MHz, $CDCl_3$) δ -61.5; **HRMS** (ESI) calculated for $C_{22}H_{22}F_3N_6$ $[M+H]^+$: 427.1853, found: 427.1857; **Enantiomeric ratio**: 87.5:12.5, determined by HPLC (Daicel Chirapak IB, isopropanol / hexanel = 30/70, flow rate 1.0 mL/min, T = 30 ºC, λ = 254 nm): t_R = 8.65 min (major), t_R = 7.85 min (minor).

(*R*)-*N*-((4-bromophenyl)(1-isopropyl-1H-tetrazol-5-yl)methyl)-4-(trifluoromethyl)aniline (92):

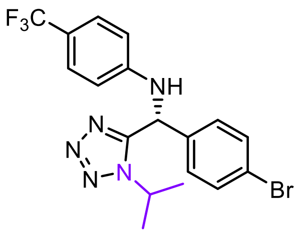

92

yield: 37.9 mg (86%); (Flash column chromatography eluent, petroleum ether/ethyl acetate/dichloromethane = 6/1/1); white foam; $[\alpha]_D^{25}$ = -81.2 (c 0.38, MeOH); **^{1}H-NMR** (600 MHz, $CDCl_3$) δ 7.52 (d, *J* = 8.4 Hz, 2H), 7.40 (d, *J* = 8.5 Hz, 2H), 7.29 (d, *J* = 8.4 Hz, 2H), 6.68 (d, *J* = 8.5 Hz, 2H), 5.85 (d, *J* = 6.6 Hz, 1H), 5.51 (d, *J* = 6.5 Hz, 1H), 4.68 – 4.62 (m, 1H), 1.60 (d, *J* = 6.7 Hz, 3H), 1.32 (d, *J* = 6.6 Hz, 3H); **^{13}C-NMR** (151 MHz, $CDCl_3$) δ 153.4, 147.8, 136.0, 132.6, 128.9, 126.8 (q, *J* = 3.7 Hz), 124.5 (q, *J* = 270.8 Hz), 123.3, 121.0 (q, *J* =

32.8 Hz), 113.1, 52.6, 51.4, 22.6, 22.3; **^{19}F-NMR** (564 MHz, $CDCl_3$) δ -61.5; **HRMS** (ESI) calculated for $C_{18}H_{17}{}^{79}BrF_3N_5Na$ $[M+Na]^+$: 462.0517, found: 462.0520; **HRMS** (ESI) calculated for $C_{18}H_{17}{}^{81}BrF_3N_5Na$ $[M+Na]^+$: 464.0497, found: 464.0500; **Enantiomeric ratio**: 86.5:13.5, determined by HPLC (Daicel Chirapak IF, isopropanol / hexanel = 30/70, flow rate 1.0 mL/min, T = 30 °C, λ = 254 nm): t_R = 4.67 min (major), t_R = 4.83 min (minor).

(*R*)-*N*-((4-bromophenyl)(1-cyclopentyl-1*H*-tetrazol-5-yl)methyl)-4-(trifluoromethyl)aniline

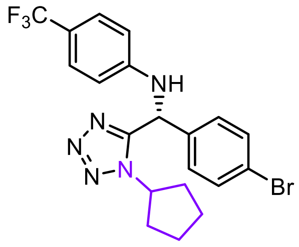

93

(93): yield: 33.6 mg (72%); (Flash column chromatography eluent, petroleum ether/ethyl acetate/dichloromethane = 5/1/1); white foam; $[\alpha]_D^{25}$ = -69.7 (c 0.34, MeOH); **^{1}H-NMR** (600 MHz, $CDCl_3$) δ 7.51 (d, *J* = 8.4 Hz, 2H), 7.39 (d, *J* = 8.5 Hz, 2H), 7.28 (d, *J* = 8.4 Hz, 2H), 6.67 (d, *J* = 8.5 Hz, 2H), 5.87 (d, *J* = 6.7 Hz, 1H), 5.52 (d, *J* = 6.6 Hz, 1H), 4.79 – 4.72 (m, 1H), 2.19 – 2.10 (m, 2H), 2.05 – 1.91 (m, 2H), 1.85 – 1.65 (m, 4H). **^{13}C-NMR** (151 MHz, $CDCl_3$) δ 153.9, 147.8, 136.0, 132.6, 129.0, 126.8 (q, *J* = 3.8 Hz), 124.5 (q, *J* = 270.8 Hz), 123.3, 120.9 (q, *J* = 32.9 Hz), 113.0, 59.6, 52.6, 33.3, 33.3, 24.6, 24.5; **^{19}F-NMR** (564 MHz, $CDCl_3$) δ -61.44; **HRMS** (ESI) calculated for $C_{20}H_{19}{}^{79}BrF_3N_5Na$ $[M+Na]^+$: 488.0674, found: 488.0677; **HRMS** (ESI) calculated for $C_{20}H_{19}{}^{81}BrF_3N_5Na$ $[M+Na]^+$: 490.0653, found: 490.0661; **Enantiomeric ratio**: 90:10, determined by HPLC (Daicel Chirapak IF, isopropanol / hexanel = 30/70, flow rate 1.0 mL/min, T = 30 °C, λ = 254 nm): t_R = 4,81 min (major), t_R = 5.22 min (minor).

(*R*)-4-((1-benzyl-1*H*-tetrazol-5-yl)((4-(trifluoromethyl)phenyl)amino)methyl)benzonitrile (94):

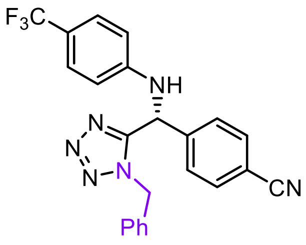

94

yield: 27.4 mg (63%); (Flash column chromatography eluent, petroleum ether/ethyl acetate/dichloromethane = 3/1/1); white foam; $[\alpha]_D^{25}$ = -48.3 (c 0.27, MeOH); **^{1}H-NMR** (600 MHz, $CDCl_3$) δ 7.57 (d, *J* = 8.2 Hz, 2H), 7.44 – 7.28 (m, 7H), 7.07 (d, *J* = 7.4 Hz, 2H), 6.41 (d, *J* = 8.4 Hz, 2H), 5.76 (d, *J* = 7.6 Hz, 1H), 5.59 (d, *J* = 15.5 Hz, 1H), 5.49 (d, *J* = 15.6 Hz, 1H), 5.12 (d, *J* = 7.6 Hz, 1H); **^{13}C-NMR** (151 MHz, $CDCl_3$) δ 154.1, 147.3, 141.2, 132.9, 132.5, 129.5 (d, *J* = 12.3 Hz), 128.0, 127.3, 126.8 (q, *J* = 3.7 Hz), 124.4 (q, *J* = 270.7 Hz), 121.5 (q, *J* = 32.8 Hz), 117.8, 113.2, 113.0, 52.2, 51.6; **^{19}F-NMR** (564 MHz, $CDCl_3$) δ -61.6; **HRMS** (ESI) calculated for $C_{23}H_{17}F_3N_6Na$ $[M+Na]^+$: 457,1364, found: 457.1366; **Enantiomeric ratio**: 71:29, determined by HPLC (Daicel Chirapak IB, isopropanol / hexanel = 30/70, flow rate 1.0 mL/min, T = 30 °C, λ = 254 nm): t_R = 10.85 min (major), t_R = 12,54 min (minor).

(*R*)-4-((1-butyl-1*H*-tetrazol-5-yl)((4-(trifluoromethyl)phenyl)amino)methyl)benzonitrile (95): yield: 23.6 mg (59%); (Flash column chromatography eluent, petroleum ether/ethyl acetate/dichloromethane = 6/1/1); white foam; $[\alpha]_D^{25}$ = -37.1 (c 0.24, MeOH); **^{1}H-NMR** (600 MHz,

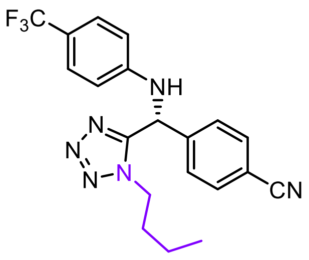

95

CDCl3) δ 7.69 (d, J = 8.3 Hz, 2H), 7.56 (d, J = 8.3 Hz, 2H), 7.41 (d, J = 8.5 Hz, 2H), 6.67 (d, J = 8.5 Hz, 2H), 5.92 (d, J = 7.0 Hz, 1H), 5.52 (d, J = 6.9 Hz, 1H), 4.31 – 4.25 (m, 2H), 1.85 – 1.68 (m, 2H), 1.34 – 1.27 (m, 2H), 0.90 (t, J = 7.4 Hz, 3H); **^{13}C-NMR** (151 MHz, CDCl3) δ 153.8, 147.4, 141.9, 133.1, 128.1, 126.9 (q, J = 3.7 Hz), 124.4 (q, J = 270.8 Hz), 121.5 (q, J = 32.9 Hz), 113.3, 113.1, 52.4, 47.7, 31.3, 19.6, 13.3.; **^{19}F-NMR** (564 MHz, CDCl3) δ -61.6; **HRMS** (ESI) calculated for $C_{20}H_{19}F_3N_6Na$ $[M+Na]^+$: 423.1521, found: 423,1517; **Enantiomeric ratio**: 84:16, determined by HPLC (Daicel Chirapak IF, isopropanol / hexanel = 10/90, flow rate 1.0 mL/min, T = 30 ºC, λ = 254 nm): t_R = 17,31 min (major), t_R = 15,66 min (minor).

Methyl (*R*)-2-(5-((4-bromophenyl)((4-(trifluoromethyl)phenyl)amino)methyl)-1H-tetrazol-1-yl)acetate (96): yield: 23.1 mg (49%); (Flash column chromatography eluent, petroleum ether/ethyl acetate/dichloromethane = 2/1/1); white foam; $[\alpha]_D^{25}$ = -59.8 (c 0.23, MeOH); **^{1}H-NMR** (600 MHz, CDCl3) δ 7.53 (d, J = 8.4 Hz, 2H), 7.41 (d, J = 8.6 Hz, 2H), 7.23 (d, J = 8.4 Hz, 2H), 6.67 (d, J = 8.5 Hz, 2H), 5.96 (d, J = 6.2 Hz, 1H), 5.31 (d, J = 6.1 Hz, 1H), 5.09 (d, J = 17.6 Hz, 1H), 4.92 (d, J = 17.6 Hz, 1H), 3.71 (s, 3H); **^{13}C-NMR** (151 MHz, CDCl3) δ 170.1, 165.7, 155.0, 147.8, 134.9, 132.7, 128.9 (s), 126.8 (q, J = 3.8 Hz), 124.5 (q, J = 271.0 Hz), 123.6, 121.4 (q, J = 32.9 Hz), 113.2 (d, J = 8.6 Hz), 53.4, 52.8, 48.4; **^{19}F-NMR** (564 MHz, CDCl3) δ -61.5; **HRMS** (ESI) calculated for $C_{18}H_{15}{}^{79}BrF_3N_5NaO_2$ $[M+Na]^+$: 492.0259, found: 492.0252; **HRMS** (ESI) calculated for $C_{18}H_{15}{}^{81}BrF_3N5NaO_2$ $[M+Na]^+$: 494.0238, found: 494.0236; **Enantiomeric ratio**: 96.5:3.5, determined by HPLC (Daicel Chirapak IF, isopropanol / hexanel = 30/70, flow rate 1.0 mL/min, T = 30 ºC, λ = 254 nm): t_R = 8.75 min (major), t_R = 7.26 min (minor).

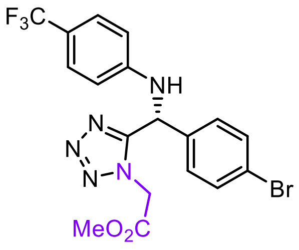

96

Ethyl (*R*)-2-(5-((4-bromophenyl)((4-(trifluoromethyl)phenyl)amino)methyl)-1H-tetrazol-1-yl)acetate (97): yield: 22.3 mg (46%); (Flash column chromatography eluent, petroleum ether/ethyl acetate/dichloromethane = 3/1/1); white foam; $[\alpha]_D^{25}$ = -67.3 (c 0.22, MeOH); **^{1}H-NMR** (600 MHz, CDCl3) δ 7.53 (d, J = 8.4 Hz, 2H), 7.40 (d, J = 8.6 Hz, 2H), 7.24 (d, J = 8.4 Hz, 2H), 6.67 (d, J = 8.5 Hz, 2H), 5.97 (d, J = 6.3 Hz, 1H), 5.35 (d, J = 6.3 Hz, 1H), 5.06 (d, J = 17.6 Hz, 1H), 4.90 (d, J = 17.6 Hz, 1H), 4.22 – 4.13 (m, 1H), 1.24 (t, J = 7.1 Hz, 3H); **^{13}C-NMR** (151 MHz, CDCl3) δ 170.0, 165.3, 155.1, 147.9, 134.9, 132.7, 128.9, 126.8 (q, J = 3.8 Hz), 124.5 (q, J = 270.8 Hz), 123.5, 121.3 (q, J = 32.8 Hz), 113.2, 63.2, 52.7, 48.6, 13.9; **^{19}F-NMR** (564 MHz, CDCl3) δ -61.5; **HRMS** (ESI) calculated for $C_{19}H_{17}{}^{79}BrF_3N_5NaO_2$ $[M+Na]^+$: 506.0415, found: 506.0411; **HRMS** (ESI) calculated for $C_{19}H_{17}{}^{81}BrF_3N_5NaO_2$ $[M+Na]^+$: 508.0395, found: 508.0391; **Enantiomeric ratio**: 97:3, determined by HPLC (Daicel Chirapak IF, isopropanol

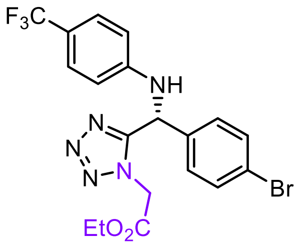

97

/ hexanel = 10/90, flow rate 1.0 mL/min, T = 30 ºC, λ = 254 nm): t_R = 9.23 min (major), t_R = 8.71 min (minor).

(*R*)-*N*-((4-bromophenyl)(1-(2,4,4-trimethylpentan-2-yl)-1*H*-tetrazol-5-yl)methyl)-4(trifluoro-methyl)aniline (98): yield: 40.3 mg (79%); (Flash column chromatography eluent, petroleum ether/ethyl acetate/dichloromethane = 6/1/1); white foam; $[\alpha]_D^{25}$ = -113.6 (c 0.40, MeOH); **^{1}H-NMR** (600 MHz, $CDCl_3$) δ 7.50 (d, *J* = 8.5 Hz, 2H), 7.40 (d, *J* = 8.4 Hz, 2H), 7.34 (d, *J* = 8.4 Hz, 2H), 6.69 (d, *J* = 8.4 Hz, 2H), 6.14 (d, *J* = 8.7 Hz, 1H), 5.34 (d, *J* = 8.8 Hz, 1H), 1.96 (s, 2H), 1.86 (s, 3H), 1.82 (s, 3H), 0.69 (s, 9H); **^{13}C-NMR** (151 MHz, $CDCl_3$) δ 154.6 , 147.9, 136.5, 132.3, 129.4, 126.8 (q, *J* = 3.7 Hz), 124.5 (q, *J* = 270.7 Hz), 123.0, 121.0 (q, *J* = 32.8 Hz), 113.2, 65.7, 54.0, 53.5, 31.6, 30.7, 30.5, 30.1; **^{19}F-NMR** (564 MHz, $CDCl_3$) δ -61.4; **HRMS** (ESI) calculated for $C_{23}H_{27}{}^{79}BrF_3N_5Na$ $[M+Na]^+$: 532,1300, found: 532,1296; **HRMS** (ESI) calculated for $C_{23}H_{27}{}^{81}BrF_3N_5Na$ $[M+Na]^+$: 534,1279, found: 534,1286; **Enantiomeric ratio**: 91:9, determined by HPLC (Daicel Chirapak IF, isopropanol / hexanel = 20/70, flow rate 1.0 mL/min, T = 30 ºC, λ = 254 nm): t_R = 7.09 min (major), t_R = 6.59 min (minor).

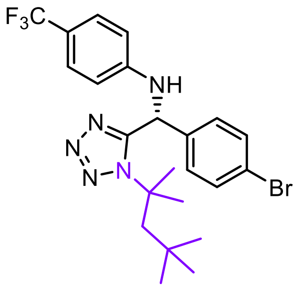

98

3.4. Asymmetric Ugi-4CRs and Ugi-azide Reactions in Gram-Scale

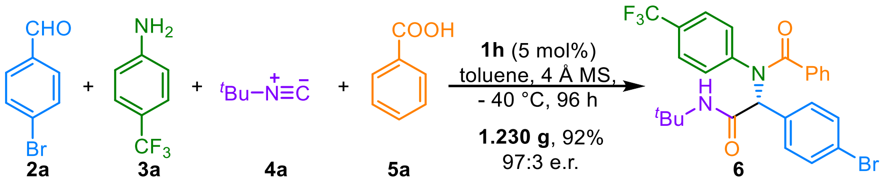

General Procedure E: Synthesis of chiral α-acylamino amide **6** in gram-scale

A 50-mL oven-dried tube was charged with 4-bromobenzaldehyde **2a** (3.75 mmol), 4-trifluroaniline **3a** (2.50 mmol), catalyst Λ-**1h** (0.125 mmol), 4 Å molecular sieves (1.0 g), and toluene (8.0 mL) at room temperature and stirred for 30 min. Then a solution of benzoic acid **5a** (12.50 mmol) in toluene (1.0 mL) was added in one portion. The mixture was cooled to -40 °C and stirred for another 30 min. A solution of *tert*-butyl isocyanide **4** (7.50 mmol) in toluene (1.0 mL) was added in one portion and the resulting solution was stirred vigorously for 96 h. The reaction was then quenched with pre-cooled NEt_3 (-40 °C, 15.0 mmol). The mixture was purified by flash column chromatography (silica gel, petroleum ether/EtOAc = 5:1) to give the enantioenriched α-acylamino amide **6** (1.230 g, 92% yield, 97:3 e.r.).

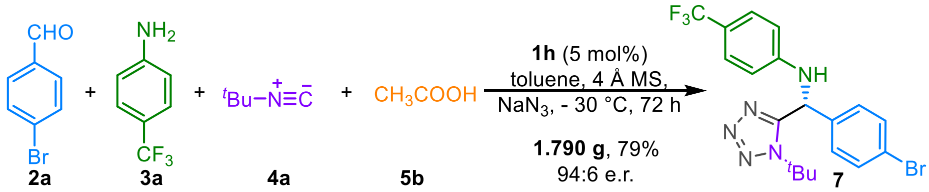

General Procedure F: Synthesis of chiral α-aminotetrazole **7** in gram-scale

A 100-mL oven-dried tube was charged with 4-bromobenzaldehyde **2a** (4.50 mmol), 4-trifluroaniline **3a** (3.0 mmol), scalemic catalyst **1h** (0.15 mmol), NaN_3 (9.0 mmol), 4 Å molecular sieves (3.0 g), and toluene (30.0 mL) at room temperature and stirred for 30 min. Then acetic acid **5b** (12.0 mmol) was added in one portion. The mixture was cooled to -30 °C and stirred for another 30 min. The *tert*-butyl isocyanide **4a** (9.0 mmol) was added in one portion and the resulting solution was stirred vigorously for 72 h. The reaction was then quenched with pre-cooled NEt_3 (-30 °C, 15.0 mmol). The mixture was purified by flash column chromatography (silica gel, petroleum ether/EtOAc/CH_2Cl_2 = 6:1:1) to give the enantioenriched α-aminotetrazole **7** (1.790 g, 79% yield, 94:6 e.r.).

3.5. Control experiments

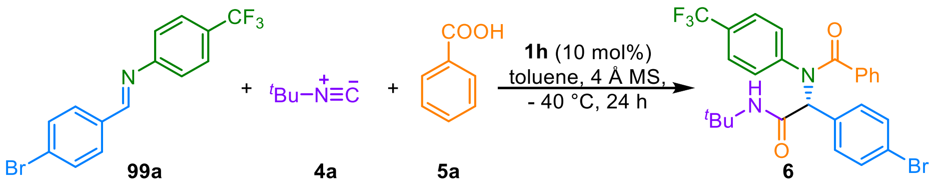

General Procedure G: Synthesis of chiral α-acylamino amide **6** via asymmetric Ugi-4CR of aldimine **99a**

A 10-mL oven-dried tube was charged with aldimine **99a** (0.10 mmol), catalyst Λ-**1h** (0.01 mmol), 4 Å molecular sieves (100 mg), and toluene (2.0 mL) at room temperature and stirred for 30 min. Then benzoic acid **5a** (0.50 mmol) was added in one portion. The mixture was cooled to -40 °C and stirred for another 30 min. The *tert*-butyl isocyanide **4a** (0.30 mmol) was added in one portion and the resulting solution was stirred vigorously for 24 h. The reaction was then quenched with pre-cooled NEt_3 (-40 °C, 1.0 mmol). The mixture was purified by flash column chromatography (silica gel, petroleum ether/EtOAc/CH_2Cl_2 = 6:1:1) to give the enantioenriched α-acylamino amide **6** (82% yield, 97:3 e.r.).

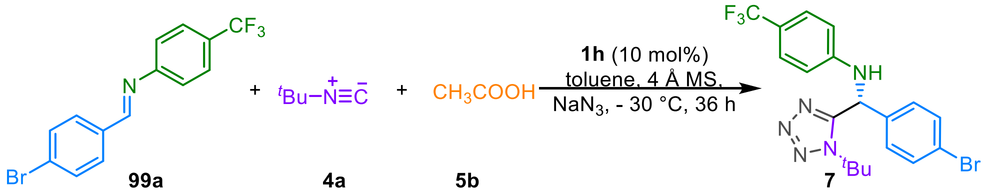

General Procedure H: Synthesis of chiral α-aminotetrazole **7** via asymmetric Ugi-azide reaction of aldimine **99a**

A 10-mL oven-dried tube was charged with aldimine **99a** (0.10 mmol), catalyst Λ-**1h** (0.01 mmol), NaN_3 (0.30 mmol), 4 Å molecular sieves (100 mg), and toluene (2.0 mL) at room temperature and stirred for 30 min. Then acetic acid **5b** (0.40 mmol) was added in one portion. The mixture was cooled to -30 °C and stirred for another 30 min. The *tert*-butyl isocyanide **4a** (0.30 mmol) was added in one portion and the resulting solution was stirred vigorously for 36 h. The reaction was then quenched with pre-cooled NEt_3 (-30 °C, 1.0 mmol). The mixture was purified by flash column chromatography (silica gel, petroleum ether/EtOAc/CH_2Cl_2 = 6:1:1) to give the enantioenriched α-aminotetrazole **7** (81% yield, 94:6 e.r.).

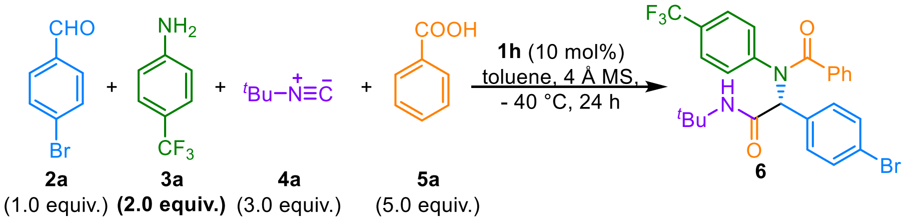

General Procedure I: Synthesis of chiral α-acylamino amide **6** via asymmetric Ugi-4CR of excess aniline **3a**

A 10-mL oven-dried tube was charged with aldehyde **2a** (0.10 mmol), amine **3a** (0.20 mmol), catalyst Λ-**1h** (0.01 mmol), 4 Å molecular sieves (100 mg), and toluene (2.0 mL) at room temperature and stirred for 30 min. Then benzoic acid **5a** (0.50 mmol) was added in one portion. The mixture was cooled to -40 °C and stirred for another 30 min. The *tert*-butyl isocyanide **4a** (0.30 mmol) was added in one portion and the resulting solution was stirred vigorously for 24 h. The reaction was then quenched with pre-cooled NEt_3 (-40 °C, 1.0 mmol). The mixture was purified by flash column chromatography (silica gel, petroleum ether/EtOAc/CH_2Cl_2 = 6:1:1) to give the enantioenriched α-acylamino amide **6** (38% yield, 91:9 e.r.).

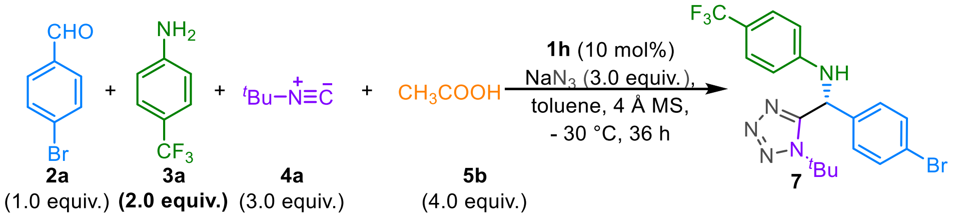

General Procedure J: Synthesis of chiral α-aminotetrazole **7** via asymmetric Ugi-azide reaction of excess aniline **3a**

A 10-mL oven-dried tube was charged with aldehyde **2a** (0.10 mmol), amine **3a** (0.20 mmol), catalyst Λ-**1h** (0.01 mmol), NaN_3 (0.30 mmol), 4 Å molecular sieves (100 mg), and toluene (2.0 mL) at room temperature and stirred for 30 min. Then acetic acid **5b** (0.40 mmol) was added in one portion. The mixture was cooled to -30 °C and stirred for another 30 min. The *tert*-butyl isocyanide **4a** (0.30 mmol) was added in one portion and the resulting solution was stirred vigorously for 36 h. The reaction was then quenched with pre-cooled NEt_3 (-30 °C, 1.0 mmol). The mixture was purified by flash column chromatography (silica gel, petroleum ether/EtOAc/CH_2Cl_2 = 6:1:1) to give the enantioenriched α-aminotetrazole **7** (46% yield, 87:13 e.r.).

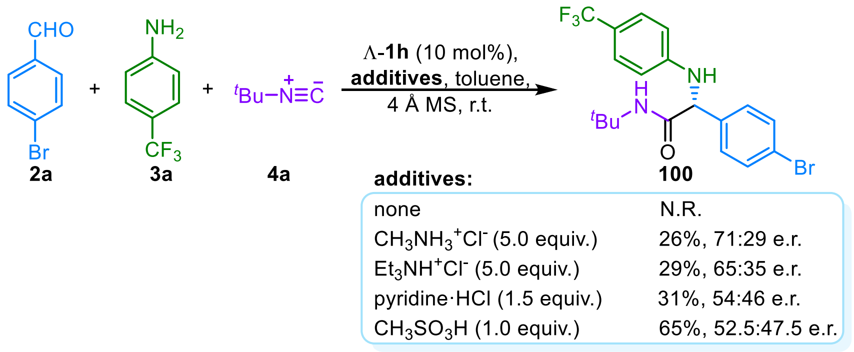

General Procedure K: Studies of the acid effect on Ugi-3CR

A 10-mL oven-dried tube was charged with aldehyde **2a** (0.15 mmol), amine **3a** (0.10 mmol), catalyst Λ-**1h** (0.01 mmol), 4 Å molecular sieves (100 mg), and toluene (2.0 mL) at room temperature and stirred for 30 min. Then the methylamine hydrochloride (0.50 mmol) was added in one portion. The mixture was stirred for another 30 min. The *tert*-butyl isocyanide **4a** (0.30 mmol) was added in one portion and the resulting solution was stirred vigorously for 24 h. The reaction was then quenched with NEt_3 (2.0 mmol). The mixture was purified by flash column chromatography (silica gel, petroleum ether/EtOAc/CH_2Cl_2 = 6:1:1) to give the enantioenriched amino amide **100** (26% yield, 71:29 e.r.).

(*R*)-2-(4-bromophenyl)-*N*-(tert-butyl)-2-((4-(trifluoromethyl)phenyl)amino)acetamide (100):

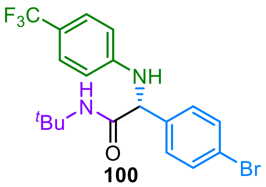

100

yield: 11.2 mg (26%); (Flash column chromatography eluent, petroleum ether/ethyl acetate/dichloromethane = 8/1/1); slight yellow oil; $[\alpha]_D^{25}$ = -40.6 (c 0.12, MeOH); **^{1}H-NMR** (600 MHz, $CDCl_3$)) δ 7.52 (d, *J* = 6.8 Hz, 2H), 7.37 (d, *J* = 6.8 Hz, 2H), 7.30 (d, *J* = 6.8 Hz, 2H), 6.57 (d, *J* = 6.9 Hz, 2H), 5.86 (s, 1H), 5.23 (s, 1H), 4.64 (s, 1H), 1.30 (s, 9H); **^{13}C-NMR** (151 MHz, $CDCl_3$) δ 168.76, 148.81, 137.96, 132.64, 128.81, 126.69 (q, *J* = 3.2 Hz), 124.79 (q, *J* = 270.6 Hz), 122.77, 120.52 (q, *J* = 32.7 Hz), 113.14, 62.62, 51.88, 28.62; **^{19}F-NMR** (564 MHz, $CDCl_3$) δ -61.3; **HRMS** (ESI) calculated for $C_{19}H_{20}{}^{79}BrF_3N_2NaO$ $[M+Na]^+$: 451.0609, found: 451.0612; $C_{19}H_{20}{}^{81}BrF_3N_2NaO$ $[M+Na]^+$: 453.0588, found: 453.0592; **Enantiomeric ratio**: 71:29, determined by HPLC (Daicel Chirapak IF, isopropanol / hexanel = 30/70, flow rate 1.0 mL/min, T = 30 °C, λ = 254 nm): t_R = 3.76 min (major), t_R = 4.47 min (minor).

3.6. NMR spectra

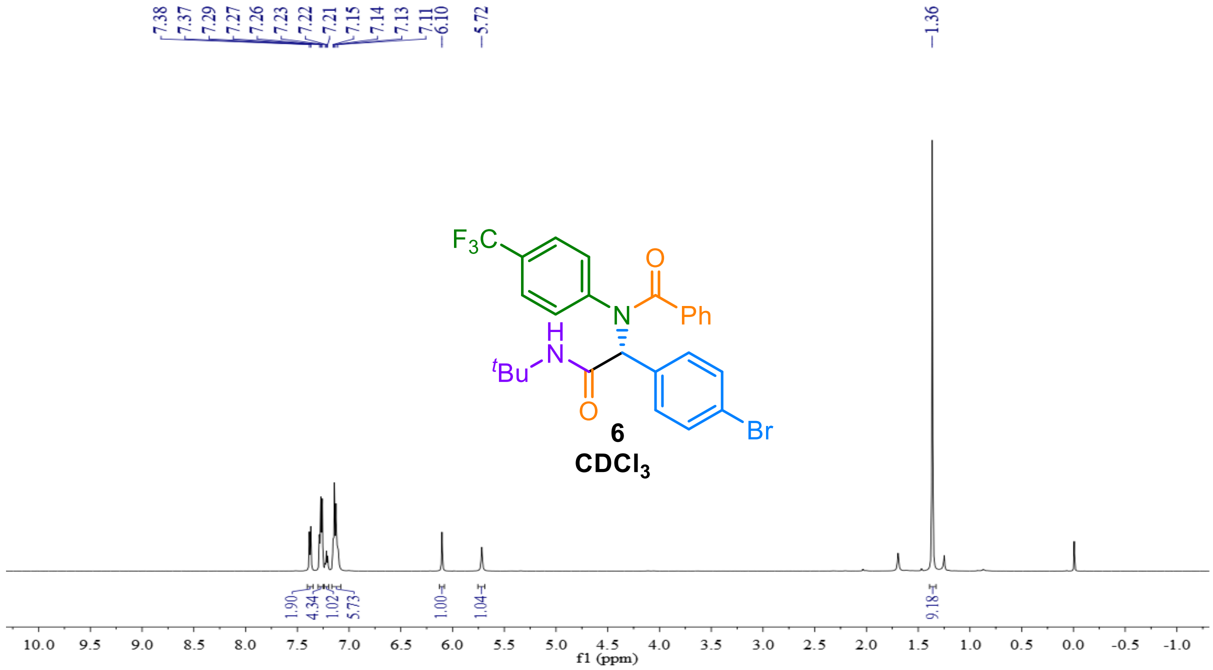

Supplementary Fig. 17. ^{1}H NMR spectrum of **6**. The sample has been recorded in 600 MHz, $CDCl_3$ at 25 °C.

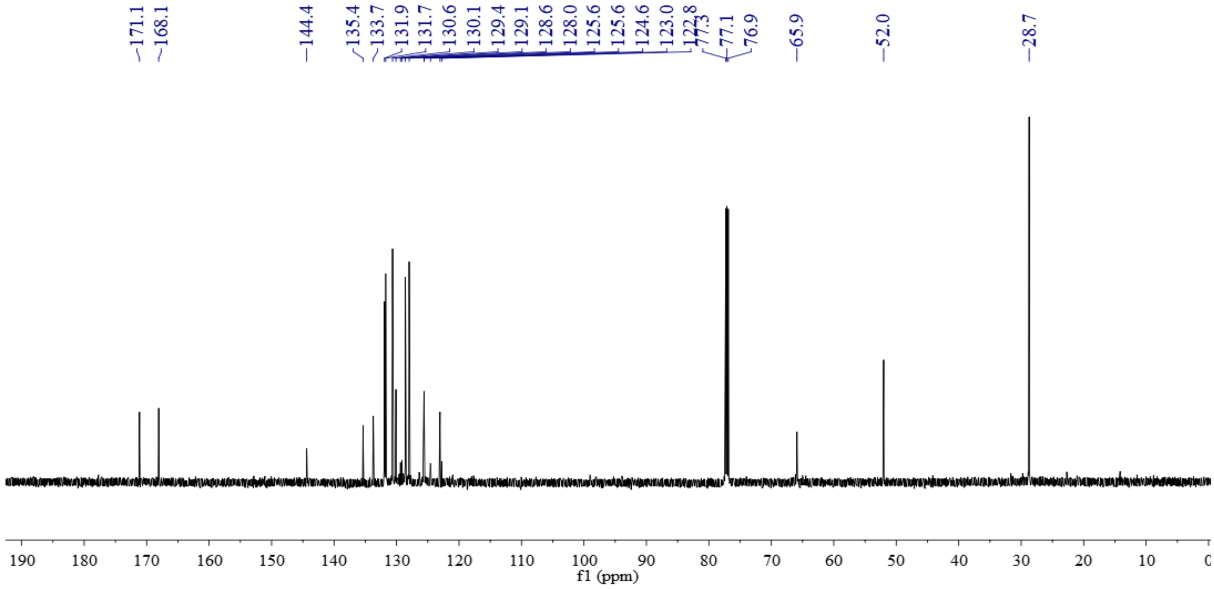

Supplementary Fig. 18.^{13}C NMR spectrum of **6**. The sample has been recorded in 151 MHz, $CDCl_3$ at 25 °C.

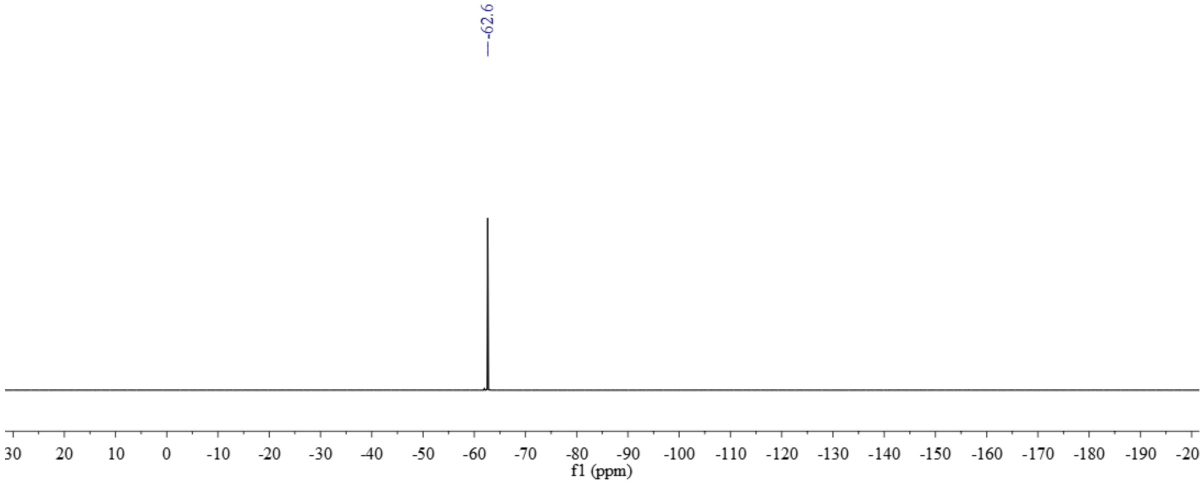

Supplementary Fig. 19. ^{31}F NMR spectrum of **6**. The sample has been recorded in 564 MHz, $CDCl_3$ at 25 °C.

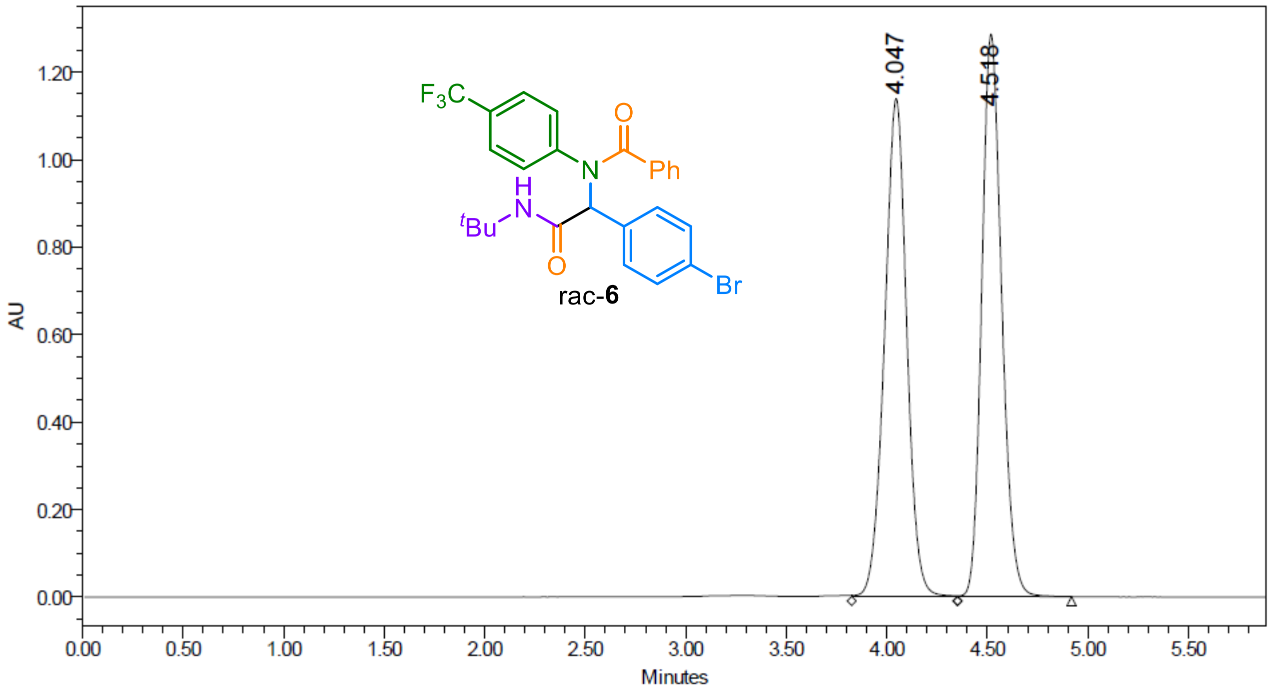

	RT (min)	Peak Type	Area (μV*sec)	% Area	Height (μV)	% Height	Integration Type	Points Across Peak	Start Time (min)	End Time (min)
1	4.047	Unknown	8713443	49.63	1137555	46.95	VV	316	3.825	4.352
2	4.518	Unknown	8841795	50.37	1285399	53.05	VB	340	4.352	4.918

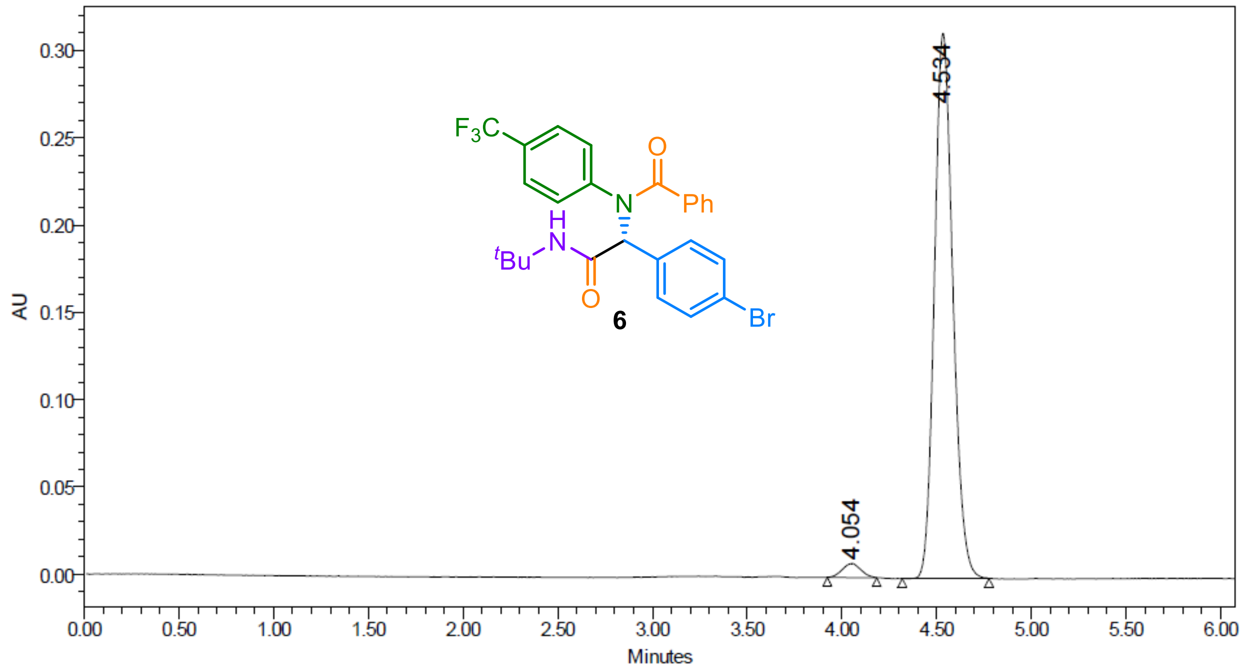

	RT (min)	Peak Type	Area (μV*sec)	% Area	Height (μV)	% Height	Integration Type	Points Across Peak	Start Time (min)	End Time (min)
1	4.054	Unknown	54811	2.53	8027	2.51	Bb	156	3.923	4.183
2	4.534	Unknown	2112788	97.47	312023	97.49	bB	275	4.318	4.777

Supplementary Fig. 20. HPLC of product **6**.

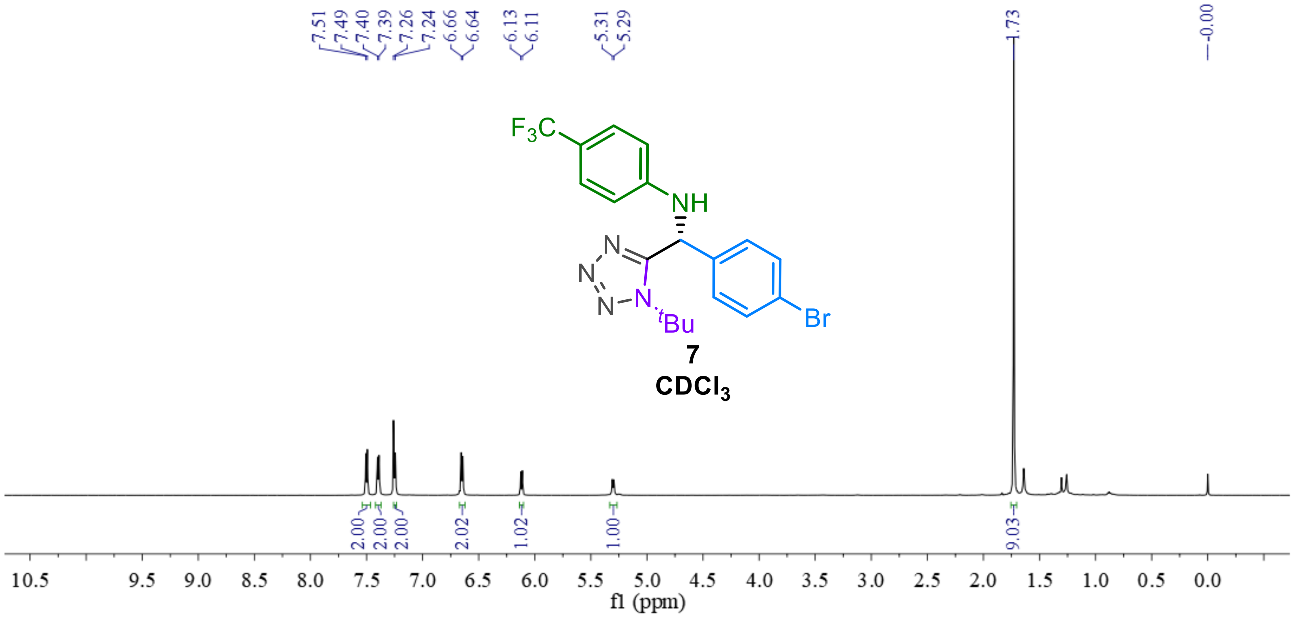

Supplementary Fig. 21. ^{1}H NMR spectrum of **7**. The sample has been recorded in 600 MHz, $CDCl_3$ at 25 °C.

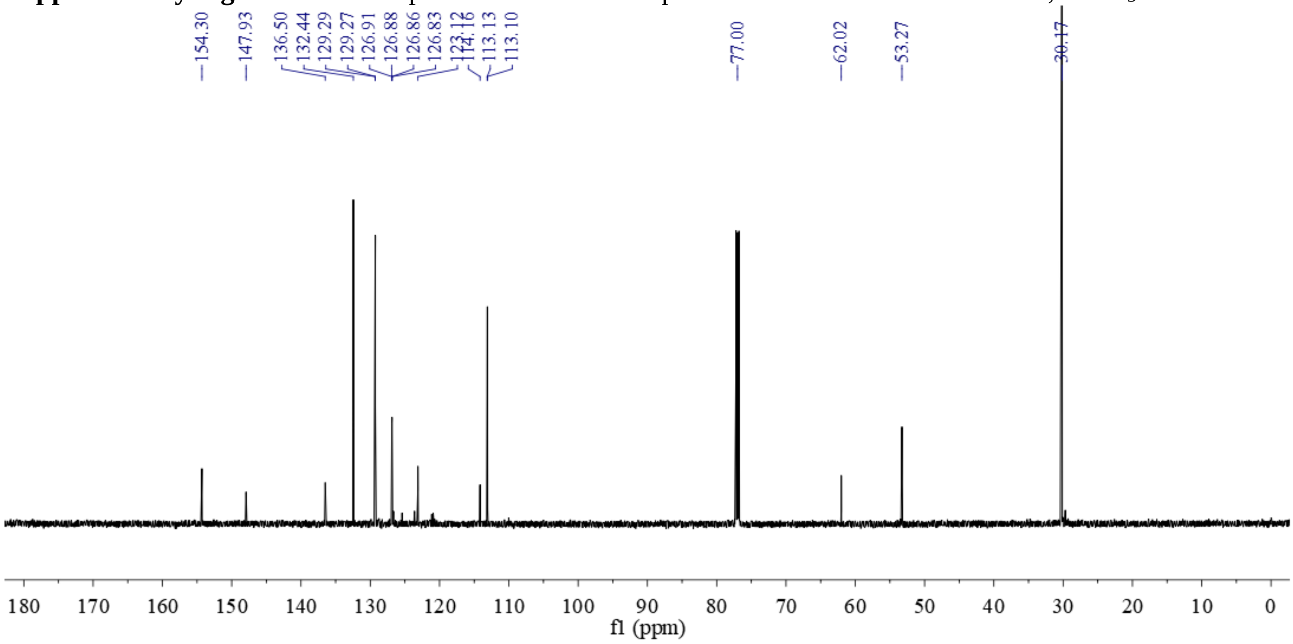

Supplementary Fig. 22.^{13}C NMR spectrum of **7**. The sample has been recorded in 151 MHz, $CDCl_3$ at 25 °C.

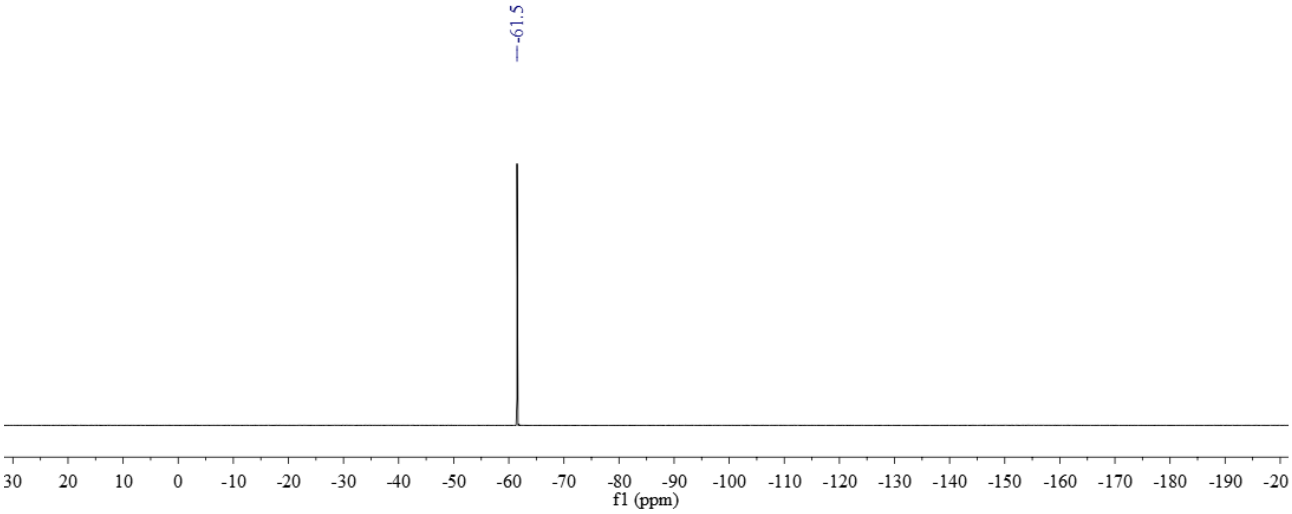

Supplementary Fig. 23. [31]F NMR spectrum of **7**. The sample has been recorded in 564 MHz, $CDCl_3$ at 25 °C.

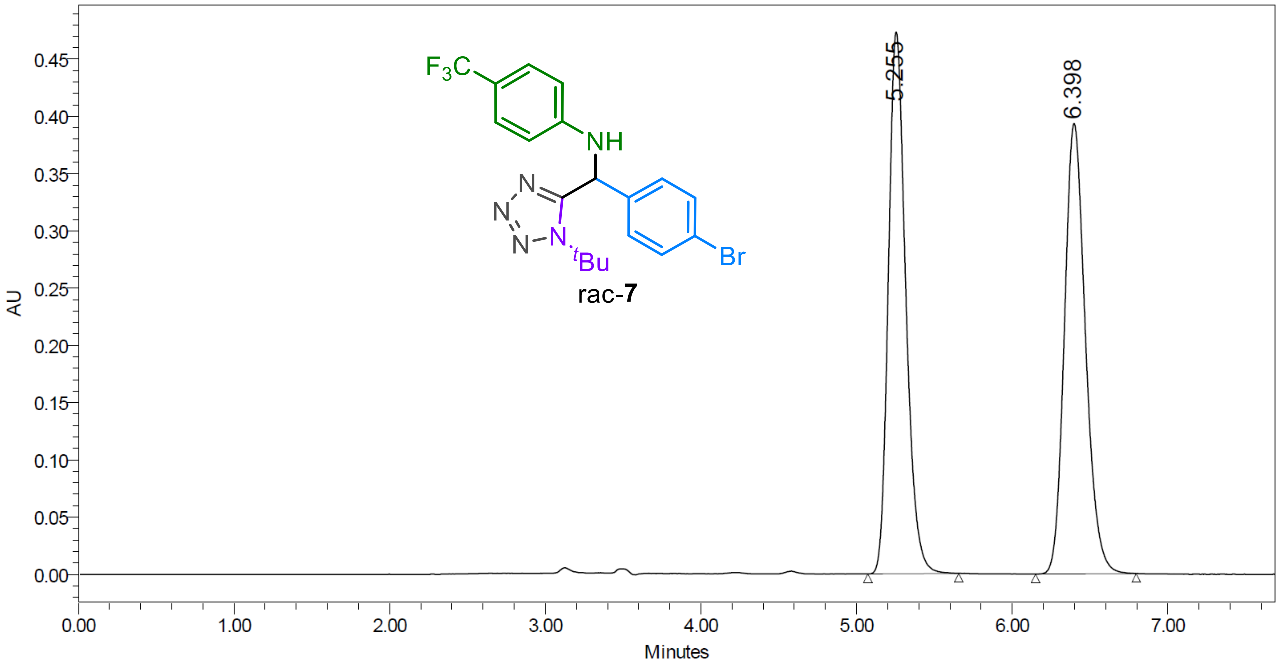

	RT (min)	Area (µV*sec)	% Area	Height (µV)	% Height
1	5.255	3673074	49.83	473207	54.61
2	6.398	3697404	50.17	393365	45.39

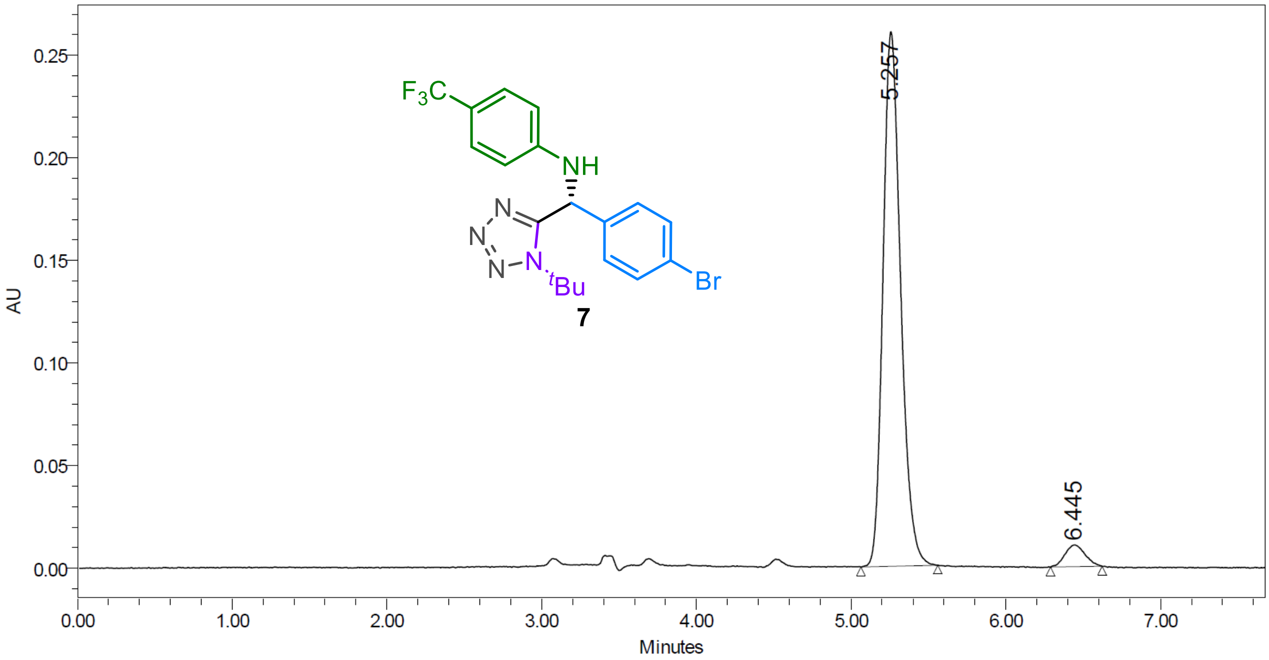

	RT (min)	Area (µV*sec)	% Area	Height (µV)	% Height
1	5.257	2061848	95.58	260532	96.12
2	6.445	95285	4.42	10525	3.88

Supplementary Fig. 24. HPLC of product **7**.

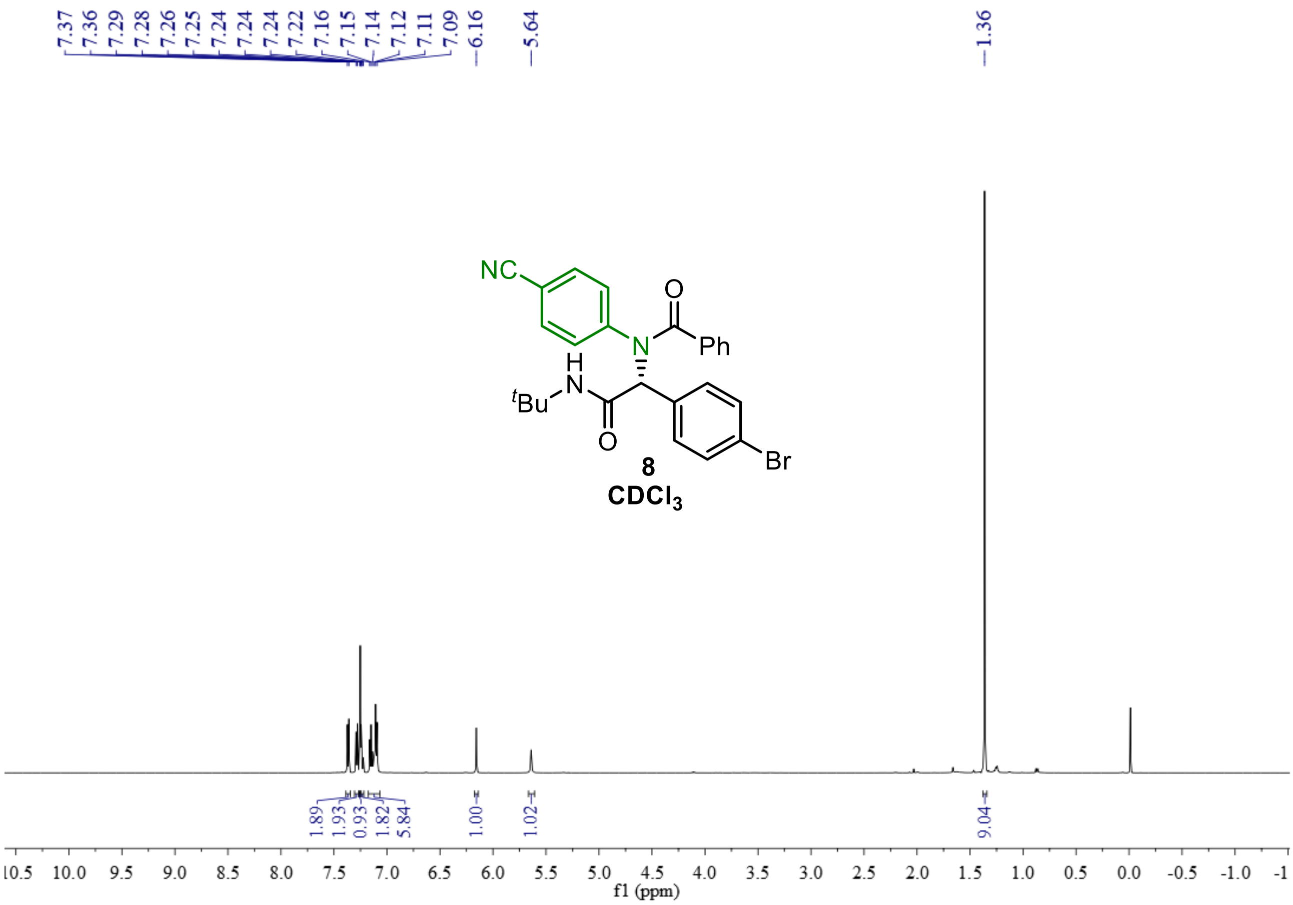

Supplementary Fig. 25. ¹H NMR spectrum of **8**. The sample has been recorded in 600 MHz, $CDCl_3$ at 25 °C.

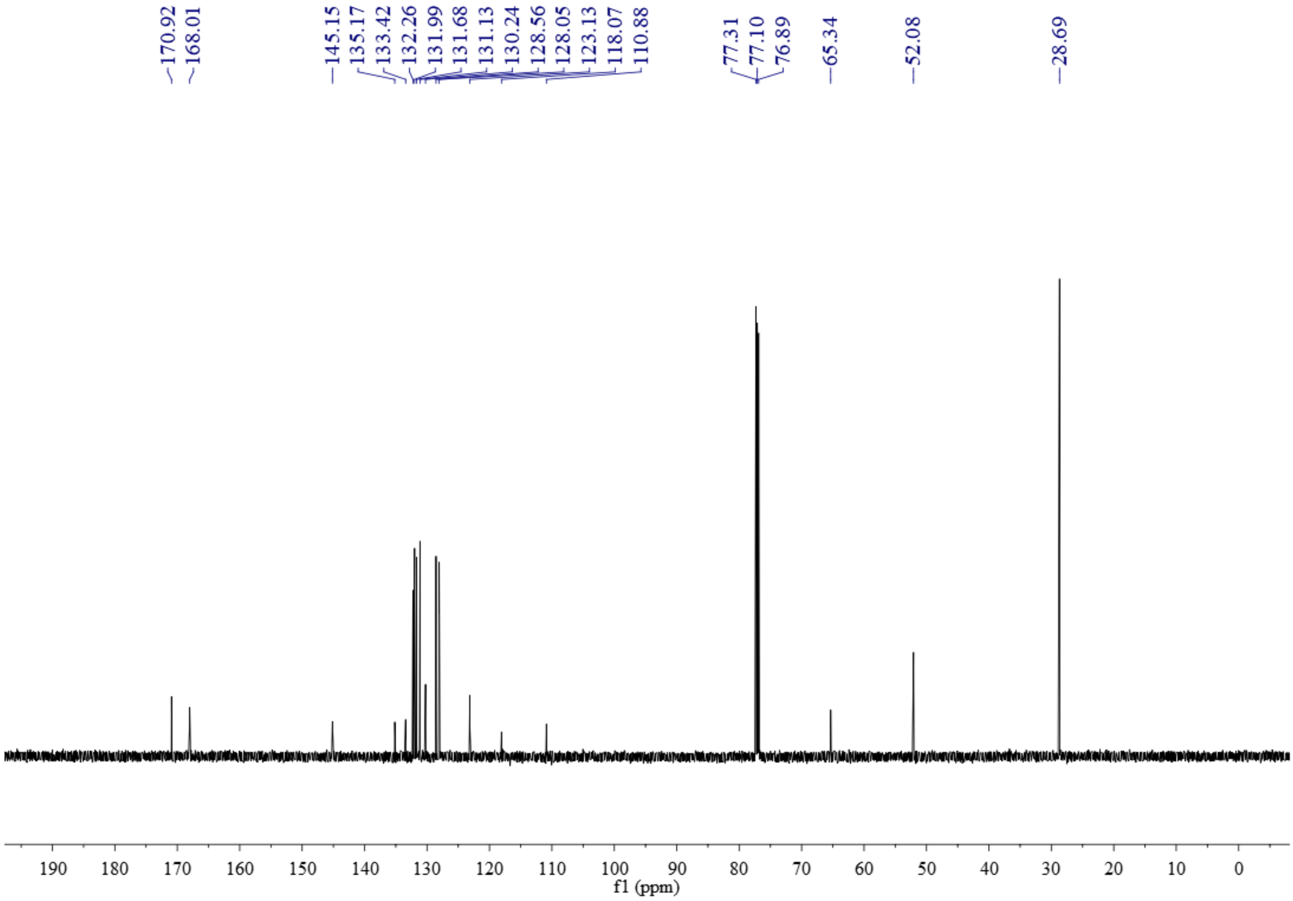

Supplementary Fig. 26.^{13}C NMR spectrum of **8**. The sample has been recorded in 151 MHz, $CDCl_3$ at 25 °C.

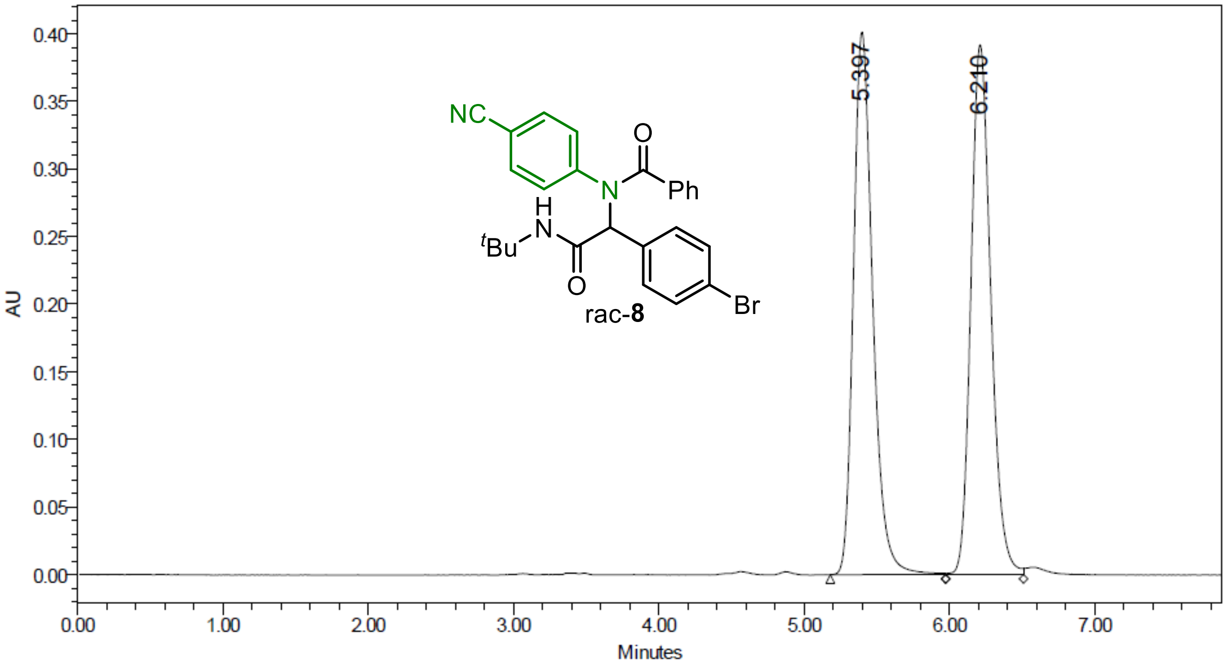

	RT (min)	Peak Type	Area (µV*sec)	% Area	Height (µV)	% Height	Integration Type	Points Across Peak	Start Time (min)	End Time (min)
1	5.397	Unknown	3702034	49.89	401378	50.61	BV	476	5.180	5.973
2	6.210	Unknown	3717624	50.11	391728	49.39	VV	321	5.973	6.508

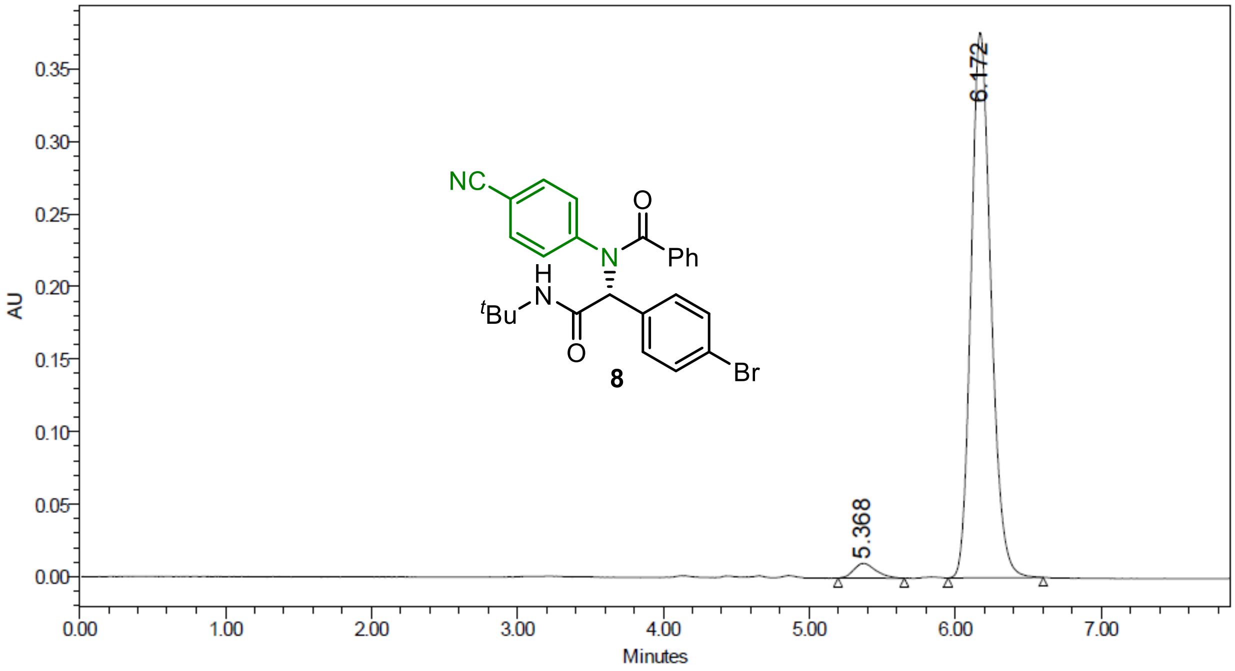

	RT (min)	Peak Type	Area (µV*sec)	% Area	Height (µV)	% Height	Integration Type	Points Across Peak	Start Time (min)	End Time (min)
1	5.368	Unknown	107195	2.92	10207	2.64	BB	272	5.198	5.652
2	6.172	Unknown	3561662	97.08	375727	97.36	BB	391	5.952	6.603

Supplementary Fig. 27. HPLC of product **8**.

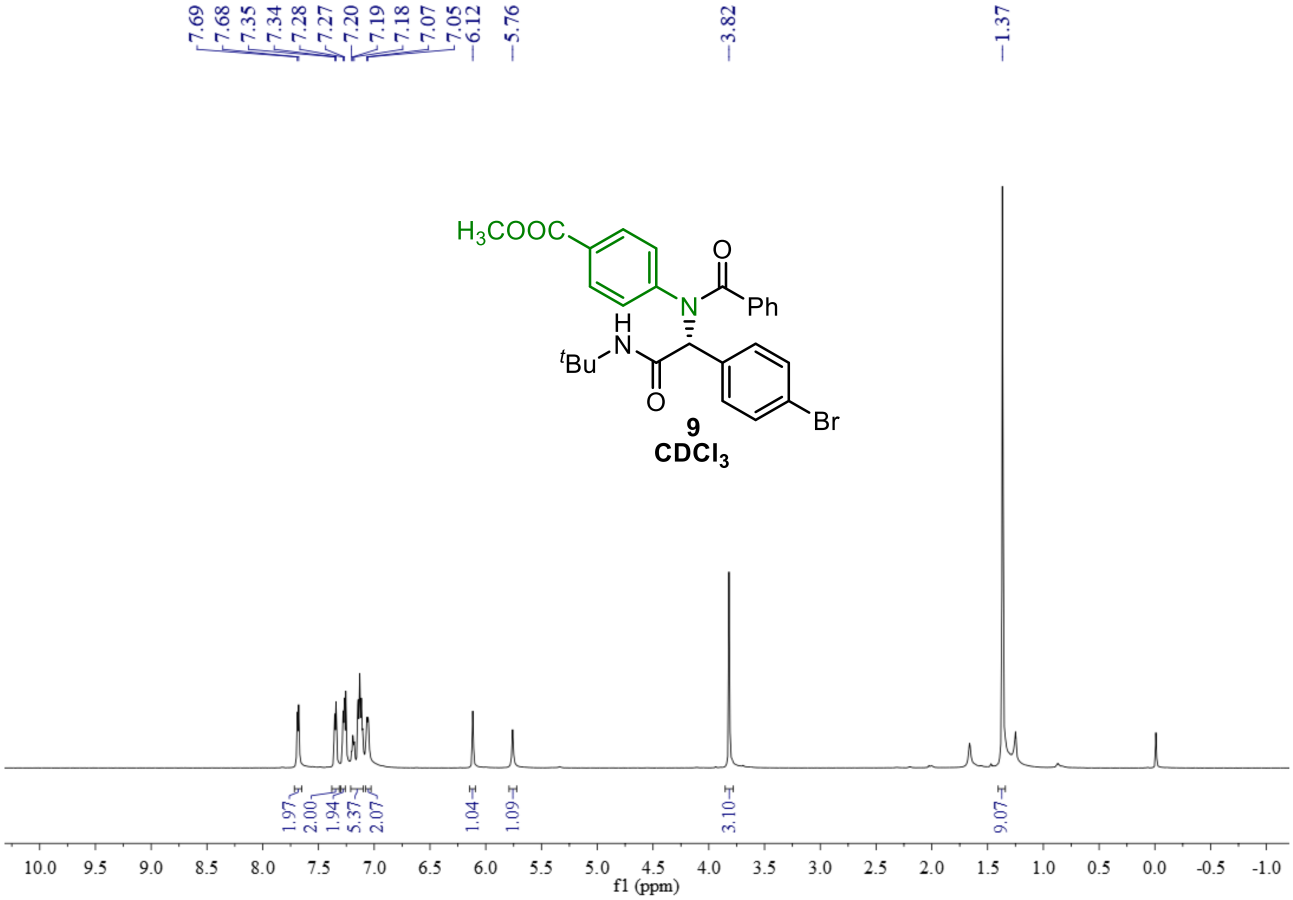

Supplementary Fig. 28. ^{1}H NMR spectrum of **9**. The sample has been recorded in 600 MHz, $CDCl_3$ at 25 °C.

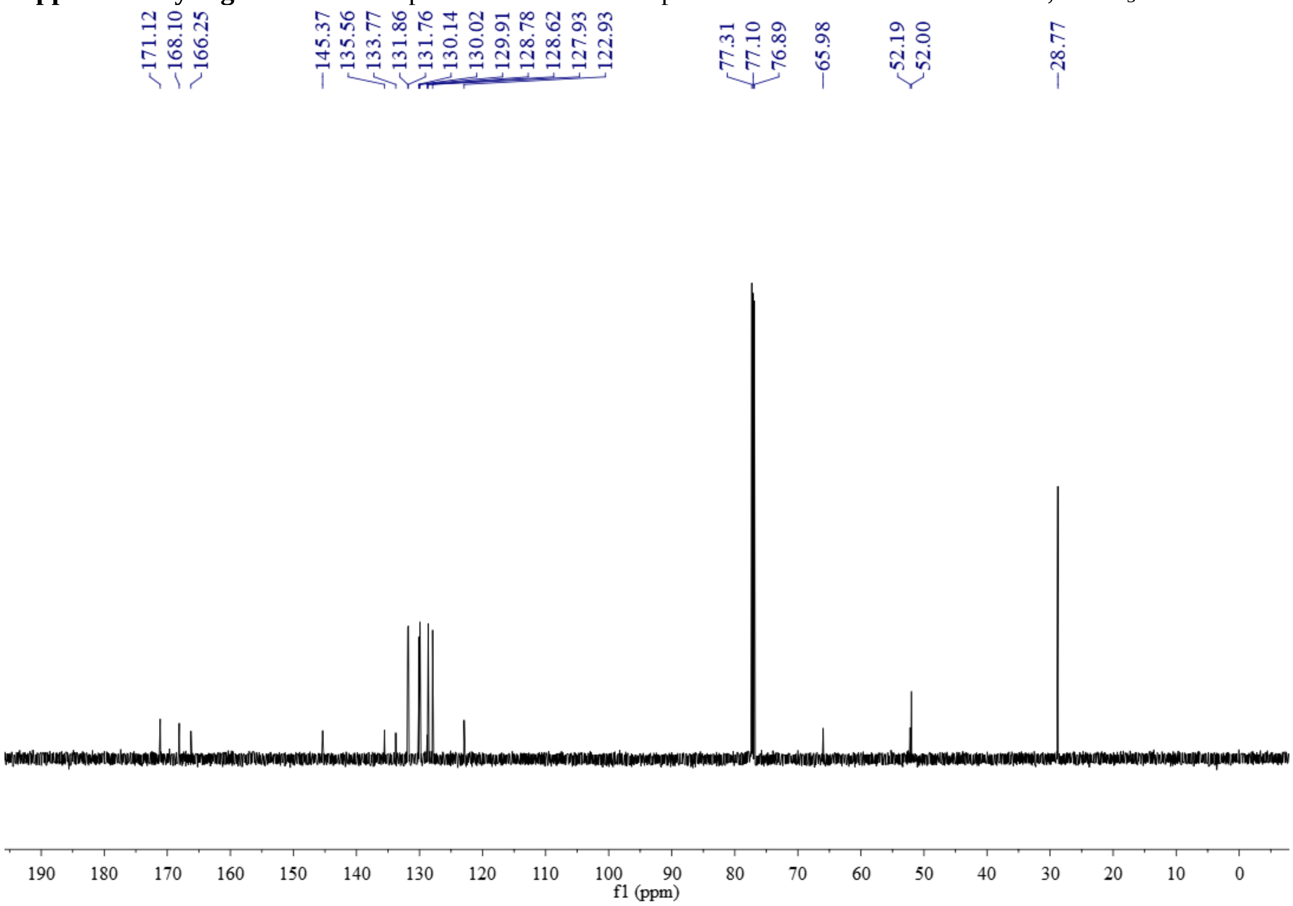

Supplementary Fig. 29.^{13}C NMR spectrum of **9**. The sample has been recorded in 151 MHz, $CDCl_3$ at 25 °C.

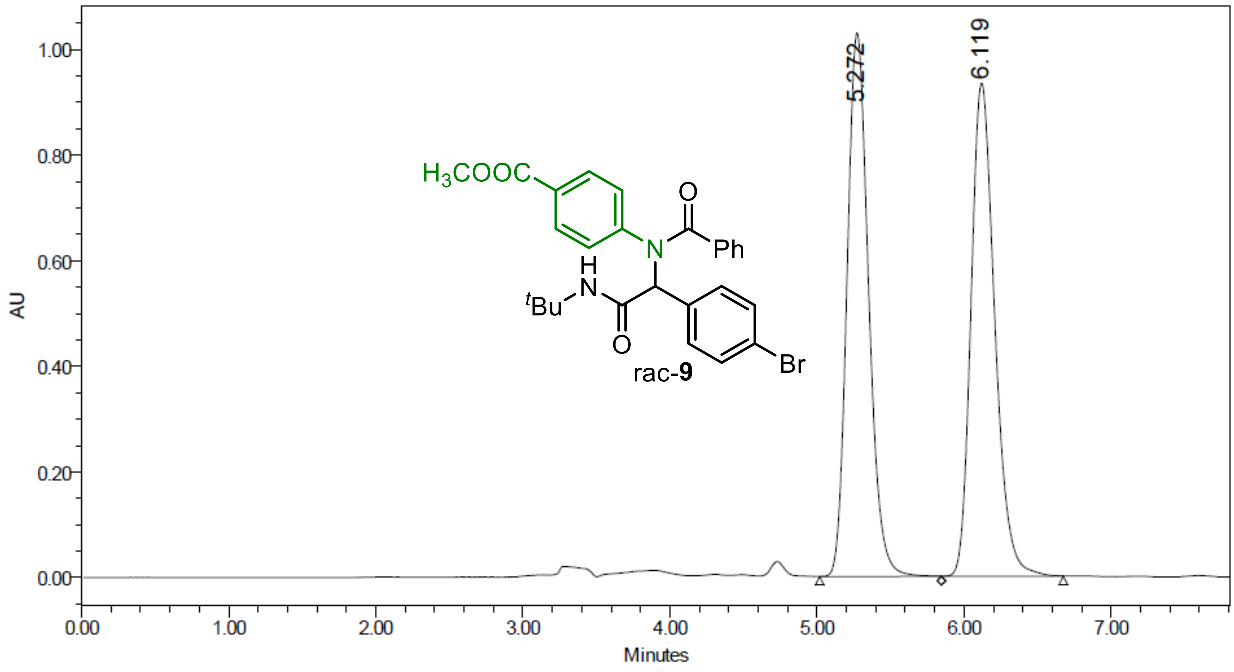

	RT (min)	Peak Type	Area (µV*sec)	% Area	Height (µV)	% Height	Integration Type	Points Across Peak	Start Time (min)	End Time (min)
1	5.272	Unknown	10467095	50.04	1029657	52.44	BV	497	5.018	5.847
2	6.119	Unknown	10451623	49.96	933793	47.56	VB	497	5.847	6.675

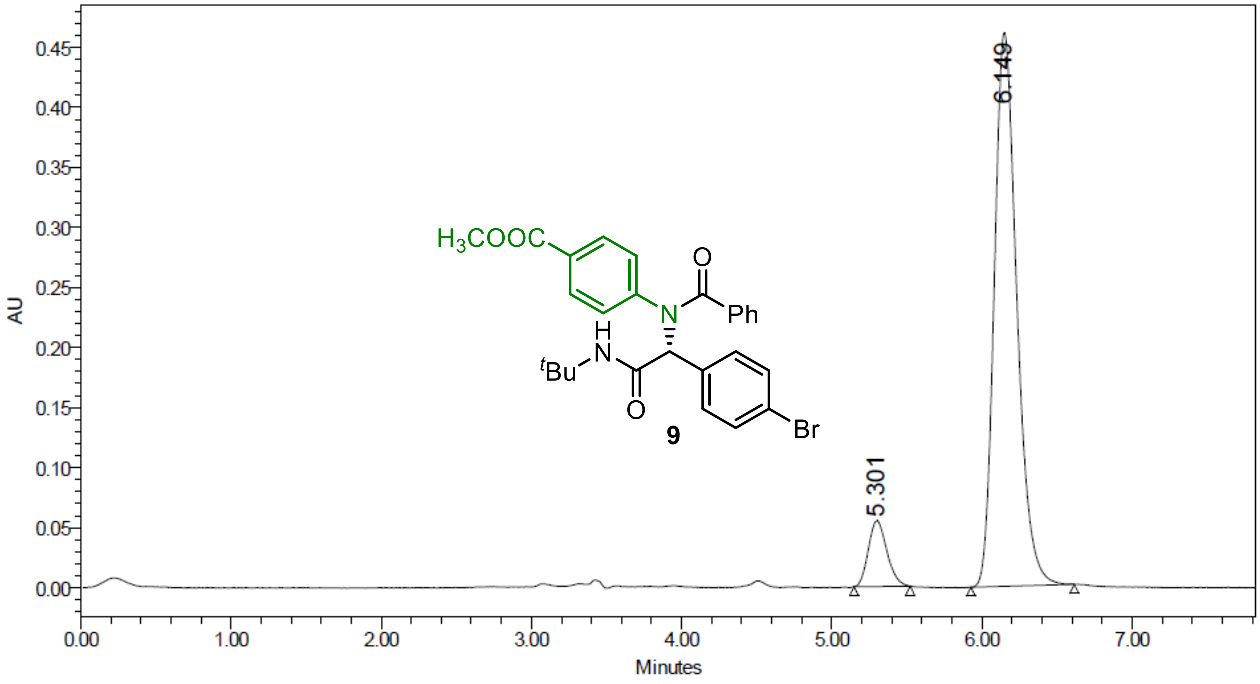

	RT (min)	Peak Type	Area (µV*sec)	% Area	Height (µV)	% Height	Integration Type	Points Across Peak	Start Time (min)	End Time (min)
1	5.301	Unknown	471009	8.94	54801	10.62	bb	224	5.148	5.522
2	6.149	Unknown	4798325	91.06	461375	89.38	BB	413	5.927	6.615

Supplementary Fig. 30. HPLC of product **9**.

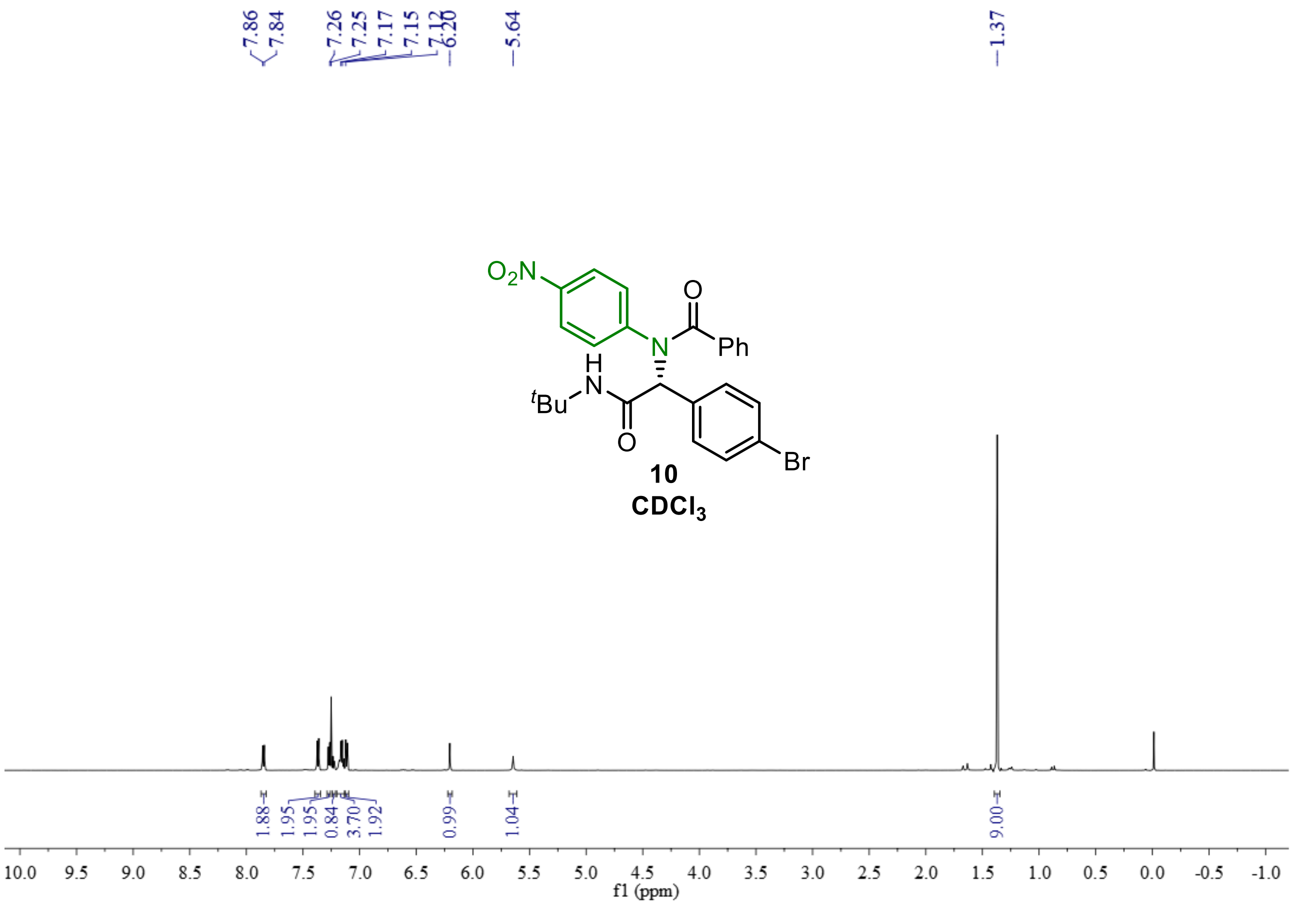

Supplementary Fig. 31. ^{1}H NMR spectrum of **10**. The sample has been recorded in 600 MHz, $CDCl_3$ at 25 °C.

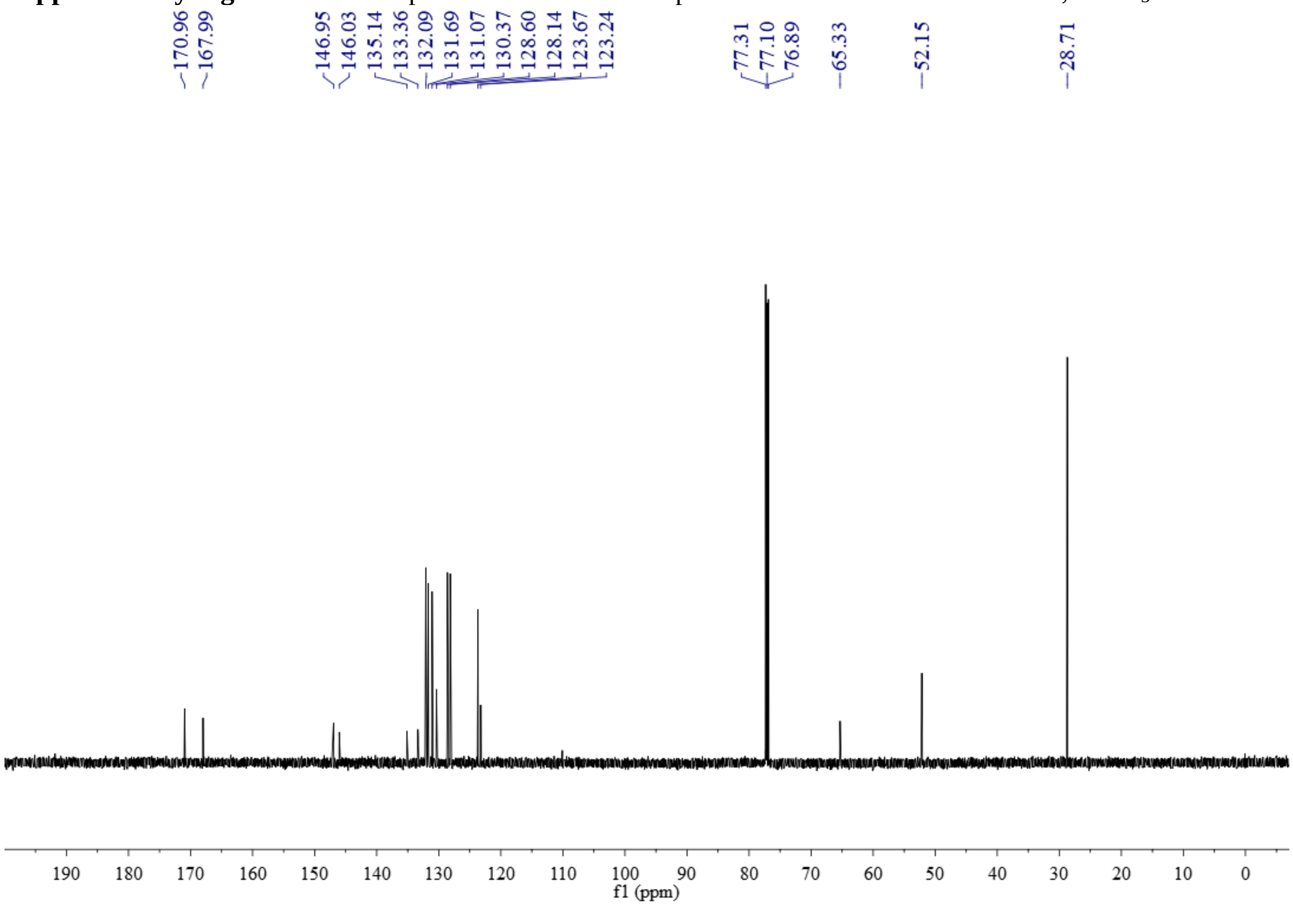

Supplementary Fig. 32.^{13}C NMR spectrum of **10**. The sample has been recorded in 151 MHz, $CDCl_3$ at 25 °C.

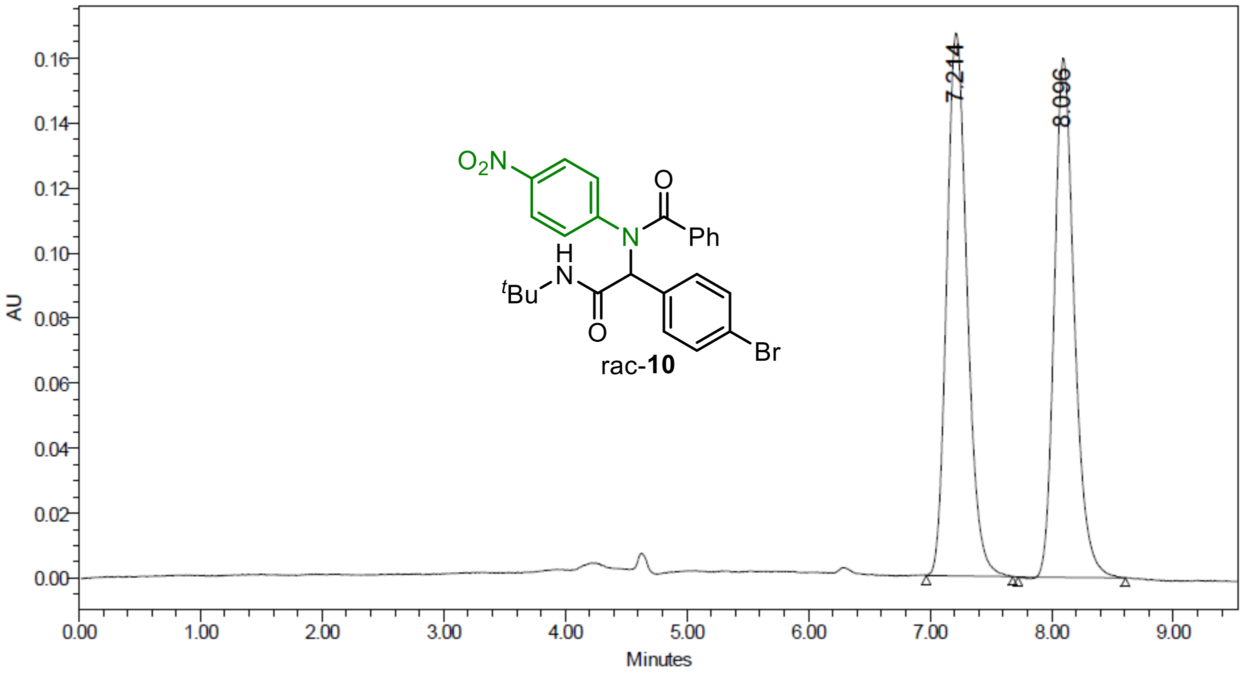

	RT (min)	Peak Type	Area (μV*sec)	% Area	Height (μV)	% Height	Integration Type	Points Across Peak	Start Time (min)	End Time (min)
1	7.214	Unknown	1948099	51.68	167015	51.10	Bb	427	6.968	7.680
2	8.096	Unknown	1821619	48.32	159798	48.90	bB	530	7.723	8.607

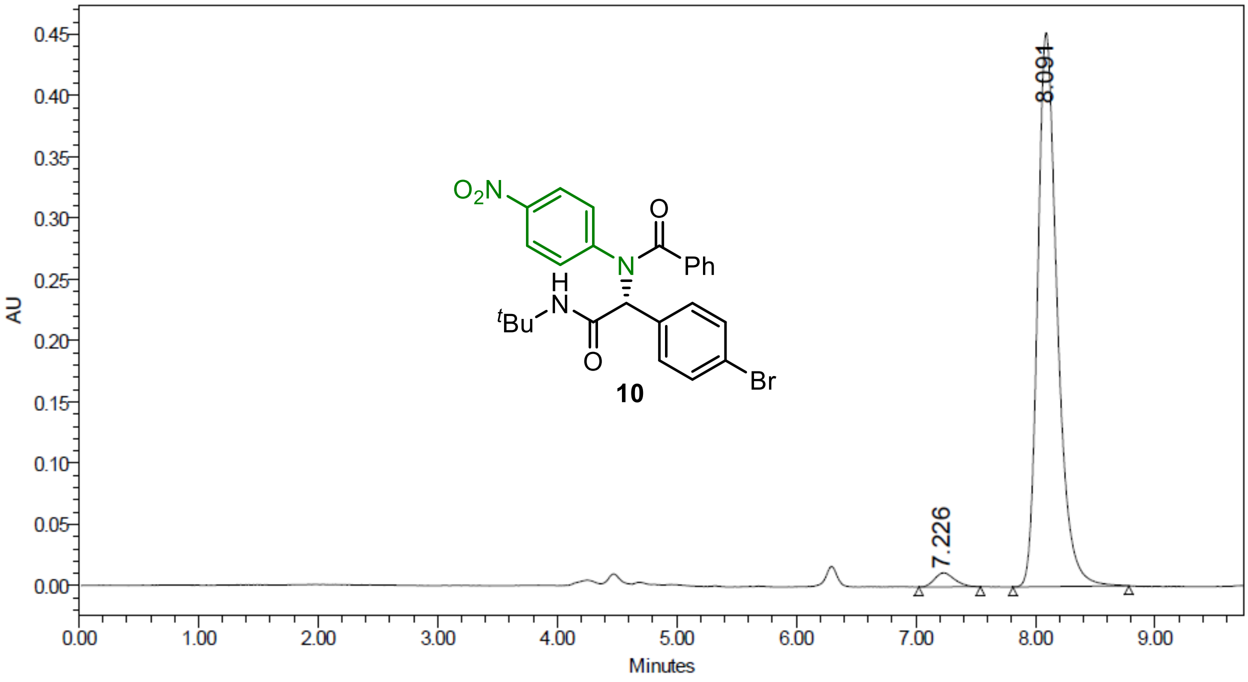

	RT (min)	Peak Type	Area (μV*sec)	% Area	Height (μV)	% Height	Integration Type	Points Across Peak	Start Time (min)	End Time (min)
1	7.226	Unknown	133549	2.52	11481	2.48	Bb	309	7.025	7.540
2	8.091	Unknown	5161046	97.48	452152	97.52	BB	582	7.812	8.782

Supplementary Fig. 33. HPLC of product **10**.

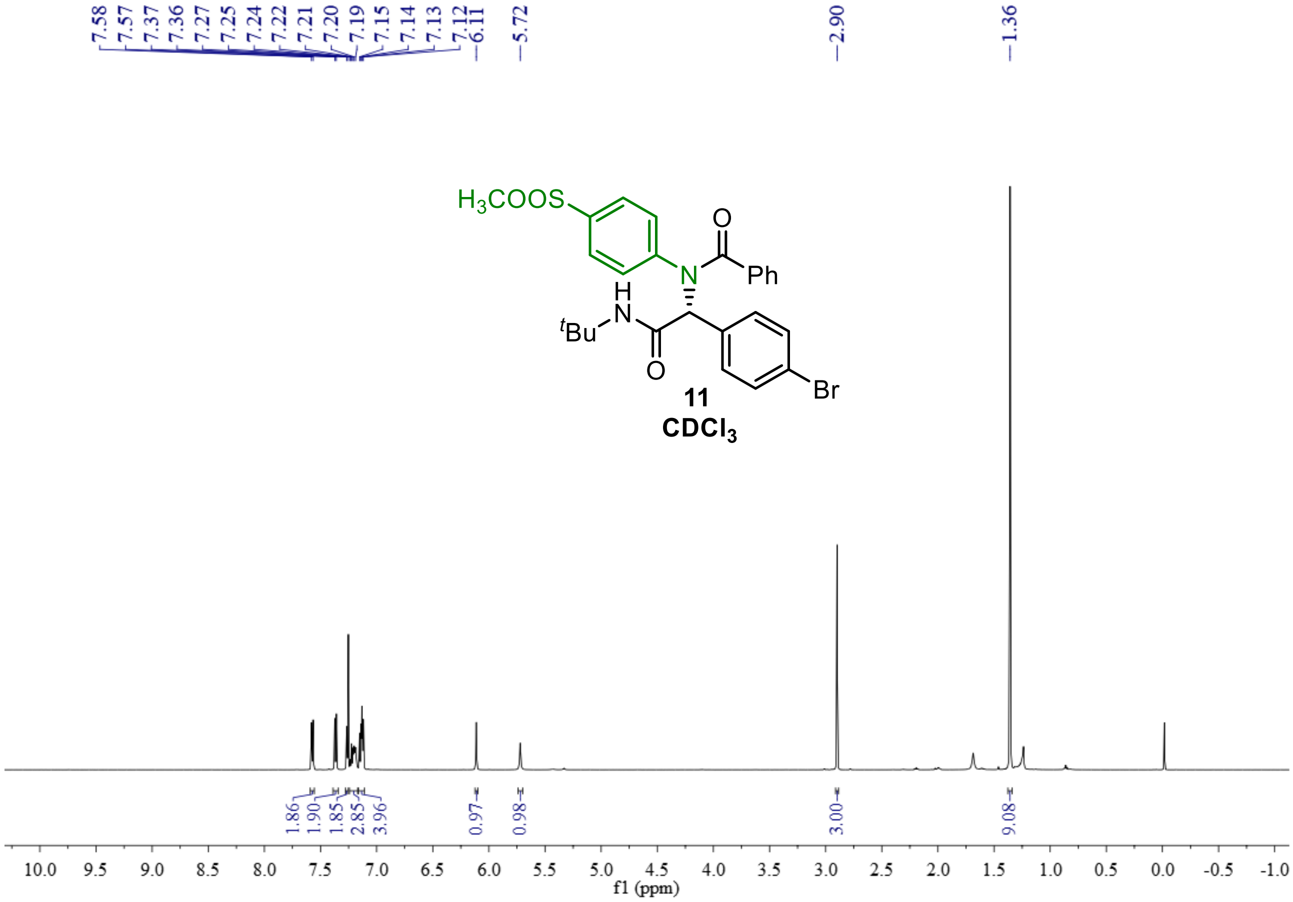

Supplementary Fig. 34. ^{1}H NMR spectrum of **11**. The sample has been recorded in 600 MHz, $CDCl_3$ at 25 °C.

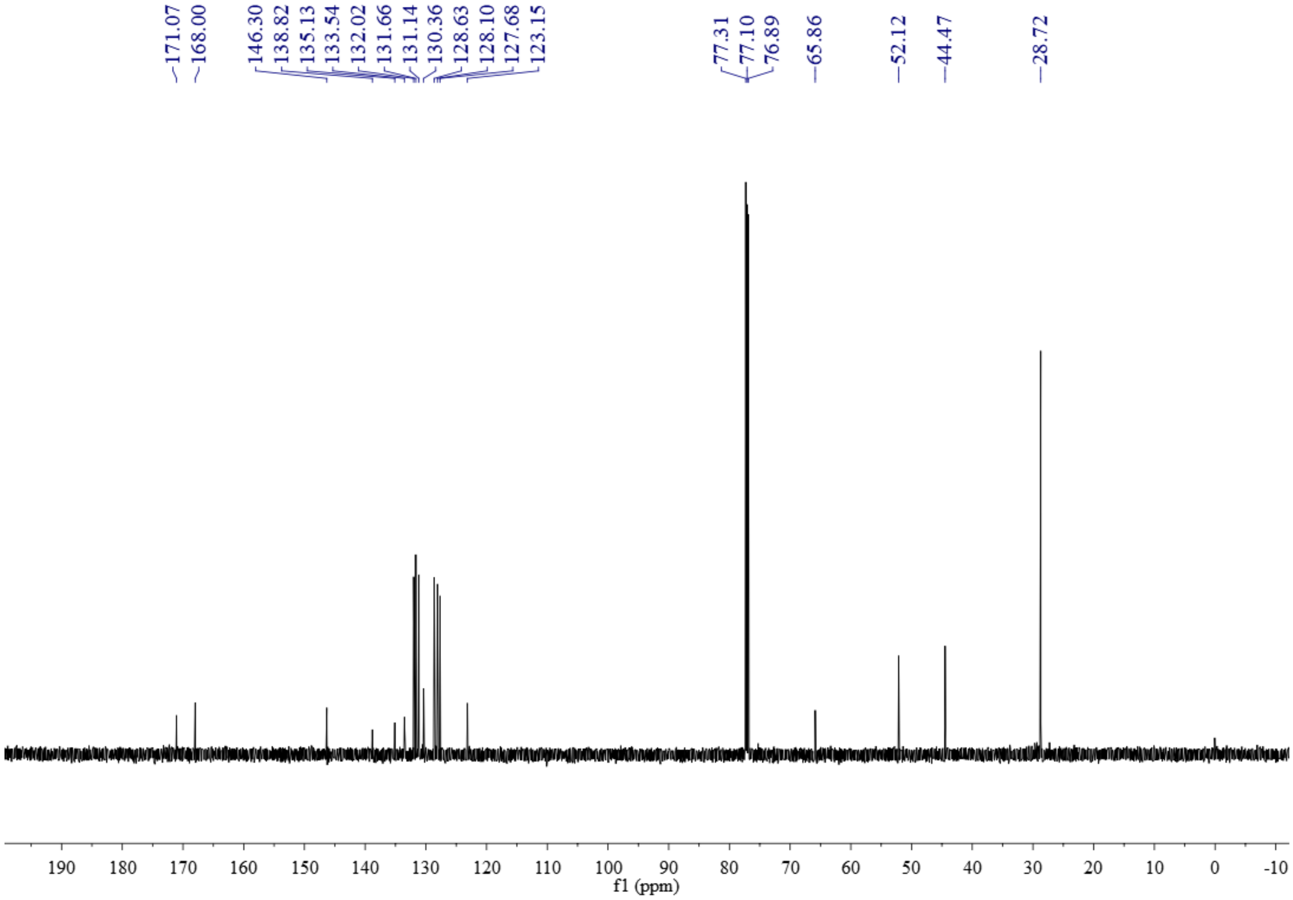

Supplementary Fig. 35.^{13}C NMR spectrum of **11**. The sample has been recorded in 151 MHz, $CDCl_3$ at 25 °C.

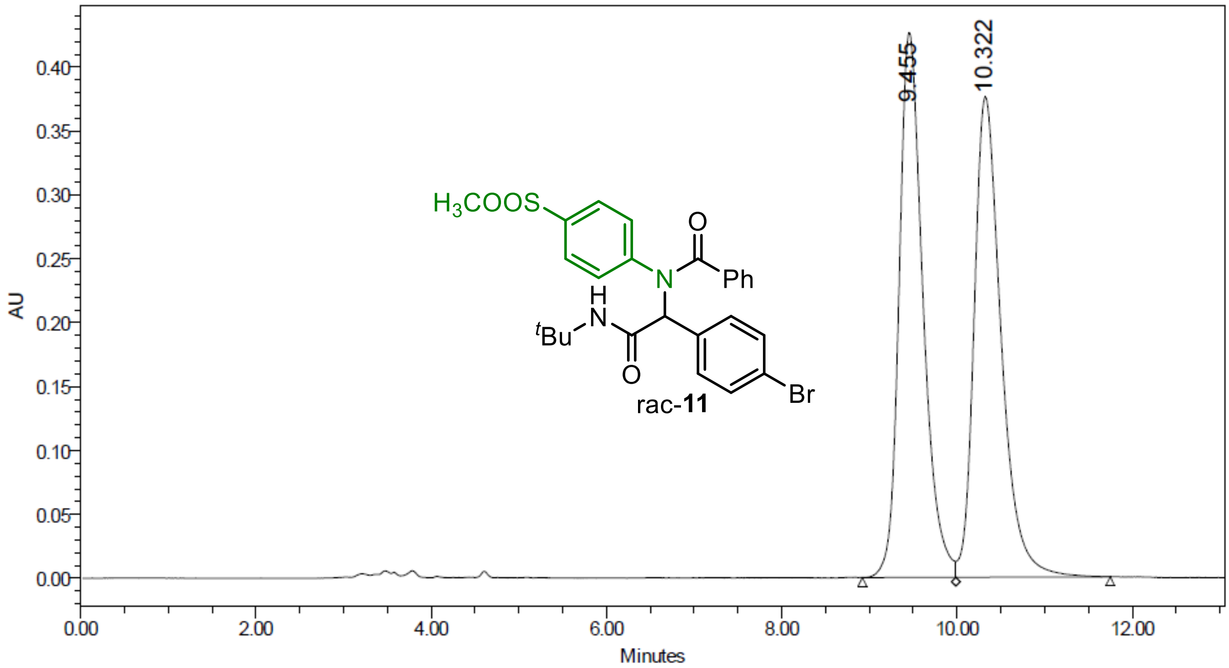

	RT (min)	Peak Type	Area (μV*sec)	% Area	Height (μV)	% Height	Integration Type	Points Across Peak	Start Time (min)	End Time (min)
1	9.455	Unknown	8079647	50.11	426221	53.15	BV	640	8.920	9.987
2	10.322	Unknown	8044253	49.89	375715	46.85	VB	1057	9.987	11.748

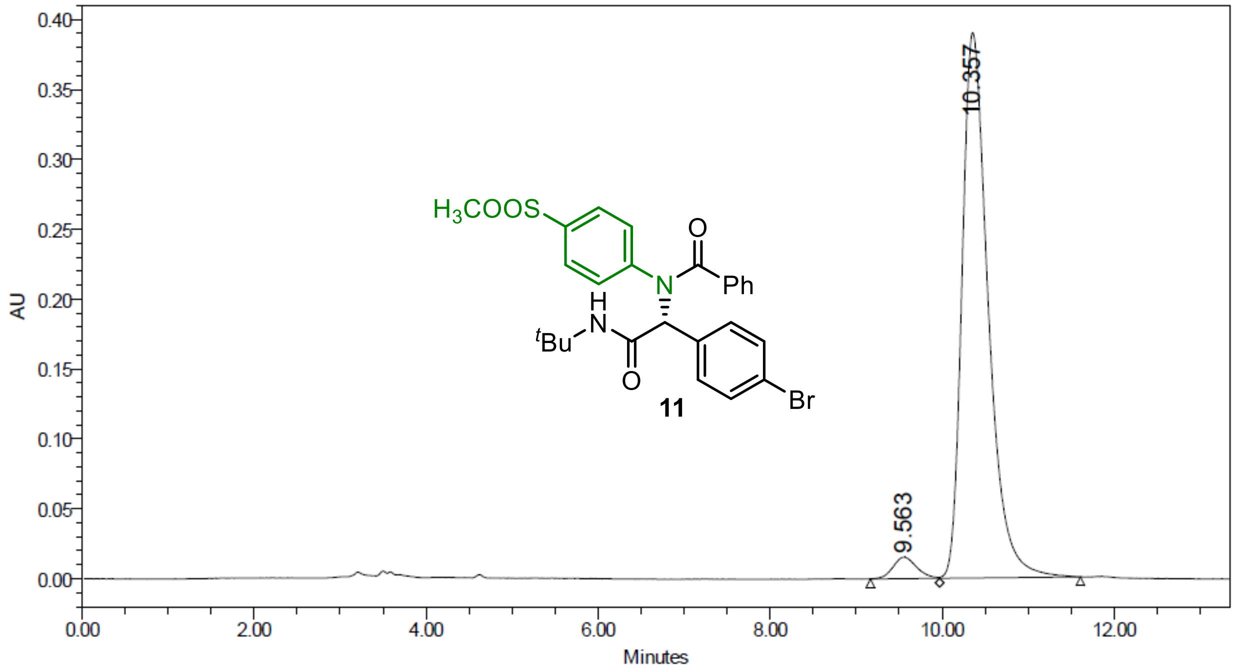

	RT (min)	Peak Type	Area (μV*sec)	% Area	Height (μV)	% Height	Integration Type	Points Across Peak	Start Time (min)	End Time (min)
1	9.563	Unknown	293979	3.44	15373	3.79	BV	483	9.167	9.972
2	10.357	Unknown	8241313	96.56	390230	96.21	VB	982	9.972	11.608

Supplementary Fig. 36. HPLC of product **11**.

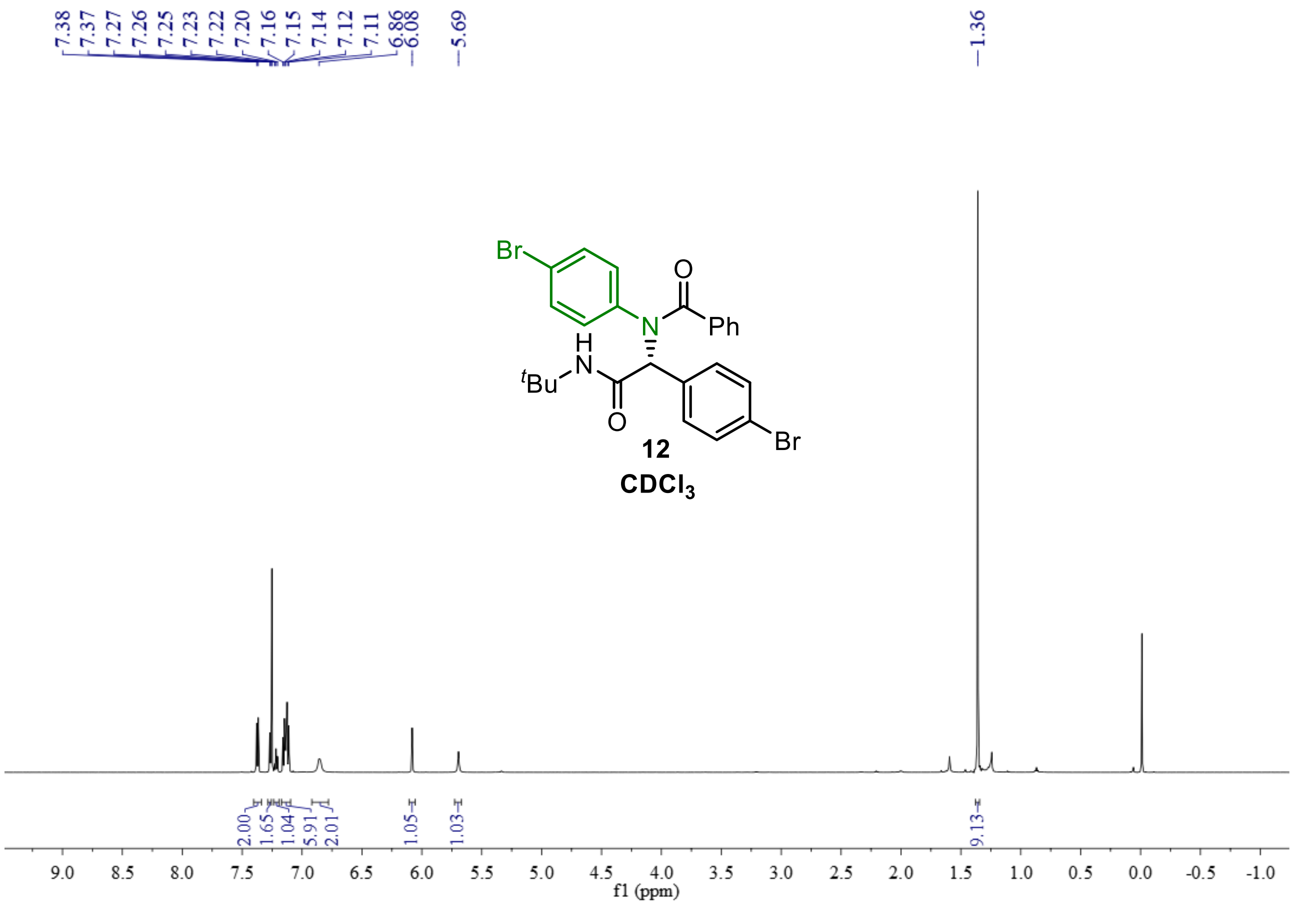

Supplementary Fig. 37. ^{1}H NMR spectrum of **12**. The sample has been recorded in 600 MHz, $CDCl_3$ at 25 °C.

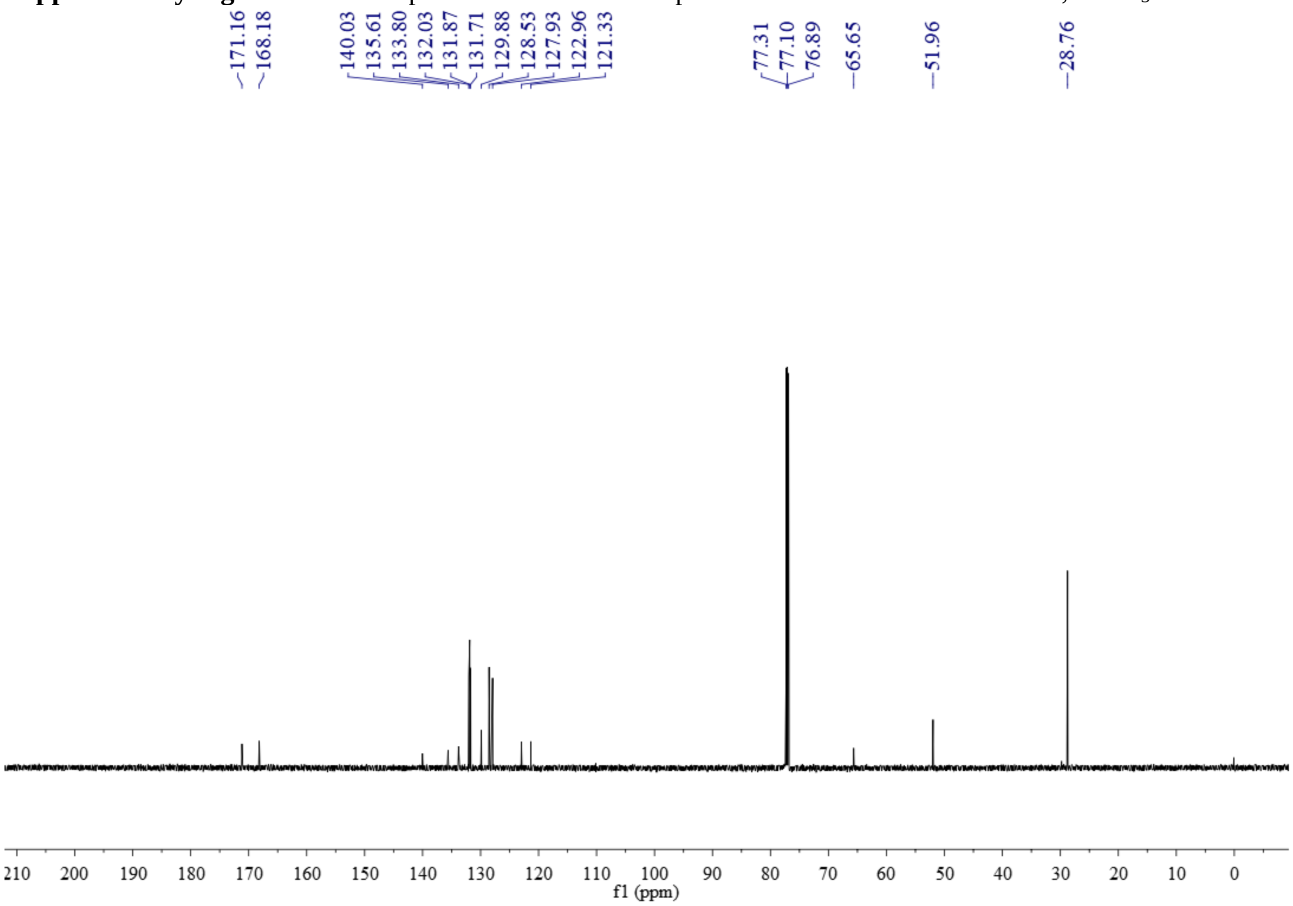

Supplementary Fig. 38.^{13}C NMR spectrum of **12**. The sample has been recorded in 151 MHz, $CDCl_3$ at 25 °C.

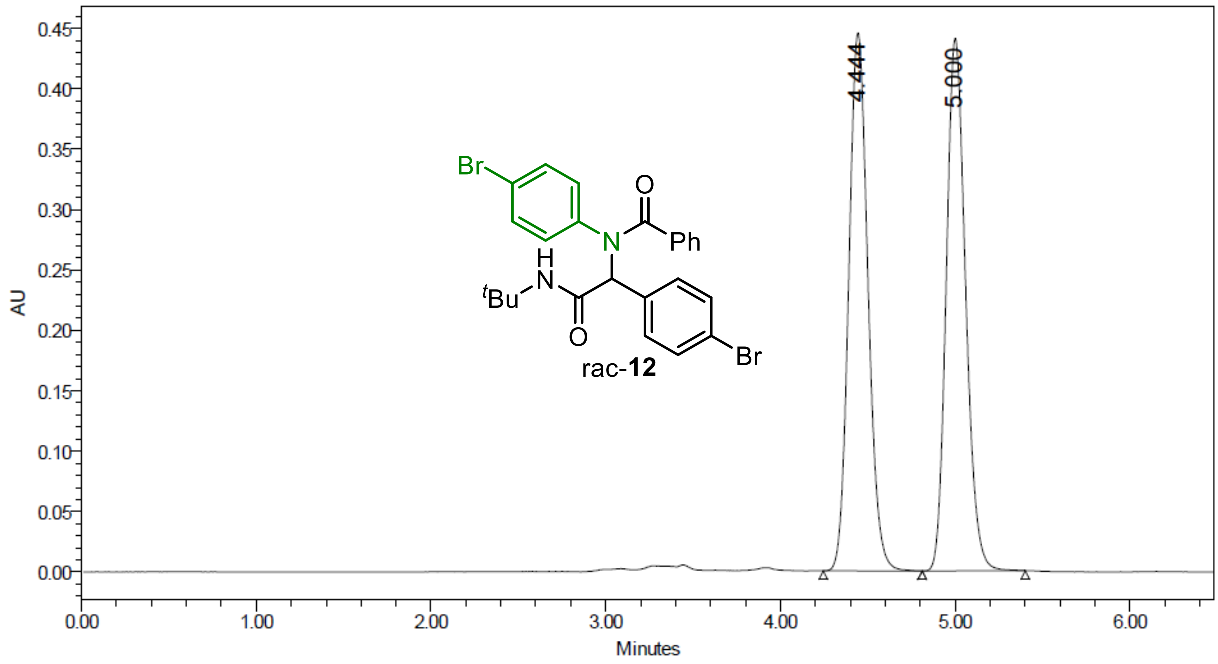

	RT (min)	Peak Type	Area (µV*sec)	% Area	Height (µV)	% Height	Integration Type	Points Across Peak	Start Time (min)	End Time (min)
1	4.444	Unknown	3402952	50.13	445405	50.26	BB	340	4.245	4.812
2	5.000	Unknown	3385504	49.87	440851	49.74	BB	354	4.812	5.402

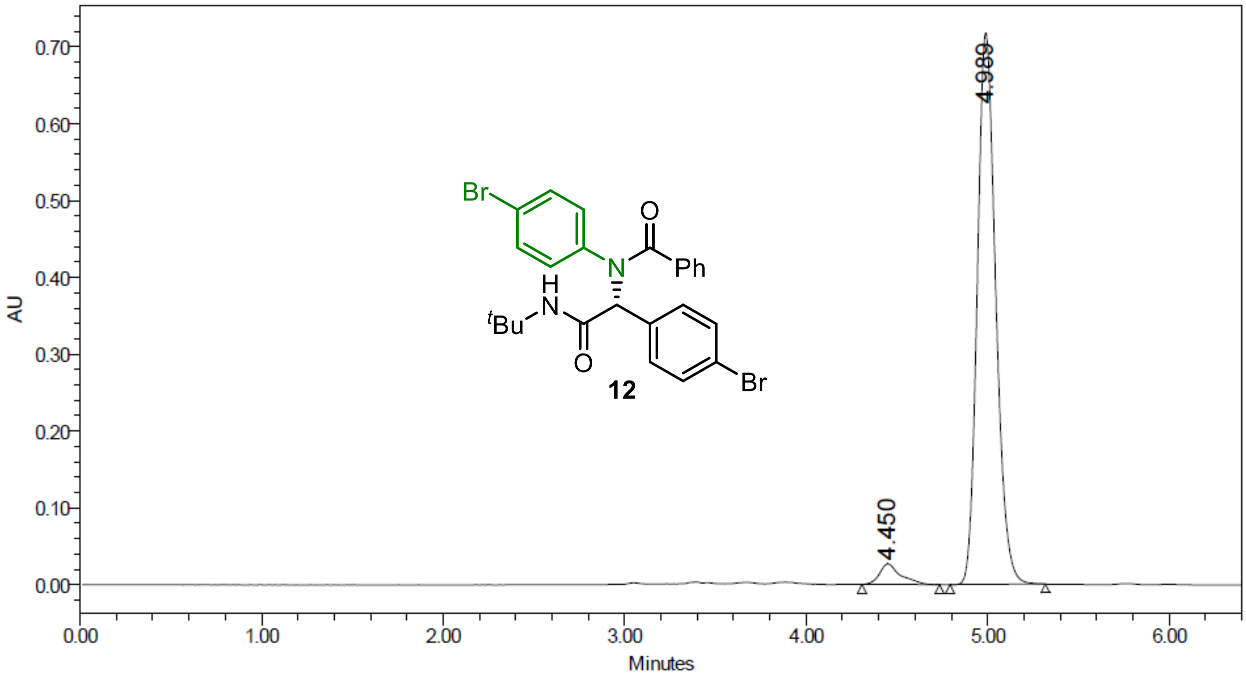

	RT (min)	Peak Type	Area (µV*sec)	% Area	Height (µV)	% Height	Integration Type	Points Across Peak	Start Time (min)	End Time (min)
1	4.450	Unknown	213491	4.05	26743	3.59	Bb	257	4.307	4.735
2	4.989	Unknown	5057161	95.95	717996	96.41	BB	315	4.792	5.317

Supplementary Fig. 39. HPLC of product **12**.

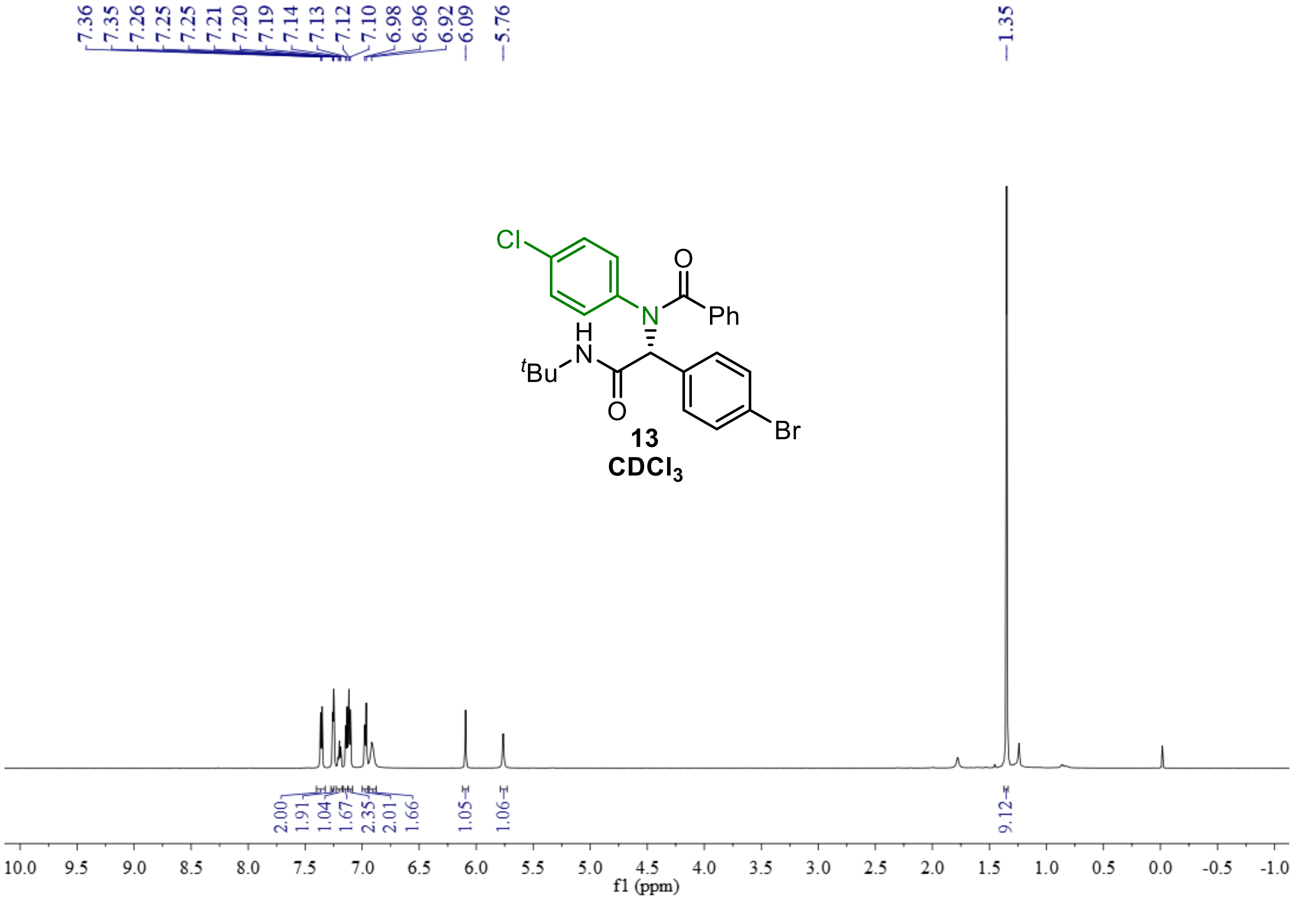

Supplementary Fig. 40. ^{1}H NMR spectrum of **13**. The sample has been recorded in 600 MHz, $CDCl_3$ at 25 °C.

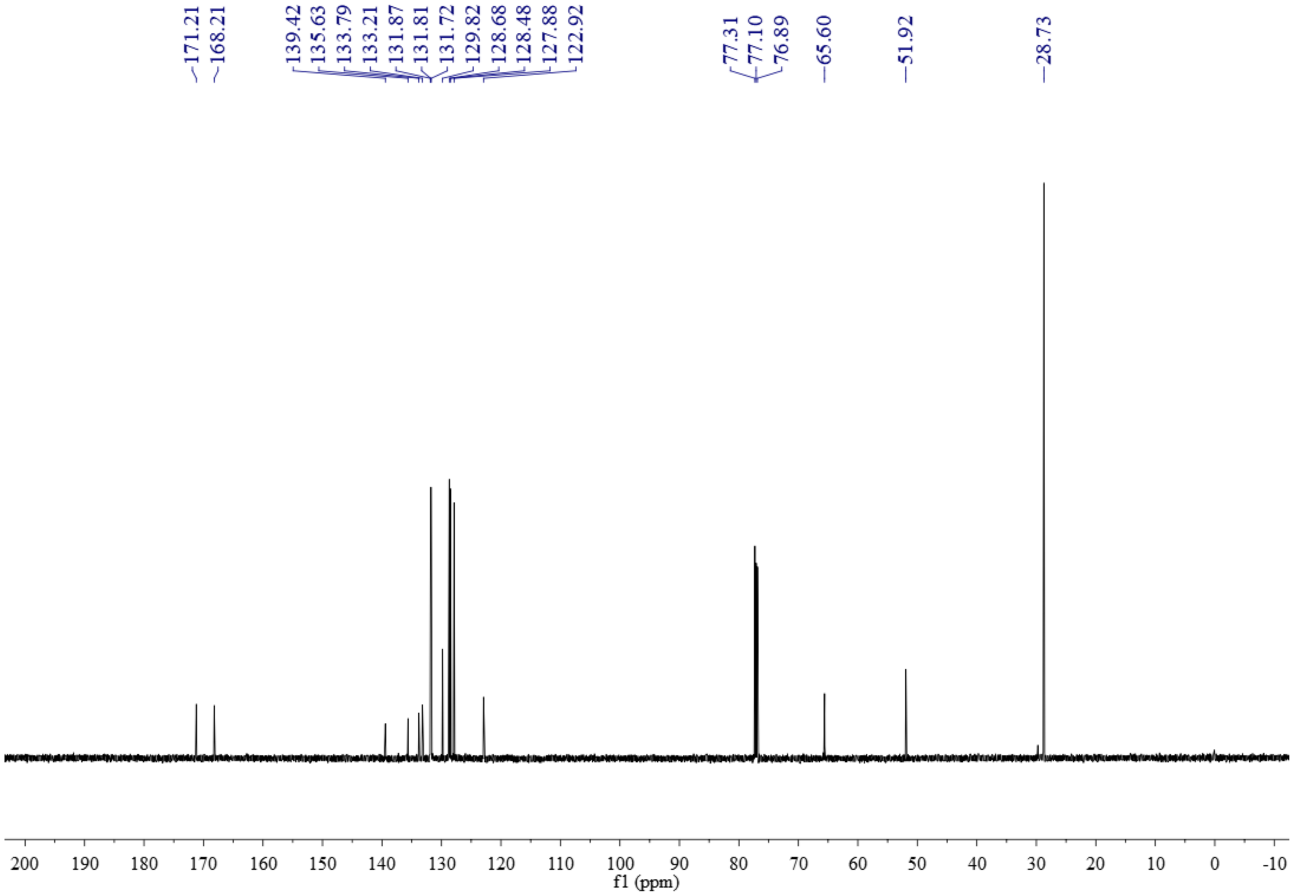

Supplementary Fig. 41.^{13}C NMR spectrum of **13**. The sample has been recorded in 151 MHz, $CDCl_3$ at 25 °C.

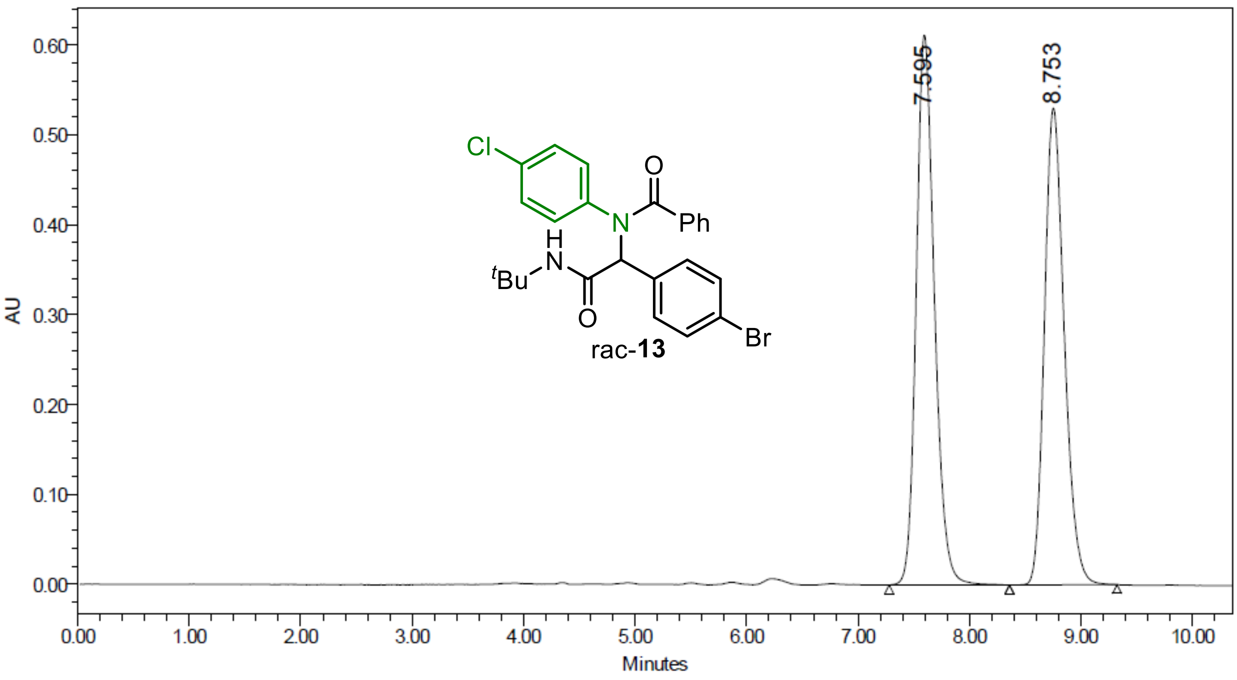

	RT (min)	Peak Type	Area (μV*sec)	% Area	Height (μV)	% Height	Integration Type	Points Across Peak	Start Time (min)	End Time (min)
1	7.595	Unknown	6934050	51.32	611855	53.55	BB	647	7.280	8.358
2	8.753	Unknown	6576198	48.68	530698	46.45	BB	579	8.358	9.323

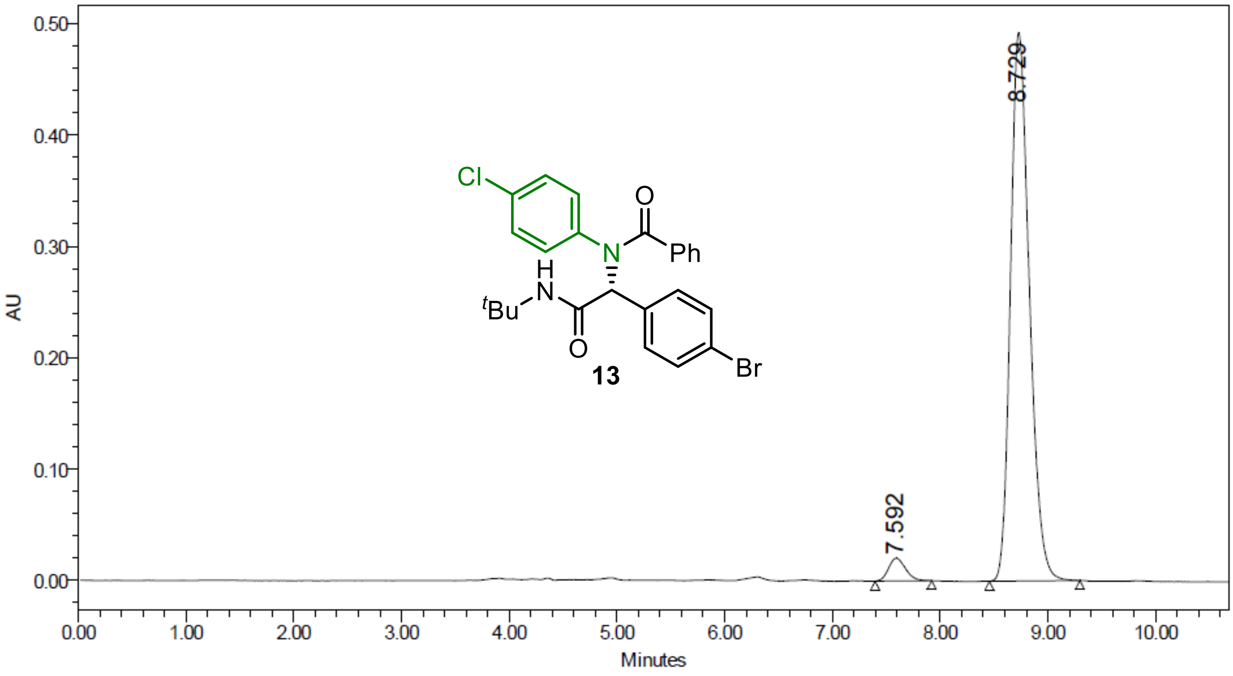

	RT (min)	Peak Type	Area (μV*sec)	% Area	Height (μV)	% Height	Integration Type	Points Across Peak	Start Time (min)	End Time (min)
1	7.592	Unknown	223227	3.58	20386	3.97	BB	315	7.397	7.922
2	8.729	Unknown	6004712	96.42	492627	96.03	BB	504	8.457	9.297

Supplementary Fig. 42. HPLC of product **13**.

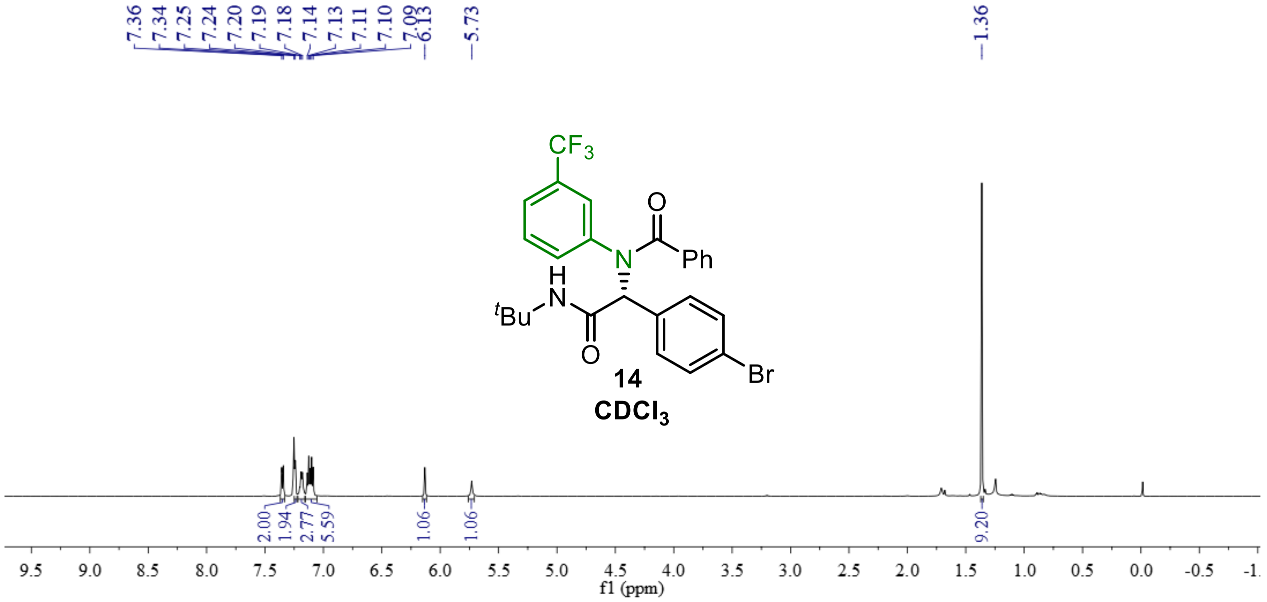

Supplementary Fig. 43. ^{1}H NMR spectrum of **14**. The sample has been recorded in 600 MHz, $CDCl_3$ at 25 °C.

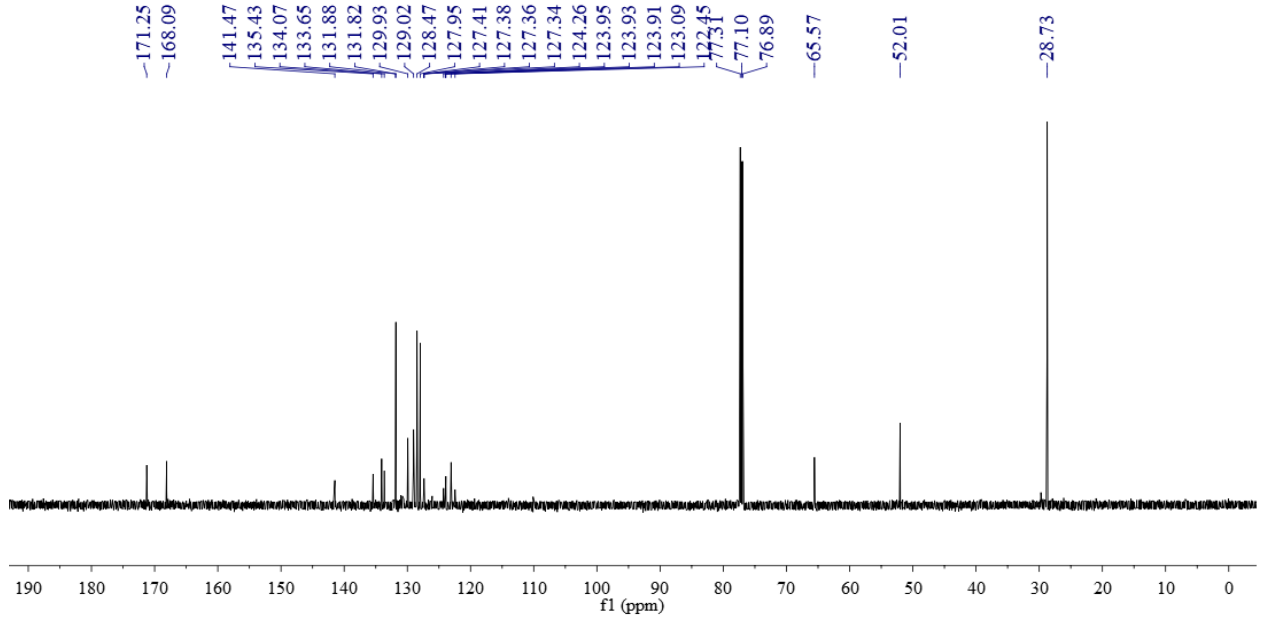

Supplementary Fig. 44.^{13}C NMR spectrum of **14**. The sample has been recorded in 151 MHz, $CDCl_3$ at 25 °C.

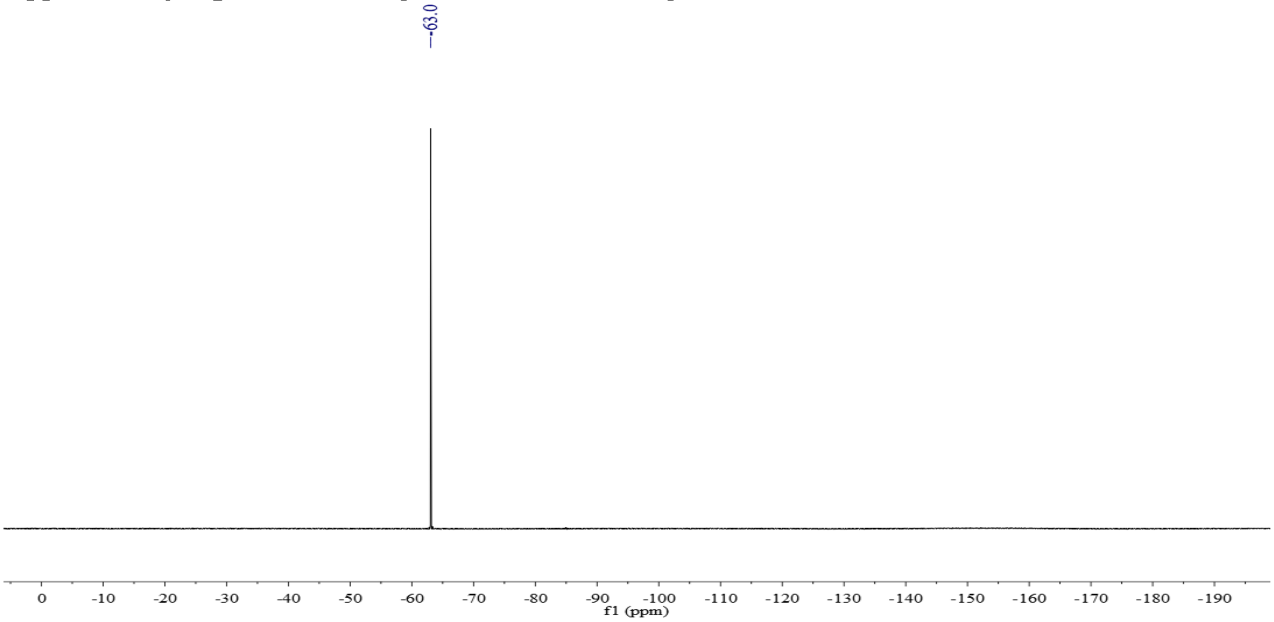

Supplementary Fig. 45. ^{31}F NMR spectrum of **14**. The sample has been recorded in 564 MHz, $CDCl_3$ at 25 °C.

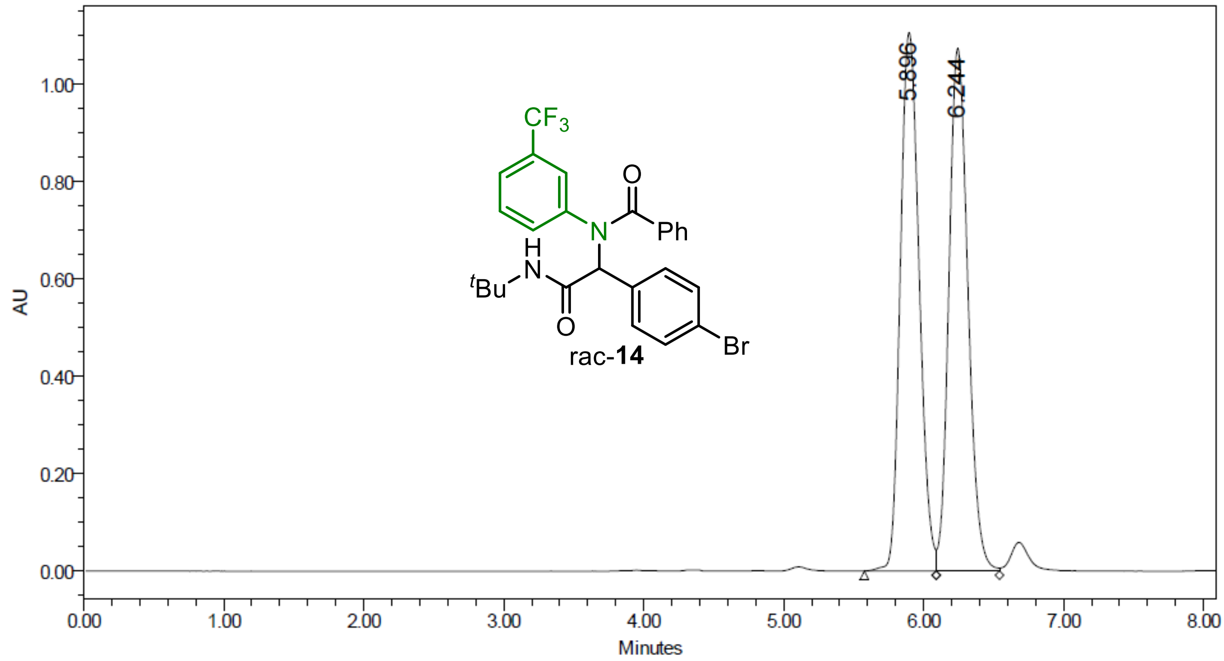

	RT (min)	Peak Type	Area (µV*sec)	% Area	Height (µV)	% Height	Integration Type	Points Across Peak	Start Time (min)	End Time (min)
1	5.896	Unknown	10316725	50.47	1105857	50.76	BV	308	5.577	6.090
2	6.244	Unknown	10125524	49.53	1072744	49.24	VV	271	6.090	6.542

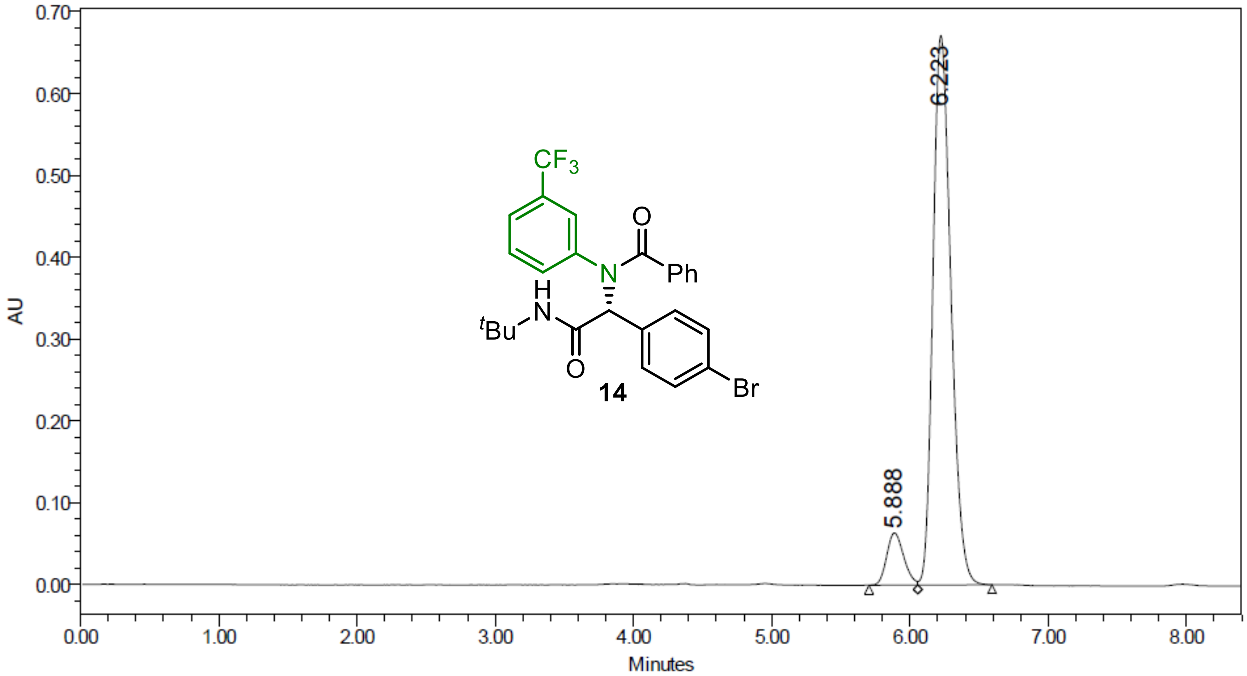

	RT (min)	Peak Type	Area (µV*sec)	% Area	Height (µV)	% Height	Integration Type	Points Across Peak	Start Time (min)	End Time (min)
1	5.888	Unknown	545727	8.26	63696	8.66	BV	212	5.703	6.057
2	6.223	Unknown	6062505	91.74	671442	91.34	VB	322	6.057	6.593

Supplementary Fig. 46. HPLC of product **14**.

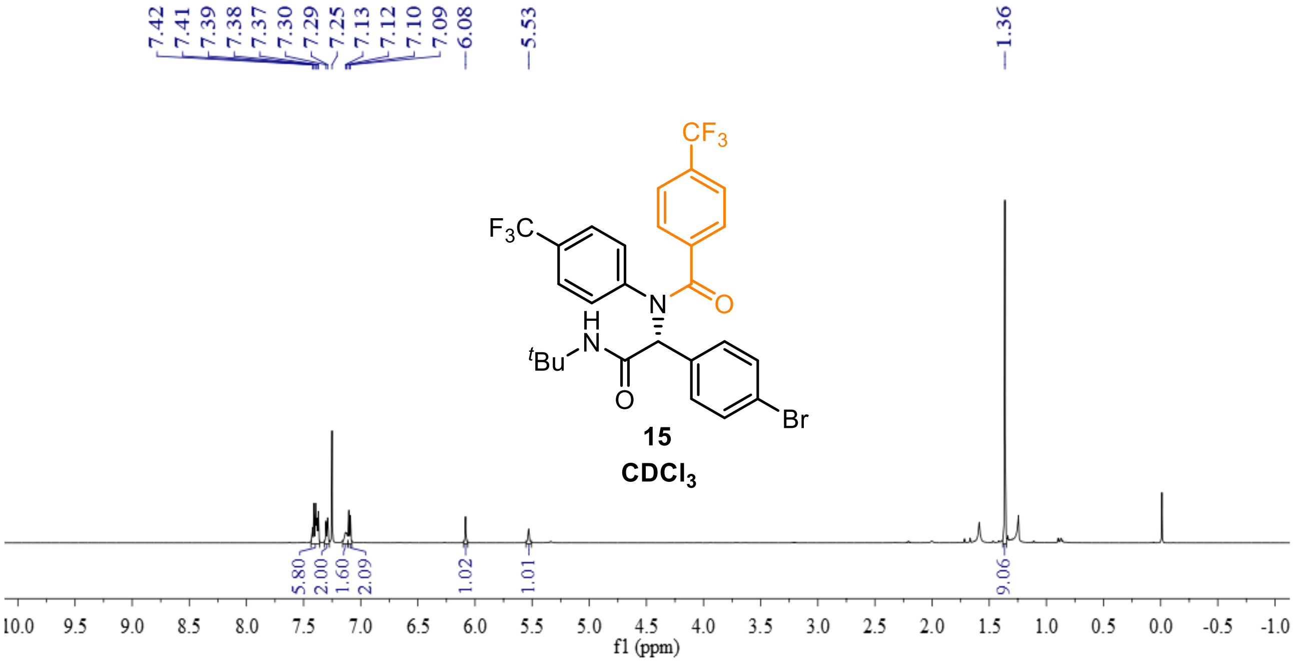

Supplementary Fig. 47. ^{1}H NMR spectrum of **15**. The sample has been recorded in 600 MHz, $CDCl_3$ at 25 °C.

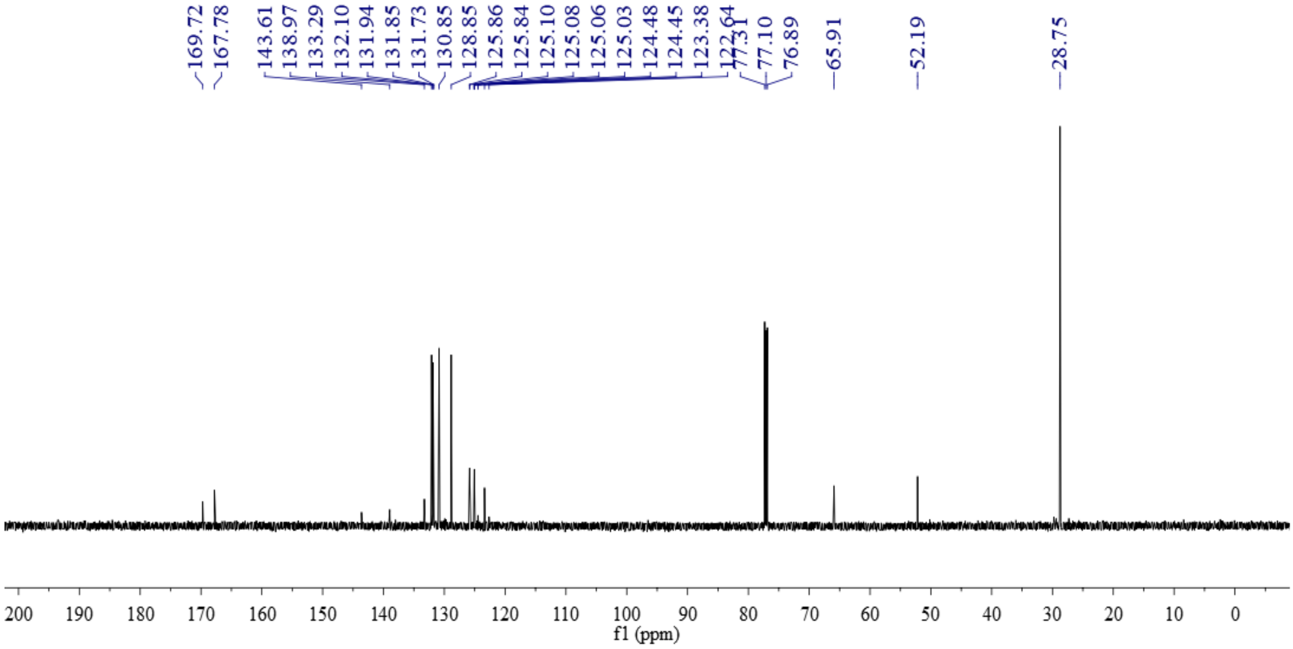

Supplementary Fig. 48.^{13}C NMR spectrum of **15**. The sample has been recorded in 151 MHz, $CDCl_3$ at 25 °C.

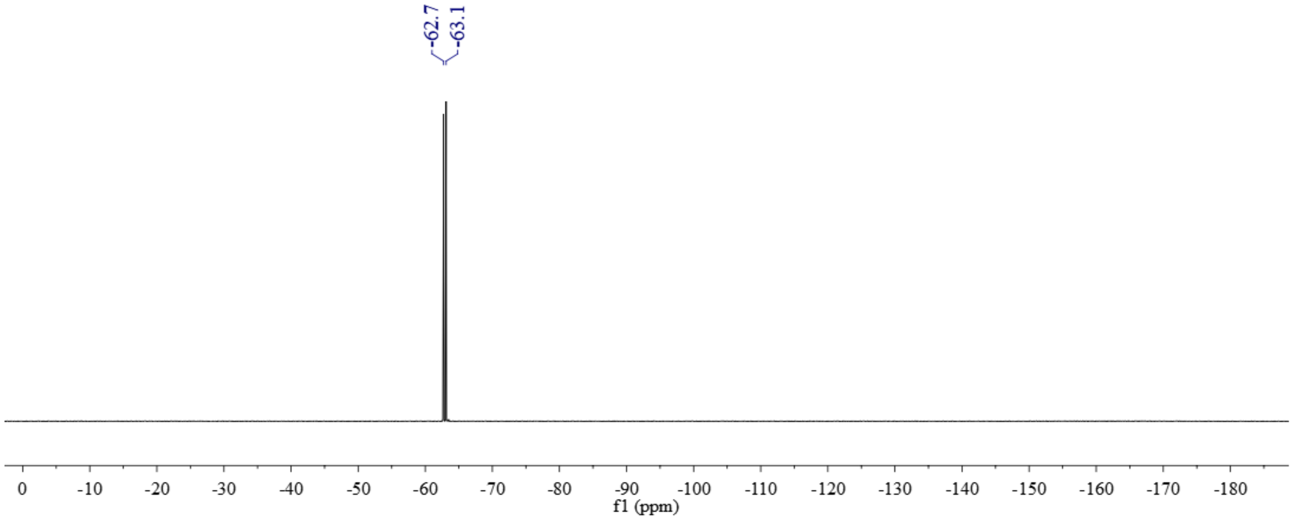

Supplementary Fig. 49. ^{31}F NMR spectrum of **15**. The sample has been recorded in 564 MHz, $CDCl_3$ at 25 °C.

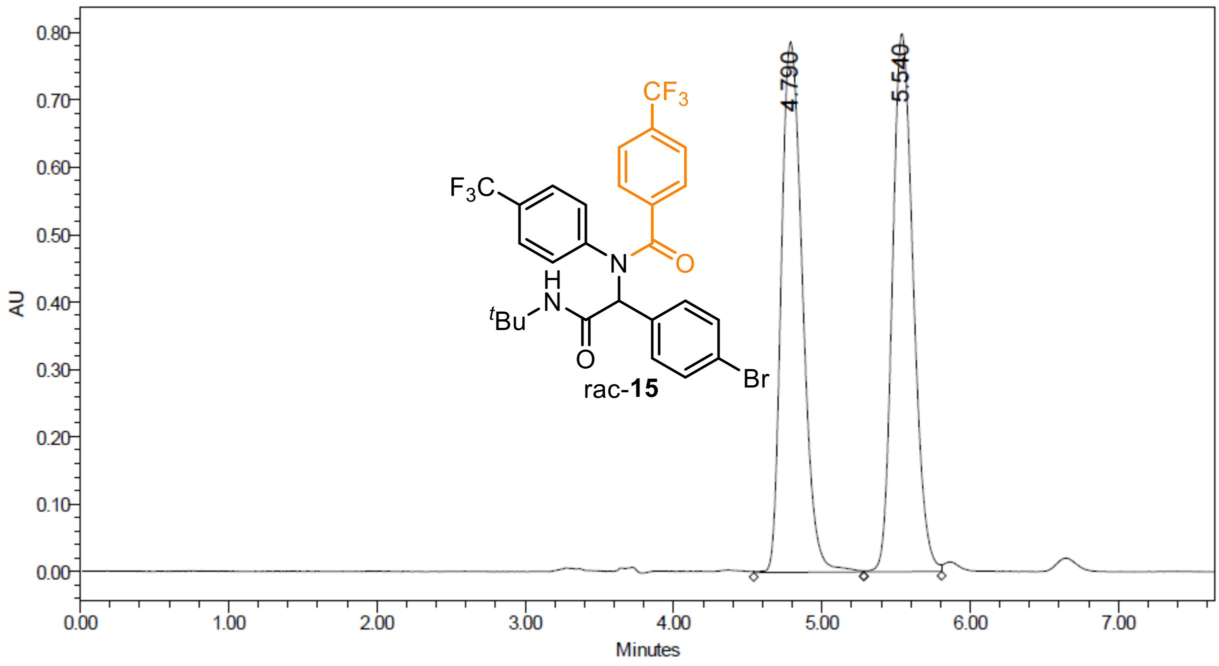

	RT (min)	Peak Type	Area (µV*sec)	% Area	Height (µV)	% Height	Integration Type	Points Across Peak	Start Time (min)	End Time (min)
1	4.790	Unknown	7821281	50.12	787338	49.63	VV	445	4.542	5.283
2	5.540	Unknown	7784219	49.88	798998	50.37	VV	315	5.283	5.808

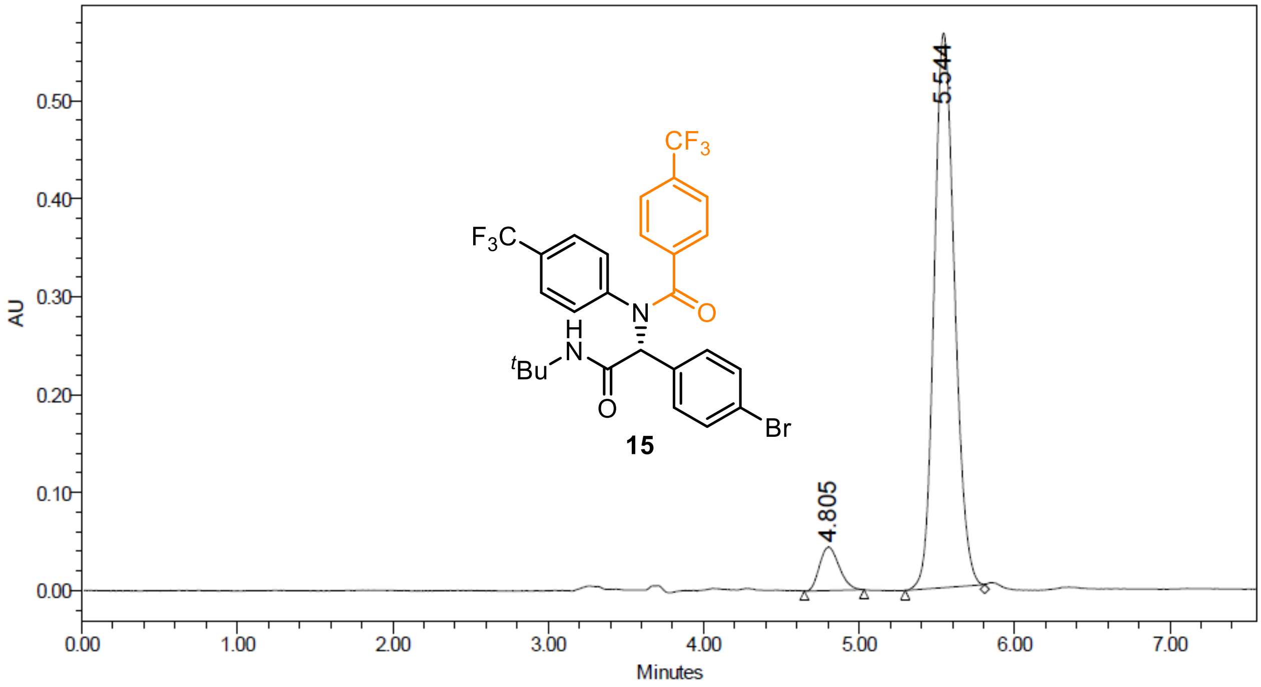

	RT (min)	Peak Type	Area (µV*sec)	% Area	Height (µV)	% Height	Integration Type	Points Across Peak	Start Time (min)	End Time (min)
1	4.805	Unknown	389188	6.99	44387	7.27	Bb	230	4.648	5.032
2	5.544	Unknown	5177916	93.01	566438	92.73	BV	307	5.297	5.808

Supplementary Fig. 50. HPLC of product **15**.

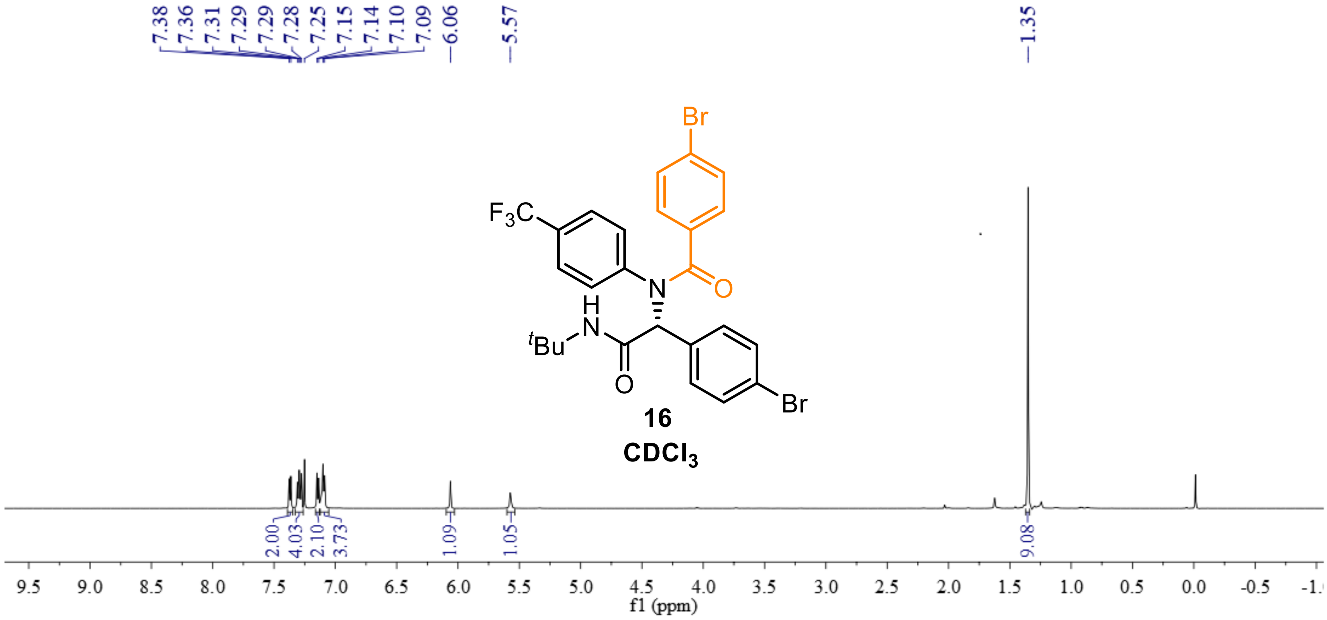

Supplementary Fig. 51. ^{1}H NMR spectrum of **16**. The sample has been recorded in 600 MHz, $CDCl_3$ at 25 °C.

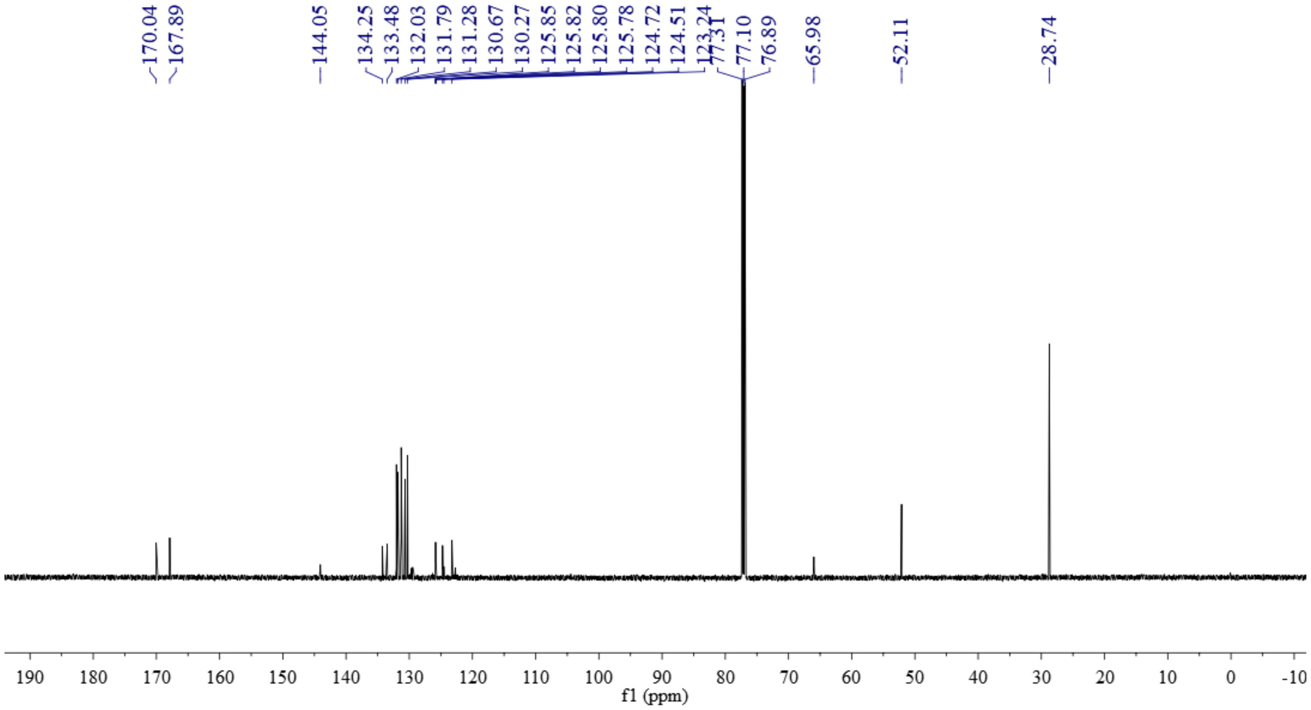

Supplementary Fig. 52.^{13}C NMR spectrum of **16**. The sample has been recorded in 151 MHz, $CDCl_3$ at 25 °C.

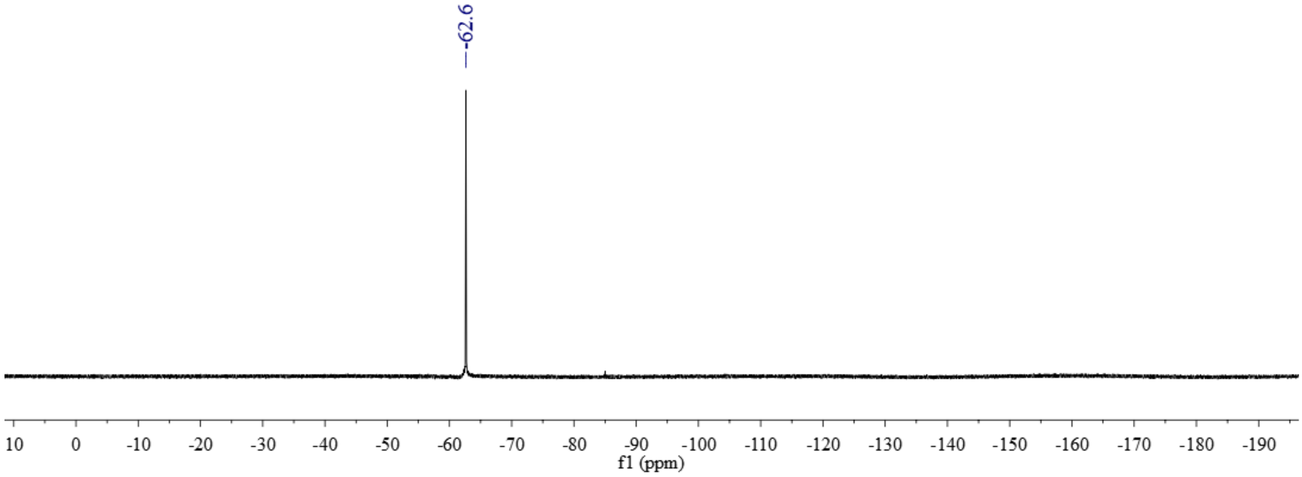

Supplementary Fig. 53. [31]F NMR spectrum of **16**. The sample has been recorded in 564 MHz, $CDCl_3$ at 25 °C.

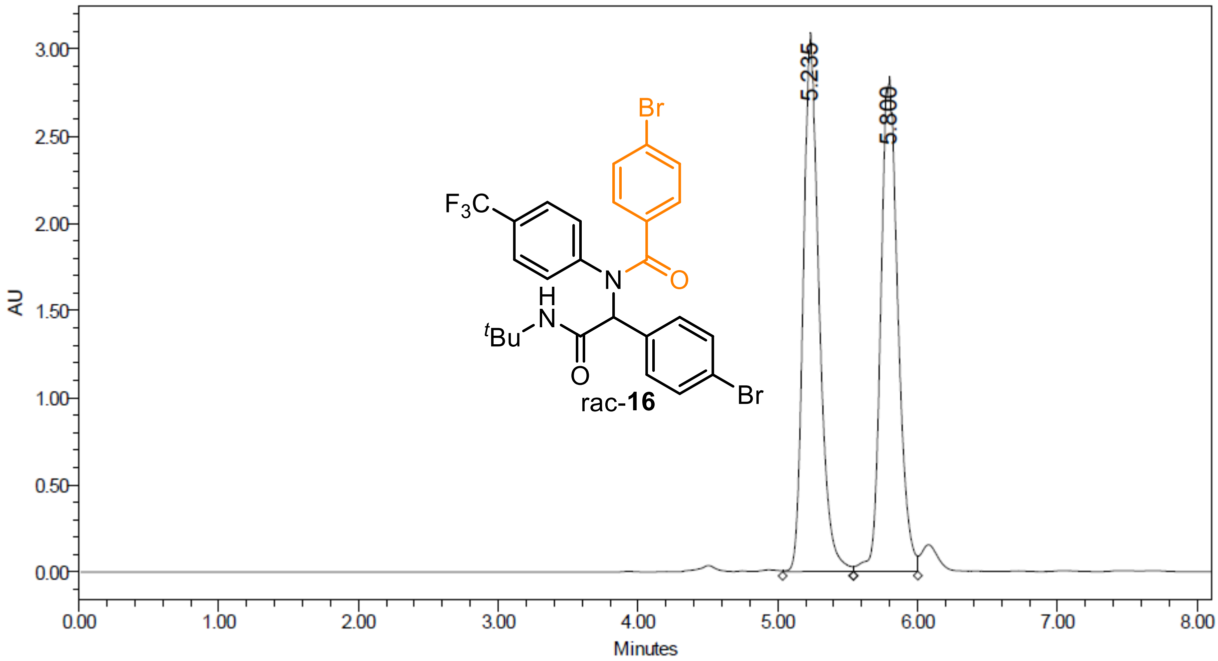

	RT (min)	Peak Type	Area (µV*sec)	% Area	Height (µV)	% Height	Integration Type	Points Across Peak	Start Time (min)	End Time (min)
1	5.235	Unknown	24172253	49.88	3094182	52.18	VV	303	5.035	5.540
2	5.800	Unknown	24290119	50.12	2835815	47.82	VV	277	5.540	6.002

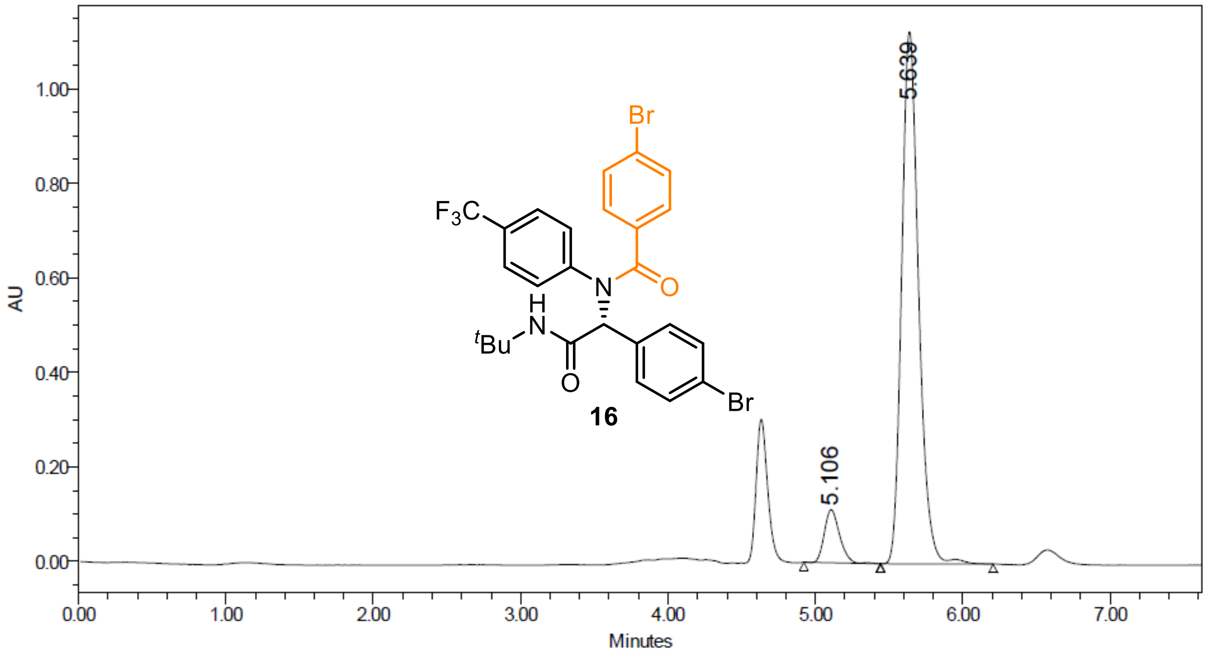

	RT (min)	Peak Type	Area (µV*sec)	% Area	Height (µV)	% Height	Integration Type	Points Across Peak	Start Time (min)	End Time (min)
1	5.106	Unknown	811871	8.26	112629	9.10	bB	310	4.922	5.438
2	5.639	Unknown	9011379	91.74	1125717	90.90	Bb	457	5.443	6.205

Supplementary Fig. 54. HPLC of product **16**.

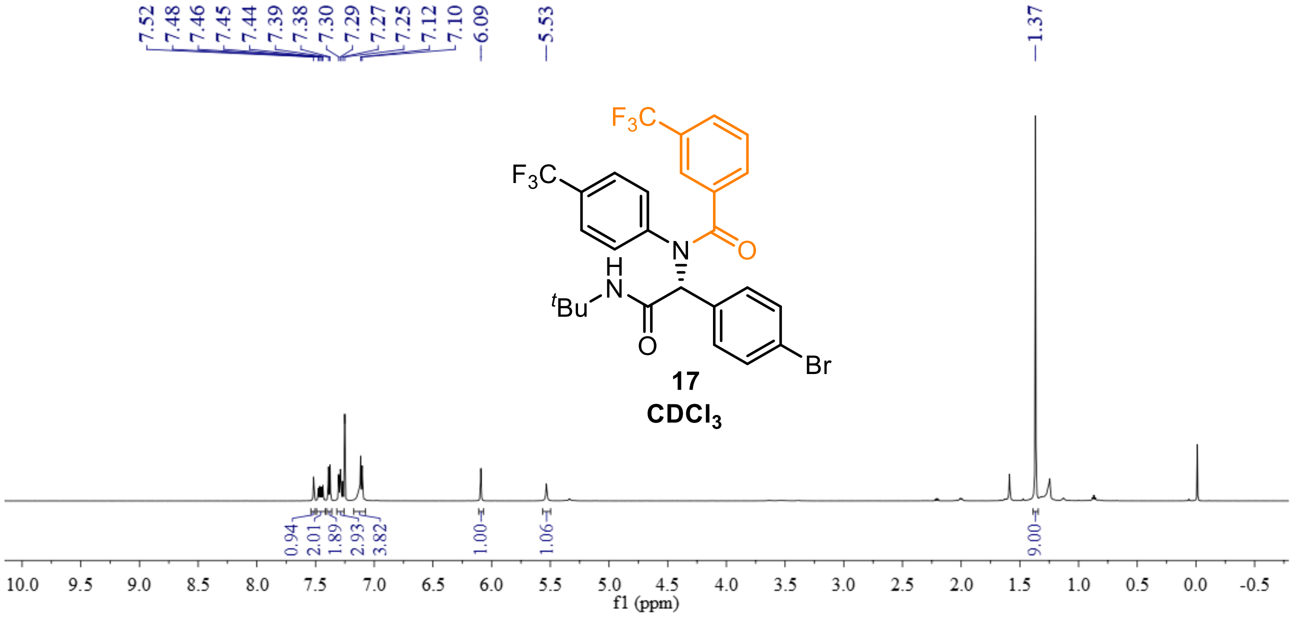

Supplementary Fig. 55. ^{1}H NMR spectrum of **17**. The sample has been recorded in 600 MHz, $CDCl_3$ at 25 °C.

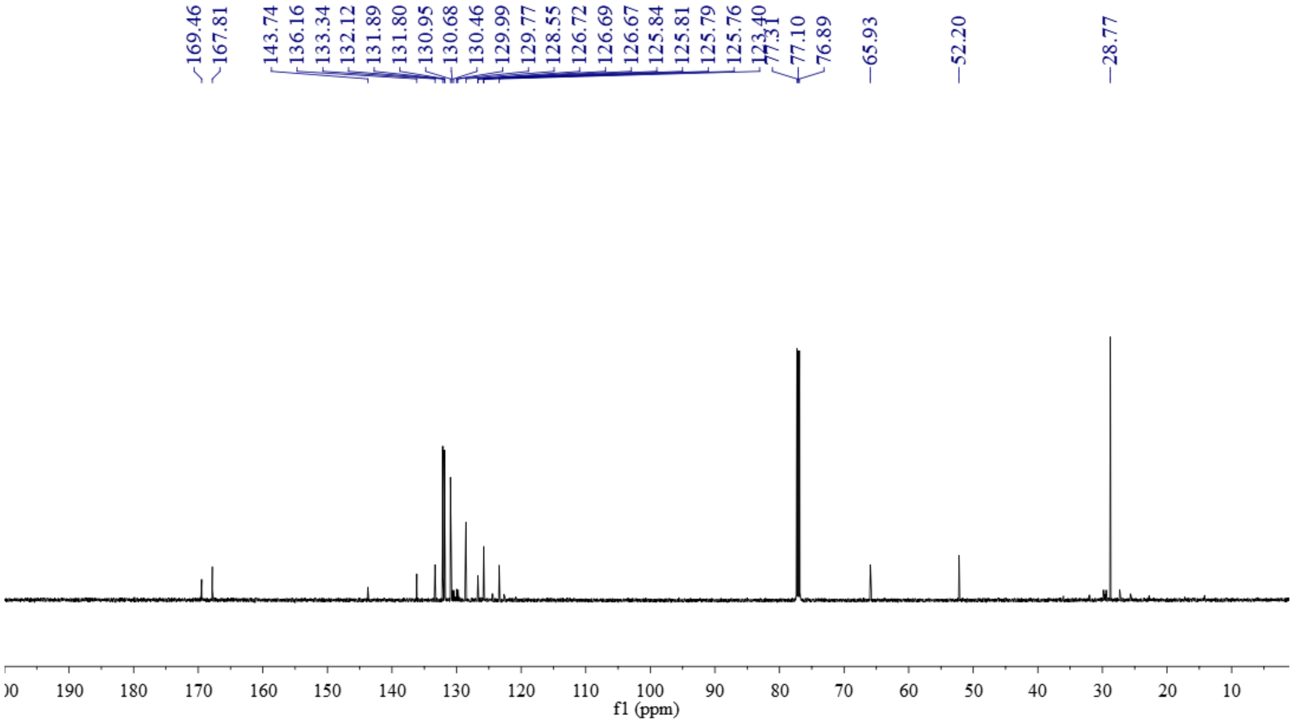

Supplementary Fig. 56.^{13}C NMR spectrum of **17**. The sample has been recorded in 151 MHz, $CDCl_3$ at 25 °C.

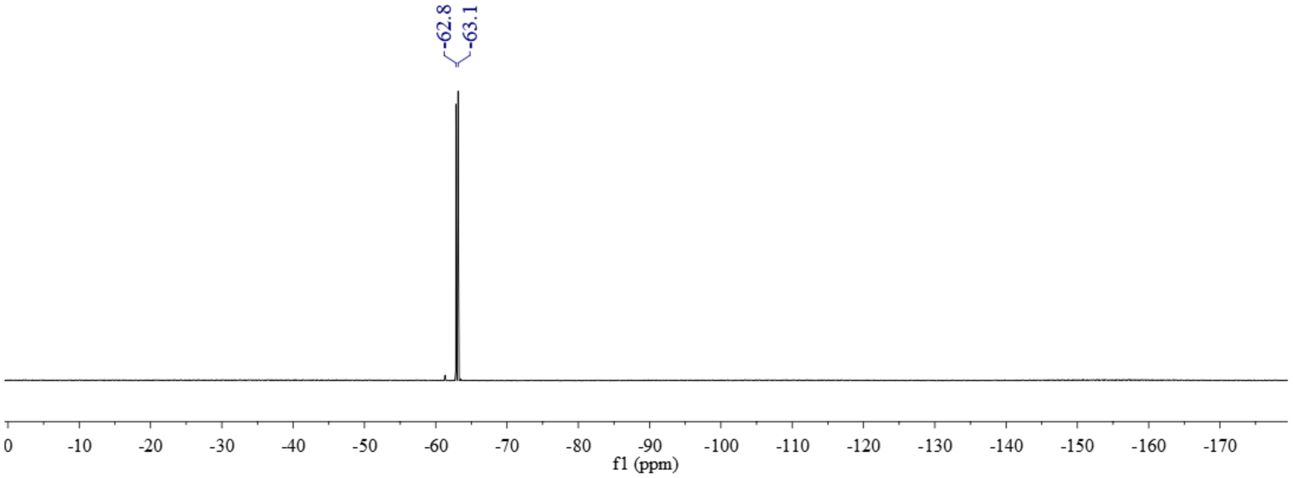

Supplementary Fig. 57. ^{31}F NMR spectrum of **17**. The sample has been recorded in 564 MHz, $CDCl_3$ at 25 °C.

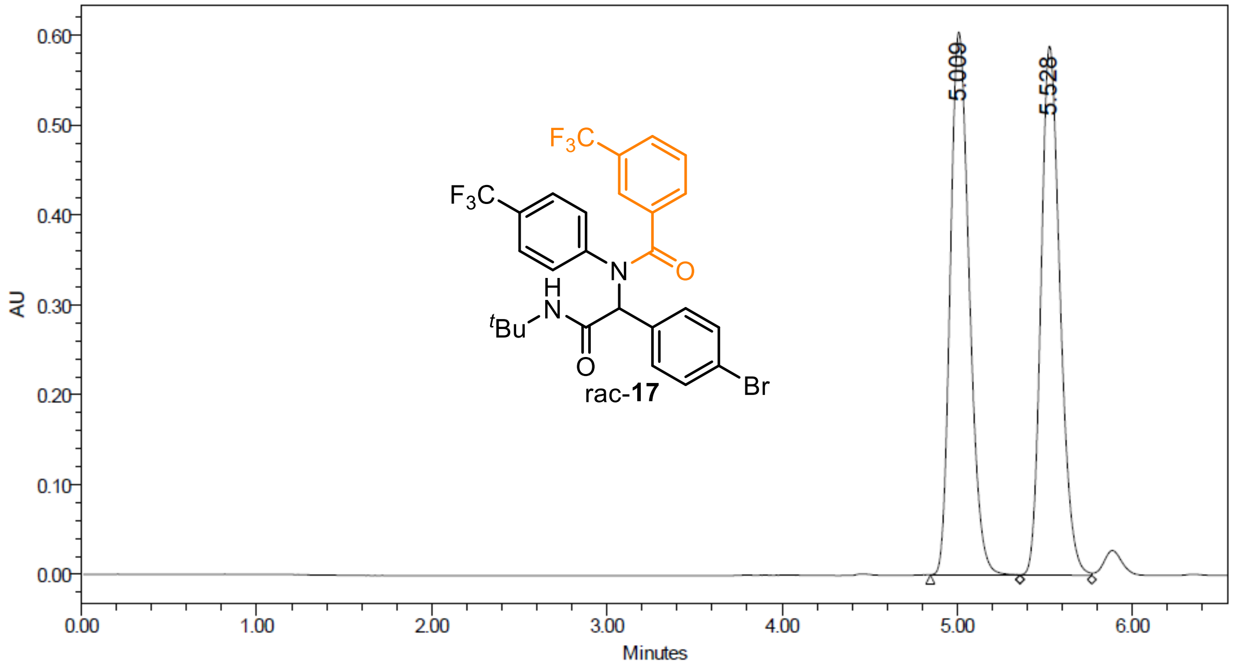

	RT (min)	Peak Type	Area (μV*sec)	% Area	Height (μV)	% Height	Integration Type	Points Across Peak	Start Time (min)	End Time (min)
1	5.009	Unknown	4549393	50.49	604351	50.64	BV	307	4.847	5.358
2	5.528	Unknown	4461293	49.51	589108	49.36	VV	246	5.358	5.768

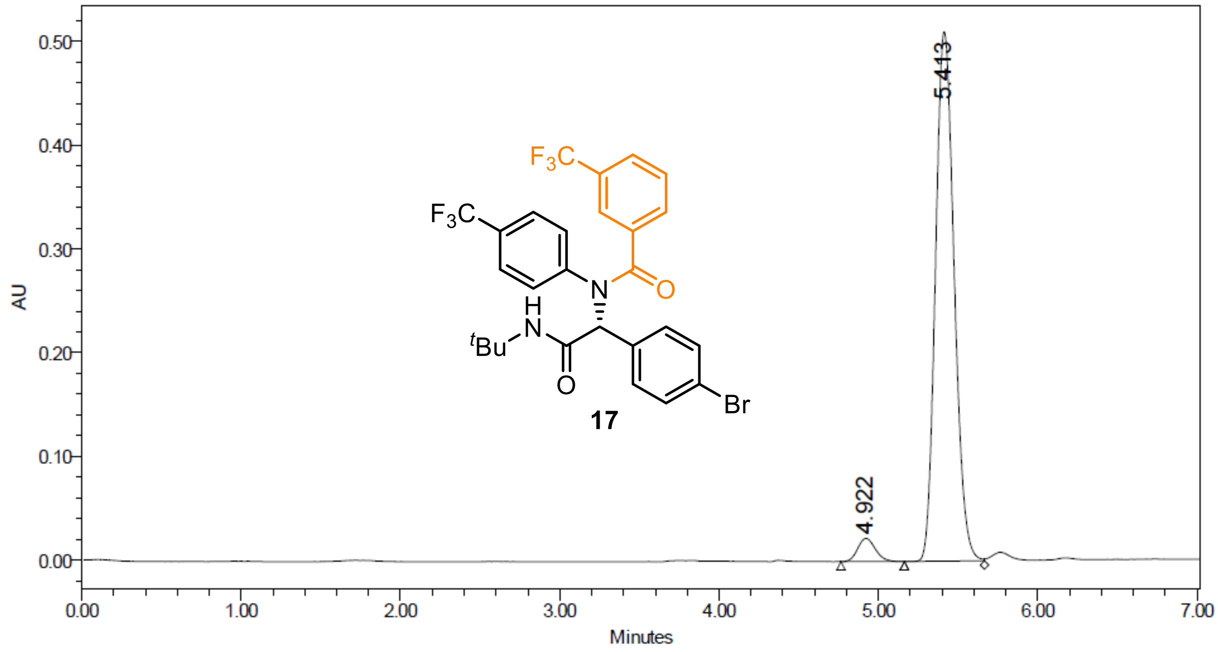

	RT (min)	Peak Type	Area (μV*sec)	% Area	Height (μV)	% Height	Integration Type	Points Across Peak	Start Time (min)	End Time (min)
1	4.922	Unknown	172122	3.98	22357	4.20	BB	239	4.765	5.163
2	5.413	Unknown	4149151	96.02	509941	95.80	BV	301	5.163	5.665

Supplementary Fig. 58. HPLC of product **17**.

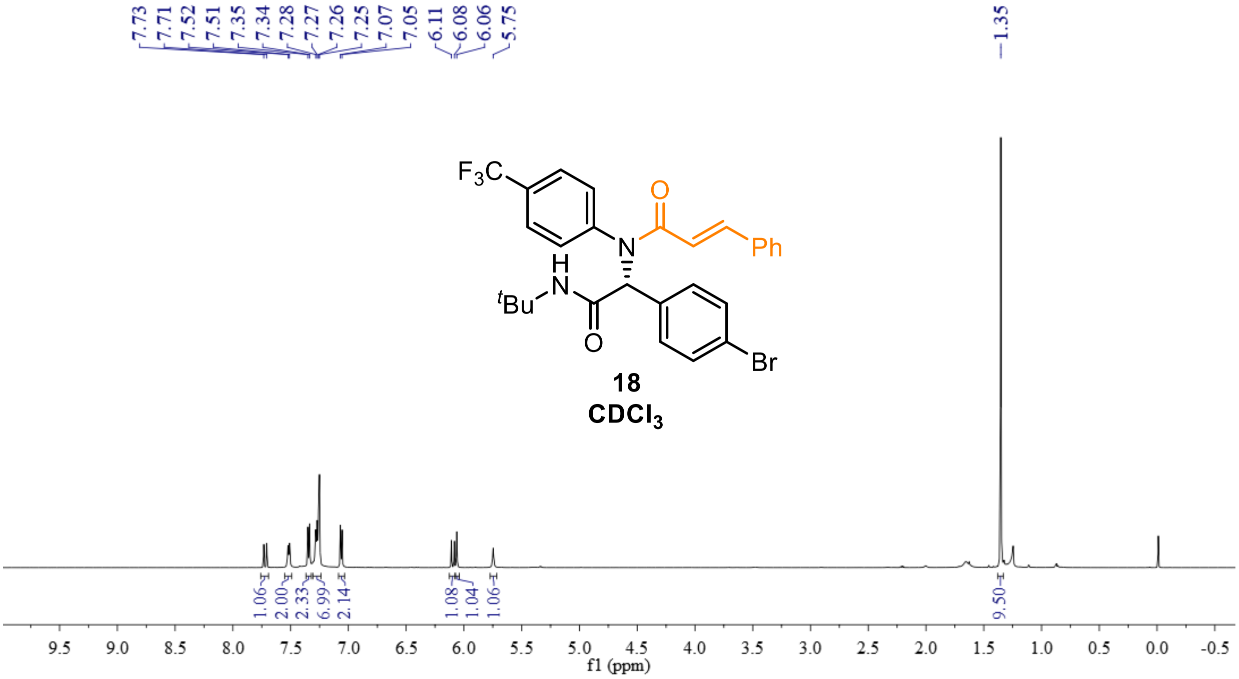

Supplementary Fig. 59. ^{1}H NMR spectrum of **18**. The sample has been recorded in 600 MHz, $CDCl_3$ at 25 °C.

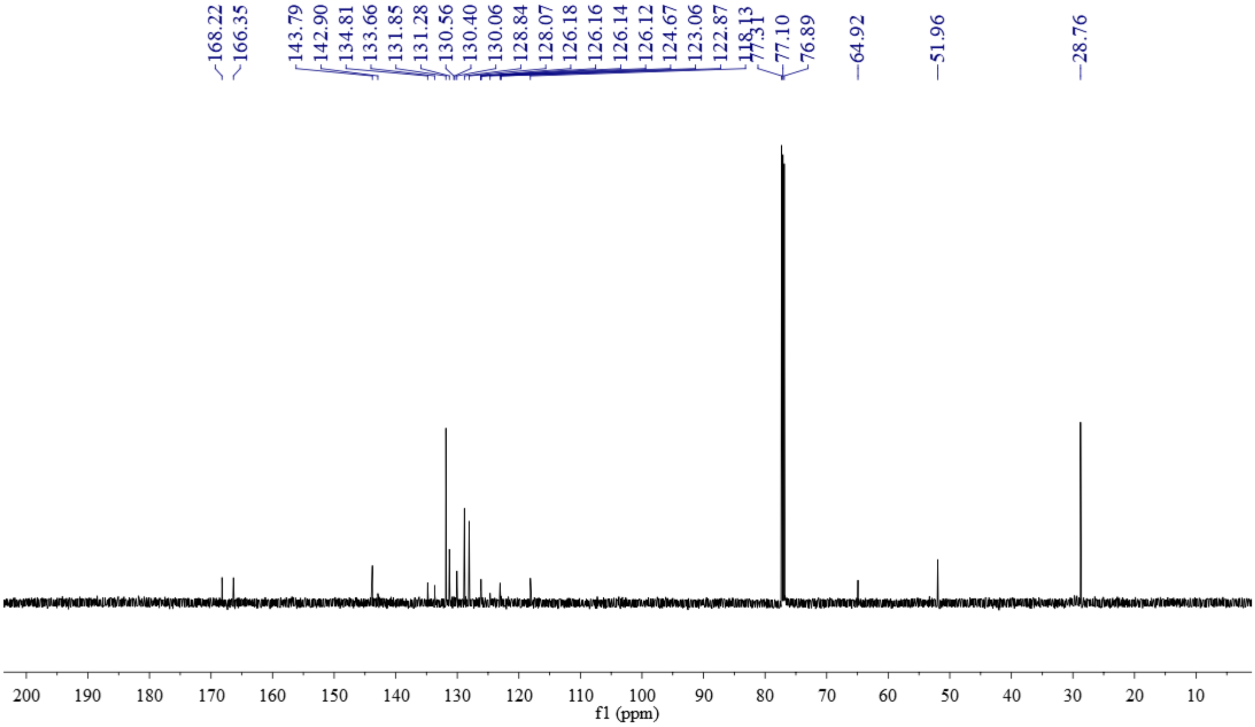

Supplementary Fig. 60.^{13}C NMR spectrum of **18**. The sample has been recorded in 151 MHz, $CDCl_3$ at 25 °C.

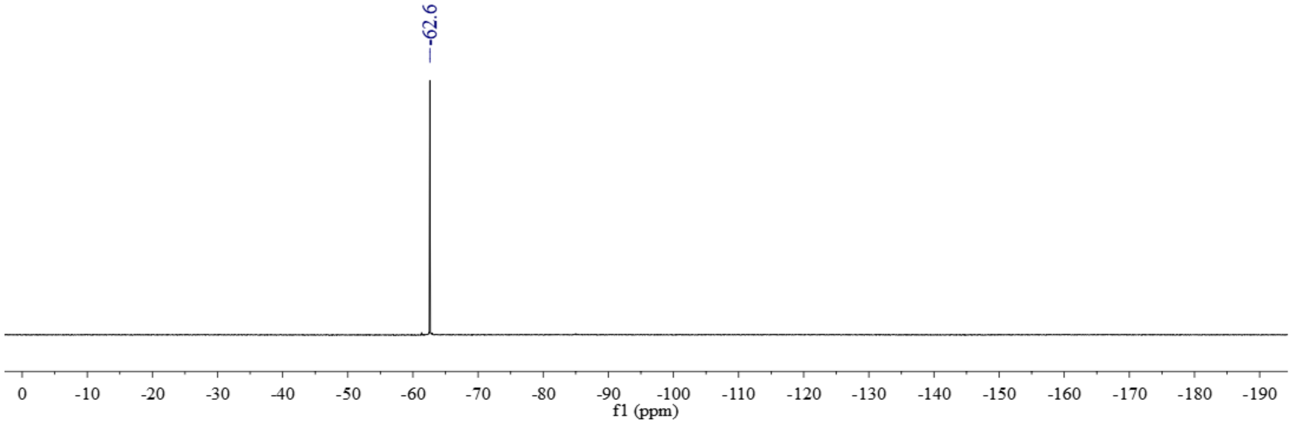

Supplementary Fig. 61. ^{31}F NMR spectrum of **18**. The sample has been recorded in 564 MHz, $CDCl_3$ at 25 °C.

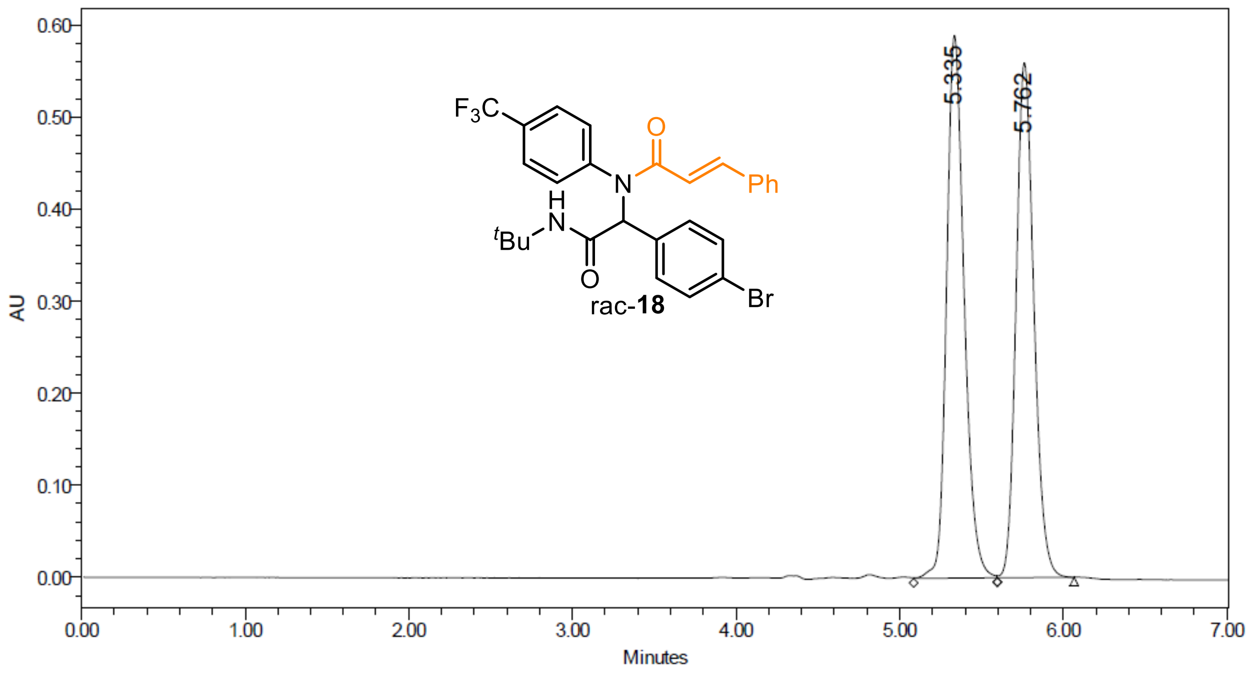

	RT (min)	Peak Type	Area (µV*sec)	% Area	Height (µV)	% Height	Integration Type	Points Across Peak	Start Time (min)	End Time (min)
1	5.335	Unknown	4411032	51.09	589536	51.33	VV	307	5.085	5.597
2	5.762	Unknown	4223317	48.91	558997	48.67	VB	282	5.597	6.067

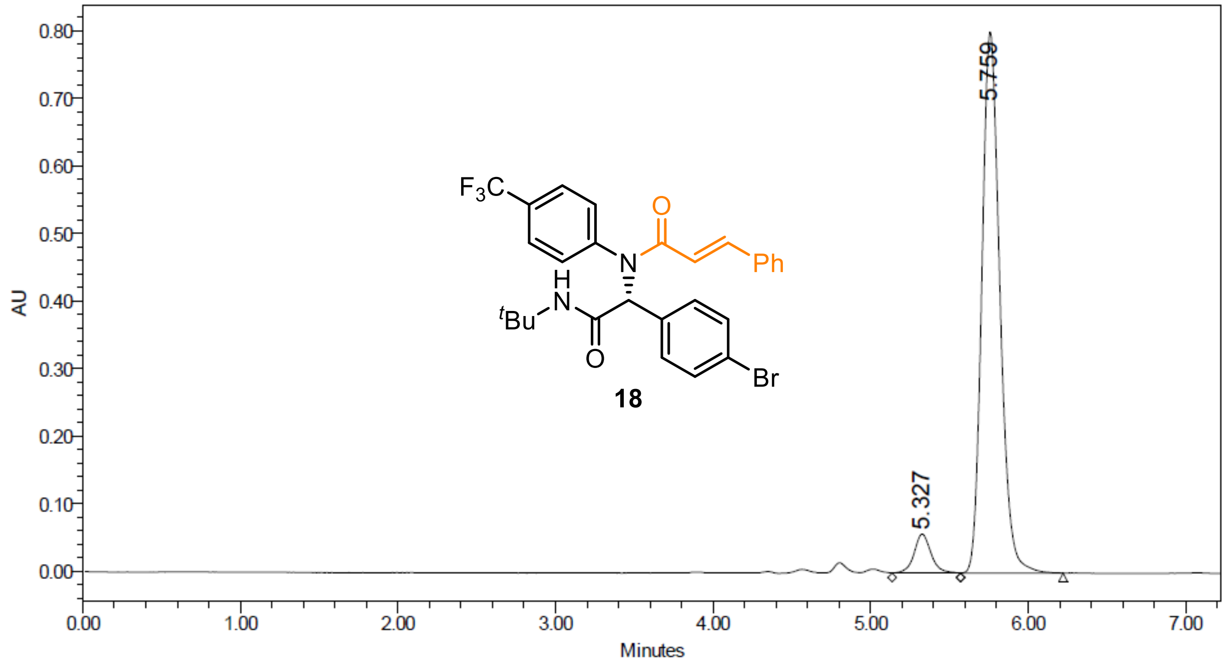

	RT (min)	Peak Type	Area (µV*sec)	% Area	Height (µV)	% Height	Integration Type	Points Across Peak	Start Time (min)	End Time (min)
1	5.327	Unknown	435312	6.41	57881	6.74	VV	261	5.137	5.572
2	5.759	Unknown	6356196	93.59	800342	93.26	VB	390	5.572	6.222

Supplementary Fig. 62. HPLC of product **18**.

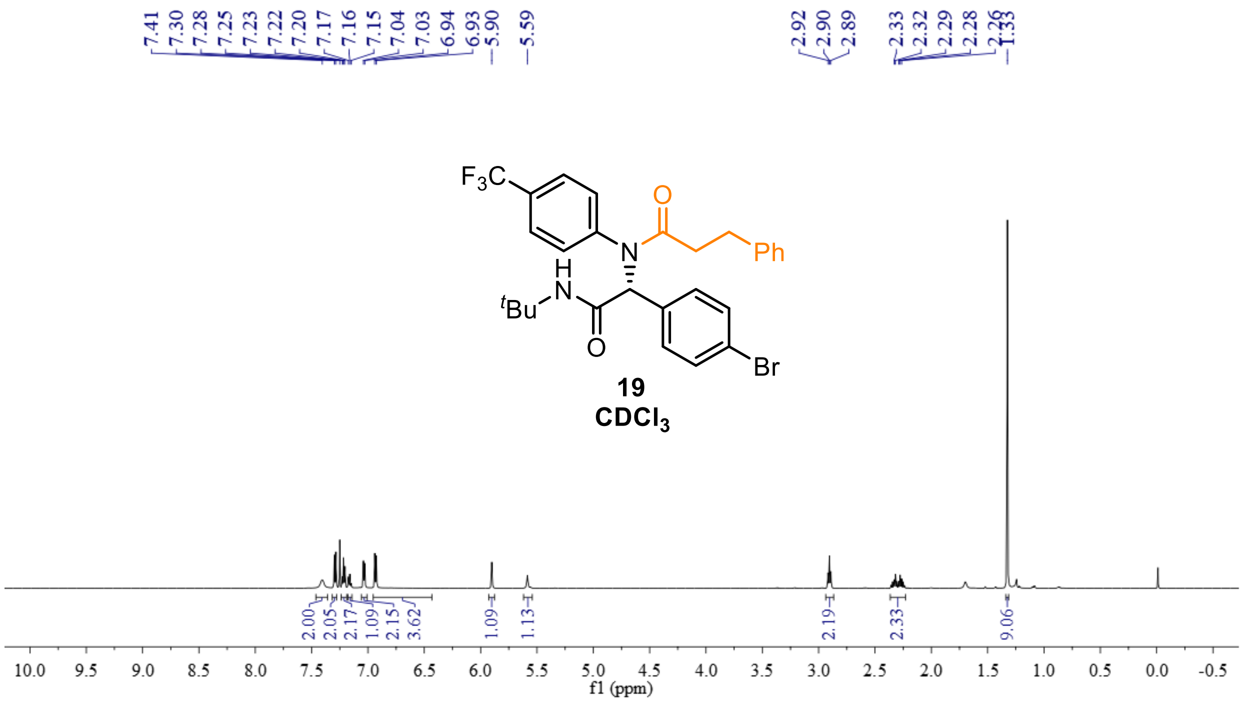

Supplementary Fig. 63. ^{1}H NMR spectrum of **19**. The sample has been recorded in 600 MHz, $CDCl_3$ at 25 °C.

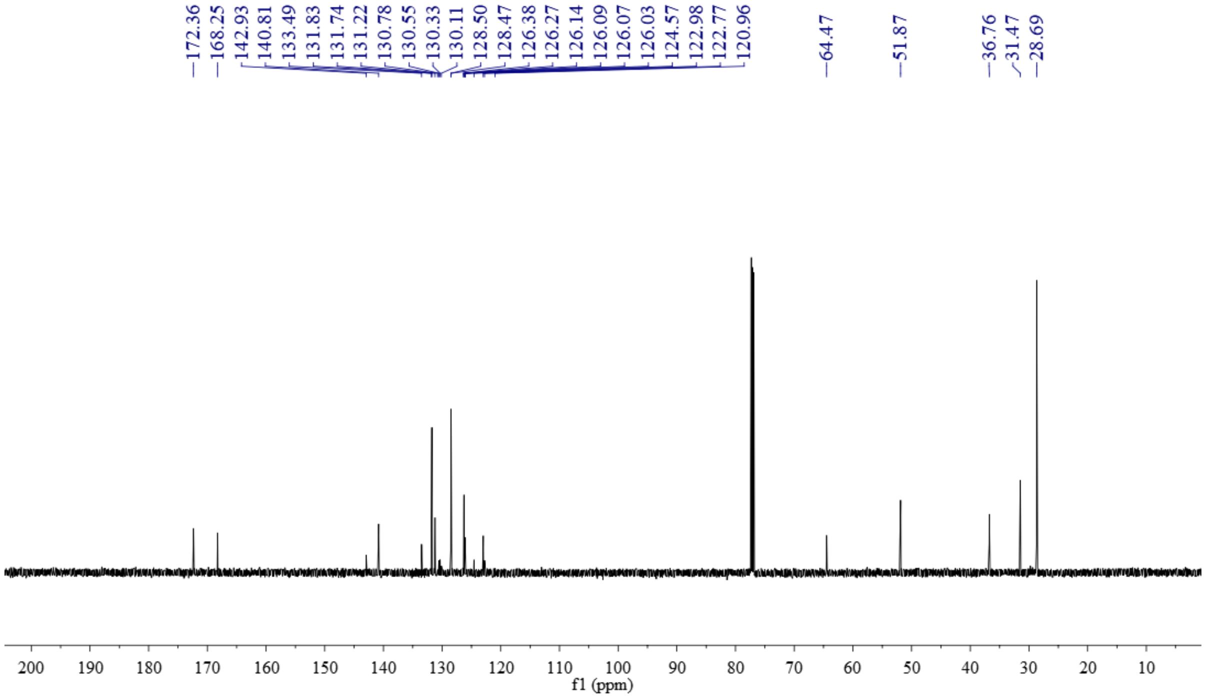

Supplementary Fig. 64.^{13}C NMR spectrum of **19**. The sample has been recorded in 151 MHz, $CDCl_3$ at 25 °C.

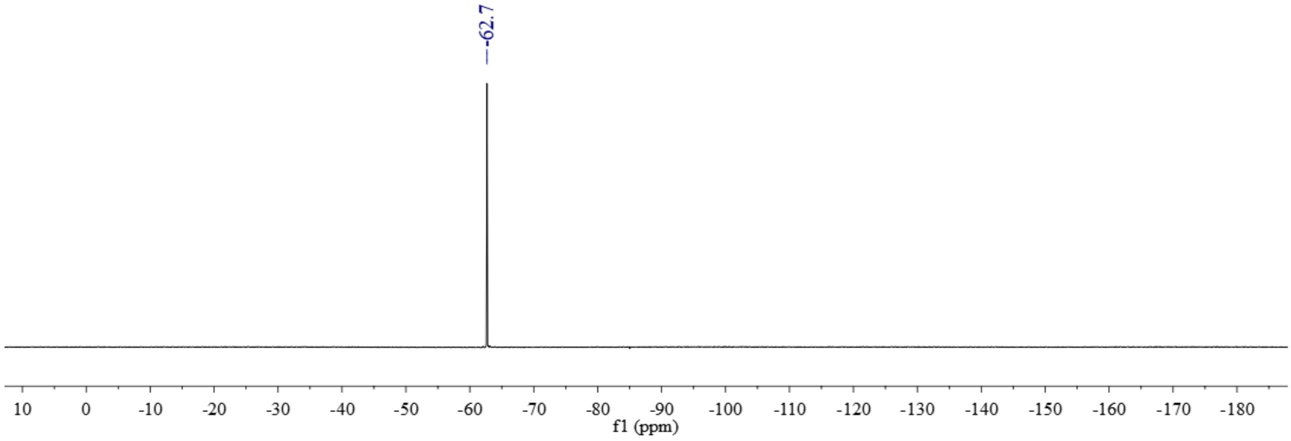

Supplementary Fig. 65. ^{31}F NMR spectrum of **19**. The sample has been recorded in 564 MHz, $CDCl_3$ at 25 °C.

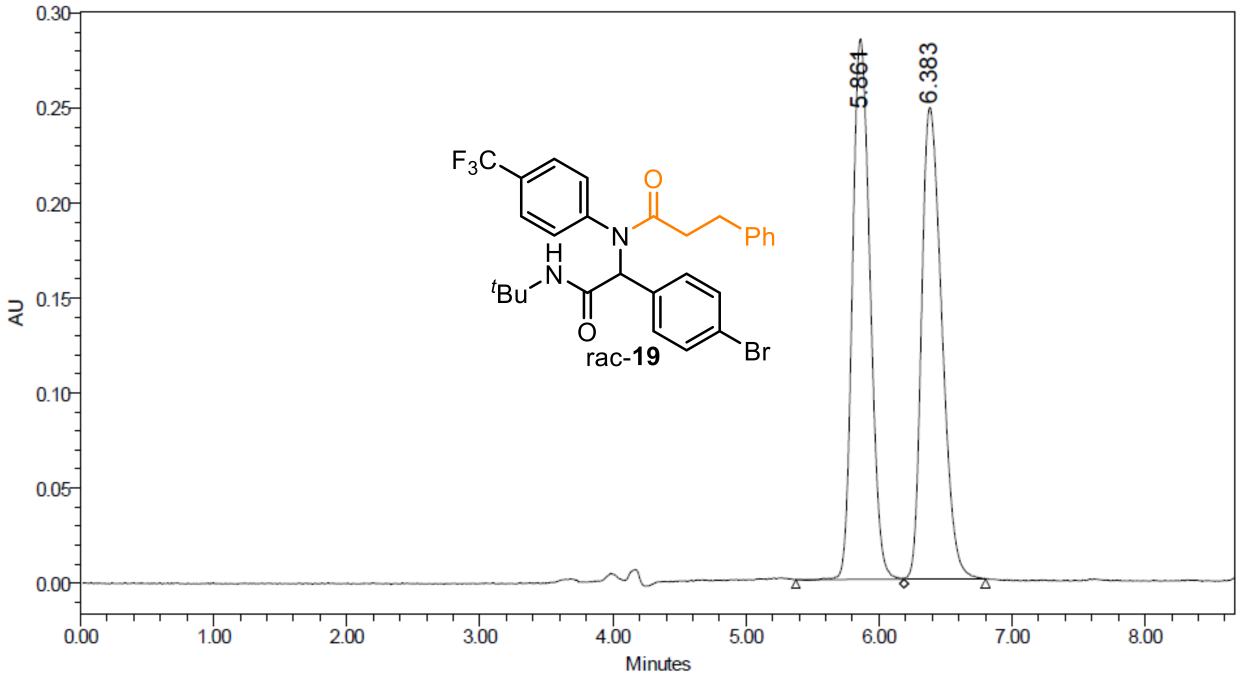

	RT (min)	Peak Type	Area (µV*sec)	% Area	Height (µV)	% Height	Integration Type	Points Across Peak	Start Time (min)	End Time (min)
1	5.861	Unknown	2627324	50.02	284505	53.40	bV	488	5.375	6.188
2	6.383	Unknown	2624920	49.98	248313	46.60	VB	368	6.188	6.802

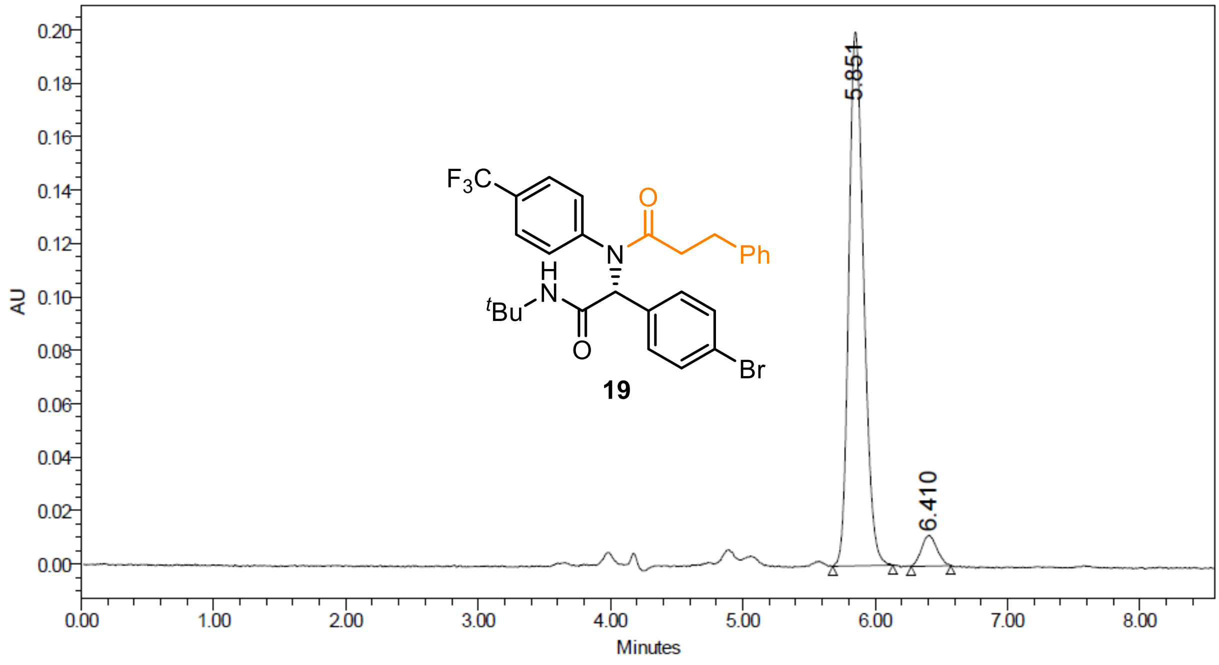

	RT (min)	Peak Type	Area (µV*sec)	% Area	Height (µV)	% Height	Integration Type	Points Across Peak	Start Time (min)	End Time (min)
1	5.851	Unknown	1561895	94.34	199885	94.55	BB	272	5.680	6.133
2	6.410	Unknown	93699	5.66	11519	5.45	BB	179	6.273	6.572

Supplementary Fig. 66. HPLC of product **19**.

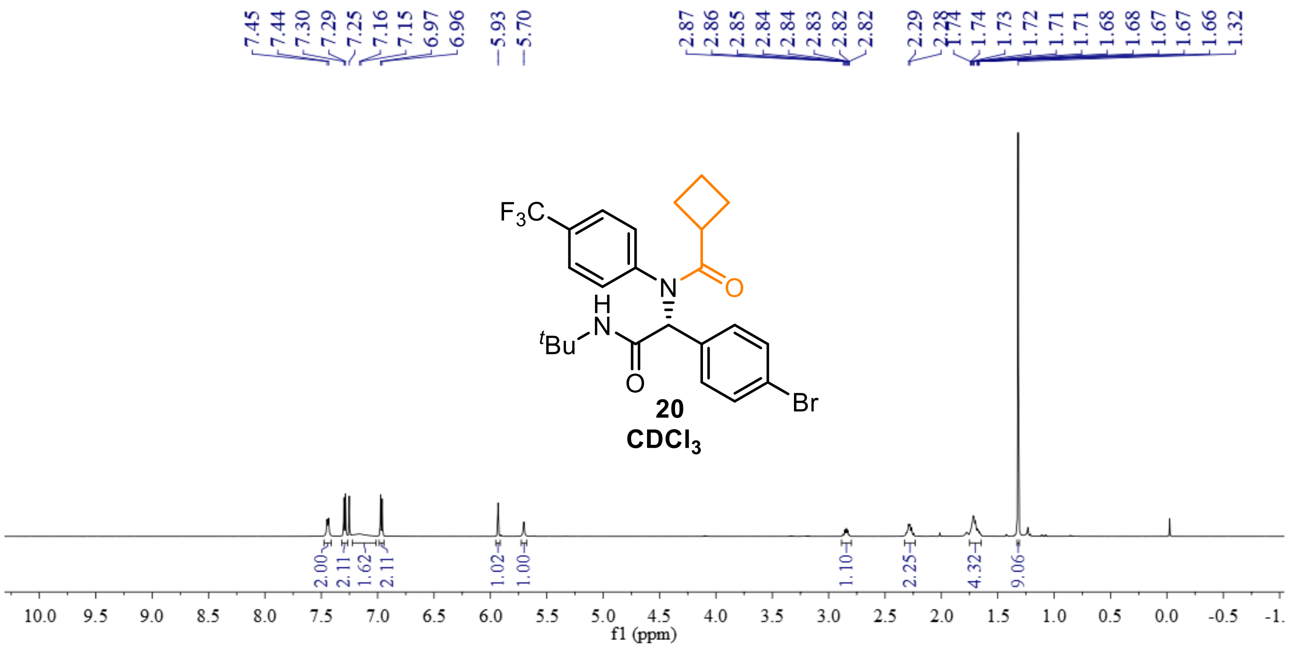

Supplementary Fig. 67. ^{1}H NMR spectrum of **20**. The sample has been recorded in 600 MHz, $CDCl_3$ at 25 °C.

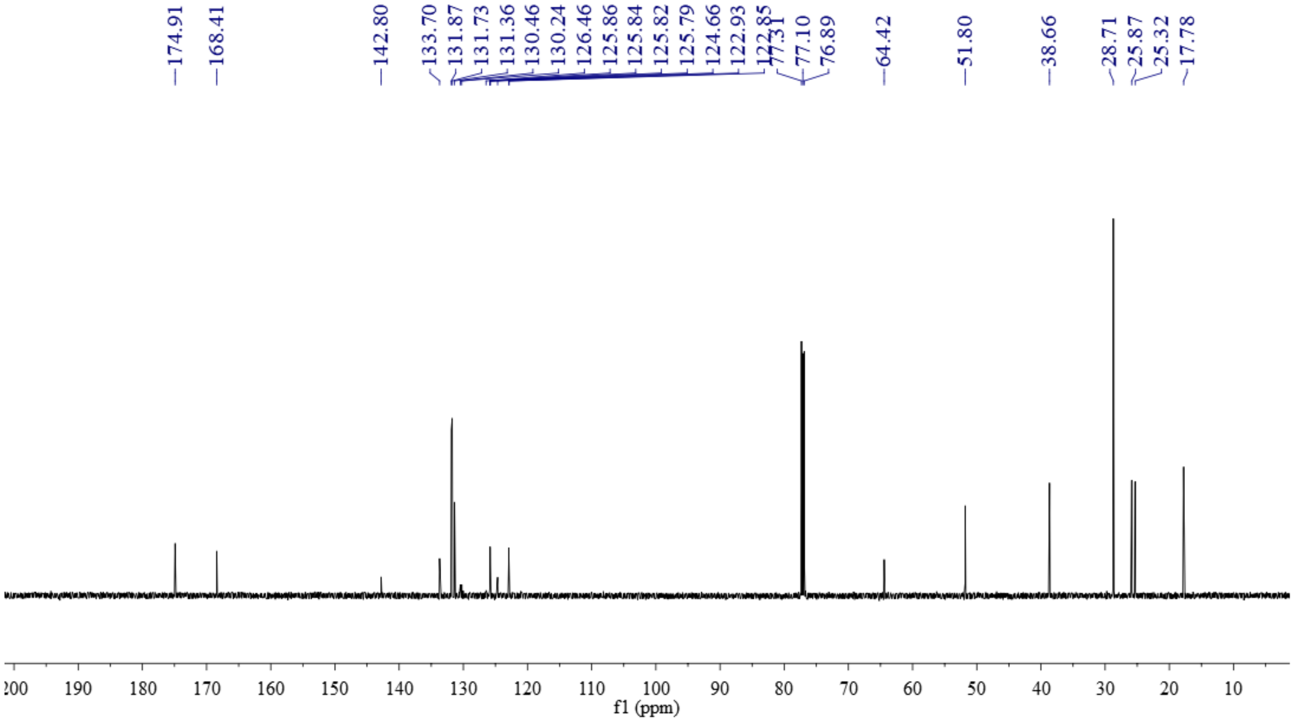

Supplementary Fig. 68.^{13}C NMR spectrum of **20**. The sample has been recorded in 151 MHz, $CDCl_3$ at 25 °C.

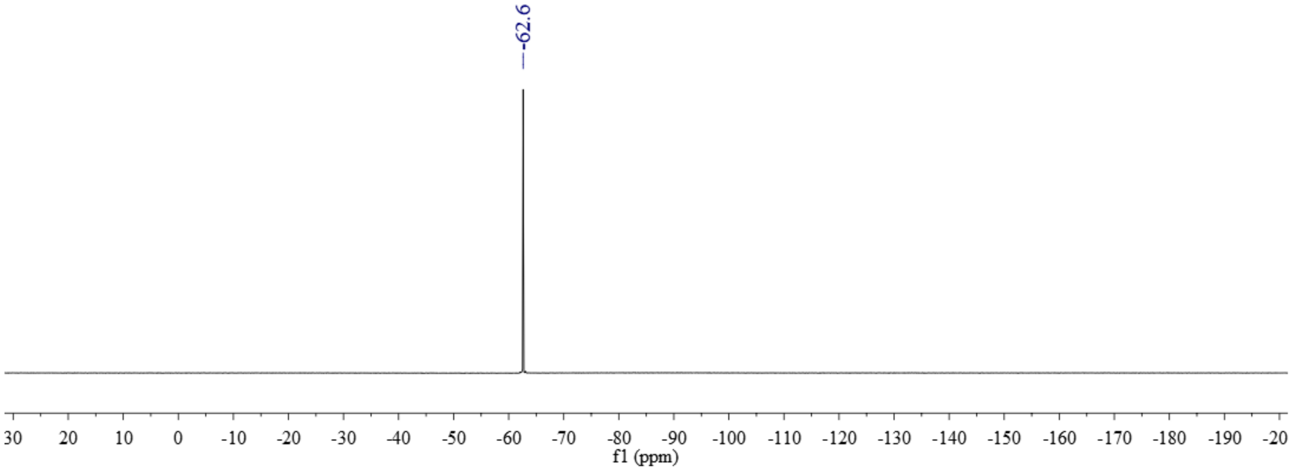

Supplementary Fig. 69. ^{31}F NMR spectrum of **20**. The sample has been recorded in 564 MHz, $CDCl_3$ at 25 °C.

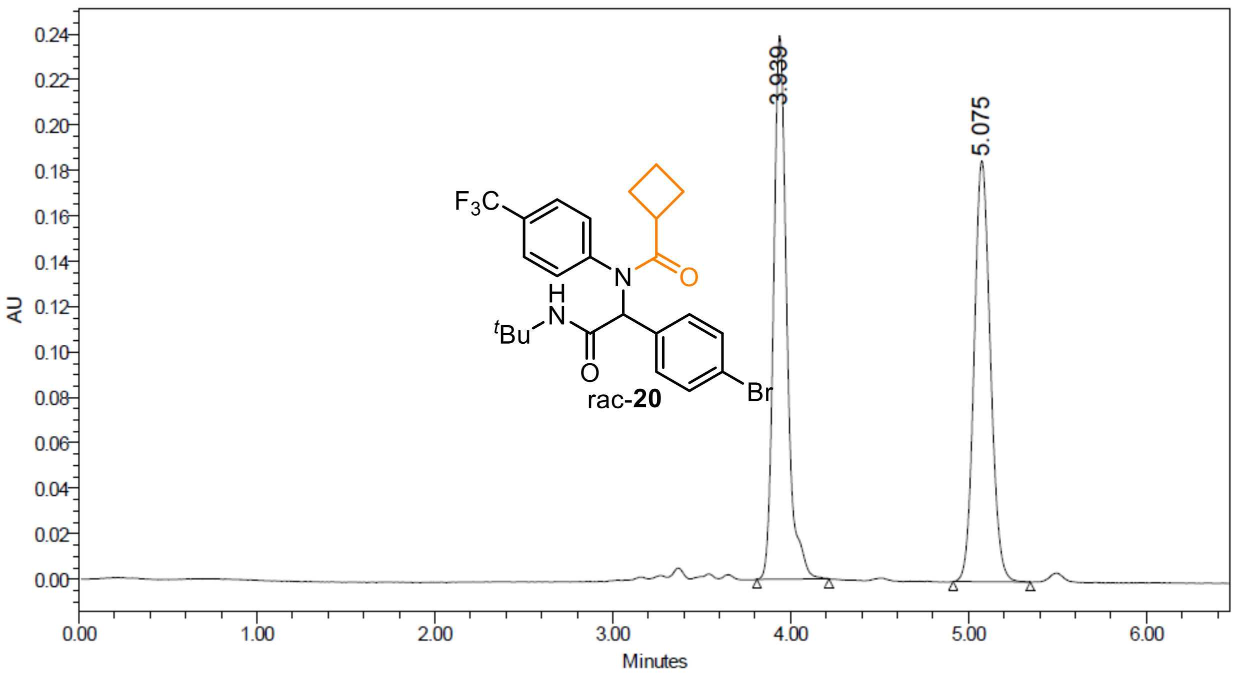

	RT (min)	Peak Type	Area (μV*sec)	% Area	Height (μV)	% Height	Integration Type	Points Across Peak	Start Time (min)	End Time (min)
1	3.939	Unknown	1233976	51.15	239332	56.36	BB	243	3.810	4.215
2	5.075	Unknown	1178678	48.85	185304	43.64	BB	260	4.913	5.347

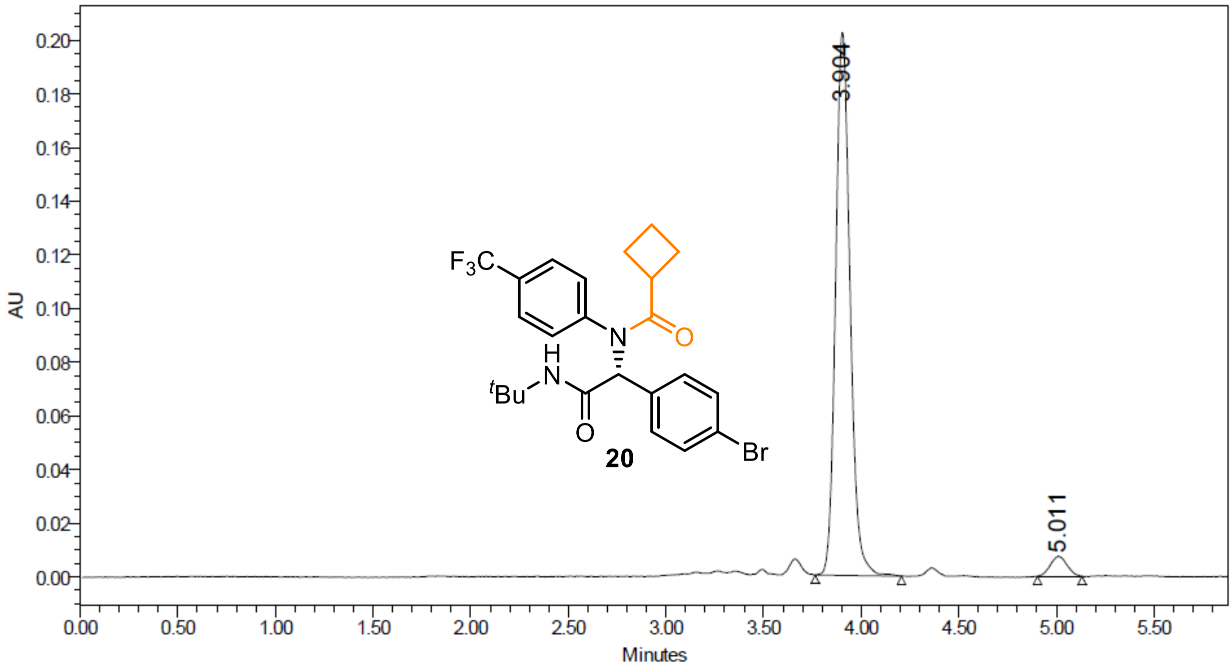

	RT (min)	Peak Type	Area (μV*sec)	% Area	Height (μV)	% Height	Integration Type	Points Across Peak	Start Time (min)	End Time (min)
1	3.904	Unknown	1039858	95.93	202329	96.50	Bb	265	3.765	4.207
2	5.011	Unknown	44123	4.07	7333	3.50	bb	137	4.903	5.132

Supplementary Fig. 70. HPLC of product **20**.

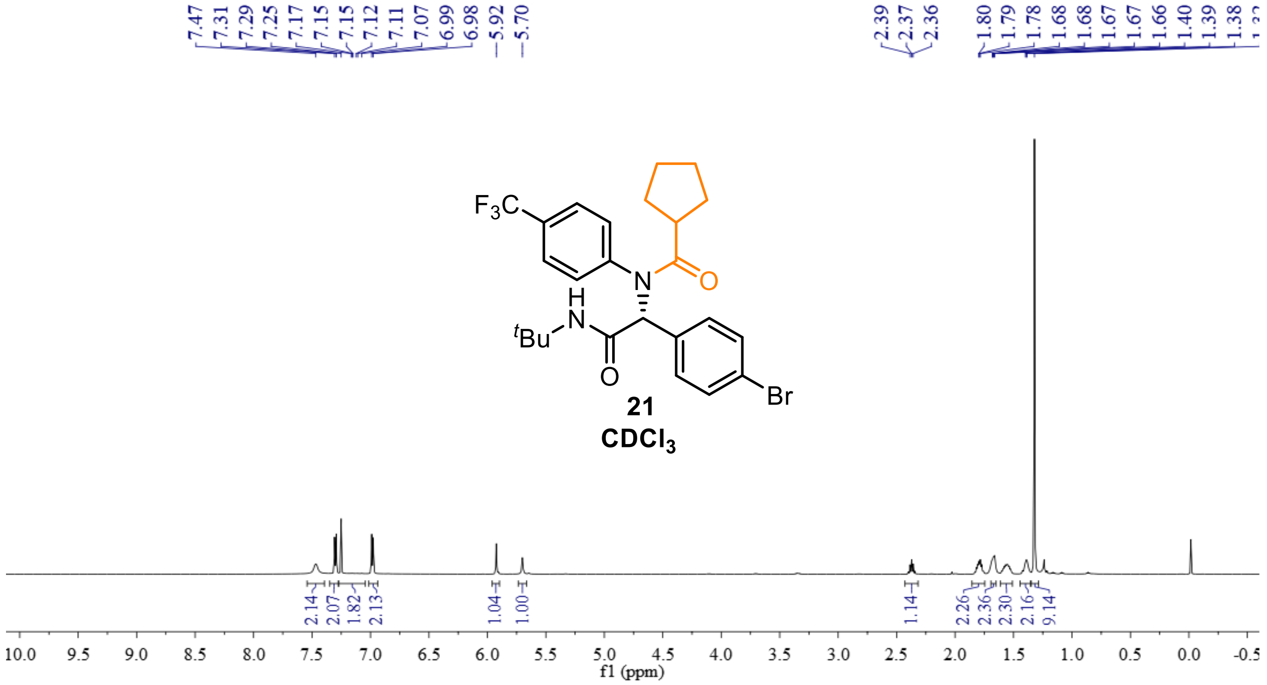

Supplementary Fig. 71. ^{1}H NMR spectrum of **21**. The sample has been recorded in 600 MHz, $CDCl_3$ at 25 °C.

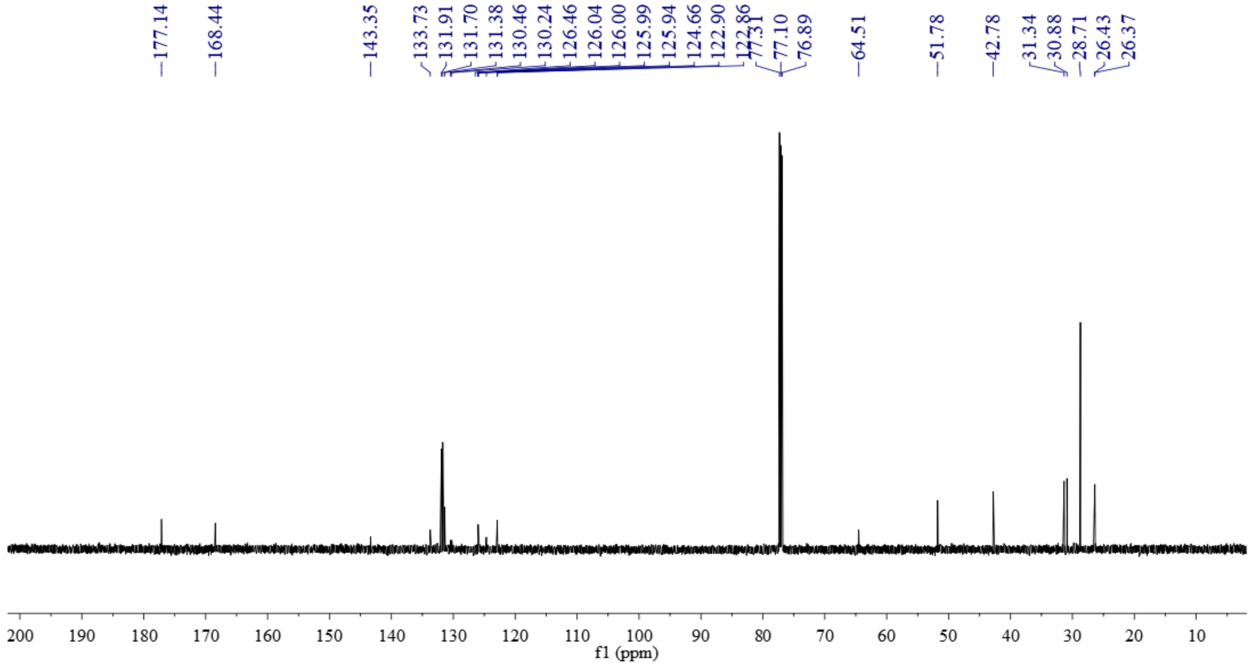

Supplementary Fig. 72.^{13}C NMR spectrum of **21**. The sample has been recorded in 151 MHz, $CDCl_3$ at 25 °C.

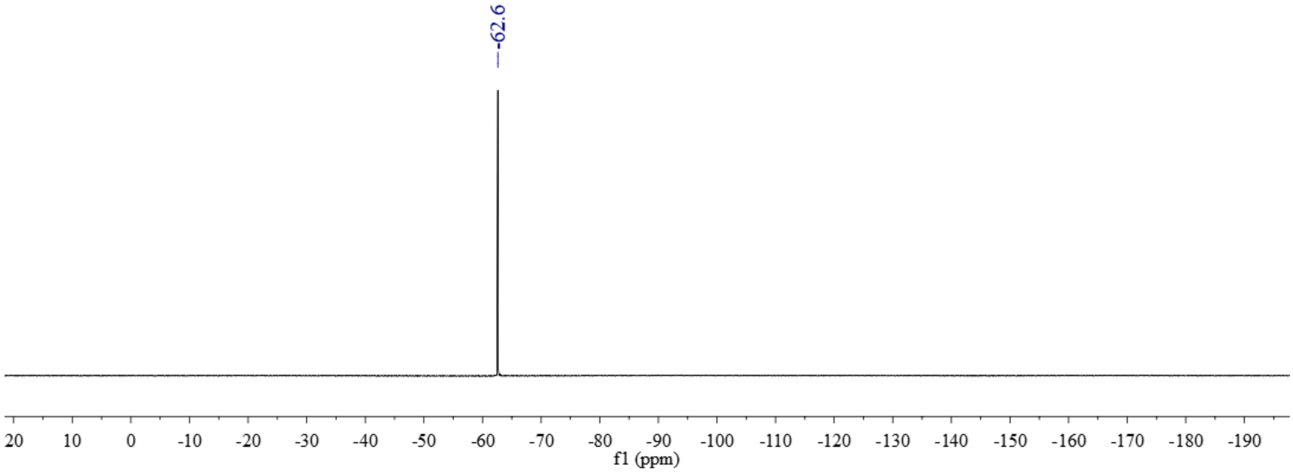

Supplementary Fig. 73. ^{31}F NMR spectrum of **21**. The sample has been recorded in 564 MHz, $CDCl_3$ at 25 °C.

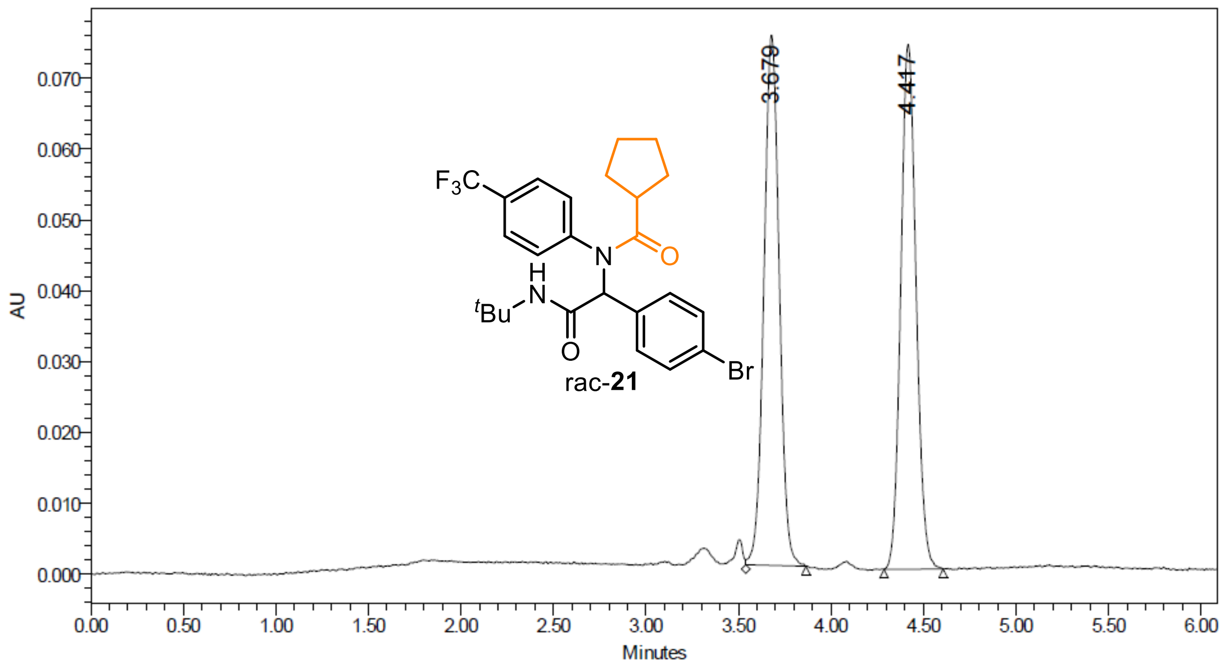

	RT (min)	Peak Type	Area (µV*sec)	% Area	Height (µV)	% Height	Integration Type	Points Across Peak	Start Time (min)	End Time (min)
1	3.679	Unknown	430520	50.26	74872	50.27	VB	195	3.540	3.865
2	4.417	Unknown	426122	49.74	74078	49.73	BB	192	4.287	4.607

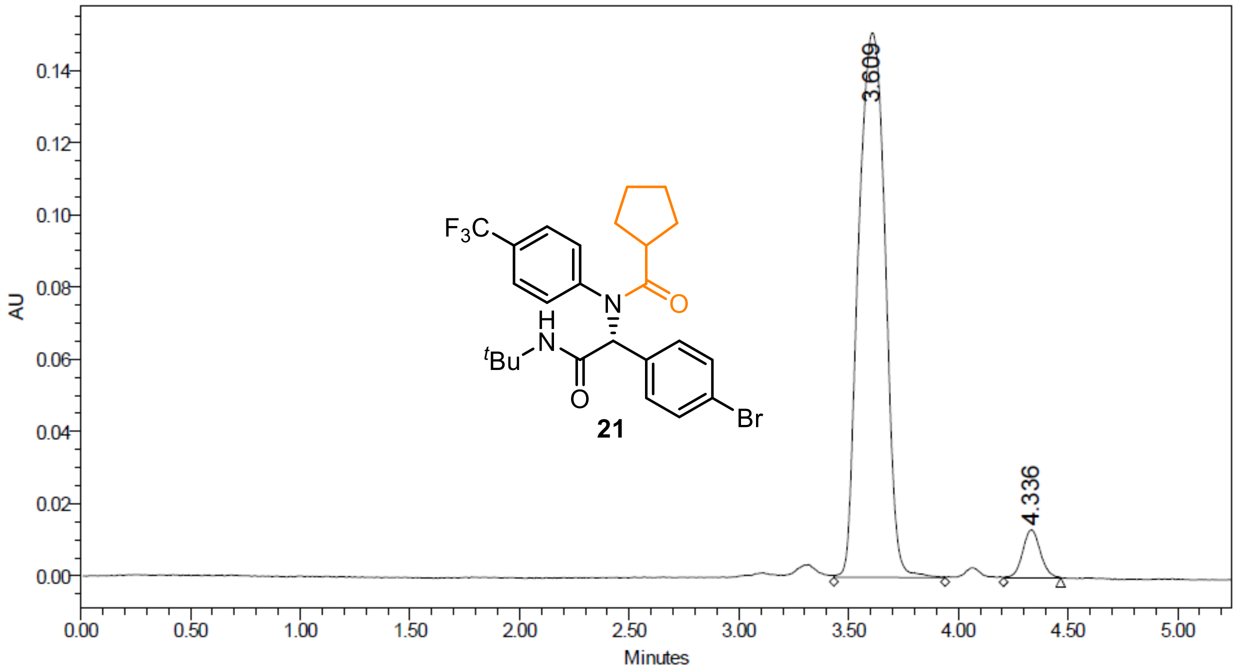

	RT (min)	Peak Type	Area (µV*sec)	% Area	Height (µV)	% Height	Integration Type	Points Across Peak	Start Time (min)	End Time (min)
1	3.609	Unknown	1269774	94.49	150734	91.95	VV	304	3.433	3.940
2	4.336	Unknown	74103	5.51	13202	8.05	Vb	156	4.207	4.467

Supplementary Fig. 74. HPLC of product **21**.

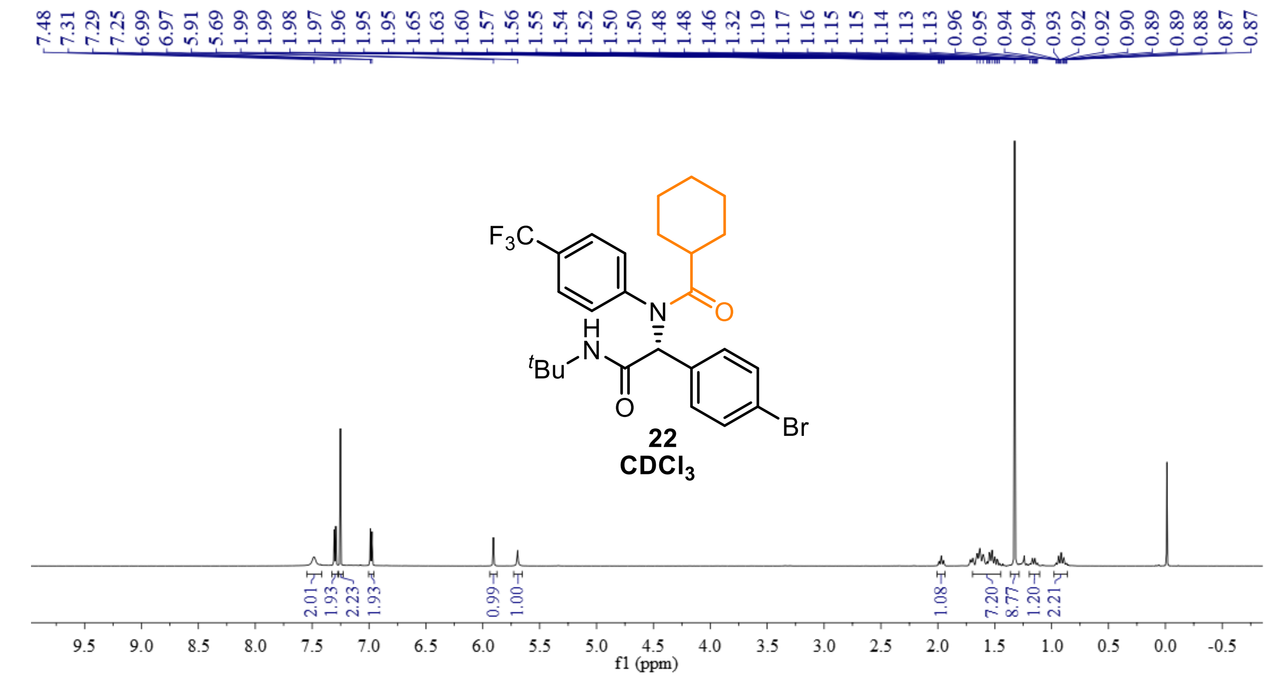

Supplementary Fig. 75. ^{1}H NMR spectrum of **22**. The sample has been recorded in 600 MHz, $CDCl_3$ at 25 °C.

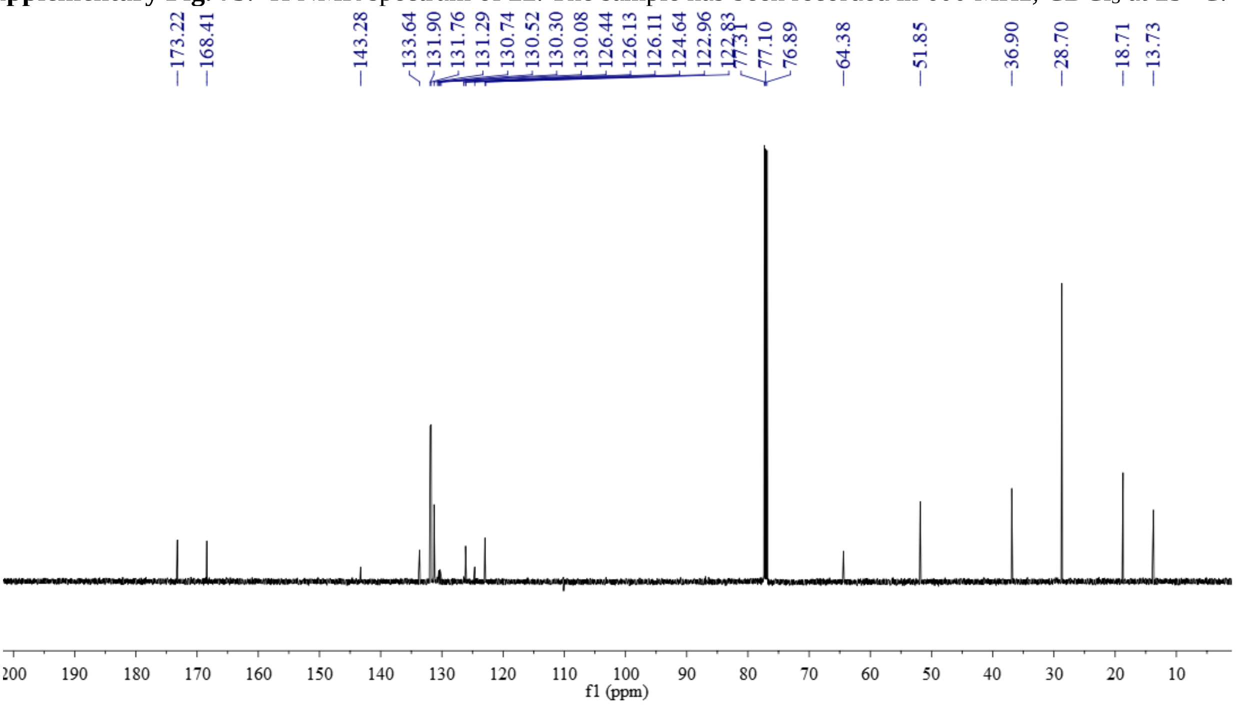

Supplementary Fig. 76.^{13}C NMR spectrum of **22**. The sample has been recorded in 151 MHz, $CDCl_3$ at 25 °C.

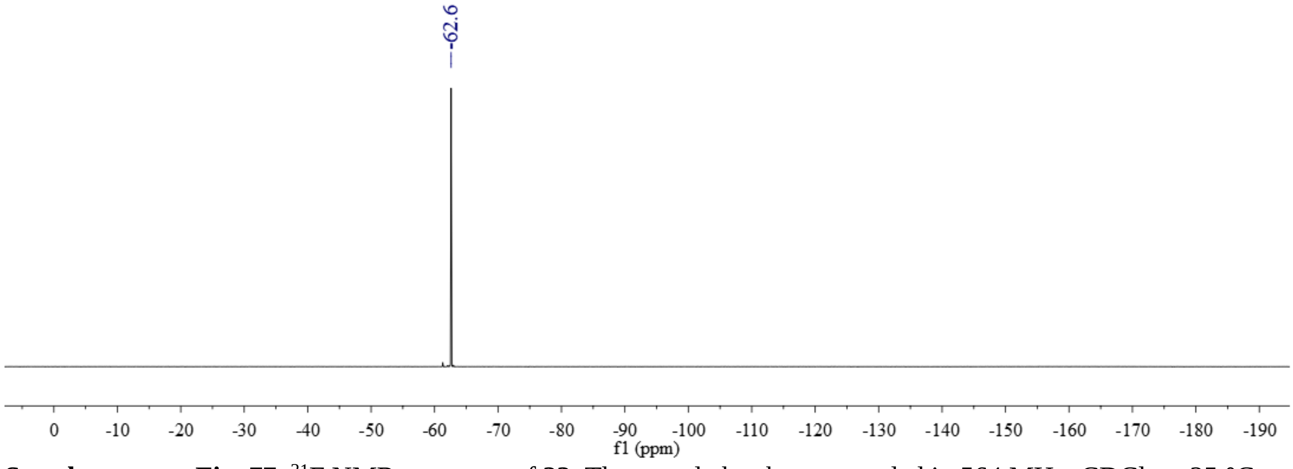

Supplementary Fig. 77. ^{31}F NMR spectrum of **23**. The sample has been recorded in 564 MHz, $CDCl_3$ at 25 °C.

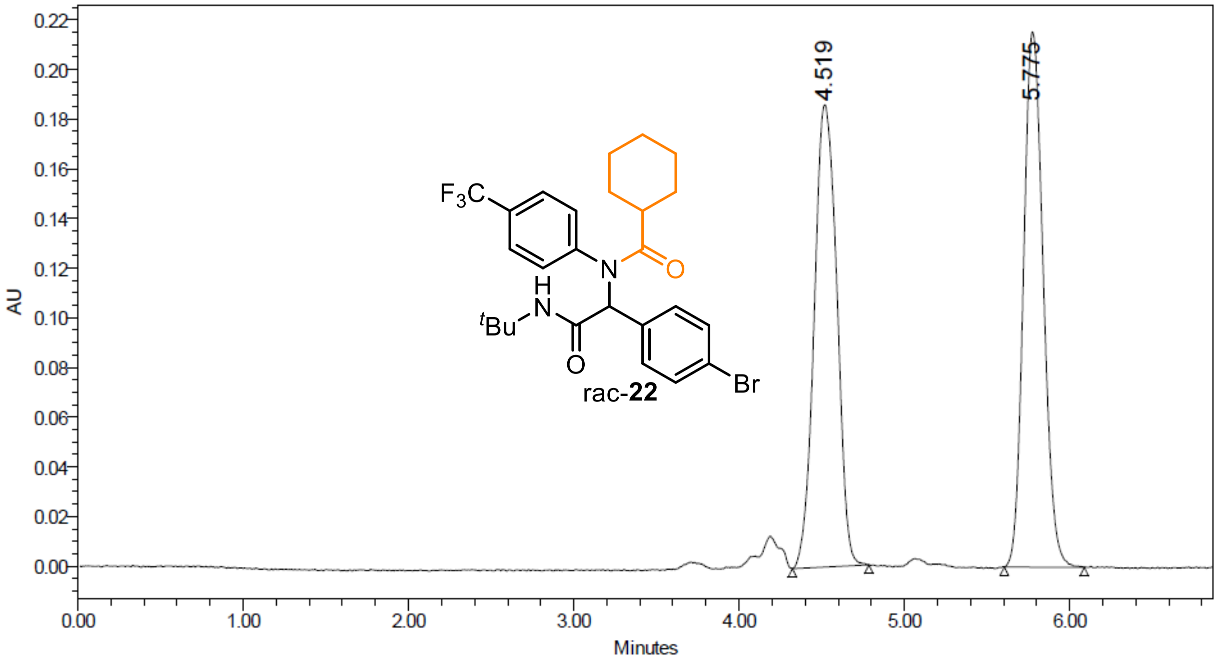

	RT (min)	Peak Type	Area (μV*sec)	% Area	Height (μV)	% Height	Integration Type	Points Across Peak	Start Time (min)	End Time (min)
1	4.519	Unknown	1769274	49.77	186113	46.34	BB	277	4.323	4.785
2	5.775	Unknown	1785698	50.23	215534	53.66	BB	290	5.605	6.088

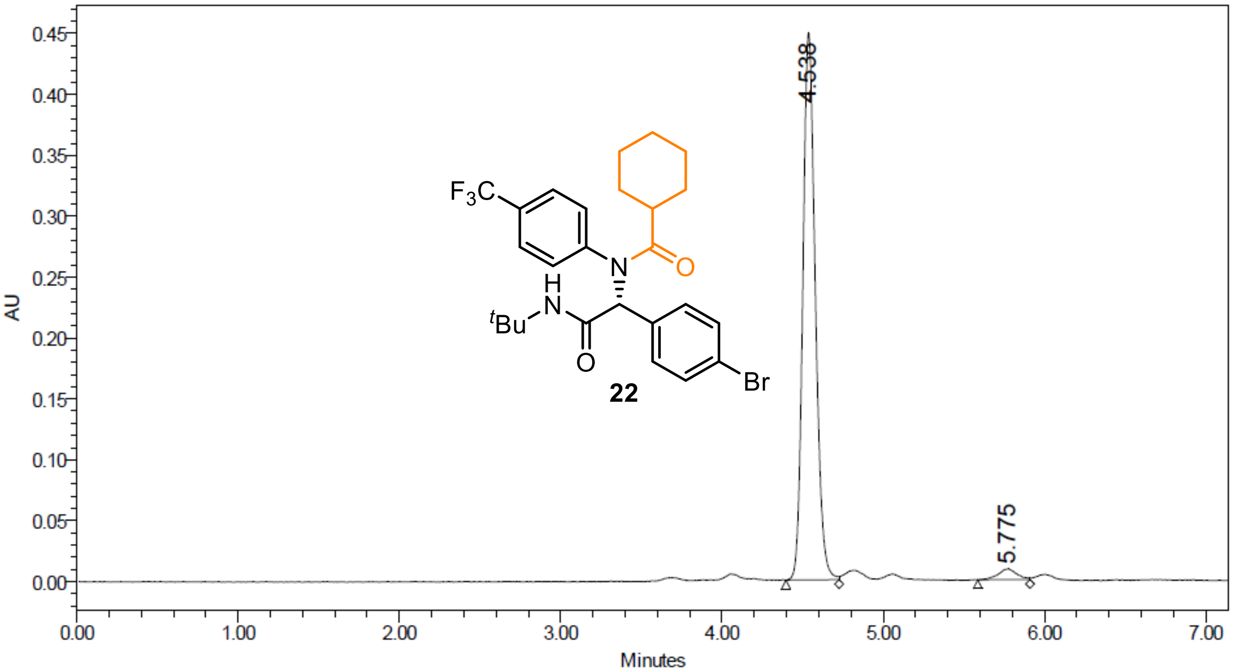

	RT (min)	Peak Type	Area (μV*sec)	% Area	Height (μV)	% Height	Integration Type	Points Across Peak	Start Time (min)	End Time (min)
1	4.538	Unknown	2406857	97.13	449633	98.06	BV	198	4.397	4.727
2	5.775	Unknown	71111	2.87	8900	1.94	bV	194	5.587	5.910

Supplementary Fig. 78. HPLC of product **22**.

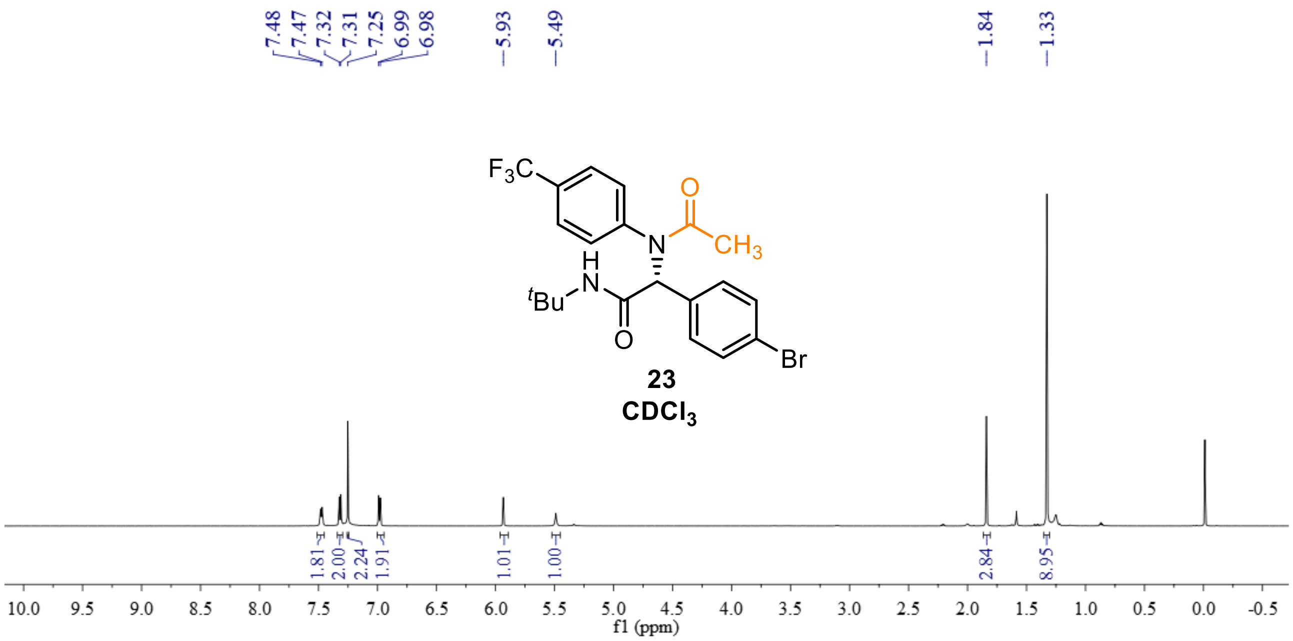

Supplementary Fig. 79. ^{1}H NMR spectrum of **23**. The sample has been recorded in 600 MHz, $CDCl_3$ at 25 °C.

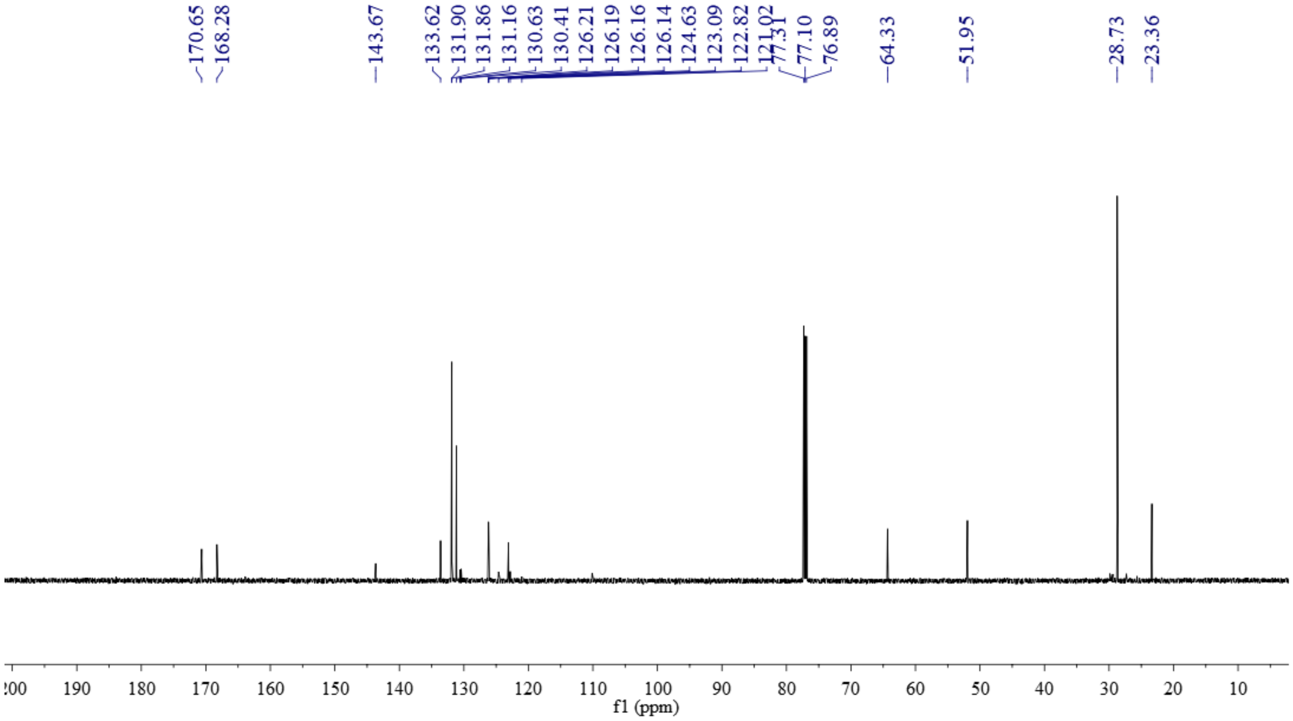

Supplementary Fig. 81.^{13}C NMR spectrum of **23**. The sample has been recorded in 151 MHz, $CDCl_3$ at 25 °C.

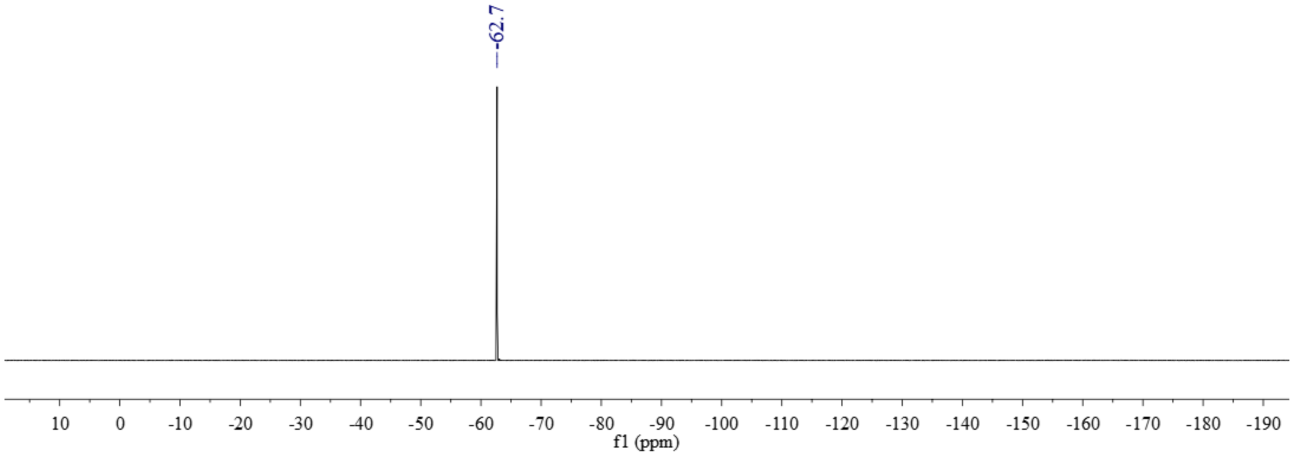

Supplementary Fig. 81. [31]F NMR spectrum of **23**. The sample has been recorded in 564 MHz, $CDCl_3$ at 25 °C.

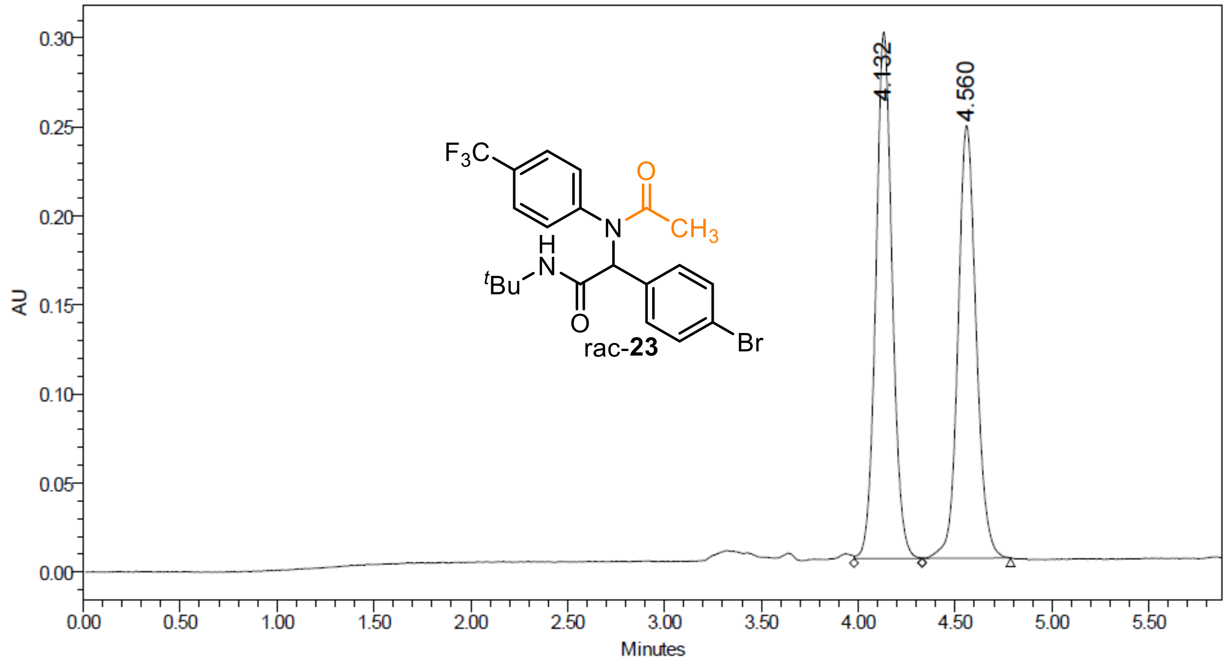

	RT (min)	Peak Type	Area (µV*sec)	% Area	Height (µV)	% Height	Integration Type	Points Across Peak	Start Time (min)	End Time (min)
1	4.132	Unknown	1738717	52.27	295946	54.91	VV	211	3.978	4.330
2	4.560	Unknown	1587418	47.73	243016	45.09	VB	274	4.330	4.787

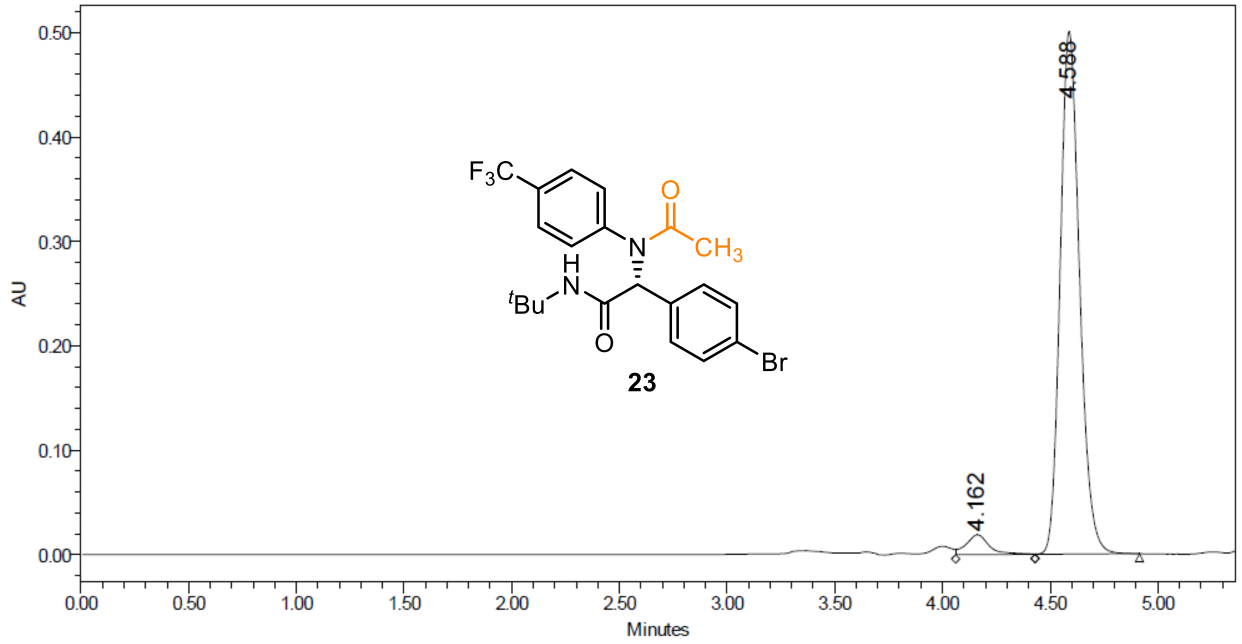

	RT (min)	Area (µV*sec)	% Area	Height (µV)	% Height
1	4.162	141874	4.33	18998	3.66
2	4.588	3131802	95.67	500501	96.34

Supplementary Fig. 82. HPLC of product **23**.

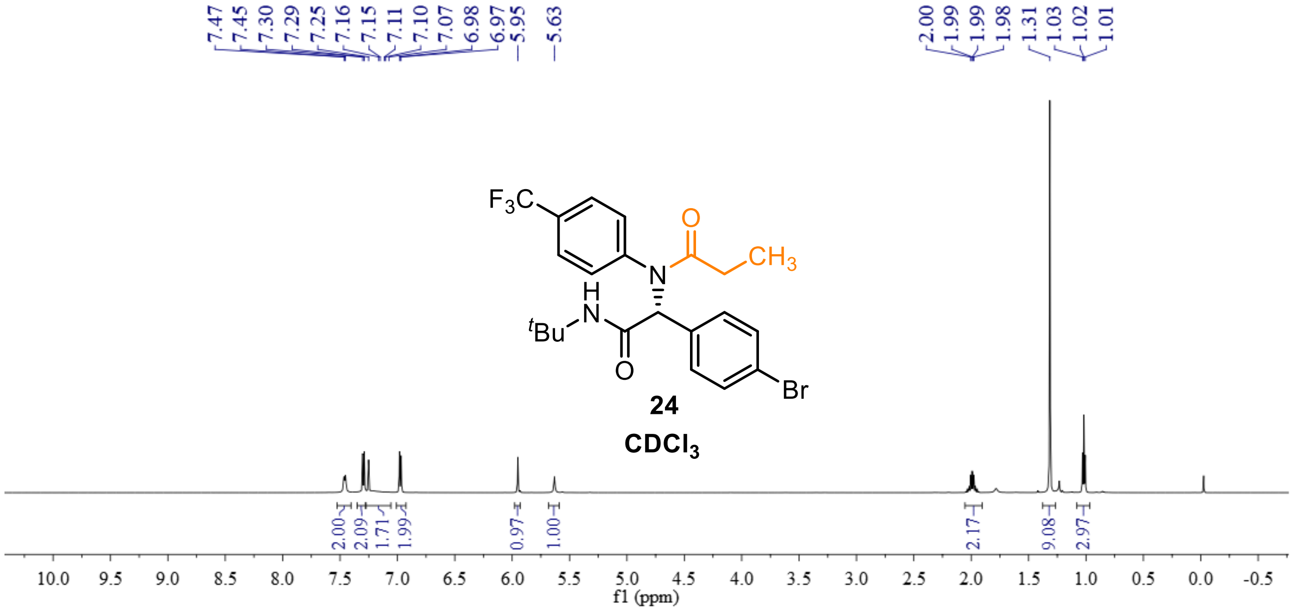

Supplementary Fig. 83. ^{1}H NMR spectrum of **24**. The sample has been recorded in 600 MHz, $CDCl_3$ at 25 °C.

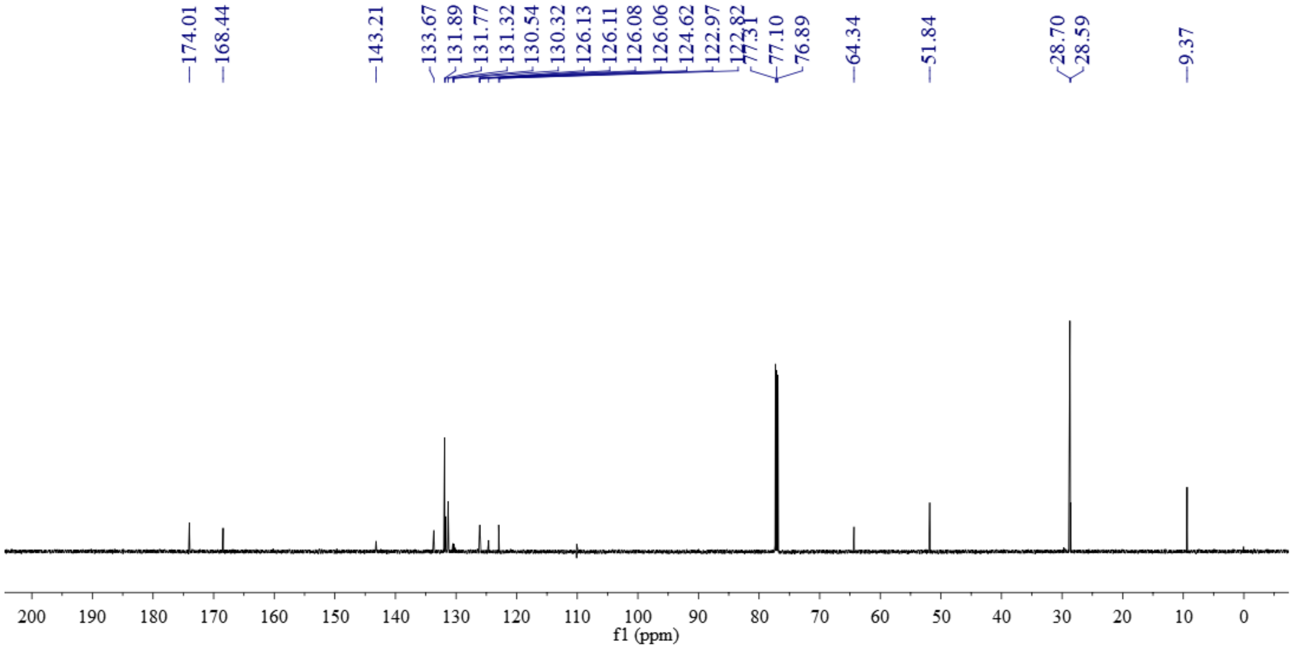

Supplementary Fig. 84.^{13}C NMR spectrum of **24**. The sample has been recorded in 151 MHz, $CDCl_3$ at 25 °C.

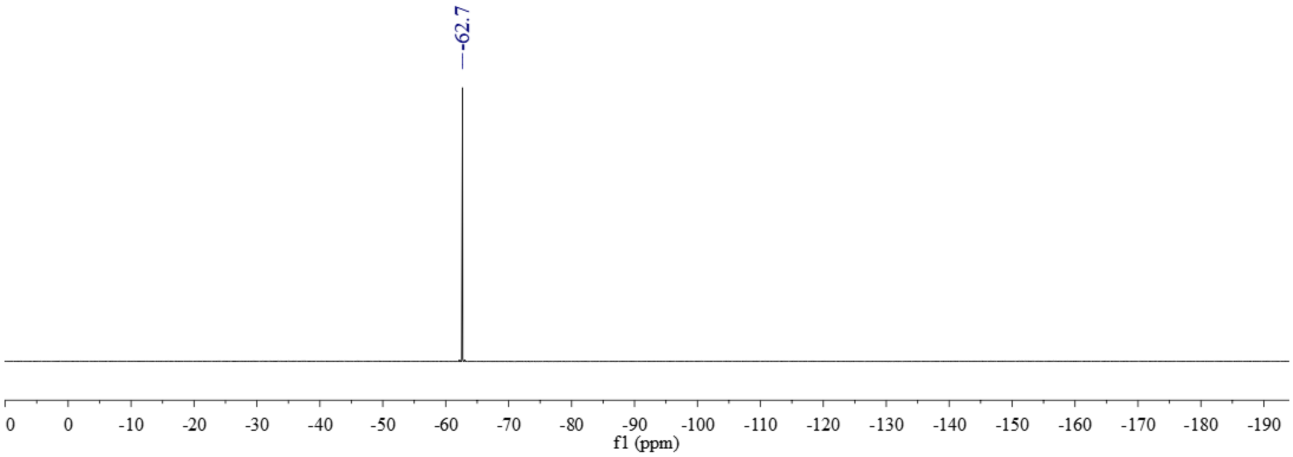

Supplementary Fig. 85. ^{31}F NMR spectrum of **24**. The sample has been recorded in 564 MHz, $CDCl_3$ at 25 °C.

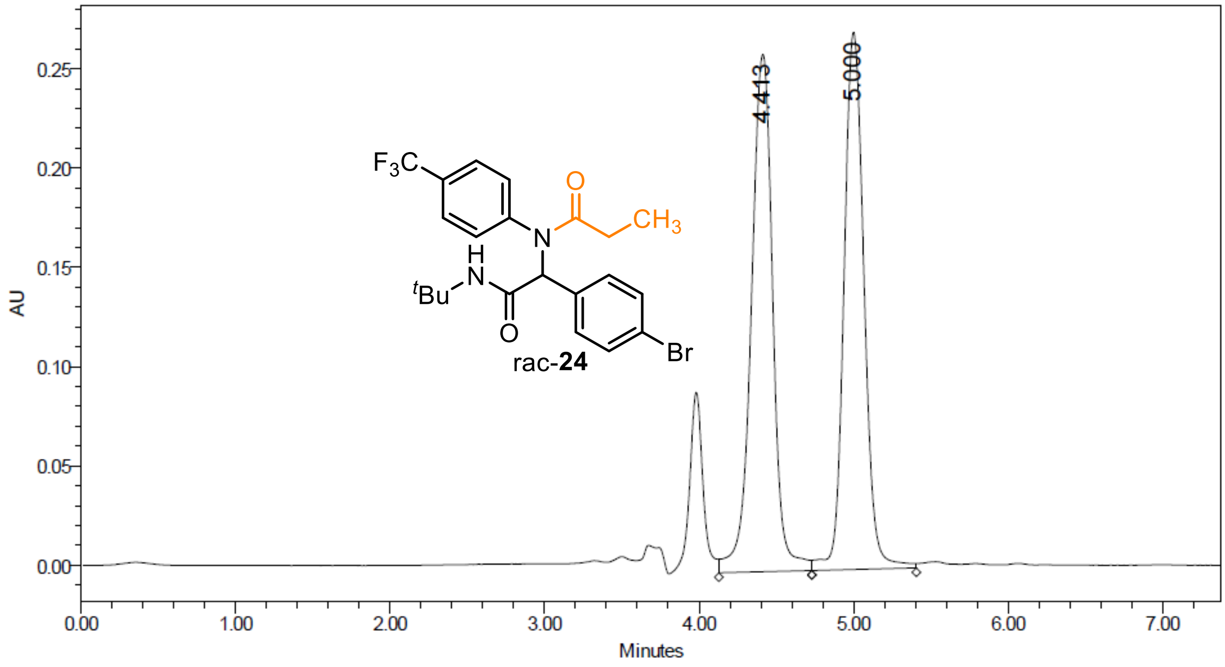

	RT (min)	Peak Type	Area (µV*sec)	% Area	Height (µV)	% Height	Integration Type	Points Across Peak	Start Time (min)	End Time (min)
1	4.413	Unknown	2522847	50.72	260403	49.05	VV	361	4.128	4.730
2	5.000	Unknown	2451495	49.28	270437	50.95	VV	406	4.730	5.407

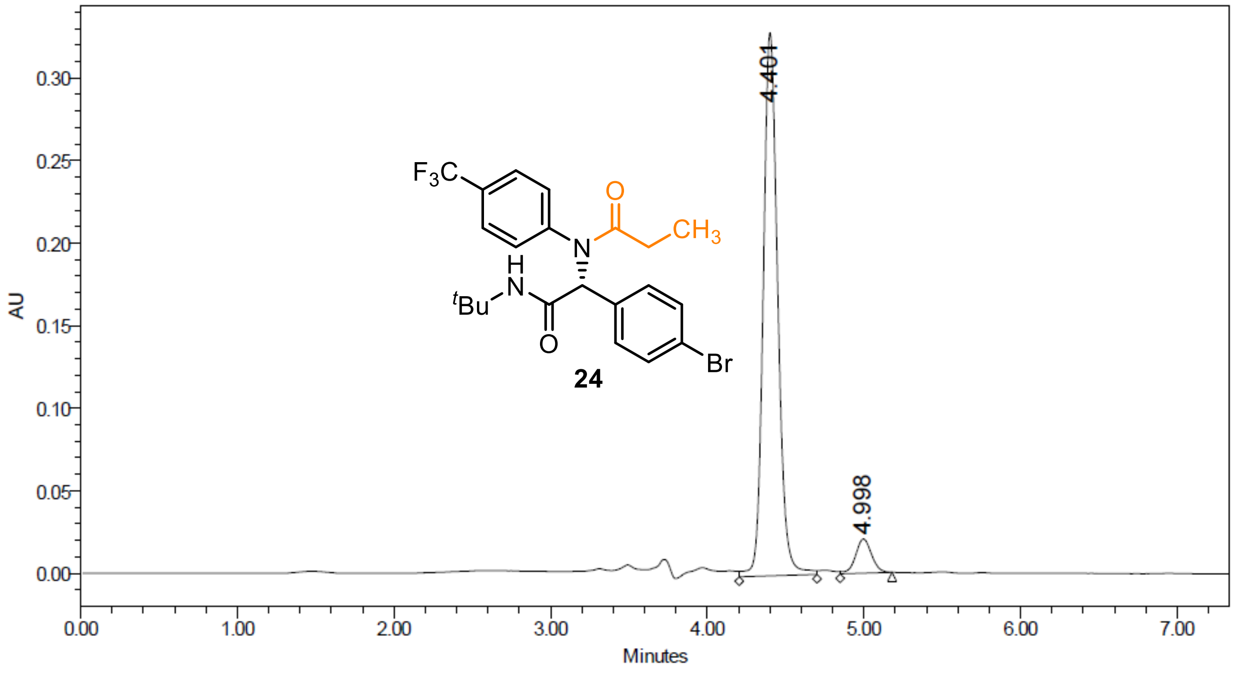

	RT (min)	Peak Type	Area (µV*sec)	% Area	Height (µV)	% Height	Integration Type	Points Across Peak	Start Time (min)	End Time (min)
1	4.401	Unknown	2117103	93.49	328977	94.09	VV	298	4.205	4.702
2	4.998	Unknown	147346	6.51	20663	5.91	Vb	199	4.848	5.180

Supplementary Fig. 86. HPLC of product **24**.

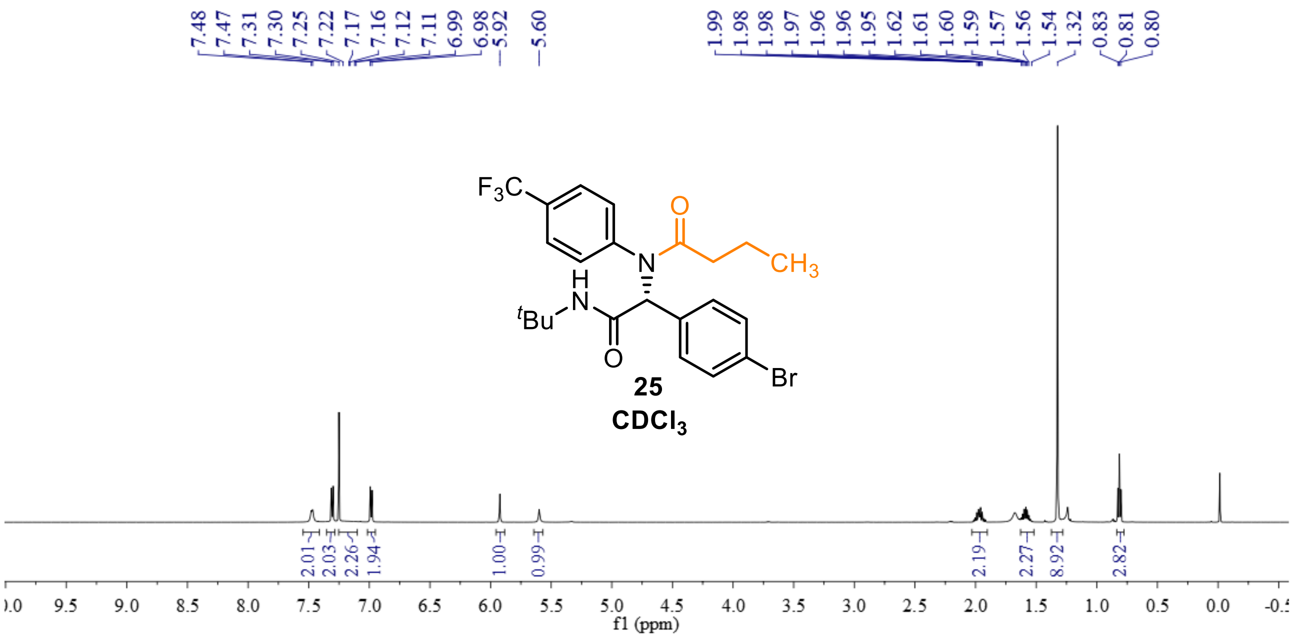

Supplementary Fig. 87. ^{1}H NMR spectrum of **25**. The sample has been recorded in 600 MHz, $CDCl_3$ at 25 °C.

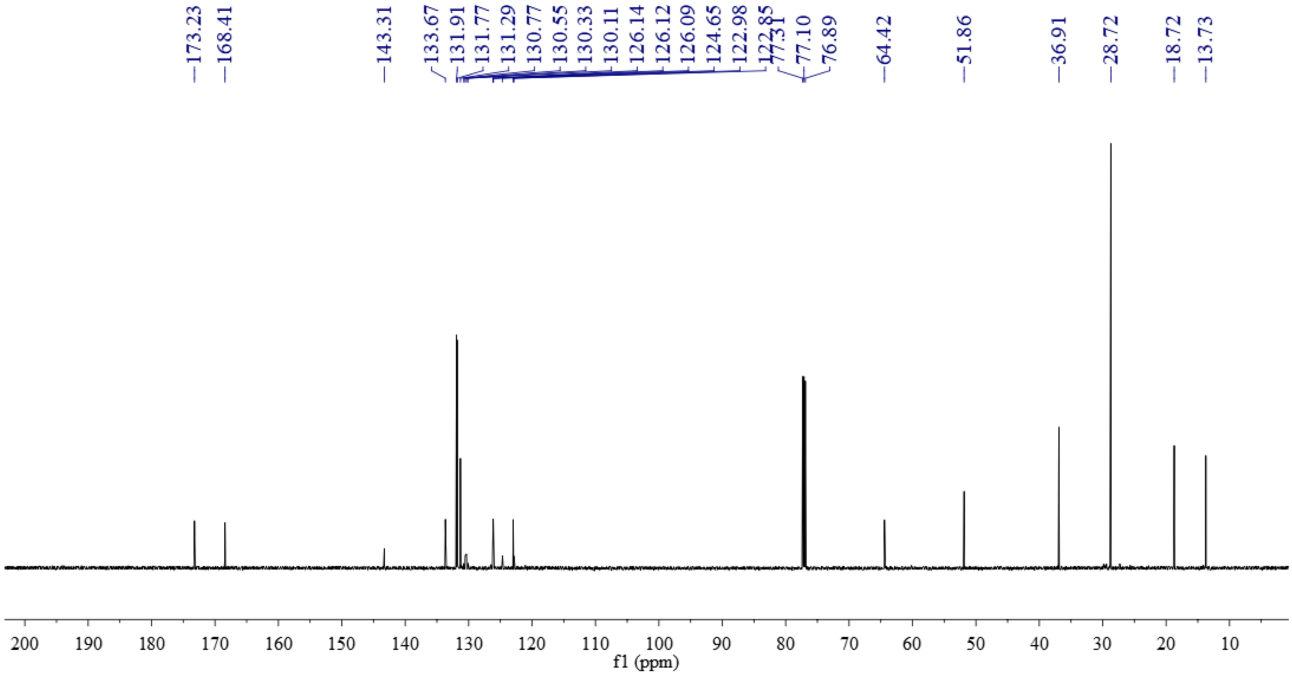

Supplementary Fig. 88.^{13}C NMR spectrum of **25**. The sample has been recorded in 151 MHz, $CDCl_3$ at 25 °C.

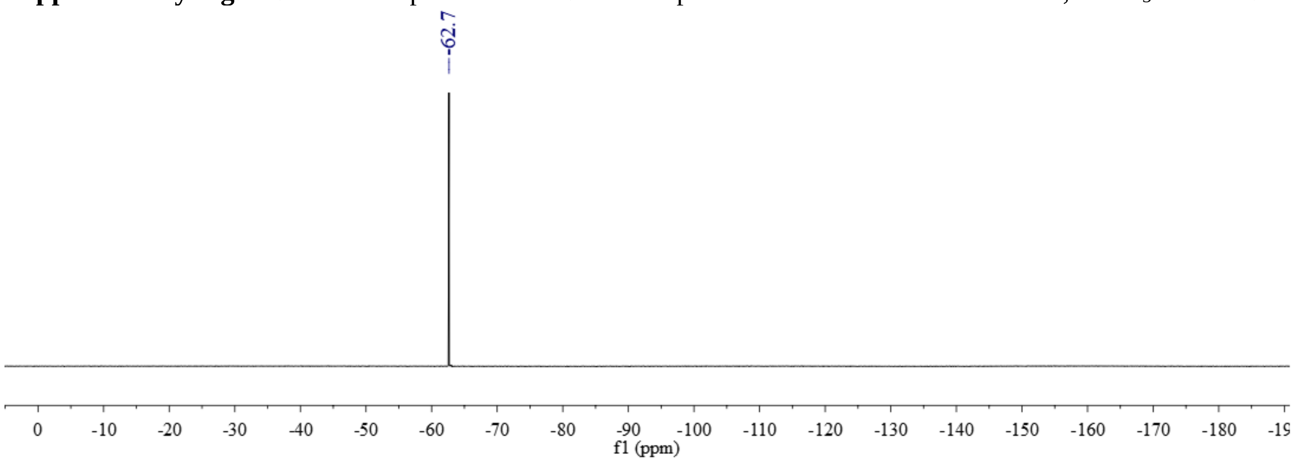

Supplementary Fig. 89. ^{31}F NMR spectrum of **25**. The sample has been recorded in 564 MHz, $CDCl_3$ at 25 °C.

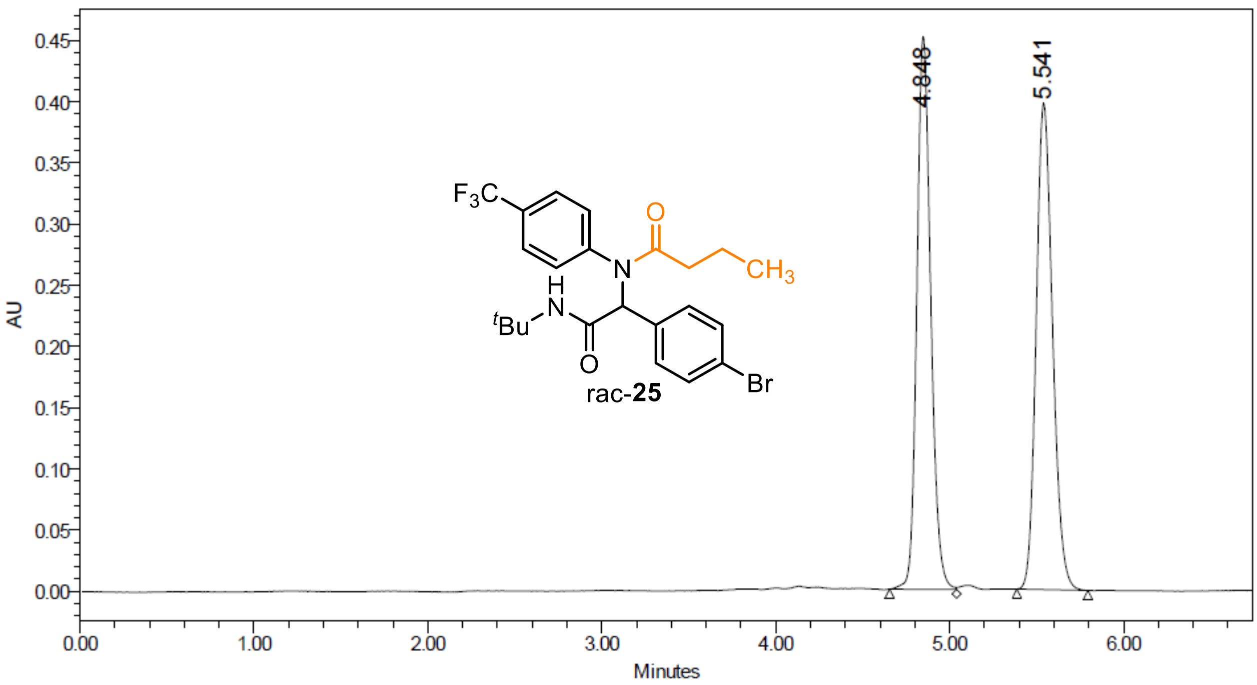

	RT (min)	Peak Type	Area (µV*sec)	% Area	Height (µV)	% Height	Integration Type	Points Across Peak	Start Time (min)	End Time (min)
1	4.848	Unknown	2501390	48.97	451595	53.21	BV	231	4.655	5.040
2	5.541	Unknown	2606165	51.03	397061	46.79	BB	245	5.387	5.795

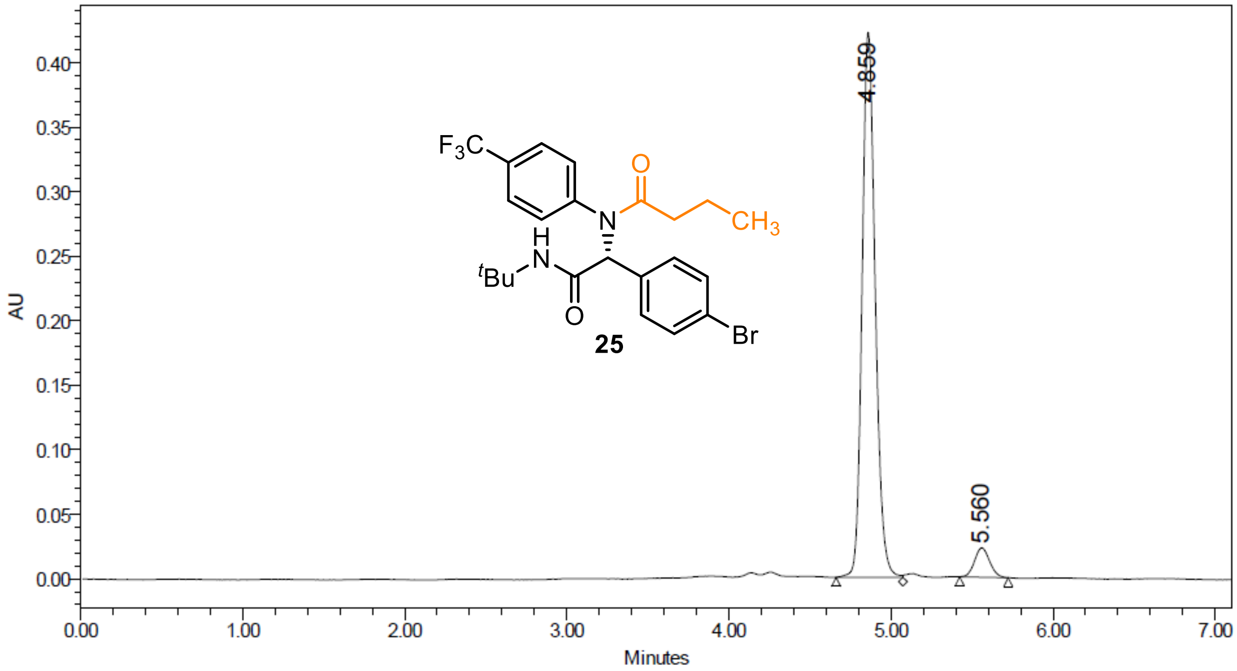

	RT (min)	Peak Type	Area (µV*sec)	% Area	Height (µV)	% Height	Integration Type	Points Across Peak	Start Time (min)	End Time (min)
1	4.859	Unknown	2371233	94.13	422260	94.88	BV	247	4.662	5.073
2	5.560	Unknown	147924	5.87	22771	5.12	BB	180	5.423	5.723

Supplementary Fig. 90. HPLC of product **25**.

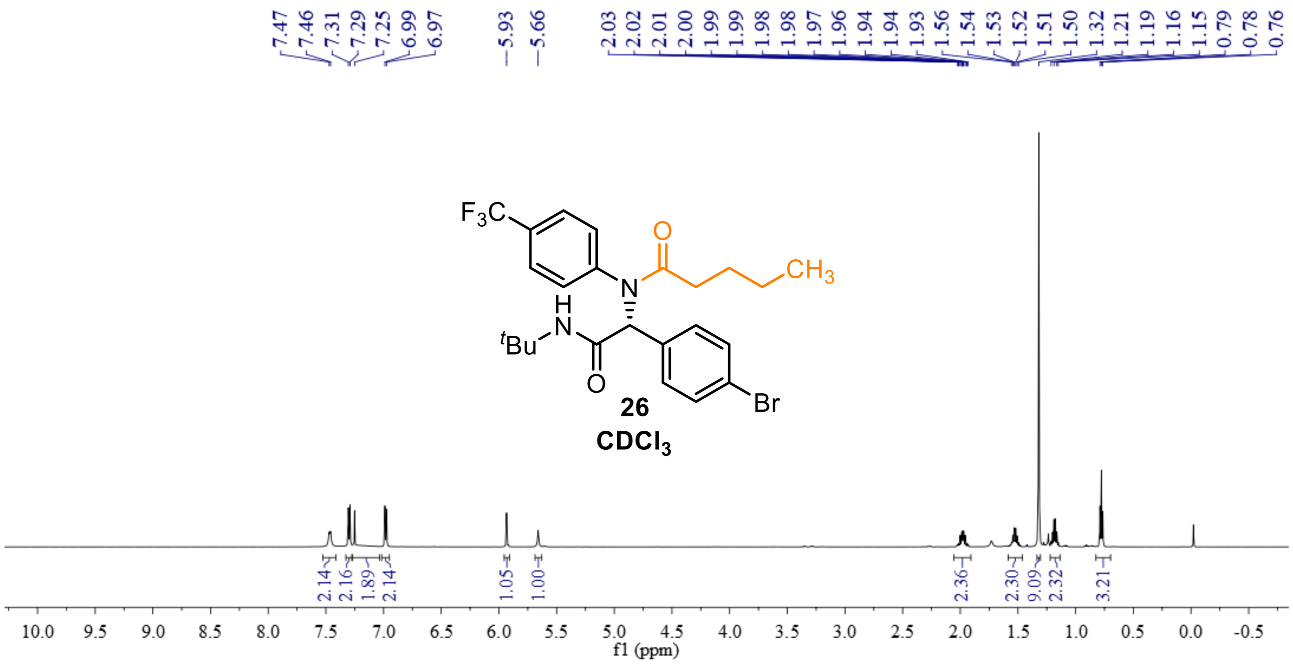

Supplementary Fig. 91. ^{1}H NMR spectrum of **26**. The sample has been recorded in 600 MHz, $CDCl_3$ at 25 °C.

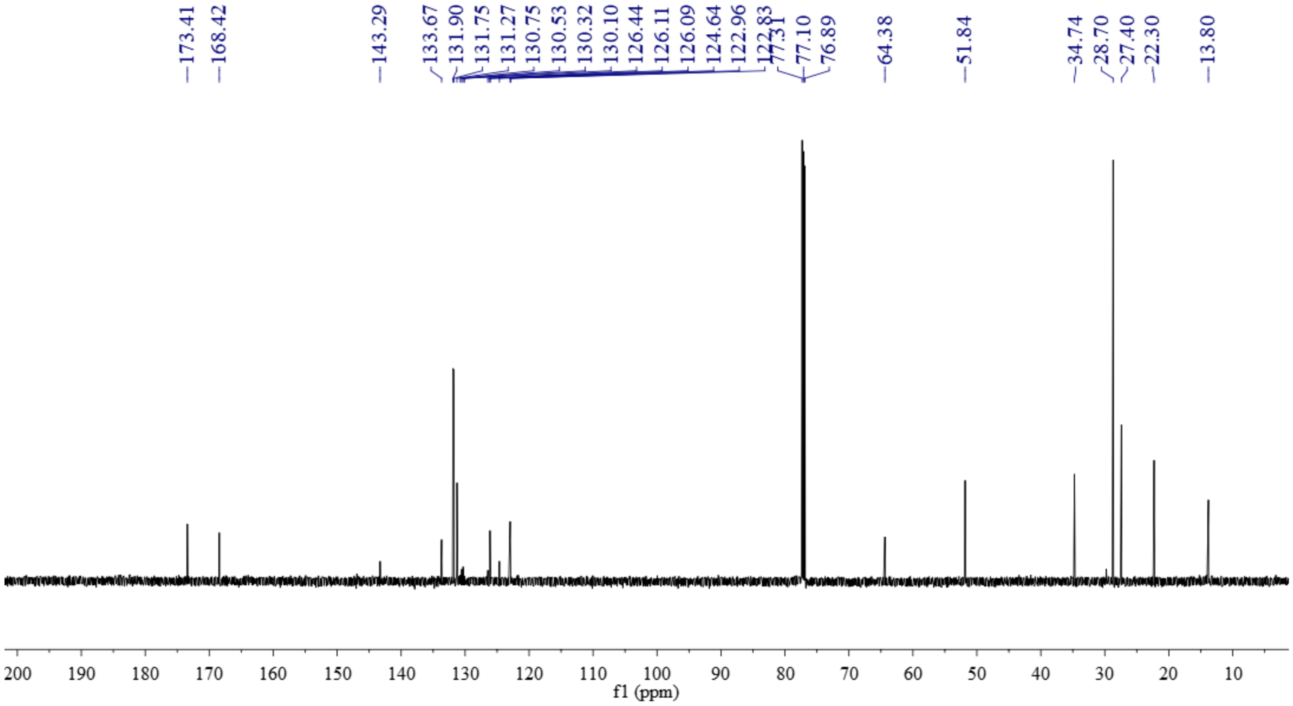

Supplementary Fig. 92.^{13}C NMR spectrum of **26**. The sample has been recorded in 151 MHz, $CDCl_3$ at 25 °C.

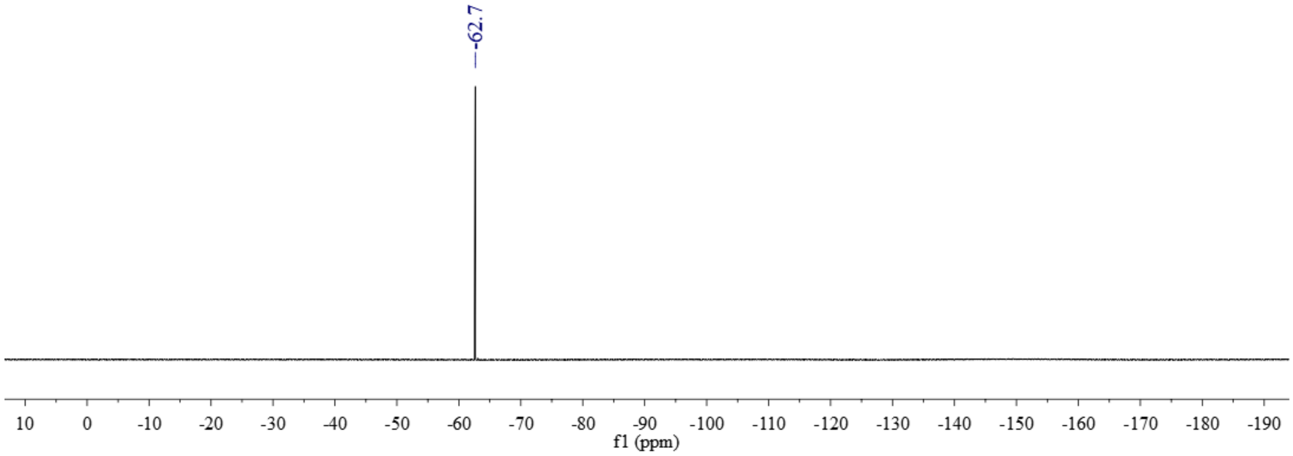

Supplementary Fig. 93. ^{31}F NMR spectrum of **26**. The sample has been recorded in 564 MHz, $CDCl_3$ at 25 °C.

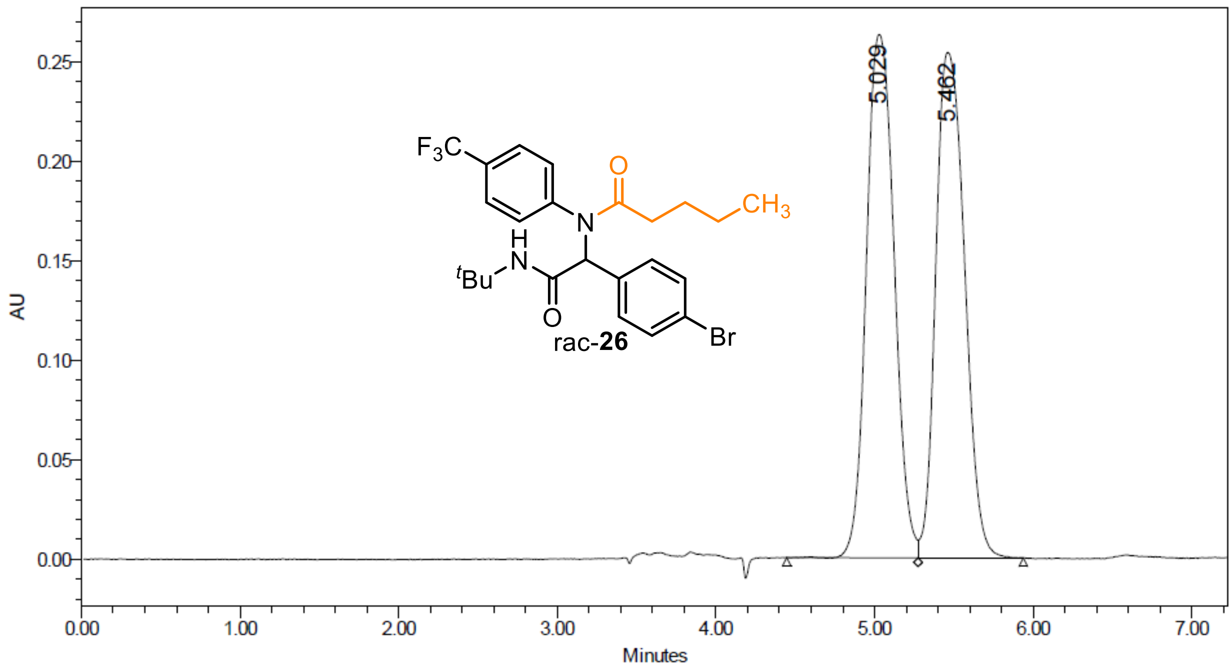

	RT (min)	Peak Type	Area (µV*sec)	% Area	Height (µV)	% Height	Integration Type	Points Across Peak	Start Time (min)	End Time (min)
1	5.029	Unknown	3212456	50.06	262883	50.86	bV	497	4.447	5.275
2	5.462	Unknown	3205235	49.94	253986	49.14	VB	398	5.275	5.938

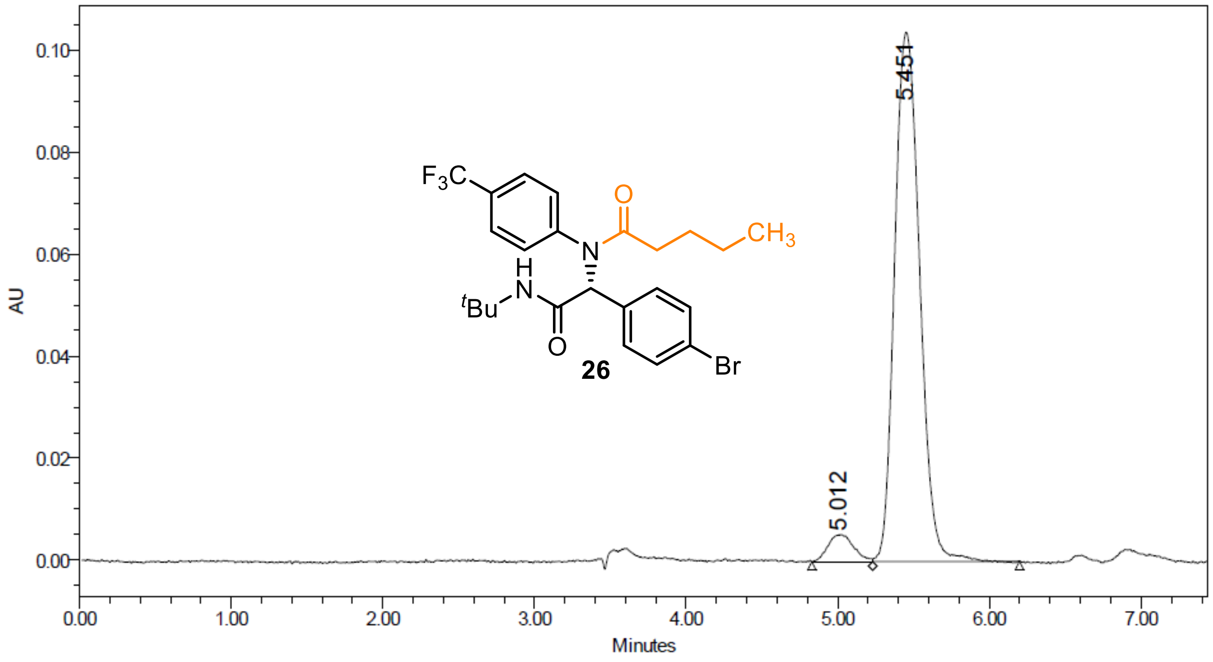

	RT (min)	Peak Type	Area (µV*sec)	% Area	Height (µV)	% Height	Integration Type	Points Across Peak	Start Time (min)	End Time (min)
1	5.012	Unknown	62620	5.00	5269	4.83	BV	241	4.828	5.230
2	5.451	Unknown	1190873	95.00	103797	95.17	Vb	581	5.230	6.198

Supplementary Fig. 94. HPLC of product **26**.

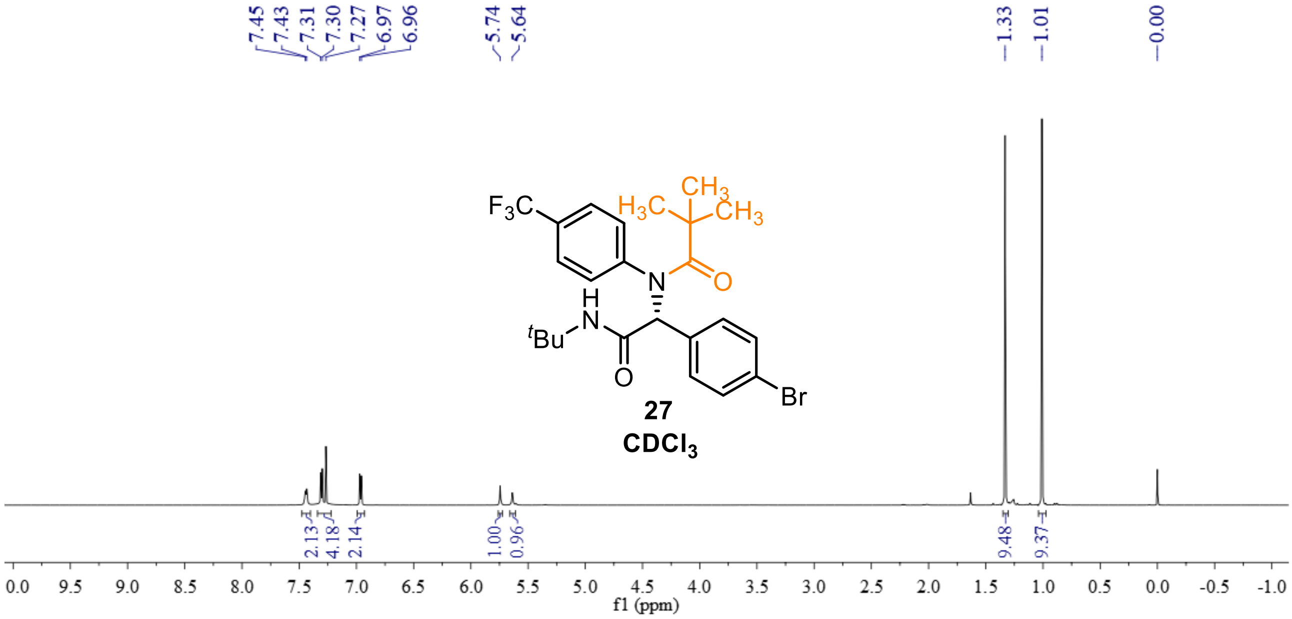

Supplementary Fig. 95. ^{1}H NMR spectrum of **27**. The sample has been recorded in 600 MHz, $CDCl_3$ at 25 °C.

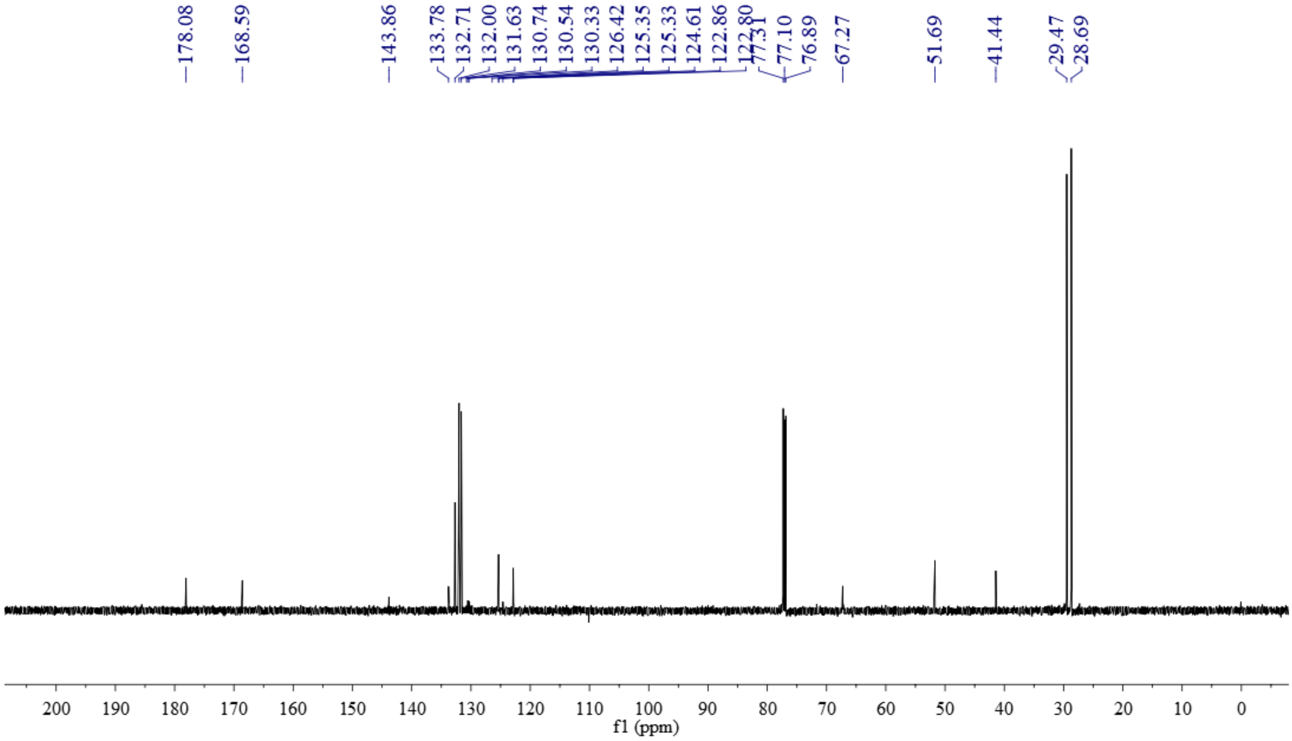

Supplementary Fig. 96. ^{13}C NMR spectrum of **27**. The sample has been recorded in 151 MHz, $CDCl_3$ at 25 °C.

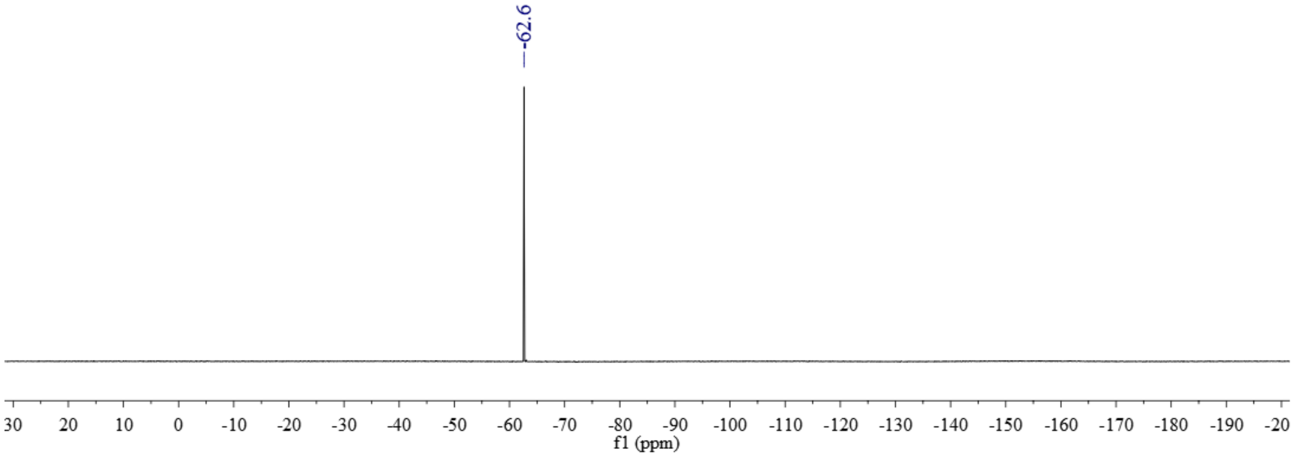

Supplementary Fig. 97. ^{31}F NMR spectrum of **27**. The sample has been recorded in 564 MHz, $CDCl_3$ at 25 °C.

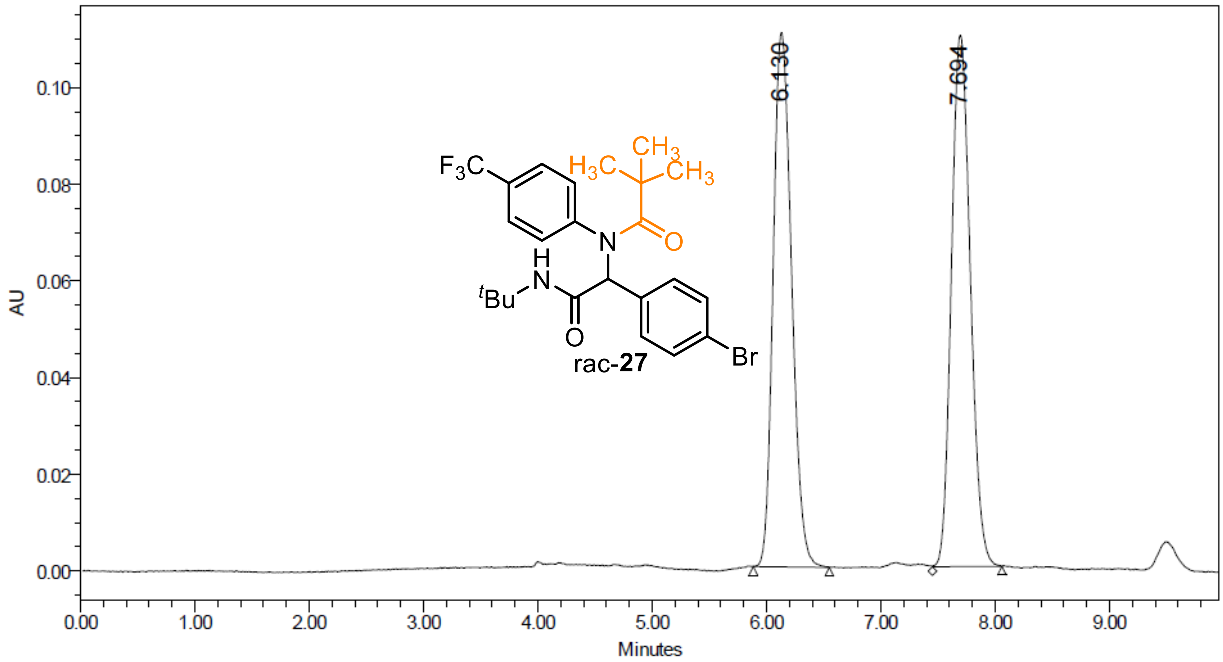

	RT (min)	Peak Type	Area (μV*sec)	% Area	Height (μV)	% Height	Integration Type	Points Across Peak	Start Time (min)	End Time (min)
1	6.130	Unknown	1237328	49.47	110552	50.16	BB	400	5.883	6.550
2	7.694	Unknown	1264079	50.53	109864	49.84	VB	364	7.452	8.058

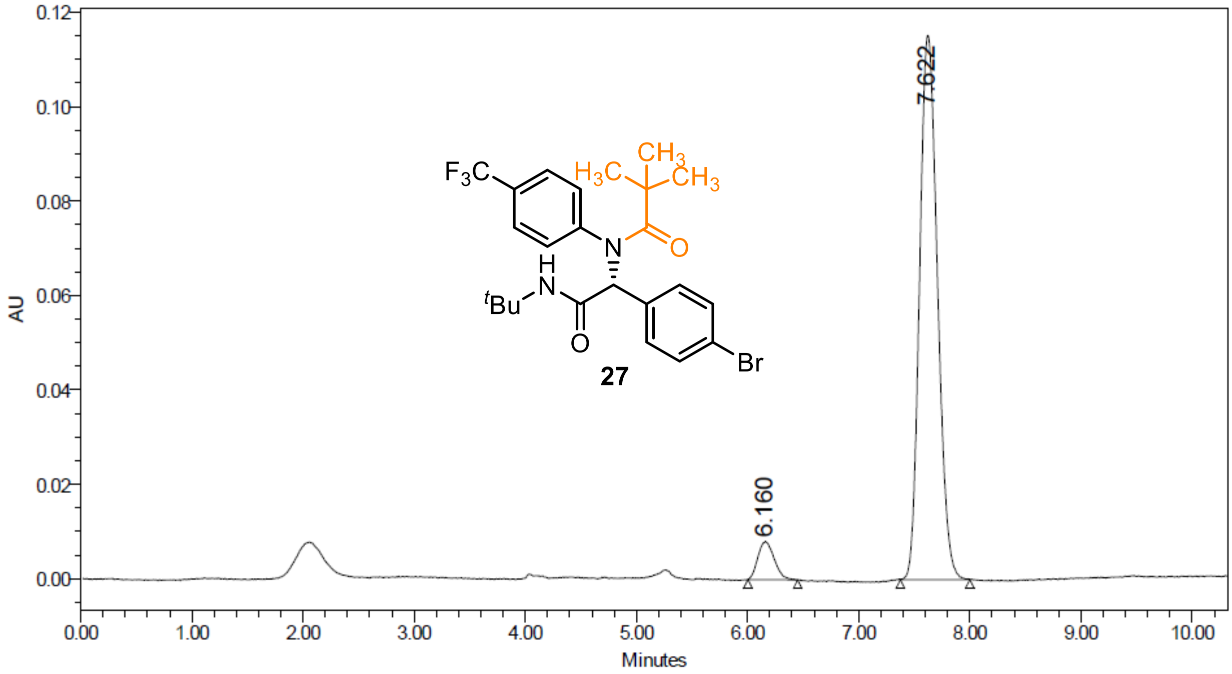

	RT (min)	Peak Type	Area (μV*sec)	% Area	Height (μV)	% Height	Integration Type	Points Across Peak	Start Time (min)	End Time (min)
1	6.160	Unknown	82492	6.03	8046	6.52	BB	269	6.003	6.452
2	7.622	Unknown	1286675	93.97	115302	93.48	BB	375	7.375	8.000

Supplementary Fig. 98. HPLC of product **27**.

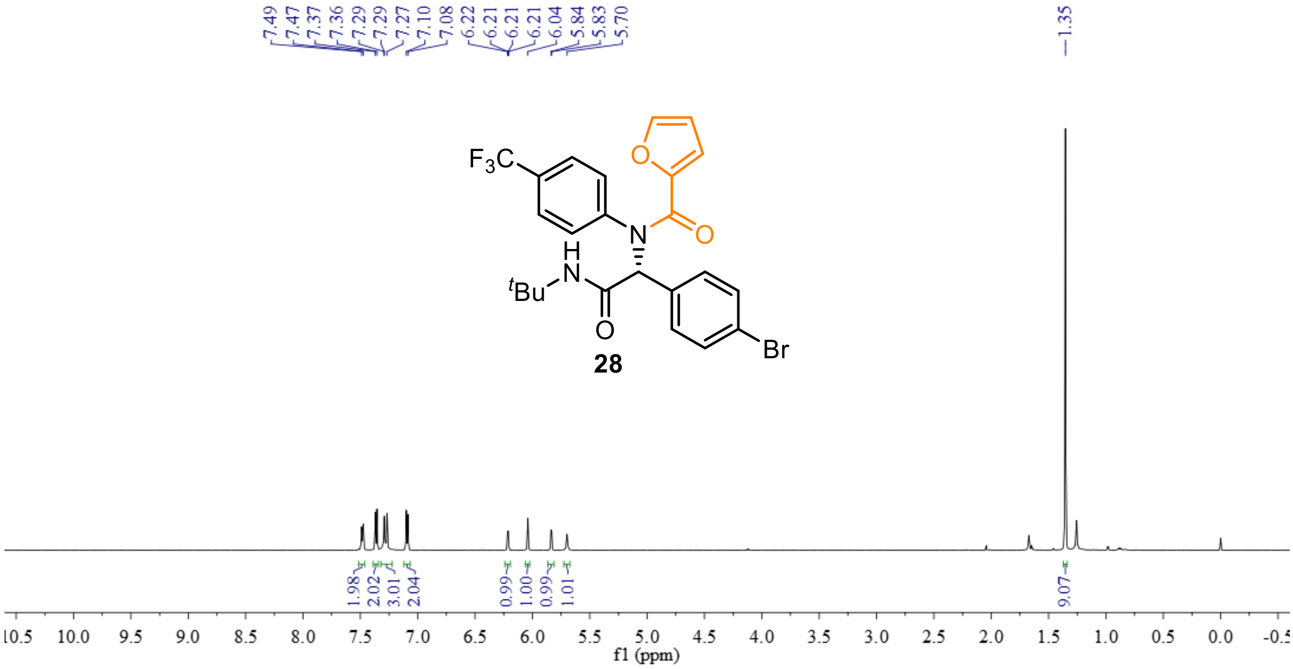

Supplementary Fig. 99. ^{1}H NMR spectrum of **28**. The sample has been recorded in 600 MHz, $CDCl_3$ at 25 °C.

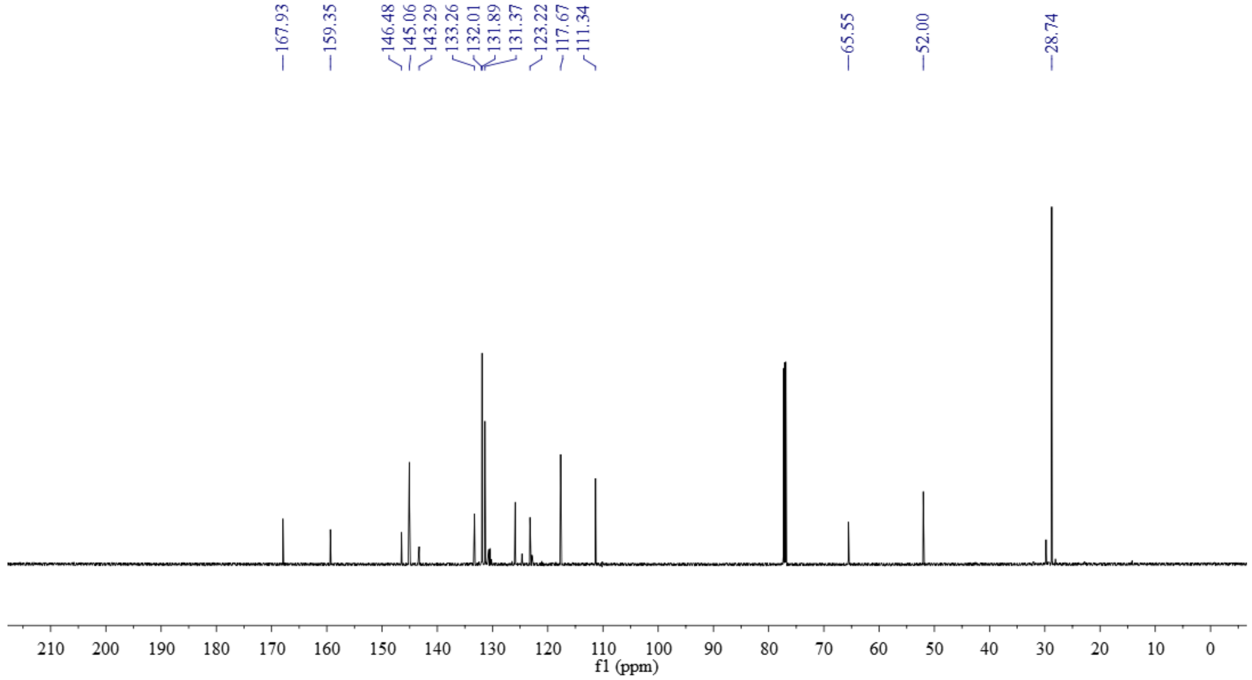

Supplementary Fig. 100. ^{13}C NMR spectrum of **28**. The sample has been recorded in 151 MHz, $CDCl_3$ at 25 °C.

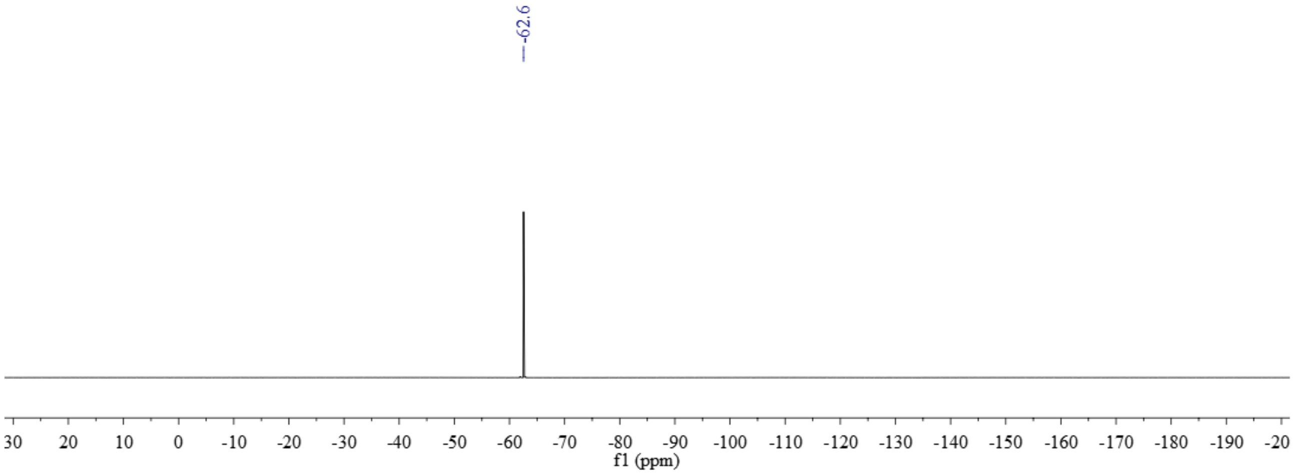

Supplementary Fig. 101. ^{31}F NMR spectrum of **28**. The sample has been recorded in 564 MHz, $CDCl_3$ at 25 °C.

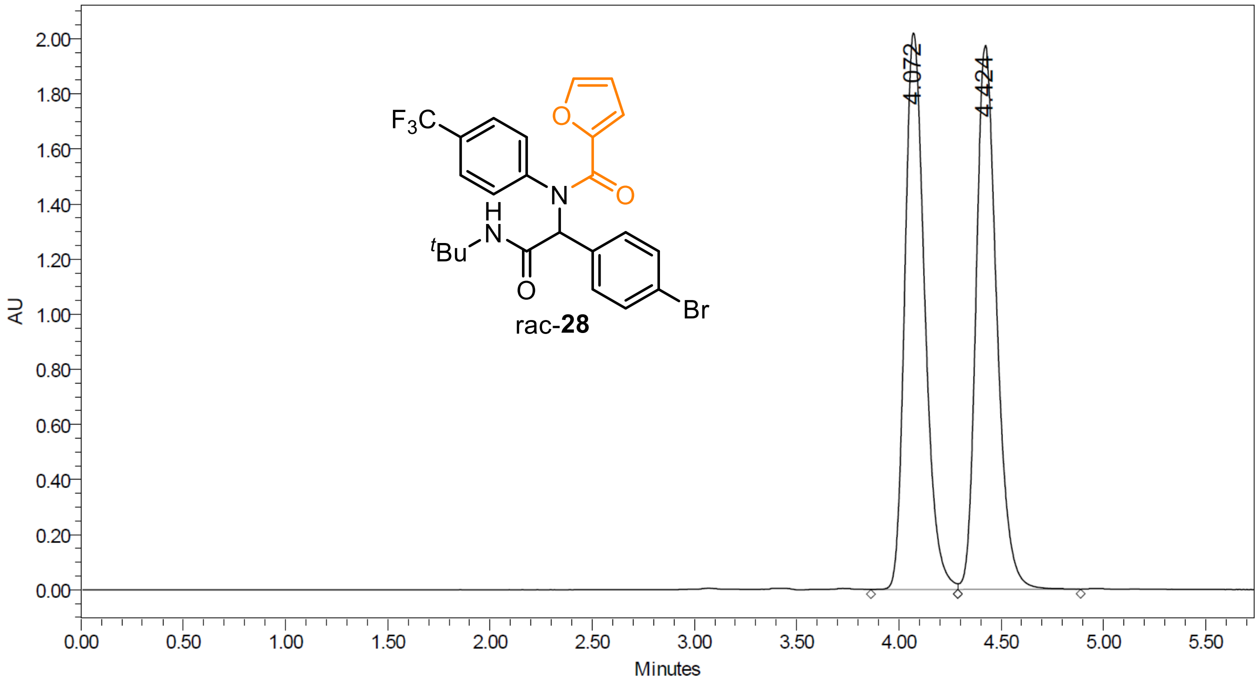

	RT (min)	Peak Type	Area (µV*sec)	% Area	Height (µV)	% Height	Integration Type	Points Across Peak	Start Time (min)	End Time (min)
1	4.072	Unknown	13679356	49.76	2020462	50.57	VV	255	3.863	4.288
2	4.424	Unknown	13811372	50.24	1975294	49.43	VV	360	4.288	4.888

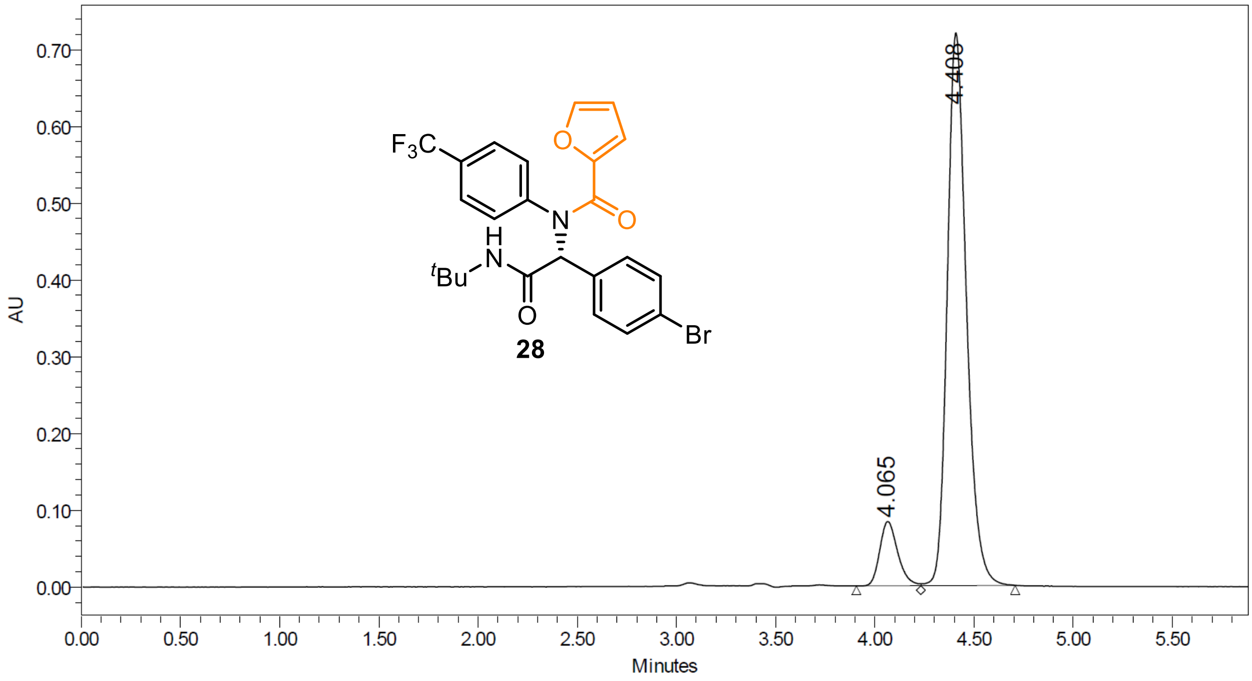

	RT (min)	Peak Type	Area (µV*sec)	% Area	Height (µV)	% Height	Integration Type	Points Across Peak	Start Time (min)	End Time (min)
1	4.065	Unknown	528023	9.87	83794	10.42	bV	195	3.907	4.232
2	4.408	Unknown	4819817	90.13	720122	89.58	Vb	285	4.232	4.707

Supplementary Fig. 102. HPLC of product **28**.

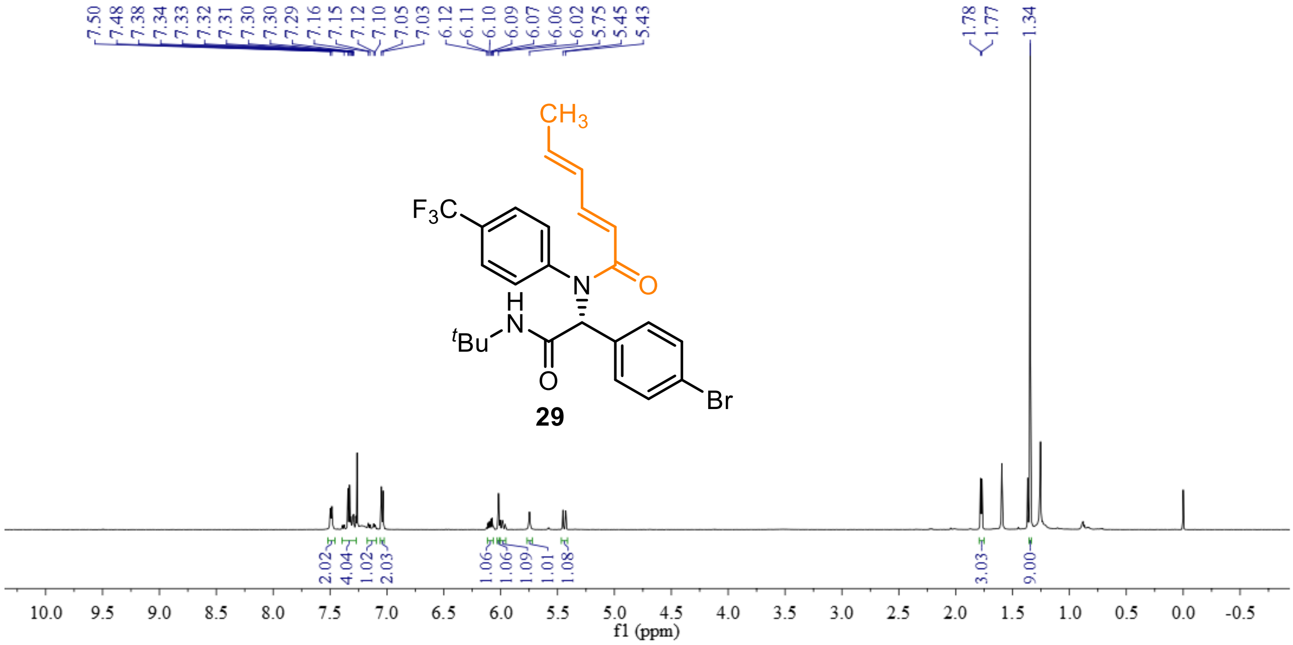

Supplementary Fig. 103. ^{1}H NMR spectrum of **29**. The sample has been recorded in 600 MHz, $CDCl_3$ at 25 °C.

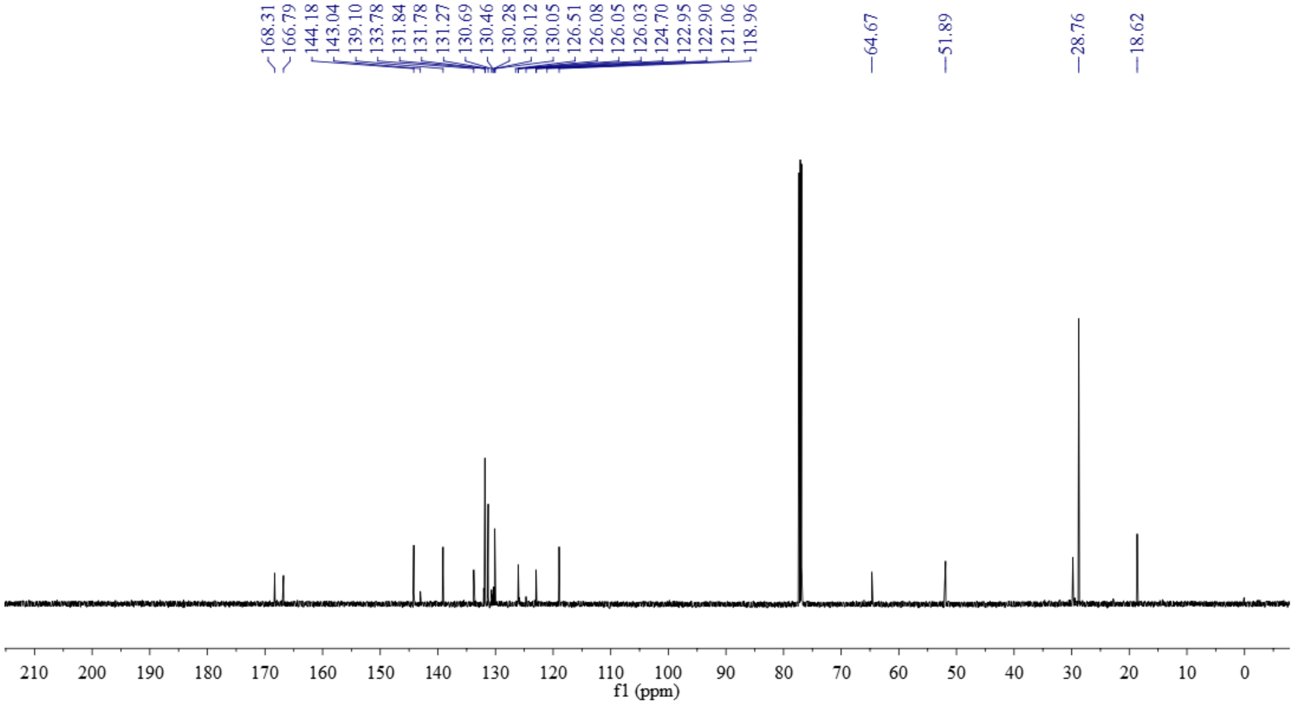

Supplementary Fig. 104. ^{13}C NMR spectrum of **29**. The sample has been recorded in 151 MHz, $CDCl_3$ at 25 °C.

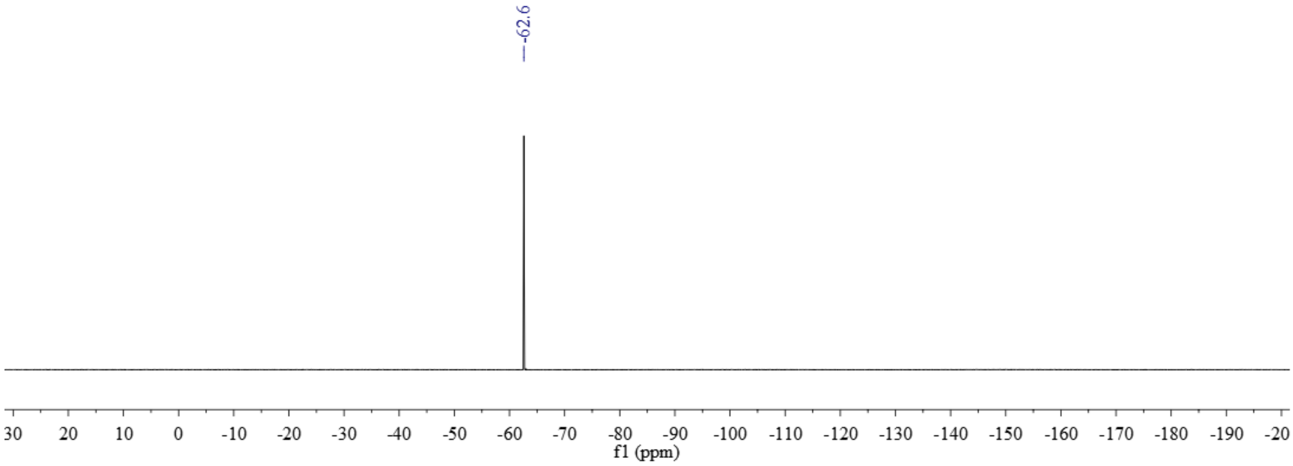

Supplementary Fig. 105. ^{31}F NMR spectrum of **29**. The sample has been recorded in 564 MHz, $CDCl_3$ at 25 °C.

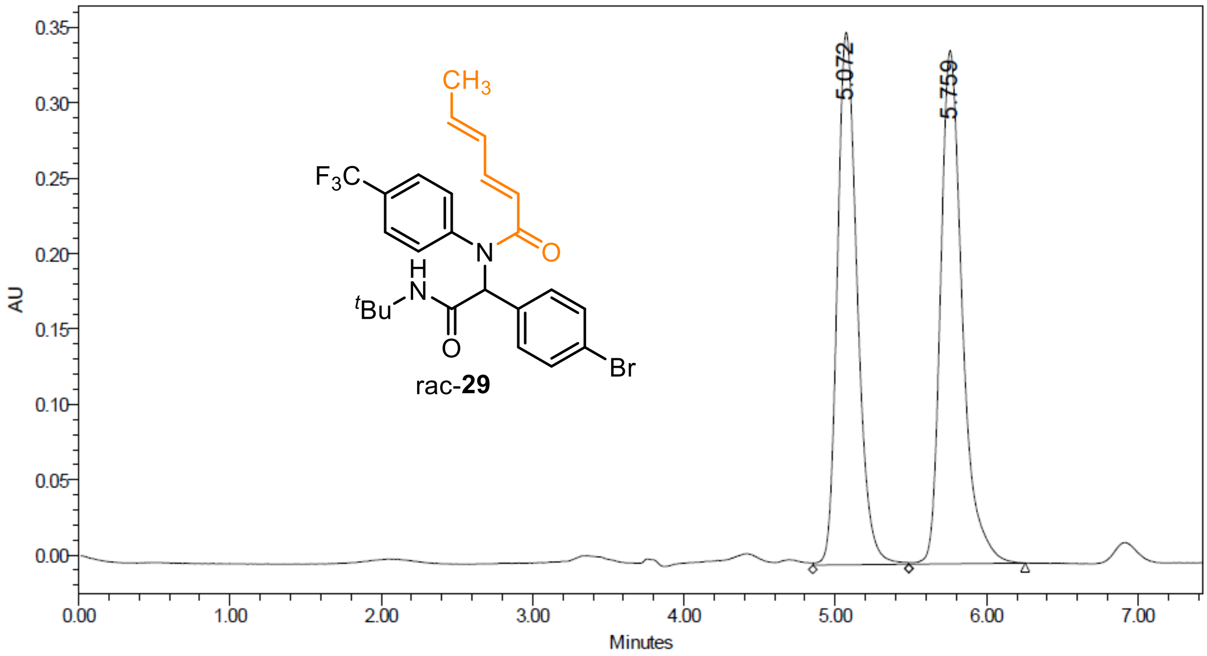

rac-**29**

	RT (min)	Peak Type	Area (µV*sec)	% Area	Height (µV)	% Height	Integration Type	Points Across Peak	Start Time (min)	End Time (min)
1	5.072	Unknown	3223229	48.74	353187	50.94	VV	381	4.852	5.487
2	5.759	Unknown	3389835	51.26	340202	49.06	VB	461	5.487	6.255

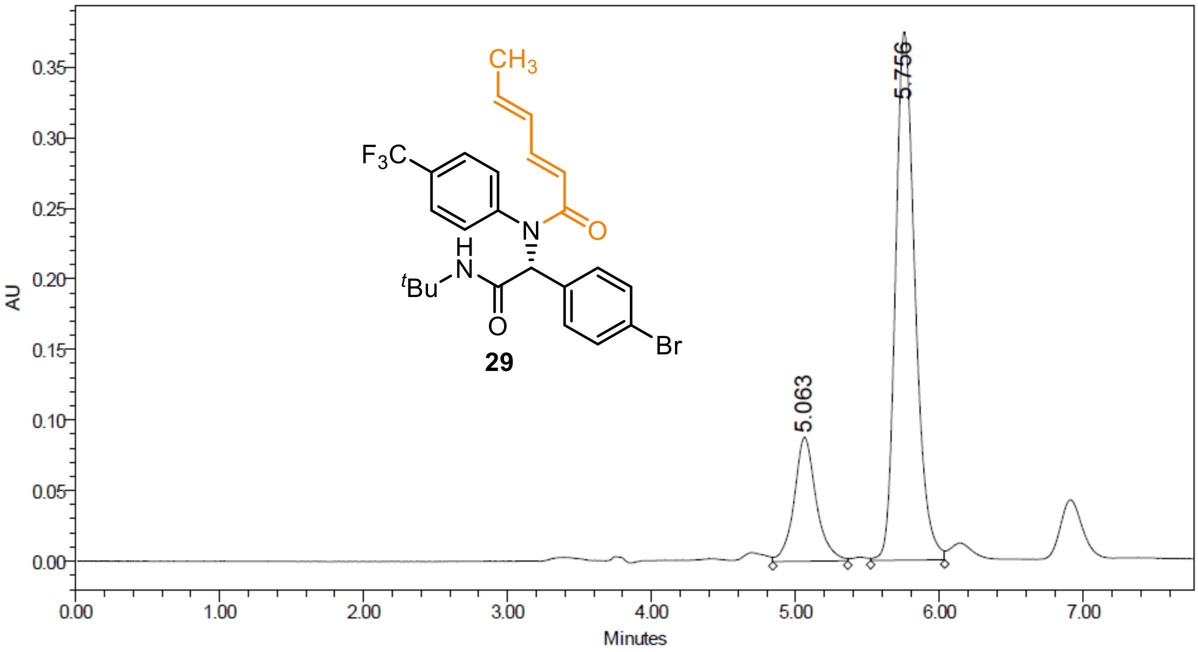

29

	RT (min)	Peak Type	Area (µV*sec)	% Area	Height (µV)	% Height	Integration Type	Points Across Peak	Start Time (min)	End Time (min)
1	5.063	Unknown	932736	20.88	87791	18.99	VV	312	4.843	5.363
2	5.756	Unknown	3534522	79.12	374418	81.01	VV	309	5.522	6.037

Supplementary Fig. 106. HPLC of product **29**.

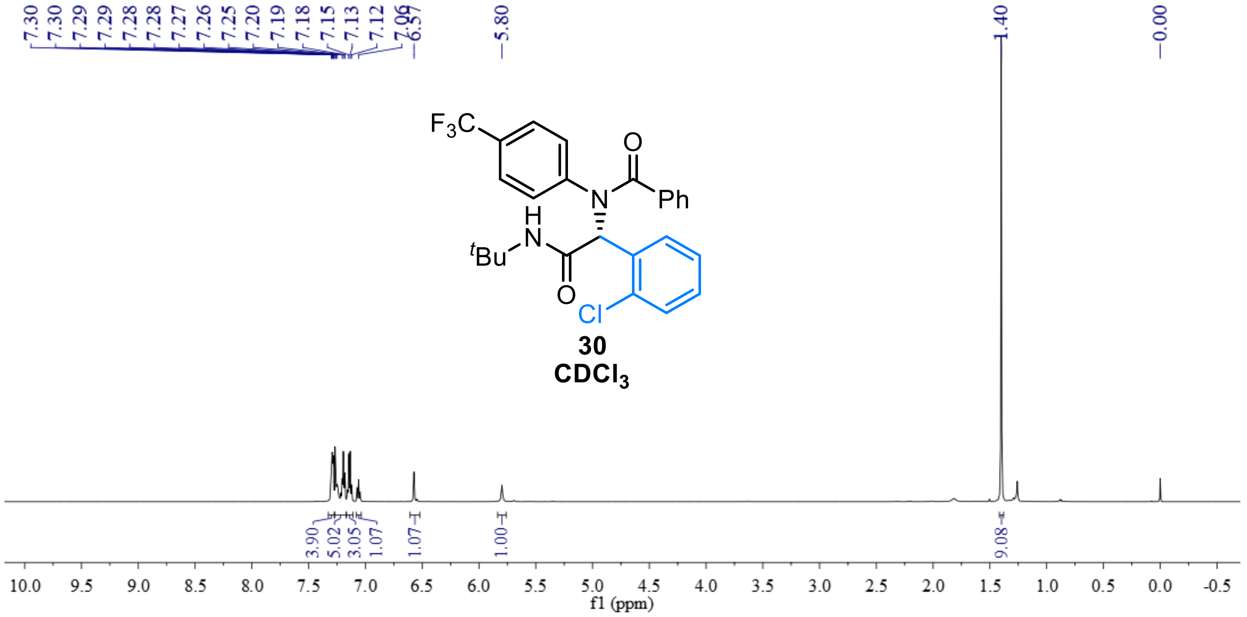

Supplementary Fig. 107. ^{1}H NMR spectrum of **30**. The sample has been recorded in 600 MHz, $CDCl_3$ at 25 °C.

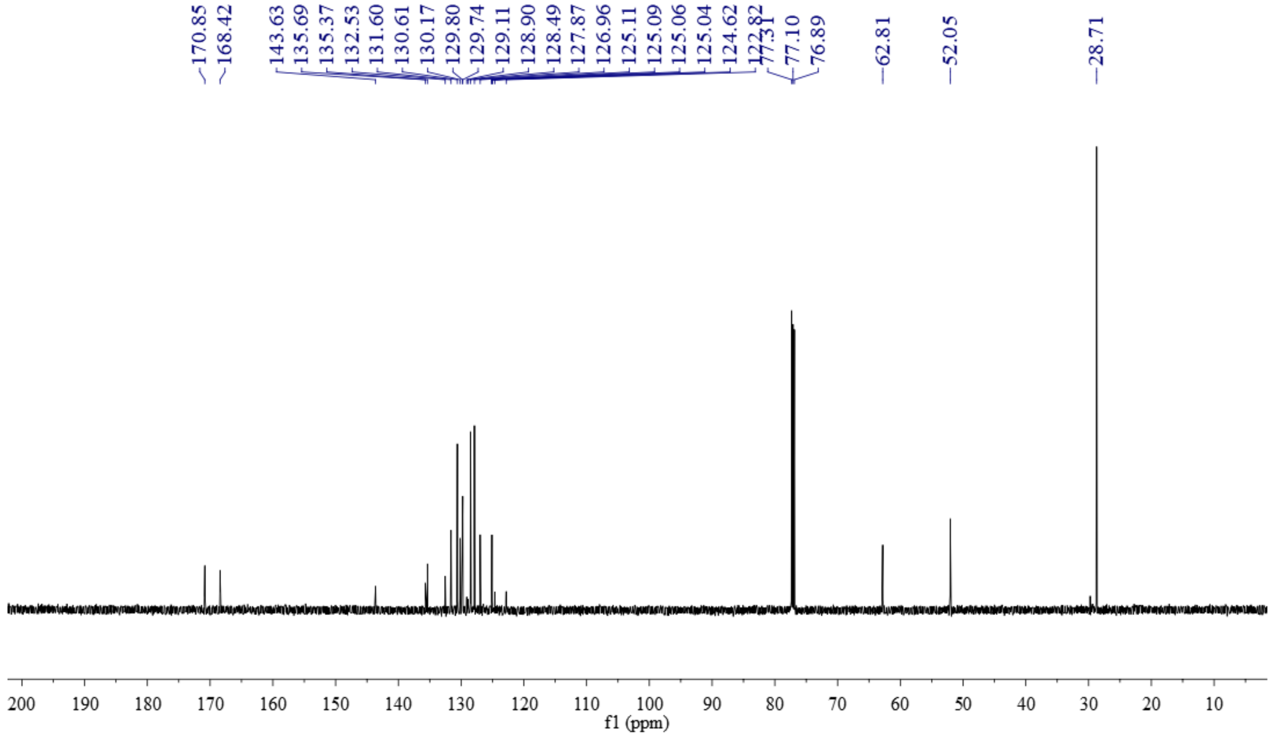

Supplementary Fig. 108. ^{13}C NMR spectrum of **30**. The sample has been recorded in 151 MHz, $CDCl_3$ at 25 °C.

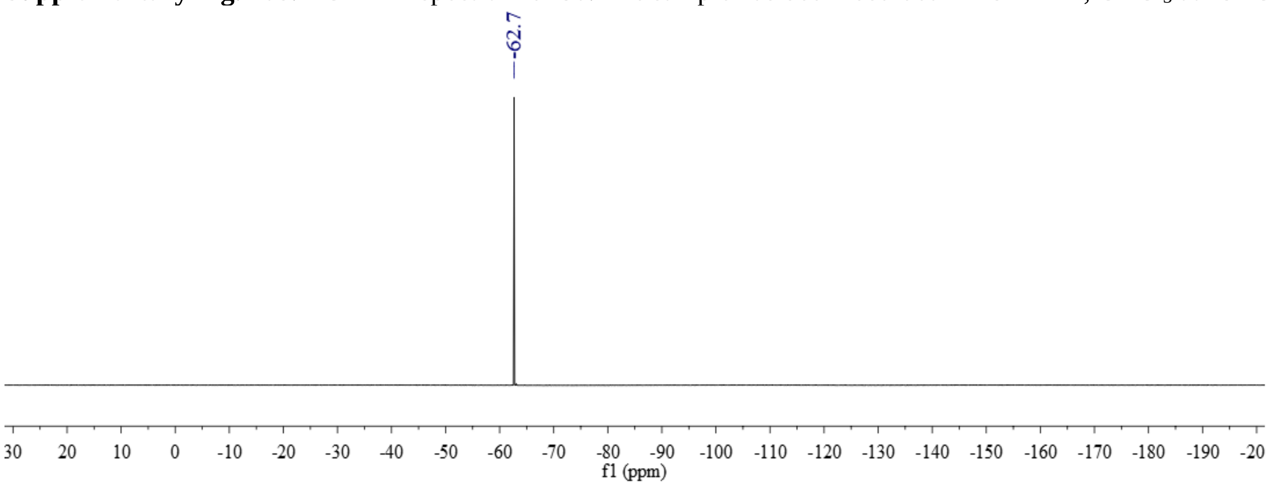

Supplementary Fig. 109. [31]F NMR spectrum of **30**. The sample has been recorded in 564 MHz, $CDCl_3$ at 25 °C.

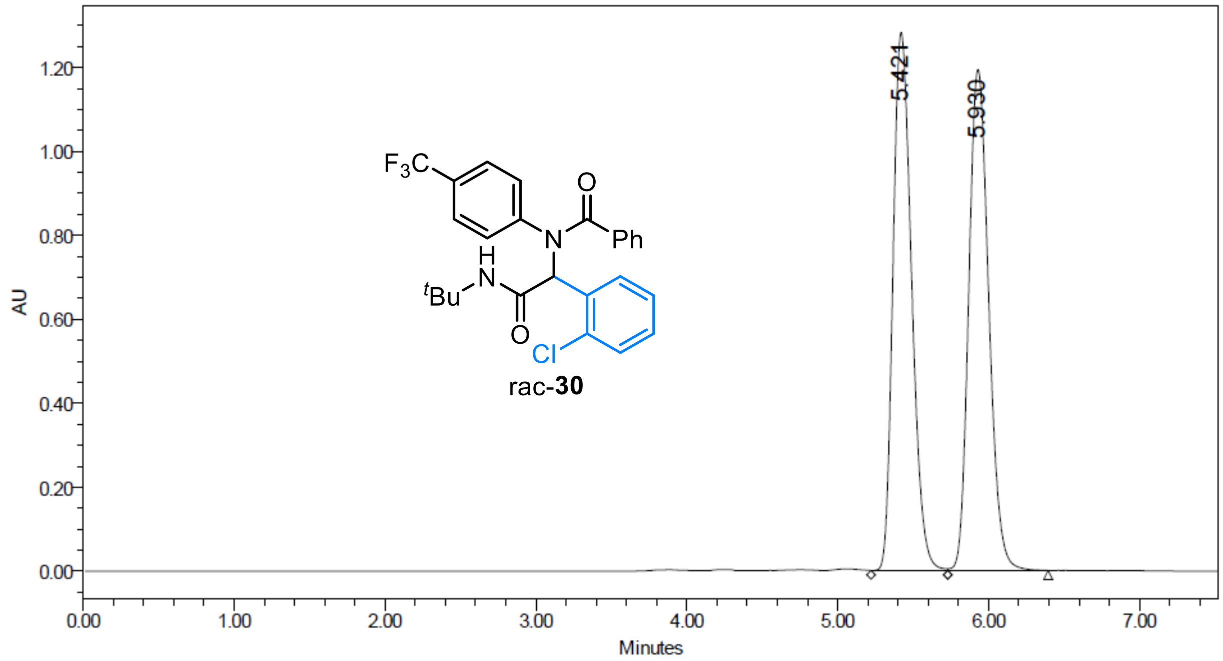

	RT (min)	Peak Type	Area (μV*sec)	% Area	Height (μV)	% Height	Integration Type	Points Across Peak	Start Time (min)	End Time (min)
1	5.421	Unknown	10960154	49.98	1281668	51.79	VV	305	5.222	5.730
2	5.930	Unknown	10967068	50.02	1193054	48.21	VB	399	5.730	6.395

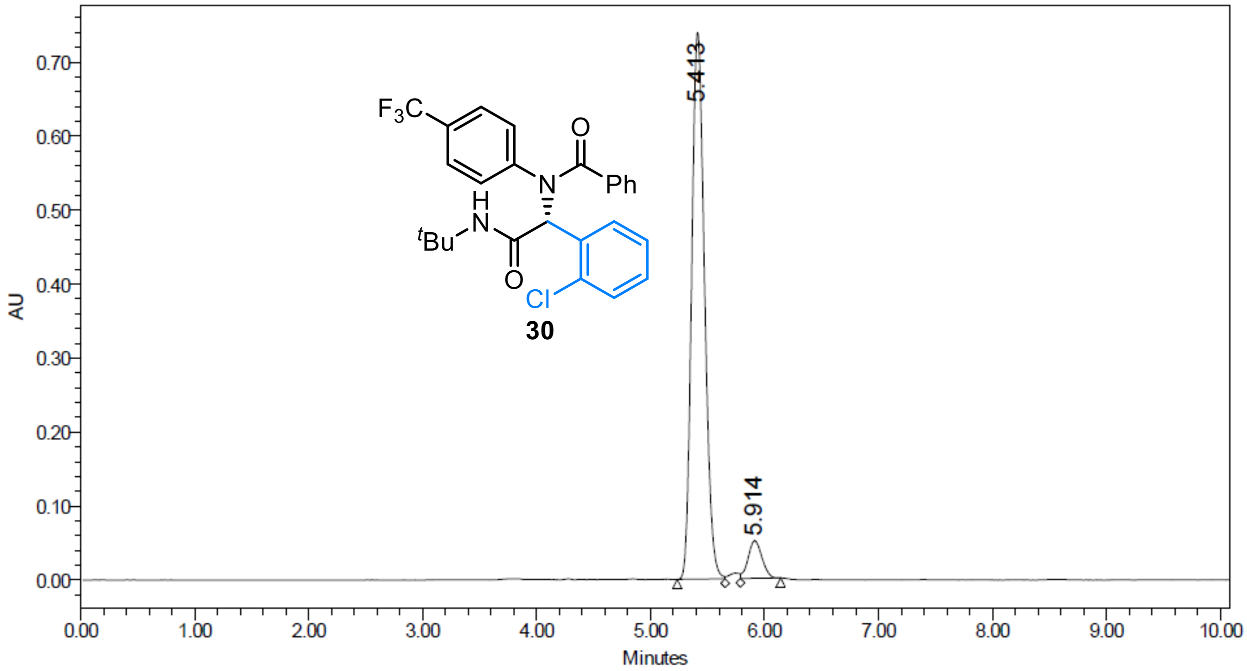

	RT (min)	Peak Type	Area (μV*sec)	% Area	Height (μV)	% Height	Integration Type	Points Across Peak	Start Time (min)	End Time (min)
1	5.413	Unknown	5784169	93.13	739025	93.57	BV	253	5.233	5.655
2	5.914	Unknown	426904	6.87	50814	6.43	VB	212	5.788	6.142

Supplementary Fig. 110. HPLC of product **30**.

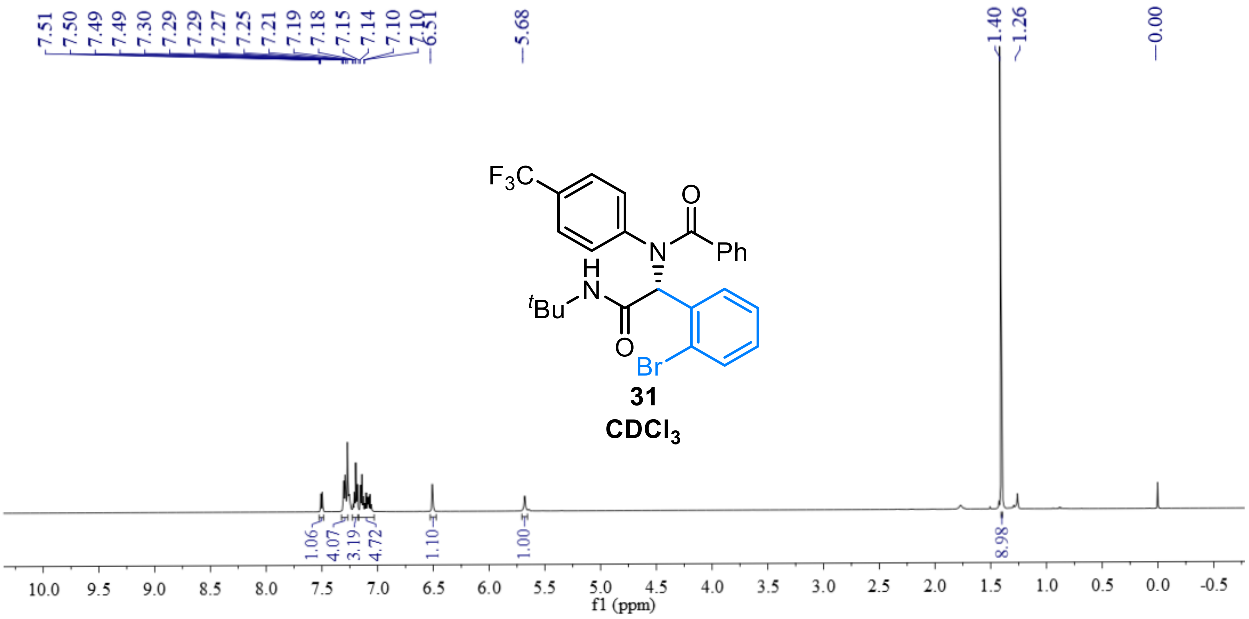

Supplementary Fig. 111. ^{1}H NMR spectrum of **31**. The sample has been recorded in 600 MHz, $CDCl_3$ at 25 °C.

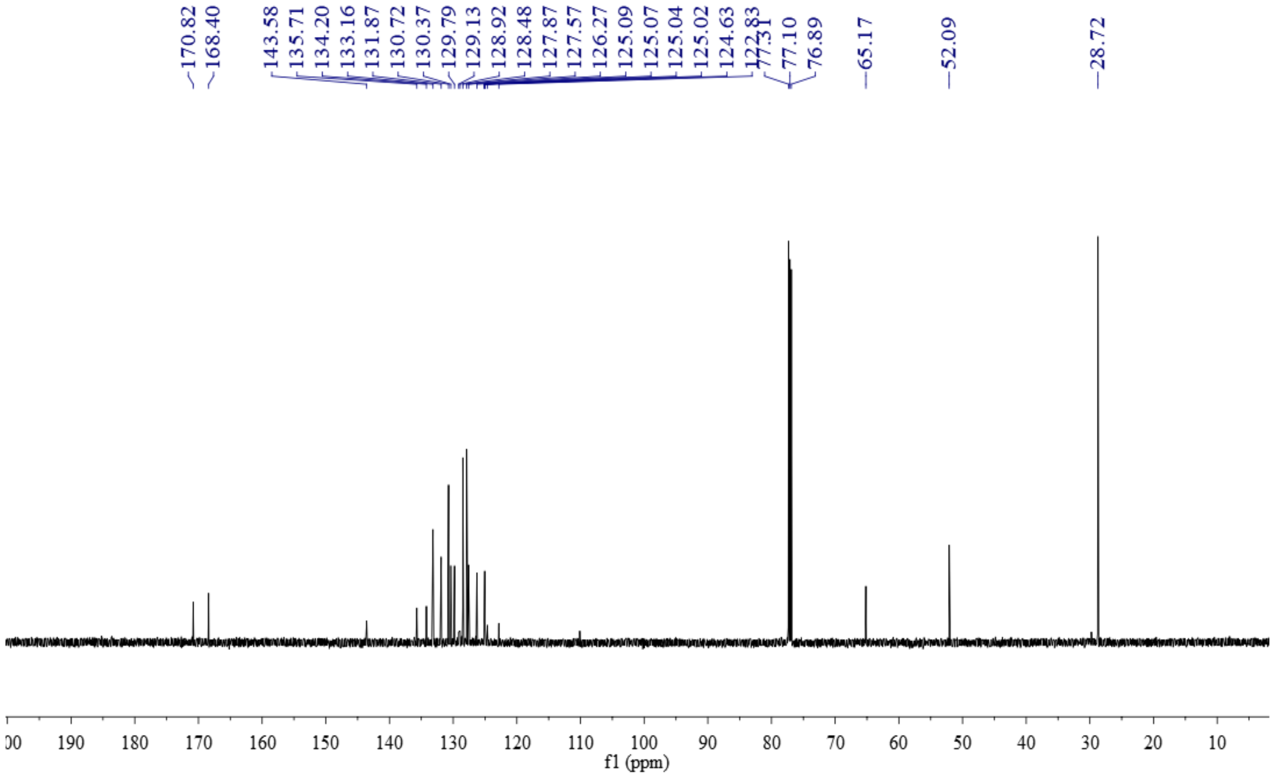

Supplementary Fig. 112. ^{13}C NMR spectrum of **31**. The sample has been recorded in 151 MHz, $CDCl_3$ at 25 °C.

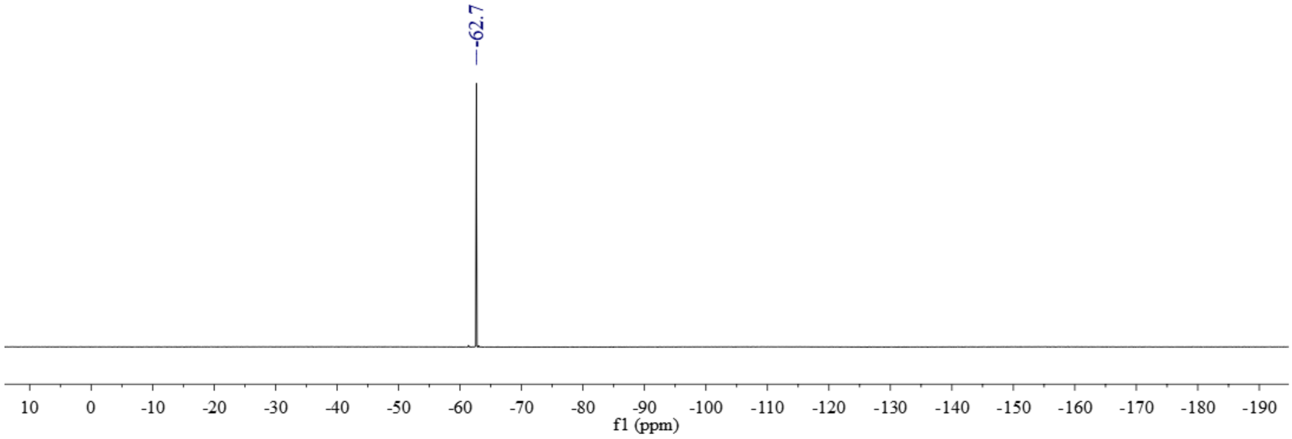

Supplementary Fig. 113. [31]F NMR spectrum of **31**. The sample has been recorded in 564 MHz, $CDCl_3$ at 25 °C.

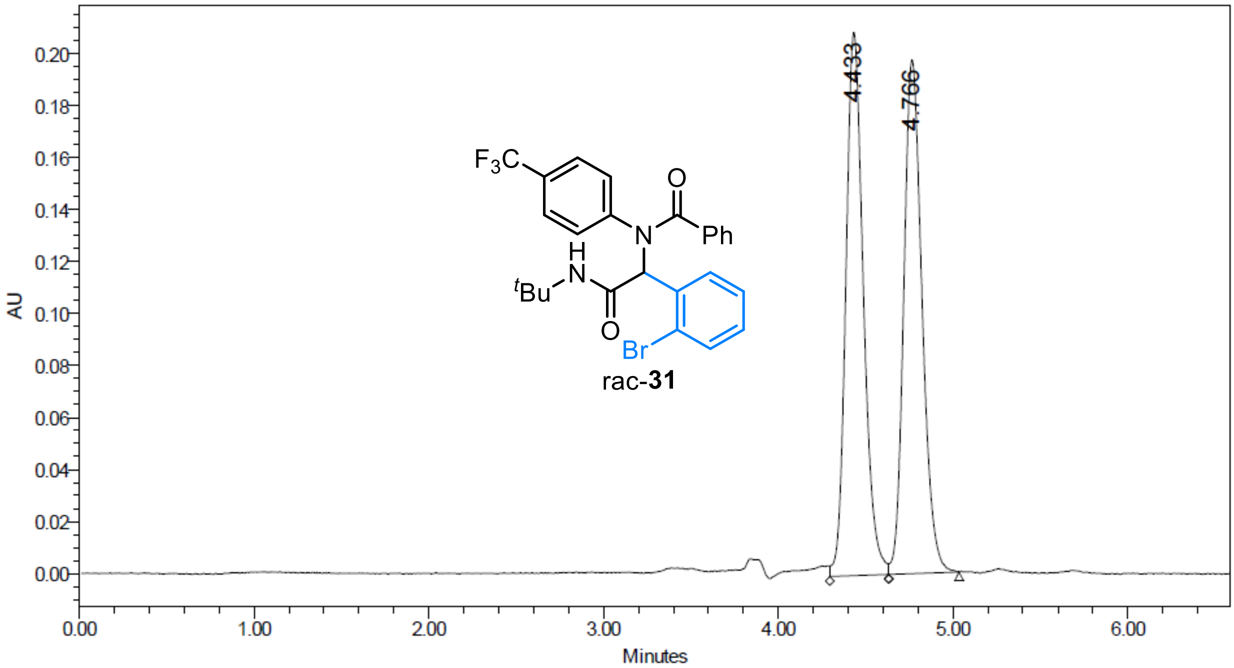

	RT (min)	Peak Type	Area (µV*sec)	% Area	Height (µV)	% Height	Integration Type	Points Across Peak	Start Time (min)	End Time (min)
1	4.433	Unknown	1458983	50.43	208930	51.40	VV	202	4.295	4.632
2	4.766	Unknown	1433850	49.57	197528	48.60	VB	242	4.632	5.035

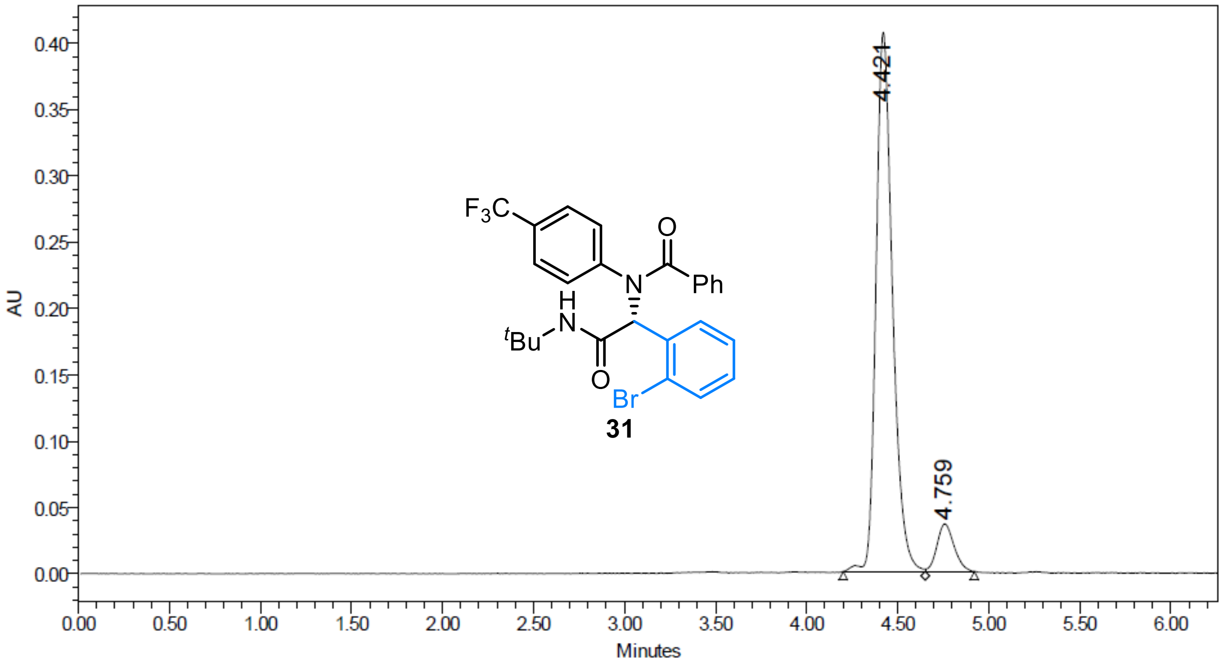

	RT (min)	Peak Type	Area (µV*sec)	% Area	Height (µV)	% Height	Integration Type	Points Across Peak	Start Time (min)	End Time (min)
1	4.421	Unknown	2560868	91.65	406536	91.91	bV	270	4.202	4.652
2	4.759	Unknown	233244	8.35	35792	8.09	Vb	162	4.652	4.922

Supplementary Fig. 114. HPLC of product **31**.

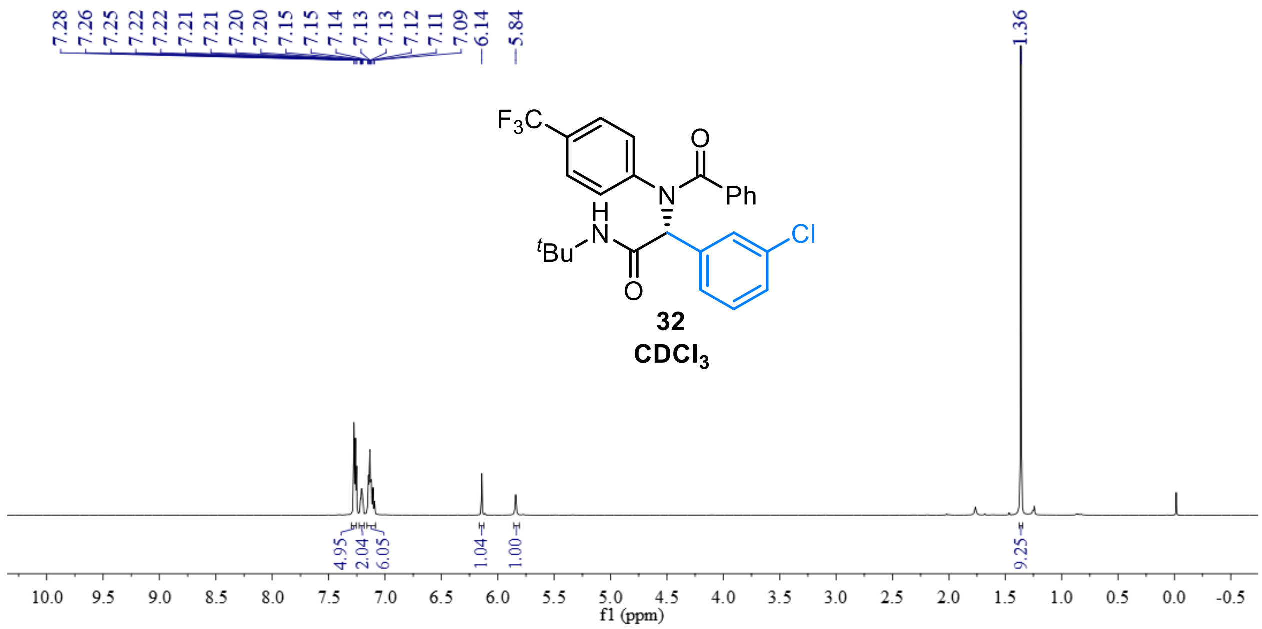

Supplementary Fig. 115. ^{1}H NMR spectrum of **32**. The sample has been recorded in 600 MHz, $CDCl_3$ at 25 °C.

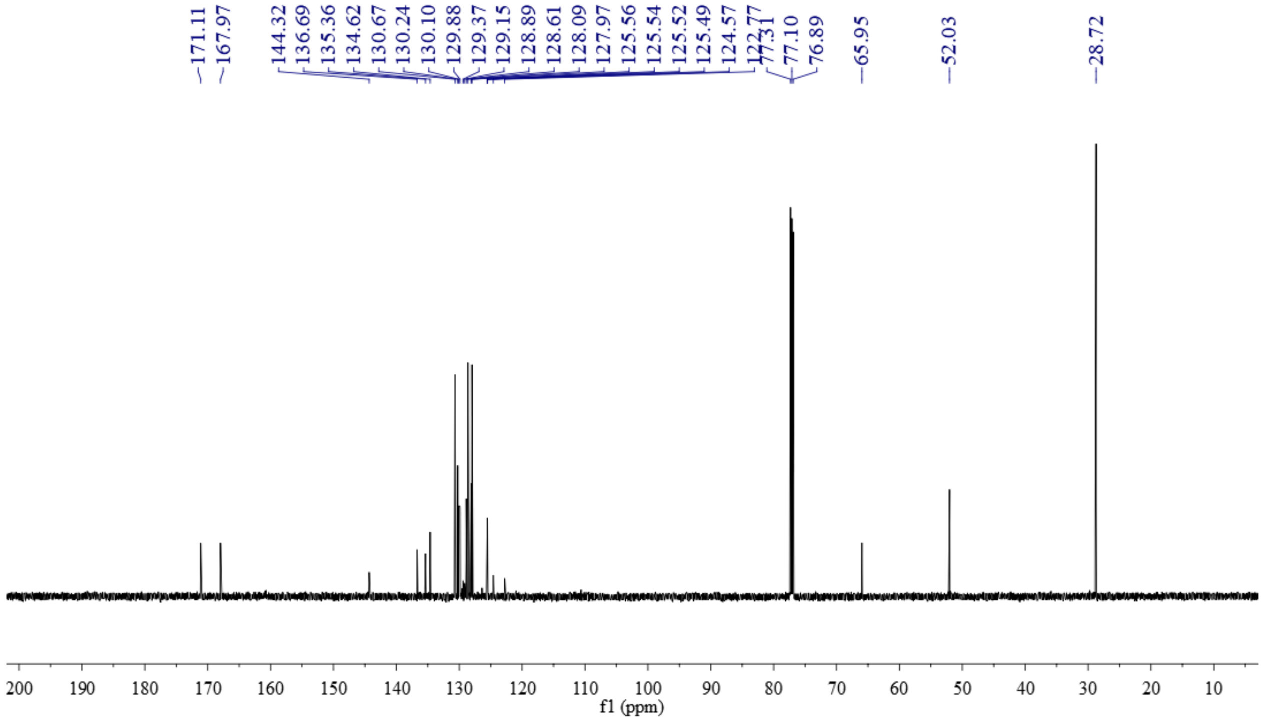

Supplementary Fig. 116. ^{13}C NMR spectrum of **32**. The sample has been recorded in 151 MHz, $CDCl_3$ at 25 °C.

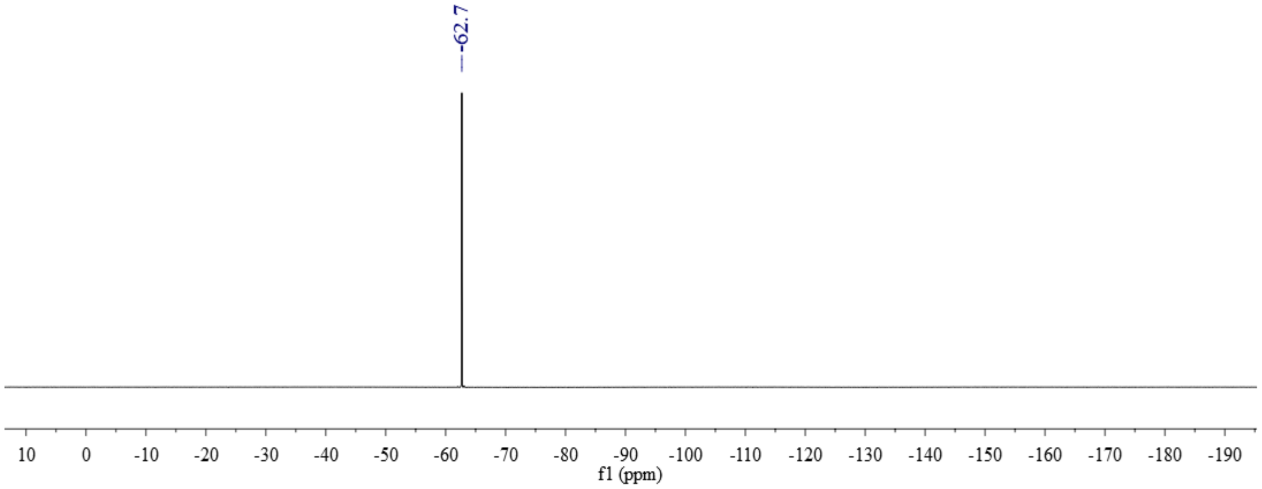

Supplementary Fig. 117. ^{31}F NMR spectrum of **32**. The sample has been recorded in 564 MHz, $CDCl_3$ at 25 °C.

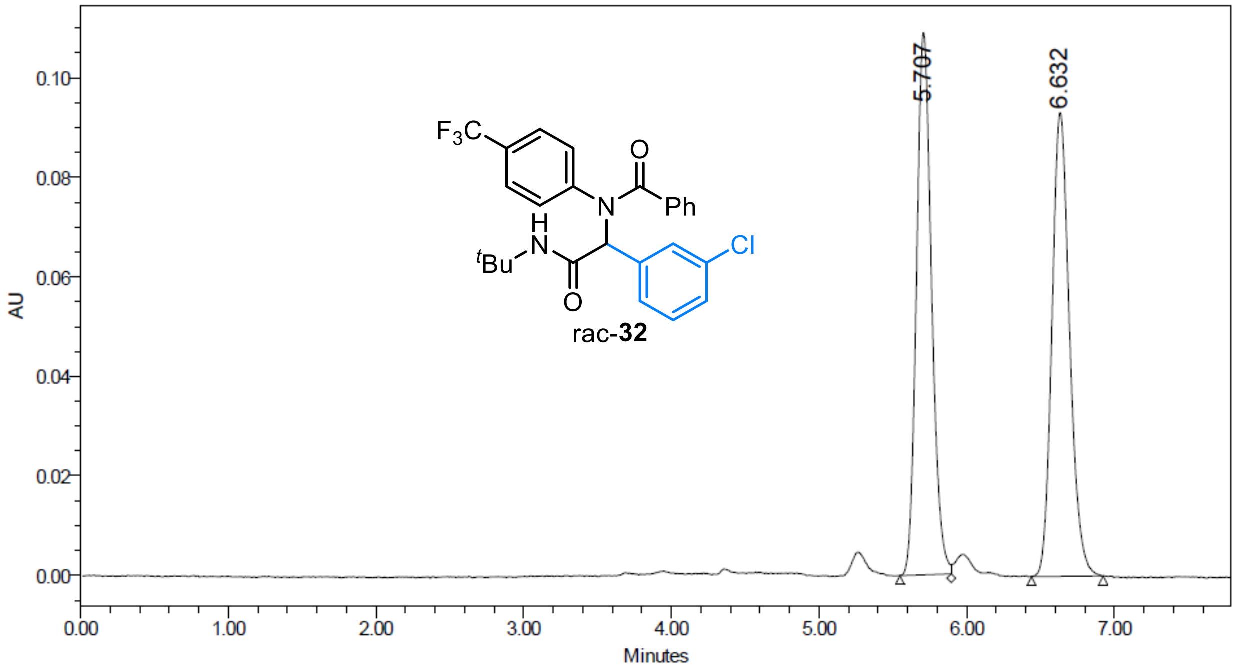

	RT (min)	Peak Type	Area (µV*sec)	% Area	Height (µV)	% Height	Integration Type	Points Across Peak	Start Time (min)	End Time (min)
1	5.707	Unknown	775901	50.01	108996	53.90	BV	209	5.547	5.895
2	6.632	Unknown	775494	49.99	93223	46.10	BB	291	6.438	6.923

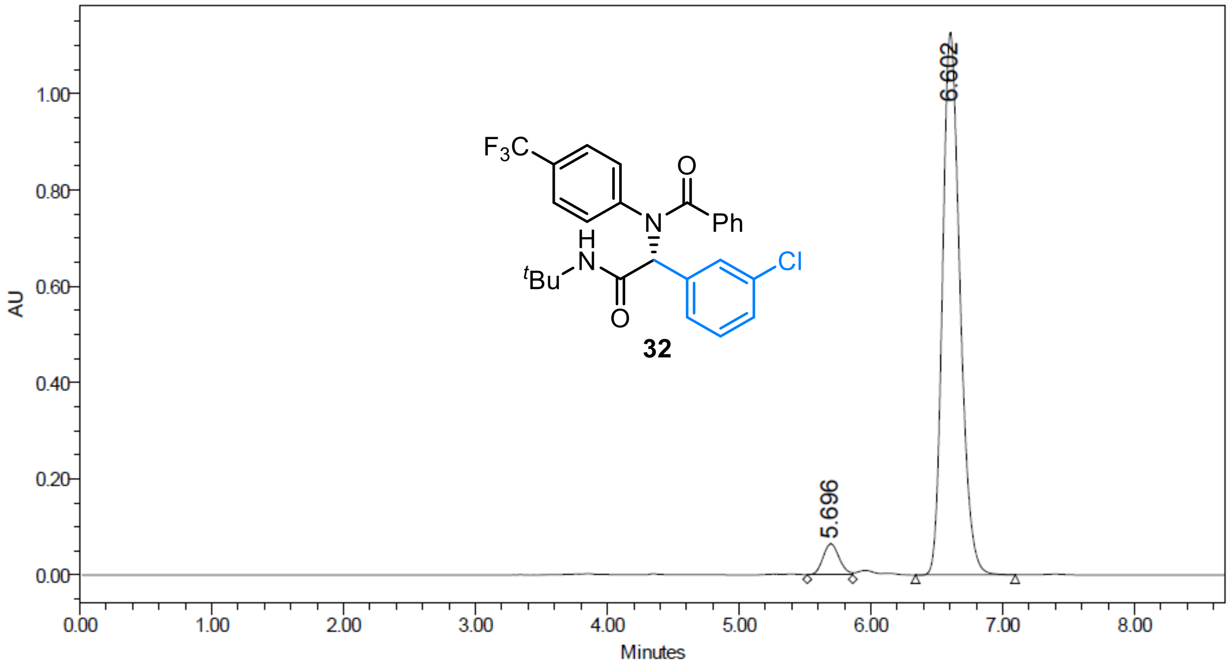

	RT (min)	Peak Type	Area (µV*sec)	% Area	Height (µV)	% Height	Integration Type	Points Across Peak	Start Time (min)	End Time (min)
1	5.696	Unknown	555143	5.07	64574	5.42	VV	207	5.517	5.862
2	6.602	Unknown	10393487	94.93	1127414	94.58	BB	454	6.338	7.095

Supplementary Fig. 118. HPLC of product **32**.

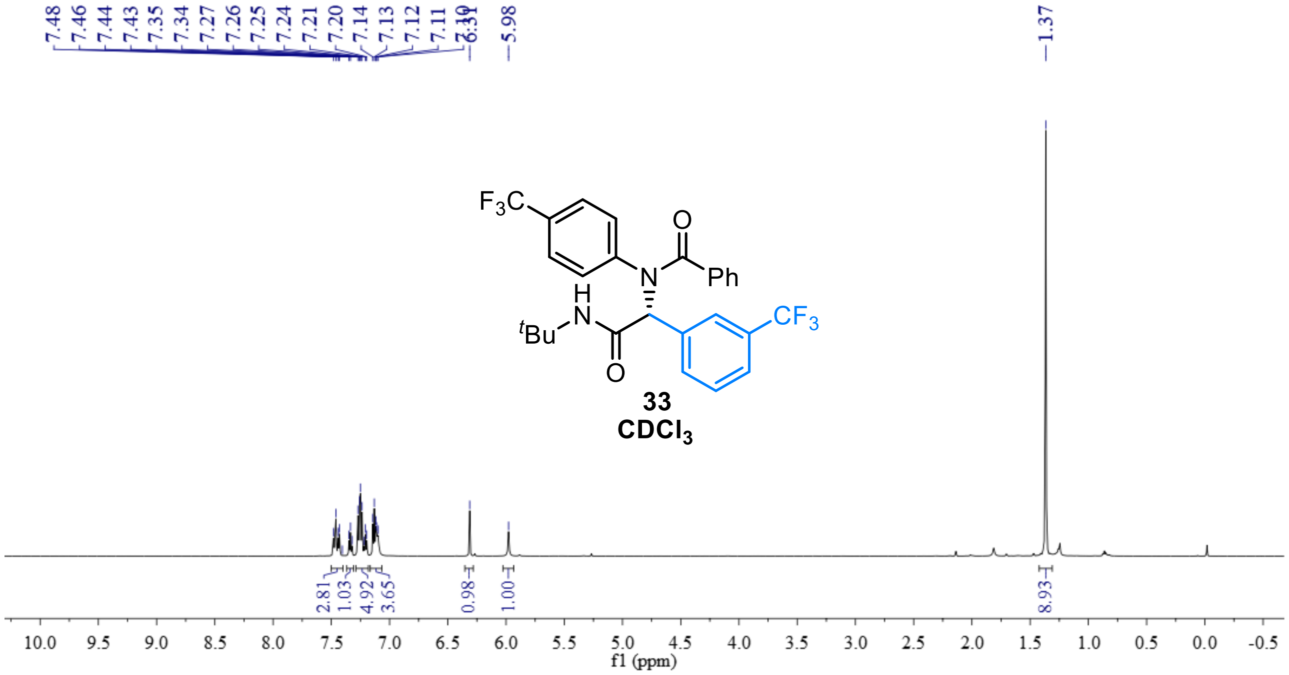

Supplementary Fig. 119. ^{1}H NMR spectrum of **33**. The sample has been recorded in 600 MHz, $CDCl_3$ at 25 °C.

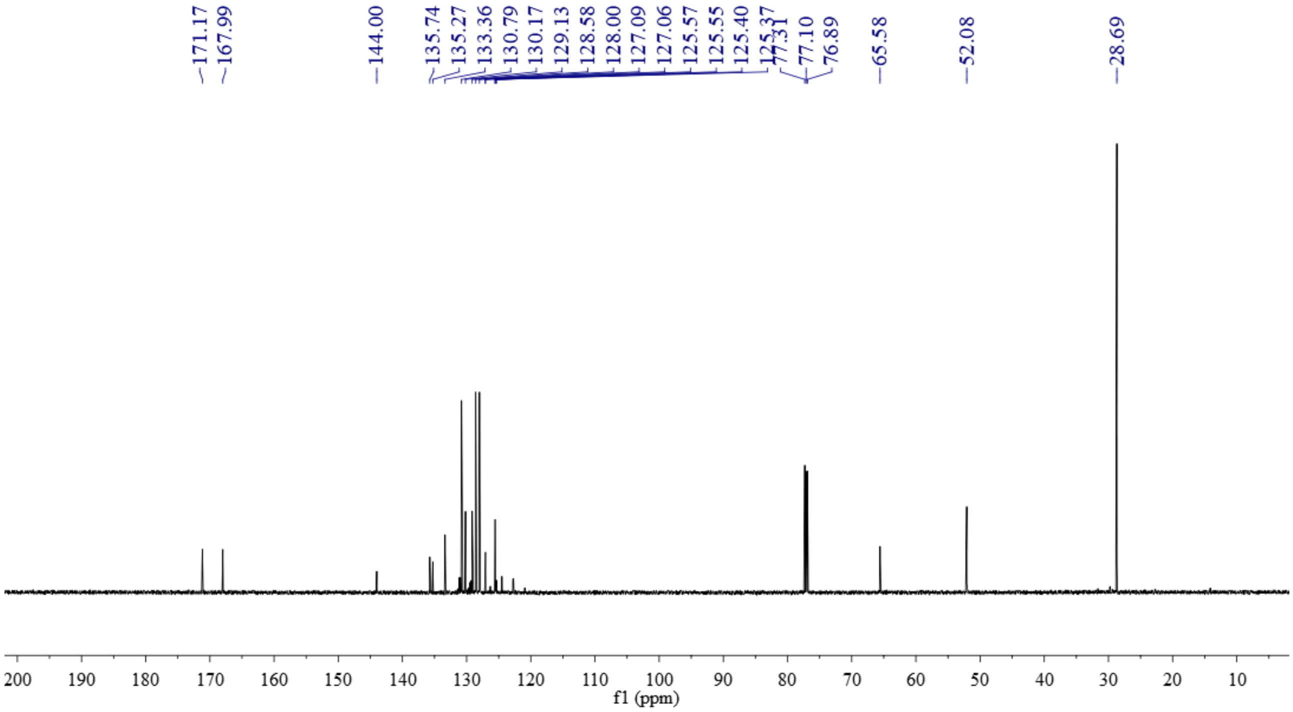

Supplementary Fig. 120. ^{13}C NMR spectrum of **33**. The sample has been recorded in 151 MHz, $CDCl_3$ at 25 °C.

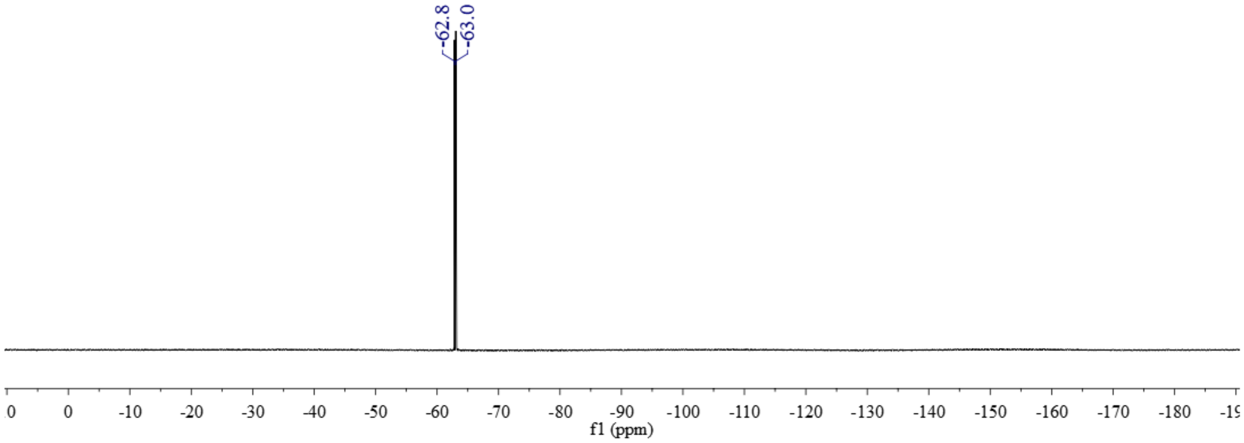

Supplementary Fig. 121. ^{31}F NMR spectrum of **33**. The sample has been recorded in 564 MHz, $CDCl_3$ at 25 °C.

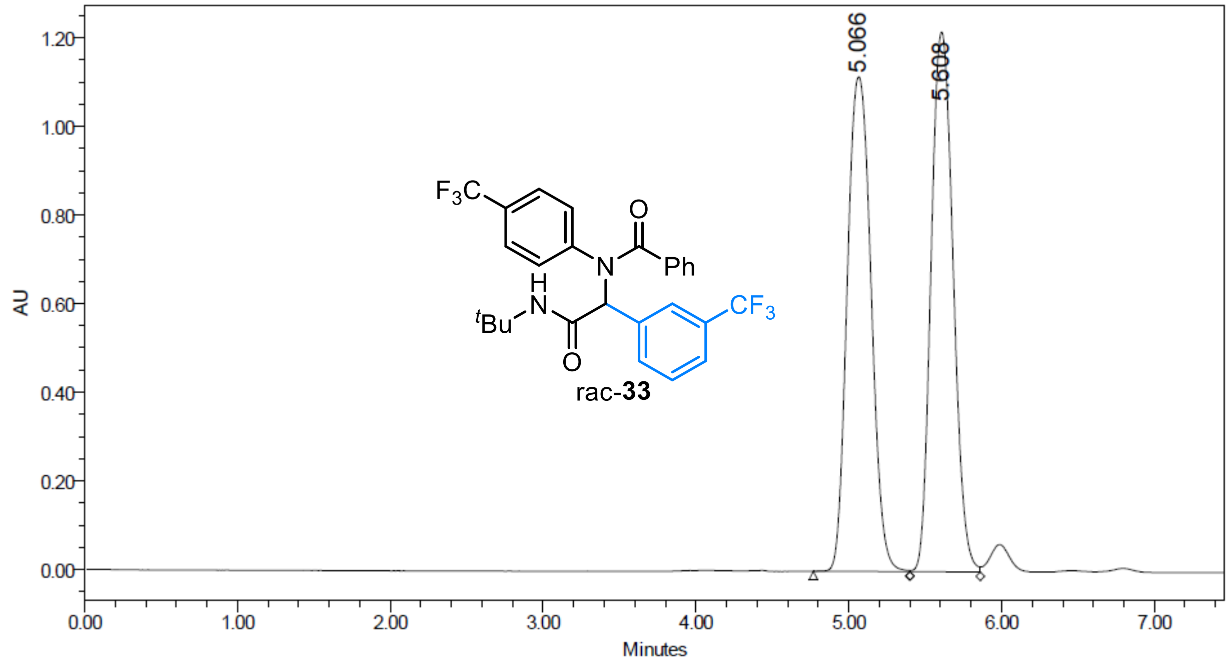

	RT (min)	Peak Type	Area (µV*sec)	% Area	Height (µV)	% Height	Integration Type	Points Across Peak	Start Time (min)	End Time (min)
1	5.066	Unknown	12104467	49.76	1116588	47.83	BV	379	4.768	5.400
2	5.608	Unknown	12218789	50.24	1217898	52.17	VV	277	5.400	5.862

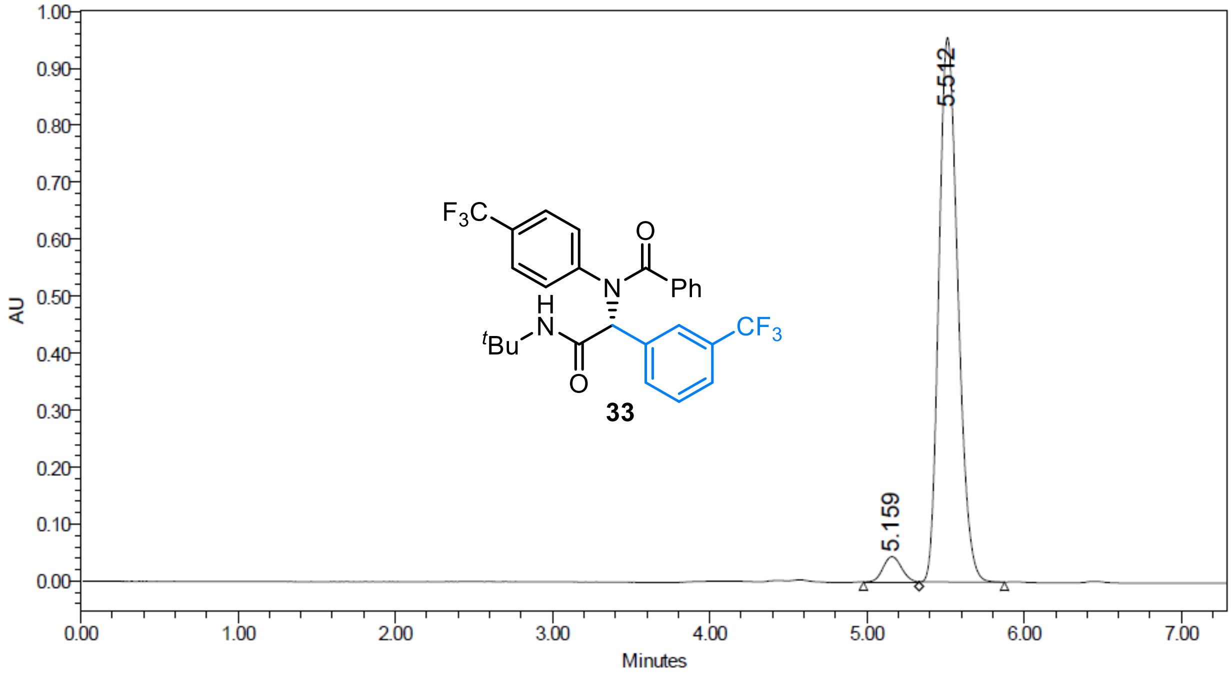

	RT (min)	Peak Type	Area (µV*sec)	% Area	Height (µV)	% Height	Integration Type	Points Across Peak	Start Time (min)	End Time (min)
1	5.159	Unknown	363062	4.33	44457	4.44	BV	212	4.978	5.332
2	5.512	Unknown	8020911	95.67	955828	95.56	VB	326	5.332	5.875

Supplementary Fig. 122. HPLC of product **33**.

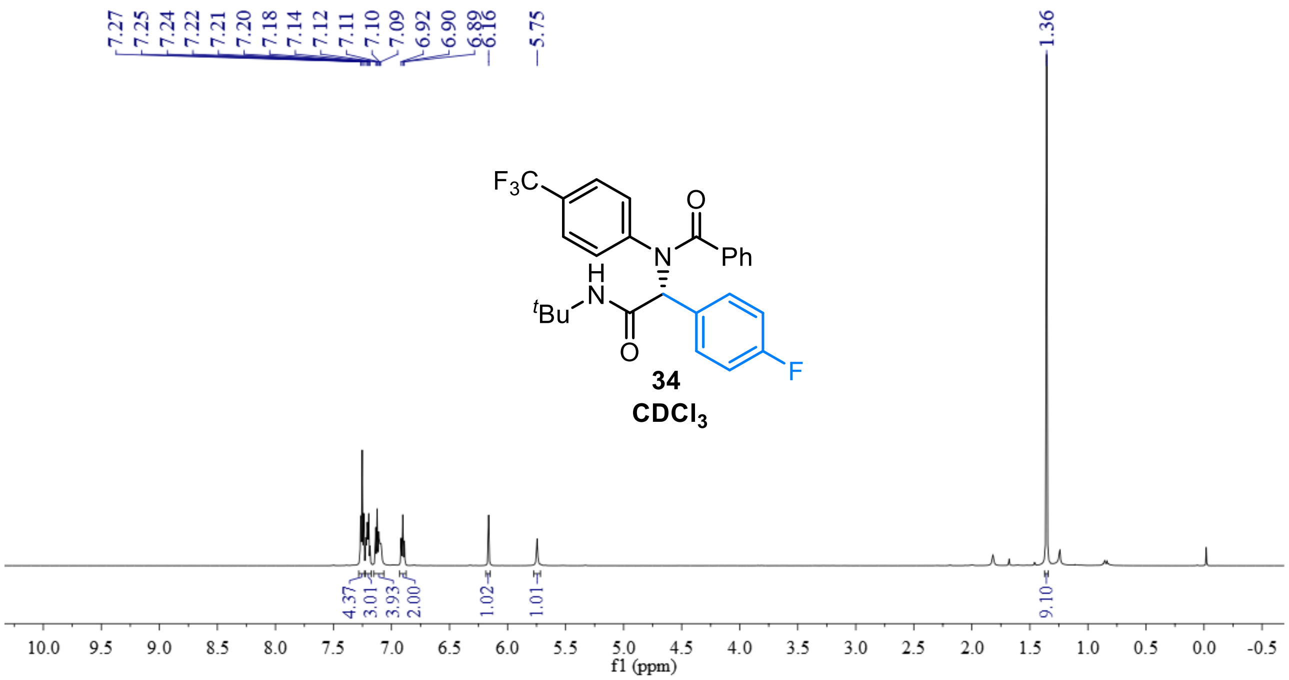

Supplementary Fig. 123. ^{1}H NMR spectrum of **34**. The sample has been recorded in 600 MHz, $CDCl_3$ at 25 °C.

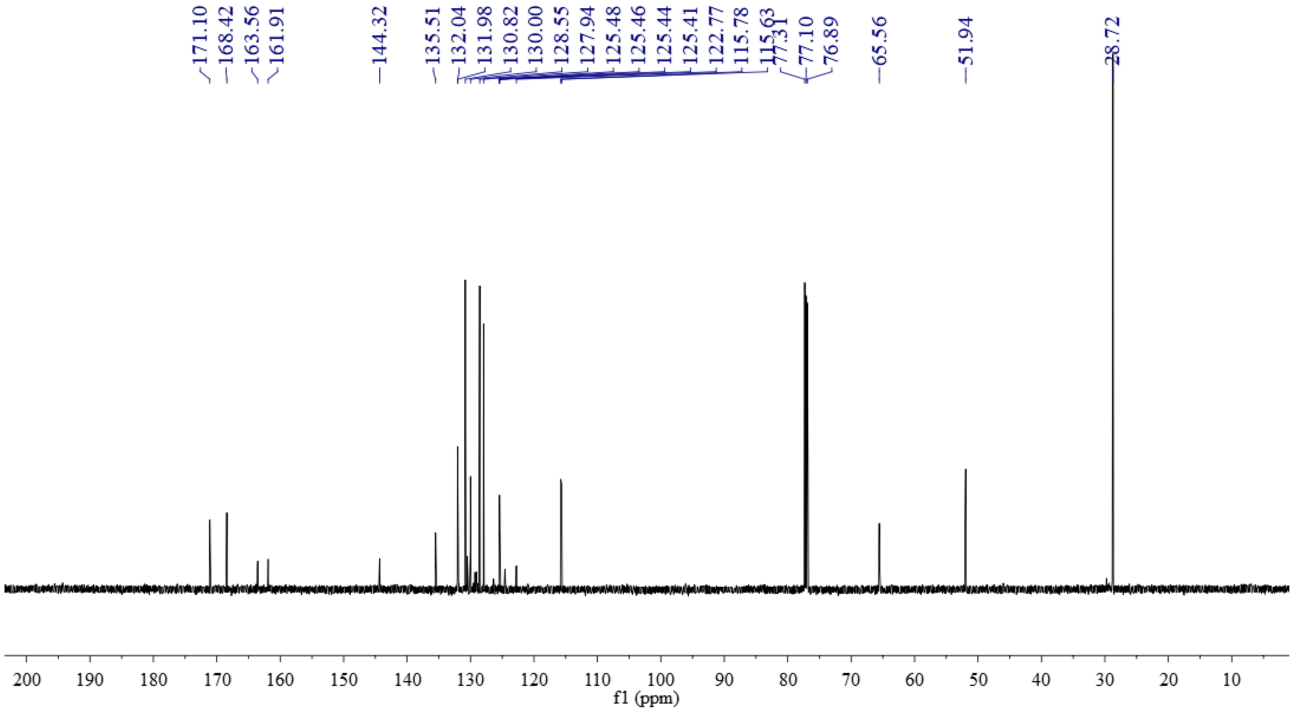

Supplementary Fig. 124. ^{13}C NMR spectrum of **34**. The sample has been recorded in 151 MHz, $CDCl_3$ at 25 °C.

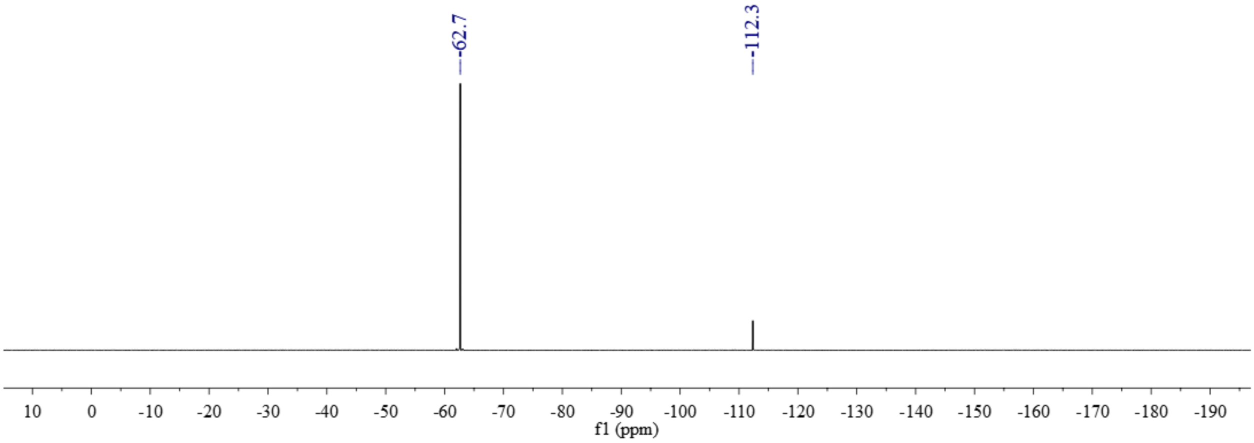

Supplementary Fig. 125. ^{31}F NMR spectrum of **34**. The sample has been recorded in 564 MHz, $CDCl_3$ at 25 °C.

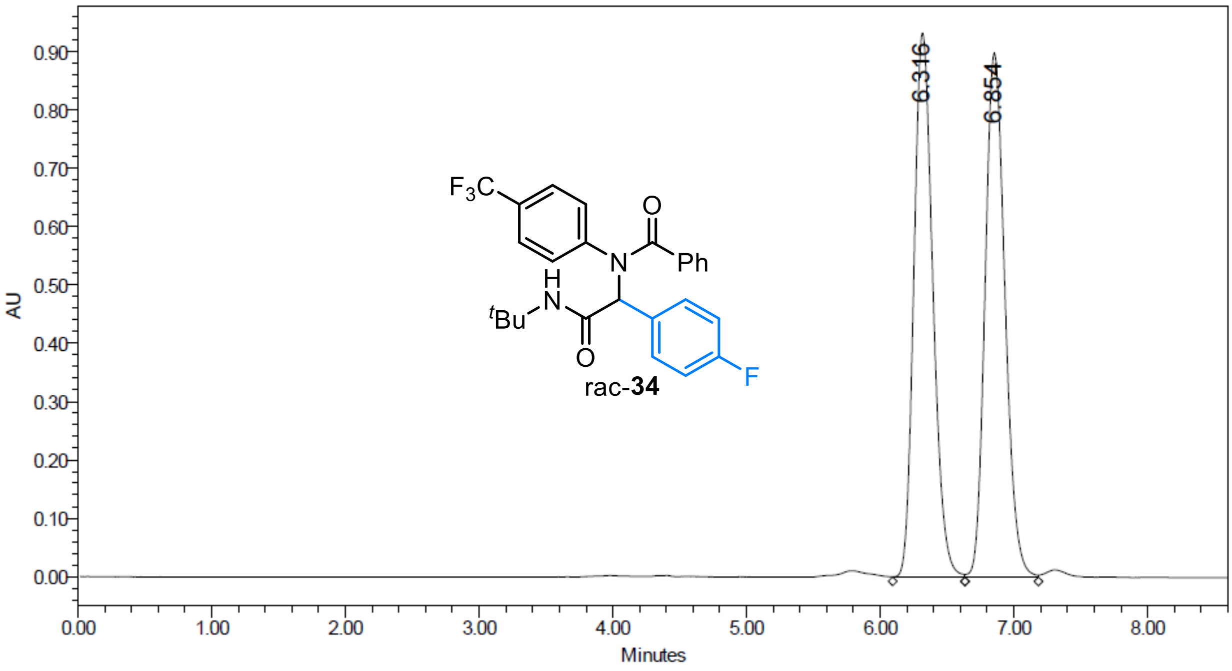

	RT (min)	Peak Type	Area (µV*sec)	% Area	Height (µV)	% Height	Integration Type	Points Across Peak	Start Time (min)	End Time (min)
1	6.316	Unknown	9206808	49.82	932443	50.95	VV	324	6.093	6.633
2	6.854	Unknown	9274557	50.18	897641	49.05	VV	330	6.633	7.183

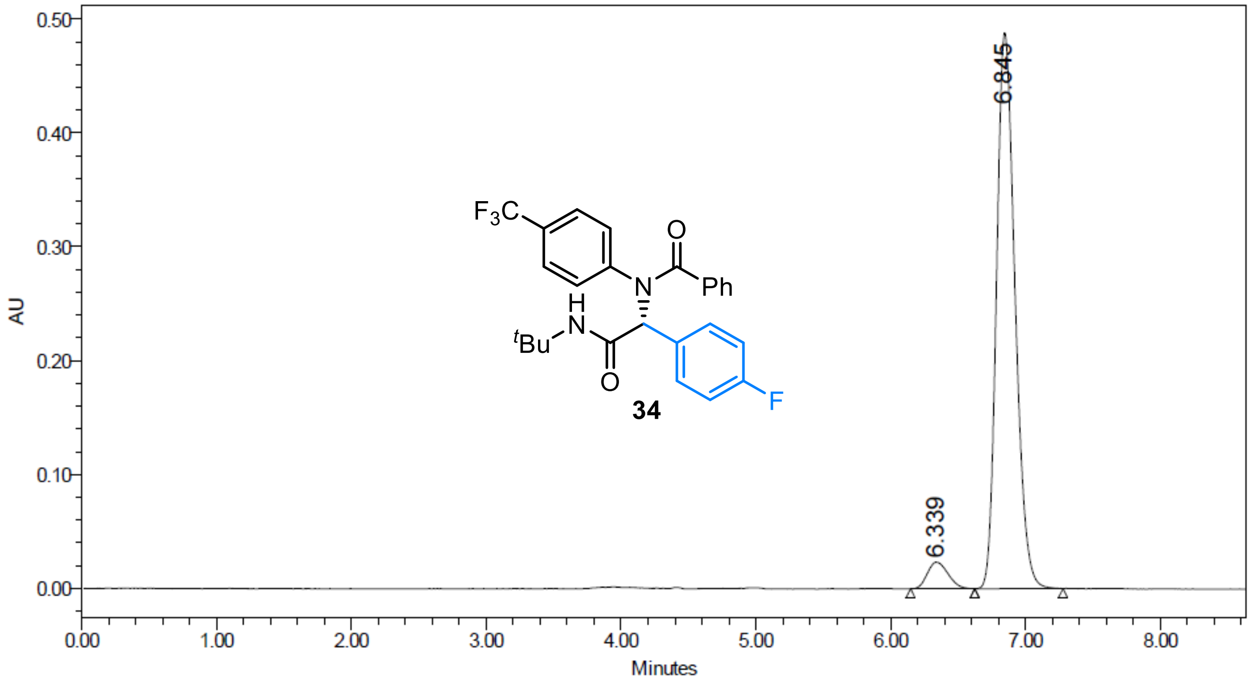

	RT (min)	Peak Type	Area (µV*sec)	% Area	Height (µV)	% Height	Integration Type	Points Across Peak	Start Time (min)	End Time (min)
1	6.339	Unknown	248483	5.08	23179	4.53	BB	286	6.147	6.623
2	6.845	Unknown	4639984	94.92	488174	95.47	BB	392	6.623	7.277

Supplementary Fig. 126. HPLC of product **34**.

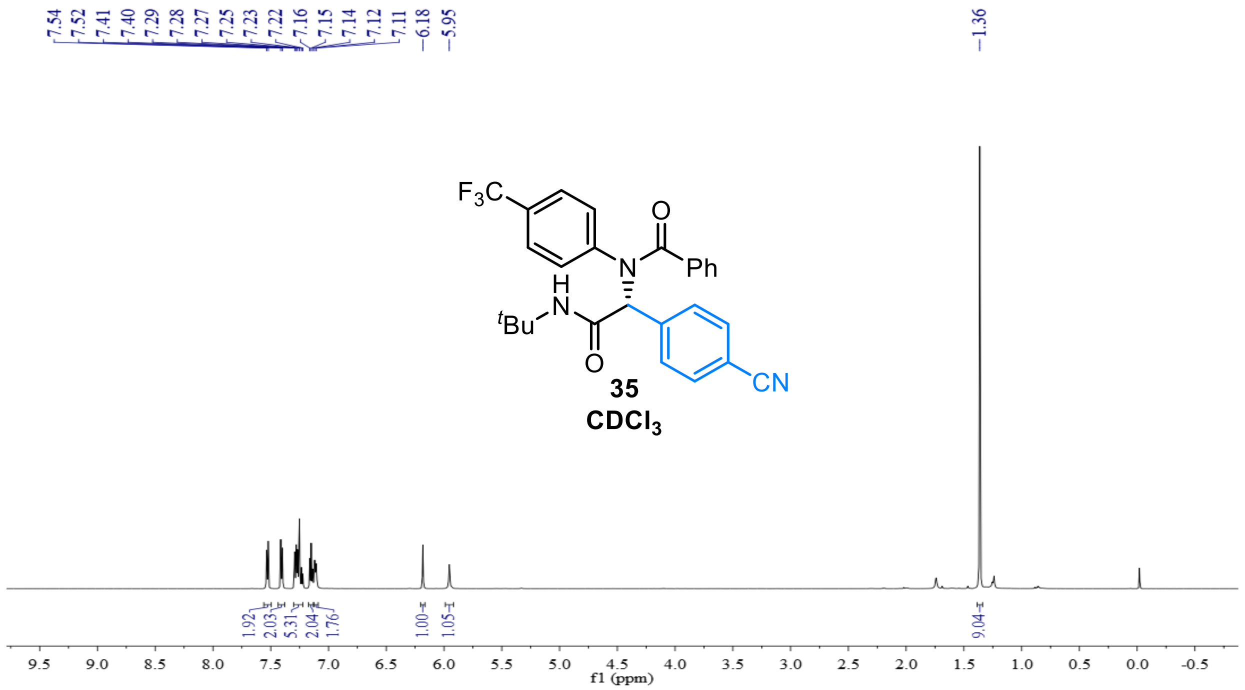

Supplementary Fig. 127. ^{1}H NMR spectrum of **35**. The sample has been recorded in 600 MHz, CDCl$_3$ at 25 °C.

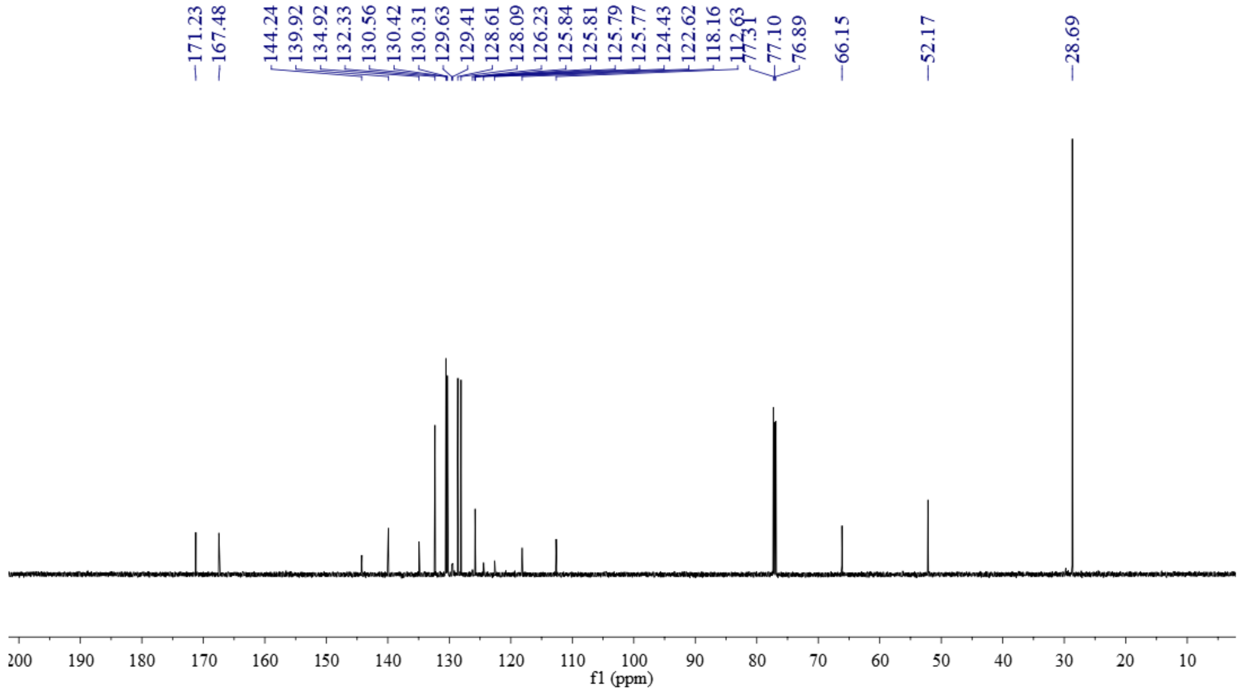

Supplementary Fig. 128. ^{13}C NMR spectrum of **35**. The sample has been recorded in 151 MHz, CDCl$_3$ at 25 °C.

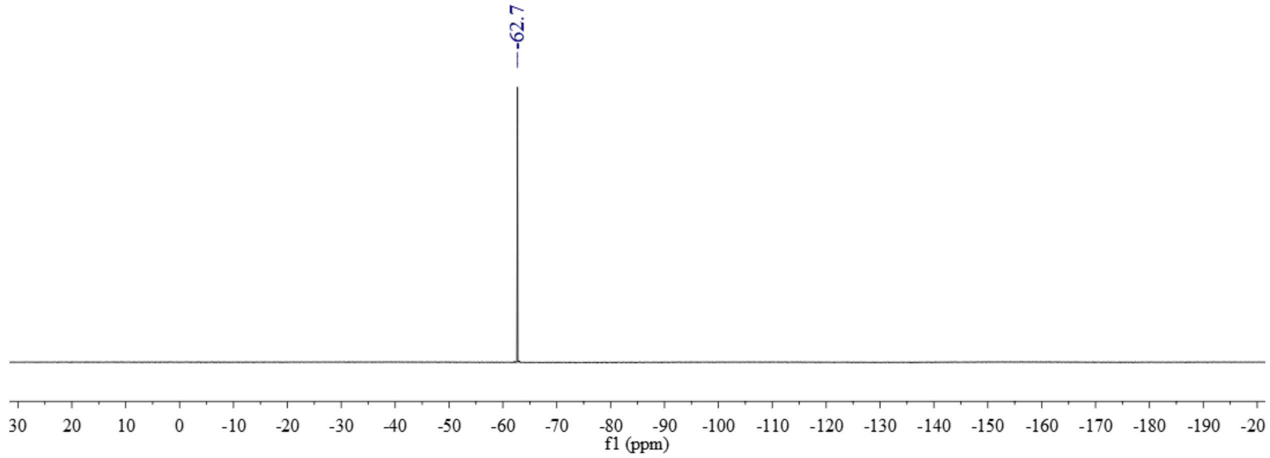

Supplementary Fig. 129. ^{31}F NMR spectrum of **35**. The sample has been recorded in 564 MHz, CDCl$_3$ at 25 °C.

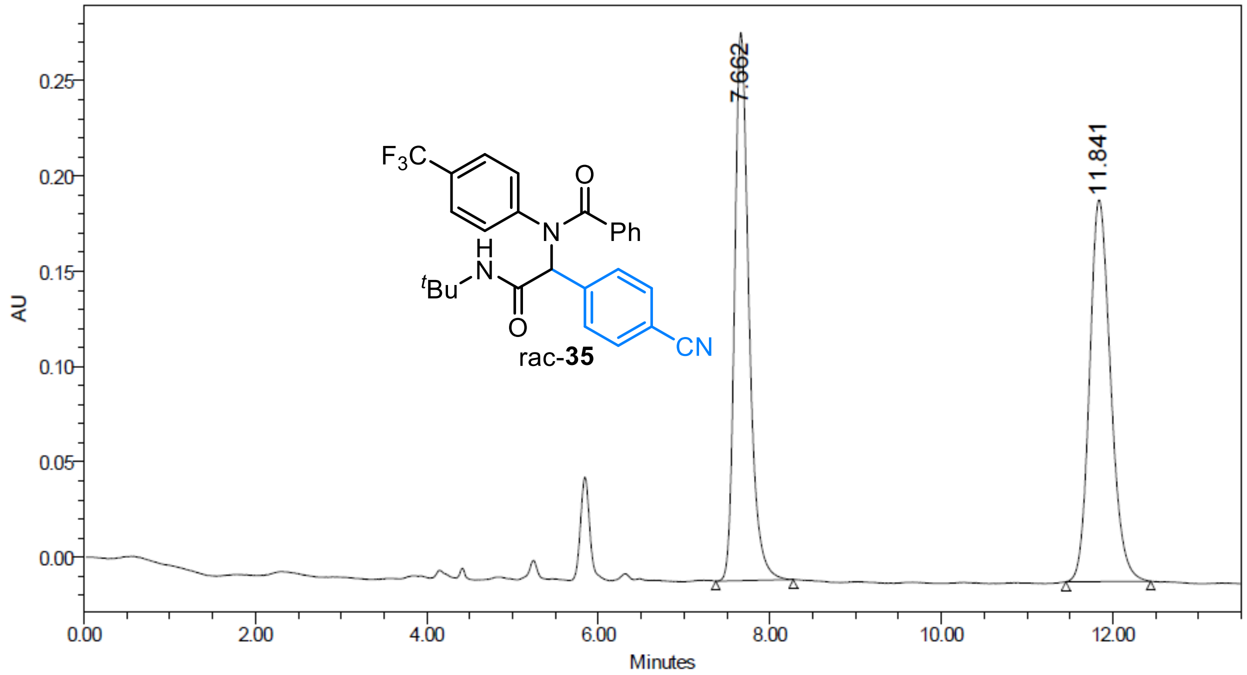

	RT (min)	Peak Type	Area (µV*sec)	% Area	Height (µV)	% Height	Integration Type	Points Across Peak	Start Time (min)	End Time (min)
1	7.662	Unknown	3339075	49.39	287513	58.95	BB	543	7.370	8.275
2	11.841	Unknown	3421306	50.61	200174	41.05	BB	593	11.455	12.443

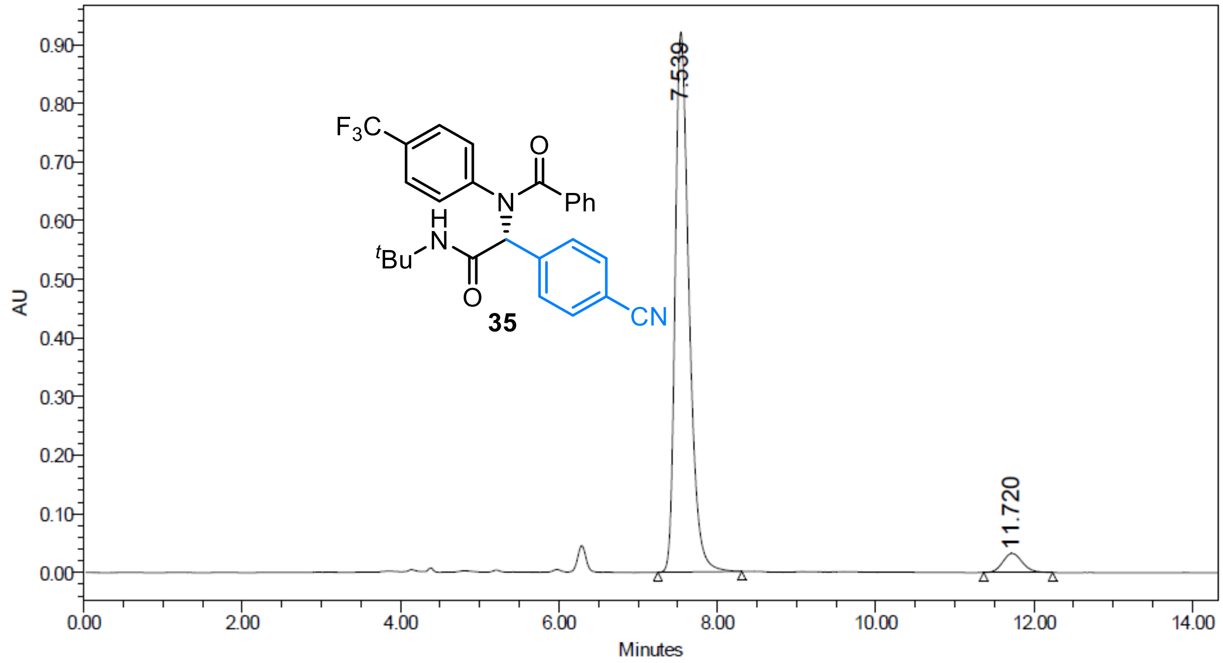

	RT (min)	Peak Type	Area (µV*sec)	% Area	Height (µV)	% Height	Integration Type	Points Across Peak	Start Time (min)	End Time (min)
1	7.539	Unknown	11196457	95.21	920735	96.58	BB	636	7.250	8.310
2	11.720	Unknown	563818	4.79	32637	3.42	BB	523	11.363	12.235

Supplementary Fig. 130. HPLC of product **35**.

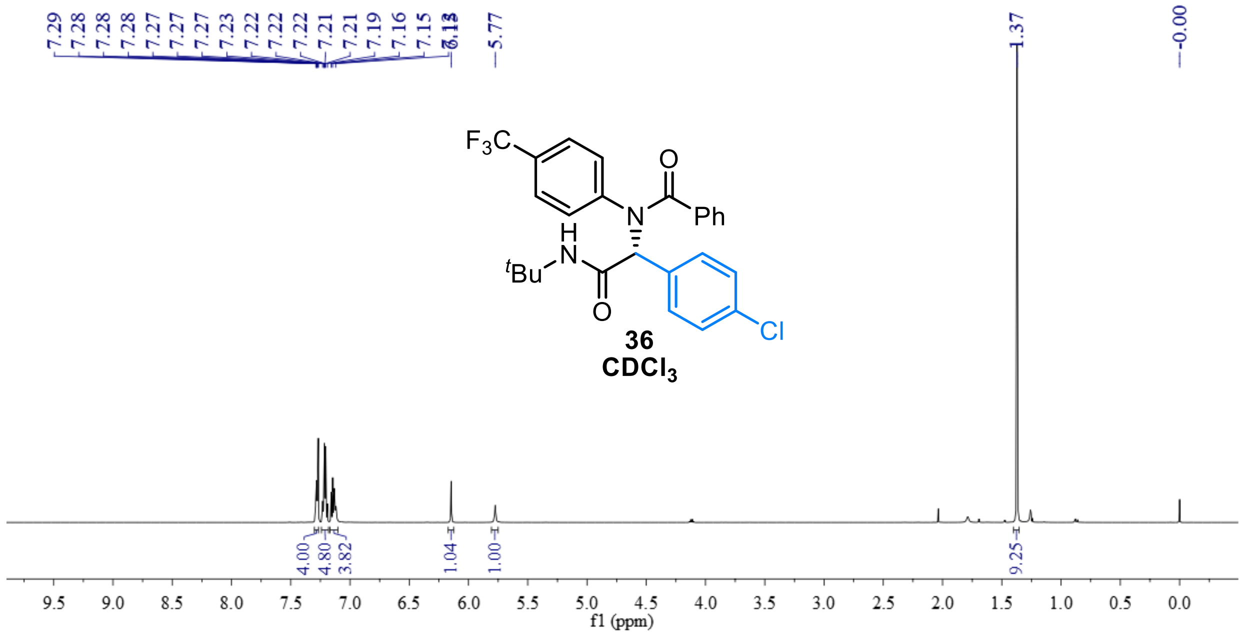

Supplementary Fig. 131. ^{1}H NMR spectrum of **36**. The sample has been recorded in 600 MHz, $CDCl_3$ at 25 °C.

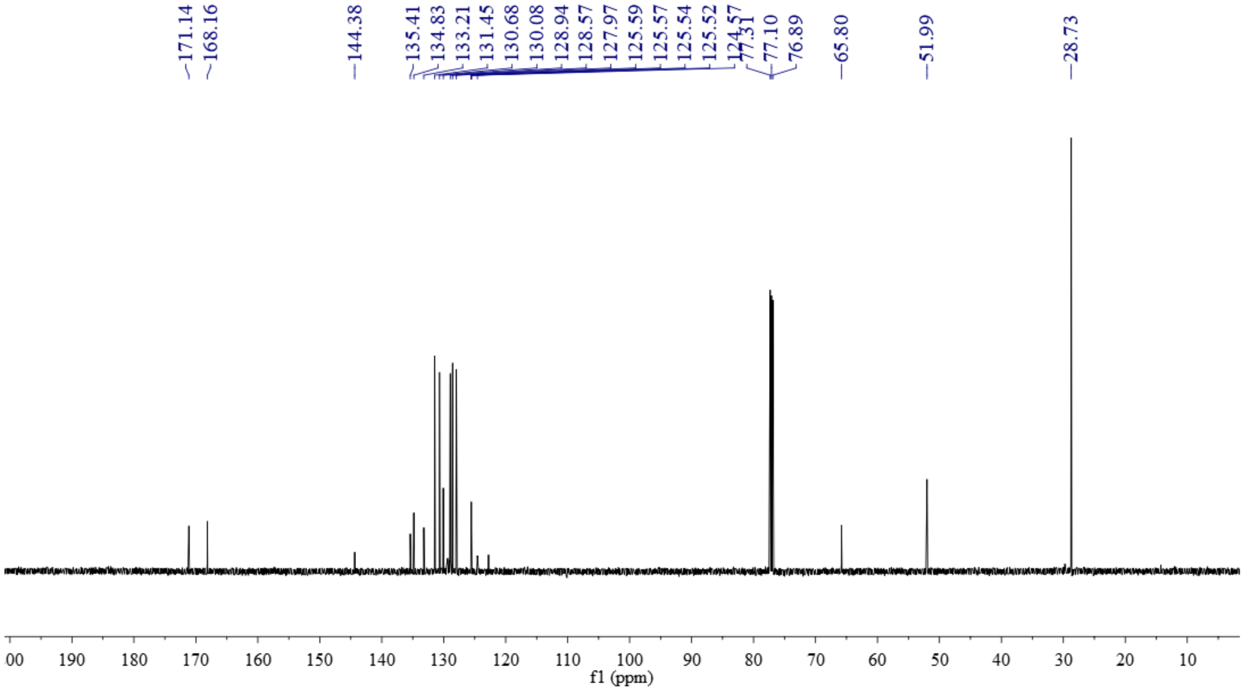

Supplementary Fig. 132. ^{13}C NMR spectrum of **36**. The sample has been recorded in 151 MHz, $CDCl_3$ at 25 °C.

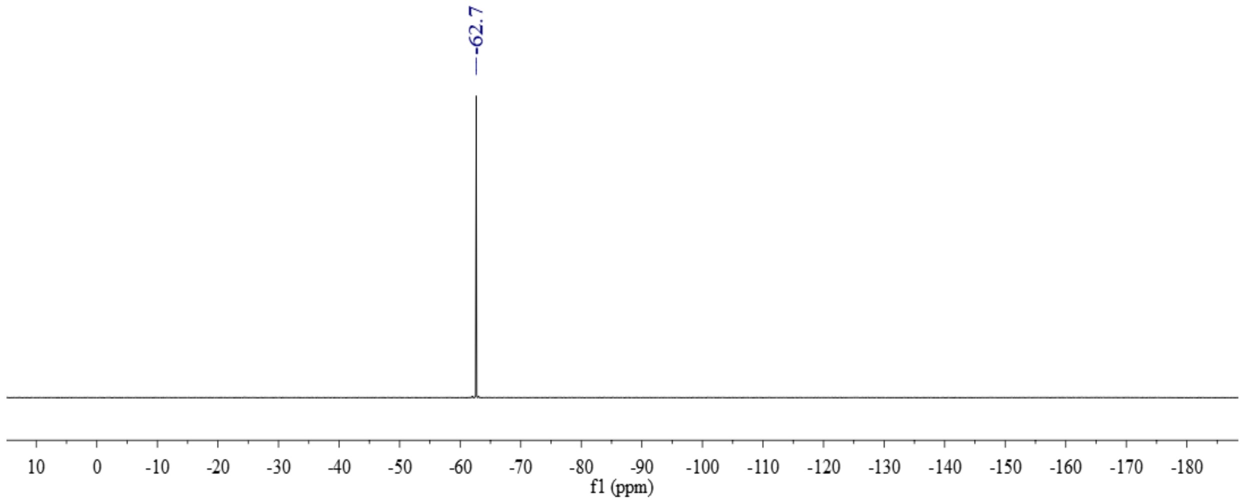

Supplementary Fig. 133. ^{31}F NMR spectrum of **36**. The sample has been recorded in 564 MHz, $CDCl_3$ at 25 °C.

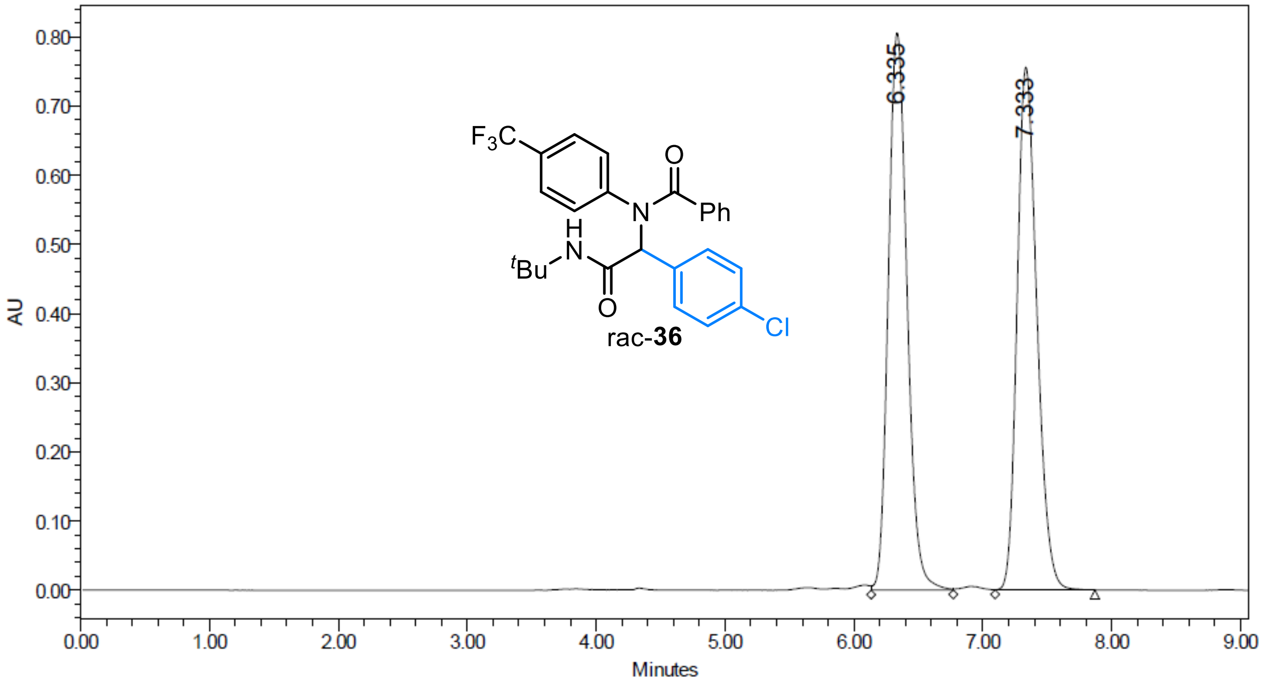

	RT (min)	Peak Type	Area (µV*sec)	% Area	Height (µV)	% Height	Integration Type	Points Across Peak	Start Time (min)	End Time (min)
1	6.335	Unknown	8122054	50.26	805410	51.59	VV	382	6.133	6.770
2	7.333	Unknown	8038204	49.74	755711	48.41	VB	464	7.095	7.868

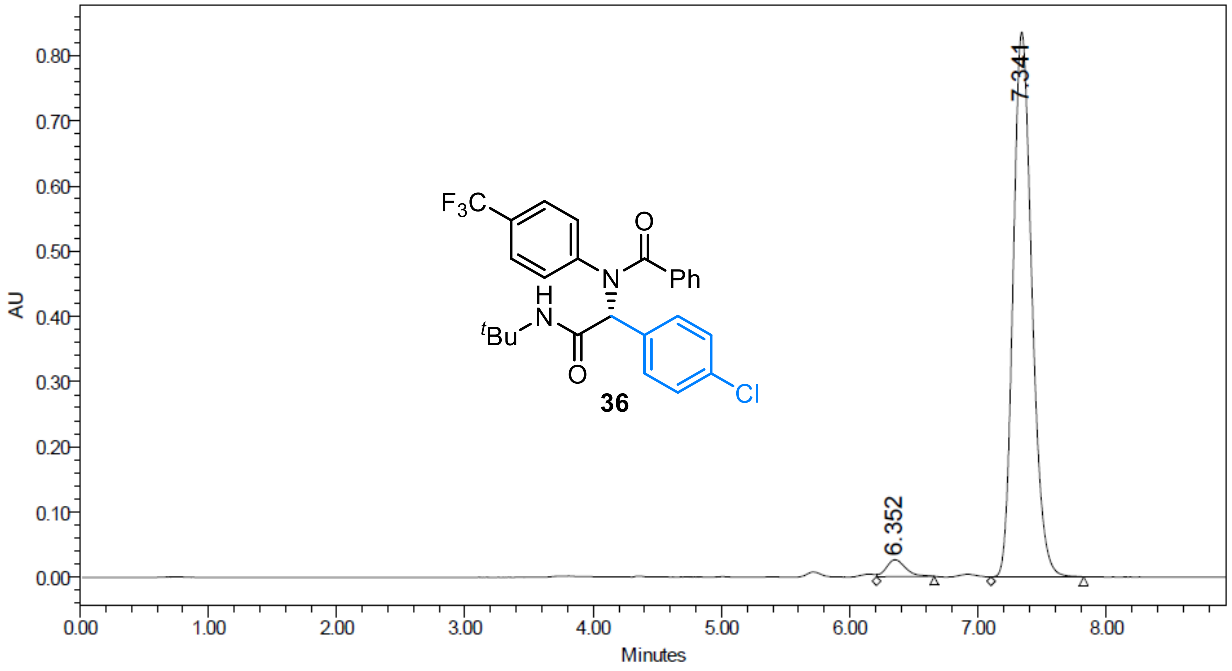

	RT (min)	Peak Type	Area (µV*sec)	% Area	Height (µV)	% Height	Integration Type	Points Across Peak	Start Time (min)	End Time (min)
1	6.352	Unknown	267021	3.05	25782	2.99	Vb	270	6.208	6.658
2	7.341	Unknown	8493913	96.95	835095	97.01	VB	433	7.102	7.823

Supplementary Fig. 134. HPLC of product 3**6**.

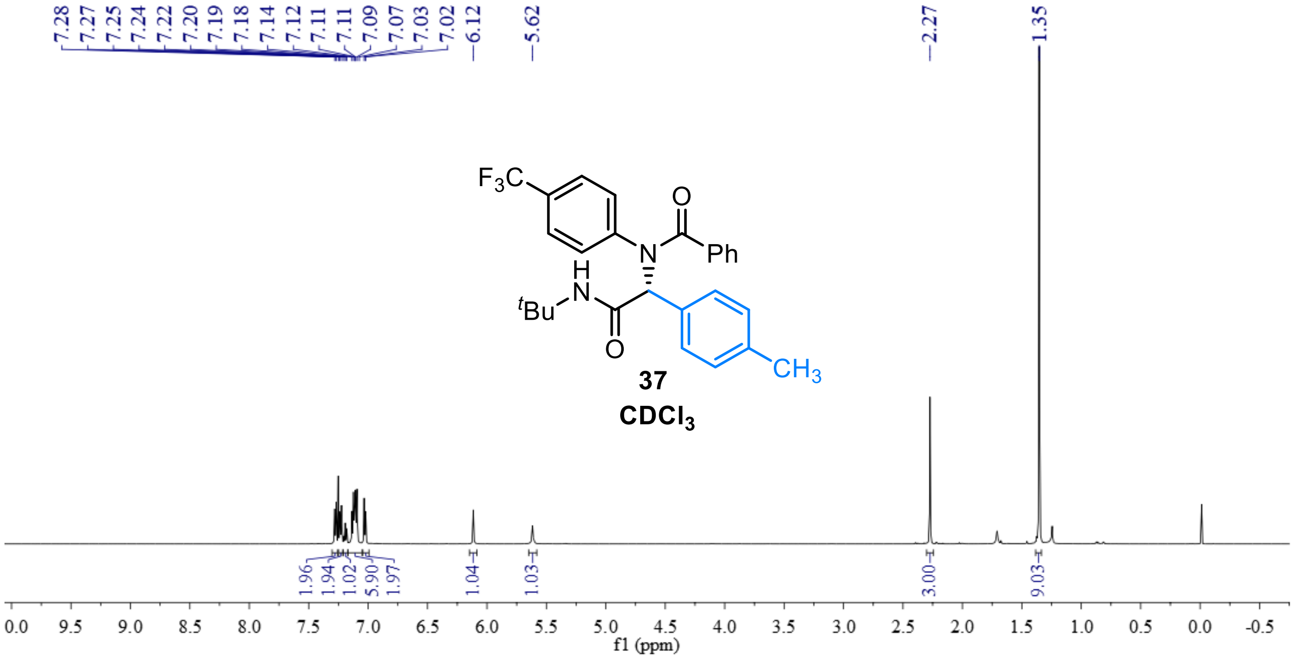

Supplementary Fig. 135. ^{1}H NMR spectrum of **37**. The sample has been recorded in 600 MHz, $CDCl_3$ at 25 °C.

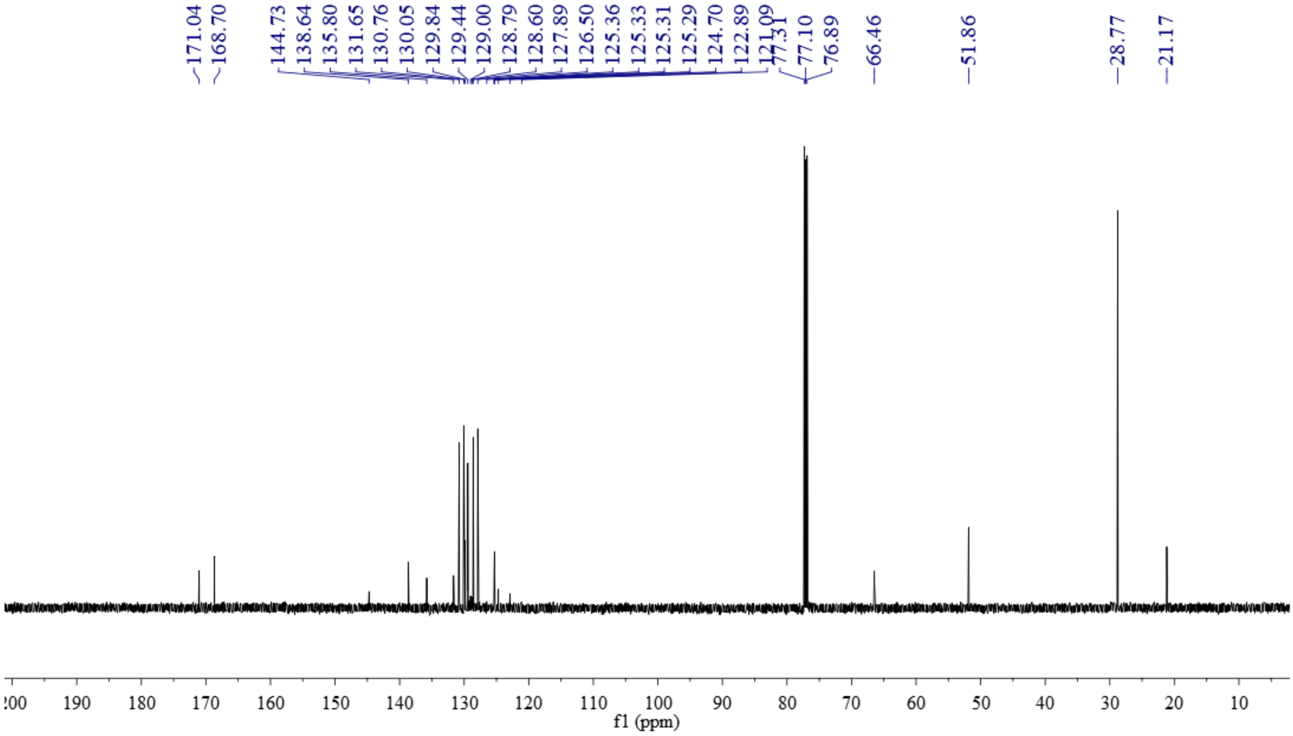

Supplementary Fig. 136. ^{13}C NMR spectrum of **37**. The sample has been recorded in 151 MHz, $CDCl_3$ at 25 °C.

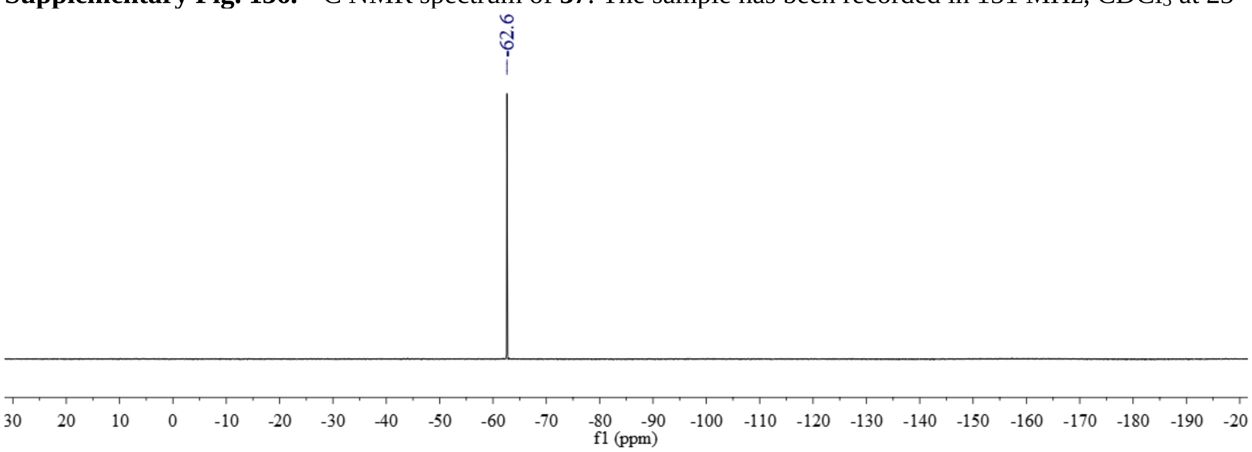

Supplementary Fig. 137. ^{31}F NMR spectrum of **37**. The sample has been recorded in 564 MHz, $CDCl_3$ at 25 °C.

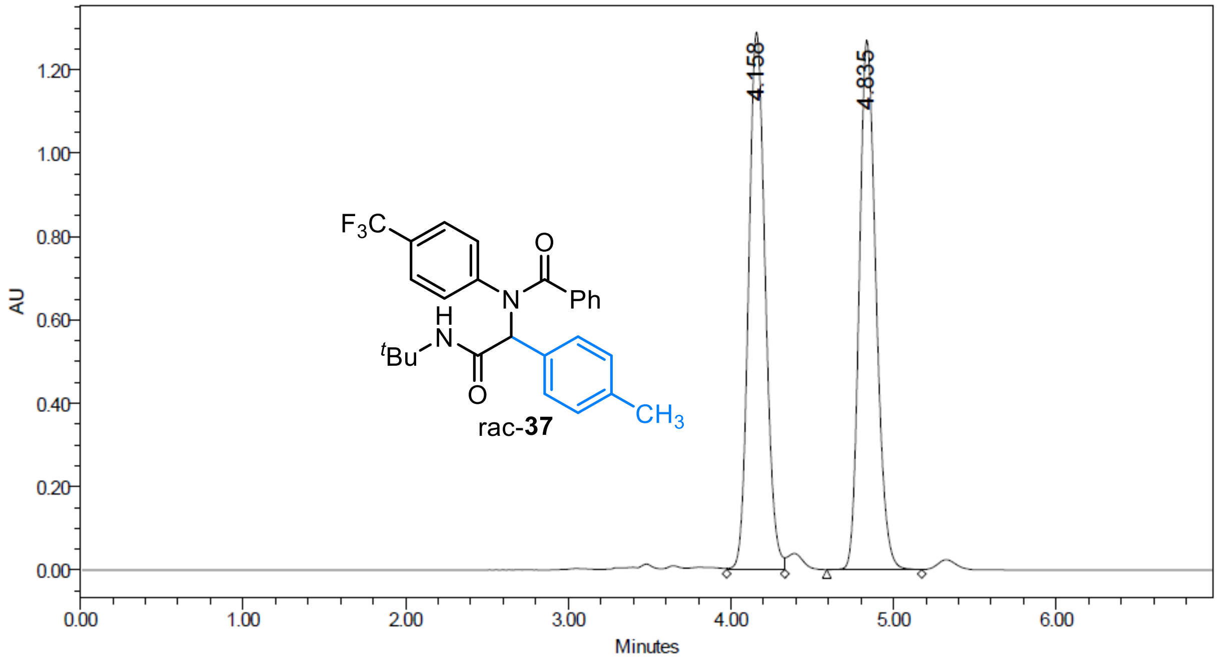

	RT (min)	Peak Type	Area (µV*sec)	% Area	Height (µV)	% Height	Integration Type	Points Across Peak	Start Time (min)	End Time (min)
1	4.158	Unknown	9152697	50.14	1289374	50.38	VV	215	3.973	4.332
2	4.835	Unknown	9103388	49.86	1269841	49.62	BV	350	4.590	5.173

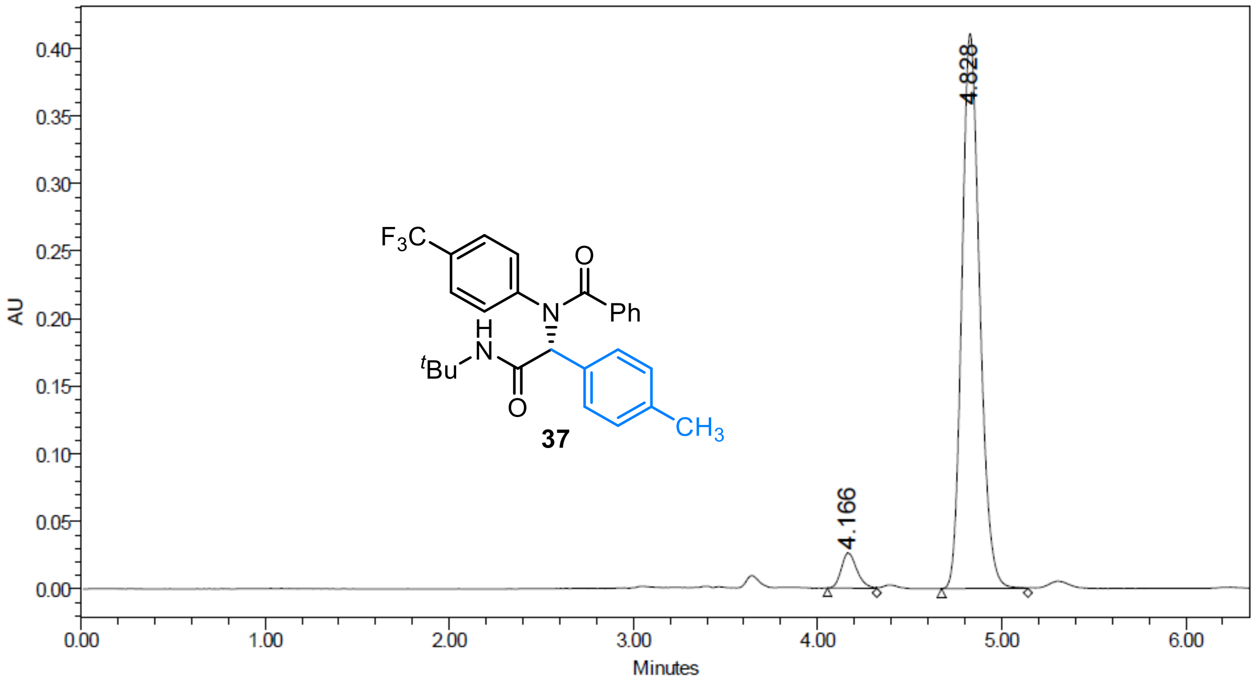

	RT (min)	Peak Type	Area (µV*sec)	% Area	Height (µV)	% Height	Integration Type	Points Across Peak	Start Time (min)	End Time (min)
1	4.166	Unknown	153496	5.26	26001	5.95	BV	161	4.053	4.322
2	4.828	Unknown	2767126	94.74	410666	94.05	BV	281	4.673	5.142

Supplementary Fig. 138. HPLC of product **37**.

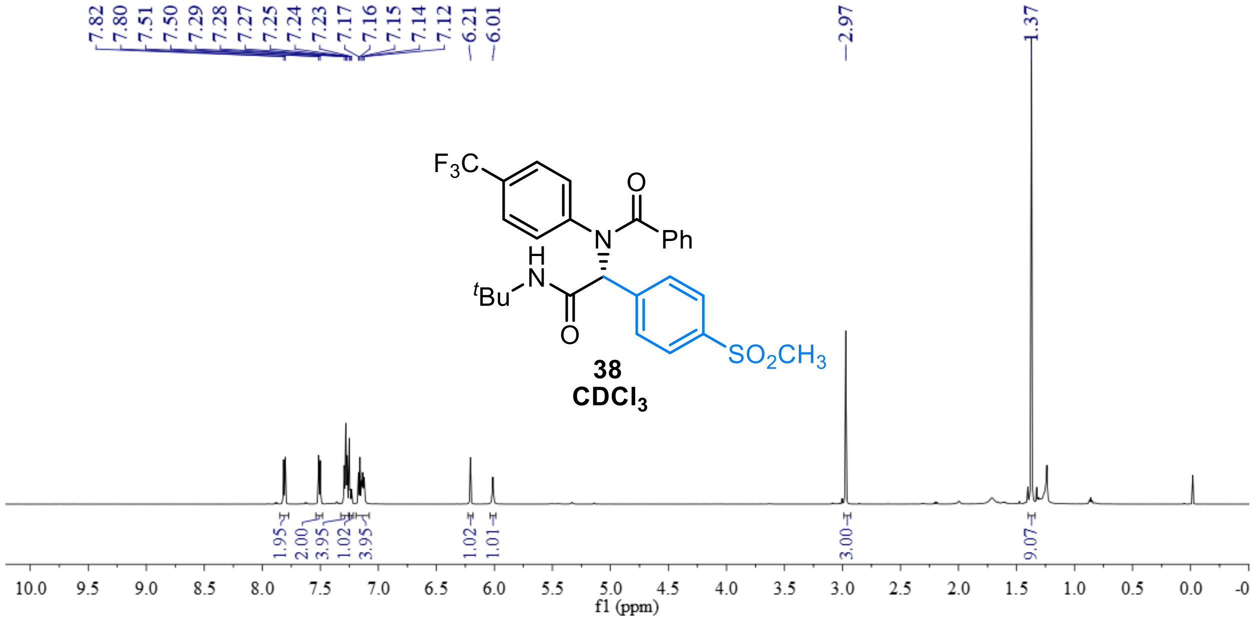

Supplementary Fig. 139. ^{1}H NMR spectrum of **38**. The sample has been recorded in 600 MHz, $CDCl_3$ at 25 °C.

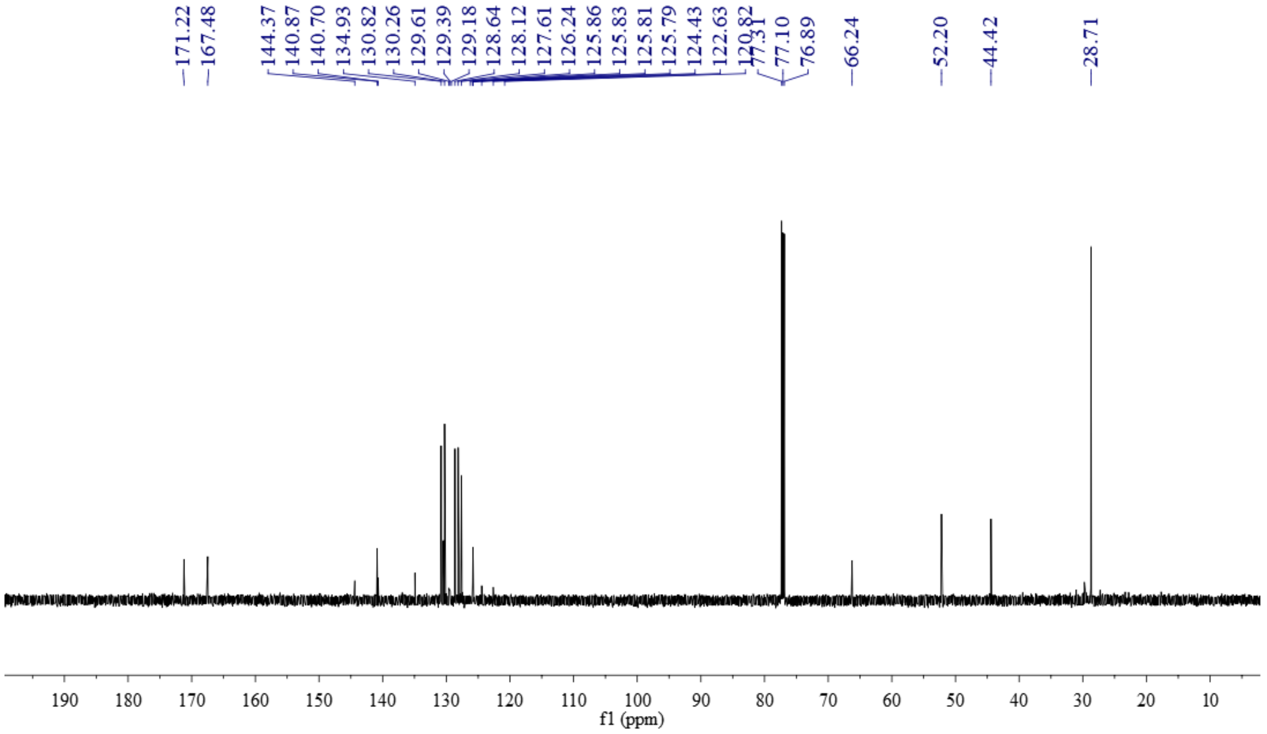

Supplementary Fig. 140. ^{13}C NMR spectrum of **38**. The sample has been recorded in 151 MHz, $CDCl_3$ at 25 °C.

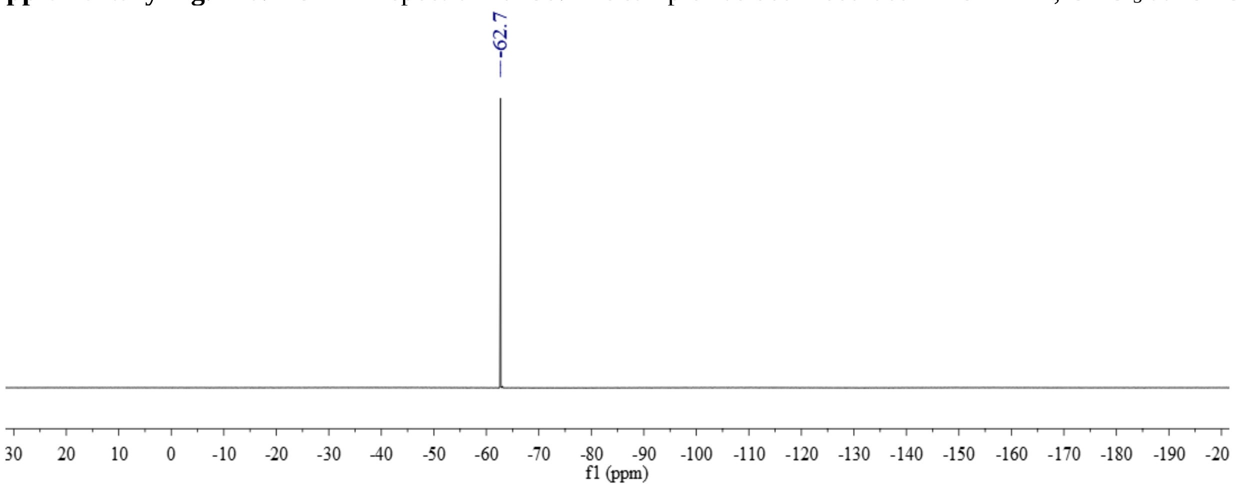

Supplementary Fig. 141. ^{31}F NMR spectrum of **38**. The sample has been recorded in 564 MHz, $CDCl_3$ at 25 °C.

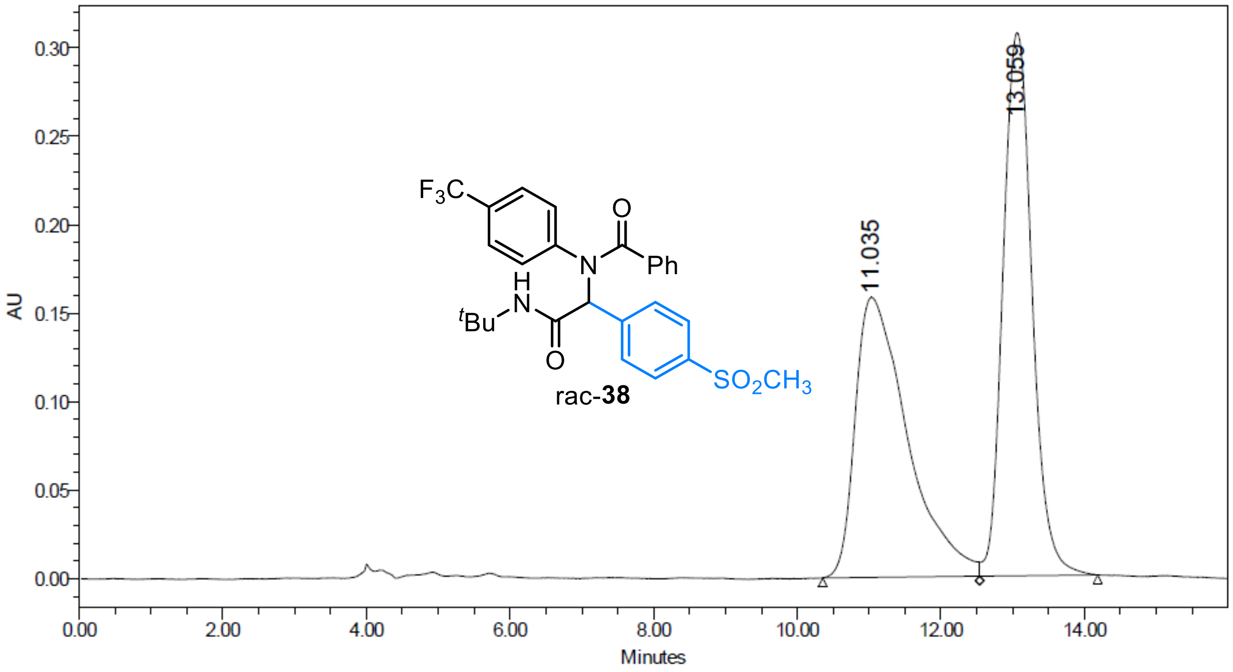

	RT (min)	Peak Type	Area (μV*sec)	% Area	Height (μV)	% Height	Integration Type	Points Across Peak	Start Time (min)	End Time (min)
1	11.035	Unknown	8000155	48.11	158235	34.02	BV	1311	10.355	12.540
2	13.059	Unknown	8628136	51.89	306849	65.98	VB	987	12.540	14.185

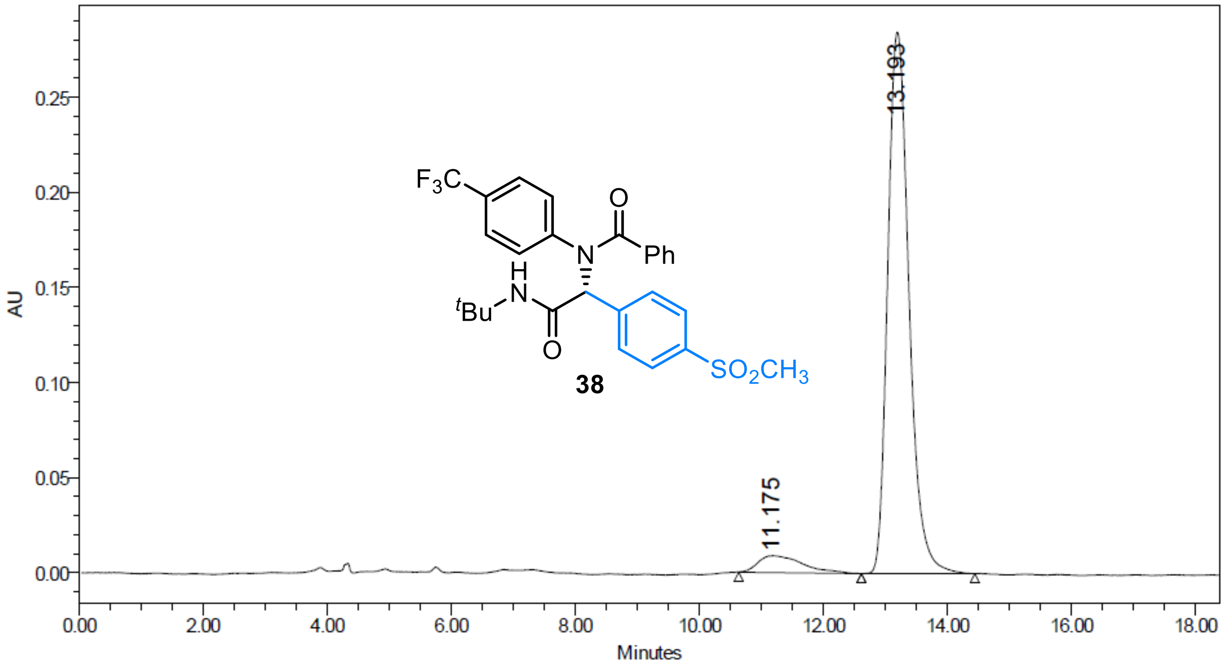

	RT (min)	Peak Type	Area (μV*sec)	% Area	Height (μV)	% Height	Integration Type	Points Across Peak	Start Time (min)	End Time (min)
1	11.175	Unknown	445847	6.18	8879	3.03	BB	1186	10.635	12.612
2	13.193	Unknown	6774291	93.82	284589	96.97	BB	1098	12.612	14.442

Supplementary Fig. 142. HPLC of product **38**.

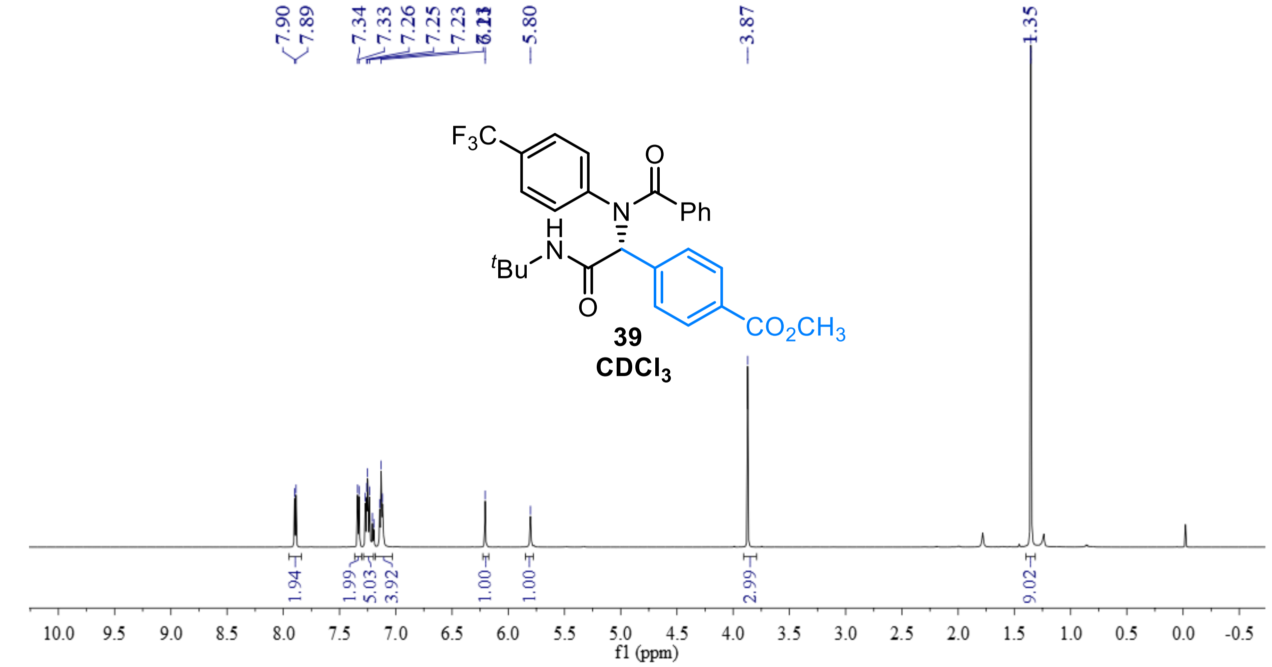

Supplementary Fig. 143. ^{1}H NMR spectrum of **39**. The sample has been recorded in 600 MHz, $CDCl_3$ at 25 °C.

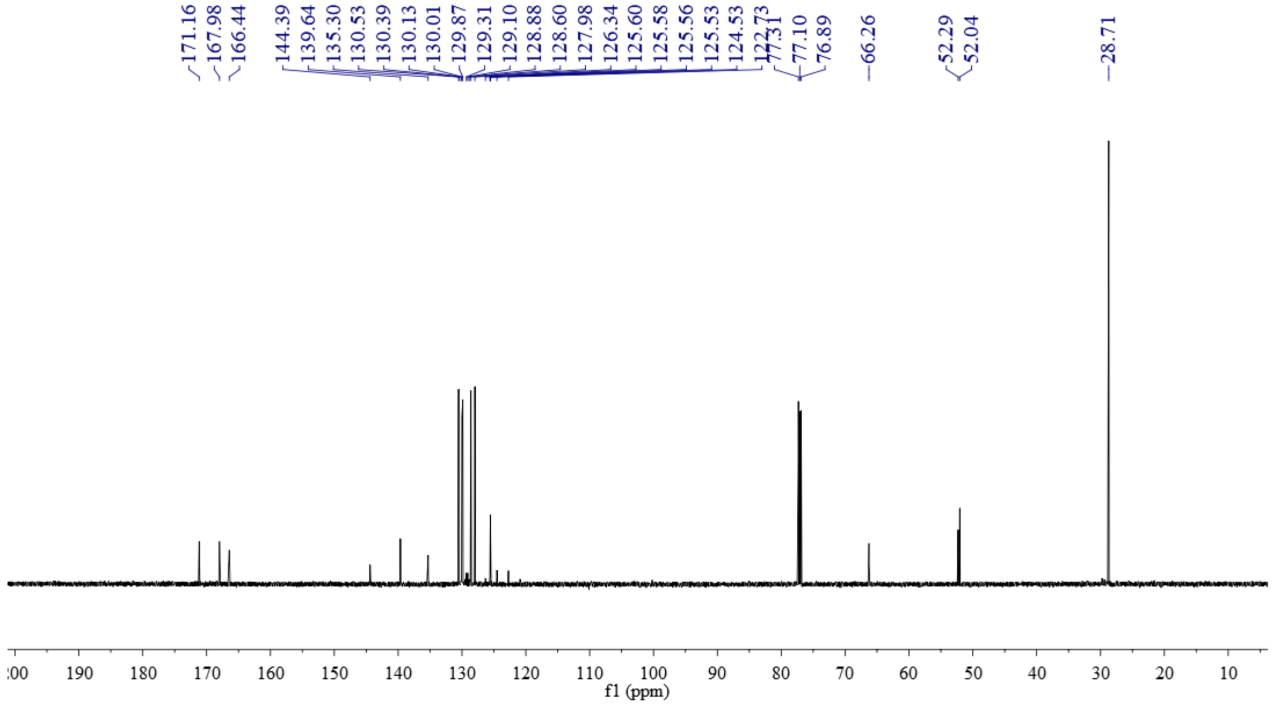

Supplementary Fig. 144. ^{13}C NMR spectrum of **39**. The sample has been recorded in 151 MHz, $CDCl_3$ at 25 °C.

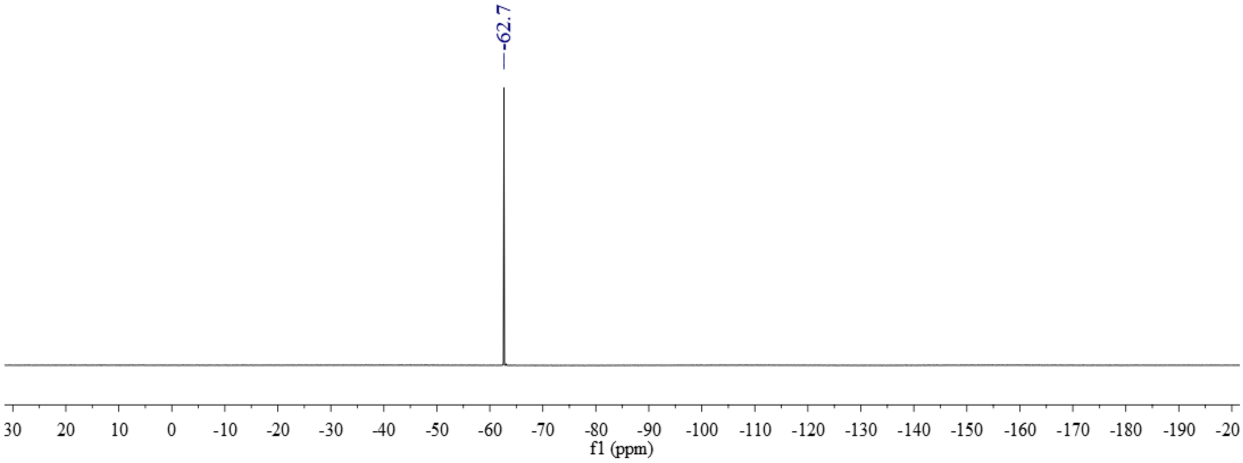

Supplementary Fig. 145. ^{31}F NMR spectrum of **39**. The sample has been recorded in 564 MHz, $CDCl_3$ at 25 °C.

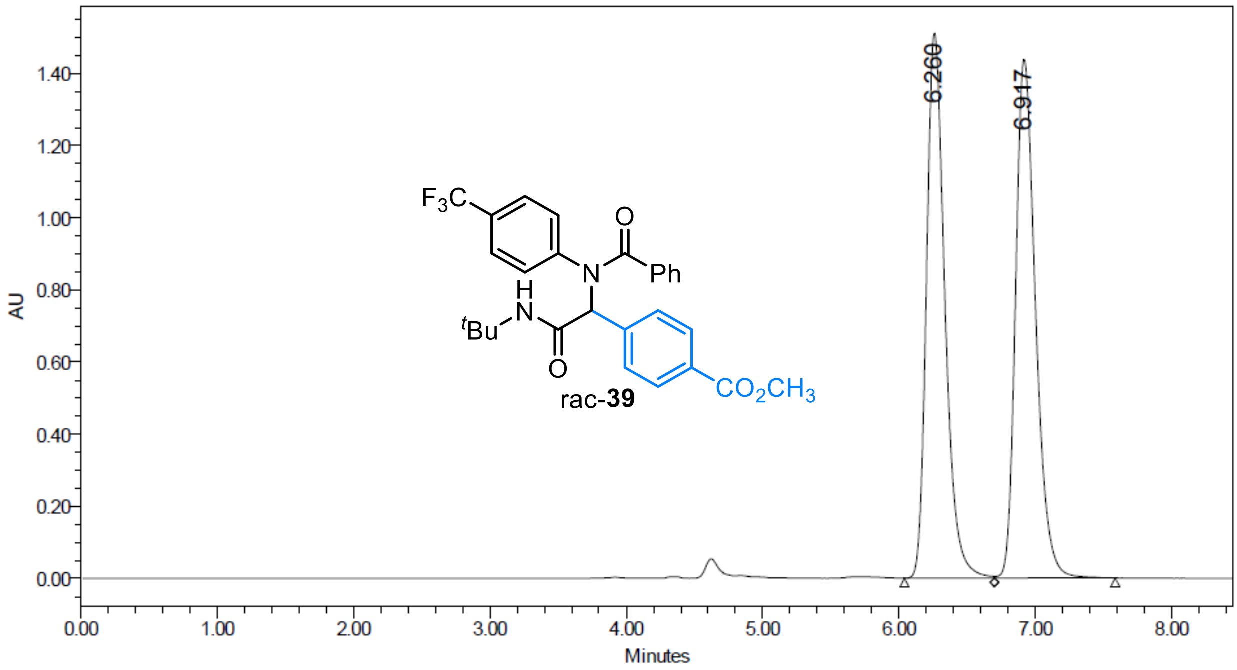

	RT (min)	Peak Type	Area (µV*sec)	% Area	Height (µV)	% Height	Integration Type	Points Across Peak	Start Time (min)	End Time (min)
1	6.260	Unknown	14188559	49.30	1510346	51.24	BV	396	6.040	6.700
2	6.917	Unknown	14591774	50.70	1437115	48.76	VB	531	6.700	7.585

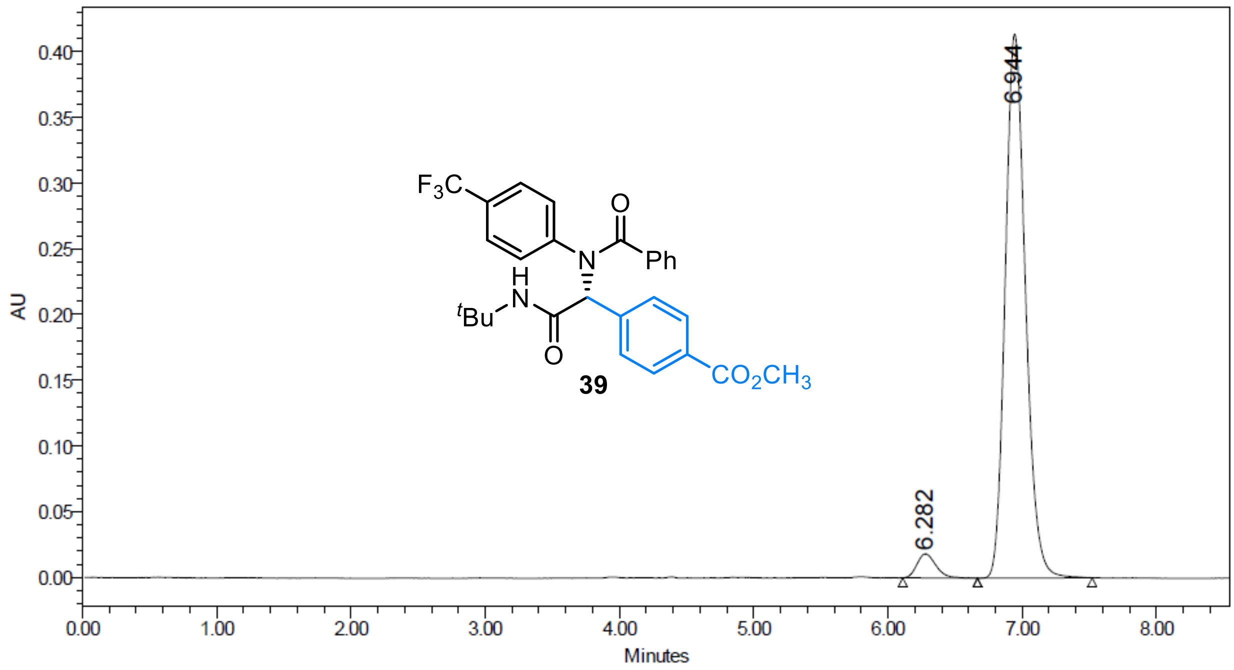

	RT (min)	Peak Type	Area (µV*sec)	% Area	Height (µV)	% Height	Integration Type	Points Across Peak	Start Time (min)	End Time (min)
1	6.282	Unknown	180145	4.00	18369	4.25	BB	334	6.110	6.667
2	6.944	Unknown	4324107	96.00	413821	95.75	BB	513	6.667	7.522

Supplementary Fig. 146. HPLC of product **39**.

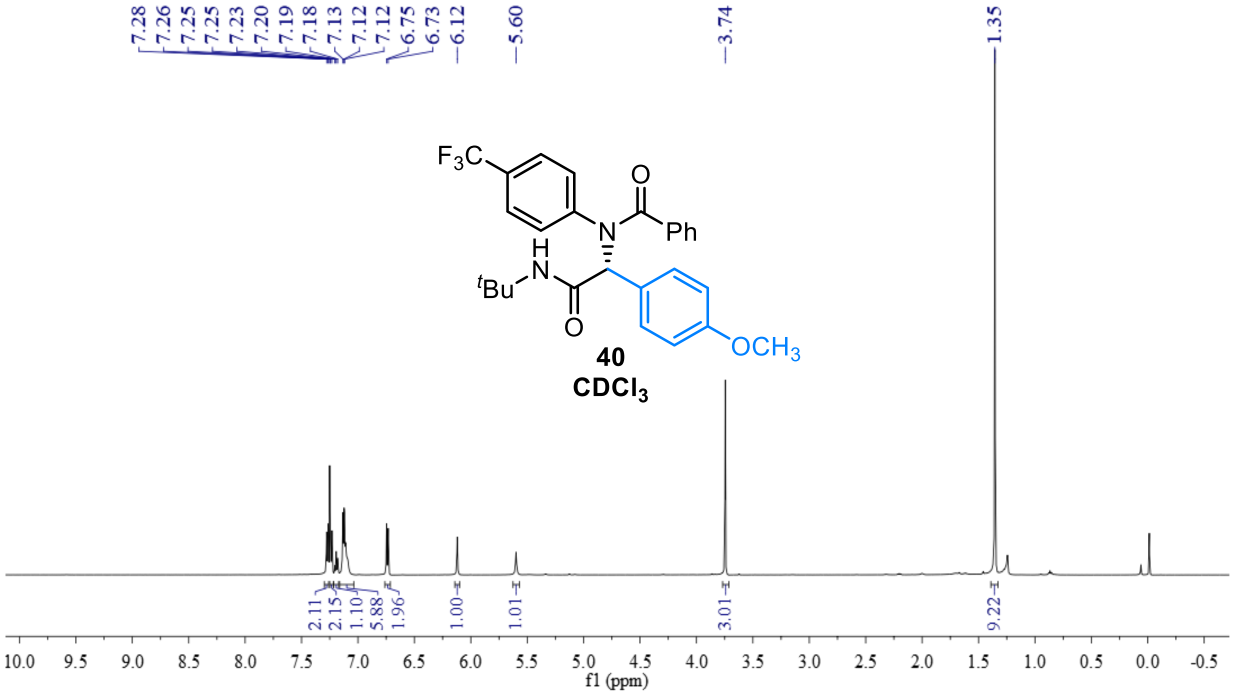

Supplementary Fig. 147. ^{1}H NMR spectrum of **40**. The sample has been recorded in 600 MHz, $CDCl_3$ at 25 °C.

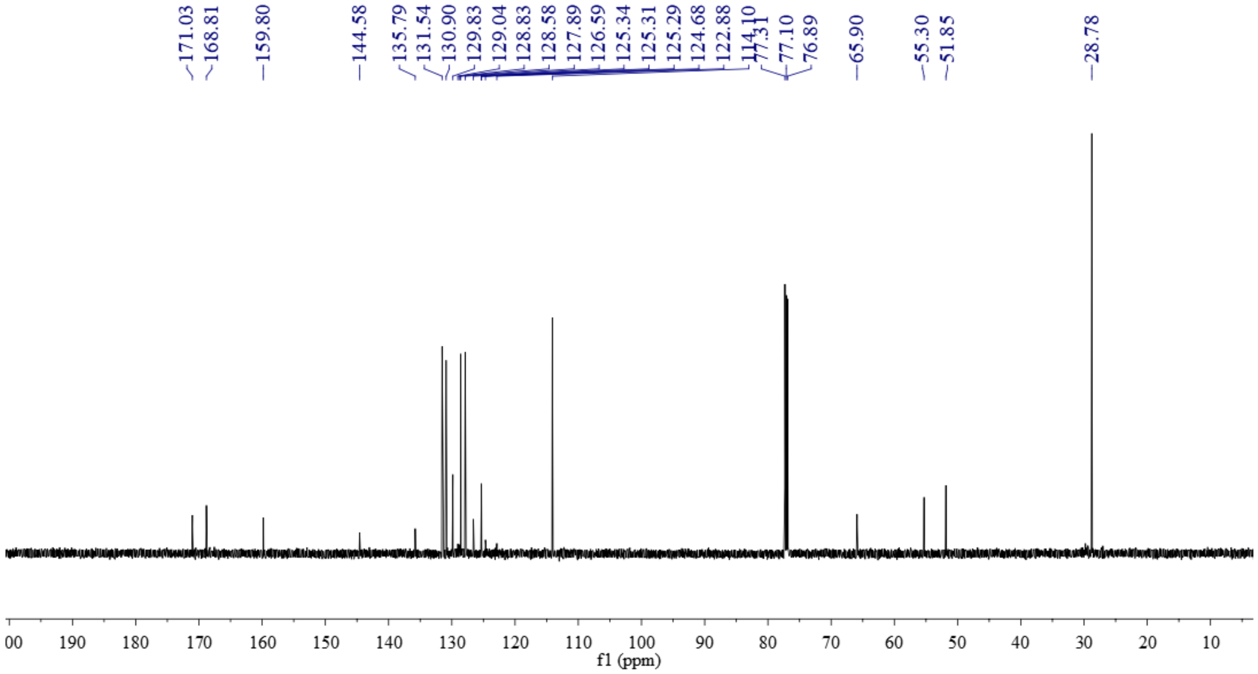

Supplementary Fig. 148. ^{13}C NMR spectrum of **40**. The sample has been recorded in 151 MHz, $CDCl_3$ at 25 °C.

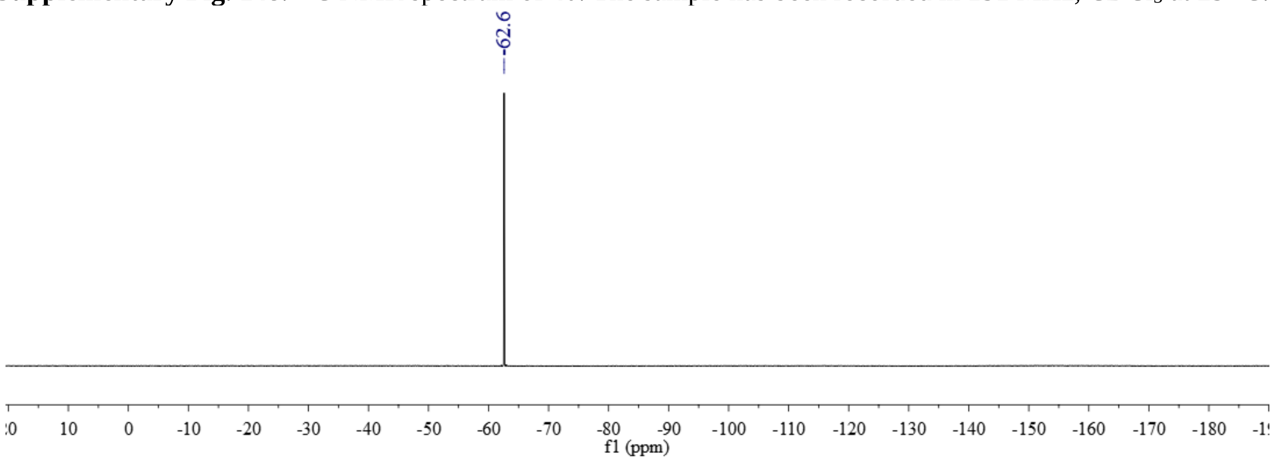

Supplementary Fig. 149. ^{31}F NMR spectrum of **40**. The sample has been recorded in 564 MHz, $CDCl_3$ at 25 °C.

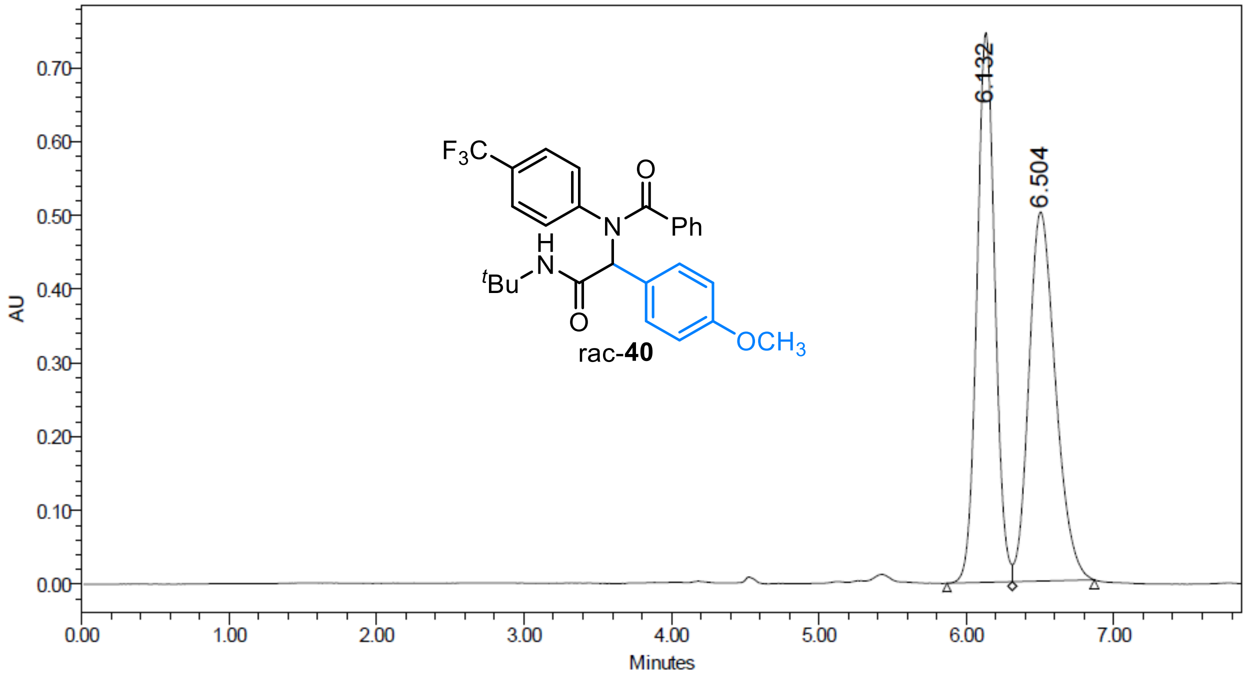

	RT (min)	Peak Type	Area (µV*sec)	% Area	Height (µV)	% Height	Integration Type	Points Across Peak	Start Time (min)	End Time (min)
1	6.132	Unknown	6301383	50.00	744996	59.83	BV	266	5.868	6.312
2	6.504	Unknown	6301768	50.00	500223	40.17	VB	334	6.312	6.868

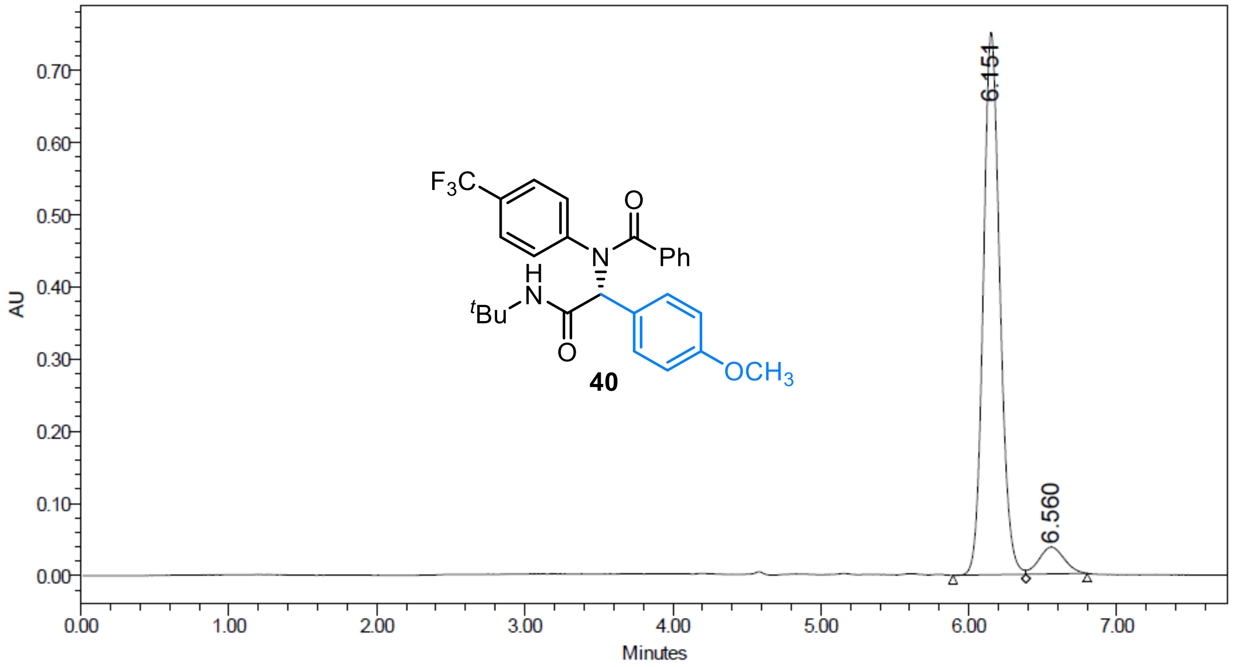

	RT (min)	Peak Type	Area (µV*sec)	% Area	Height (µV)	% Height	Integration Type	Points Across Peak	Start Time (min)	End Time (min)
1	6.151	Unknown	5847855	93.07	751363	95.30	bV	296	5.893	6.387
2	6.560	Unknown	435759	6.93	37047	4.70	Vb	248	6.387	6.800

Supplementary Fig. 150. HPLC of product **40**.

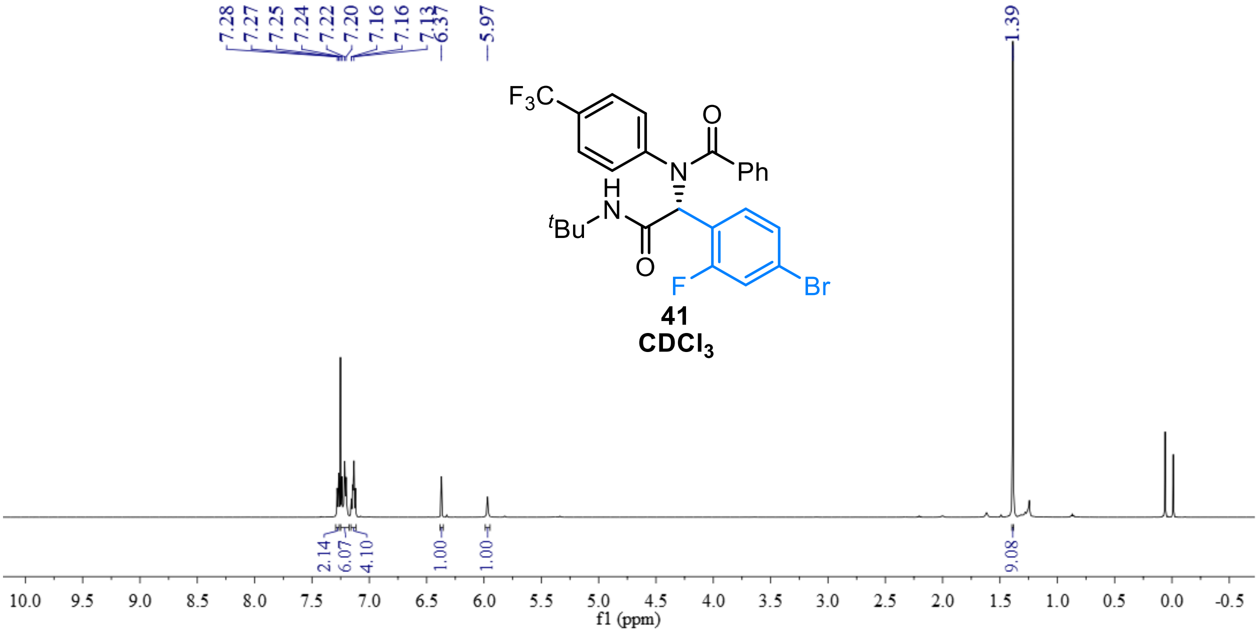

Supplementary Fig. 151. ^{1}H NMR spectrum of **41**. The sample has been recorded in 600 MHz, $CDCl_3$ at 25 °C.

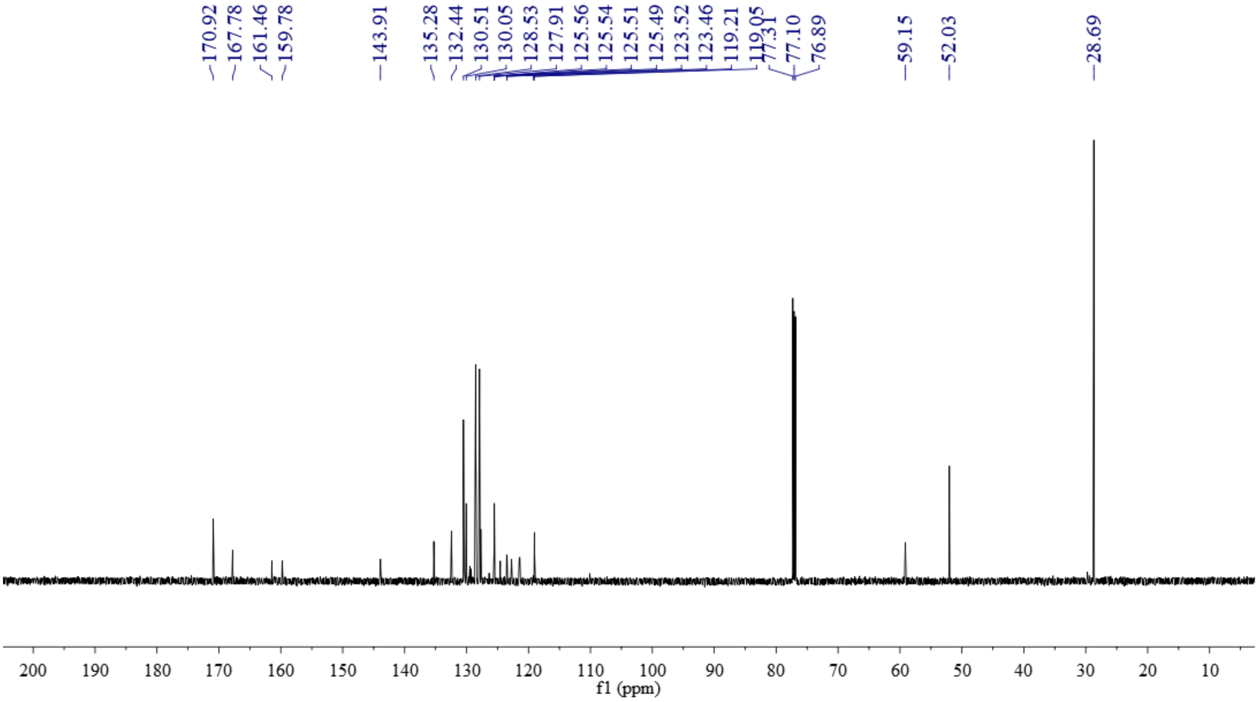

Supplementary Fig. 152. ^{13}C NMR spectrum of **41**. The sample has been recorded in 151 MHz, $CDCl_3$ at 25 °C.

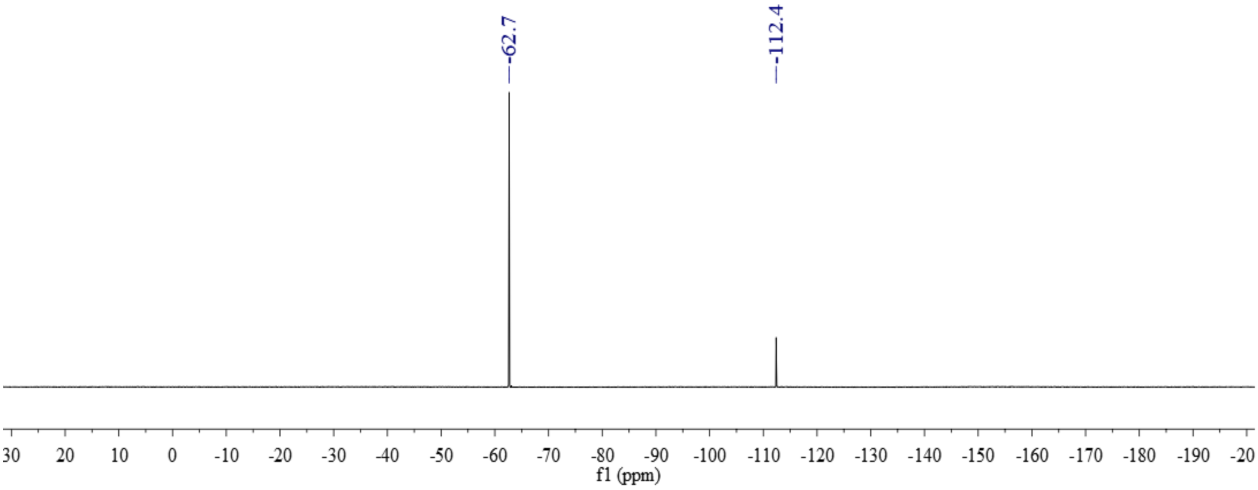

Supplementary Fig. 153. ^{31}F NMR spectrum of **41**. The sample has been recorded in 564 MHz, $CDCl_3$ at 25 °C.

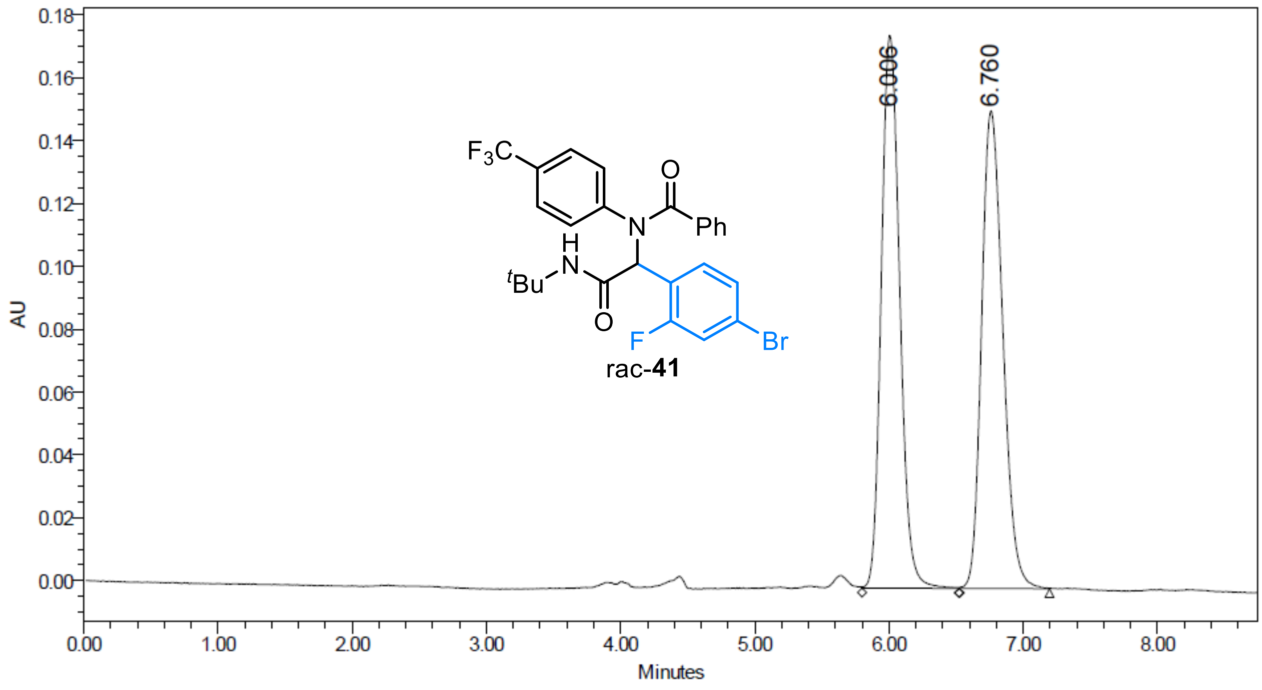

	RT (min)	Peak Type	Area (μV*sec)	% Area	Height (μV)	% Height	Integration Type	Points Across Peak	Start Time (min)	End Time (min)
1	6.006	Unknown	1676704	50.10	175985	53.65	VV	435	5.798	6.523
2	6.760	Unknown	1670236	49.90	152048	46.35	VB	403	6.523	7.195

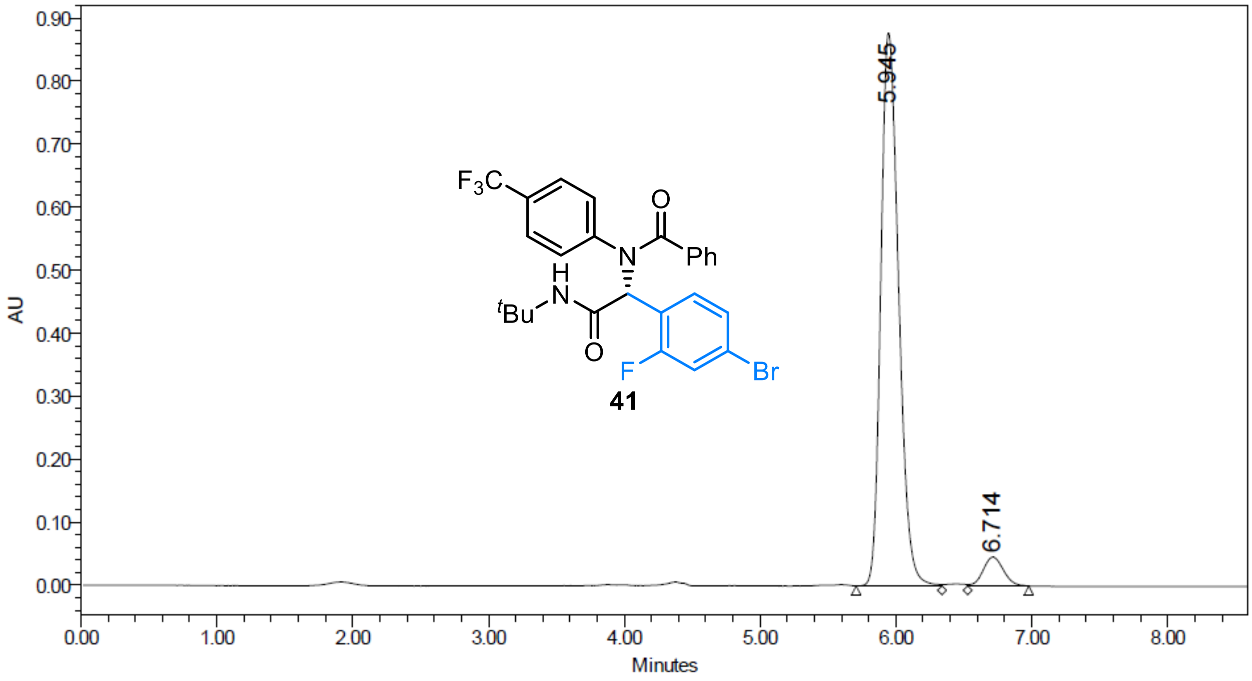

	RT (min)	Peak Type	Area (μV*sec)	% Area	Height (μV)	% Height	Integration Type	Points Across Peak	Start Time (min)	End Time (min)
1	5.945	Unknown	7988365	94.42	877926	95.05	BV	379	5.707	6.338
2	6.714	Unknown	472350	5.58	45735	4.95	Vb	270	6.528	6.978

Supplementary Fig. 154. HPLC of product **41**.

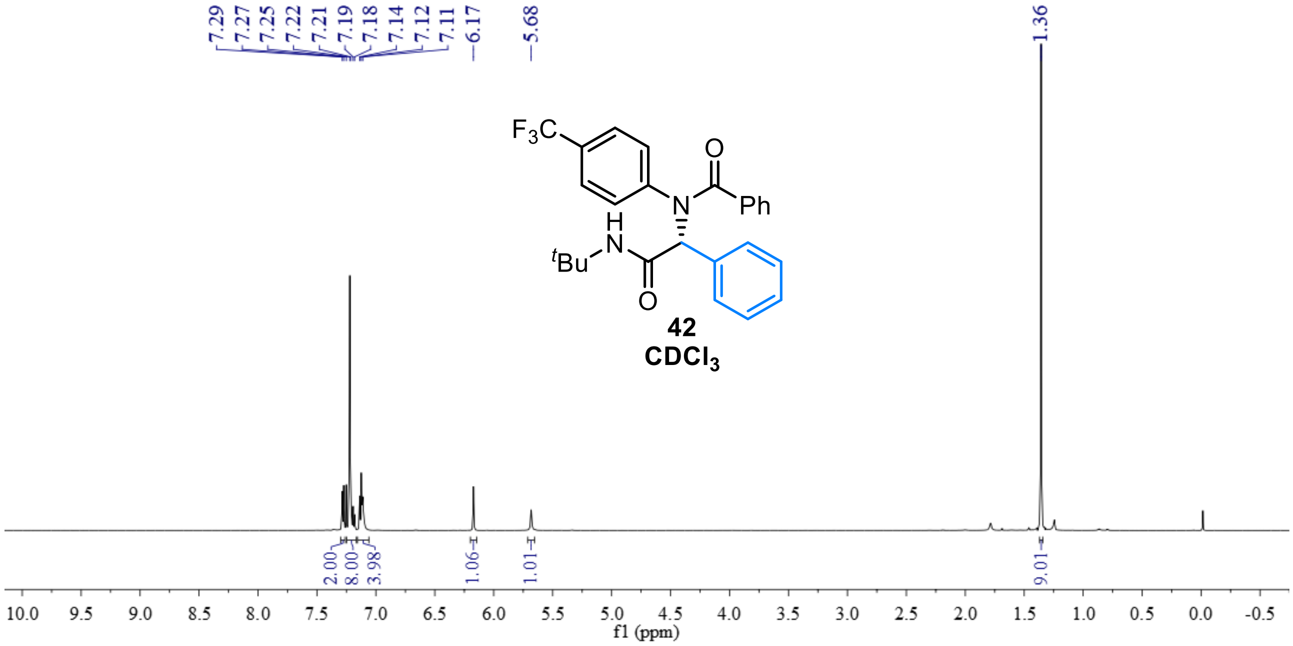

Supplementary Fig. 155. ^{1}H NMR spectrum of **42**. The sample has been recorded in 600 MHz, $CDCl_3$ at 25 °C.

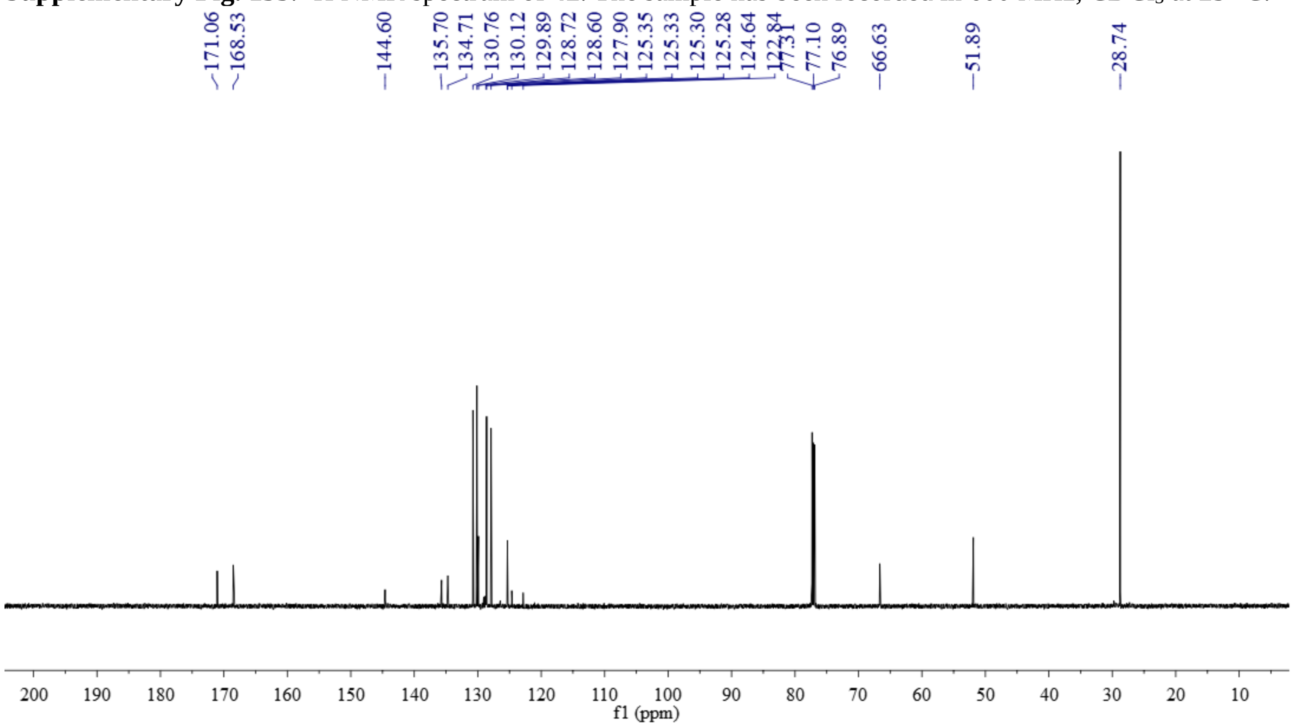

Supplementary Fig. 156. ^{13}C NMR spectrum of **42**. The sample has been recorded in 151 MHz, $CDCl_3$ at 25 °C.

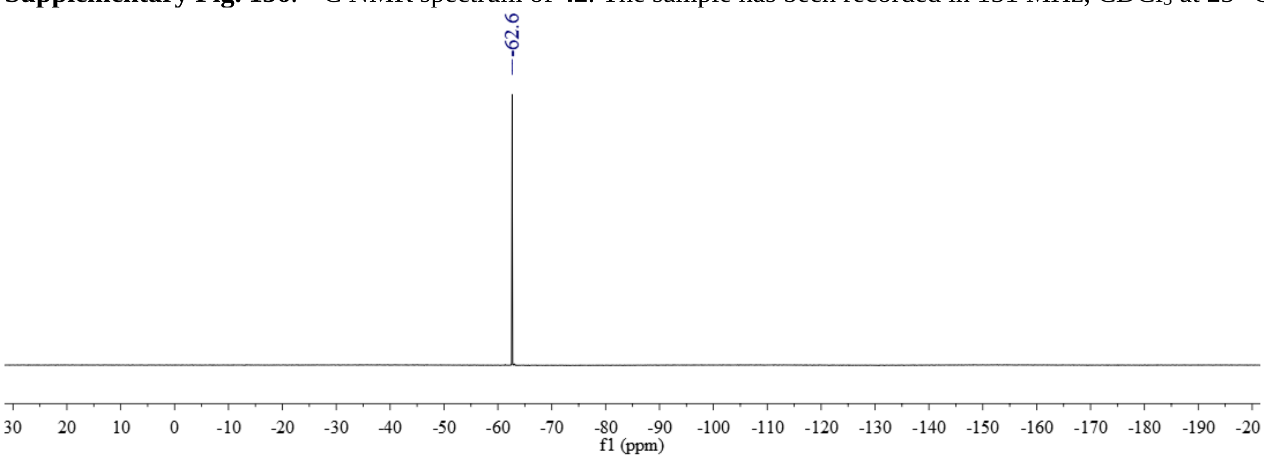

Supplementary Fig. 157. ^{31}F NMR spectrum of **42**. The sample has been recorded in 564 MHz, $CDCl_3$ at 25 °C.

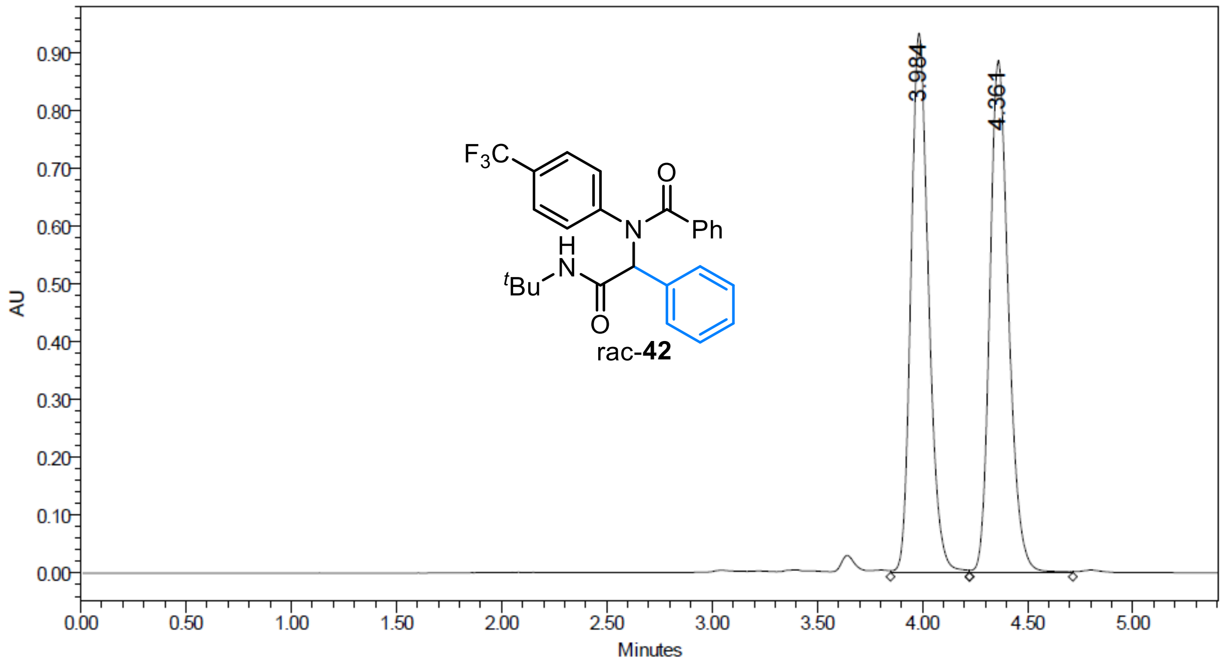

	RT (min)	Peak Type	Area (µV*sec)	% Area	Height (µV)	% Height	Integration Type	Points Across Peak	Start Time (min)	End Time (min)
1	3.984	Unknown	5422903	49.75	932629	51.31	VV	225	3.848	4.223
2	4.361	Unknown	5477904	50.25	885143	48.69	VV	295	4.223	4.715

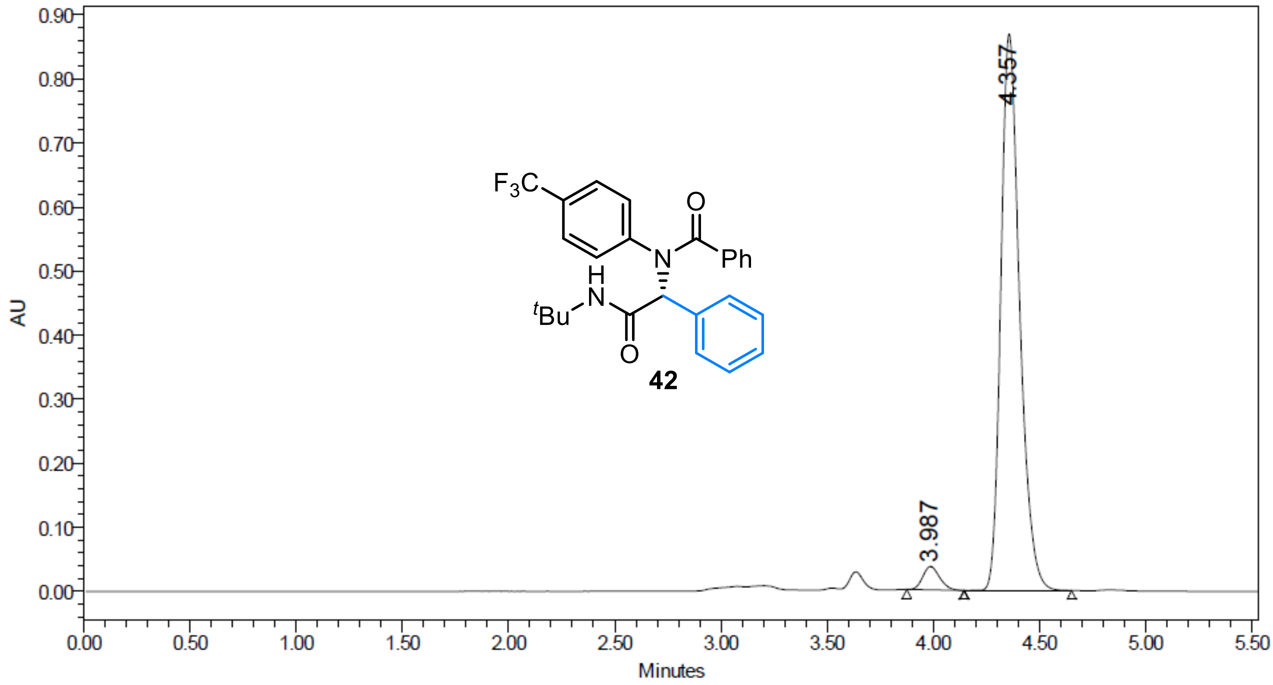

	RT (min)	Peak Type	Area (µV*sec)	% Area	Height (µV)	% Height	Integration Type	Points Across Peak	Start Time (min)	End Time (min)
1	3.987	Unknown	201584	3.64	36560	4.04	Bb	161	3.875	4.143
2	4.357	Unknown	5342349	96.36	868865	95.96	BB	303	4.147	4.652

Supplementary Fig. 158. HPLC of product **42**.

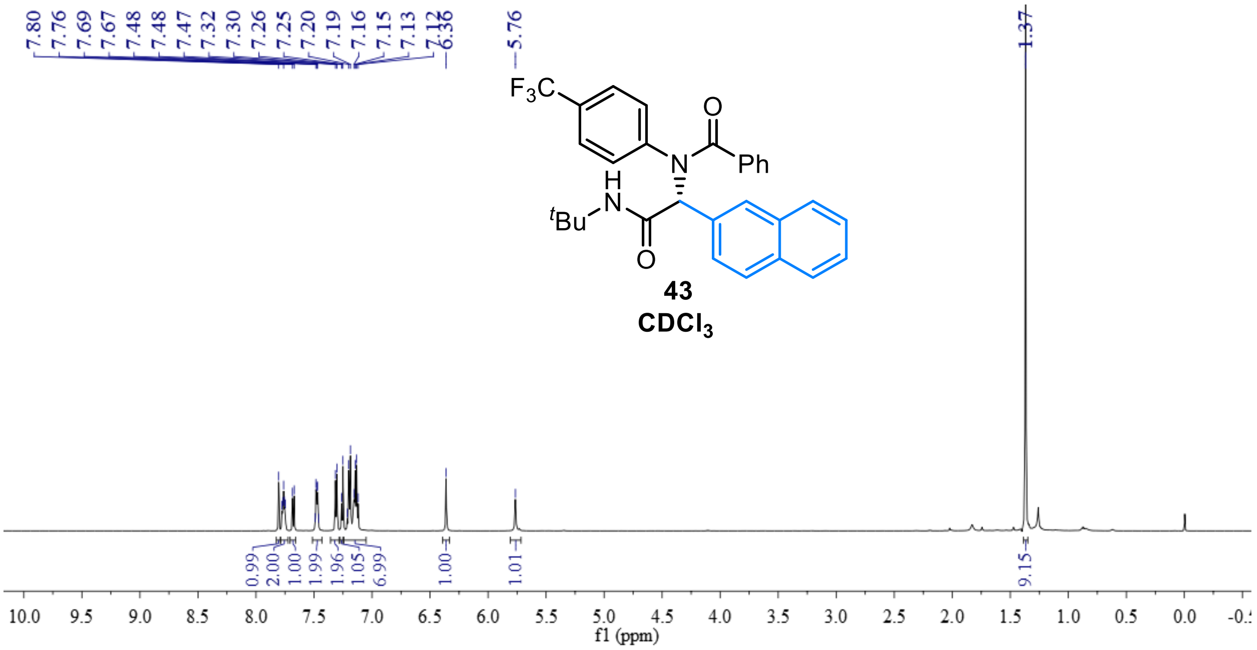

Supplementary Fig. 159. ^{1}H NMR spectrum of **43**. The sample has been recorded in 600 MHz, $CDCl_3$ at 25 °C.

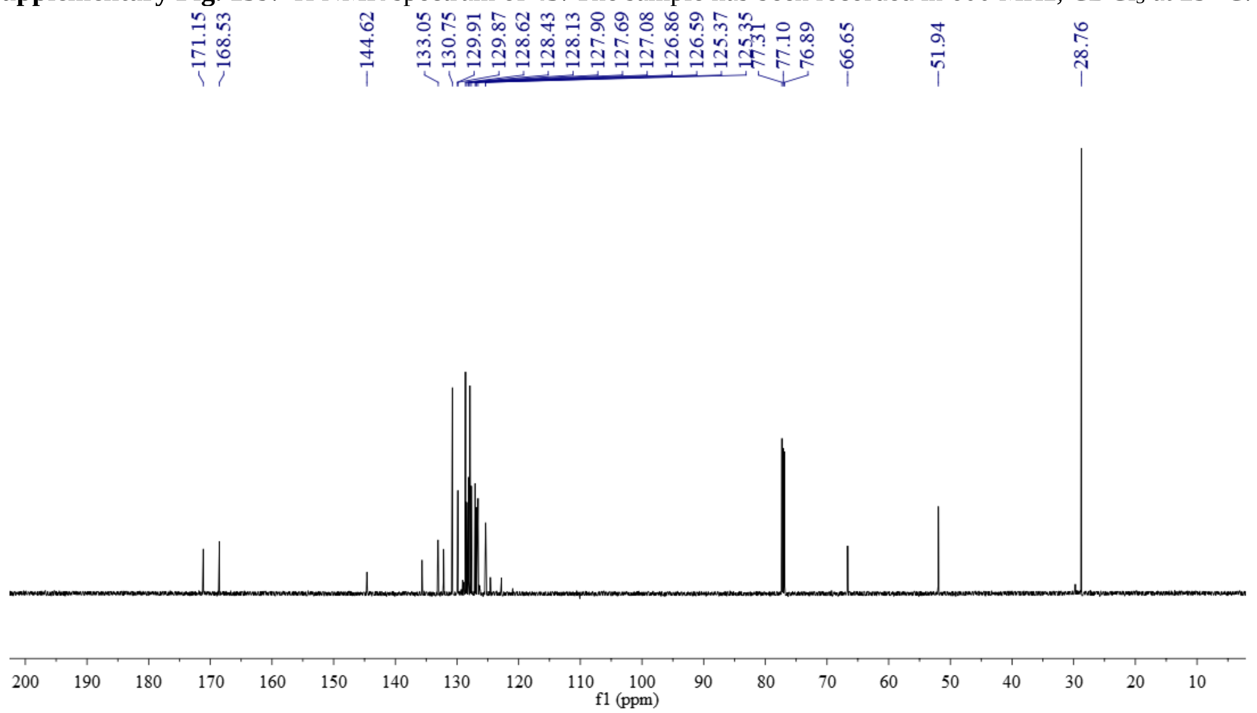

Supplementary Fig. 160. ^{13}C NMR spectrum of **43**. The sample has been recorded in 151 MHz, $CDCl_3$ at 25 °C.

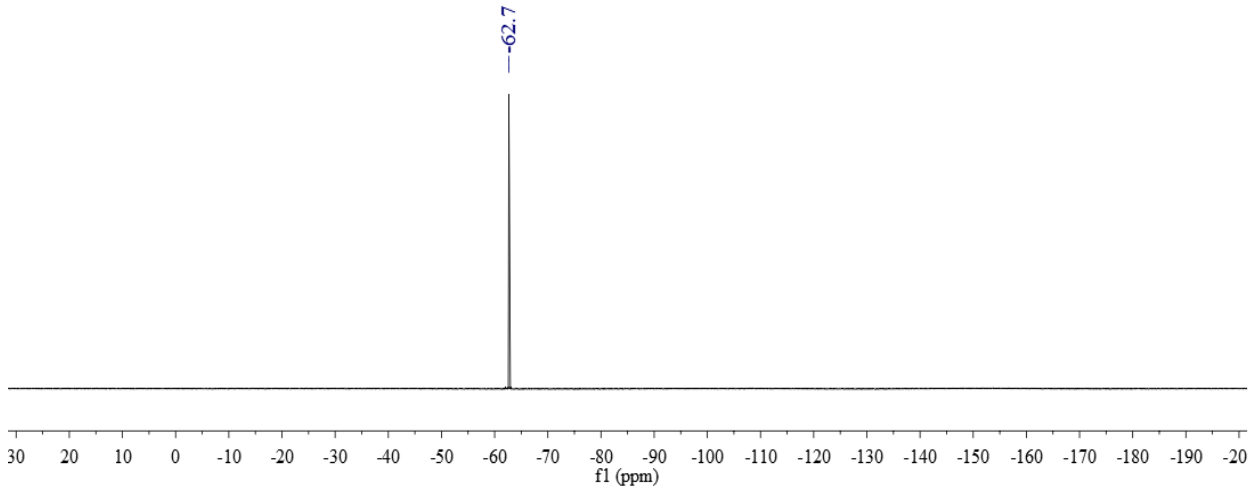

Supplementary Fig. 161. [31]F NMR spectrum of **43**. The sample has been recorded in 564 MHz, $CDCl_3$ at 25 °C.

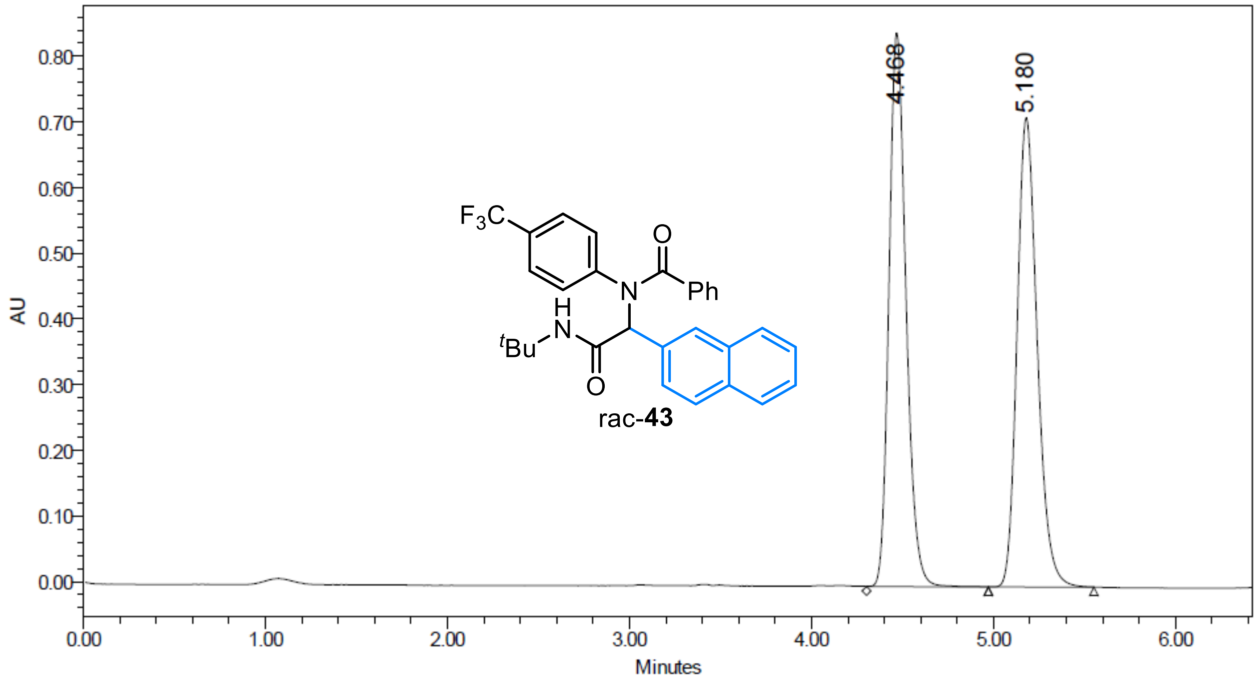

	RT (min)	Peak Type	Area (µV*sec)	% Area	Height (µV)	% Height	Integration Type	Points Across Peak	Start Time (min)	End Time (min)
1	4.468	Unknown	5470373	49.88	843192	54.12	VB	402	4.302	4.972
2	5.180	Unknown	5496823	50.12	714743	45.88	BB	348	4.972	5.552

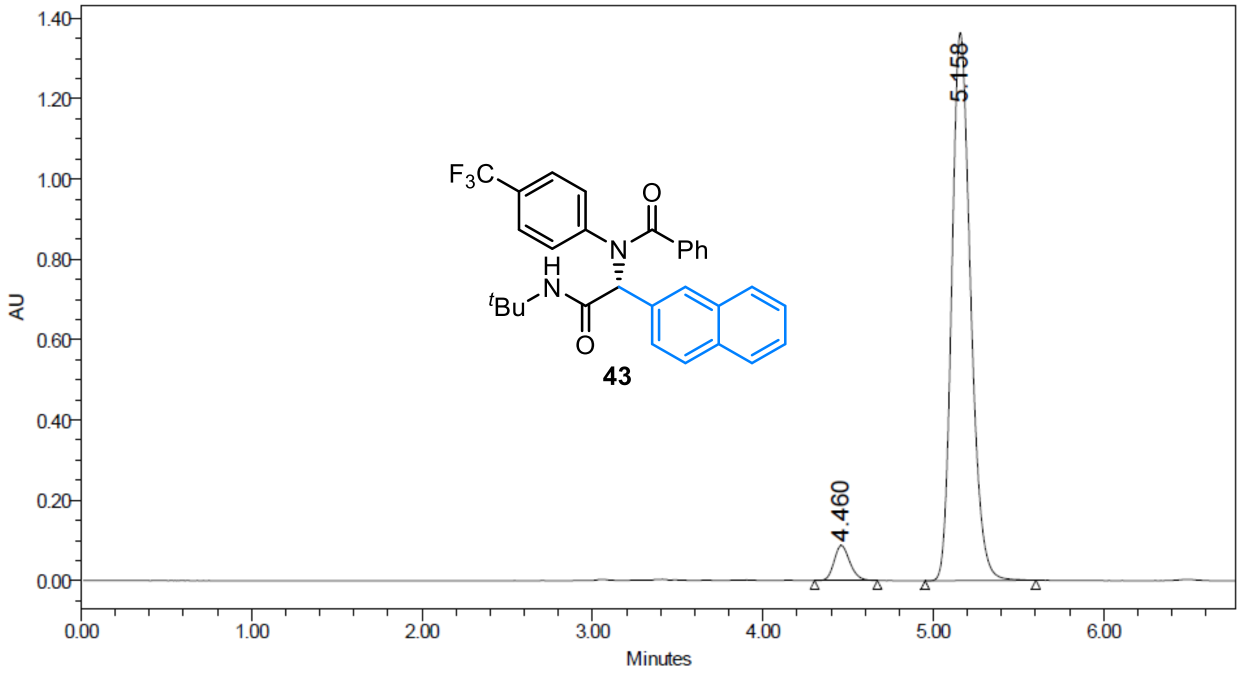

	RT (min)	Peak Type	Area (µV*sec)	% Area	Height (µV)	% Height	Integration Type	Points Across Peak	Start Time (min)	End Time (min)
1	4.460	Unknown	562028	5.10	87478	6.02	Bb	221	4.303	4.672
2	5.158	Unknown	10454752	94.90	1365130	93.98	BB	390	4.952	5.602

Supplementary Fig. 162. HPLC of product **43**.

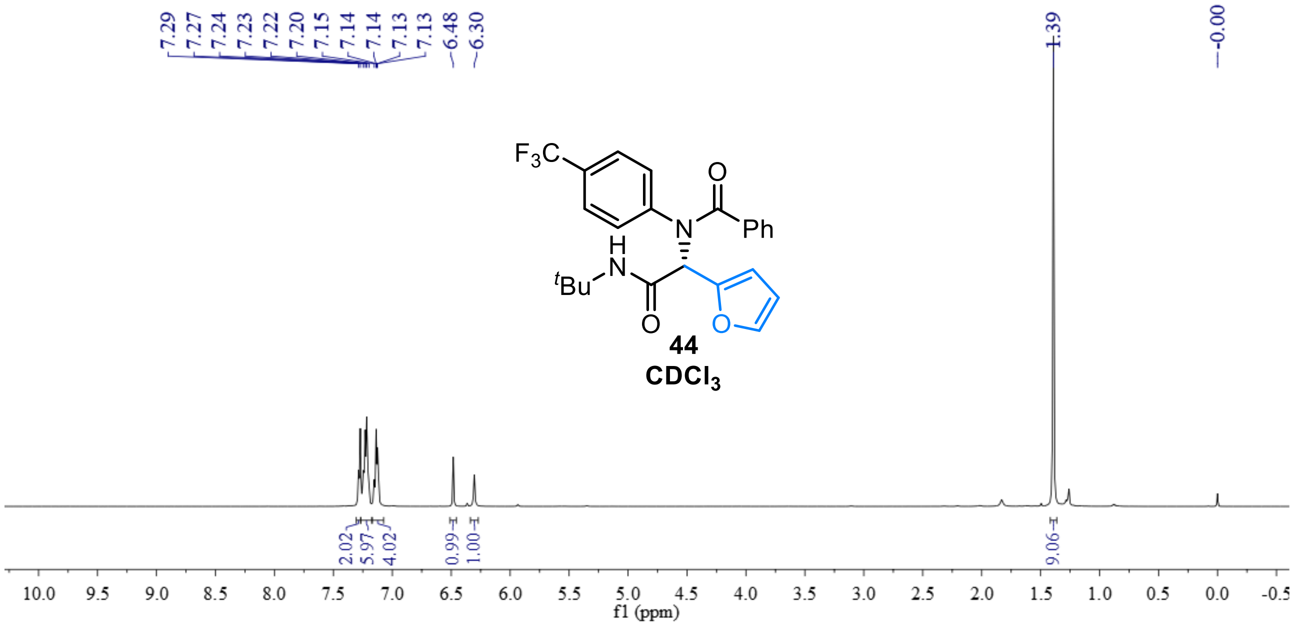

Supplementary Fig. 163. ^{1}H NMR spectrum of **44**. The sample has been recorded in 600 MHz, $CDCl_3$ at 25 °C.

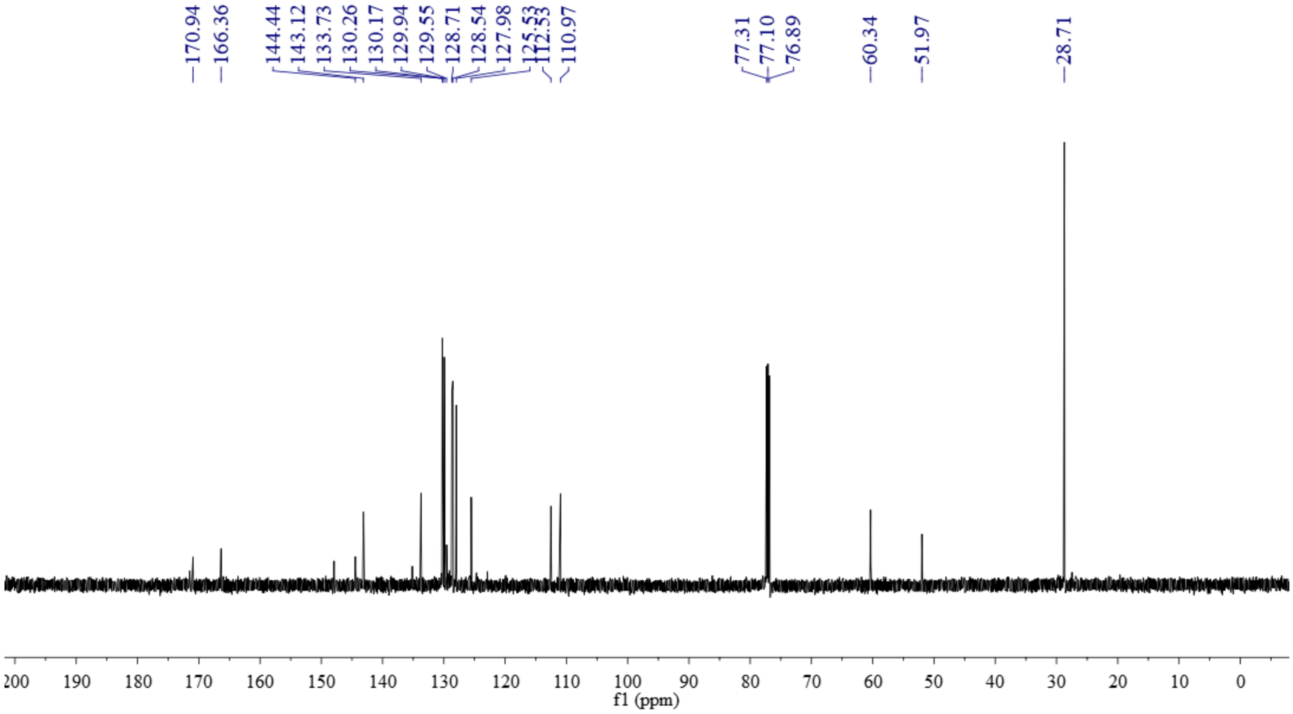

Supplementary Fig. 164. ^{13}C NMR spectrum of **44**. The sample has been recorded in 151 MHz, $CDCl_3$ at 25 °C.

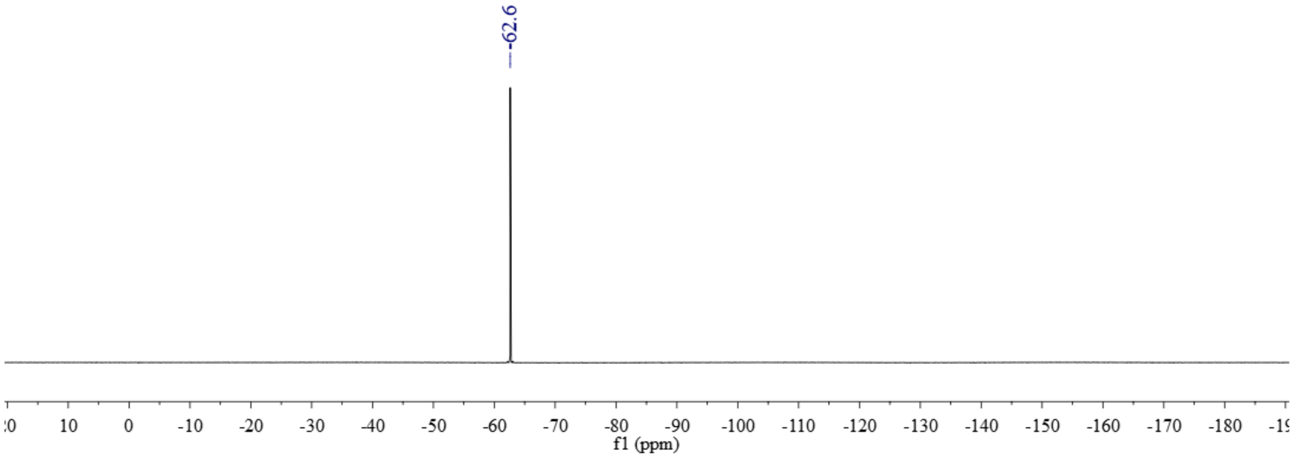

Supplementary Fig. 165. ^{31}F NMR spectrum of **44**. The sample has been recorded in 564 MHz, $CDCl_3$ at 25 °C.

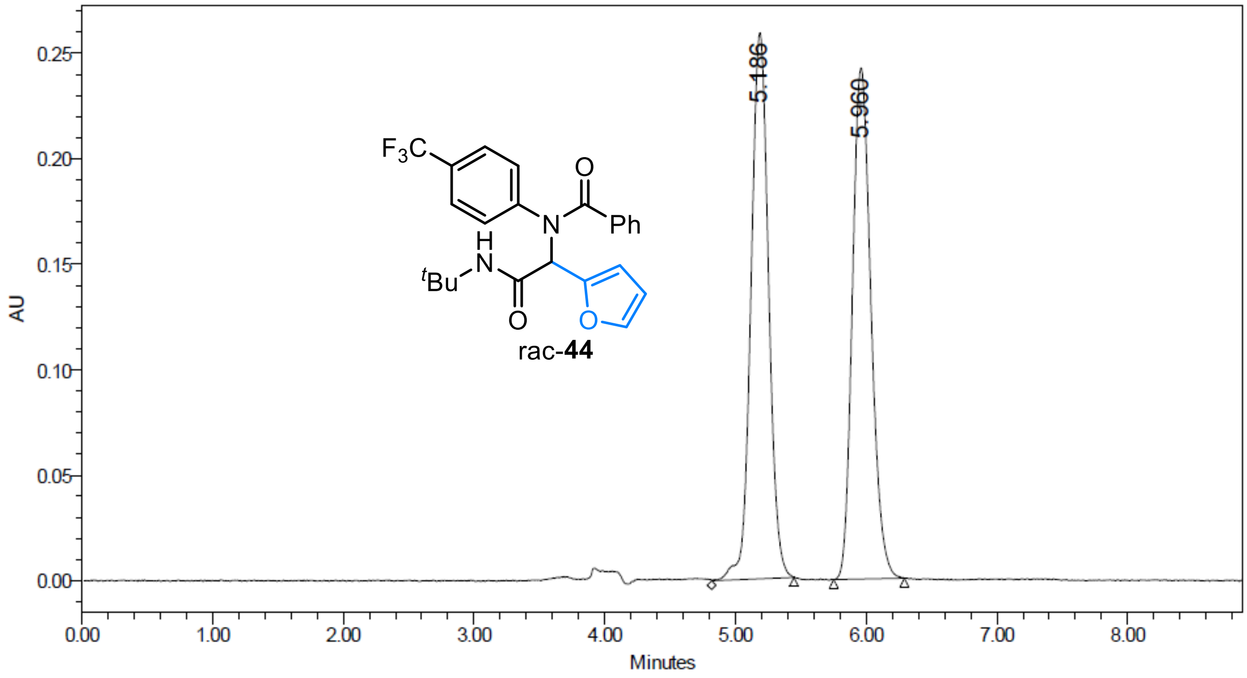

	RT (min)	Peak Type	Area (µV*sec)	% Area	Height (µV)	% Height	Integration Type	Points Across Peak	Start Time (min)	End Time (min)
1	5.186	Unknown	2424094	50.37	258892	51.65	Vb	378	4.817	5.447
2	5.960	Unknown	2388325	49.63	242307	48.35	BB	324	5.752	6.292

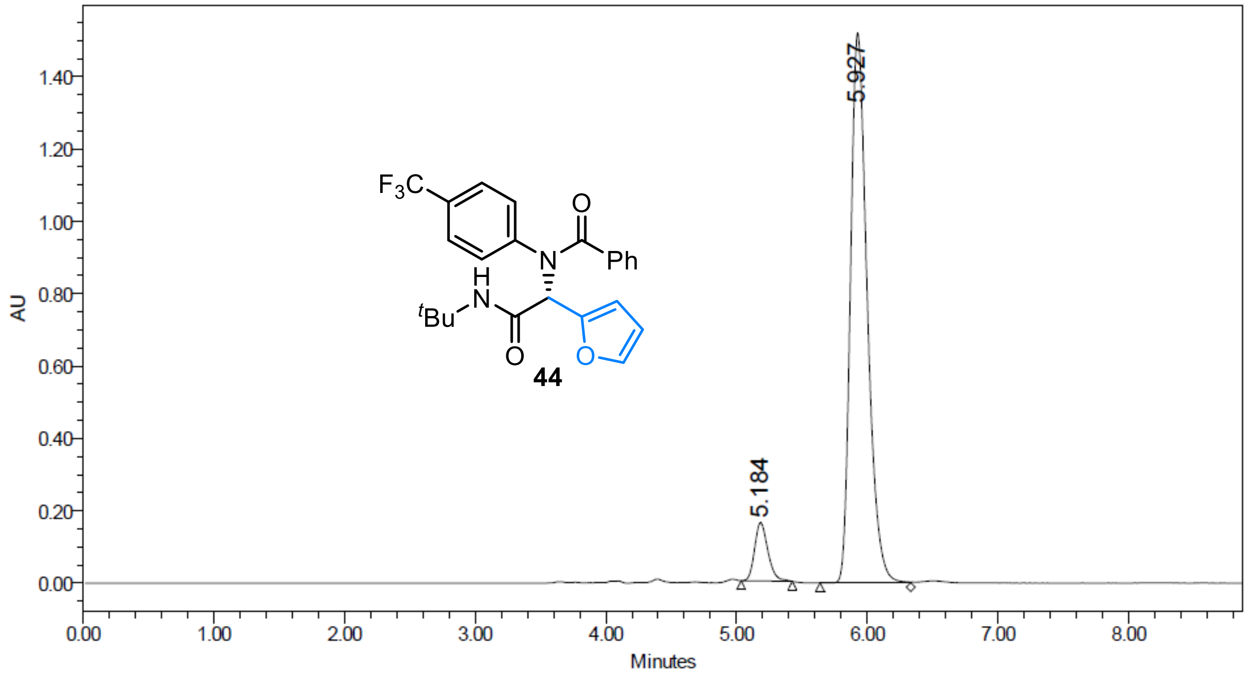

	RT (min)	Peak Type	Area (µV*sec)	% Area	Height (µV)	% Height	Integration Type	Points Across Peak	Start Time (min)	End Time (min)
1	5.184	Unknown	1136912	7.77	161286	9.59	bb	235	5.035	5.427
2	5.927	Unknown	13503437	92.23	1519837	90.41	bV	417	5.638	6.333

Supplementary Fig. 166. HPLC of product **44**.

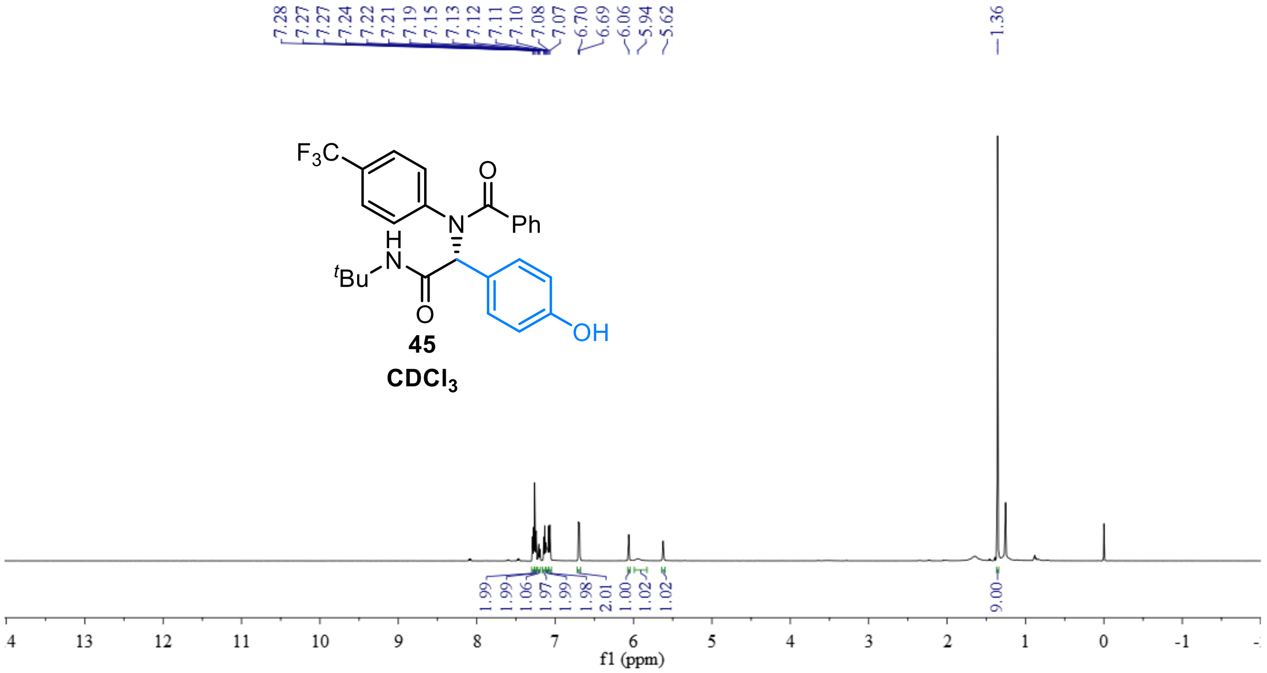

Supplementary Fig. 167. ^{1}H NMR spectrum of **45**. The sample has been recorded in 600 MHz, $CDCl_3$ at 25 °C.

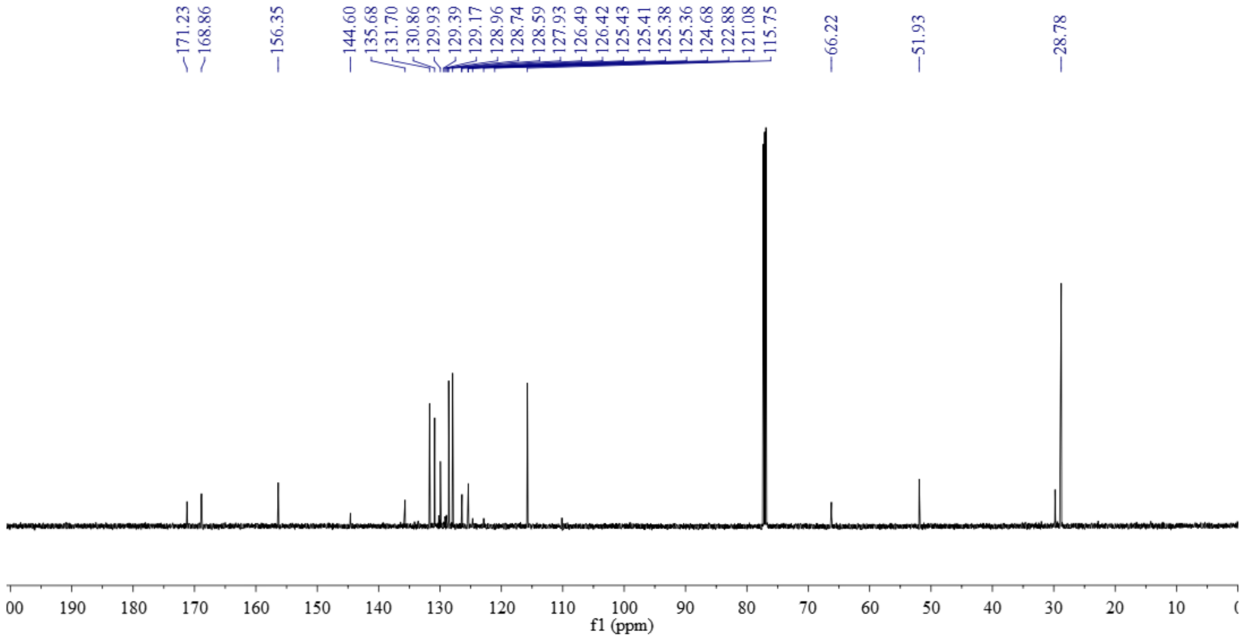

Supplementary Fig. 168. ^{13}C NMR spectrum of **45**. The sample has been recorded in 151 MHz, $CDCl_3$ at 25 °C.

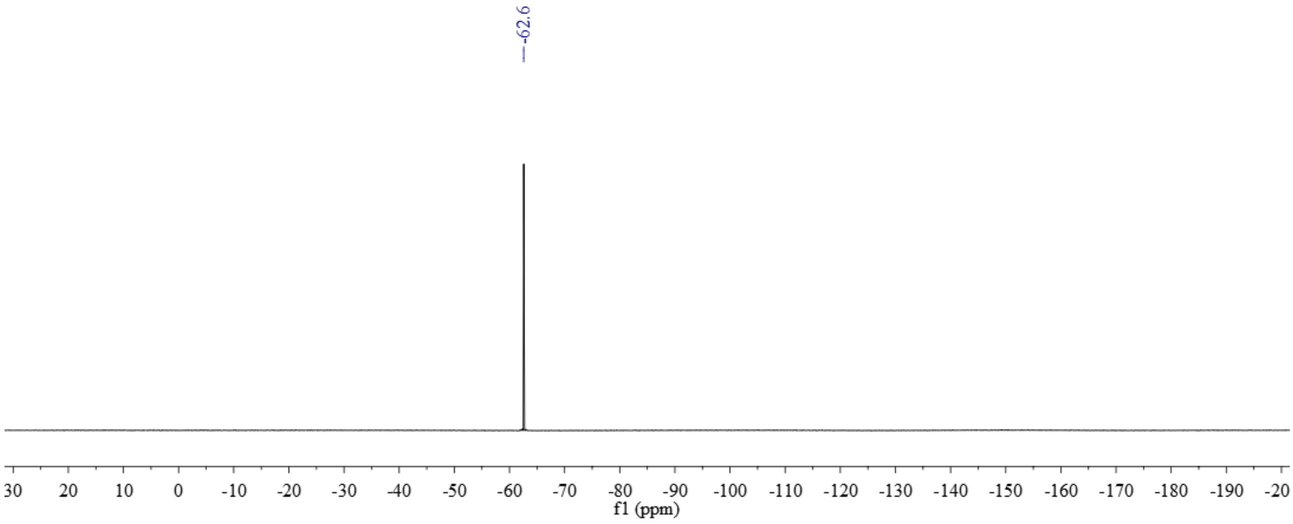

Supplementary Fig. 169. ^{31}F NMR spectrum of **45**. The sample has been recorded in 564 MHz, $CDCl_3$ at 25 °C.

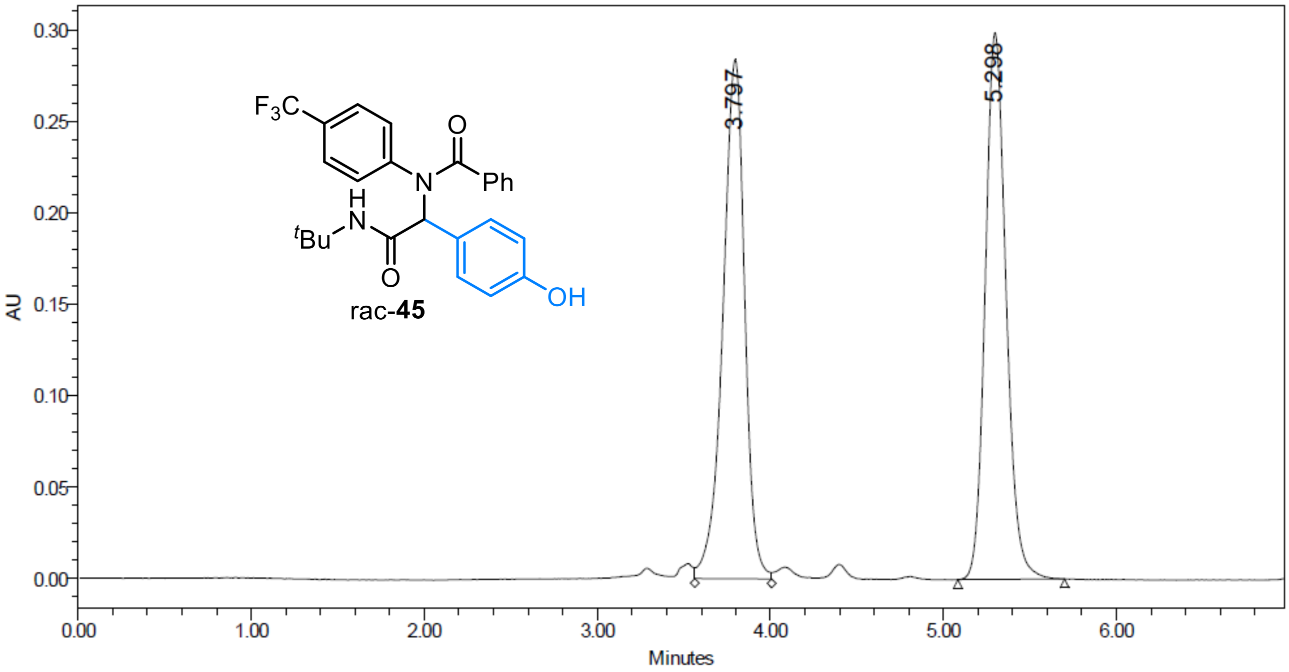

	RT (min)	Area (µV*sec)	% Area	Height (µV)	% Height
1	3.797	2489839	49.88	284029	48.72
2	5.298	2502166	50.12	298960	51.28

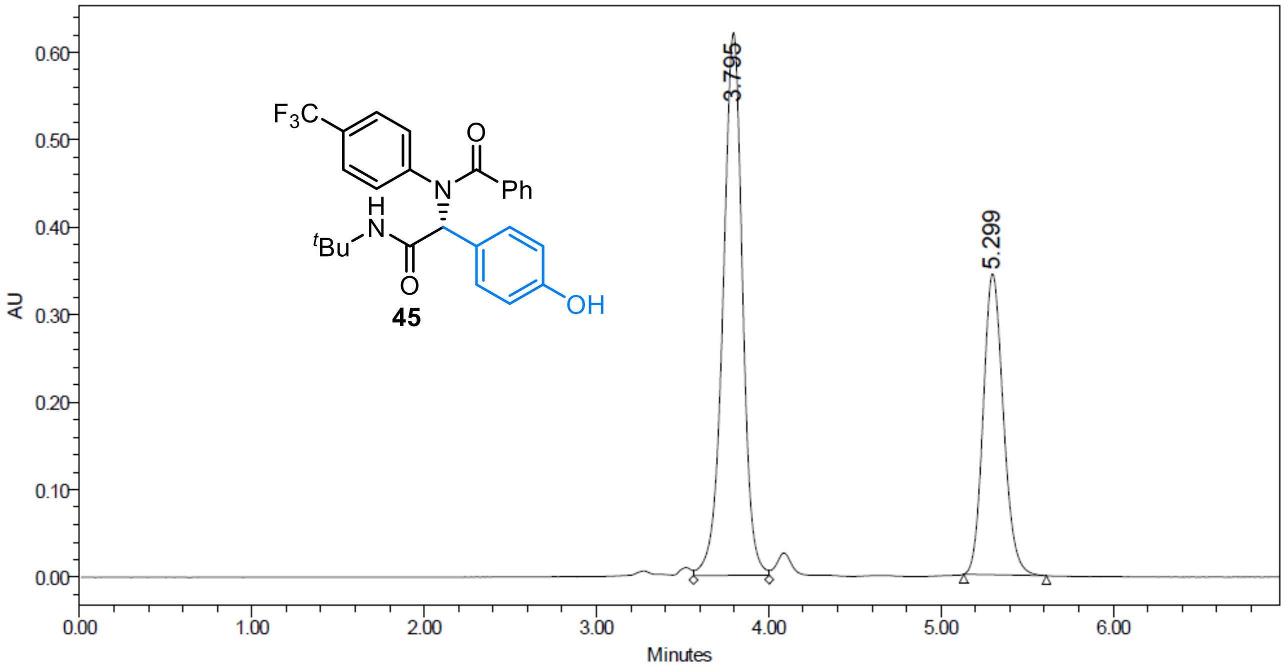

	RT (min)	Area (µV*sec)	% Area	Height (µV)	% Height
1	3.795	4869602	63.50	620442	64.34
2	5.299	2799331	36.50	343878	35.66

Supplementary Fig. 170. HPLC of product **45**.

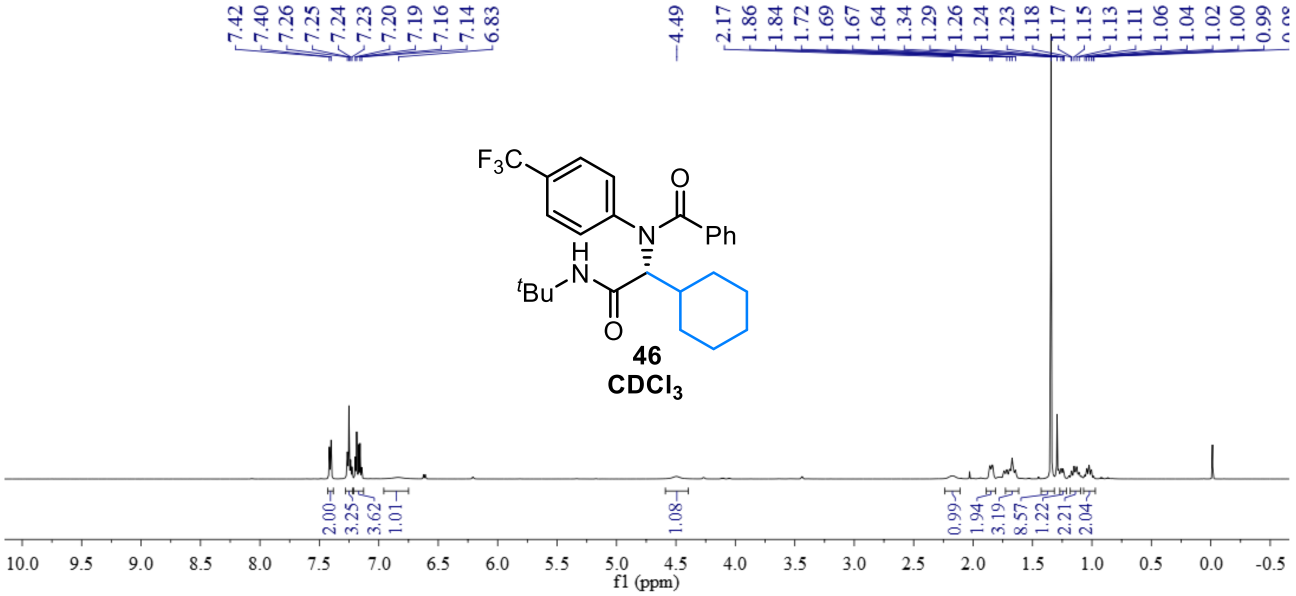

Supplementary Fig. 171. ^{1}H NMR spectrum of **46**. The sample has been recorded in 600 MHz, $CDCl_3$ at 25 °C.

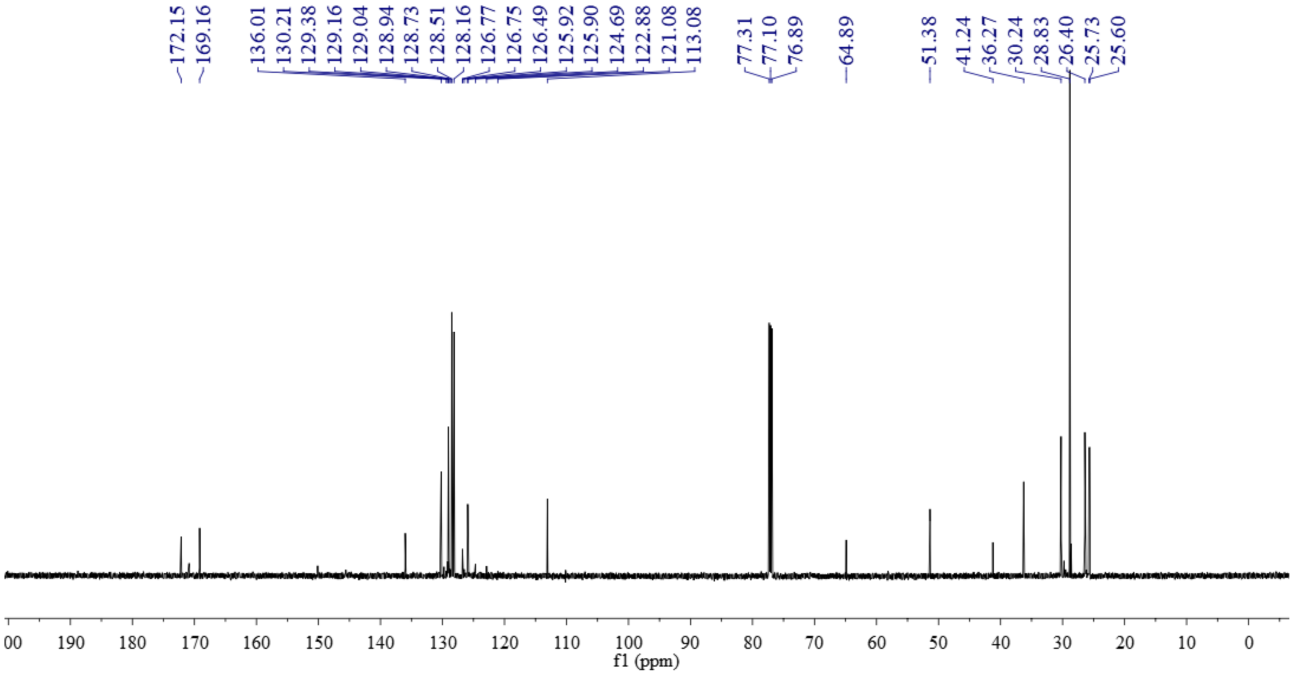

Supplementary Fig. 172. ^{13}C NMR spectrum of **46**. The sample has been recorded in 151 MHz, $CDCl_3$ at 25 °C.

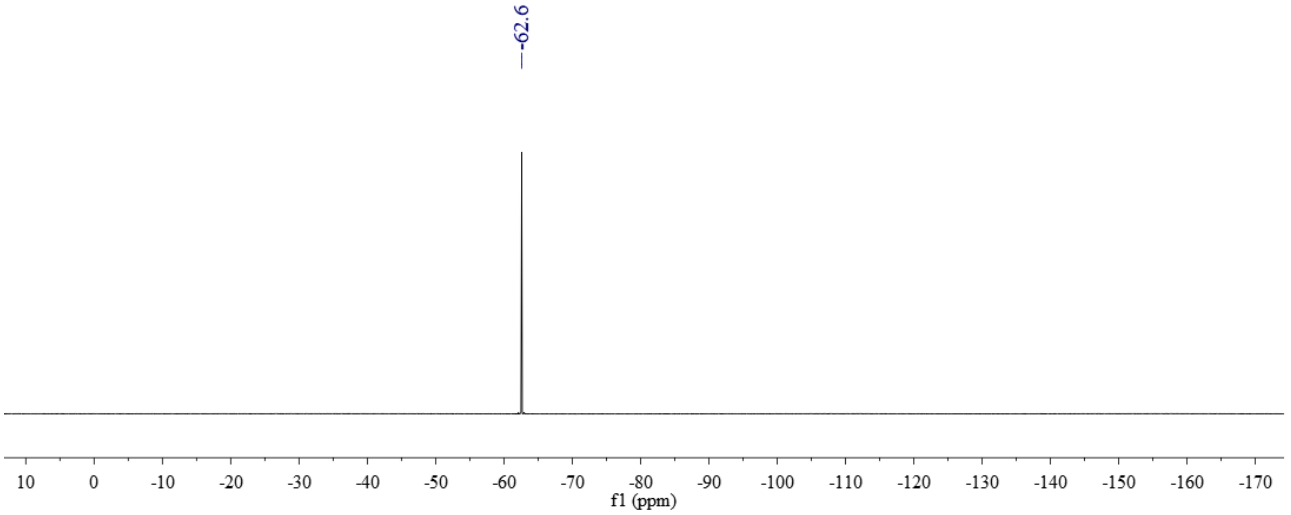

Supplementary Fig. 173. ^{31}F NMR spectrum of **46**. The sample has been recorded in 564 MHz, $CDCl_3$ at 25 °C.

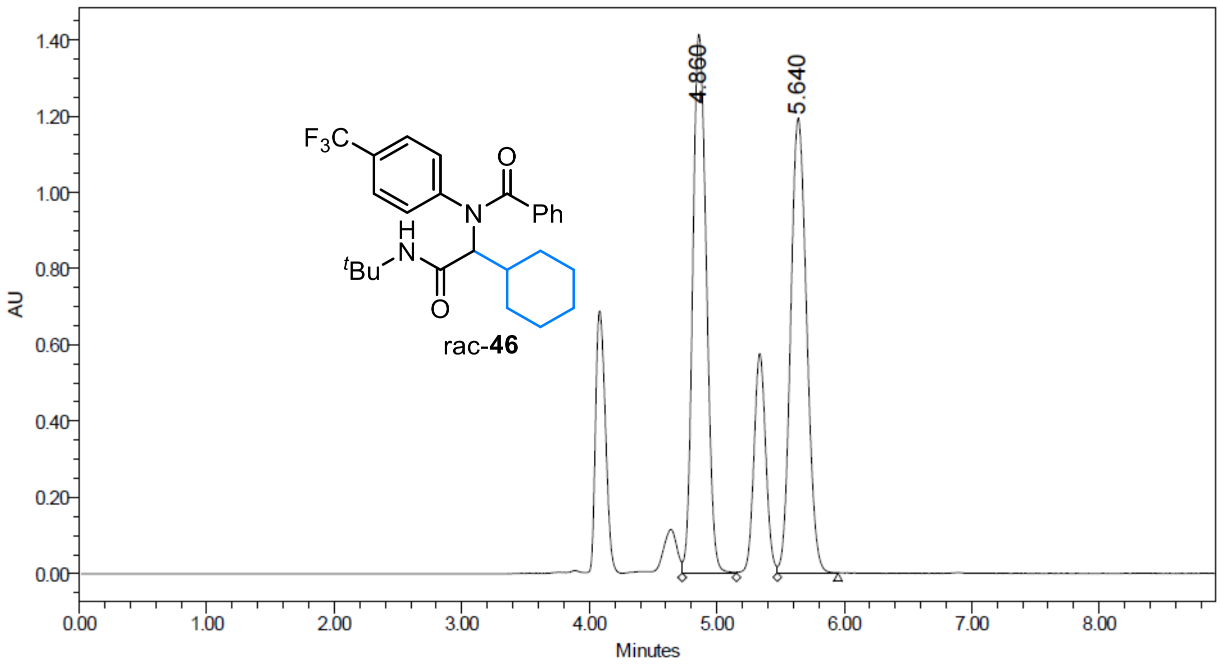

	RT (min)	Peak Type	Area (µV*sec)	% Area	Height (µV)	% Height	Integration Type	Points Across Peak	Start Time (min)	End Time (min)
1	4.860	Unknown	10479441	50.24	1412747	54.24	VV	256	4.728	5.155
2	5.640	Unknown	10380809	49.76	1191790	45.76	VB	286	5.475	5.952

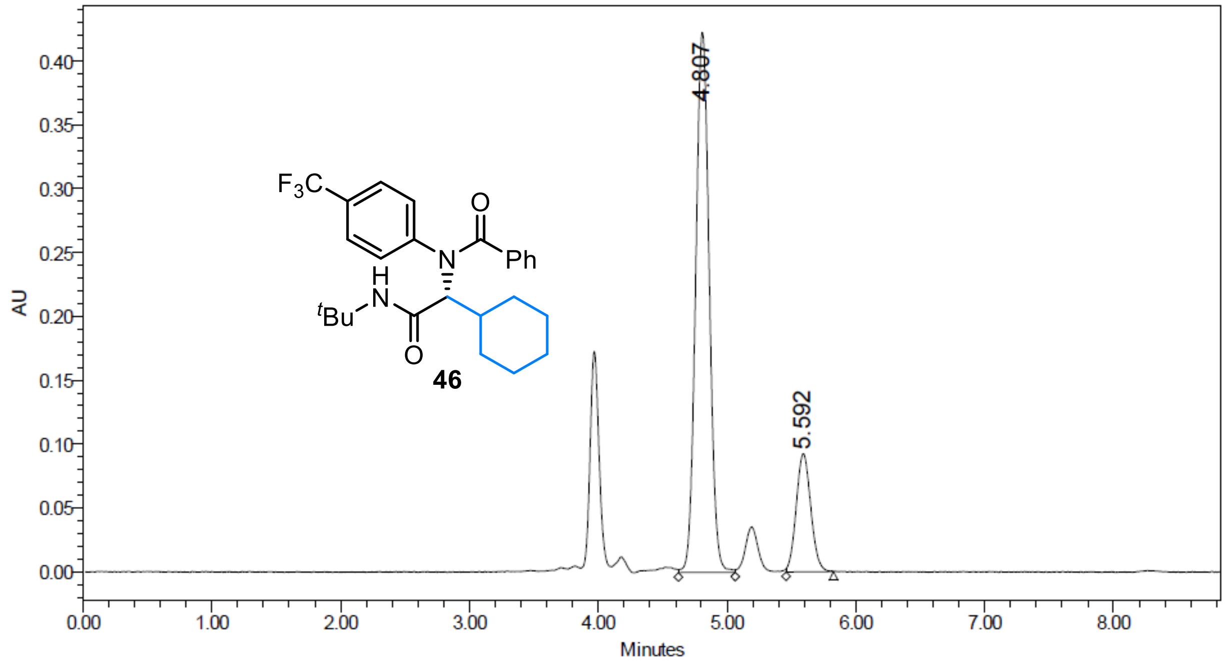

	RT (min)	Peak Type	Area (µV*sec)	% Area	Height (µV)	% Height	Integration Type	Points Across Peak	Start Time (min)	End Time (min)
1	4.807	Unknown	3097067	81.00	423123	82.05	VV	265	4.622	5.063
2	5.592	Unknown	726573	19.00	92570	17.95	vB	221	5.458	5.827

Supplementary Fig. 174. HPLC of product **46**.

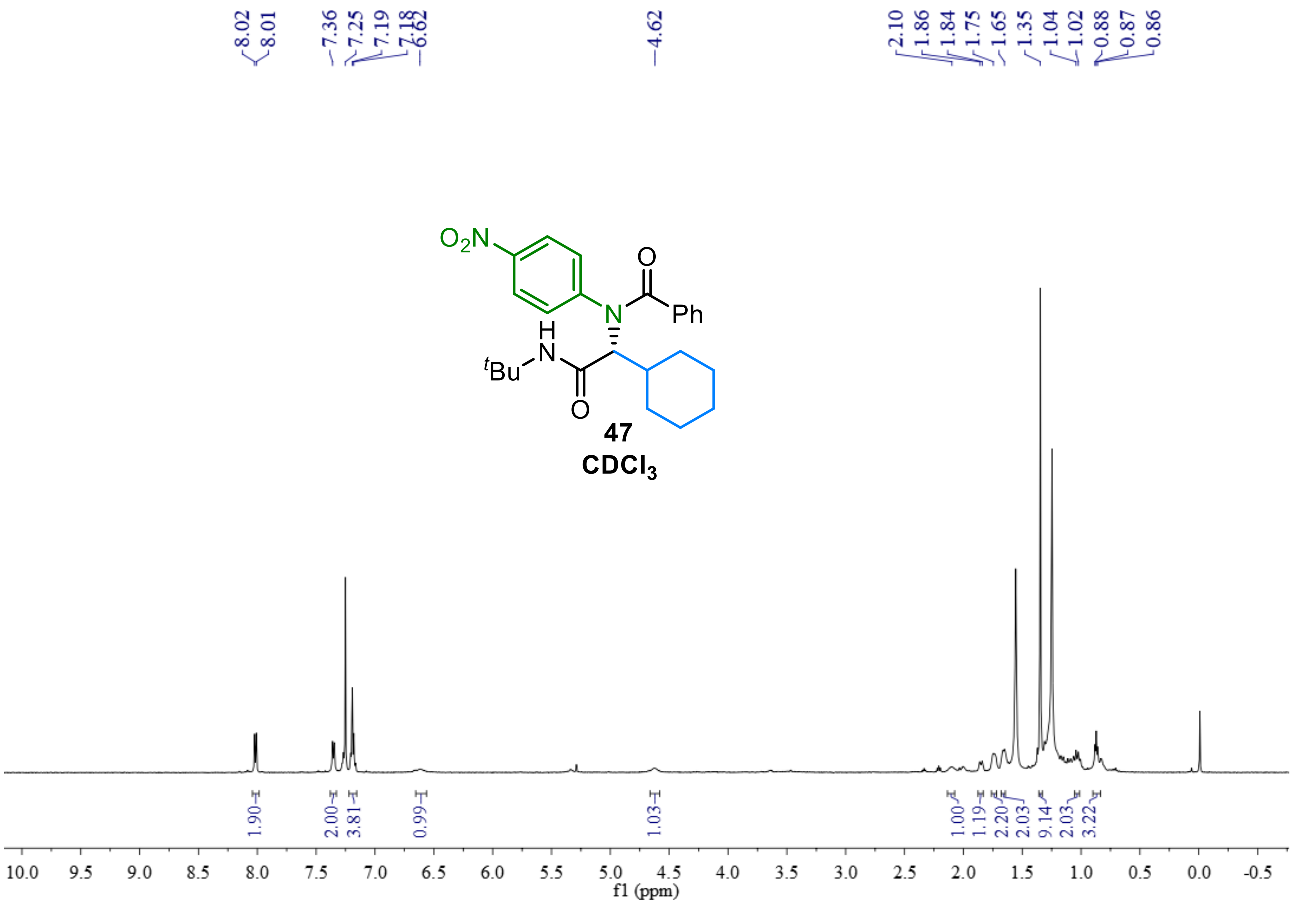

Supplementary Fig. 175. ^{1}H NMR spectrum of **47**. The sample has been recorded in 600 MHz, $CDCl_3$ at 25 °C.

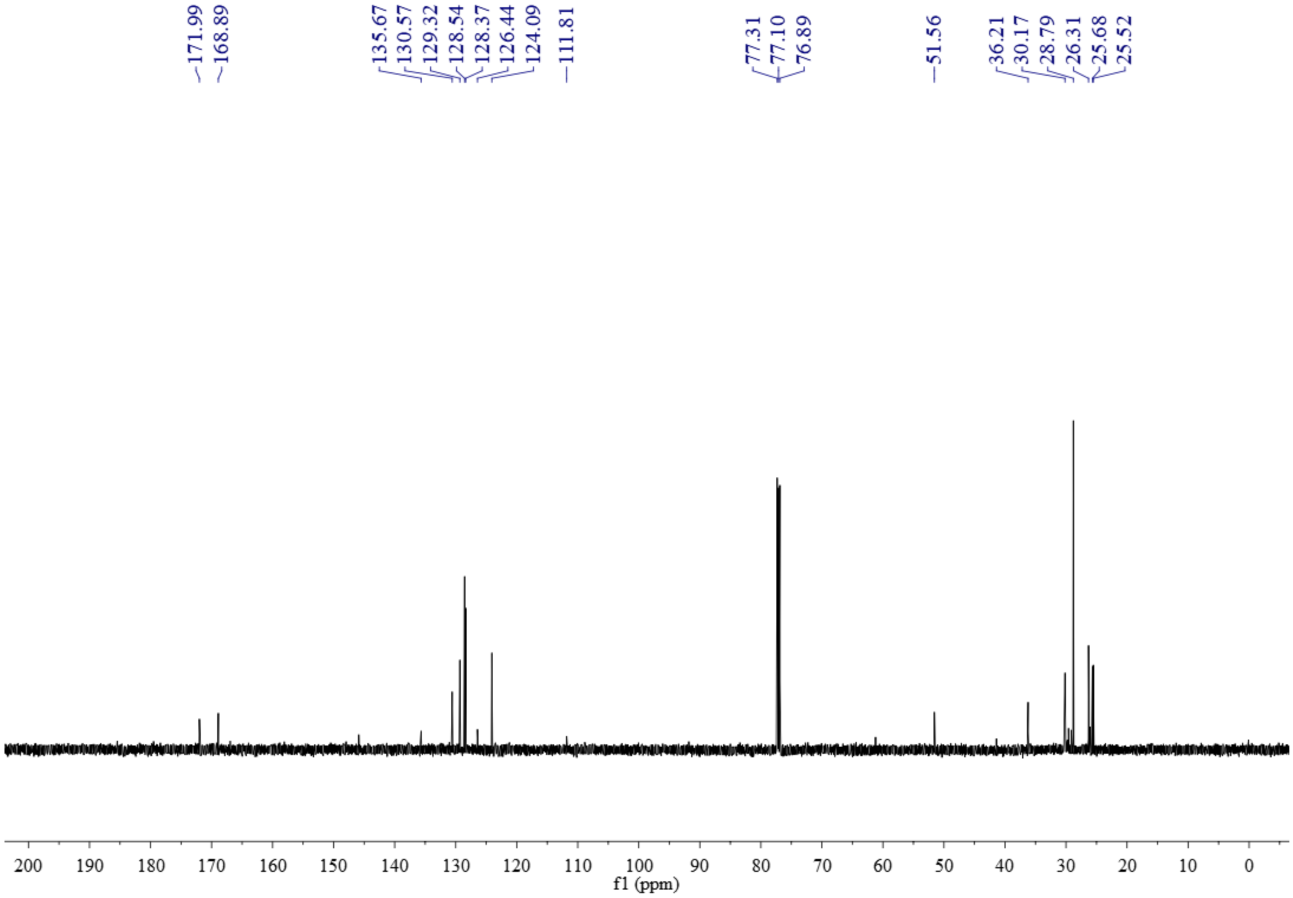

Supplementary Fig. 176. ^{13}C NMR spectrum of **47**. The sample has been recorded in 151 MHz, $CDCl_3$ at 25 °C.

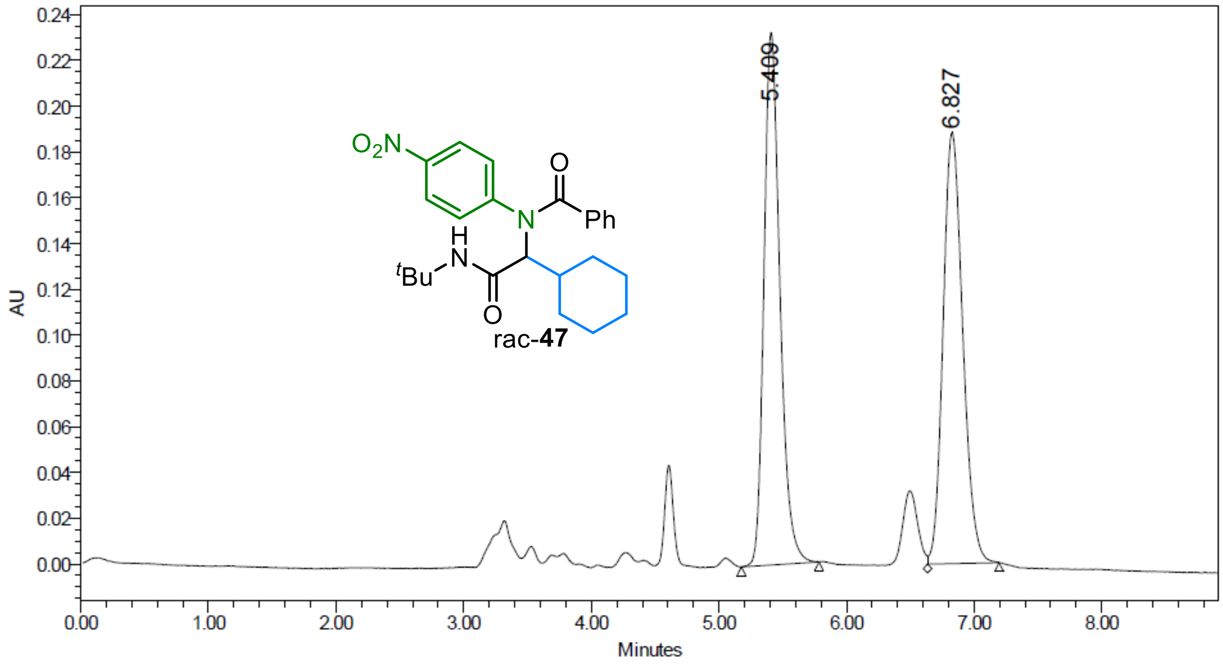

	RT (min)	Peak Type	Area (µV*sec)	% Area	Height (µV)	% Height	Integration Type	Points Across Peak	Start Time (min)	End Time (min)
1	5.409	Unknown	2024425	50.07	232718	55.25	BB	365	5.177	5.785
2	6.827	Unknown	2018795	49.93	188463	44.75	VB	336	6.635	7.195

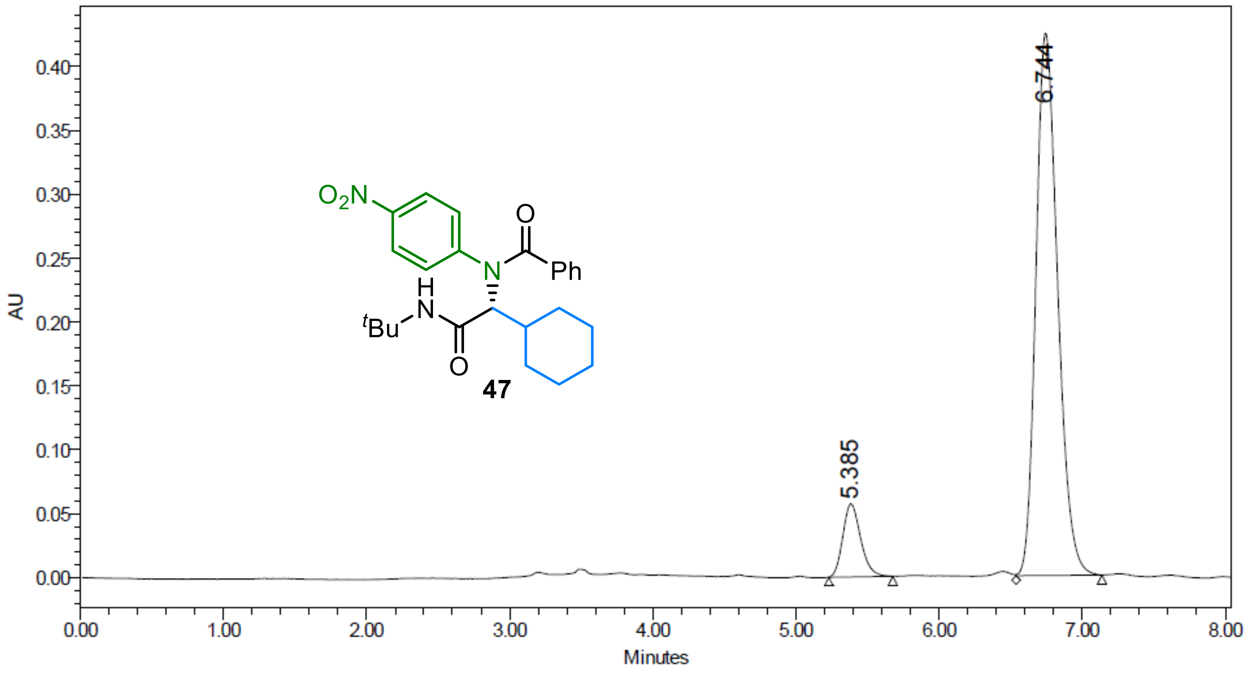

	RT (min)	Peak Type	Area (µV*sec)	% Area	Height (µV)	% Height	Integration Type	Points Across Peak	Start Time (min)	End Time (min)
1	5.385	Unknown	495198	9.94	57015	11.84	bb	268	5.230	5.677
2	6.744	Unknown	4486940	90.06	424576	88.16	VB	360	6.538	7.138

Supplementary Fig. 177. HPLC of product **47**.

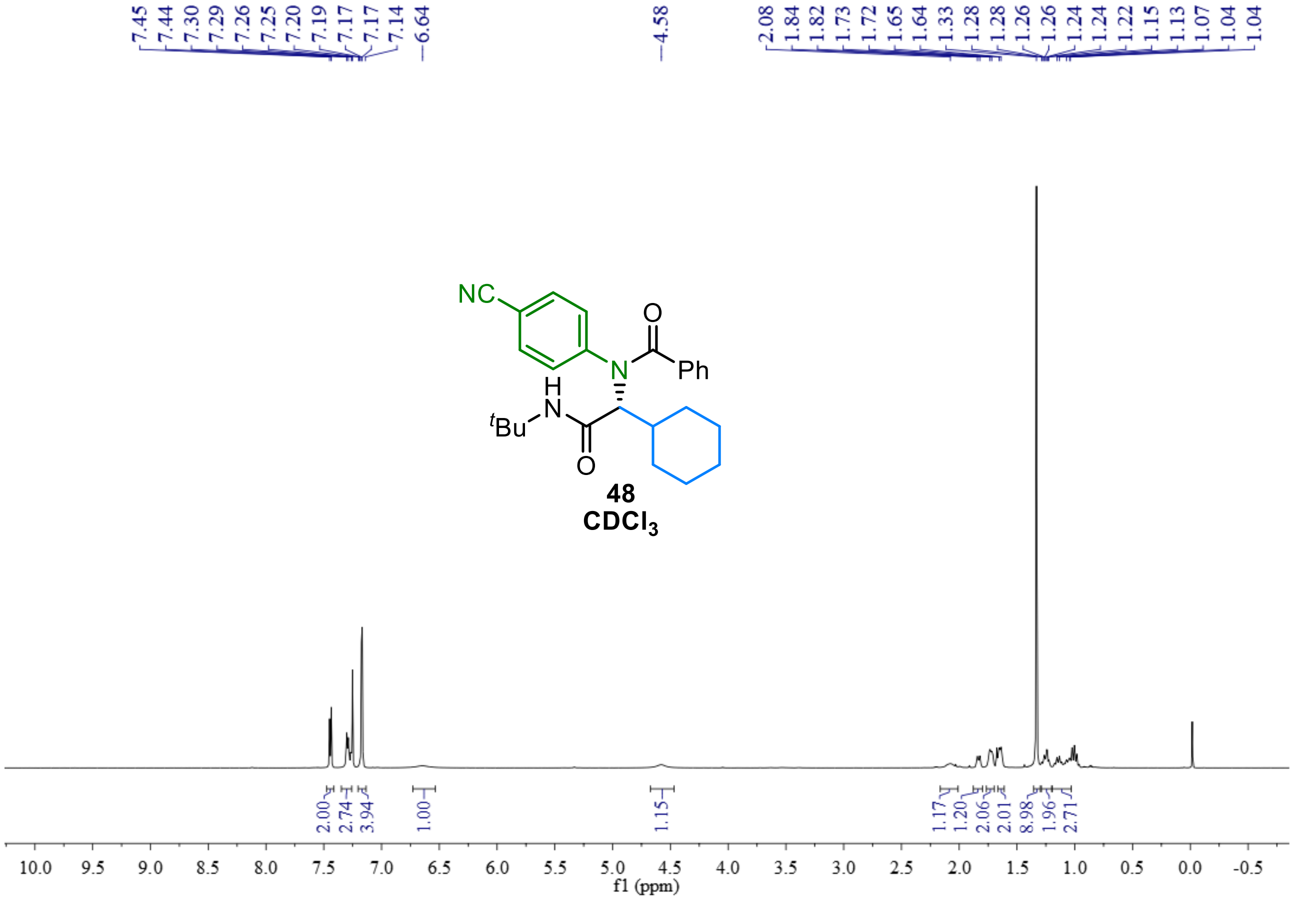

Supplementary Fig. 178. ^{1}H NMR spectrum of **48**. The sample has been recorded in 600 MHz, $CDCl_3$ at 25 °C.

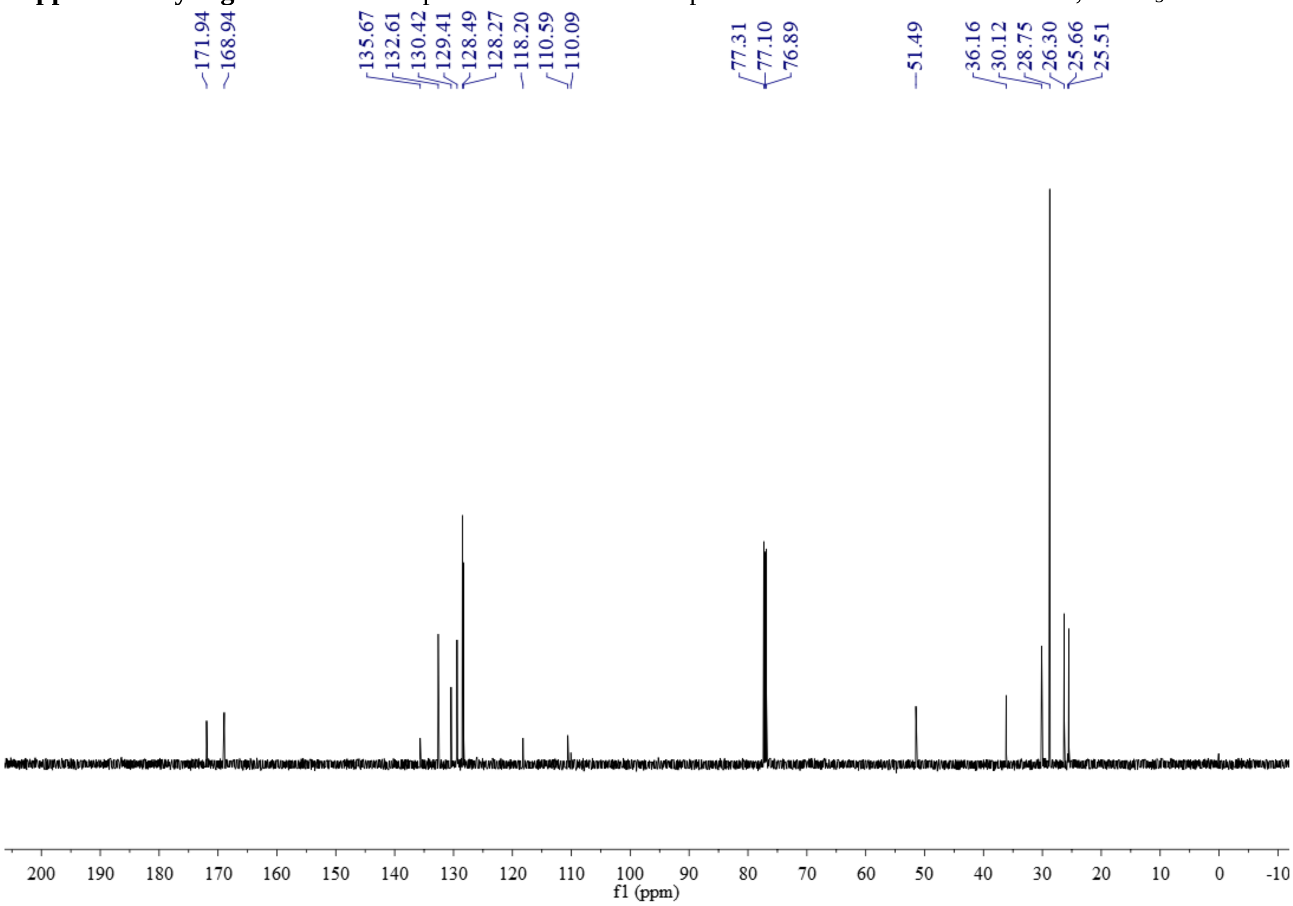

Supplementary Fig. 179. ^{13}C NMR spectrum of **48**. The sample has been recorded in 151 MHz, $CDCl_3$ at 25 °C.

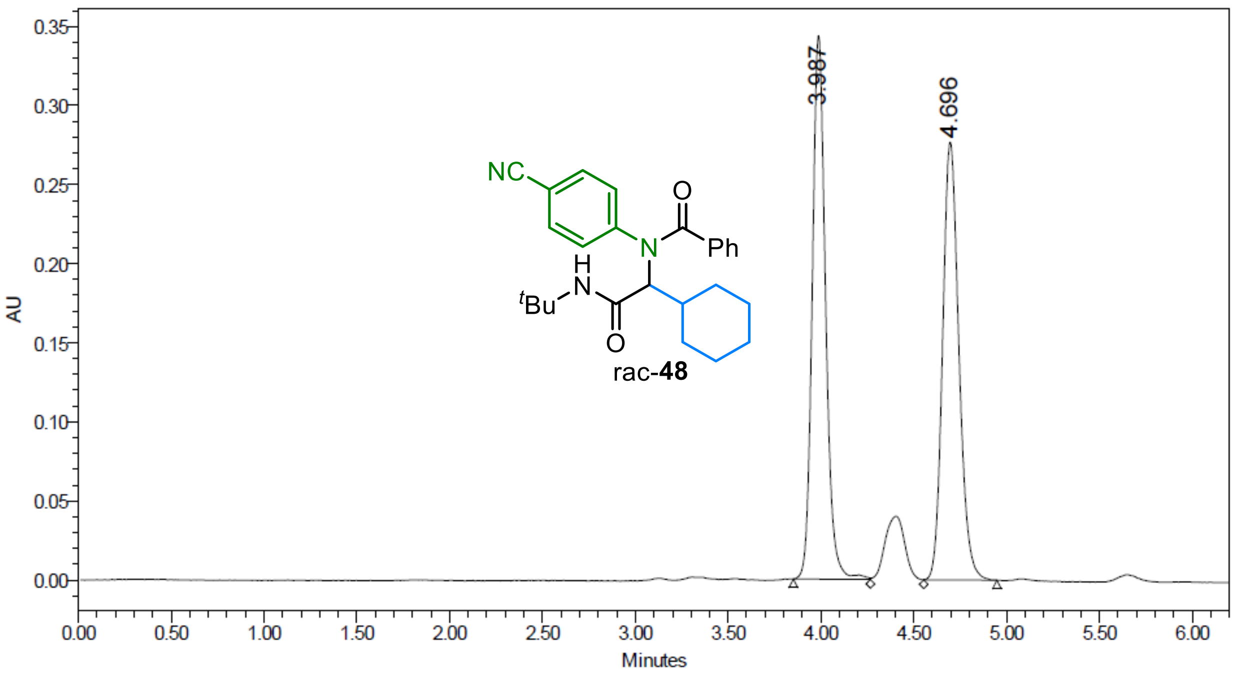

	RT (min)	Peak Type	Area (µV*sec)	% Area	Height (µV)	% Height	Integration Type	Points Across Peak	Start Time (min)	End Time (min)
1	3.987	Unknown	1715347	50.44	343630	55.38	BV	249	3.852	4.267
2	4.696	Unknown	1685221	49.56	276809	44.62	VB	237	4.553	4.948

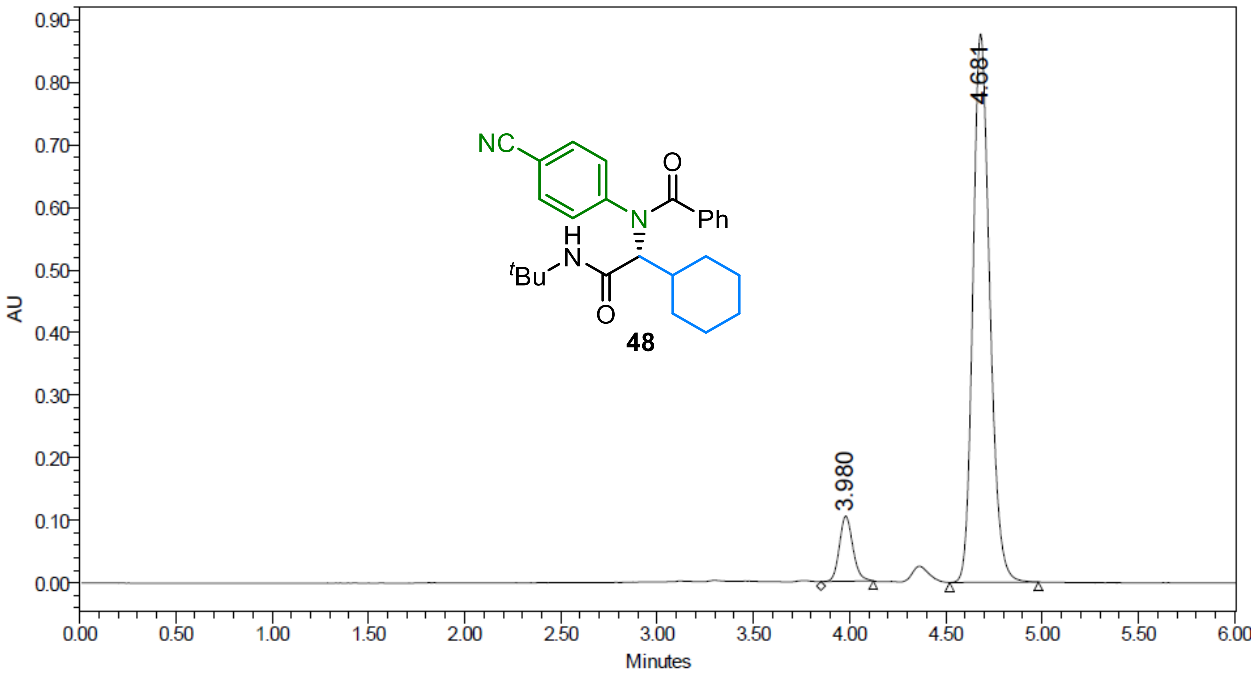

	RT (min)	Peak Type	Area (µV*sec)	% Area	Height (µV)	% Height	Integration Type	Points Across Peak	Start Time (min)	End Time (min)
1	3.980	Unknown	506299	8.67	104052	10.61	Vb	163	3.852	4.123
2	4.681	Unknown	5330811	91.33	876745	89.39	BB	277	4.520	4.982

Supplementary Fig. 180. HPLC of product **49**.

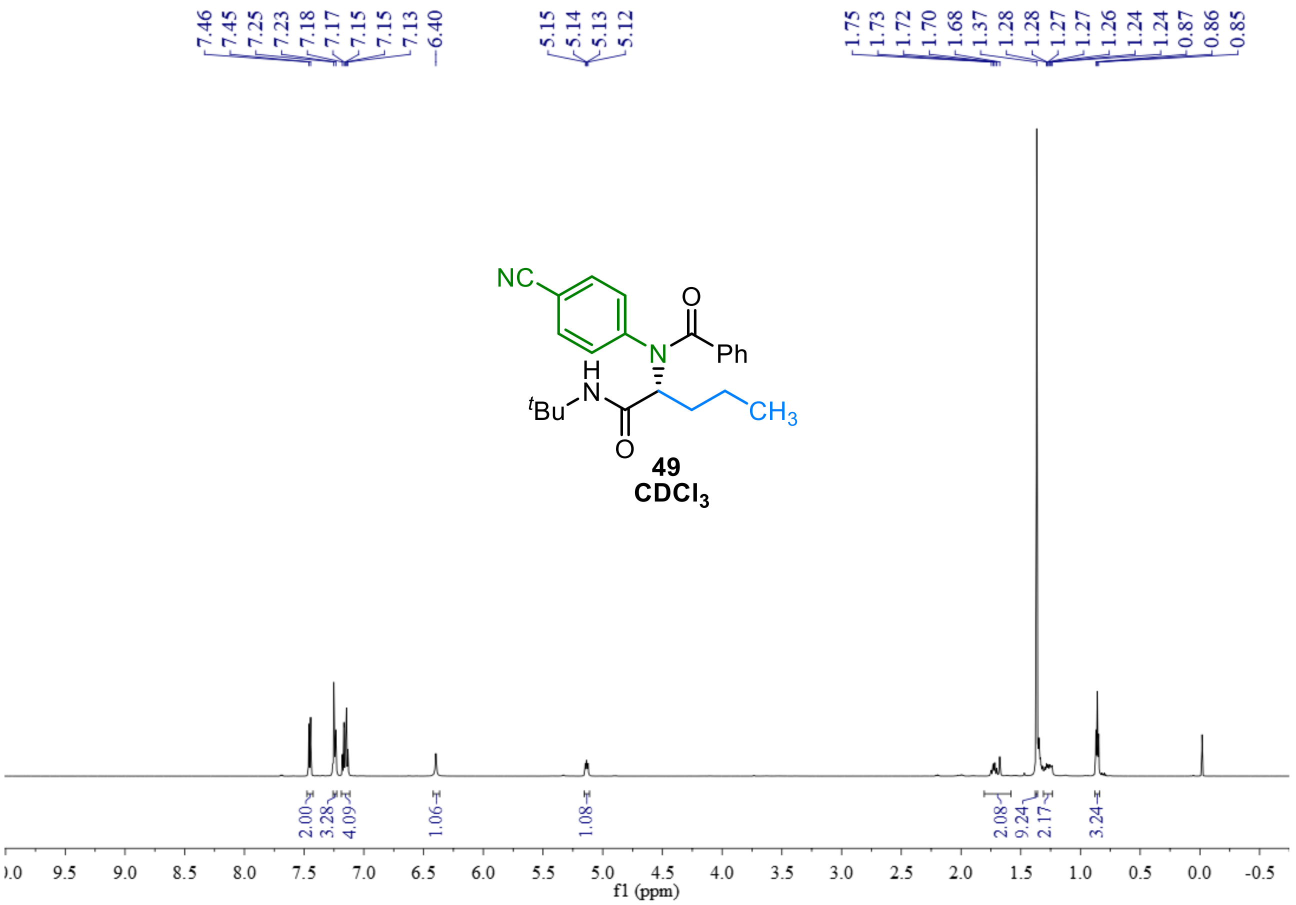

Supplementary Fig. 181. ^{1}H NMR spectrum of **49**. The sample has been recorded in 600 MHz, $CDCl_3$ at 25 °C.

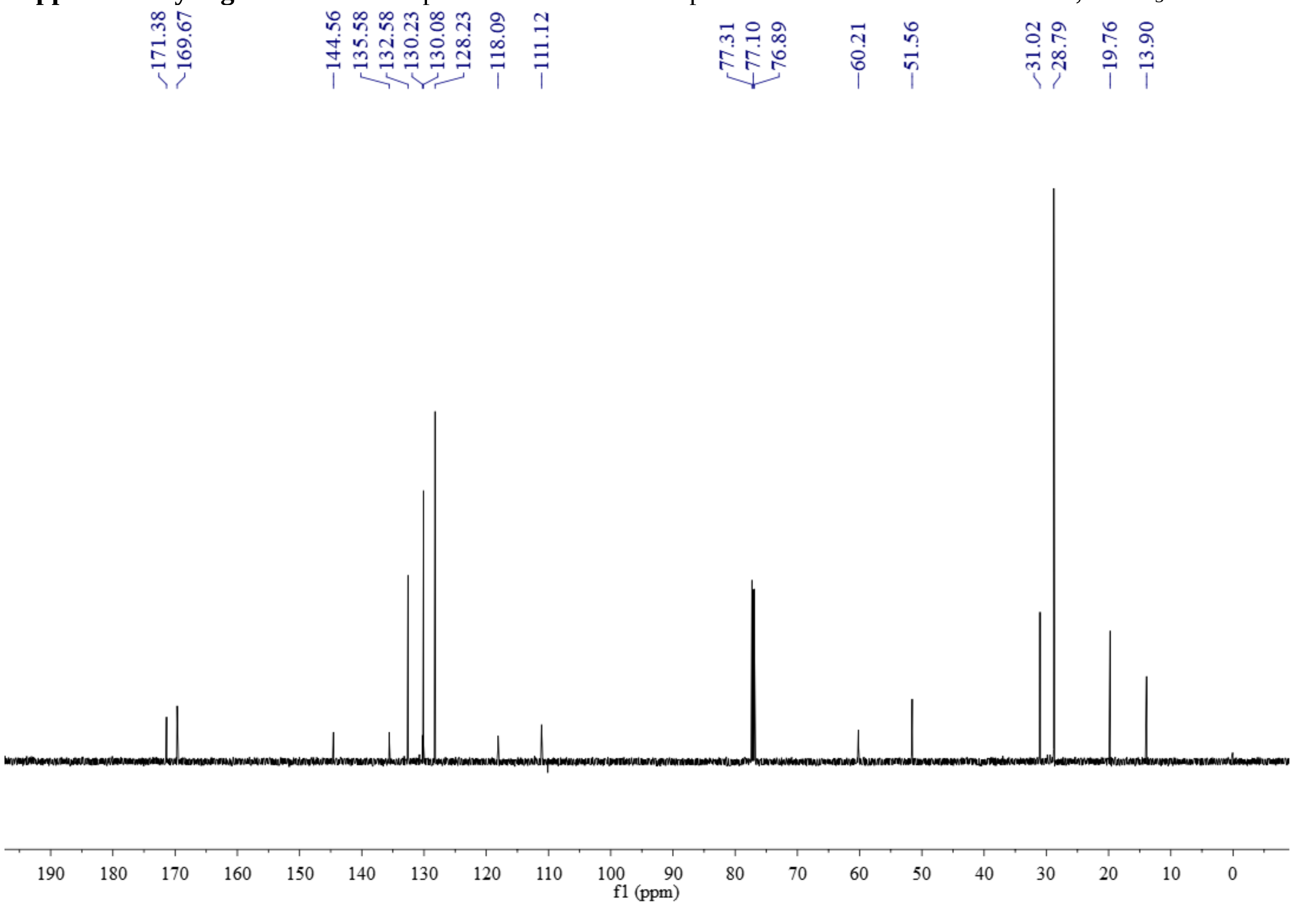

Supplementary Fig. 182. ^{13}C NMR spectrum of **49**. The sample has been recorded in 151 MHz, $CDCl_3$ at 25 °C.

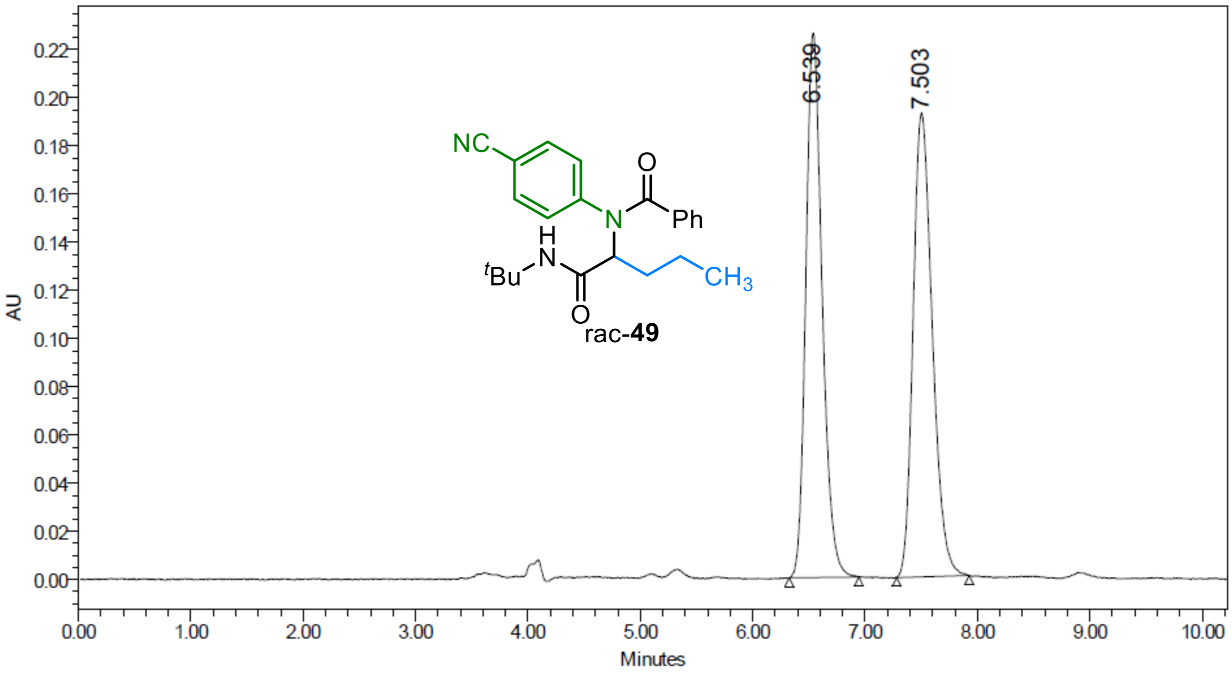

	RT (min)	Peak Type	Area (µV*sec)	% Area	Height (µV)	% Height	Integration Type	Points Across Peak	Start Time (min)	End Time (min)
1	6.539	Unknown	2282251	49.93	226102	54.02	BB	372	6.325	6.945
2	7.503	Unknown	2288247	50.07	192459	45.98	BB	387	7.282	7.927

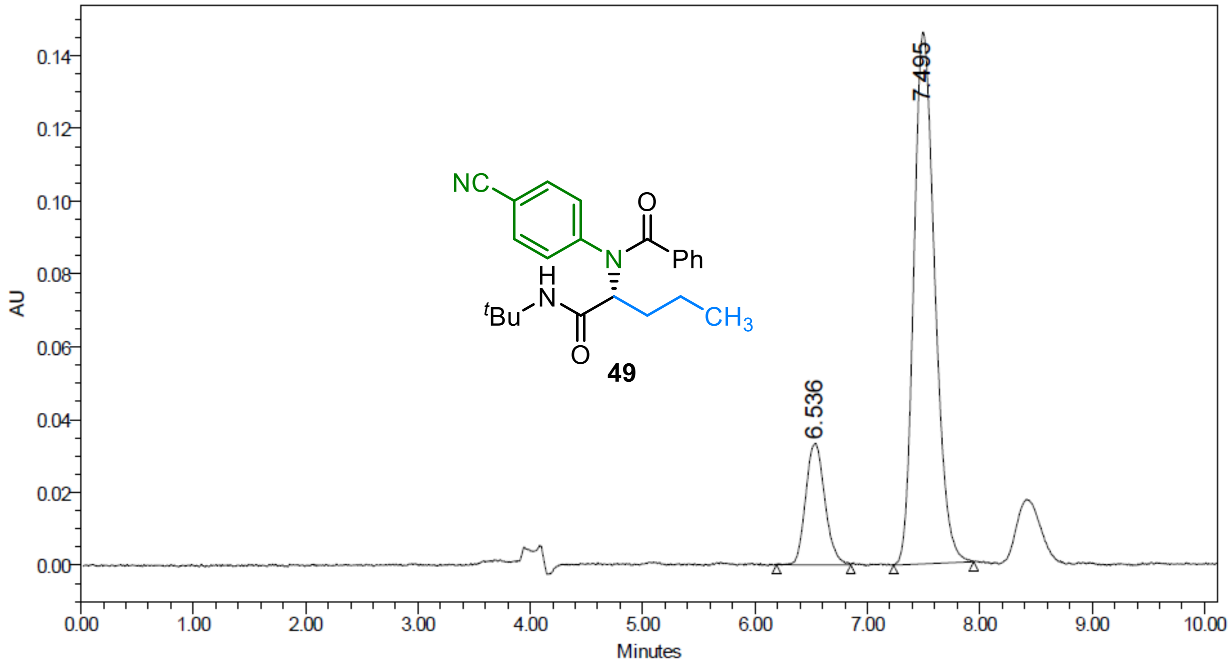

	RT (min)	Peak Type	Area (µV*sec)	% Area	Height (µV)	% Height	Integration Type	Points Across Peak	Start Time (min)	End Time (min)
1	6.536	Unknown	396929	17.31	33380	18.59	bB	397	6.190	6.852
2	7.495	Unknown	1896604	82.69	146170	81.41	BB	428	7.233	7.947

Supplementary Fig. 183. HPLC of product **49**.

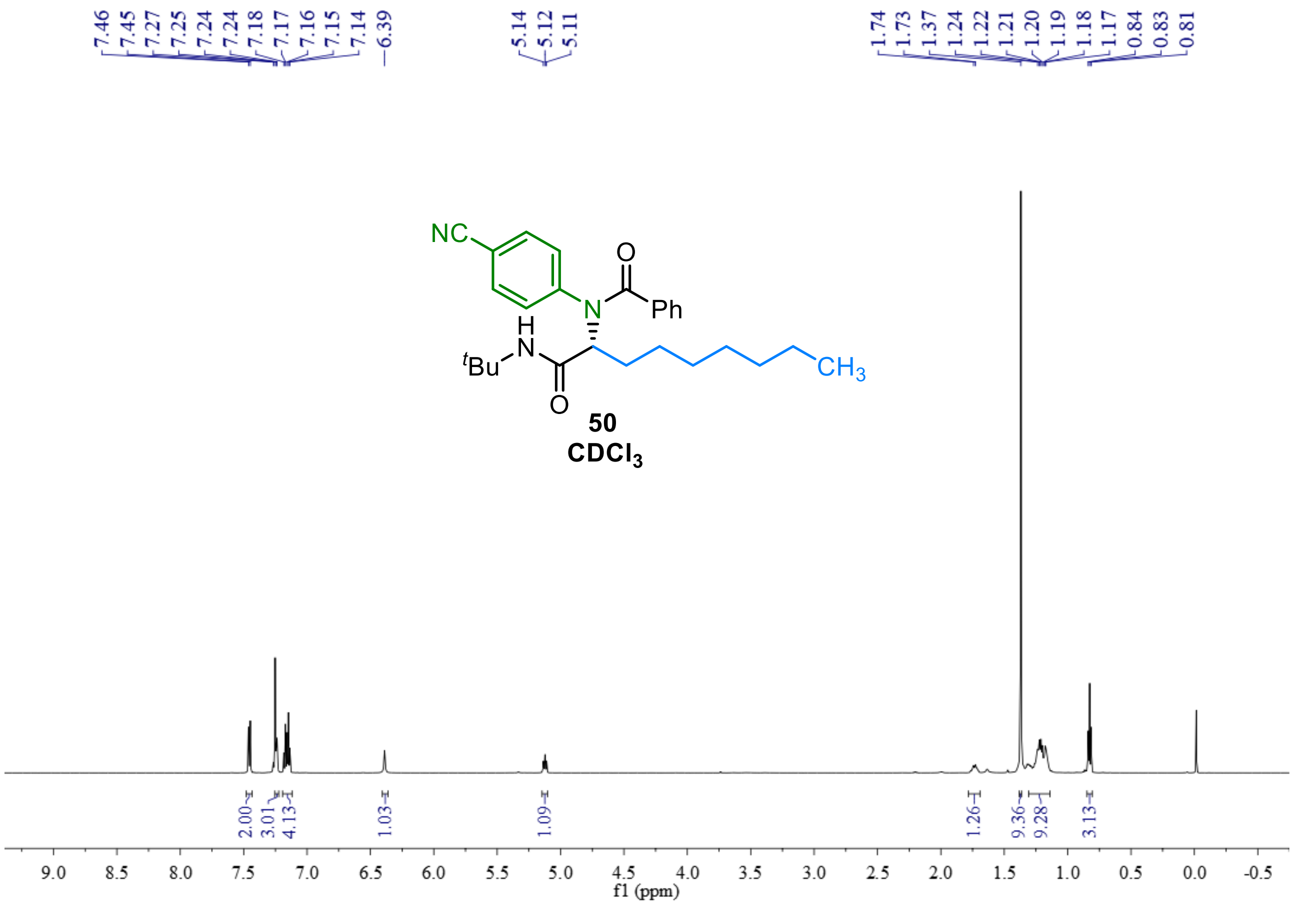

Supplementary Fig. 184. ^{1}H NMR spectrum of **50**. The sample has been recorded in 600 MHz, $CDCl_3$ at 25 °C.

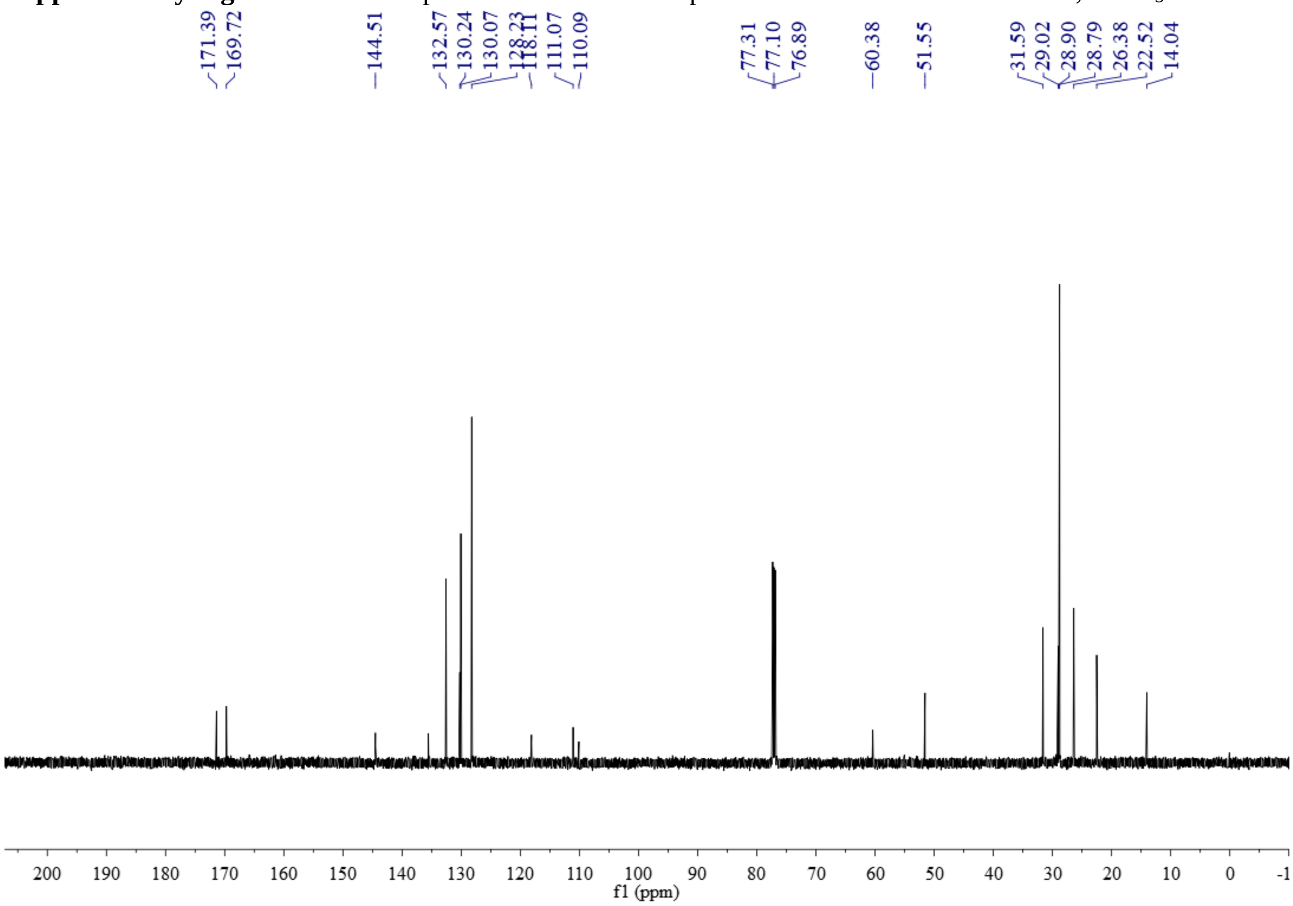

Supplementary Fig. 185. ^{13}C NMR spectrum of **50**. The sample has been recorded in 151 MHz, $CDCl_3$ at 25 °C.

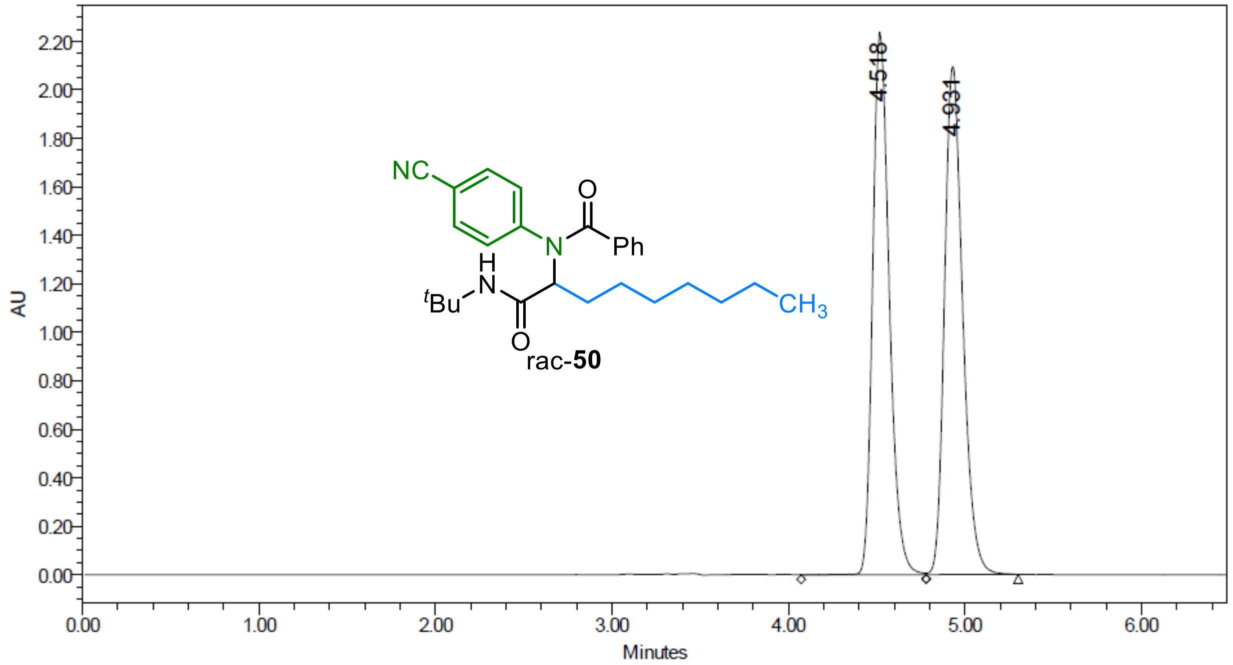

	RT (min)	Peak Type	Area (µV*sec)	% Area	Height (µV)	% Height	Integration Type	Points Across Peak	Start Time (min)	End Time (min)
1	4.518	Unknown	14626713	49.37	2237174	51.65	VV	425	4.072	4.780
2	4.931	Unknown	15001039	50.63	2093938	48.35	VB	313	4.780	5.302

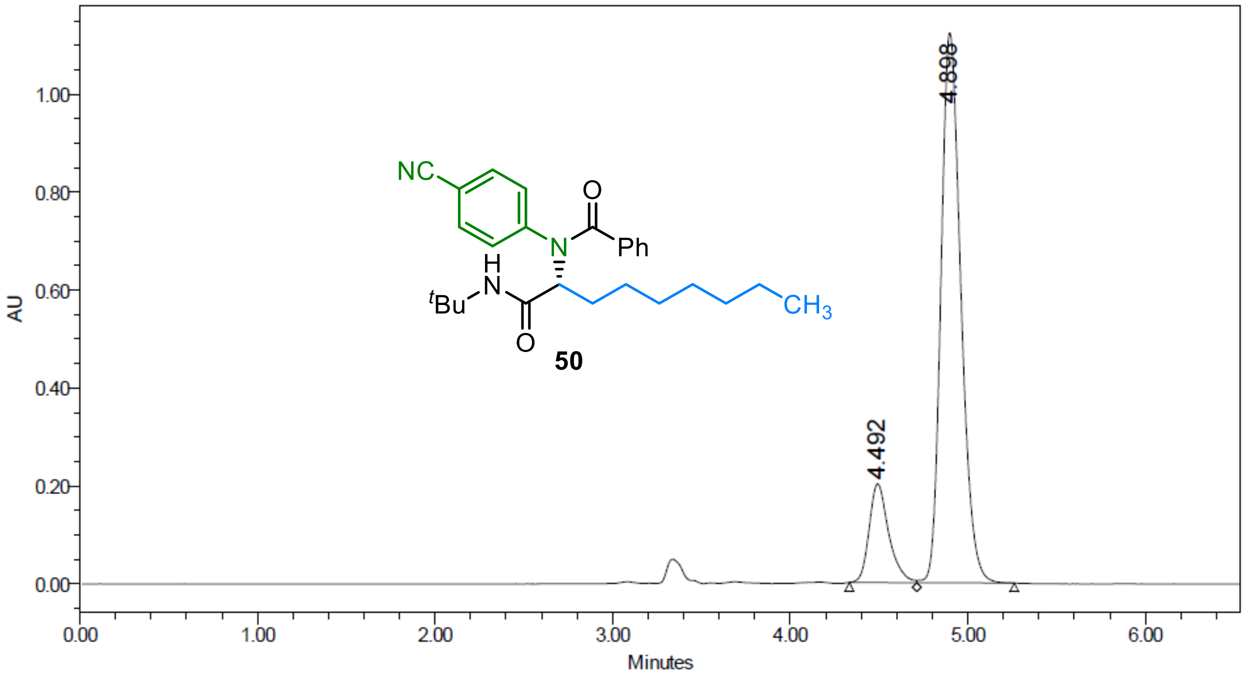

	RT (min)	Peak Type	Area (µV*sec)	% Area	Height (µV)	% Height	Integration Type	Points Across Peak	Start Time (min)	End Time (min)
1	4.492	Unknown	1550180	15.06	201541	15.21	bV	228	4.333	4.713
2	4.898	Unknown	8741743	84.94	1123345	84.79	VB	329	4.713	5.262

Supplementary Fig. 186. HPLC of product **50**.

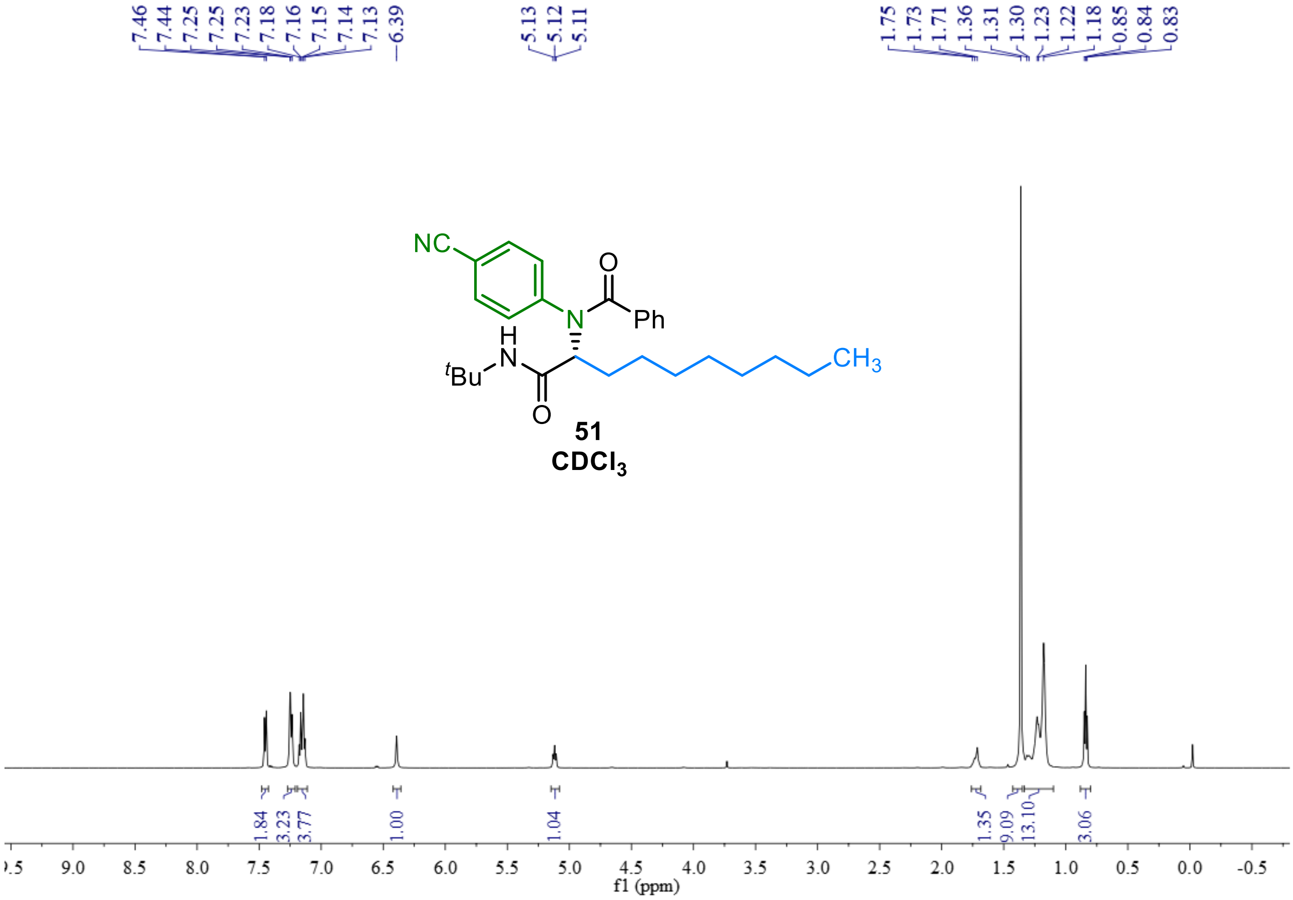

Supplementary Fig. 187. ^{1}H NMR spectrum of **51**. The sample has been recorded in 600 MHz, $CDCl_3$ at 25 °C.

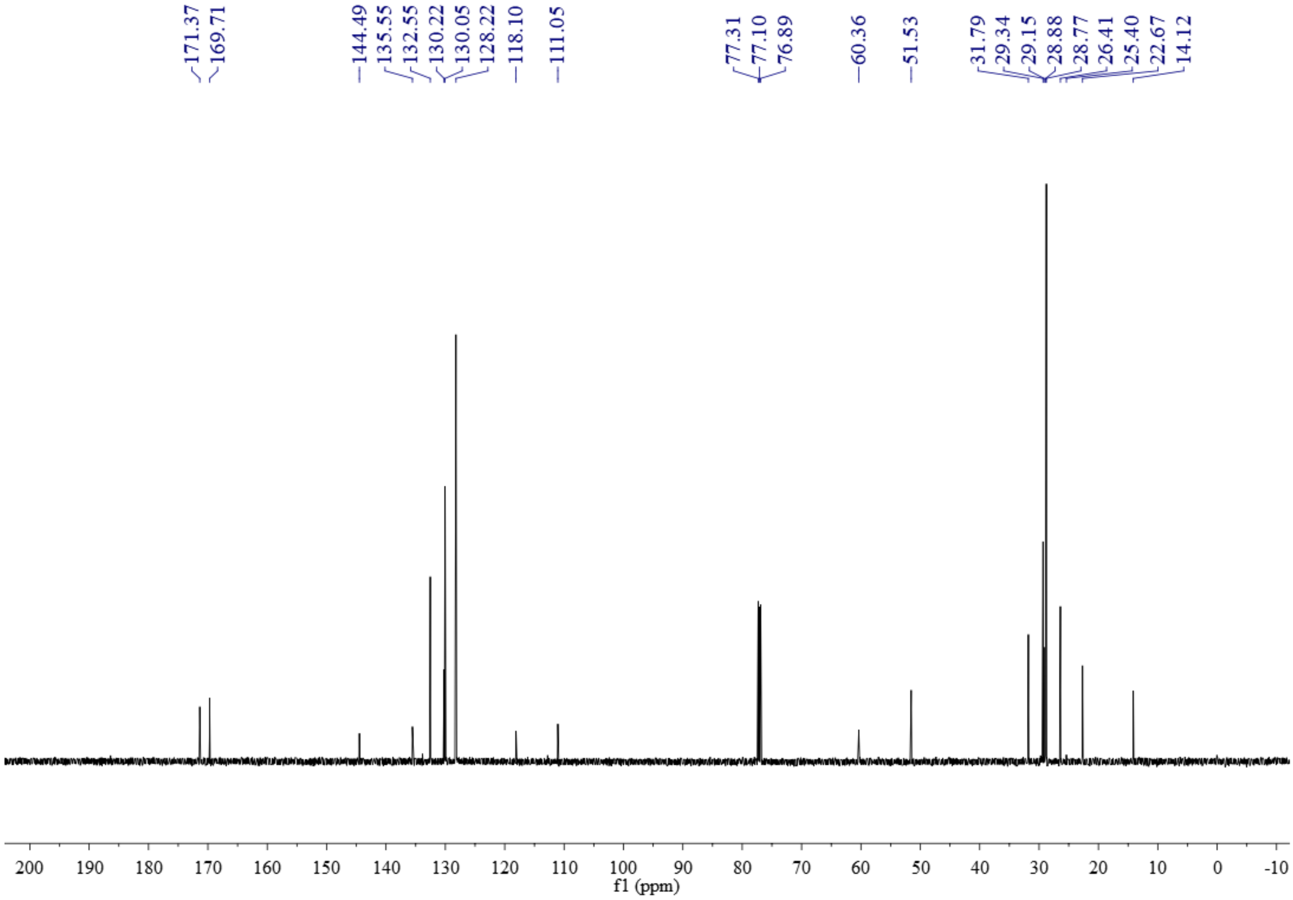

Supplementary Fig. 188. ^{13}C NMR spectrum of **51**. The sample has been recorded in 151 MHz, $CDCl_3$ at 25 °C.

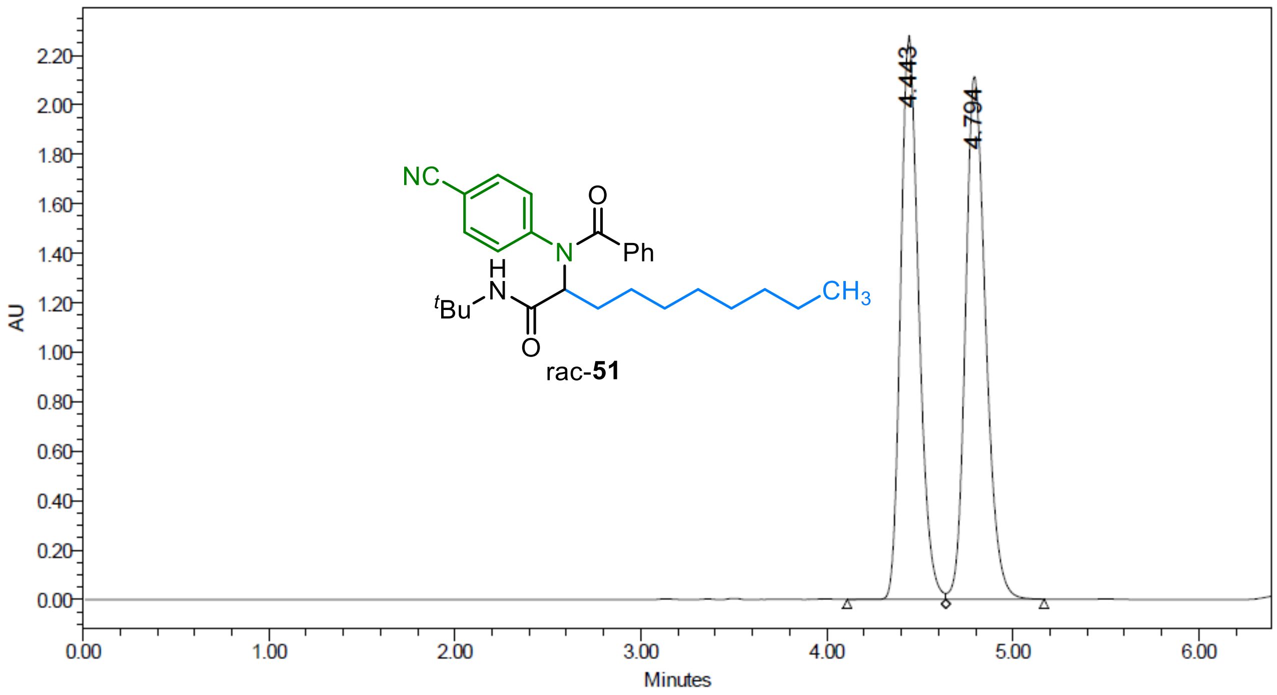

	RT (min)	Peak Type	Area (µV*sec)	% Area	Height (µV)	% Height	Integration Type	Points Across Peak	Start Time (min)	End Time (min)
1	4.443	Unknown	15387238	49.07	2278481	51.90	BV	318	4.110	4.640
2	4.794	Unknown	15971224	50.93	211148[illegible]	48.10	VB	317	4.640	5.168

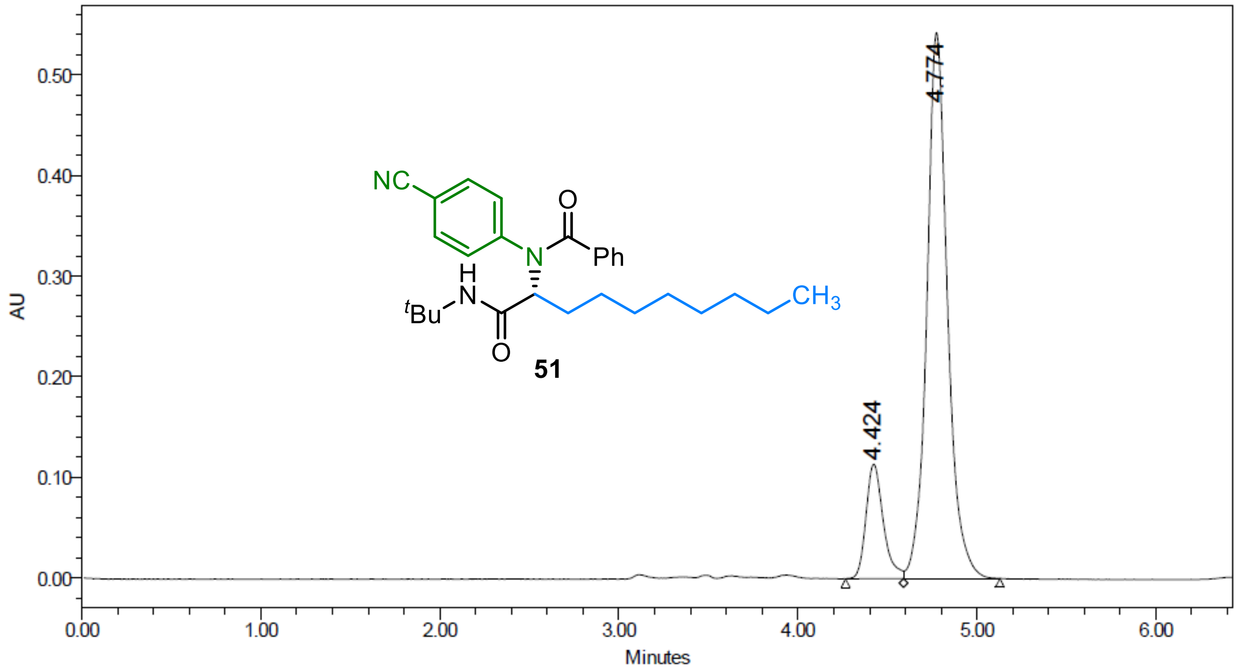

	RT (min)	Peak Type	Area (µV*sec)	% Area	Height (µV)	% Height	Integration Type	Points Across Peak	Start Time (min)	End Time (min)
1	4.424	Unknown	767878	14.98	113791	17.33	BV	194	4.267	4.590
2	4.774	Unknown	4357703	85.02	543007	82.67	VB	323	4.590	5.128

Supplementary Fig. 189. HPLC of product **51**.

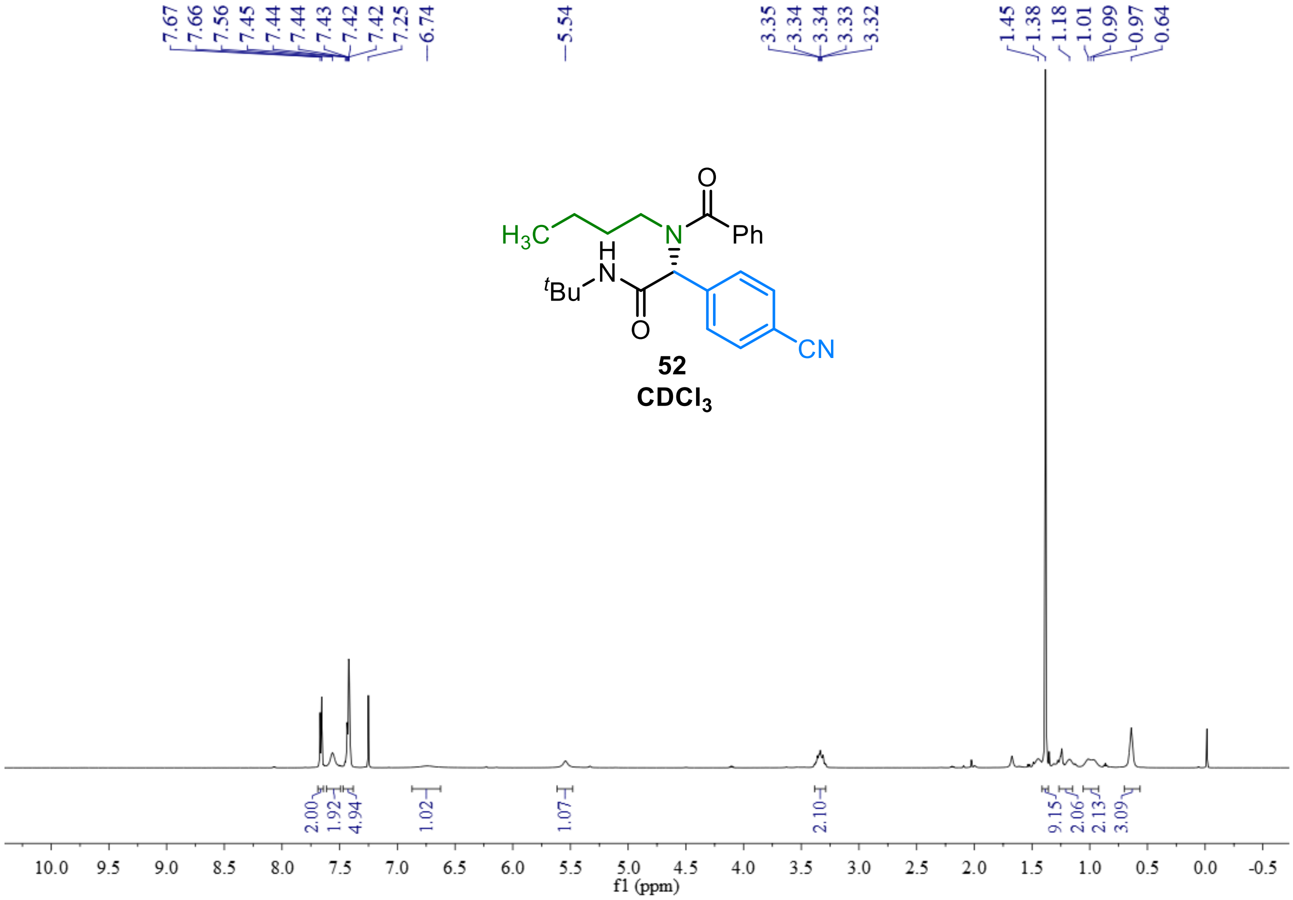

Supplementary Fig. 190. ^{1}H NMR spectrum of **52**. The sample has been recorded in 600 MHz, $CDCl_3$ at 25 °C.

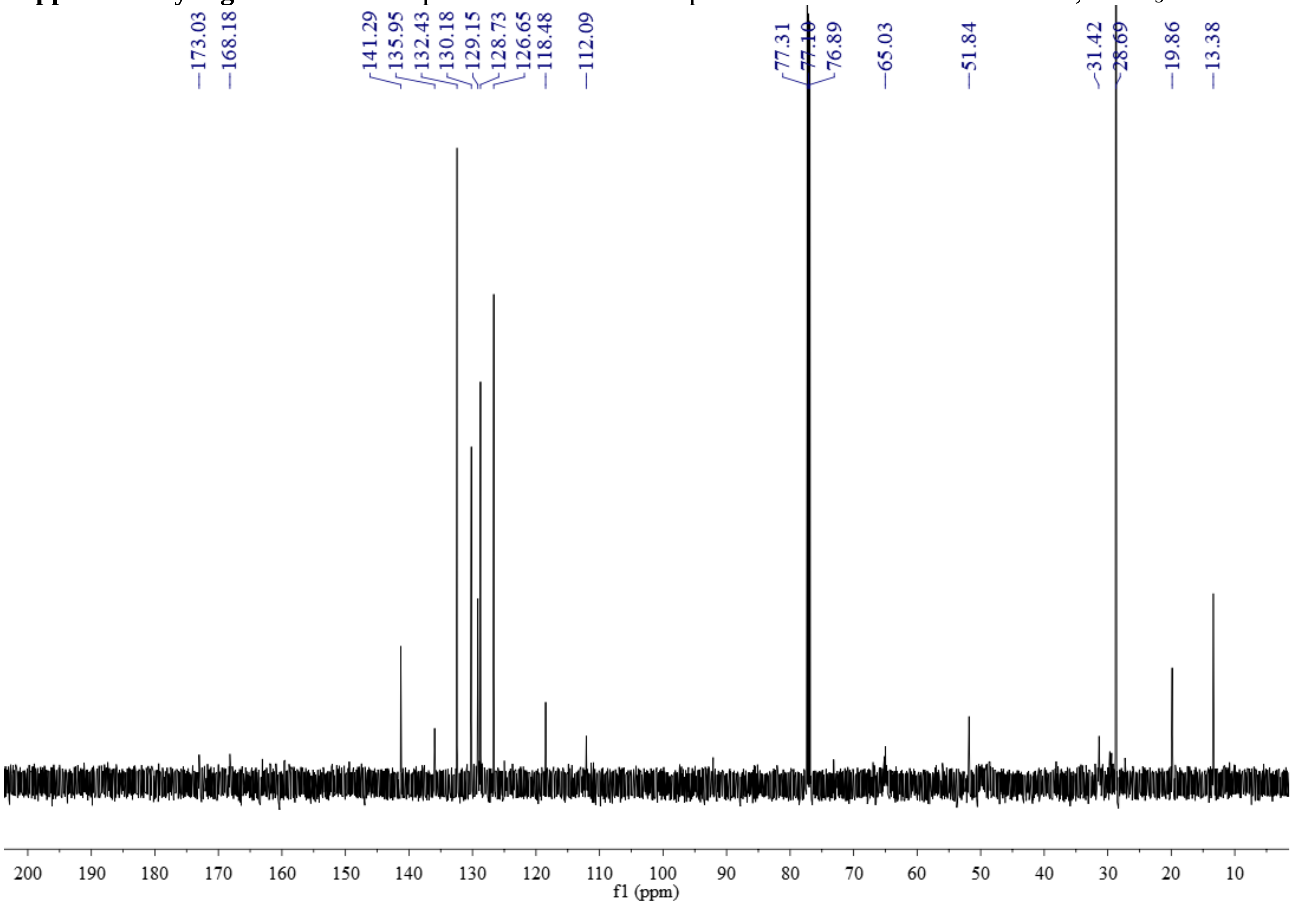

Supplementary Fig. 191. ^{13}C NMR spectrum of **52**. The sample has been recorded in 151 MHz, $CDCl_3$ at 25 °C.

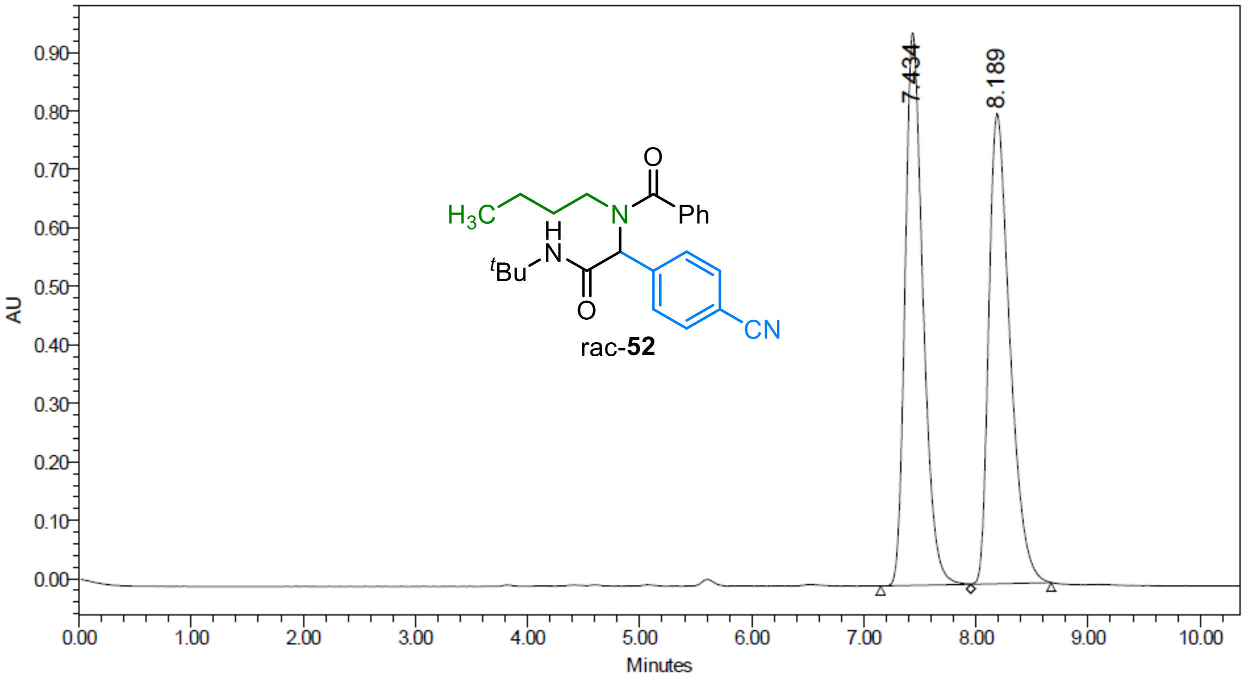

	RT (min)	Peak Type	Area (μV*sec)	% Area	Height (μV)	% Height	Integration Type	Points Across Peak	Start Time (min)	End Time (min)
1	7.434	Unknown	10492571	49.97	944476	54.03	BV	482	7.150	7.953
2	8.189	Unknown	10505165	50.03	803517	45.97	Vb	430	7.953	8.670

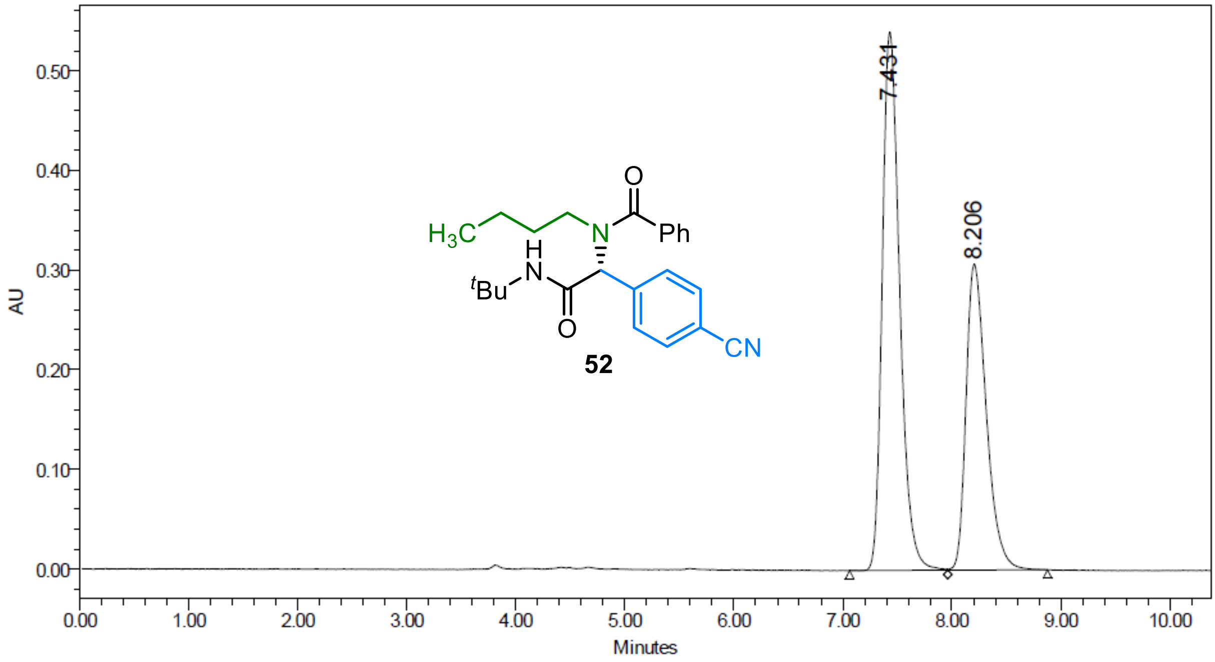

	RT (min)	Peak Type	Area (μV*sec)	% Area	Height (μV)	% Height	Integration Type	Points Across Peak	Start Time (min)	End Time (min)
1	7.431	Unknown	6012211	60.30	540394	63.78	bV	540	7.062	7.962
2	8.206	Unknown	3957674	39.70	306932	36.22	Vb	550	7.962	8.878

Supplementary Fig. 192. HPLC of product **52**.

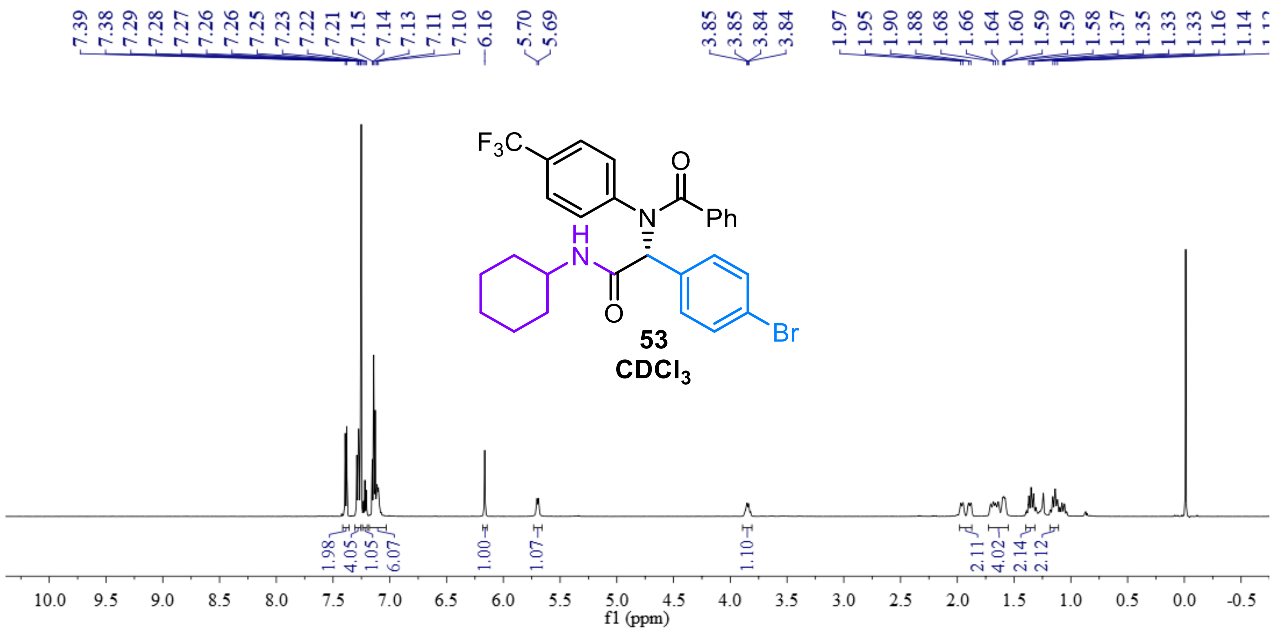

Supplementary Fig. 193. ^{1}H NMR spectrum of **53**. The sample has been recorded in 600 MHz, $CDCl_3$ at 25 °C.

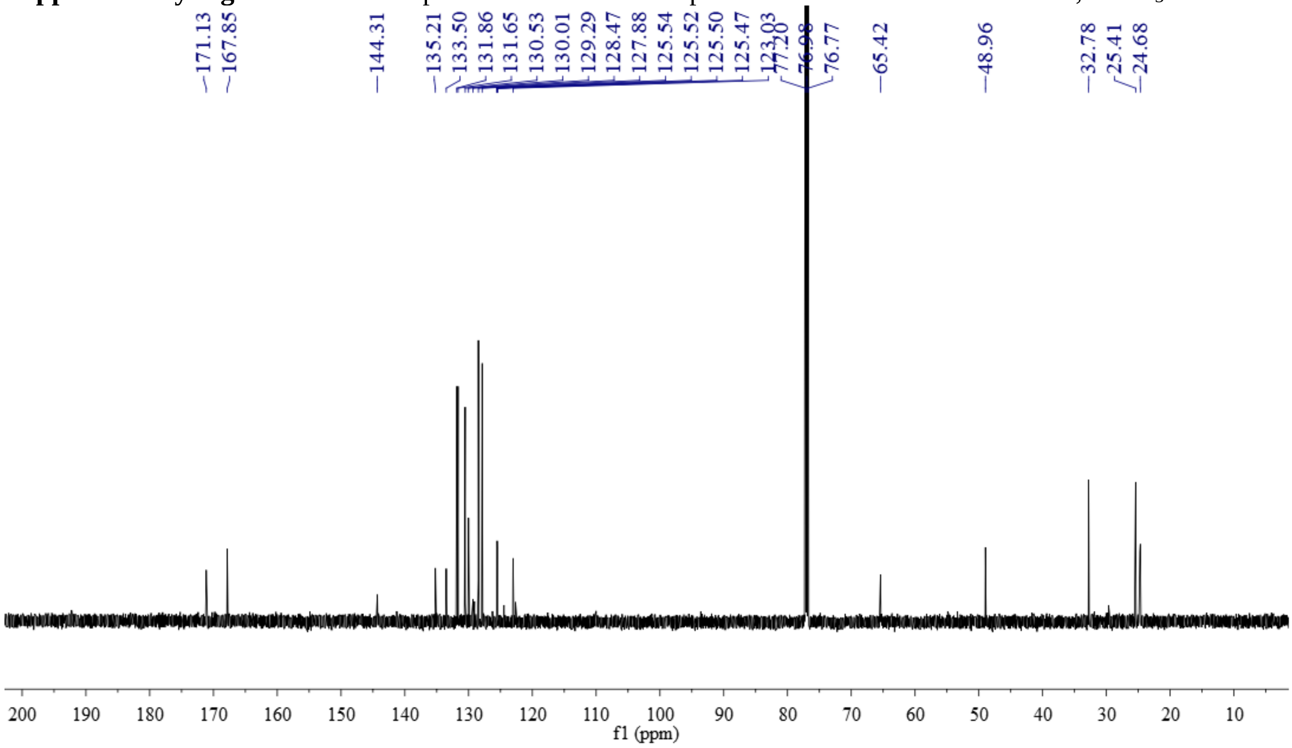

Supplementary Fig. 194. ^{13}C NMR spectrum of **53**. The sample has been recorded in 151 MHz, $CDCl_3$ at 25 °C.

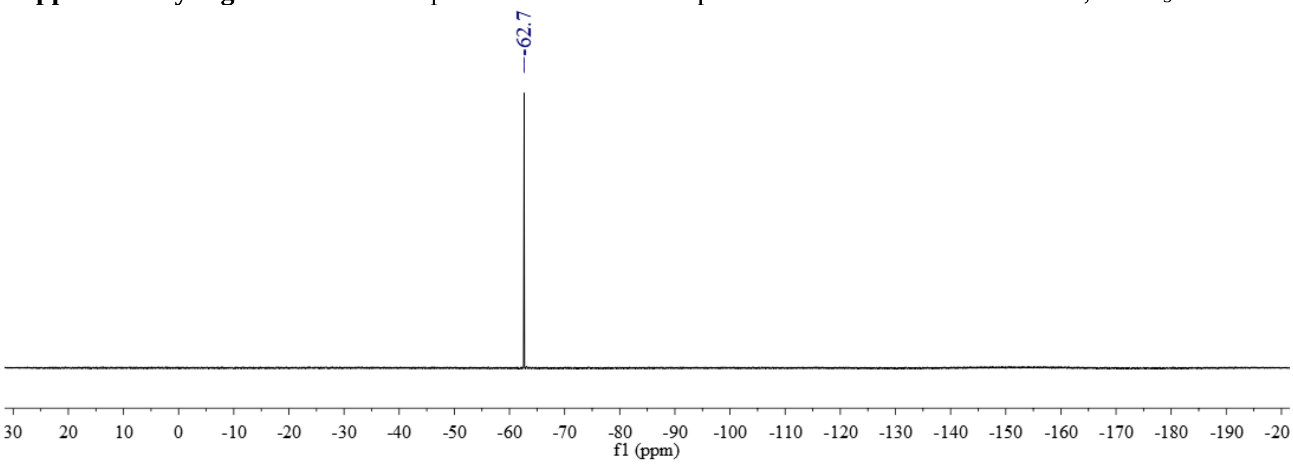

Supplementary Fig. 195. ^{31}F NMR spectrum of **53**. The sample has been recorded in 564 MHz, $CDCl_3$ at 25 °C.

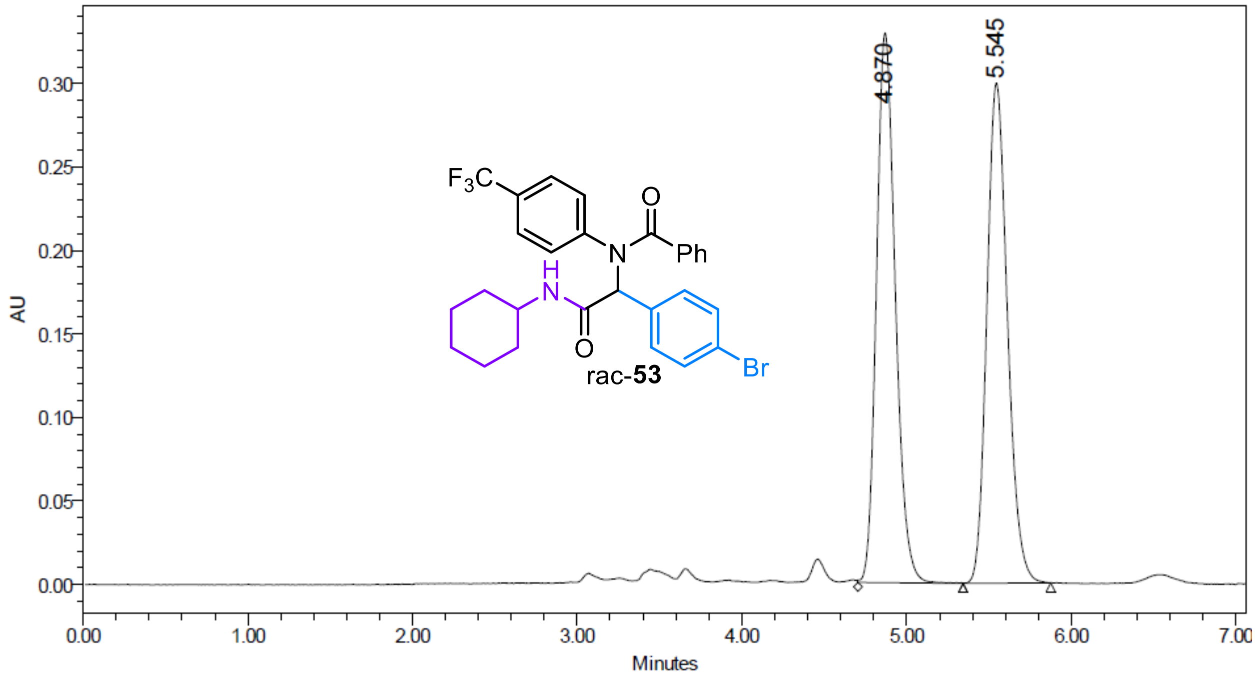

	RT (min)	Peak Type	Area (µV*sec)	% Area	Height (µV)	% Height	Integration Type	Points Across Peak	Start Time (min)	End Time (min)
1	4.870	Unknown	2620226	50.19	329201	52.36	VB	383	4.705	5.343
2	5.545	Unknown	2600034	49.81	299514	47.64	BB	319	5.343	5.875

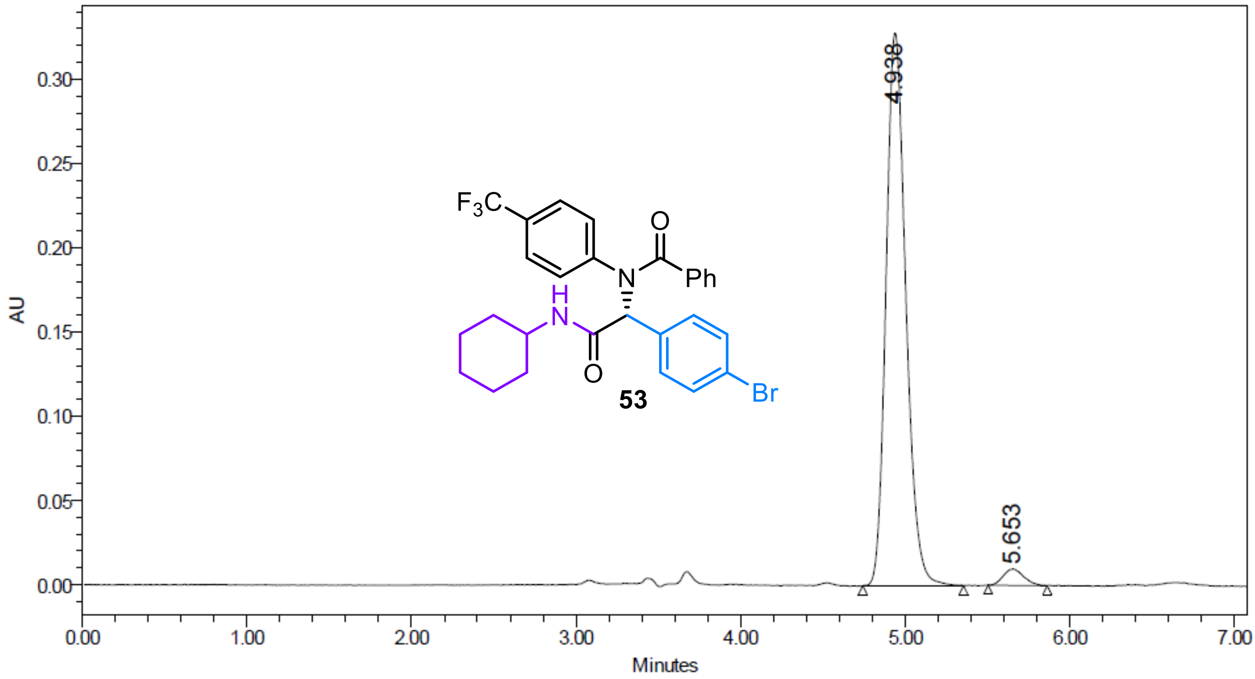

	RT (min)	Peak Type	Area (µV*sec)	% Area	Height (µV)	% Height	Integration Type	Points Across Peak	Start Time (min)	End Time (min)
1	4.938	Unknown	2737908	97.01	327986	97.12	BB	367	4.742	5.353
2	5.653	Unknown	84506	2.99	9714	2.88	BB	215	5.503	5.862

Supplementary Fig. 196. HPLC of product **53**.

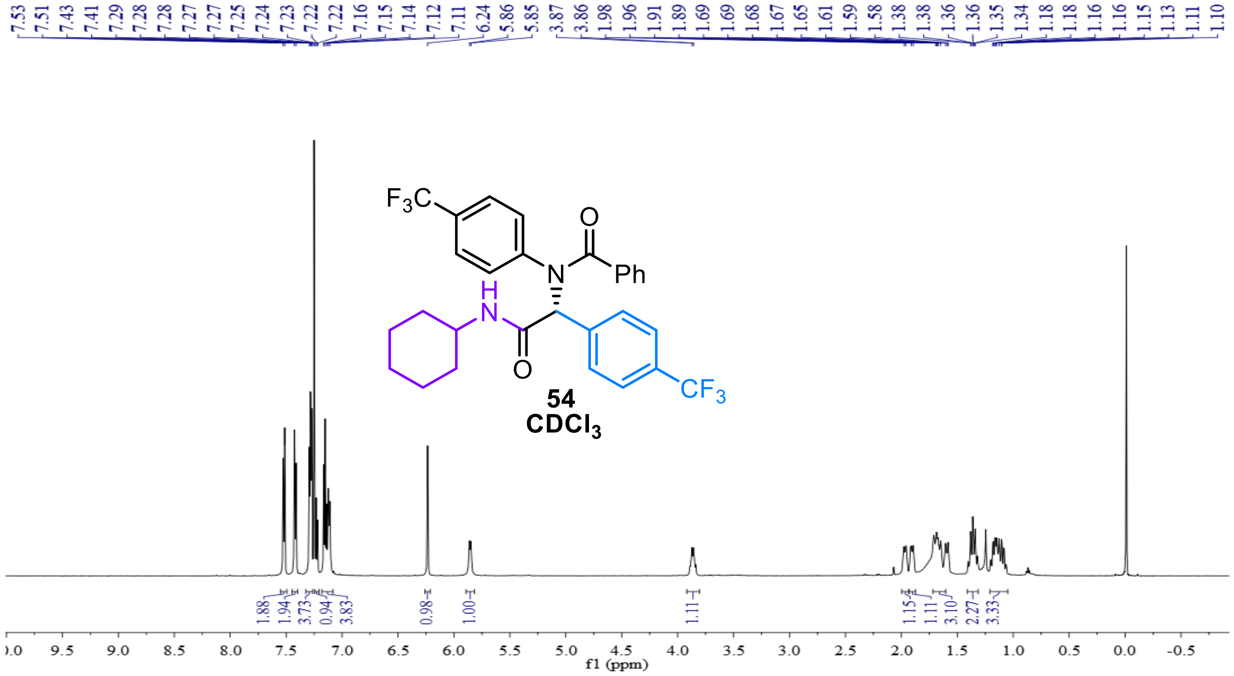

Supplementary Fig. 197. ^{1}H NMR spectrum of **54**. The sample has been recorded in 600 MHz, $CDCl_3$ at 25 °C.

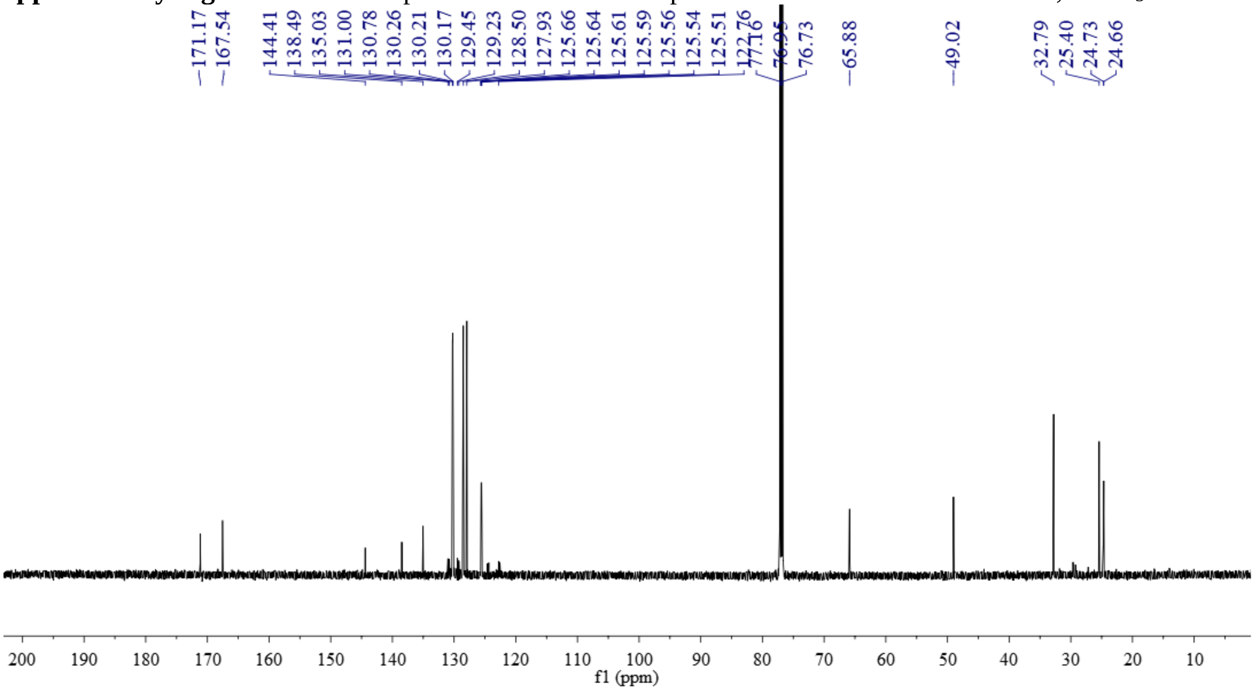

Supplementary Fig. 198. ^{13}C NMR spectrum of **54**. The sample has been recorded in 151 MHz, $CDCl_3$ at 25 °C.

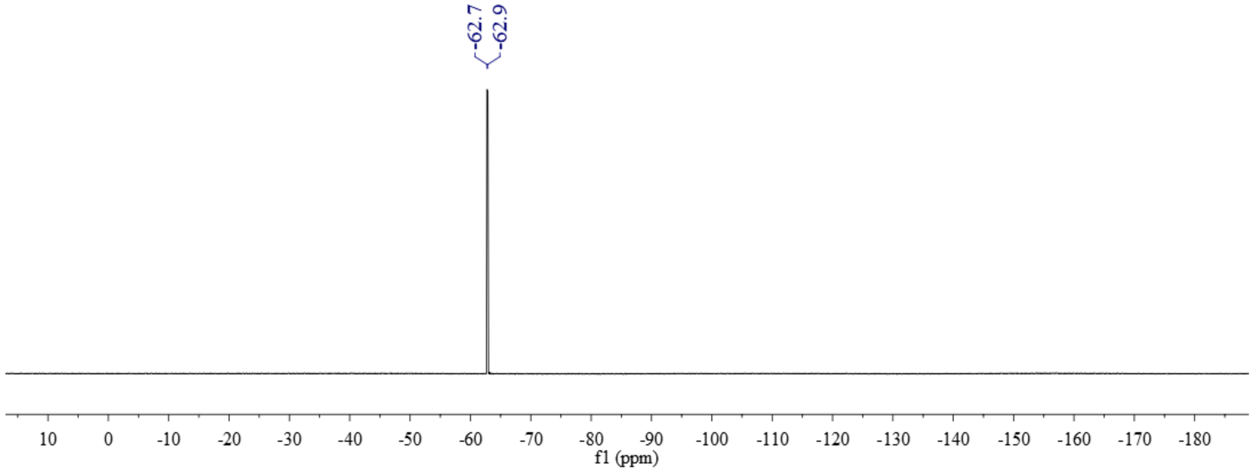

Supplementary Fig. 199. ^{31}F NMR spectrum of **54**. The sample has been recorded in 564 MHz, $CDCl_3$ at 25 °C.

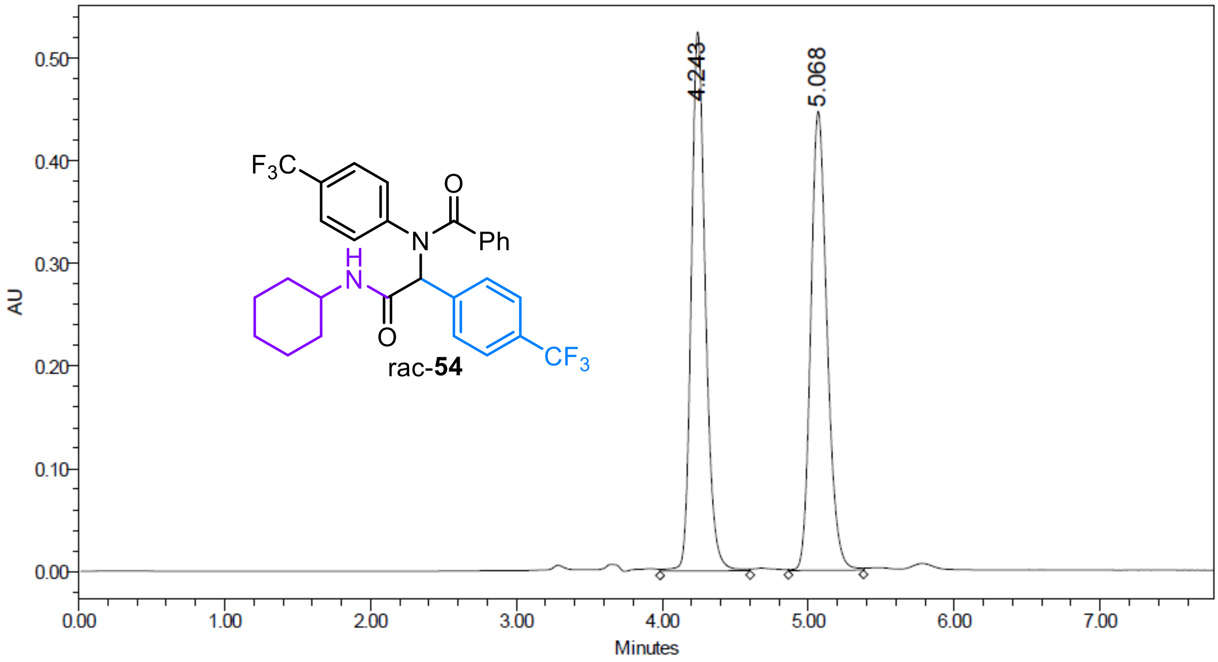

	RT (min)	Peak Type	Area (μV*sec)	% Area	Height (μV)	% Height	Integration Type	Points Across Peak	Start Time (min)	End Time (min)
1	4.243	Unknown	3537898	50.44	525069	54.01	VV	370	3.985	4.602
2	5.068	Unknown	3475568	49.56	447133	45.99	VV	309	4.863	5.378

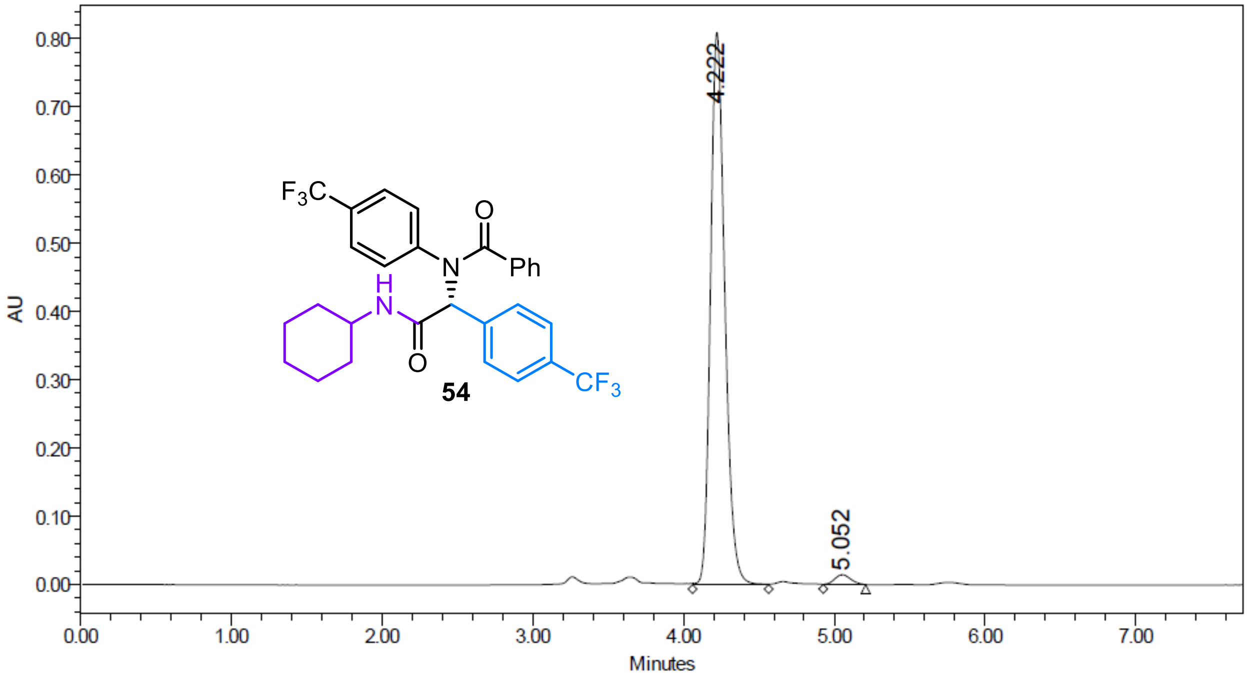

	RT (min)	Peak Type	Area (μV*sec)	% Area	Height (μV)	% Height	Integration Type	Points Across Peak	Start Time (min)	End Time (min)
1	4.222	Unknown	5334670	98.06	809876	98.26	VV	303	4.060	4.565
2	5.052	Unknown	105507	1.94	14341	1.74	Vb	169	4.927	5.208

Supplementary Fig. 200. HPLC of product **54**.

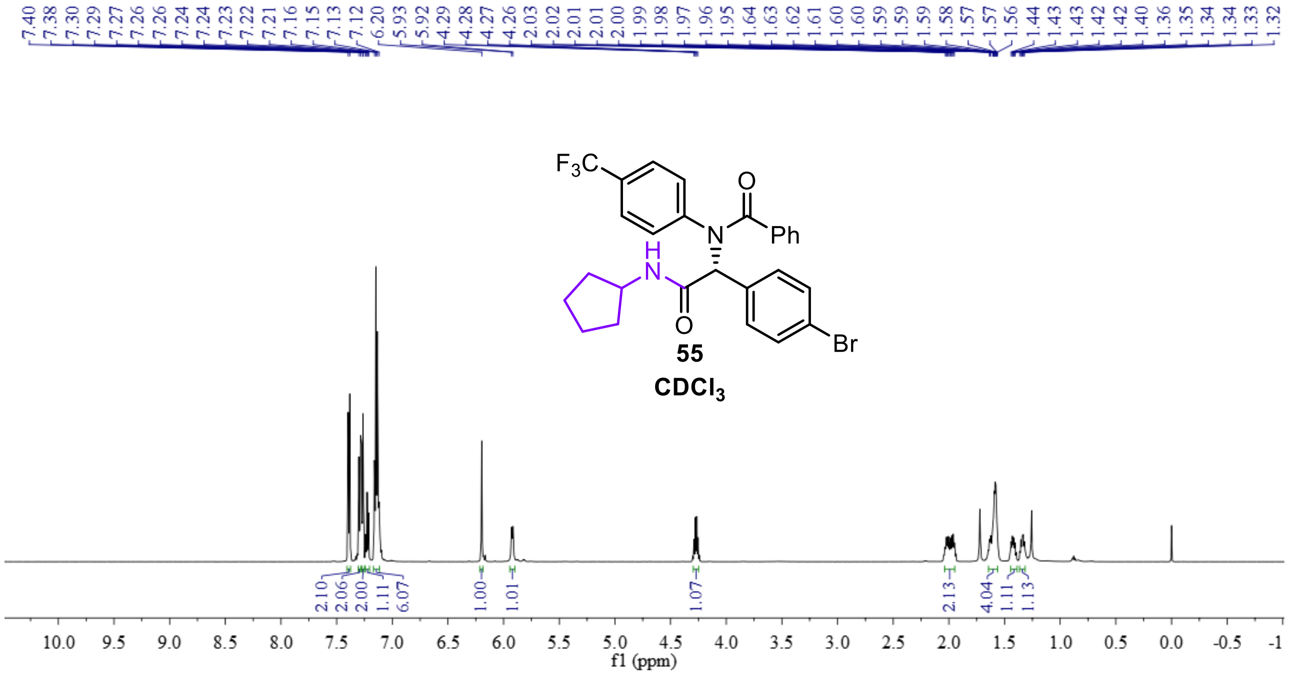

Supplementary Fig. 201. ^{1}H NMR spectrum of **55**. The sample has been recorded in 600 MHz, $CDCl_3$ at 25 °C.

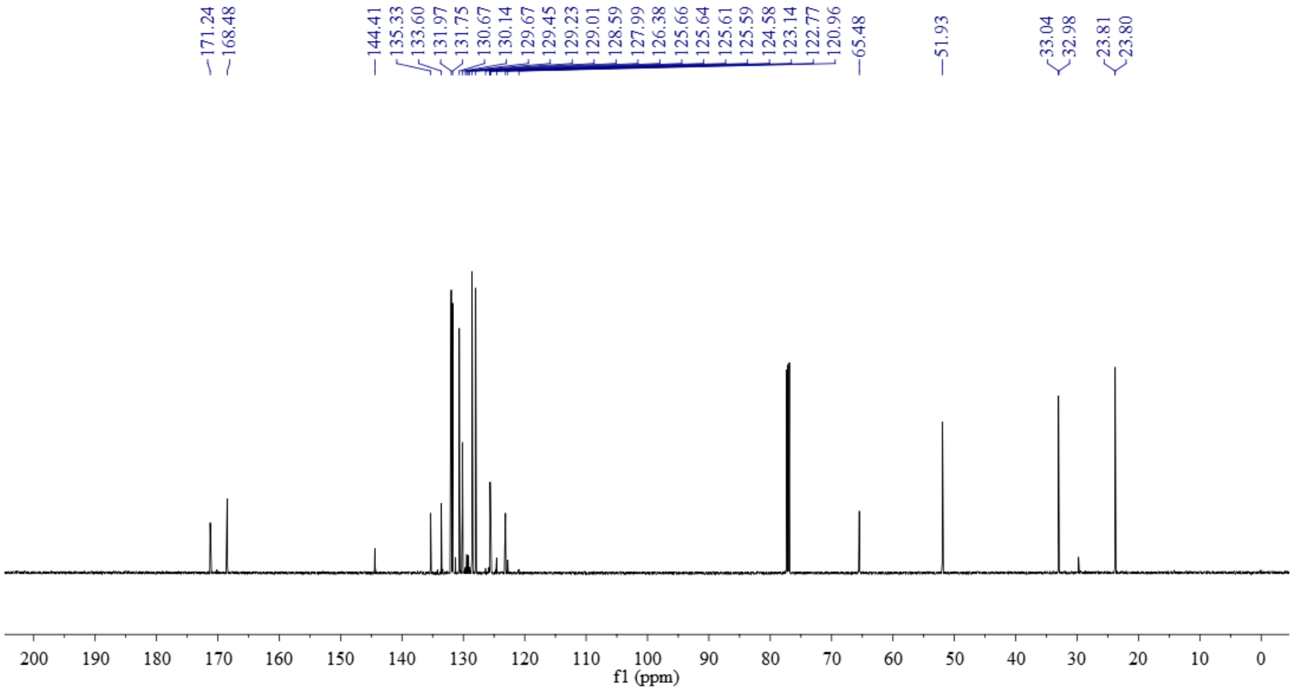

Supplementary Fig. 202. ^{13}C NMR spectrum of **55**. The sample has been recorded in 151 MHz, $CDCl_3$ at 25 °C.

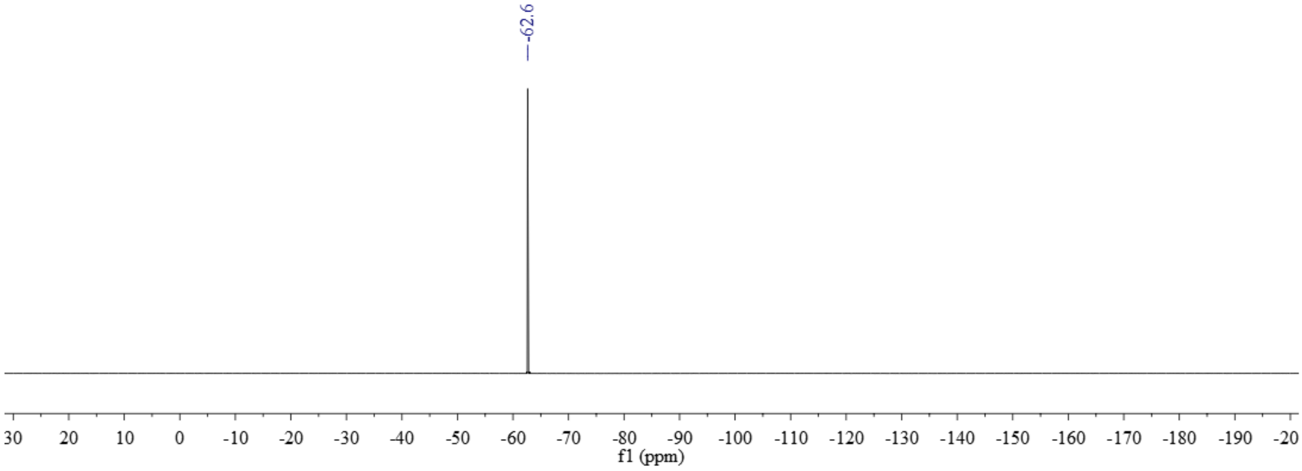

Supplementary Fig. 203. [31]F NMR spectrum of **55**. The sample has been recorded in 564 MHz, $CDCl_3$ at 25 °C.

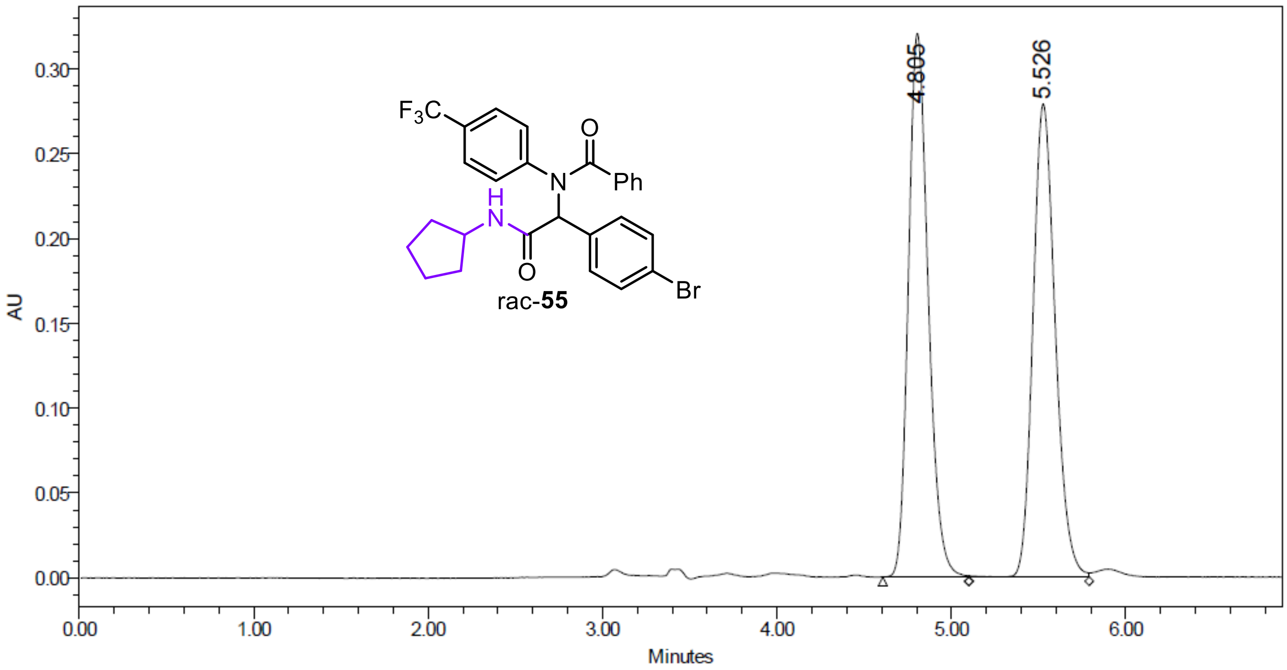

	RT (min)	Area (μV*sec)	% Area	Height (μV)	% Height
1	4.805	2523481	50.52	320312	53.47
2	5.526	2471832	49.48	278721	46.53

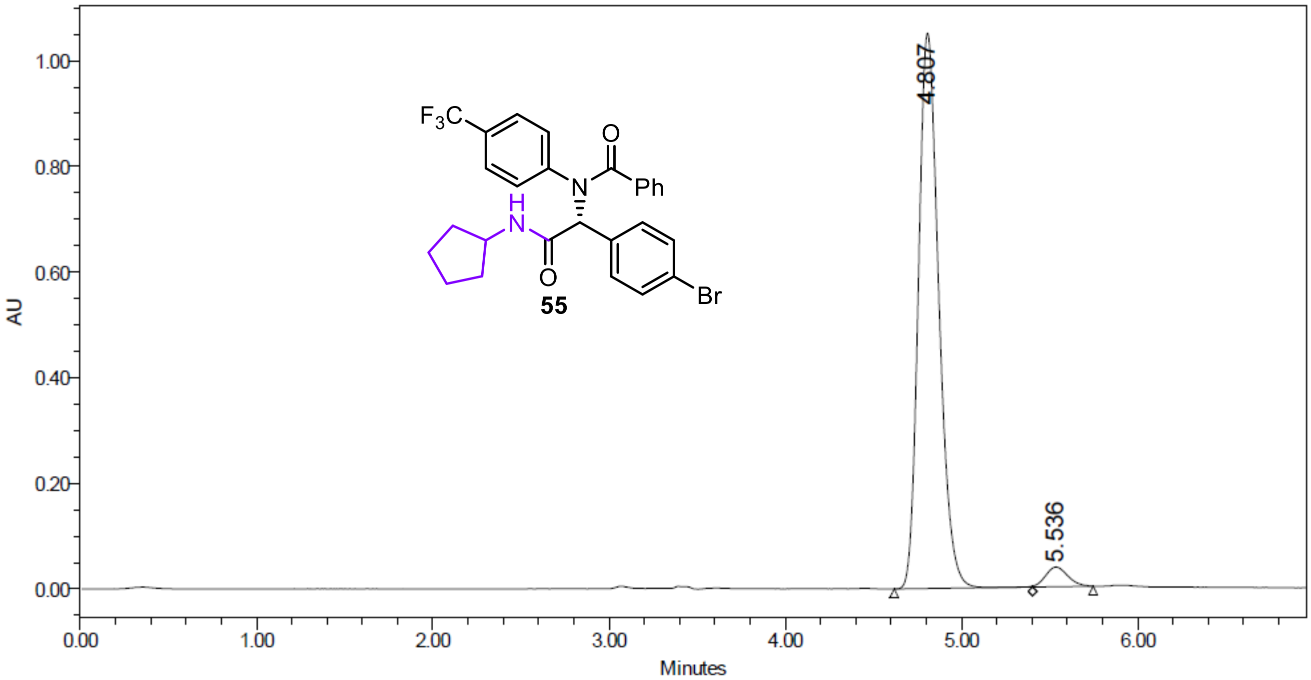

	RT (min)	Area (μV*sec)	% Area	Height (μV)	% Height
1	4.807	8427307	96.26	1051660	96.58
2	5.536	327053	3.74	37244	3.42

Supplementary Fig. 204. HPLC of product **55**.

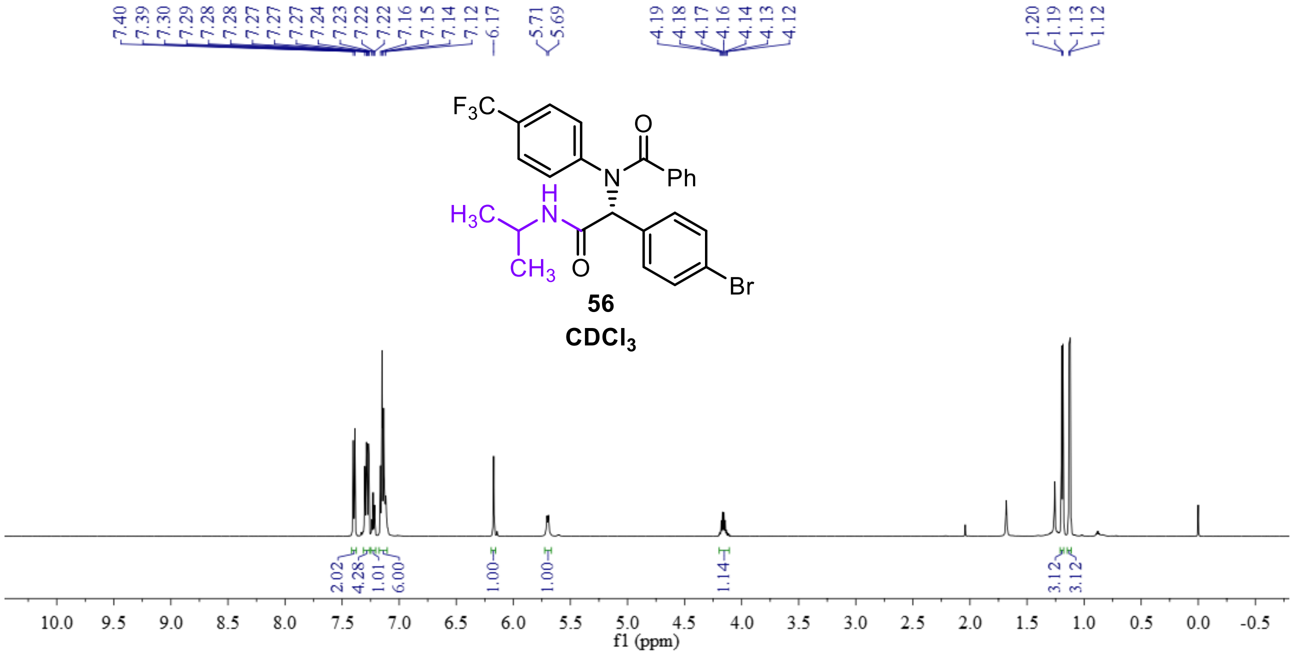

Supplementary Fig. 205. ^{1}H NMR spectrum of **56**. The sample has been recorded in 600 MHz, $CDCl_3$ at 25 °C.

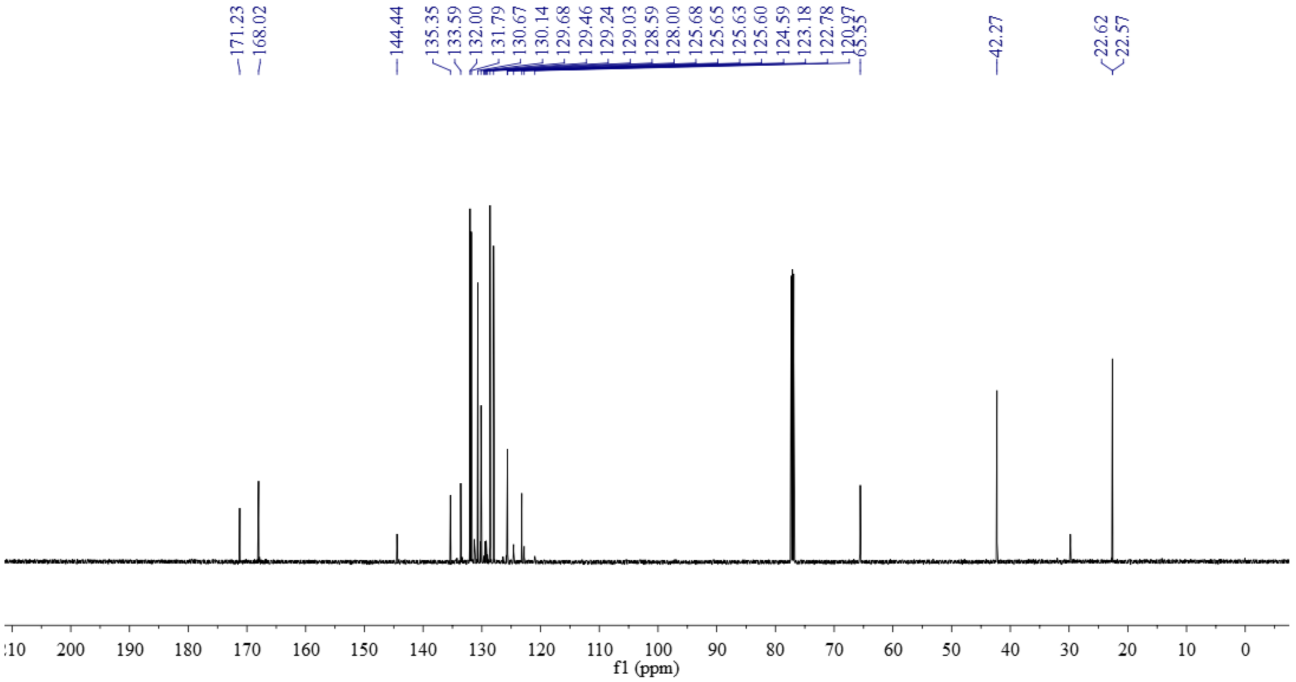

Supplementary Fig. 206. ^{13}C NMR spectrum of **56**. The sample has been recorded in 151 MHz, $CDCl_3$ at 25 °C.

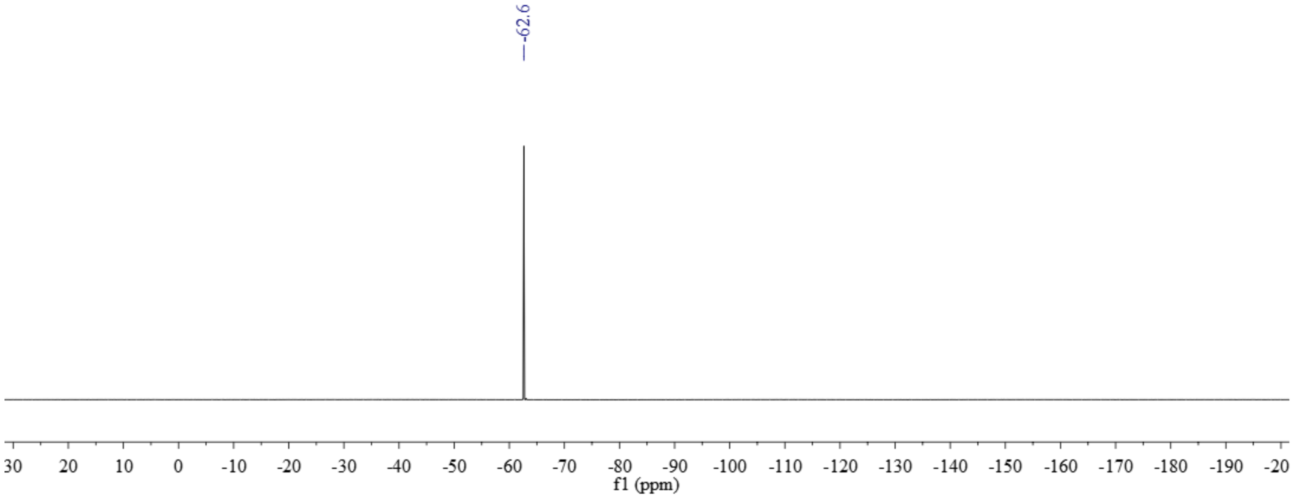

Supplementary Fig. 207. ^{31}F NMR spectrum of **56**. The sample has been recorded in 564 MHz, $CDCl_3$ at 25 °C.

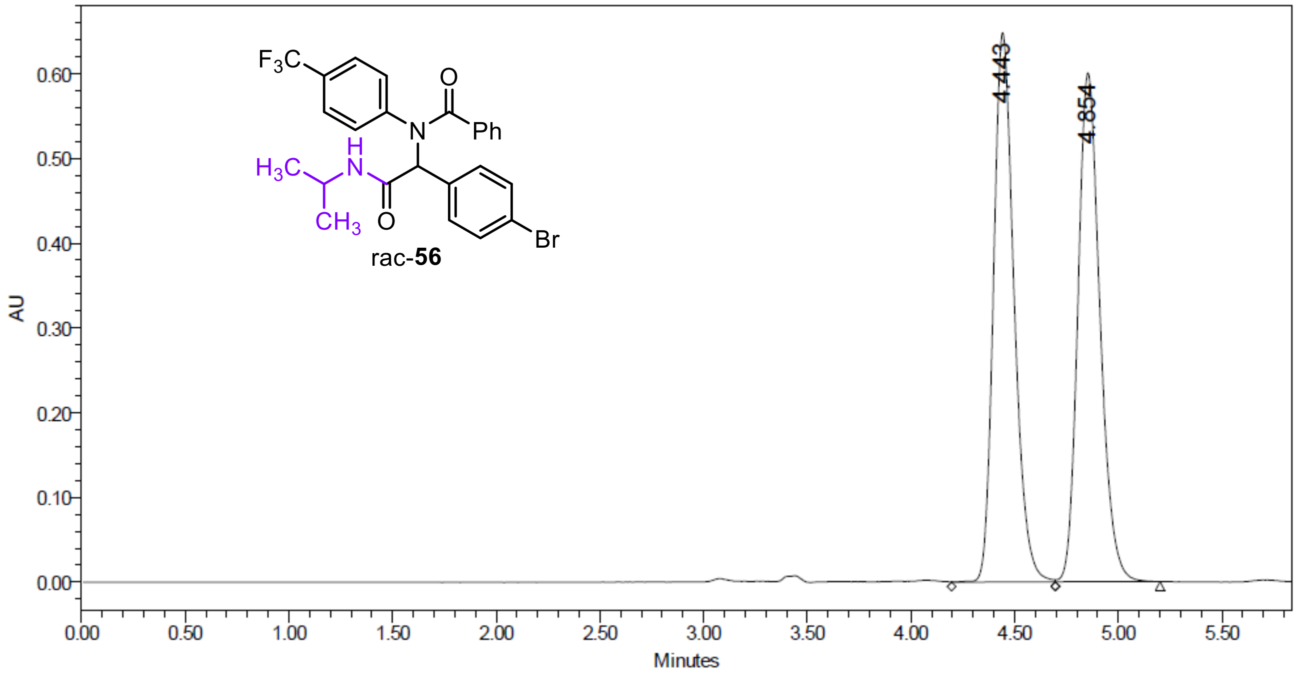

	RT (min)	Area (µV*sec)	% Area	Height (µV)	% Height
1	4.443	4453307	50.08	647989	51.91
2	4.854	4439041	49.92	600297	48.09

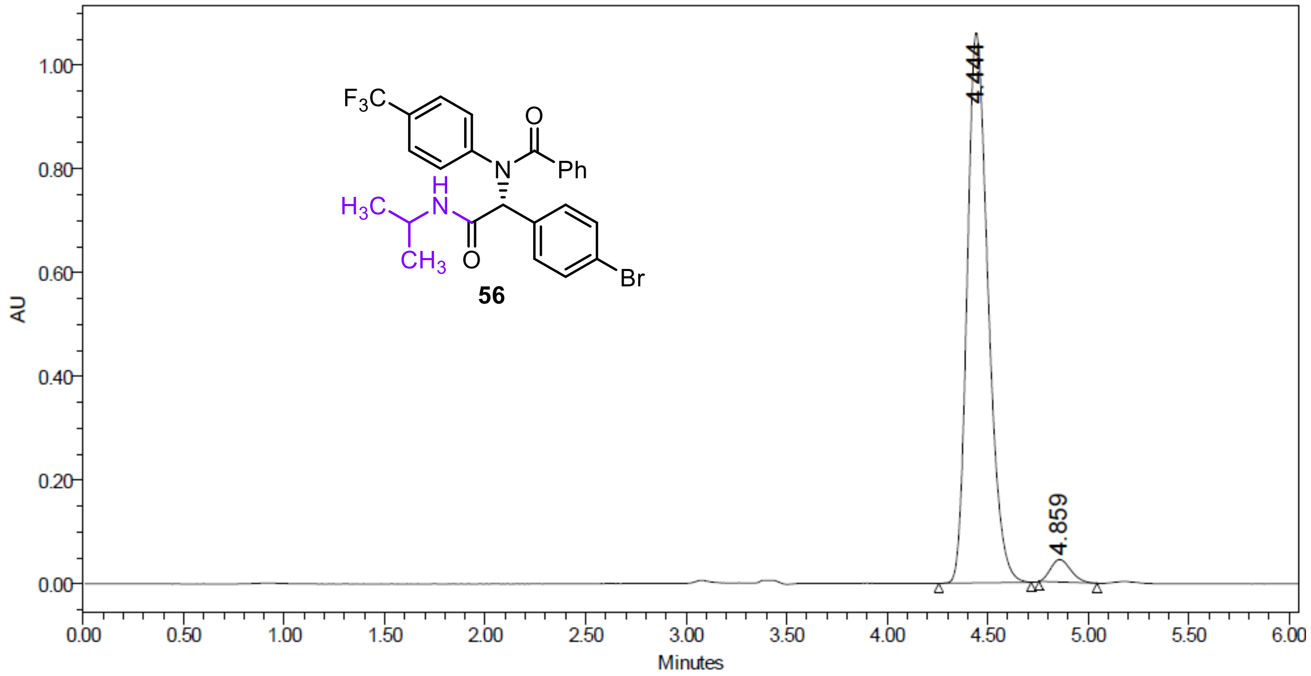

	RT (min)	Area (µV*sec)	% Area	Height (µV)	% Height
1	4.444	7662967	96.18	1060327	96.06
2	4.859	304196	3.82	43495	3.94

Supplementary Fig. 208. HPLC of product **56**.

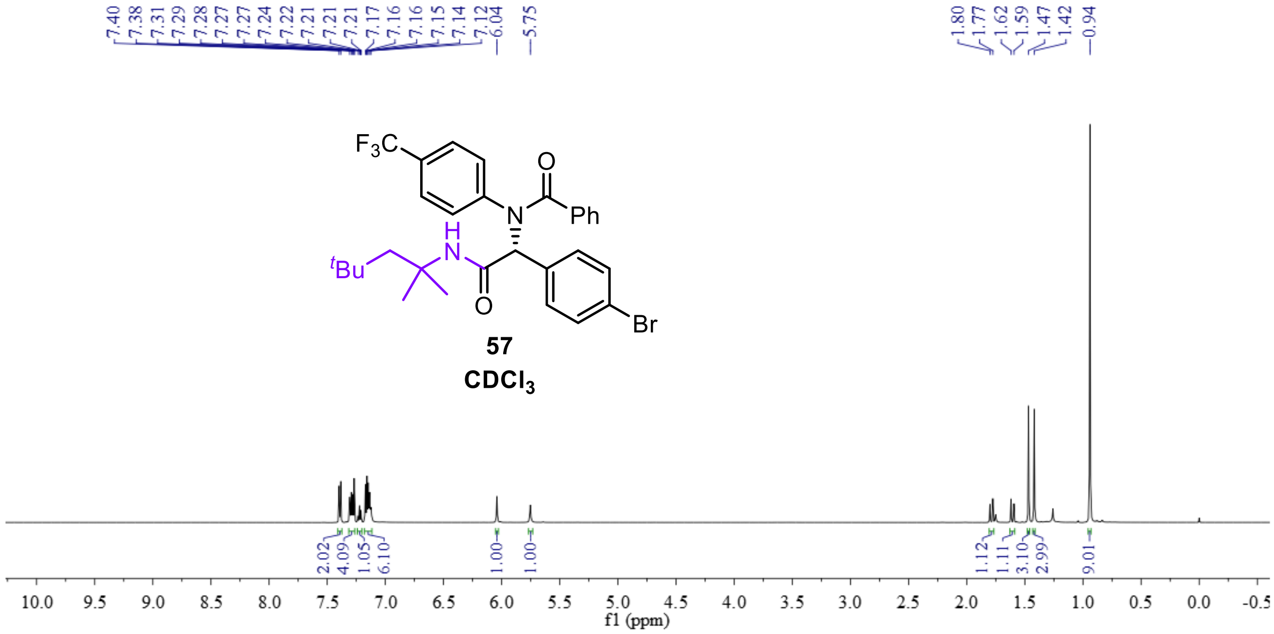

Supplementary Fig. 209. ^{1}H NMR spectrum of **57**. The sample has been recorded in 600 MHz, $CDCl_3$ at 25 °C.

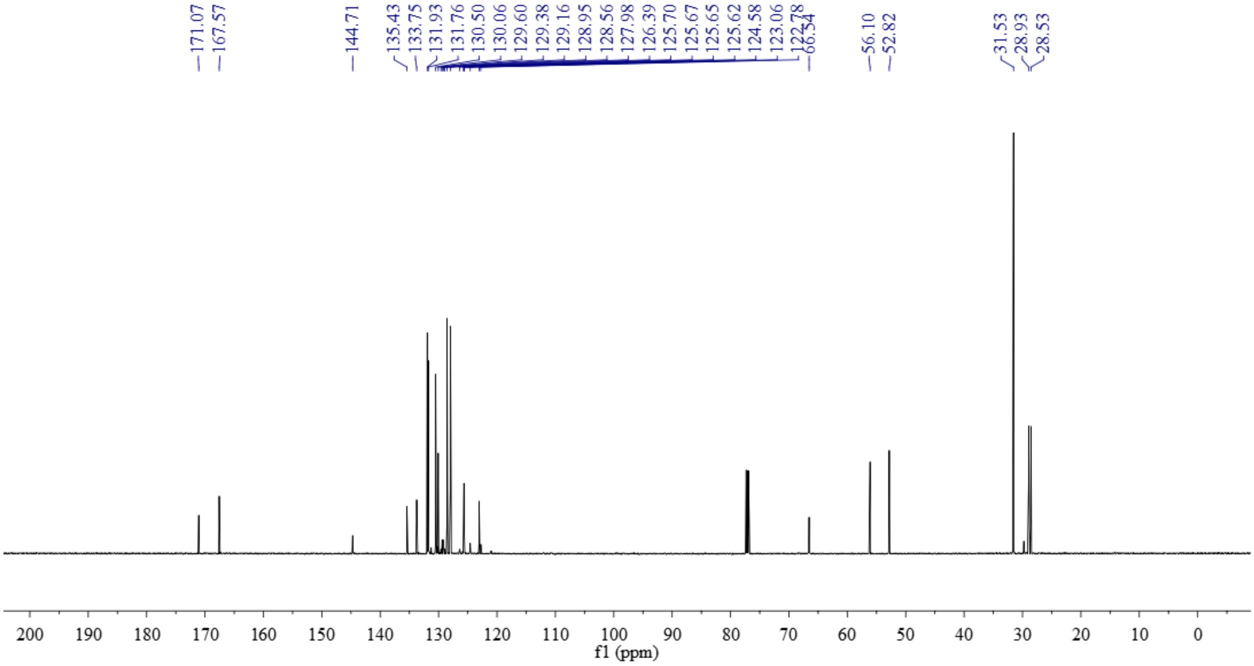

Supplementary Fig. 210. ^{13}C NMR spectrum of **57**. The sample has been recorded in 151 MHz, $CDCl_3$ at 25 °C.

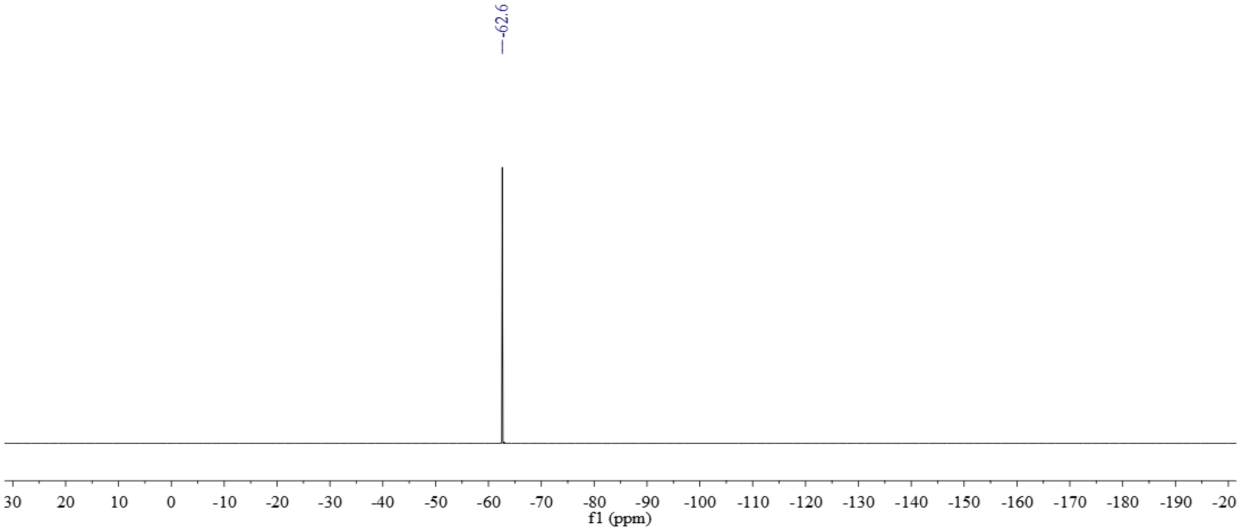

Supplementary Fig. 211. [31]F NMR spectrum of **57**. The sample has been recorded in 564 MHz, $CDCl_3$ at 25 °C.

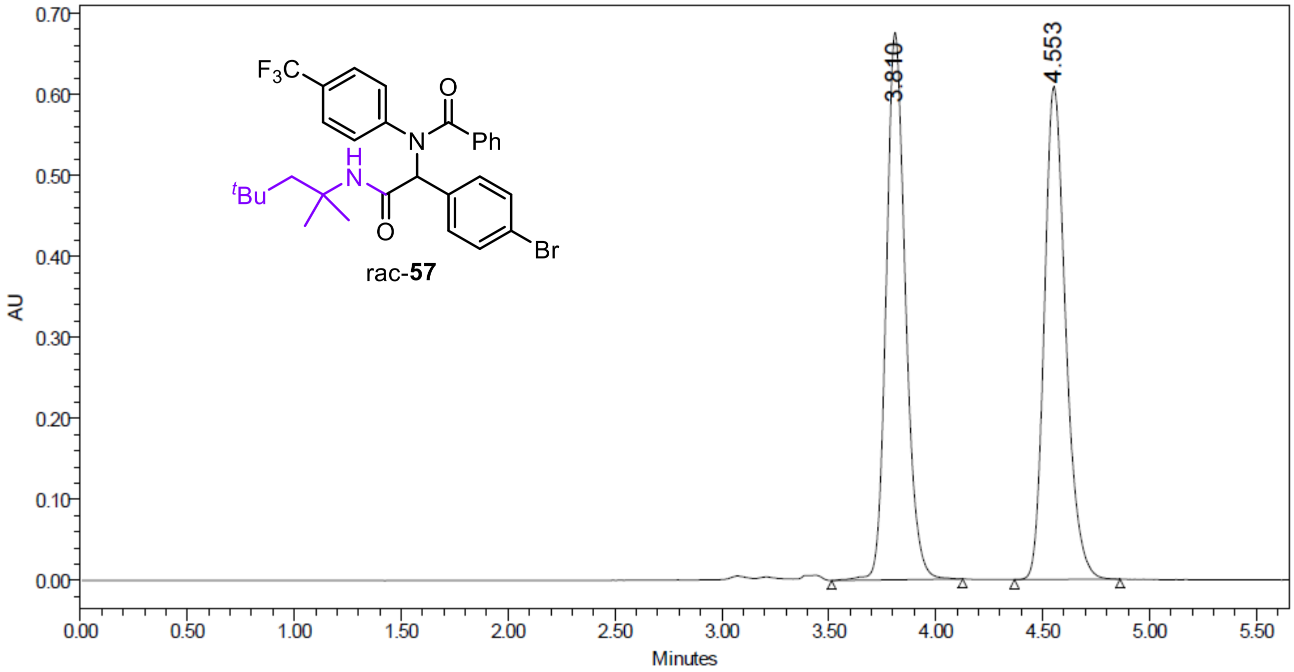

	RT (min)	Area (μV*sec)	% Area	Height (μV)	% Height
1	3.810	4224606	50.04	675346	52.63
2	4.553	4217380	49.96	607822	47.37

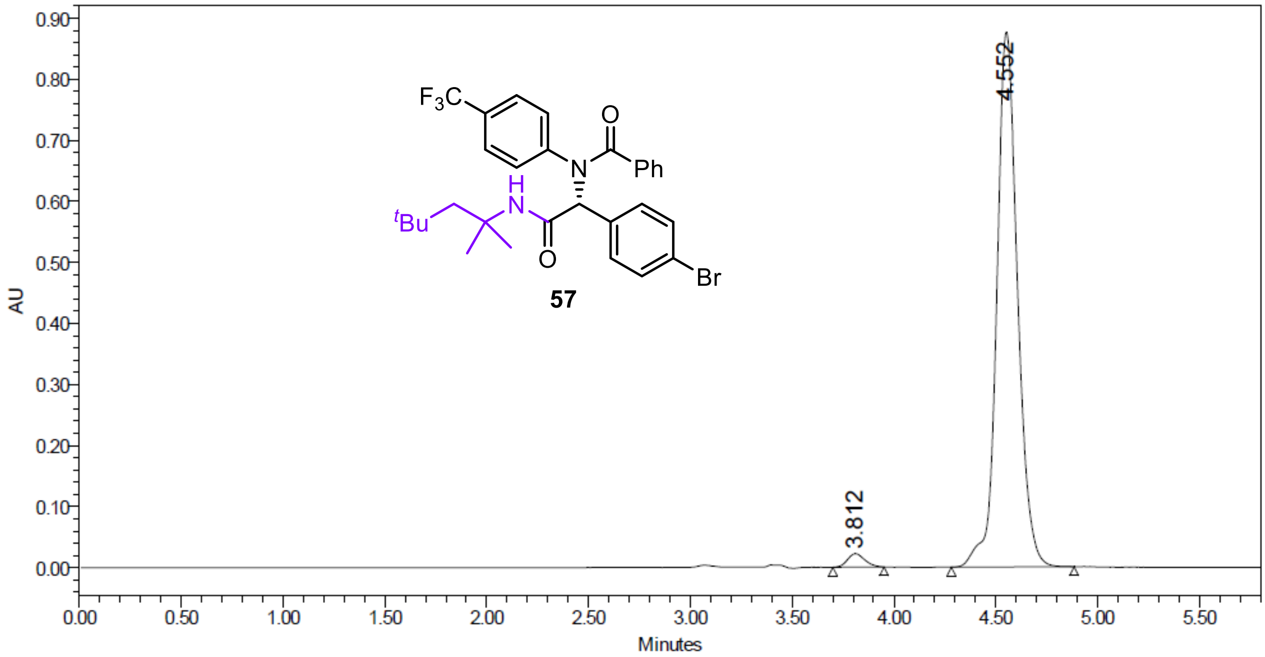

	RT (min)	Area (μV*sec)	% Area	Height (μV)	% Height
1	3.812	128813	2.03	21903	2.44
2	4.552	6226619	97.97	876242	97.56

Supplementary Fig. 212. HPLC of product **57**.

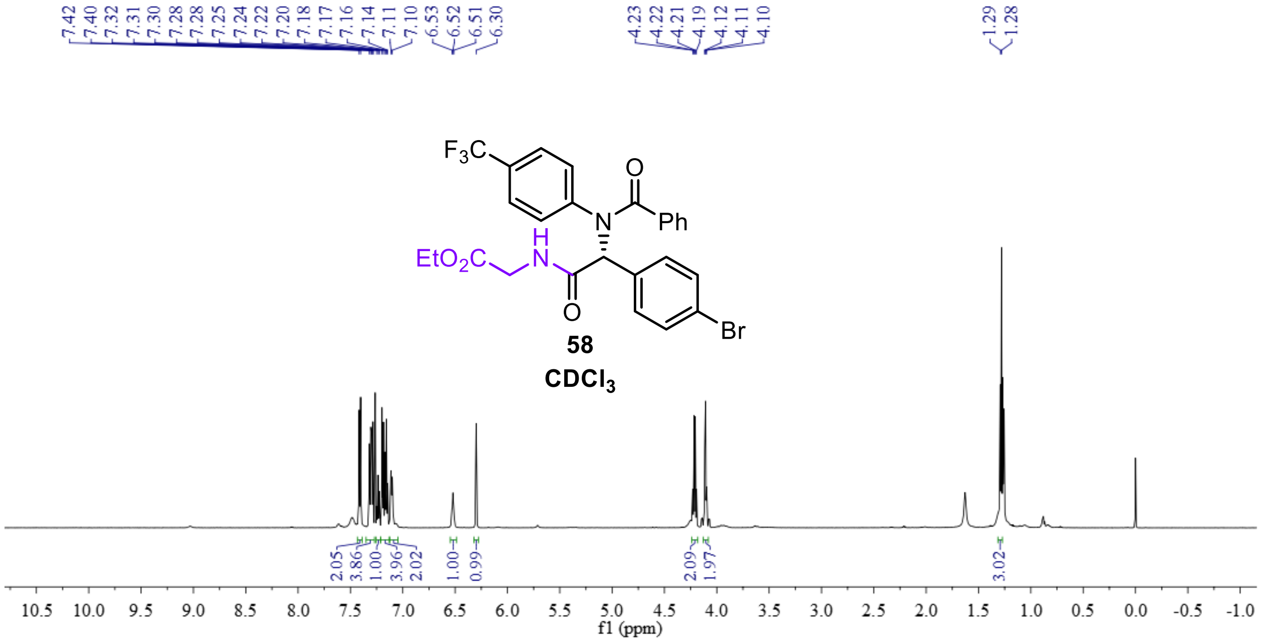

Supplementary Fig. 213. ^{1}H NMR spectrum of **58**. The sample has been recorded in 600 MHz, $CDCl_3$ at 25 °C.

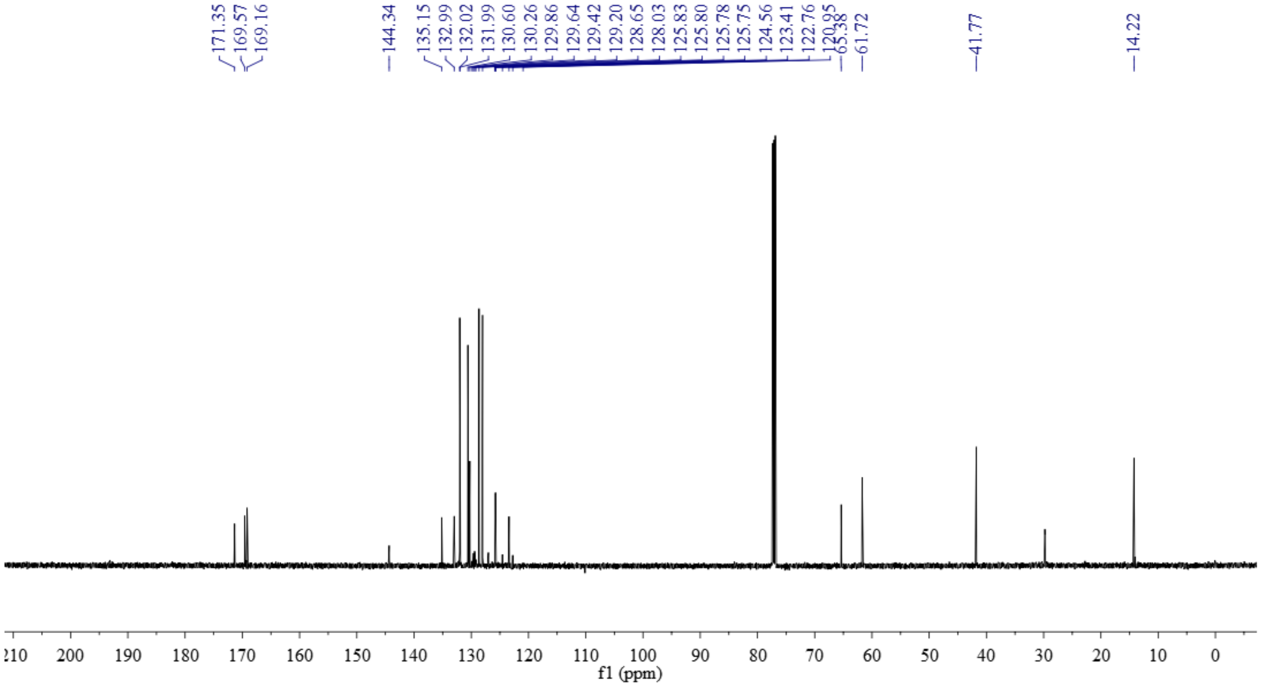

Supplementary Fig. 214. ^{13}C NMR spectrum of **58**. The sample has been recorded in 151 MHz, $CDCl_3$ at 25 °C.

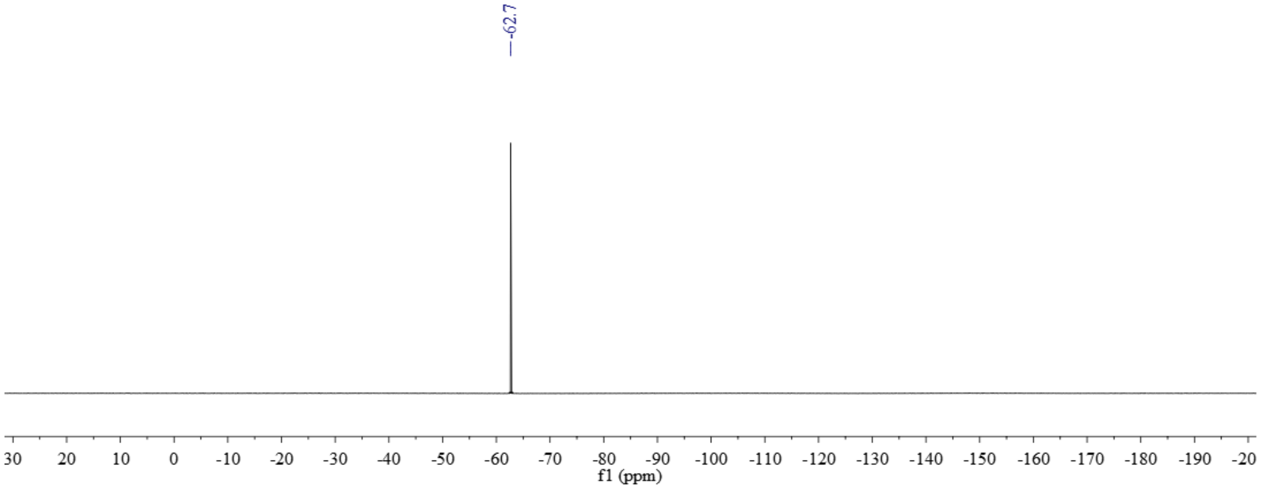

Supplementary Fig. 215. ^{31}F NMR spectrum of **58**. The sample has been recorded in 564 MHz, $CDCl_3$ at 25 °C.

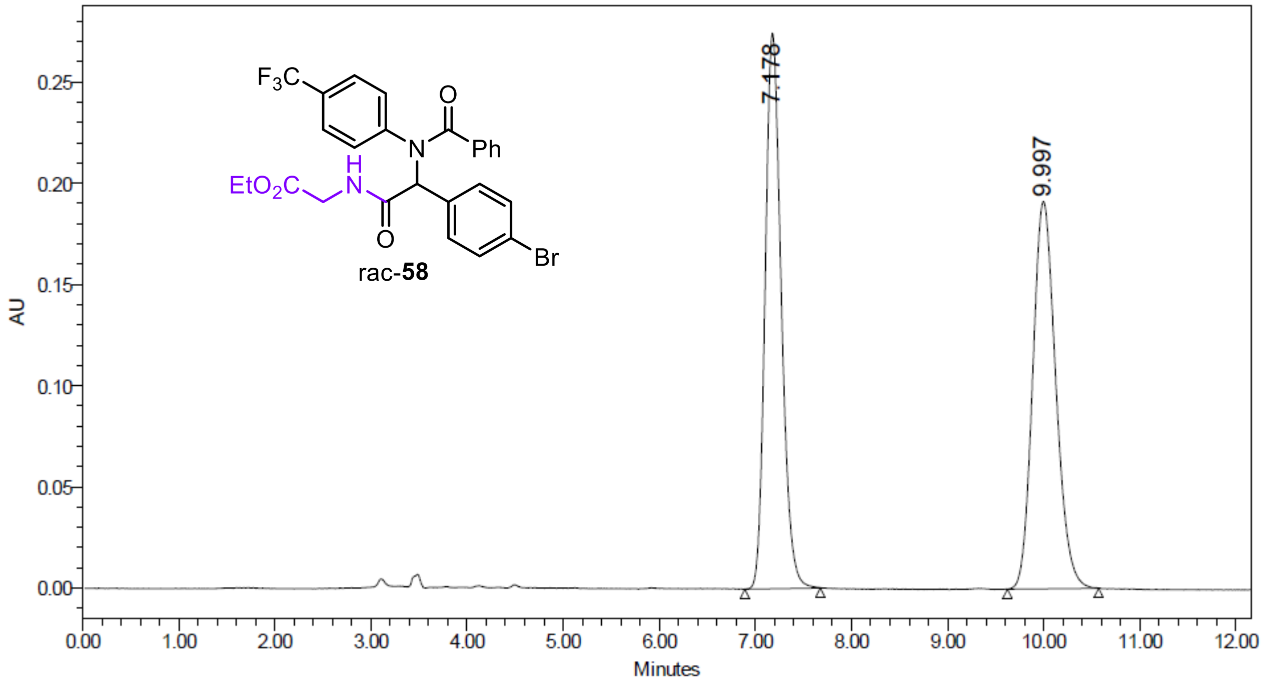

	RT (min)	Peak Type	Area (µV*sec)	% Area	Height (µV)	% Height	Integration Type	Points Across Peak	Start Time (min)	End Time (min)
1	7.178	Unknown	3202152	50.06	274631	58.93	BB	472	6.890	7.677
2	9.997	Unknown	3194860	49.94	191433	41.07	BB	570	9.622	10.572

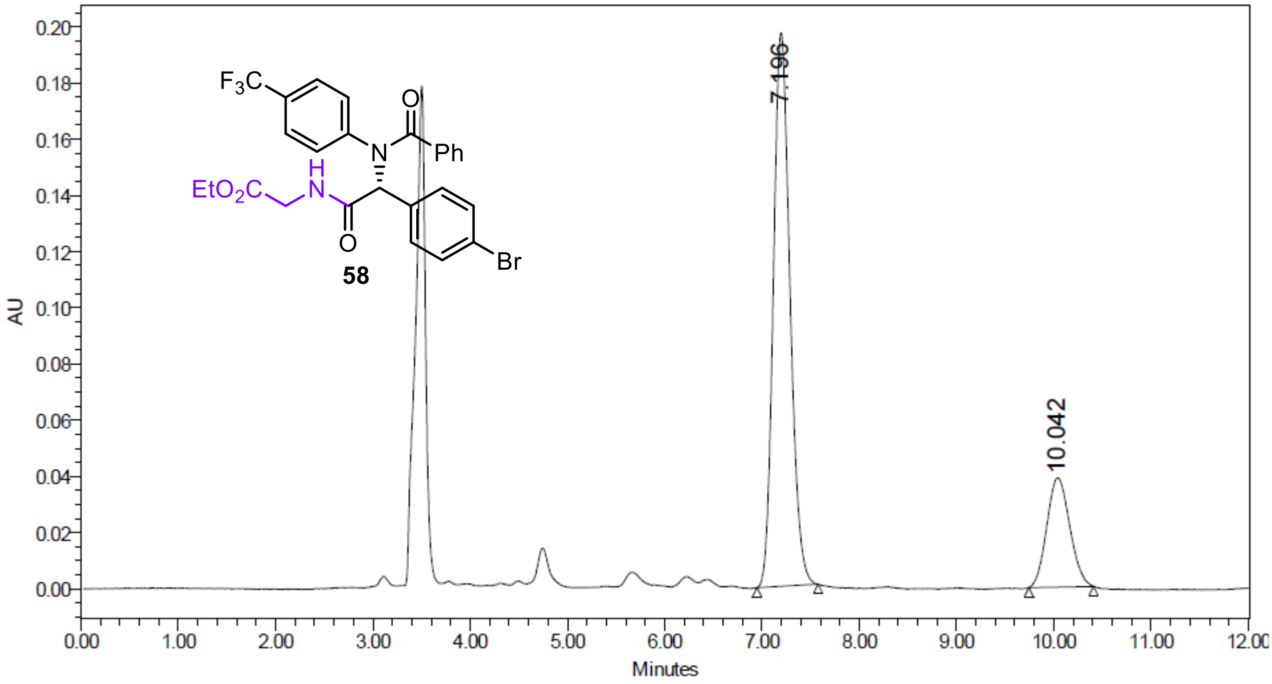

	RT (min)	Peak Type	Area (µV*sec)	% Area	Height (µV)	% Height	Integration Type	Points Across Peak	Start Time (min)	End Time (min)
1	7.196	Unknown	2332162	78.46	196959	83.50	BB	376	6.950	7.577
2	10.042	Unknown	640238	21.54	38913	16.50	Bb	398	9.745	10.408

Supplementary Fig. 216. HPLC of product **58**.

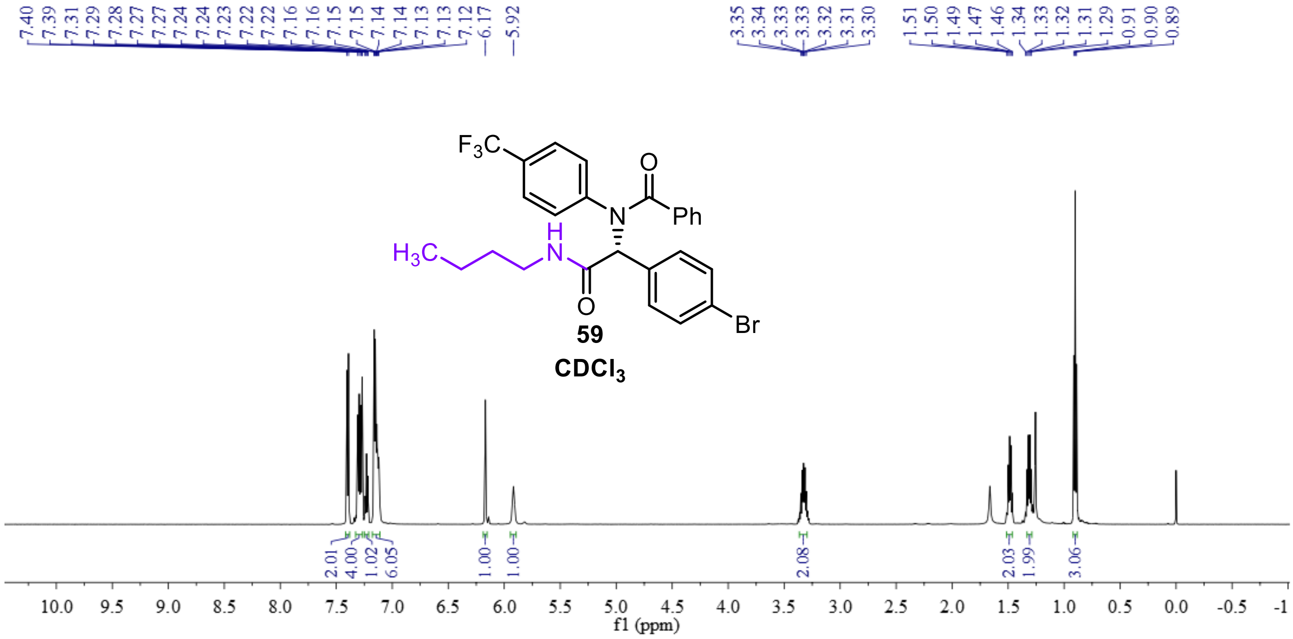

Supplementary Fig. 217. ^{1}H NMR spectrum of **59**. The sample has been recorded in 600 MHz, $CDCl_3$ at 25 °C.

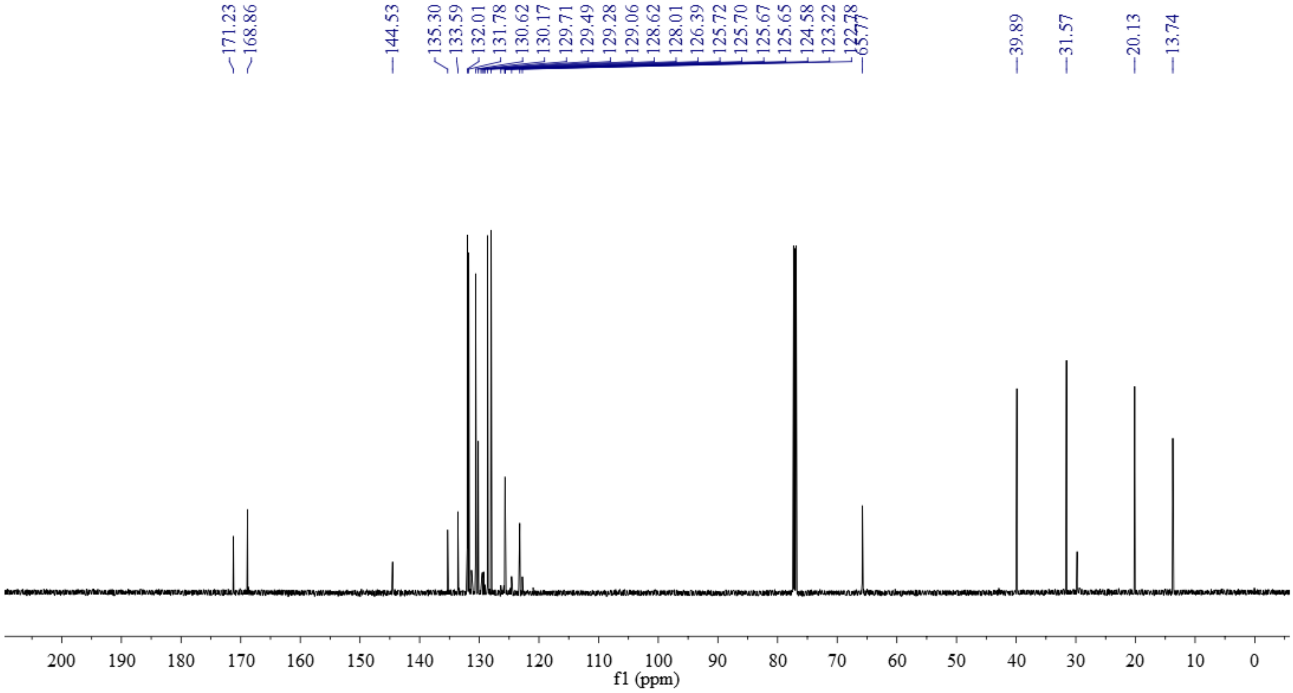

Supplementary Fig. 218. ^{13}C NMR spectrum of **59**. The sample has been recorded in 151 MHz, $CDCl_3$ at 25 °C.

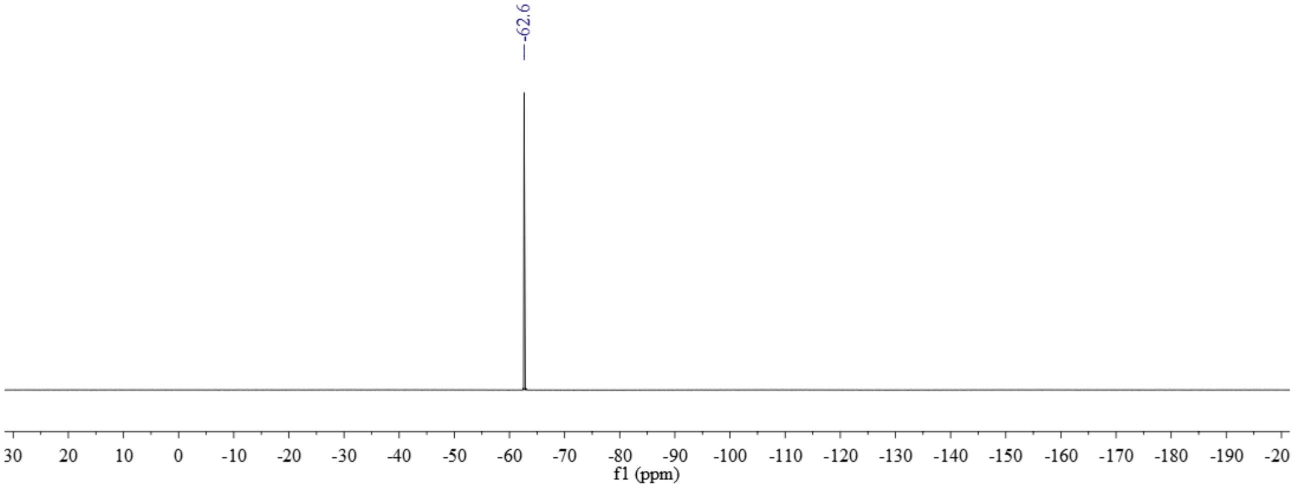

Supplementary Fig. 219. ^{31}F NMR spectrum of **59**. The sample has been recorded in 564 MHz, $CDCl_3$ at 25 °C.

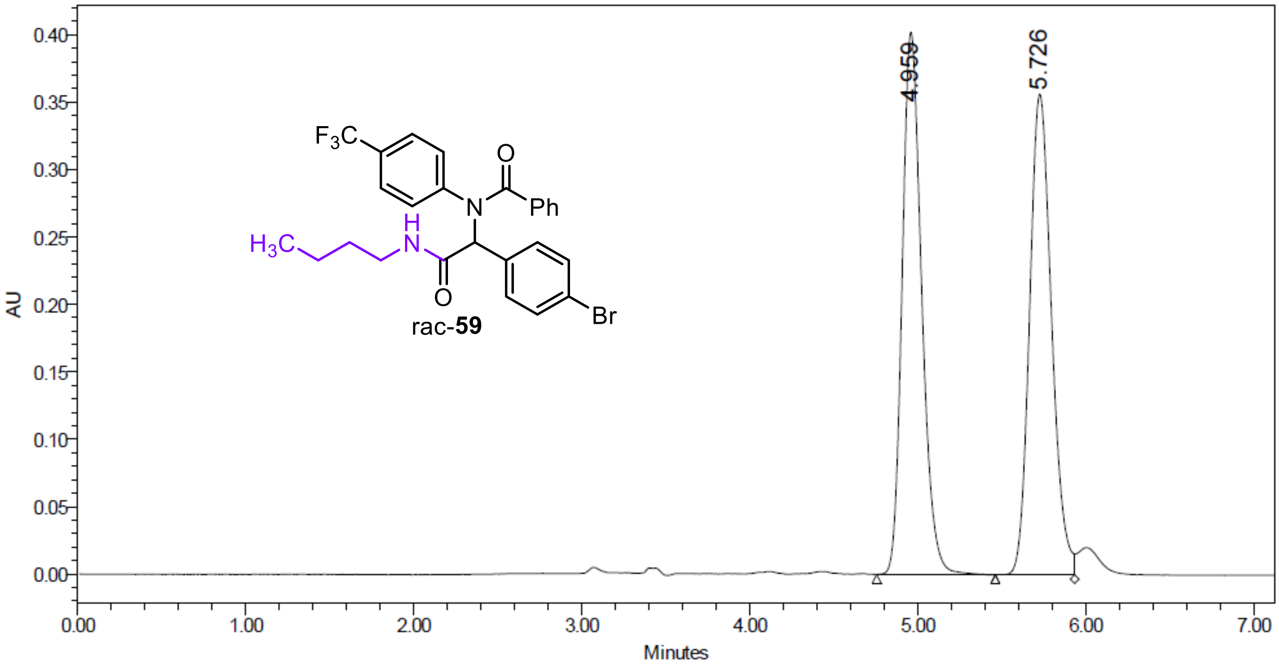

	RT (min)	Area (μV*sec)	% Area	Height (μV)	% Height
1	4.959	3239347	50.02	402211	53.01
2	5.726	3236706	49.98	356521	46.99

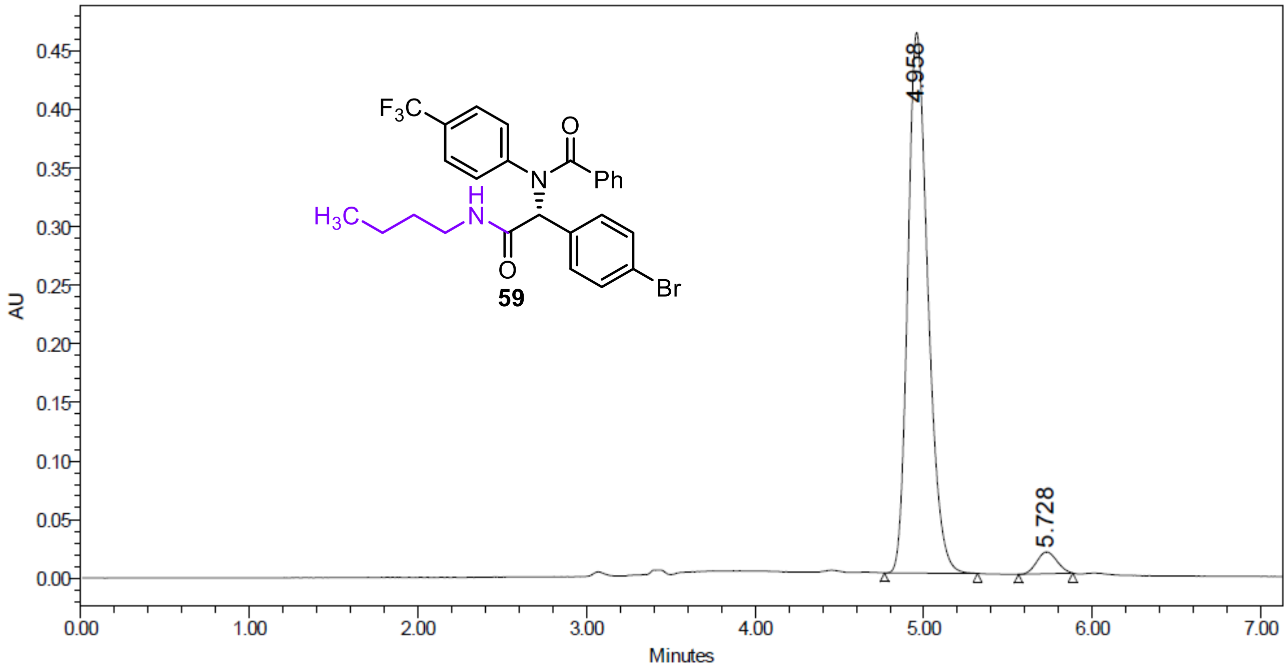

	RT (min)	Area (μV*sec)	% Area	Height (μV)	% Height
1	4.958	3828609	96.11	461410	96.13
2	5.728	154784	3.89	18577	3.87

Supplementary Fig. 220. HPLC of product **59**.

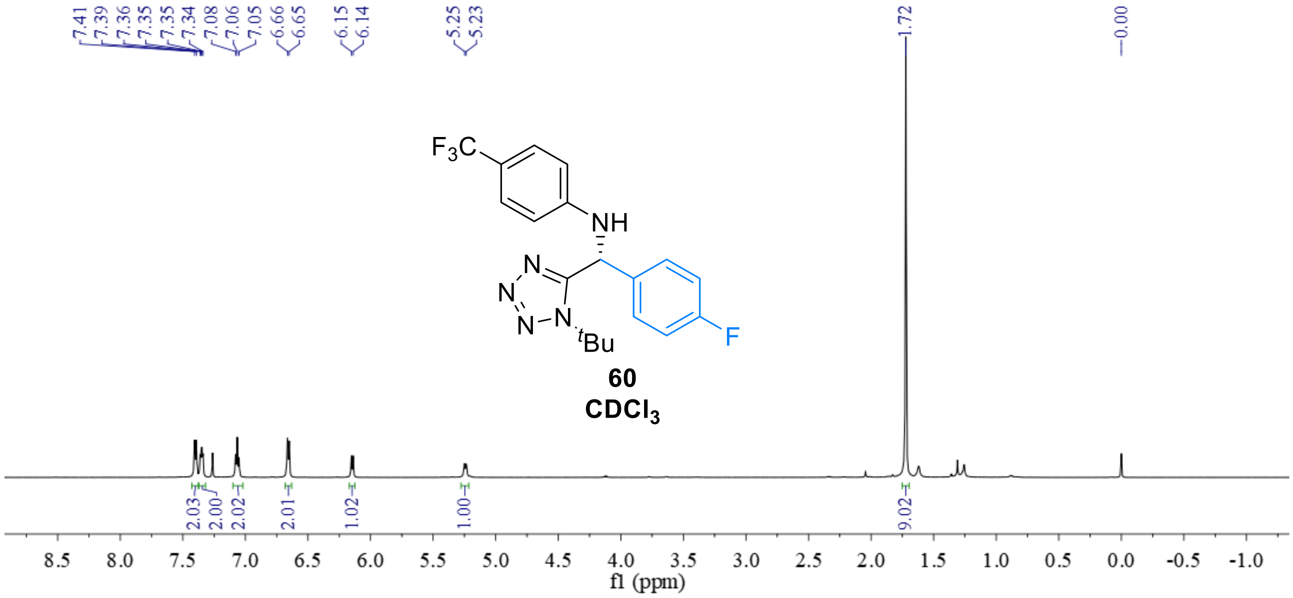

Supplementary Fig. 221. ^{1}H NMR spectrum of **60**. The sample has been recorded in 600 MHz, $CDCl_3$ at 25 °C.

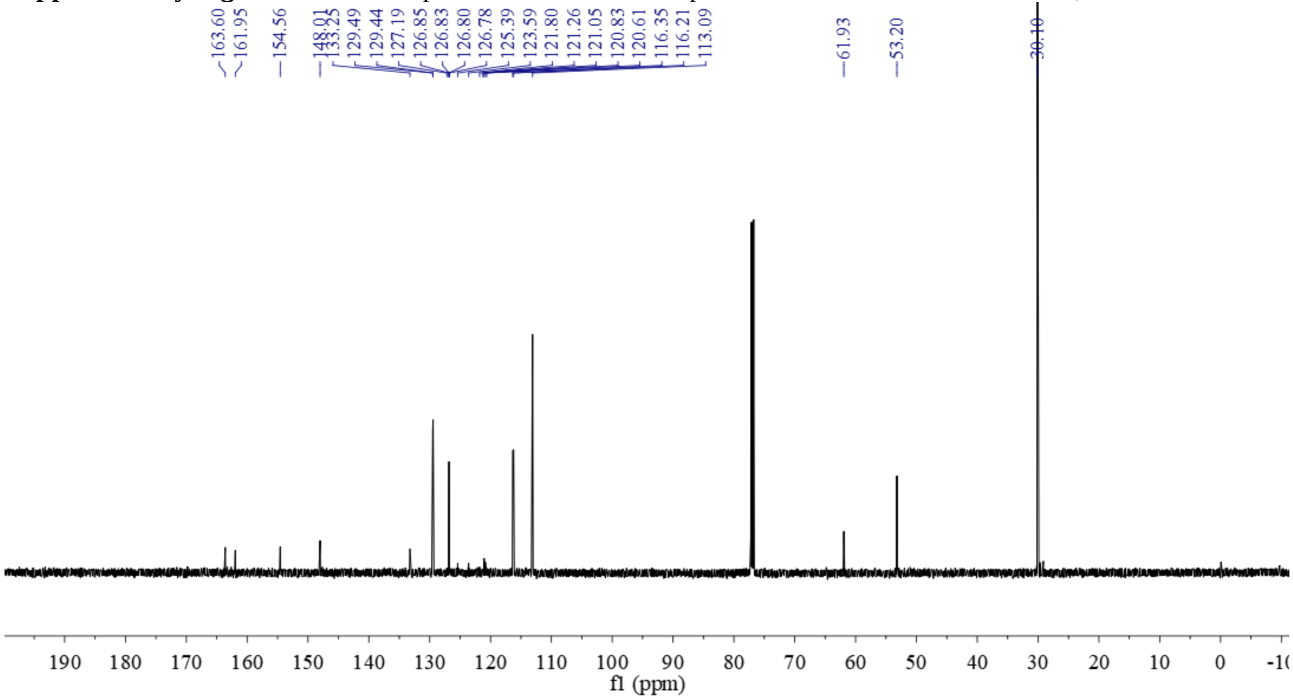

Supplementary Fig. 222. ^{13}C NMR spectrum of **60**. The sample has been recorded in 151 MHz, $CDCl_3$ at 25 °C.

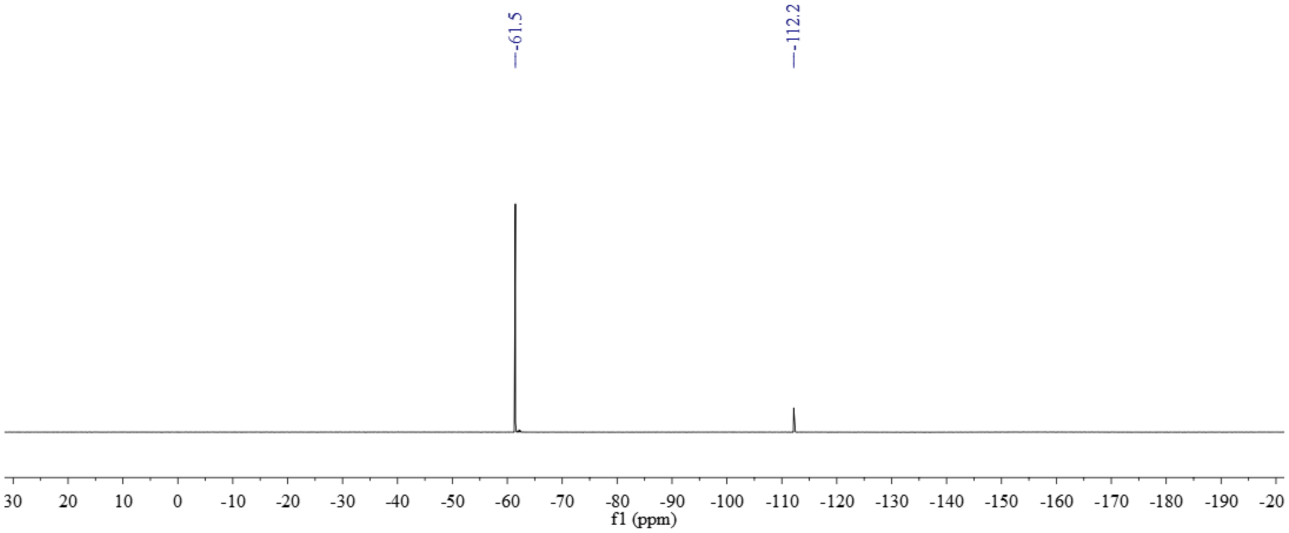

Supplementary Fig. 223. ^{31}F NMR spectrum of **60**. The sample has been recorded in 564 MHz, $CDCl_3$ at 25 °C.

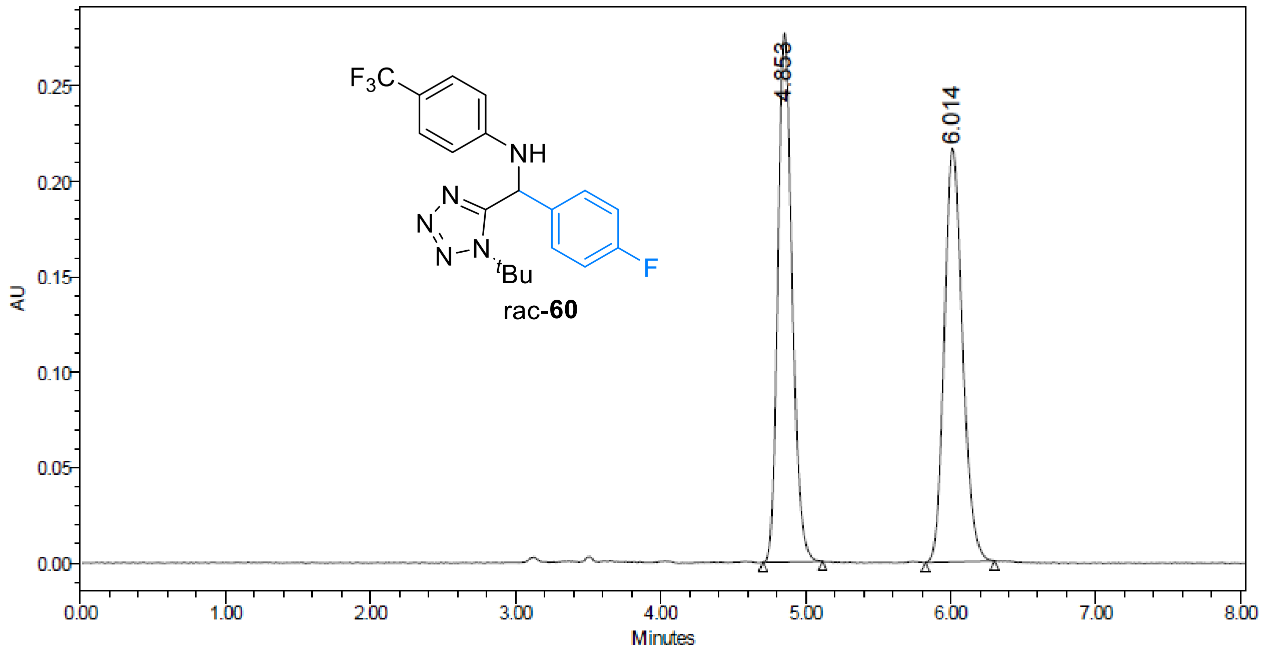

	RT (min)	Area (µV*sec)	% Area	Height (µV)	% Height
1	4.853	1902663	50.14	277309	56.13
2	6.014	1891873	49.86	216699	43.87

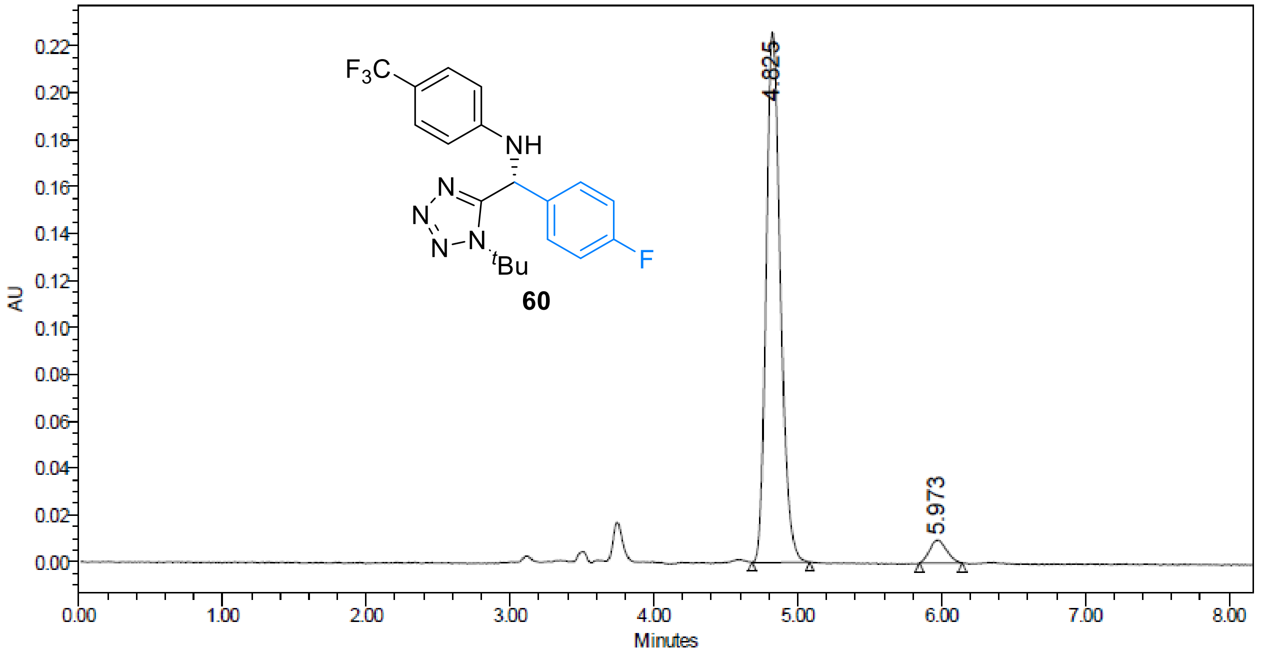

	RT (min)	Area (µV*sec)	% Area	Height (µV)	% Height
1	4.825	1567424	95.07	226142	95.79
2	5.973	81285	4.93	9936	4.21

Supplementary Fig. 224. HPLC of product **60**.

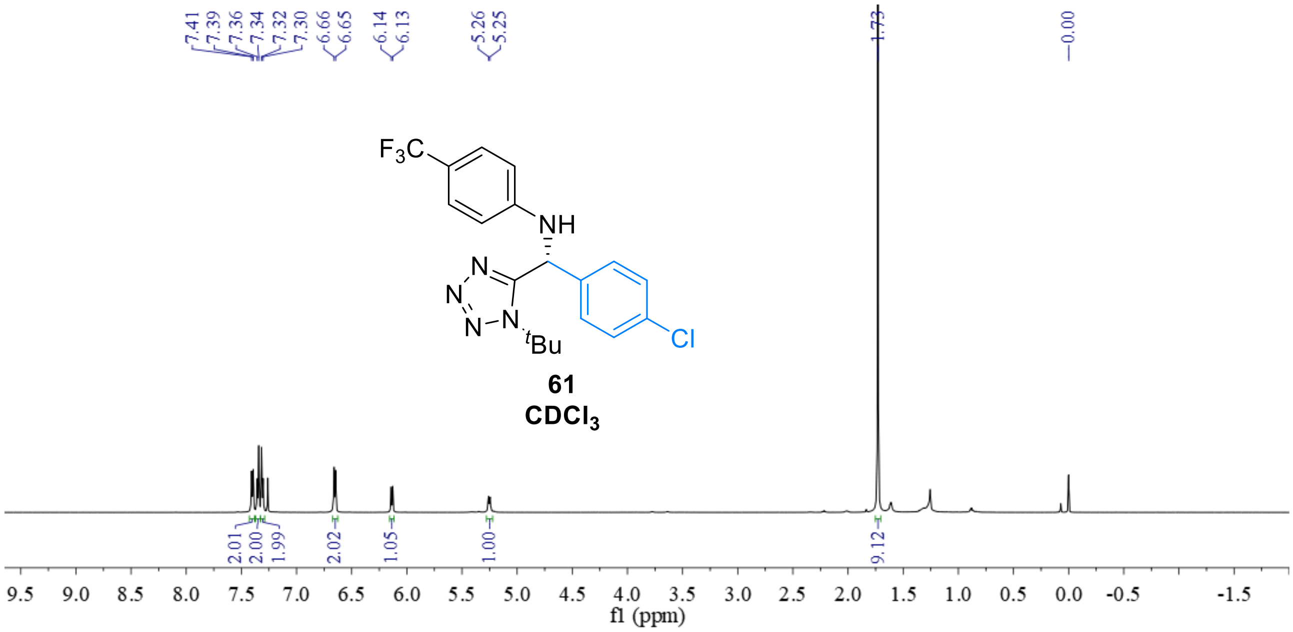

Supplementary Fig. 225. ^{1}H NMR spectrum of **61**. The sample has been recorded in 600 MHz, $CDCl_3$ at 25 °C.

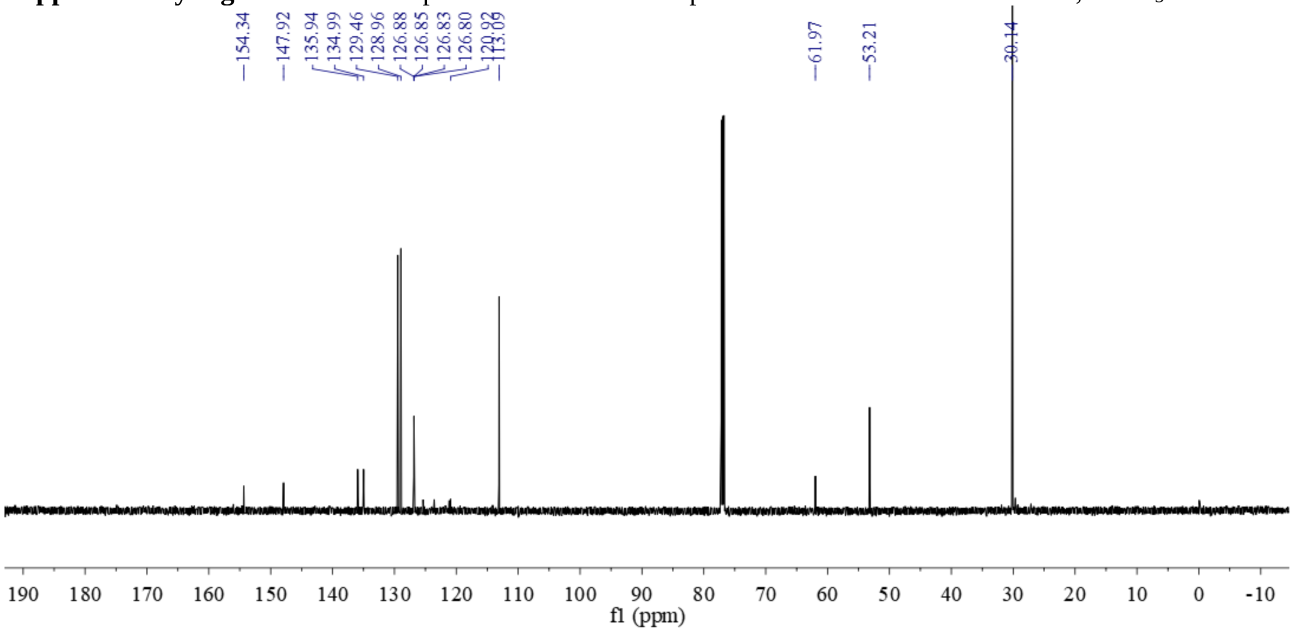

Supplementary Fig. 226. ^{13}C NMR spectrum of **61**. The sample has been recorded in 151 MHz, $CDCl_3$ at 25 °C.

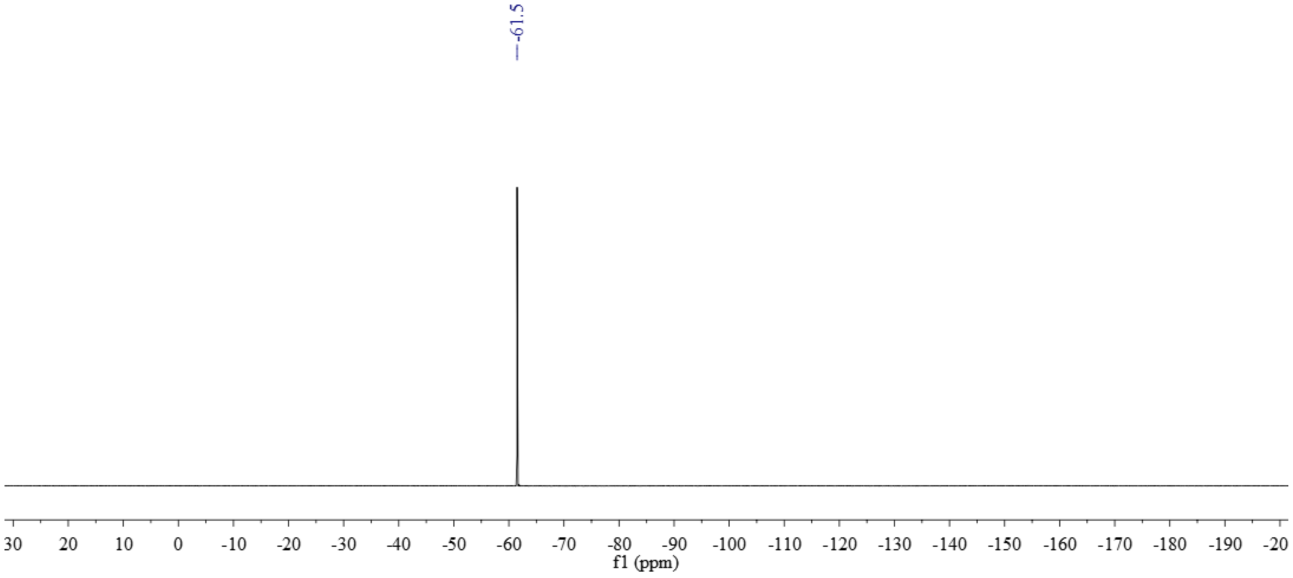

Supplementary Fig. 227. ^{31}F NMR spectrum of **61**. The sample has been recorded in 564 MHz, $CDCl_3$ at 25 °C.

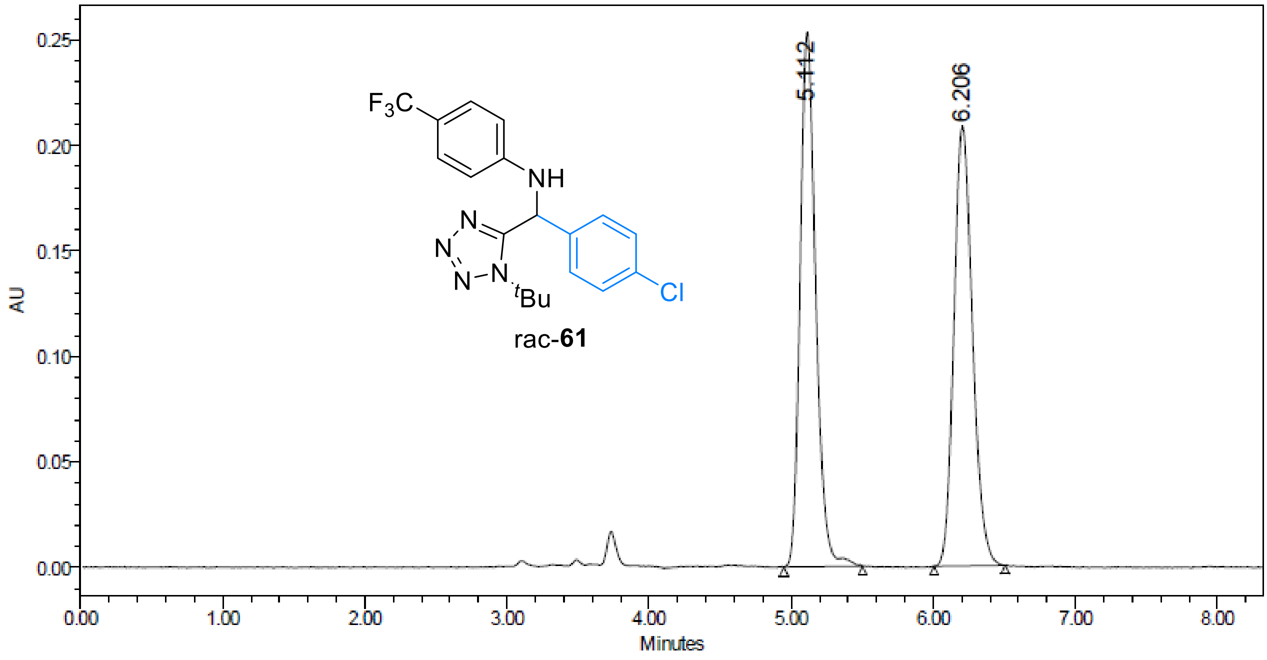

	RT (min)	Area (µV*sec)	% Area	Height (µV)	% Height
1	5.112	1940806	50.53	253470	54.89
2	6.206	1900145	49.47	208320	45.11

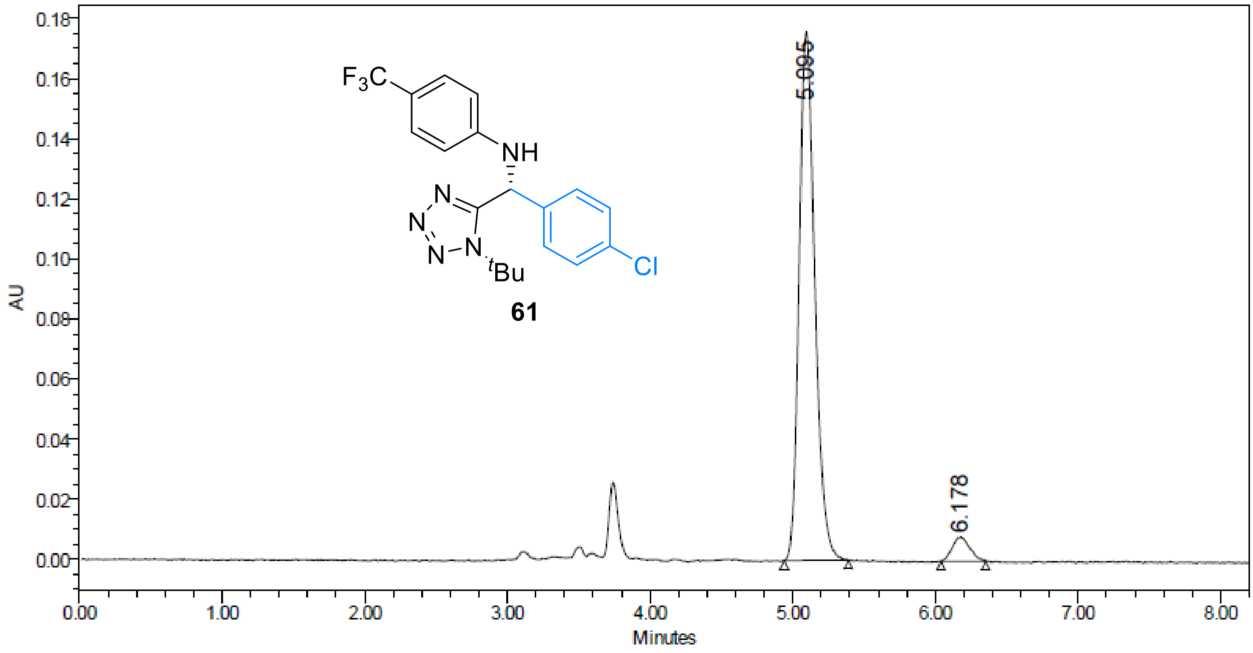

	RT (min)	Area (µV*sec)	% Area	Height (µV)	% Height
1	5.095	1341109	95.04	175884	95.63
2	6.178	69930	4.96	8041	4.37

Supplementary Fig. 228. HPLC of product **61**.

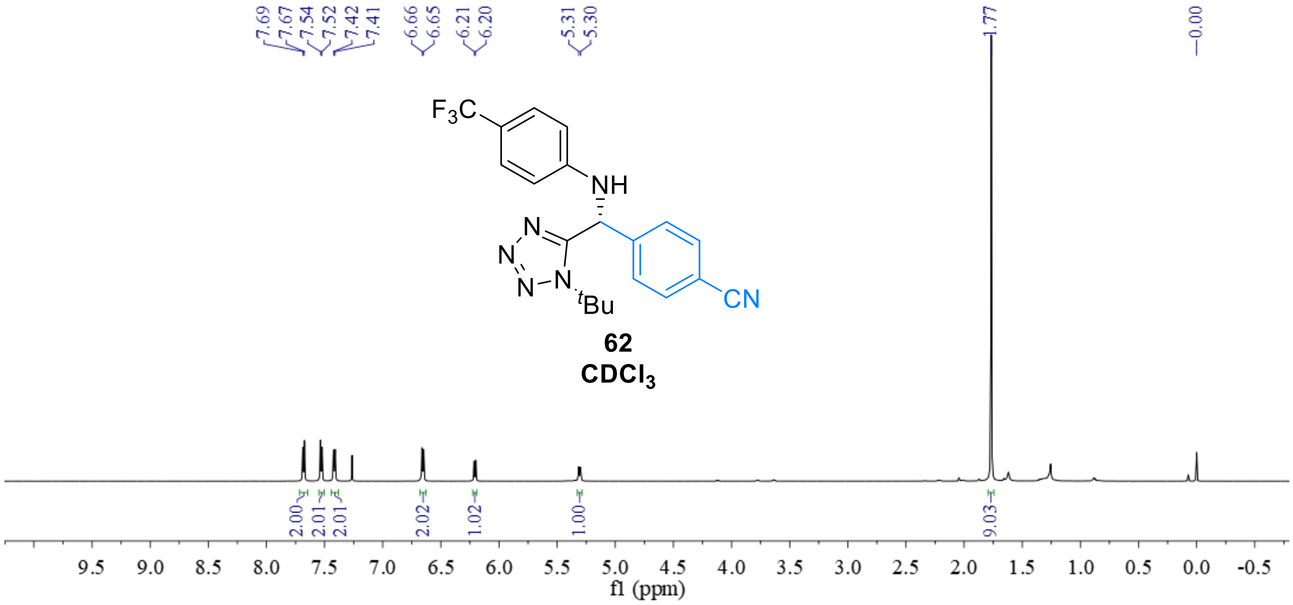

Supplementary Fig. 229. ¹H NMR spectrum of **62**. The sample has been recorded in 600 MHz, $CDCl_3$ at 25 °C.

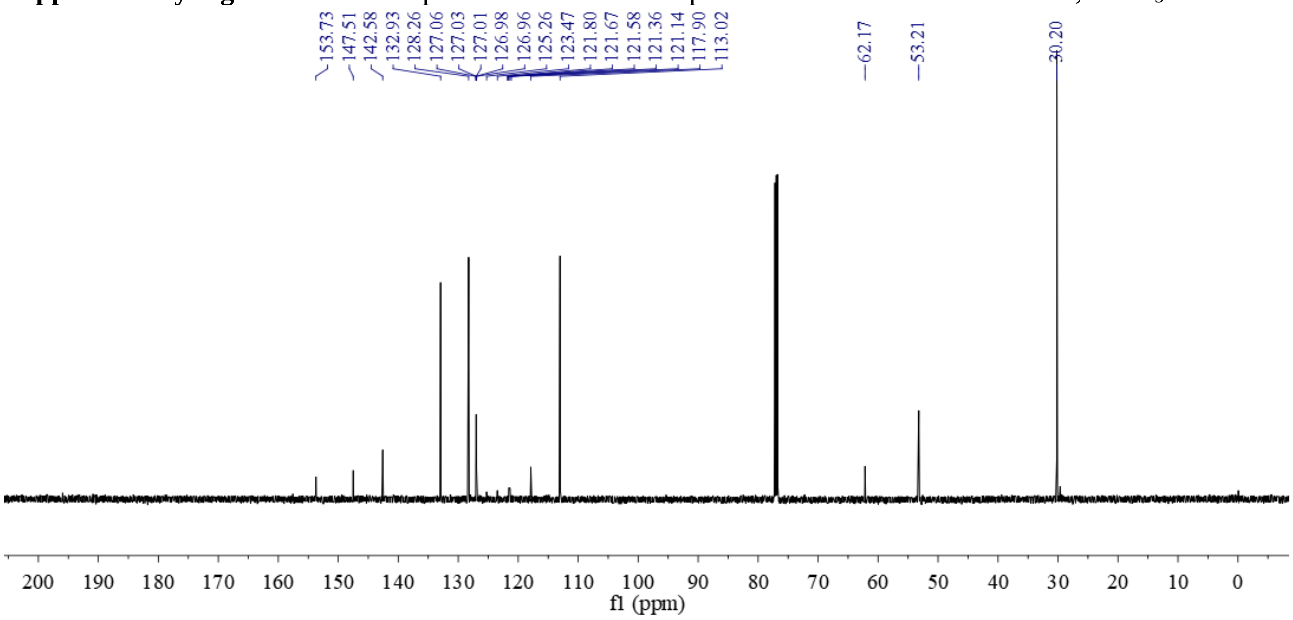

Supplementary Fig. 230. ¹³C NMR spectrum of **62**. The sample has been recorded in 151 MHz, $CDCl_3$ at 25 °C.

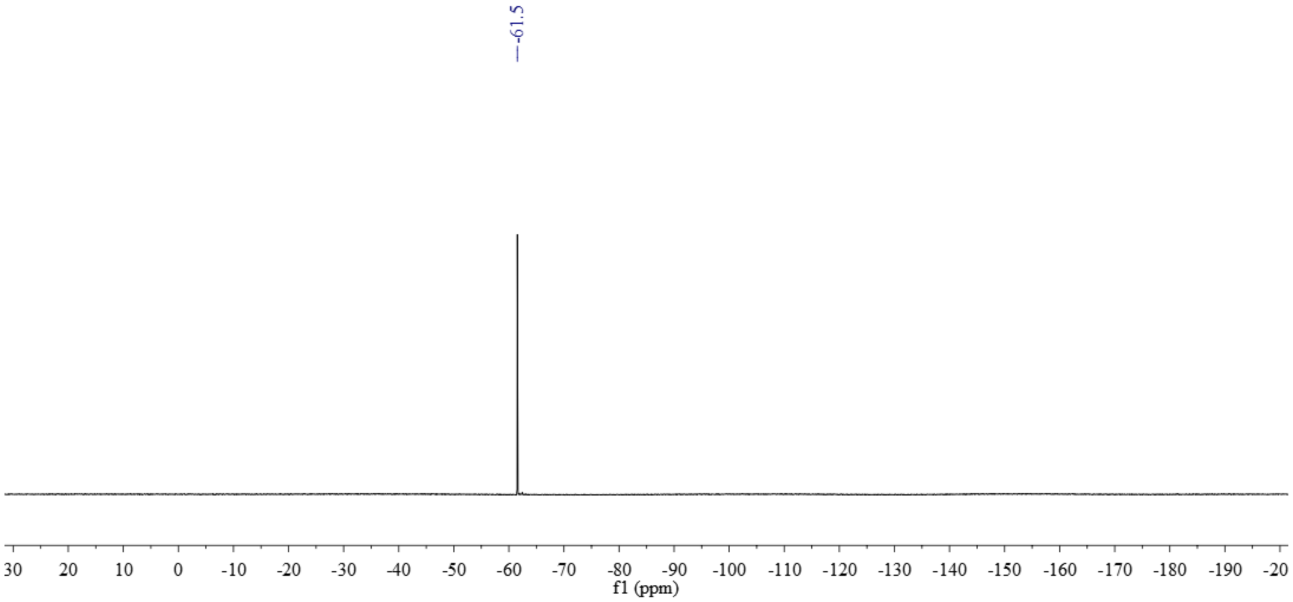

Supplementary Fig. 231. ³¹F NMR spectrum of **62**. The sample has been recorded in 564 MHz, $CDCl_3$ at 25 °C.

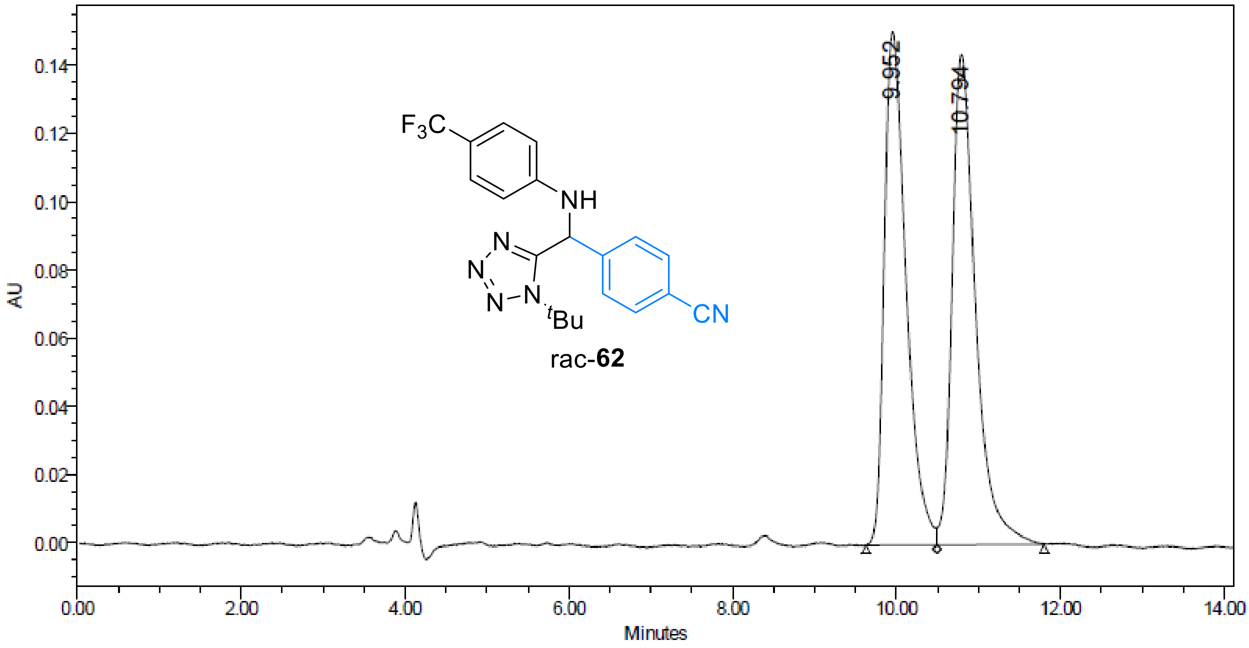

	RT (min)	Area (μV*sec)	% Area	Height (μV)	% Height
1	9.952	2781002	49.34	150639	51.15
2	10.794	2855781	50.66	143840	48.85

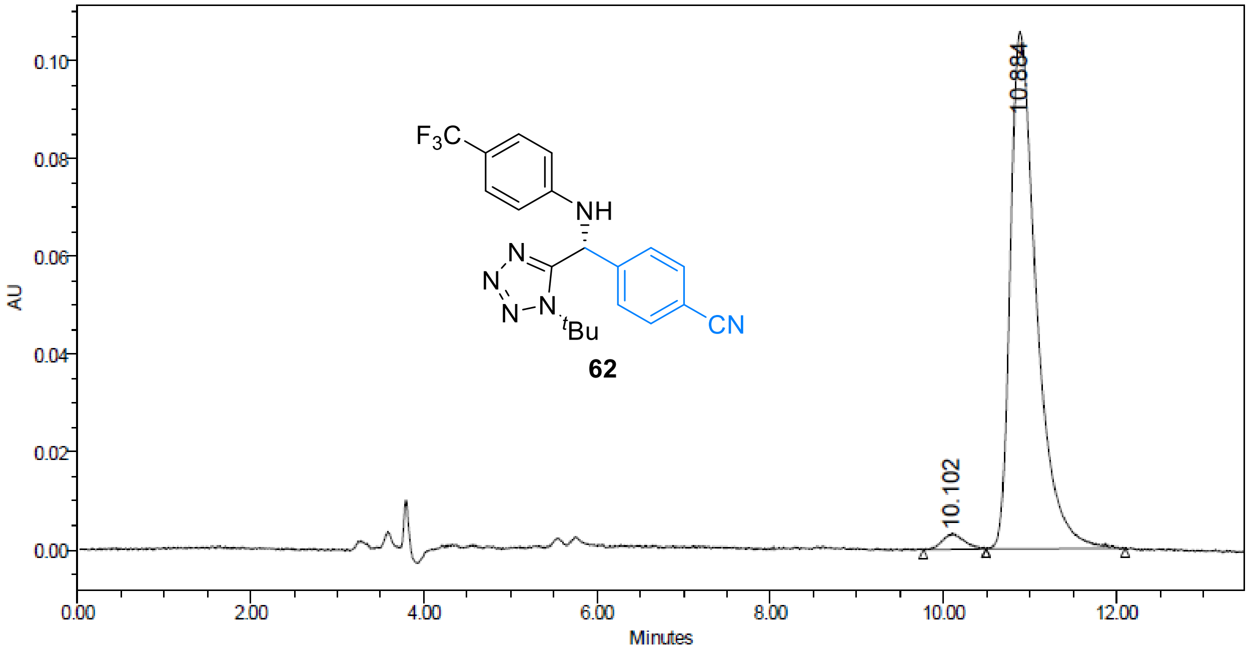

	RT (min)	Area (μV*sec)	% Area	Height (μV)	% Height
1	10.102	55953	2.47	3139	2.88
2	10.884	2208132	97.53	105703	97.12

Supplementary Fig. 232. HPLC of product **62**.

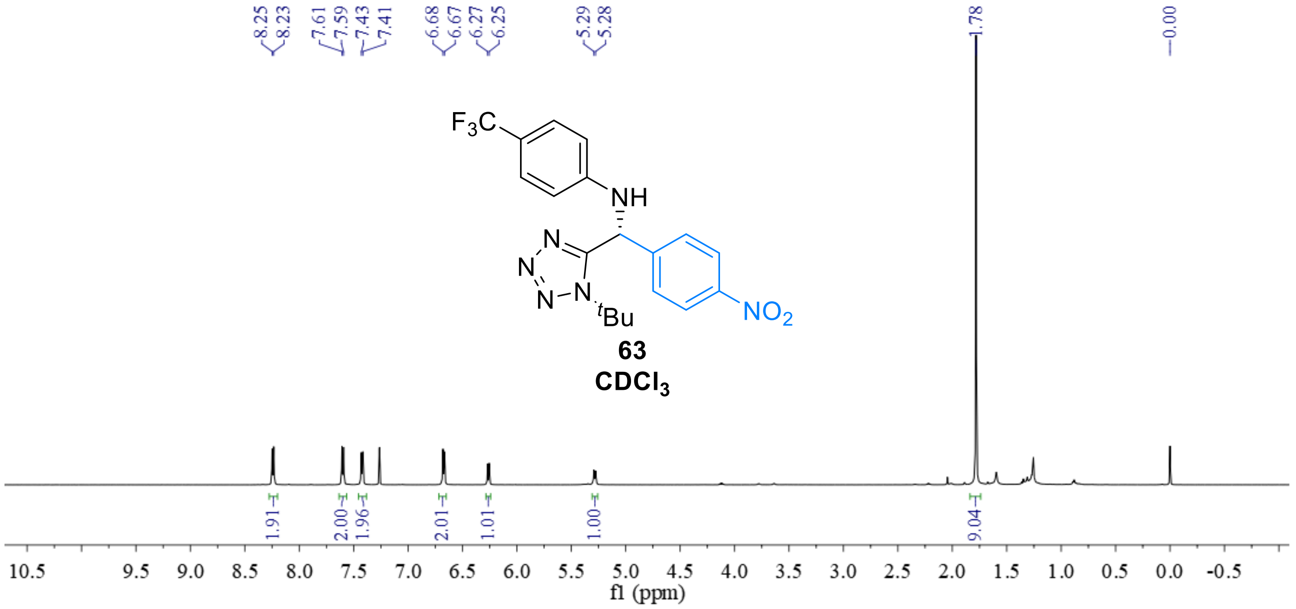

Supplementary Fig. 233. ^{1}H NMR spectrum of **63**. The sample has been recorded in 600 MHz, $CDCl_3$ at 25 °C.

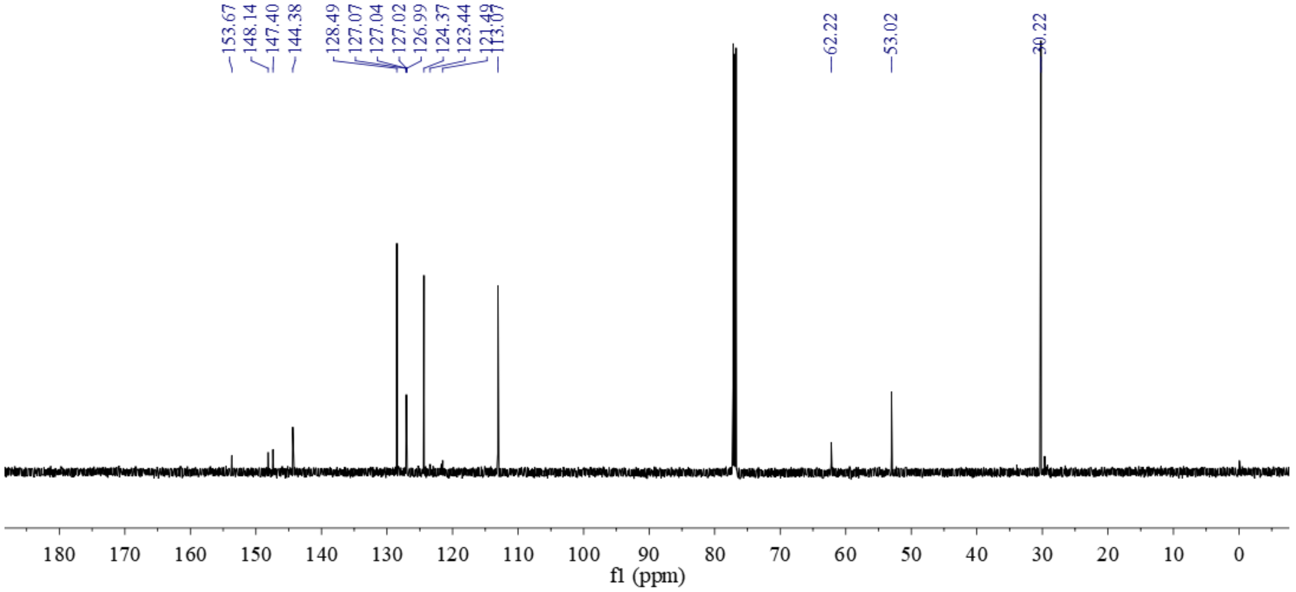

Supplementary Fig. 234. ^{13}C NMR spectrum of **63**. The sample has been recorded in 151 MHz, $CDCl_3$ at 25 °C.

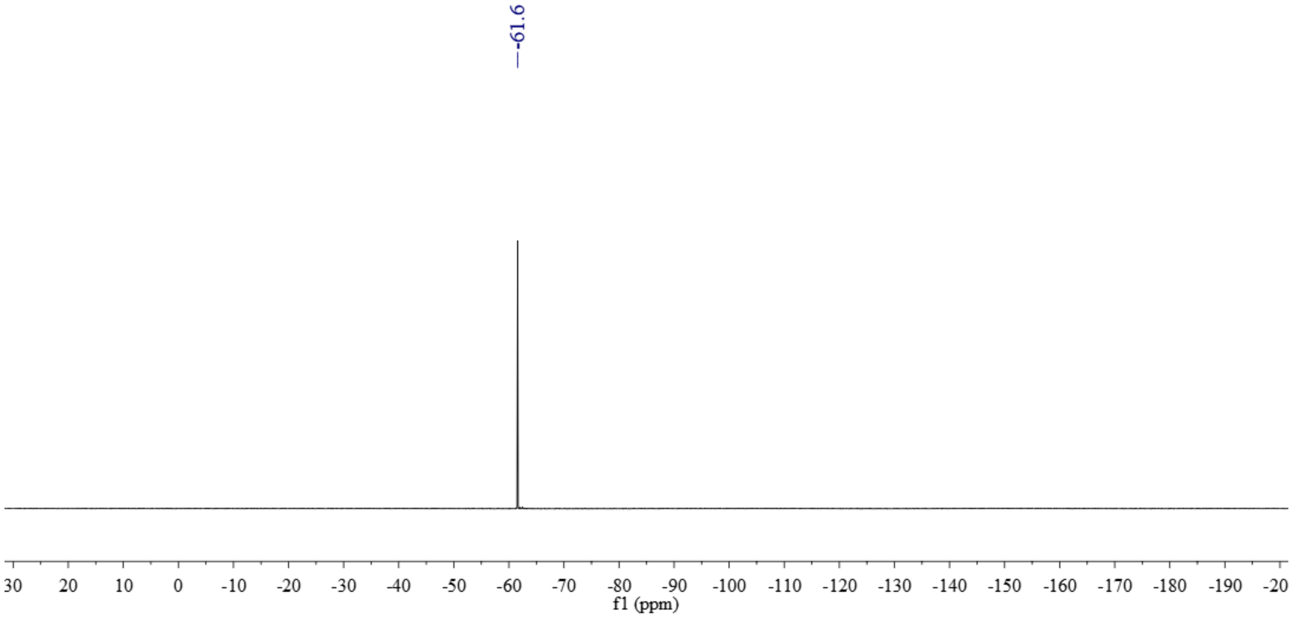

Supplementary Fig. 235. ^{31}F NMR spectrum of **63**. The sample has been recorded in 564 MHz, $CDCl_3$ at 25 °C.

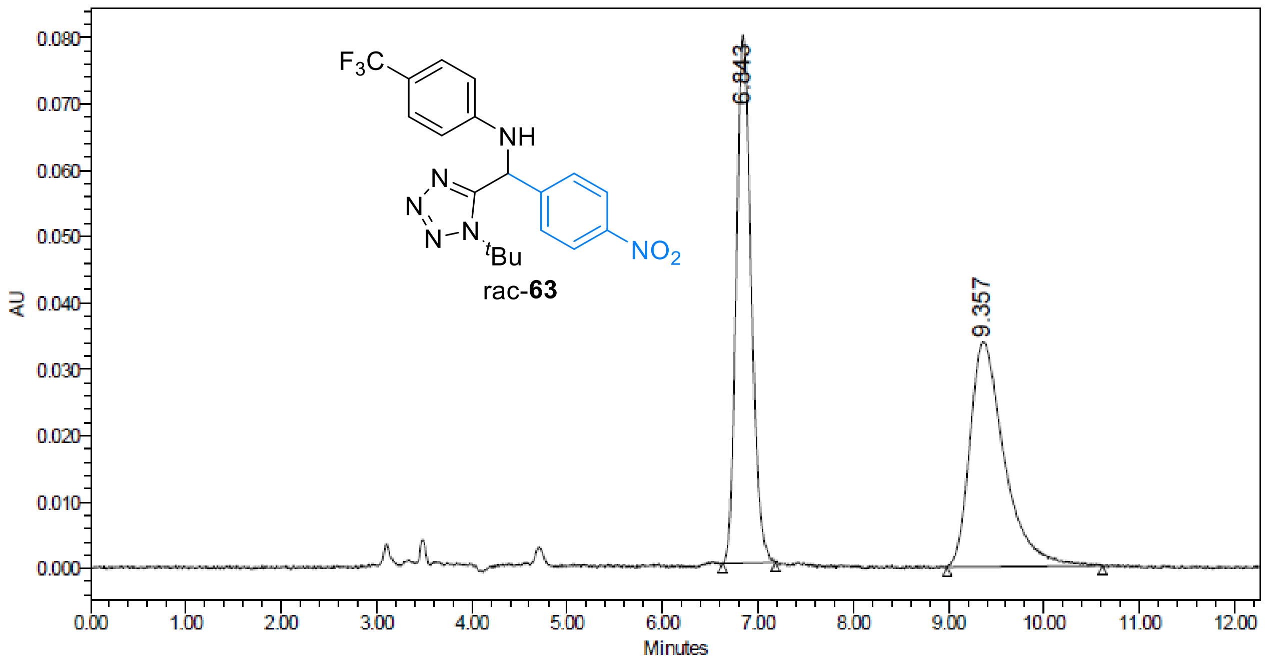

	RT (min)	Area (μV*sec)	% Area	Height (μV)	% Height
1	6.843	863633	50.26	79567	70.08
2	9.357	854678	49.74	33971	29.92

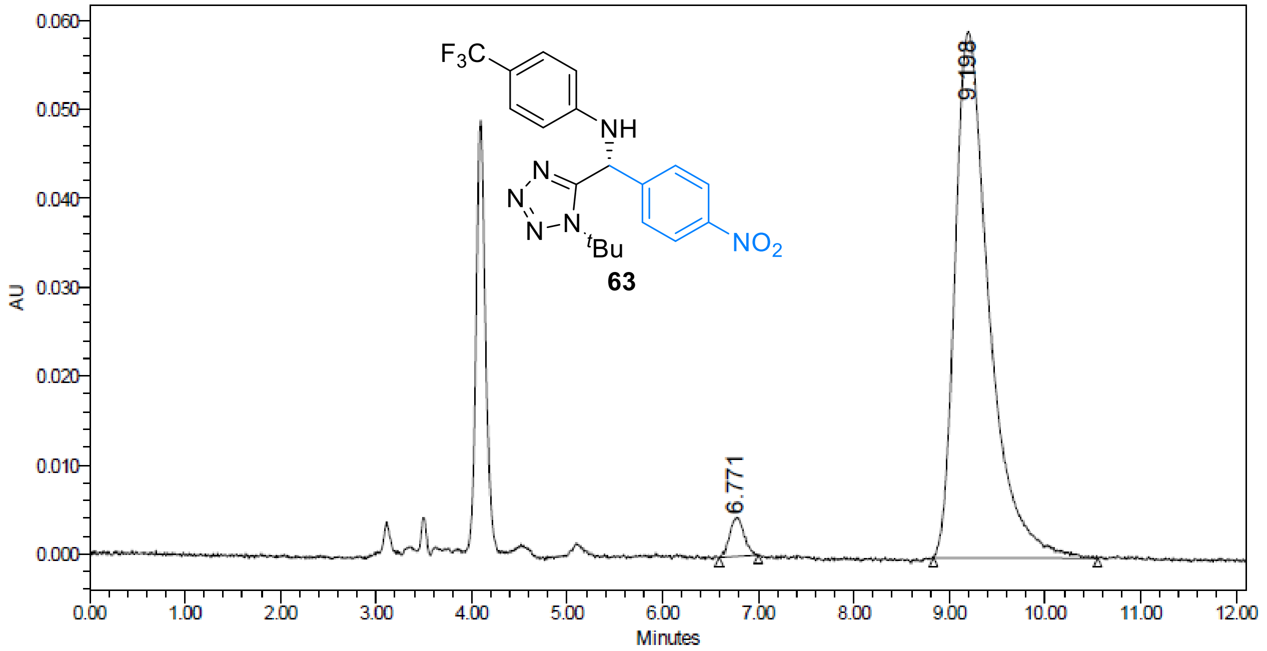

	RT (min)	Area (μV*sec)	% Area	Height (μV)	% Height
1	6.771	45601	3.02	4375	6.88
2	9.198	1464138	96.98	59195	93.12

Supplementary Fig. 236. HPLC of product **63**.

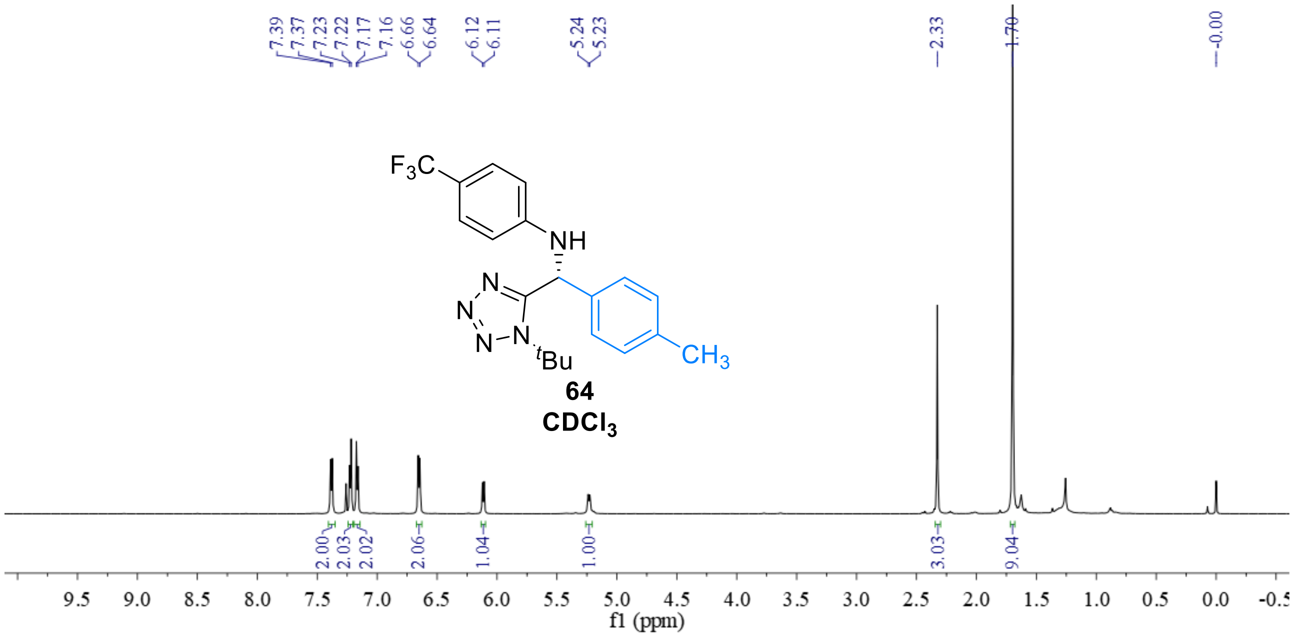

Supplementary Fig. 237. ^{1}H NMR spectrum of **64**. The sample has been recorded in 600 MHz, $CDCl_3$ at 25 °C.

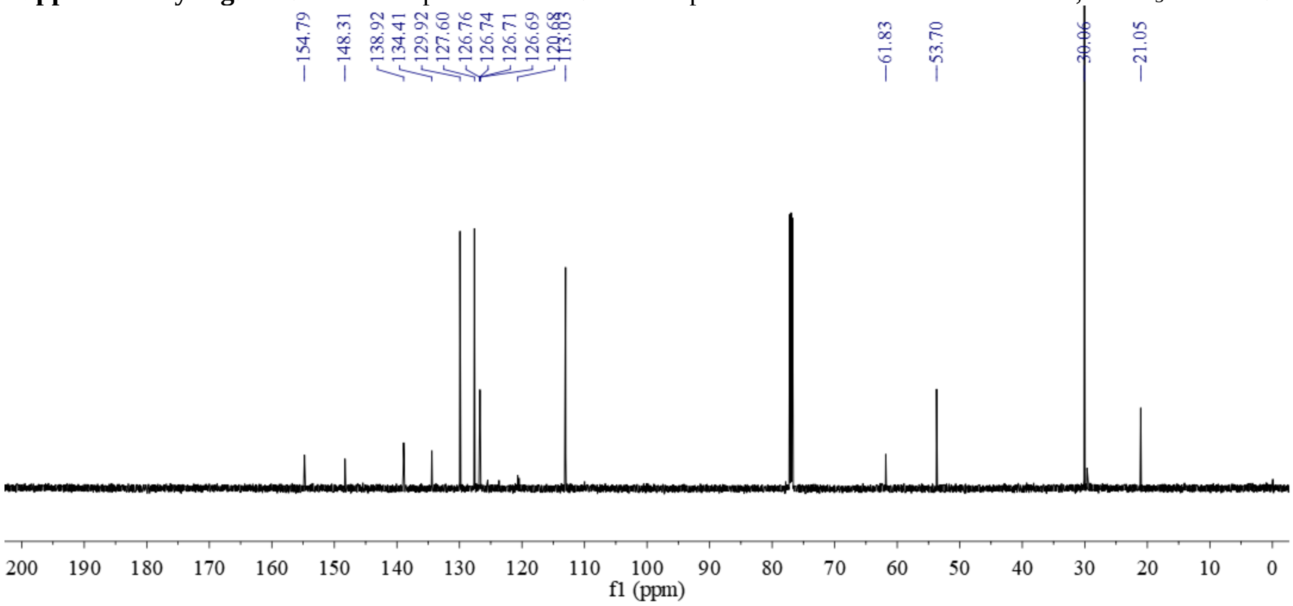

Supplementary Fig. 238. ^{13}C NMR spectrum of **64**. The sample has been recorded in 151 MHz, $CDCl_3$ at 25 °C.

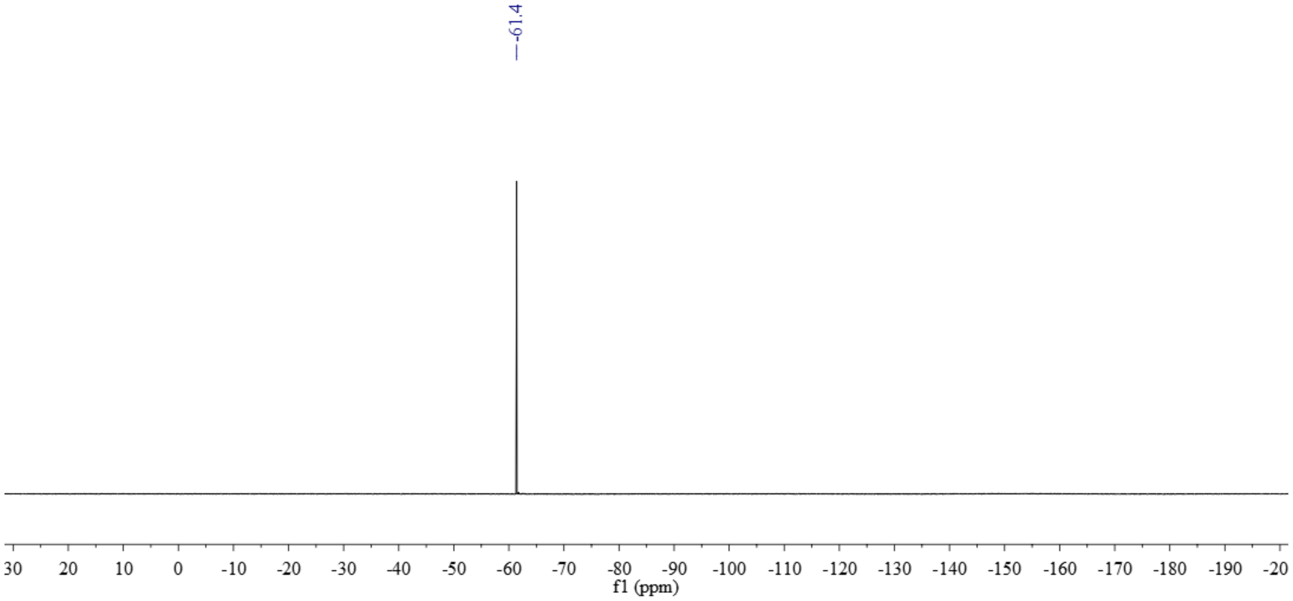

Supplementary Fig. 239. ^{31}F NMR spectrum of **64**. The sample has been recorded in 564 MHz, $CDCl_3$ at 25 °C.

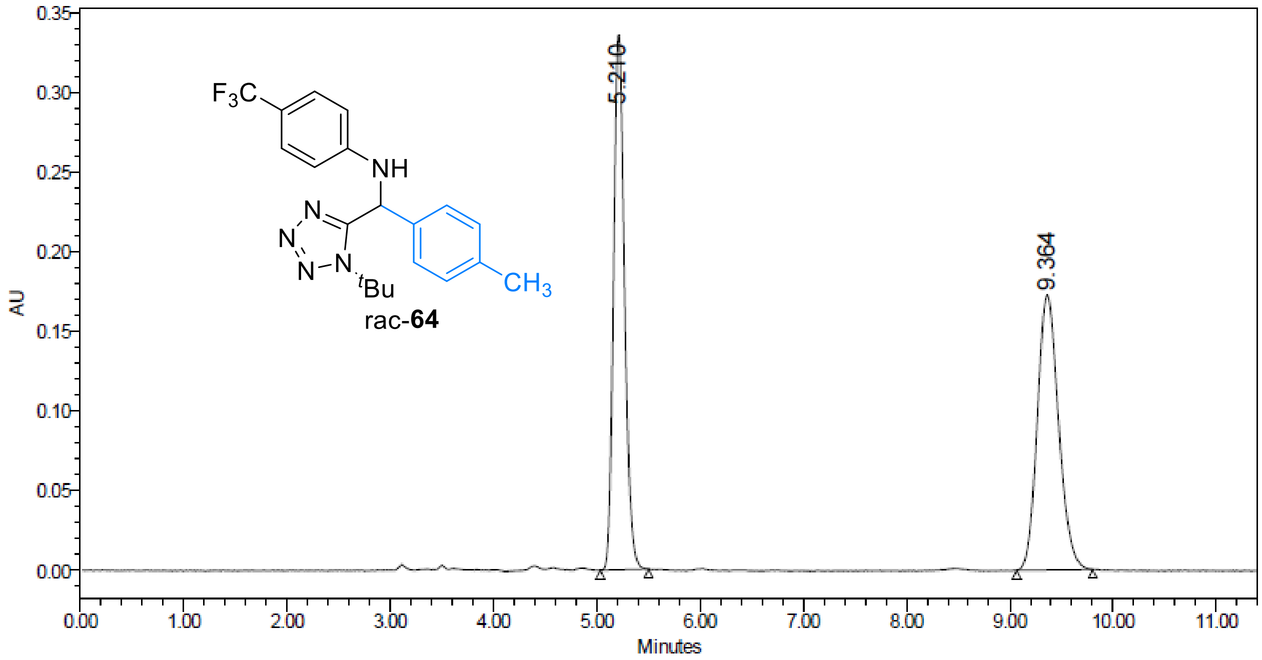

	RT (min)	Area (µV*sec)	% Area	Height (µV)	% Height
1	5.210	2475944	50.07	335540	66.04
2	9.364	2468763	49.93	172569	33.96

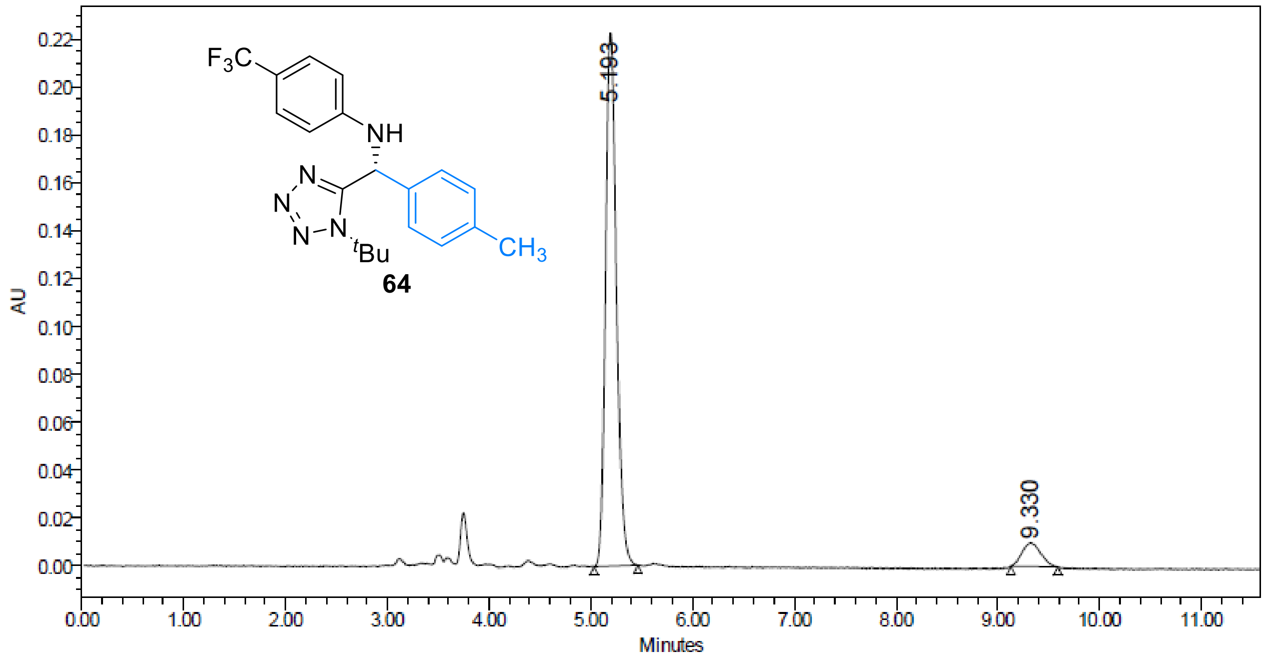

	RT (min)	Area (µV*sec)	% Area	Height (µV)	% Height
1	5.193	1655149	92.94	222794	95.76
2	9.330	125786	7.06	9866	4.24

Supplementary Fig. 240. HPLC of product **64**.

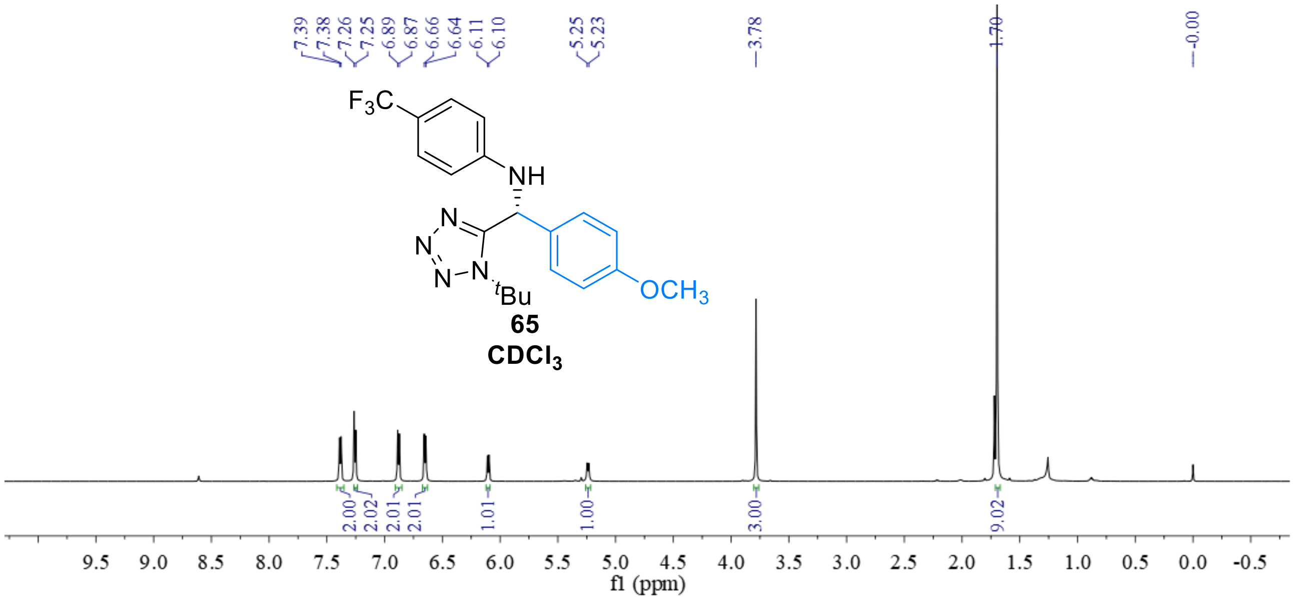

Supplementary Fig. 241. ^{1}H NMR spectrum of **65**. The sample has been recorded in 600 MHz, $CDCl_3$ at 25 °C.

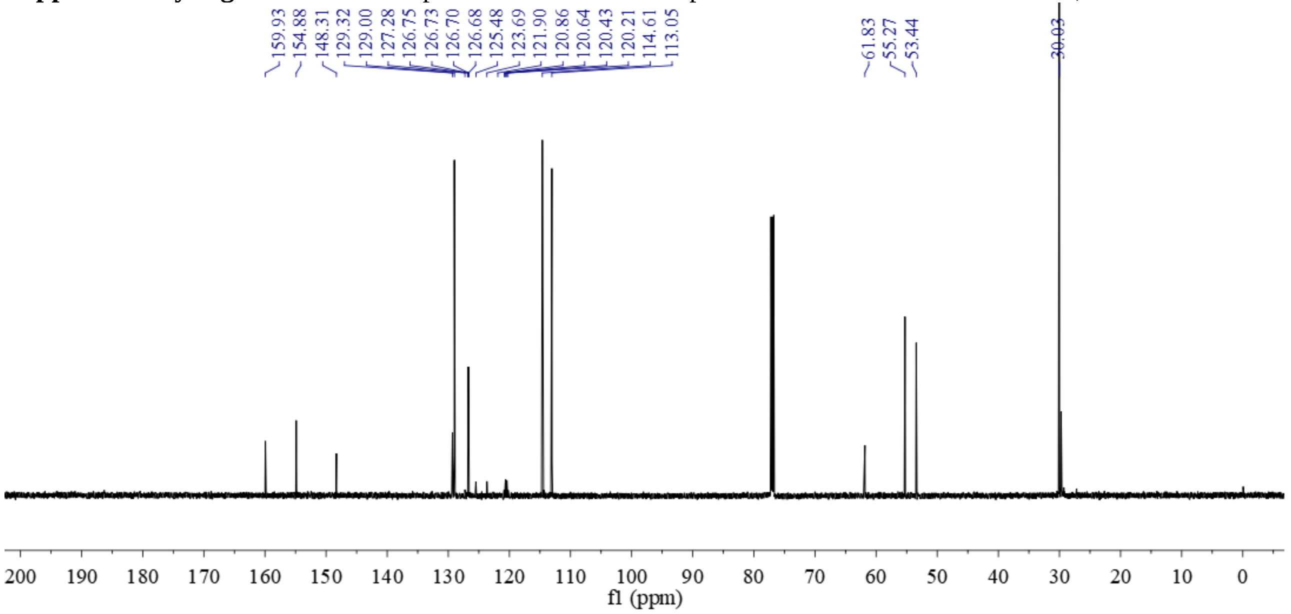

Supplementary Fig. 242. ^{13}C NMR spectrum of **65**. The sample has been recorded in 151 MHz, $CDCl_3$ at 25 °C.

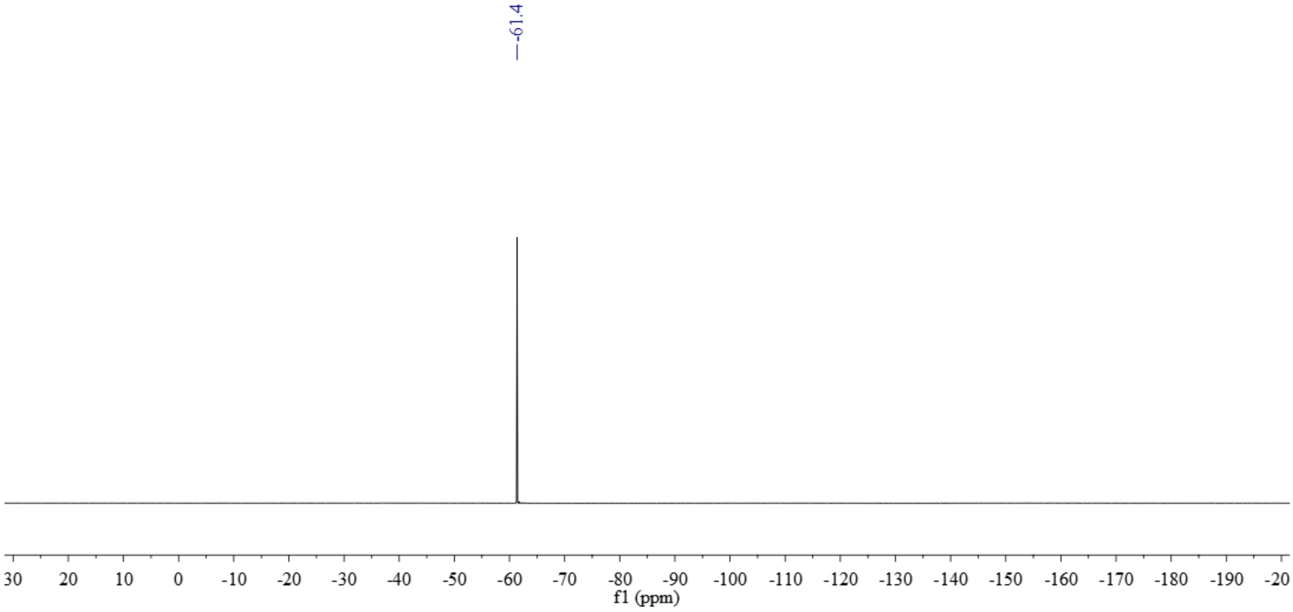

Supplementary Fig. 243. ^{31}F NMR spectrum of **65**. The sample has been recorded in 564 MHz, $CDCl_3$ at 25 °C.

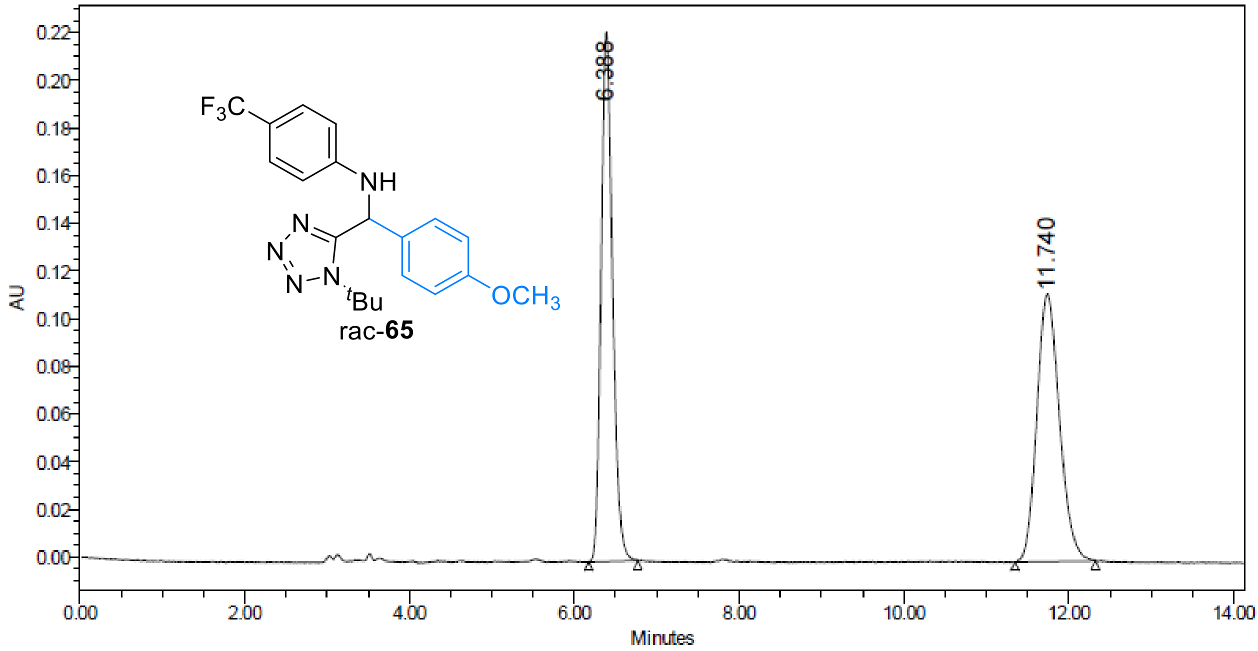

	RT (min)	Area (µV*sec)	% Area	Height (µV)	% Height
1	6.388	2155229	50.16	221961	66.43
2	11.740	2141751	49.84	112176	33.57

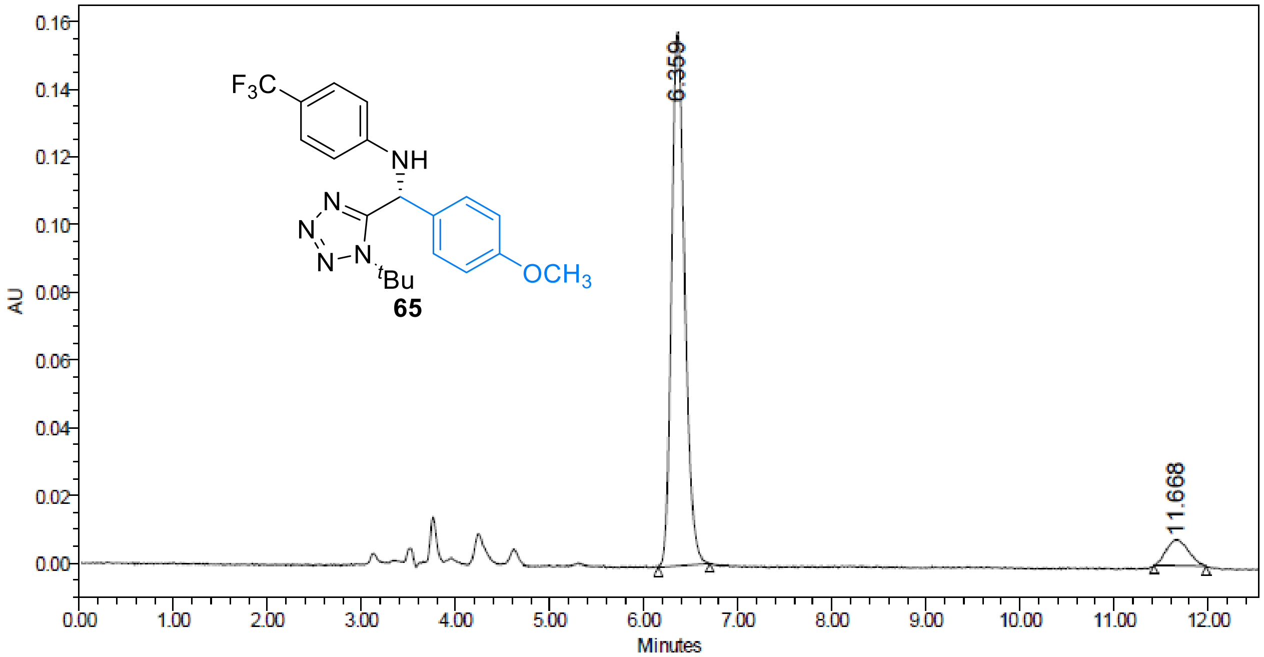

	RT (min)	Area (µV*sec)	% Area	Height (µV)	% Height
1	6.359	1531220	92.49	157691	95.35
2	11.668	124376	7.51	7694	4.65

Supplementary Fig. 244. HPLC of product **65**.

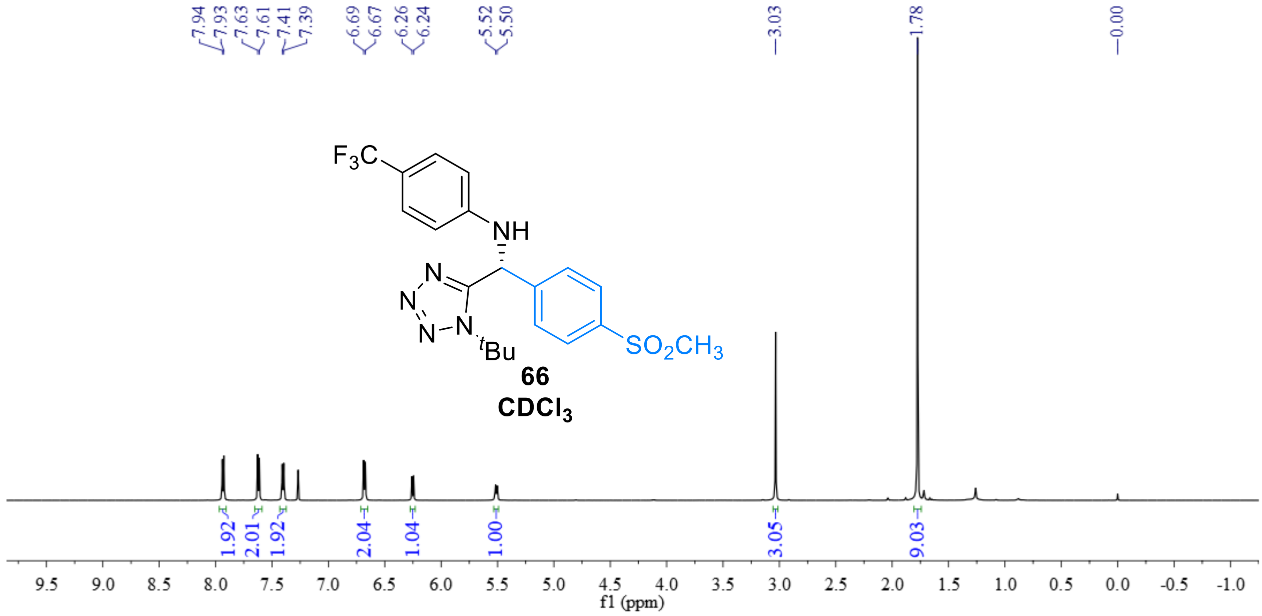

Supplementary Fig. 245. ^{1}H NMR spectrum of **66**. The sample has been recorded in 600 MHz, $CDCl_3$ at 25 °C.

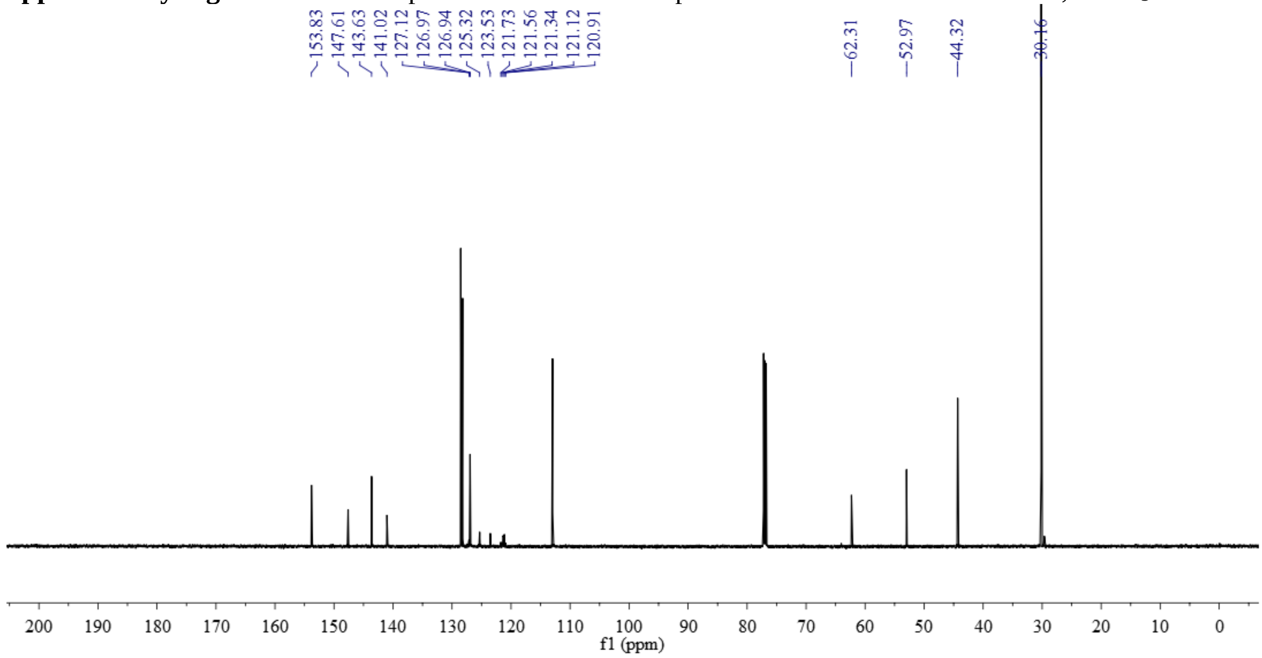

Supplementary Fig. 246. ^{13}C NMR spectrum of **66**. The sample has been recorded in 151 MHz, $CDCl_3$ at 25 °C.

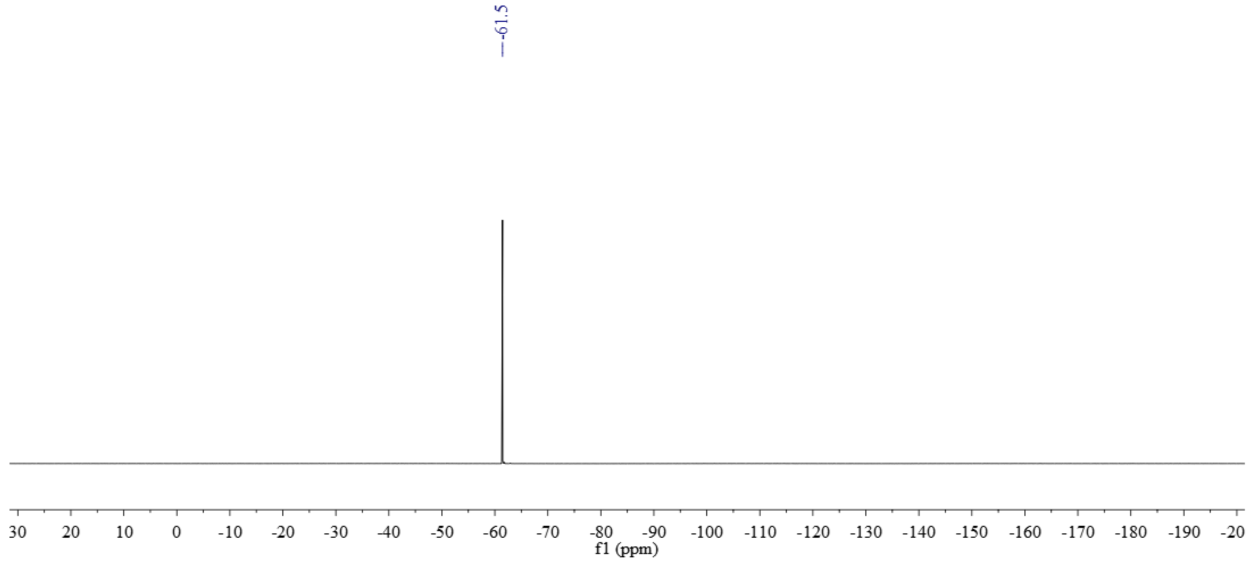

Supplementary Fig. 247. [31]F NMR spectrum of **66**. The sample has been recorded in 564 MHz, $CDCl_3$ at 25 °C.

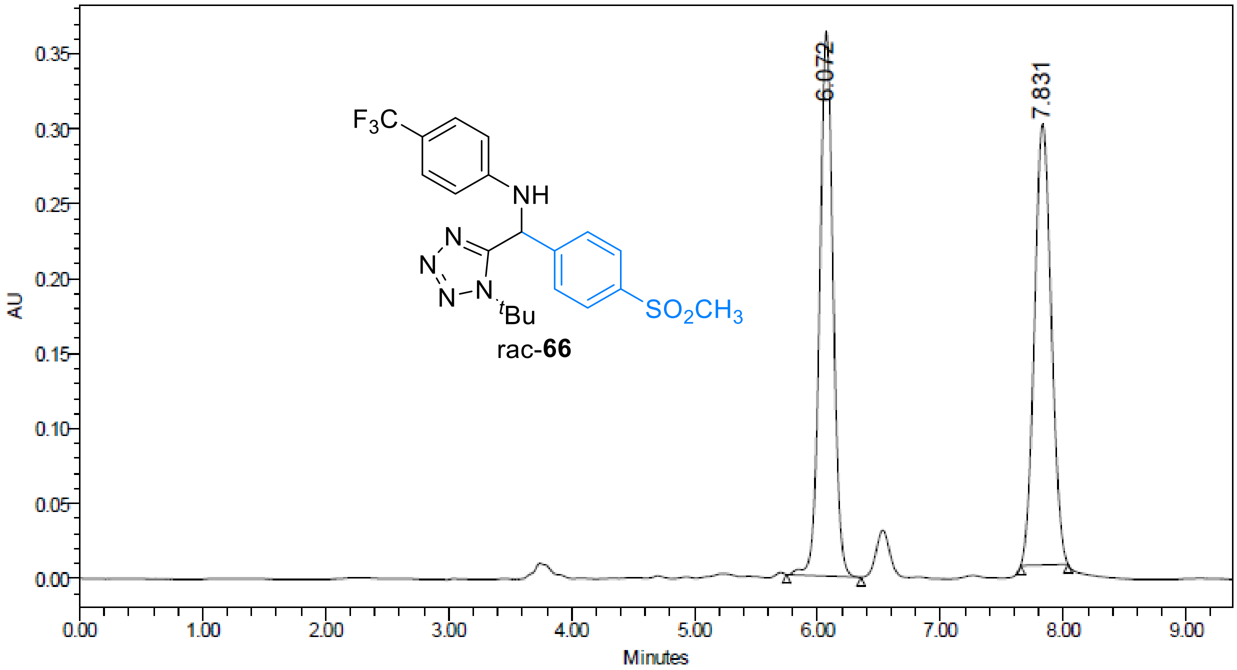

	RT (min)	Peak Type	Area (µV*sec)	% Area	Height (µV)	% Height	Integration Type	Points Across Peak	Start Time (min)	End Time (min)
1	6.072	Unknown	2762750	49.73	363265	55.24	bb	364	5.747	6.353
2	7.831	Unknown	2792461	50.27	294383	44.76	bb	231	7.653	8.038

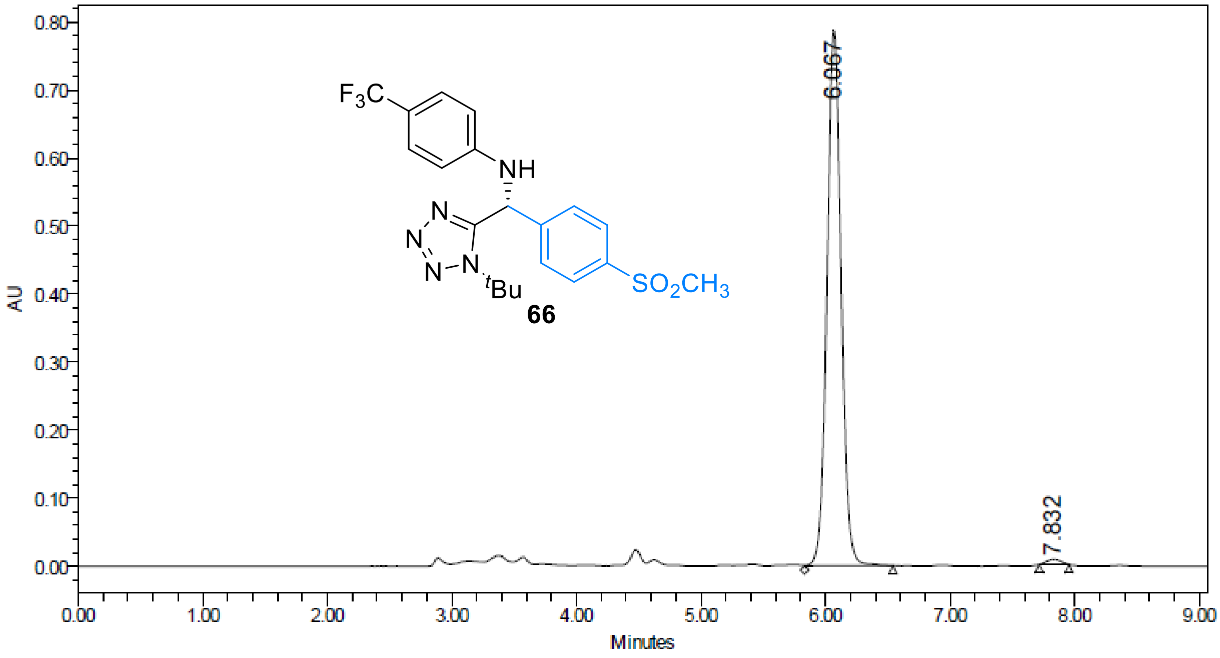

	RT (min)	Peak Type	Area (µV*sec)	% Area	Height (µV)	% Height	Integration Type	Points Across Peak	Start Time (min)	End Time (min)
1	6.067	Unknown	6181429	99.02	785717	99.02	VB	426	5.832	6.542
2	7.832	Unknown	61238	0.98	7812	0.98	bb	143	7.715	7.953

Supplementary Fig. 248. HPLC of product **66**.

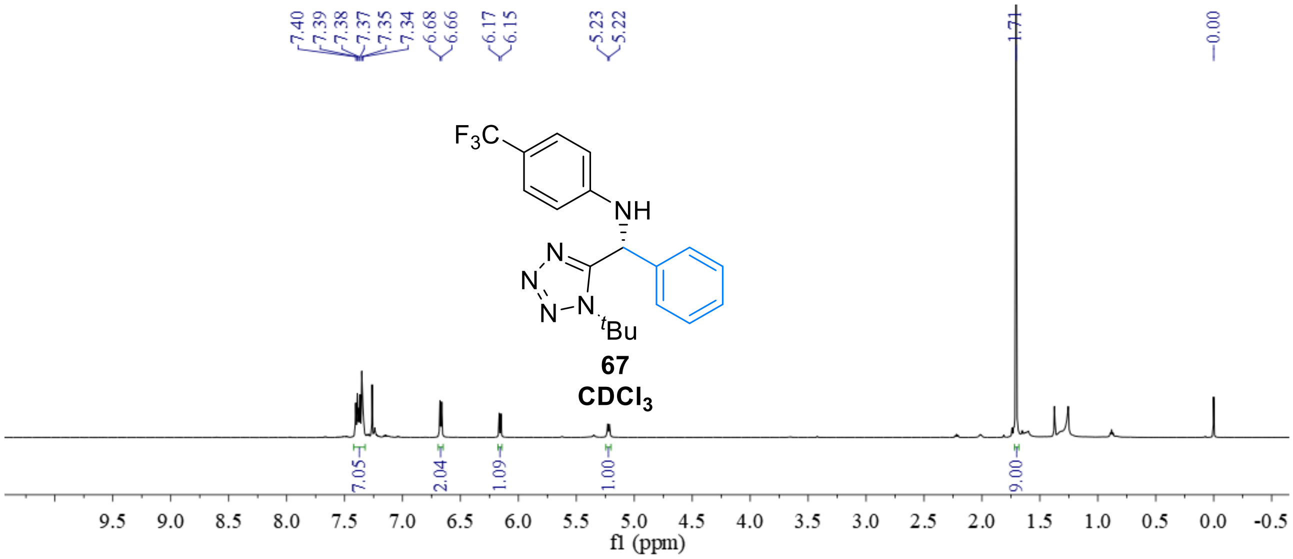

Supplementary Fig. 249. ^{1}H NMR spectrum of **67**. The sample has been recorded in 600 MHz, $CDCl_3$ at 25 °C.

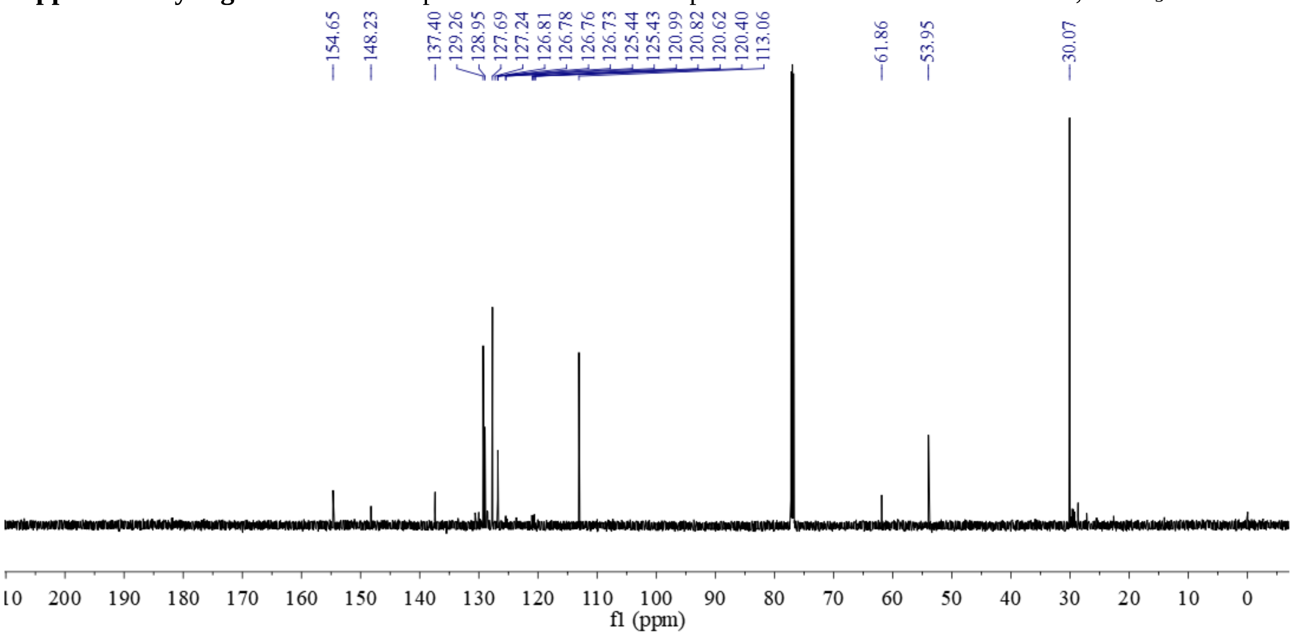

Supplementary Fig. 250. ^{13}C NMR spectrum of **67**. The sample has been recorded in 151 MHz, $CDCl_3$ at 25 °C.

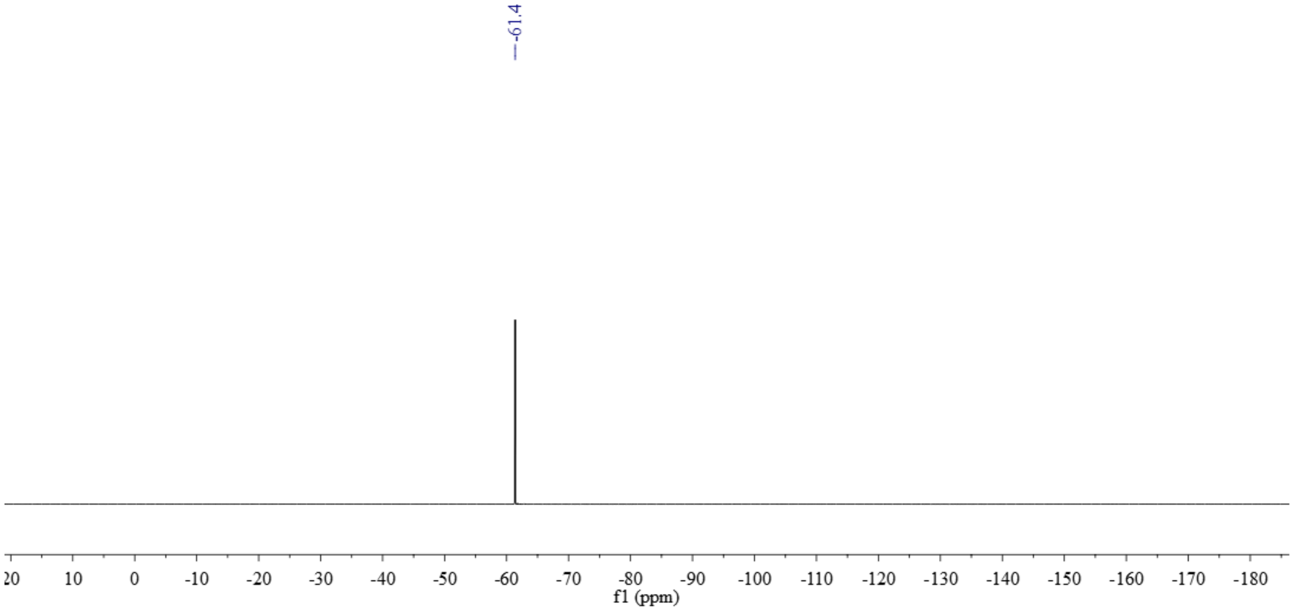

Supplementary Fig. 251. ^{31}F NMR spectrum of **67**. The sample has been recorded in 564 MHz, $CDCl_3$ at 25 °C.

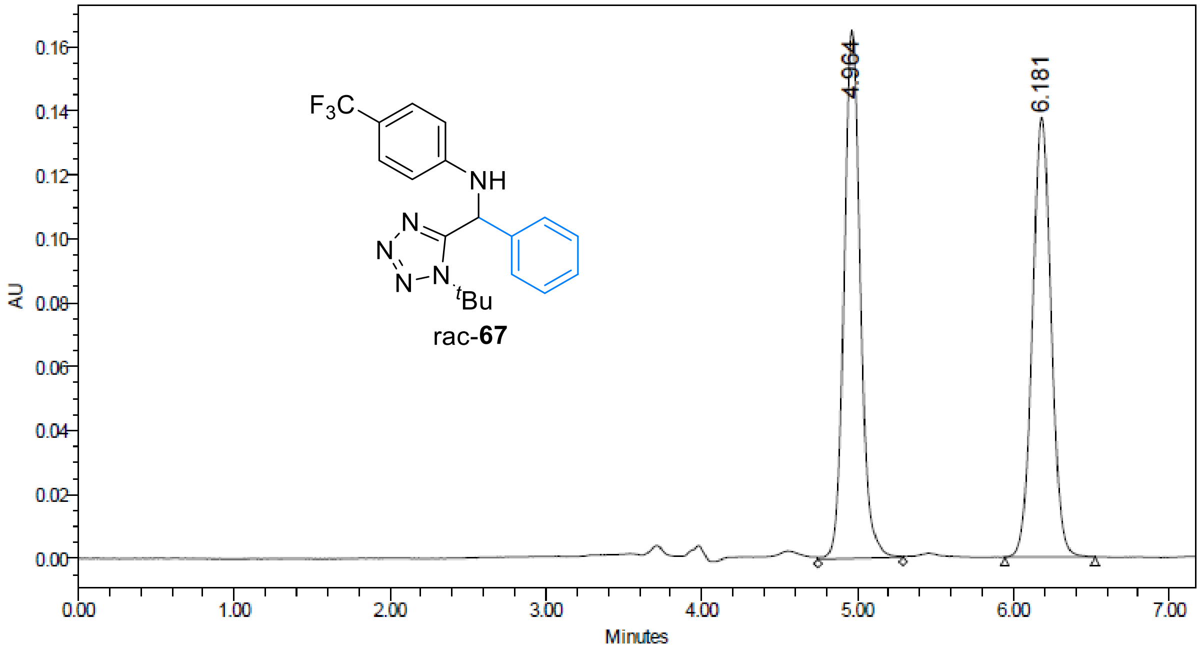

	RT (min)	Peak Type	Area (µV*sec)	% Area	Height (µV)	% Height	Integration Type	Points Across Peak	Start Time (min)	End Time (min)
1	4.964	Unknown	1199932	51.09	165086	54.56	VV	328	4.745	5.292
2	6.181	Unknown	1148954	48.91	137508	45.44	BB	347	5.945	6.523

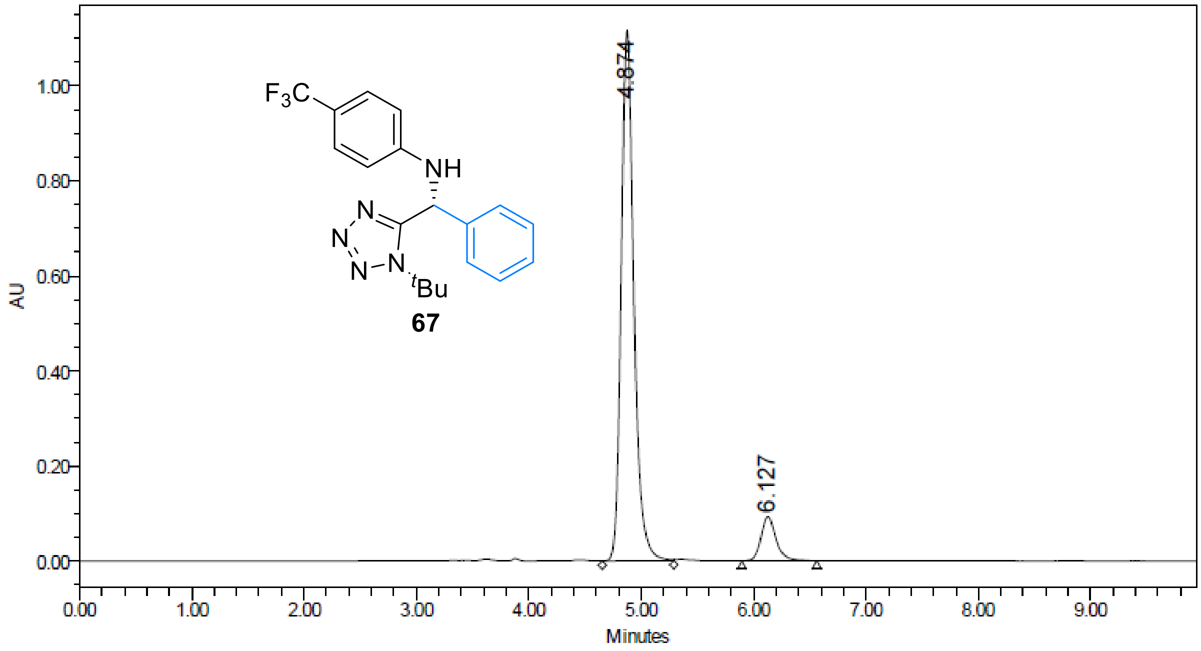

	RT (min)	Peak Type	Area (µV*sec)	% Area	Height (µV)	% Height	Integration Type	Points Across Peak	Start Time (min)	End Time (min)
1	4.874	Unknown	8725252	91.09	1115361	92.35	VV	382	4.653	5.290
2	6.127	Unknown	853204	8.91	92362	7.65	BB	399	5.897	6.562

Supplementary Fig. 252. HPLC of product **67**.

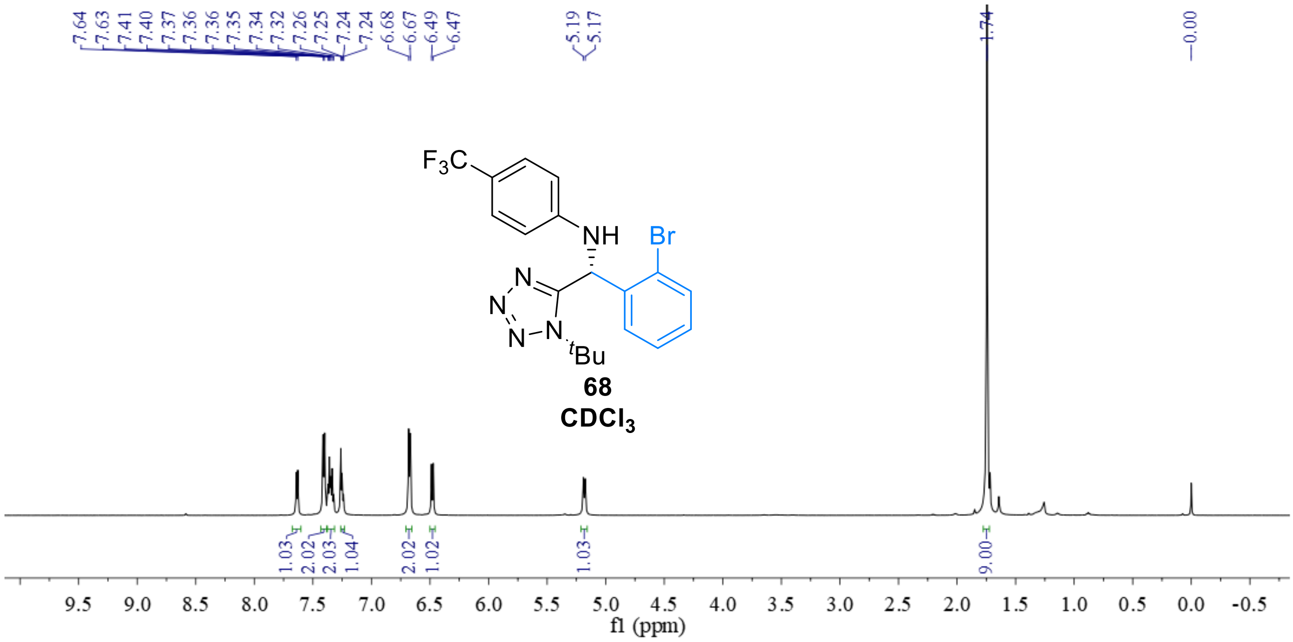

Supplementary Fig. 253. ^{1}H NMR spectrum of **68**. The sample has been recorded in 600 MHz, $CDCl_3$ at 25 °C.

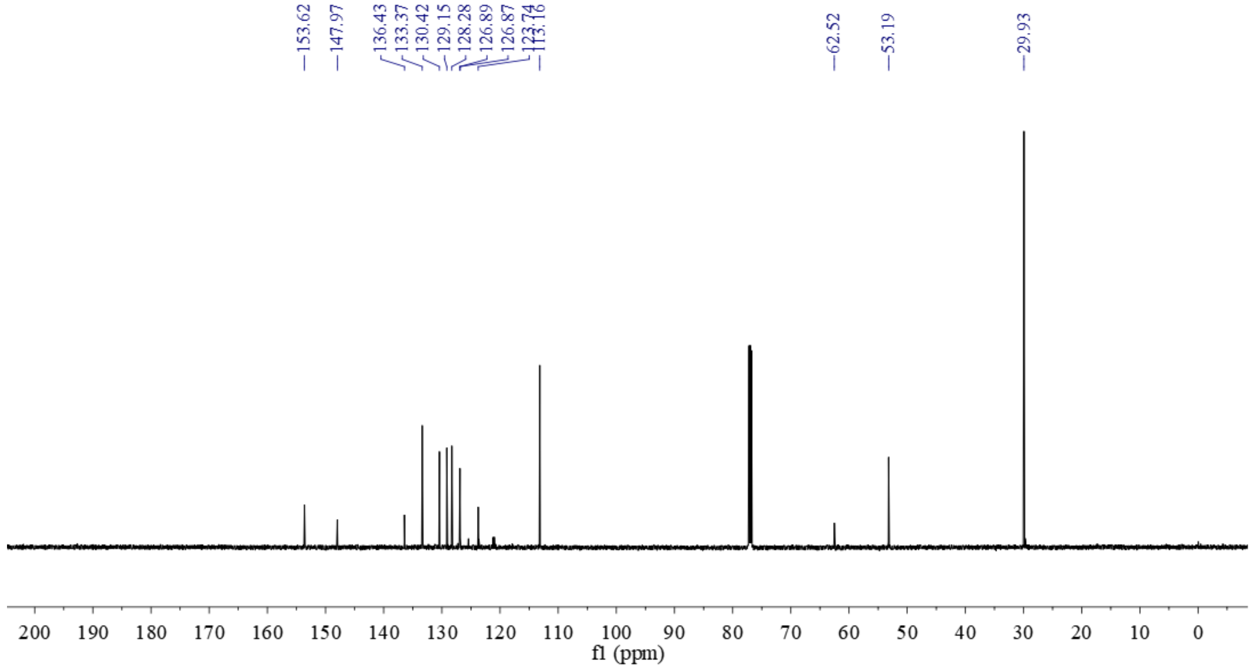

Supplementary Fig. 254. ^{13}C NMR spectrum of **68**. The sample has been recorded in 151 MHz, $CDCl_3$ at 25 °C.

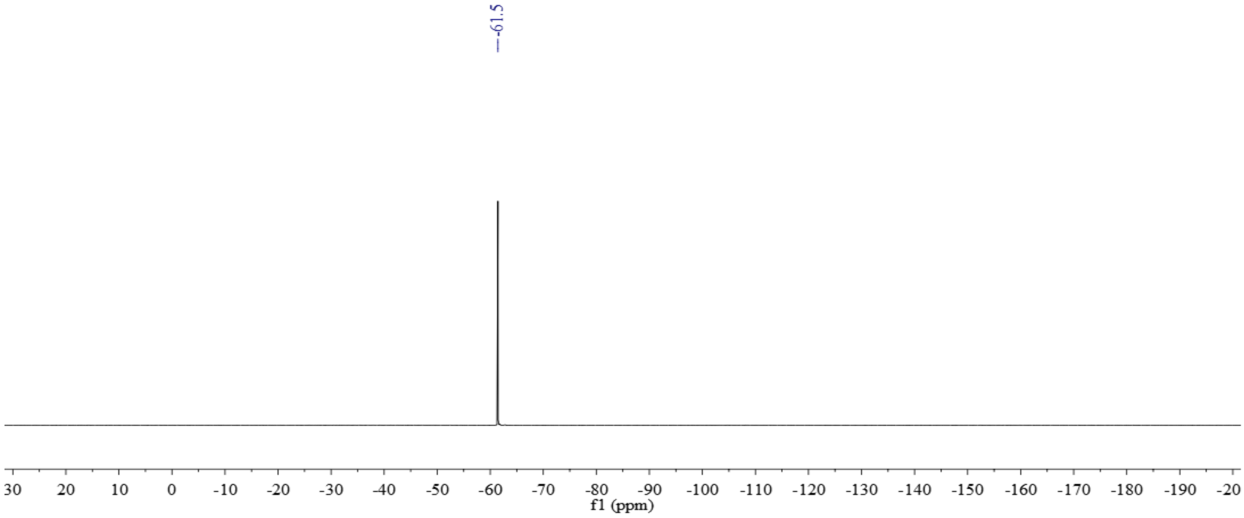

Supplementary Fig. 255. ^{31}F NMR spectrum of **68**. The sample has been recorded in 564 MHz, $CDCl_3$ at 25 °C.

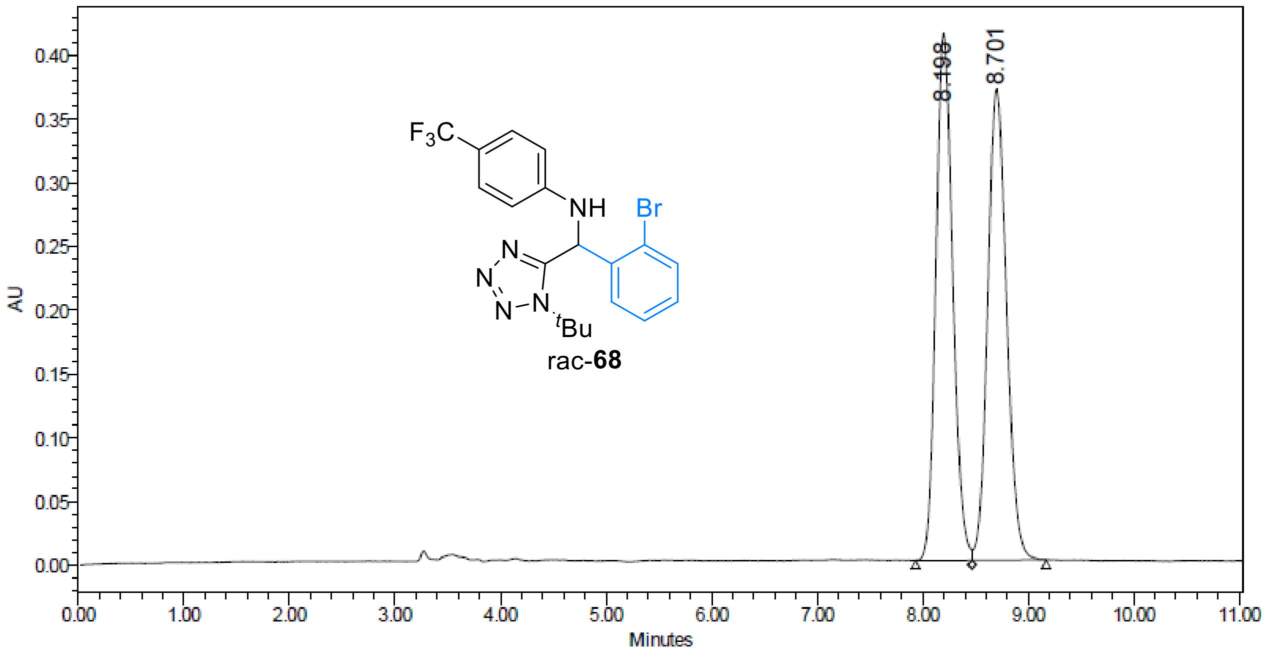

	RT (min)	Area (µV*sec)	% Area	Height (µV)	% Height
1	8.198	4578567	49.98	413849	52.81
2	8.701	4582441	50.02	369846	47.19

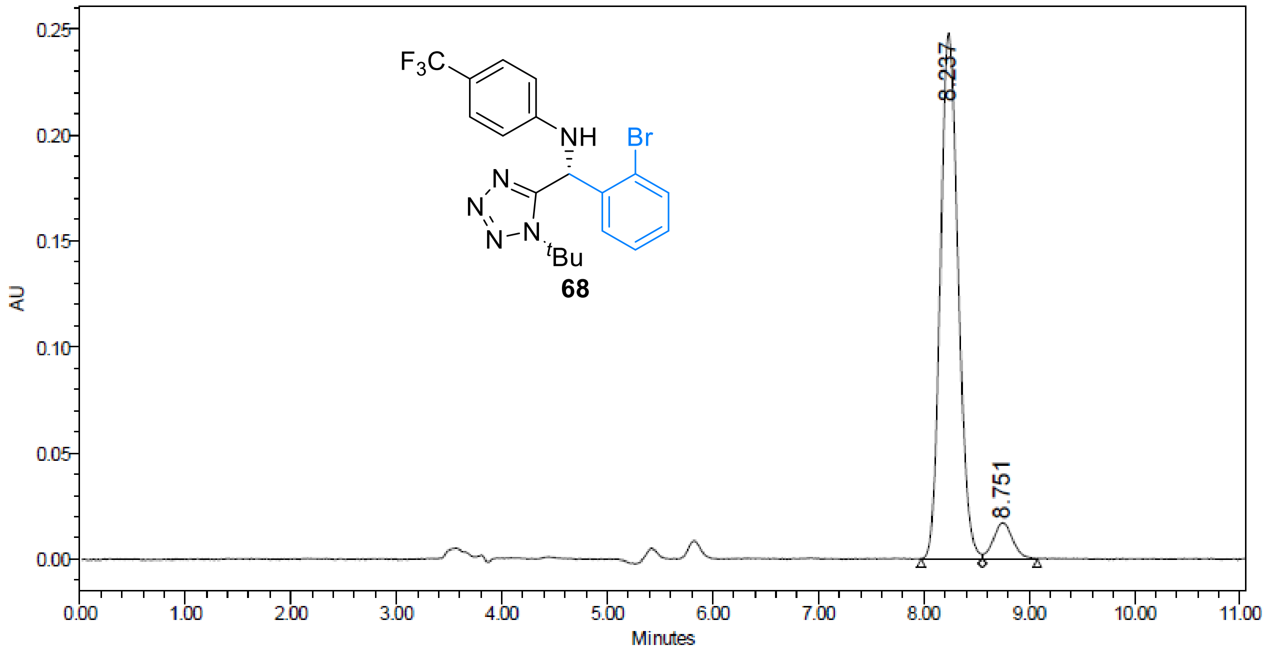

	RT (min)	Area (µV*sec)	% Area	Height (µV)	% Height
1	8.237	2907265	93.01	247961	93.57
2	8.751	218325	6.99	17034	6.43

Supplementary Fig. 256. HPLC of product **68**.

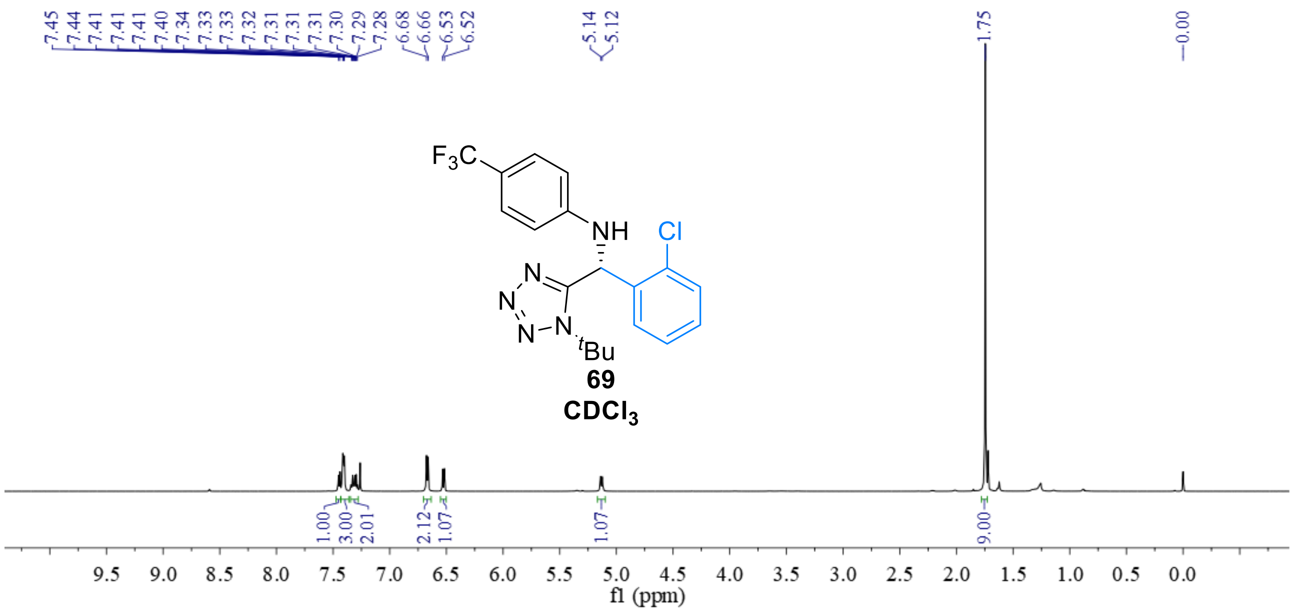

Supplementary Fig. 257. ^{1}H NMR spectrum of **69**. The sample has been recorded in 600 MHz, $CDCl_3$ at 25 °C.

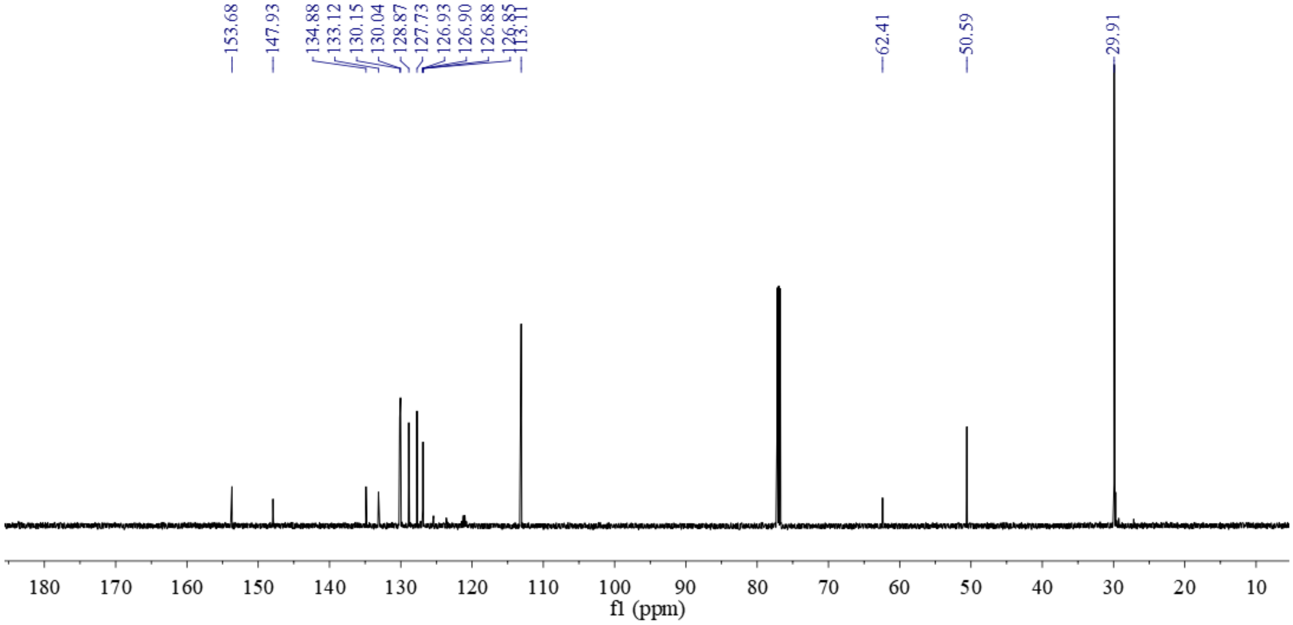

Supplementary Fig. 258. ^{13}C NMR spectrum of **69**. The sample has been recorded in 151 MHz, $CDCl_3$ at 25 °C.

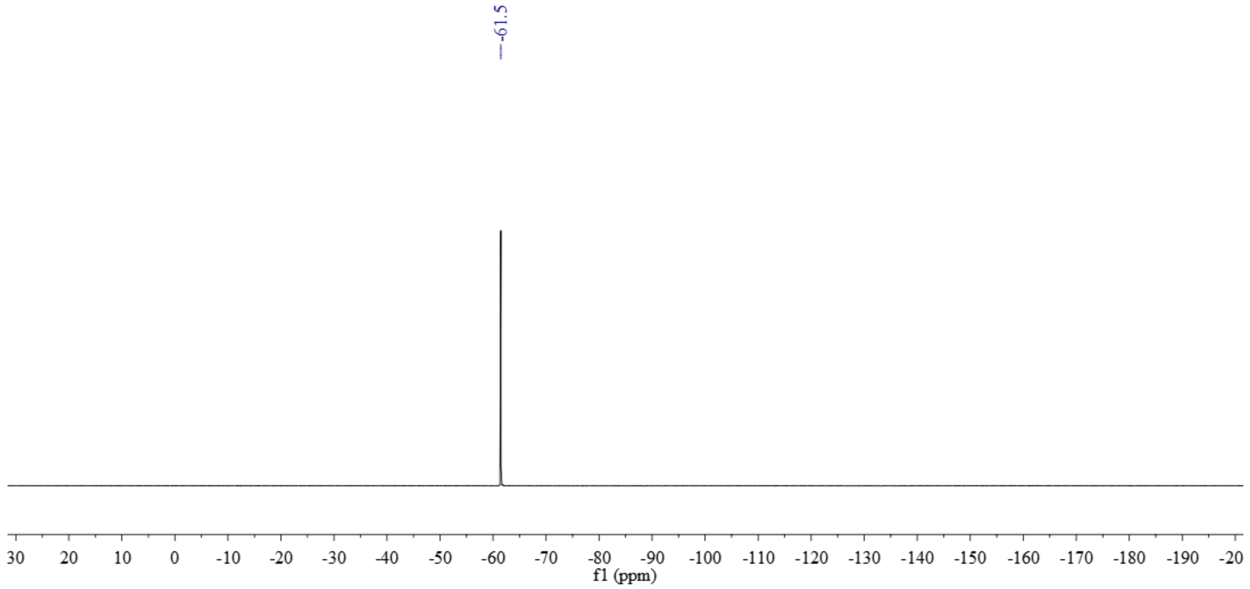

Supplementary Fig. 259. ^{31}F NMR spectrum of **69**. The sample has been recorded in 564 MHz, $CDCl_3$ at 25 °C.

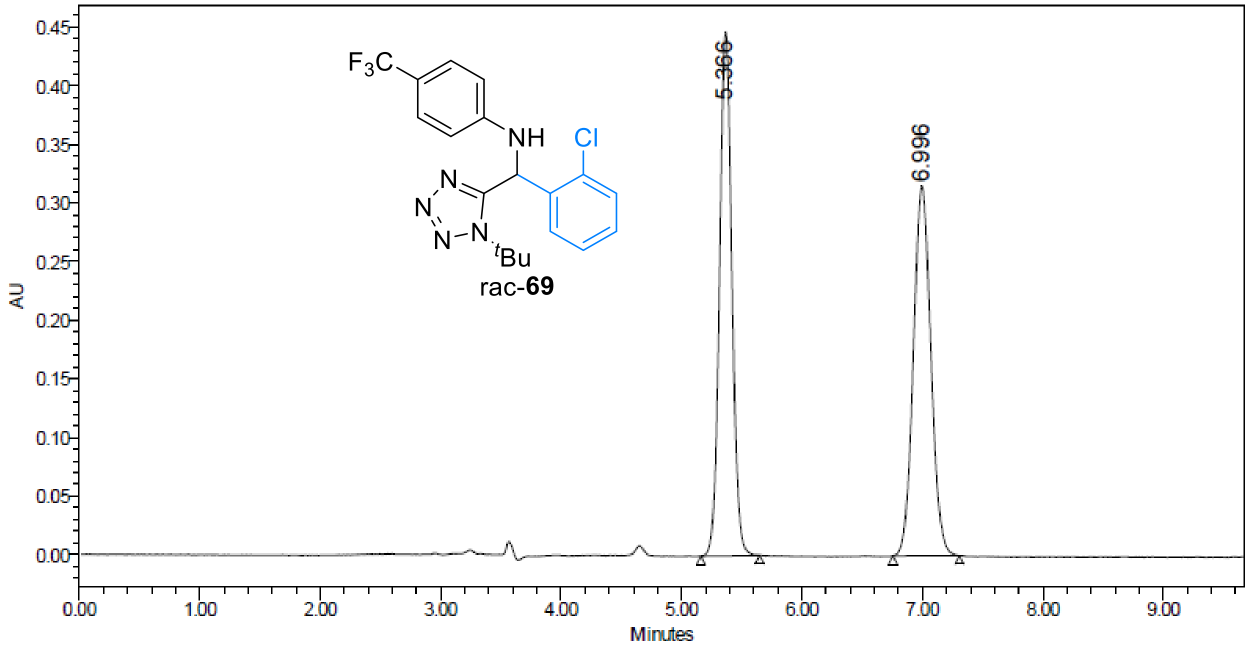

	RT (min)	Area (µV*sec)	% Area	Height (µV)	% Height
1	5.366	3187582	50.05	447333	58.63
2	6.996	3180857	49.95	315635	41.37

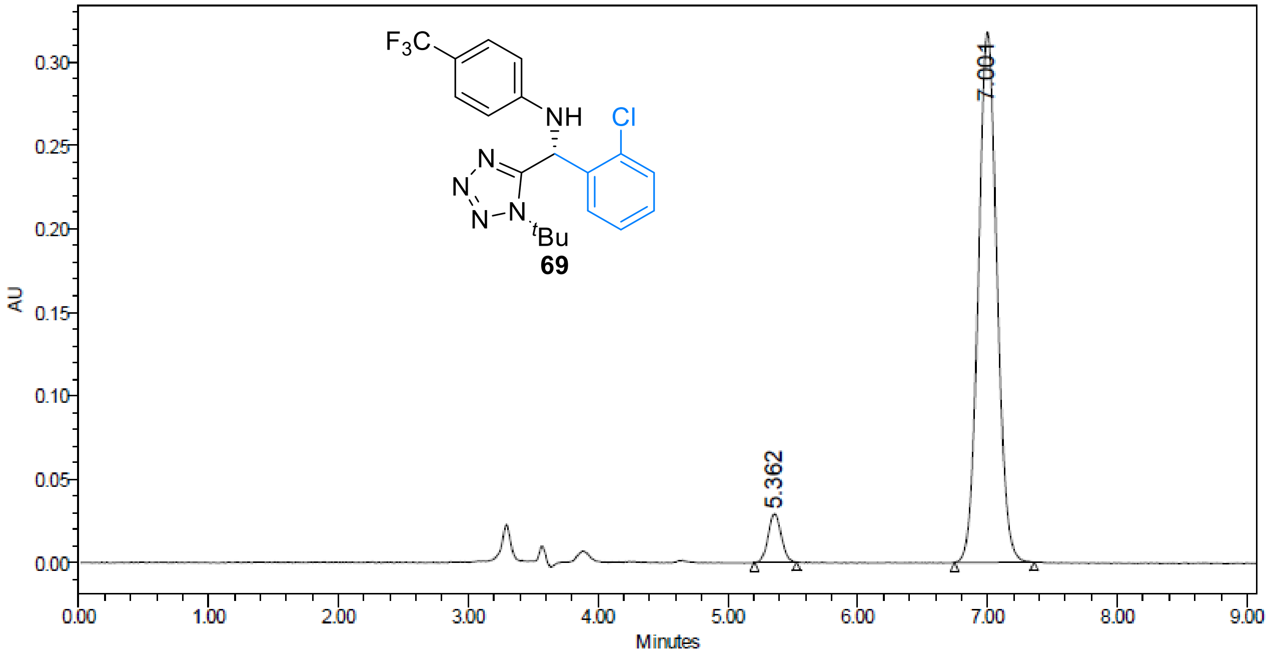

	RT (min)	Area (µV*sec)	% Area	Height (µV)	% Height
1	5.362	202814	6.01	29067	8.38
2	7.001	3173149	93.99	317951	91.62

Supplementary Fig. 260. HPLC of product **69**.

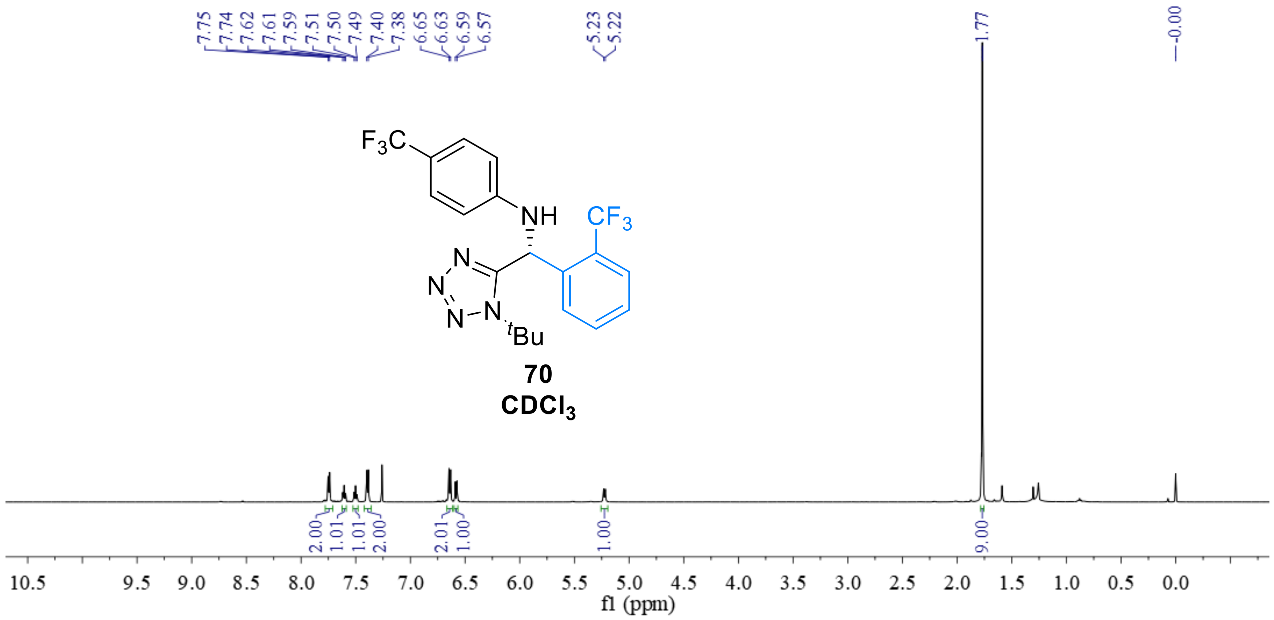

Supplementary Fig. 261. ^{1}H NMR spectrum of **70**. The sample has been recorded in 600 MHz, $CDCl_3$ at 25 °C.

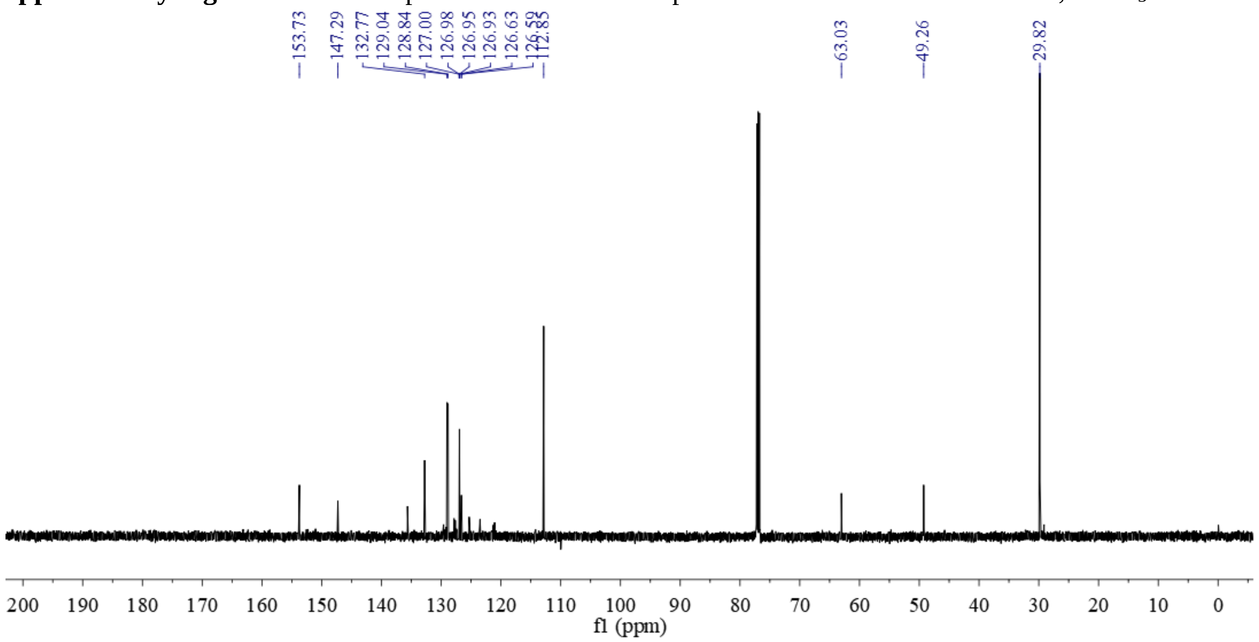

Supplementary Fig. 262. ^{13}C NMR spectrum of **70**. The sample has been recorded in 151 MHz, $CDCl_3$ at 25 °C.

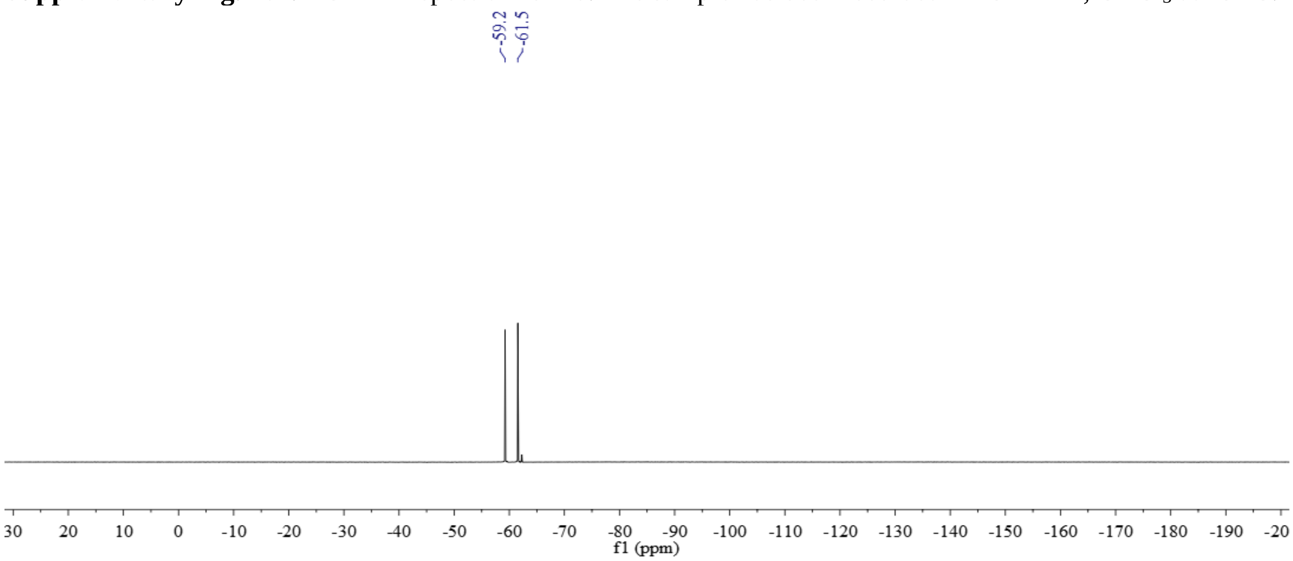

Supplementary Fig. 263. [31]F NMR spectrum of **70**. The sample has been recorded in 564 MHz, $CDCl_3$ at 25 °C.

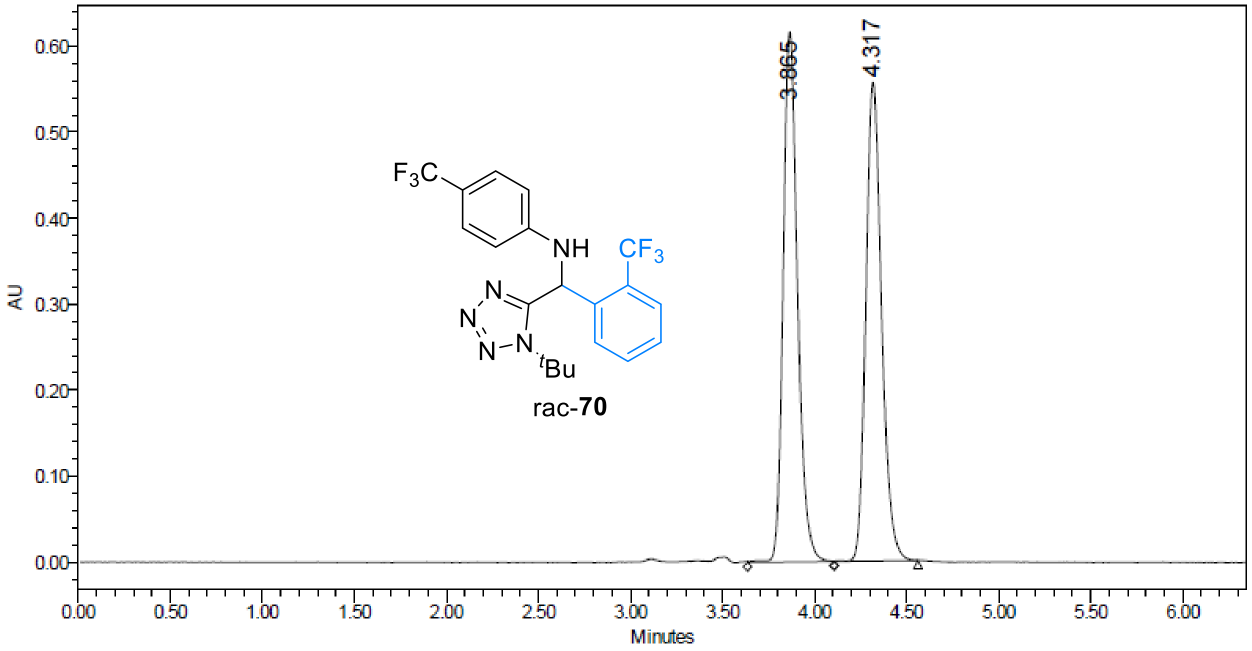

	RT (min)	Area (µV*sec)	% Area	Height (µV)	% Height
1	3.865	3343028	50.24	616627	52.53
2	4.317	3310464	49.76	557306	47.47

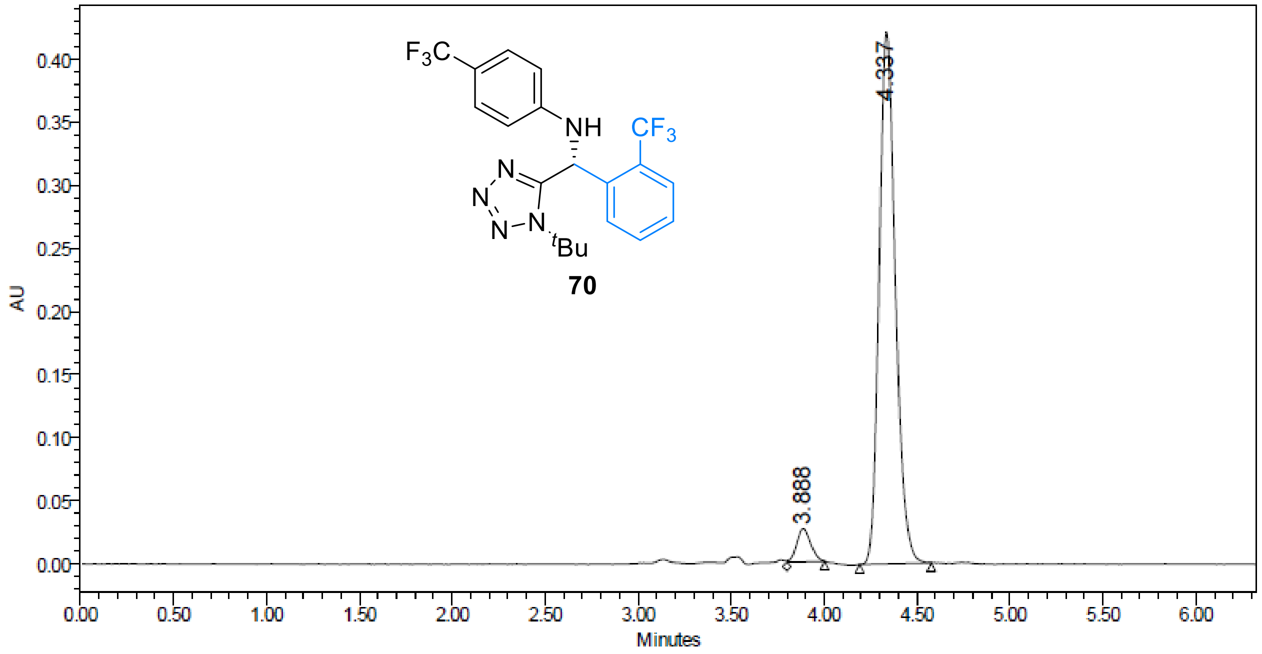

	RT (min)	Area (µV*sec)	% Area	Height (µV)	% Height
1	3.888	136987	5.08	26056	5.82
2	4.337	2559907	94.92	421842	94.18

Supplementary Fig. 264. HPLC of product **70**.

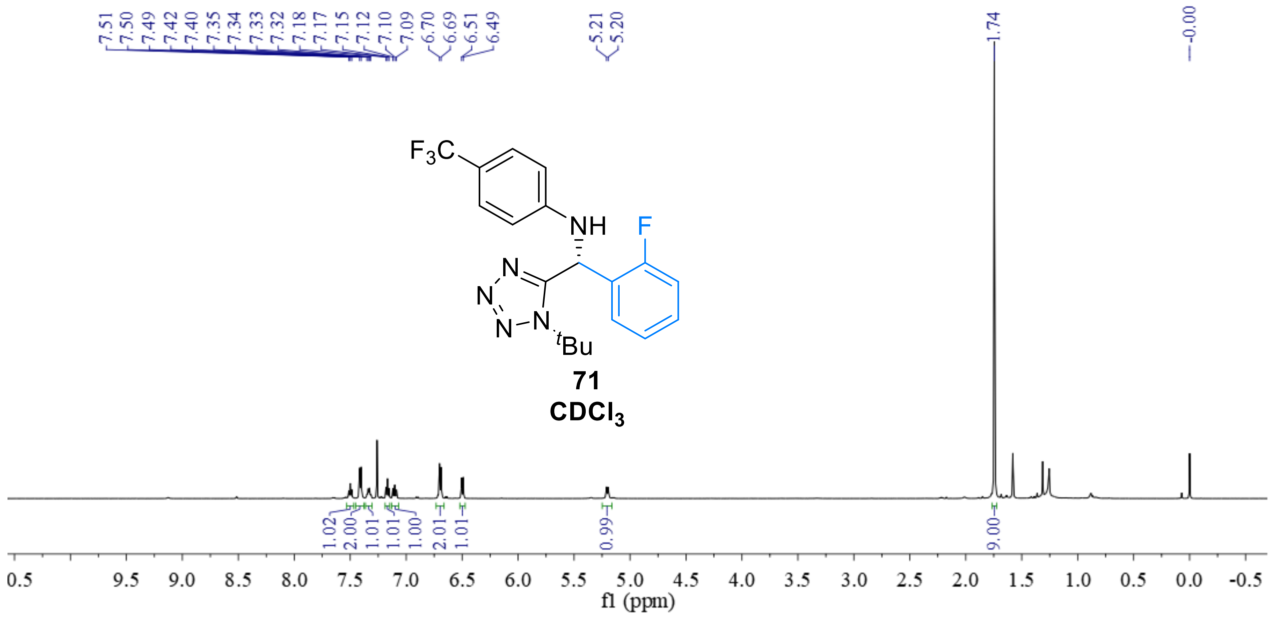

Supplementary Fig. 265. ^{1}H NMR spectrum of **71**. The sample has been recorded in 600 MHz, $CDCl_3$ at 25 °C.

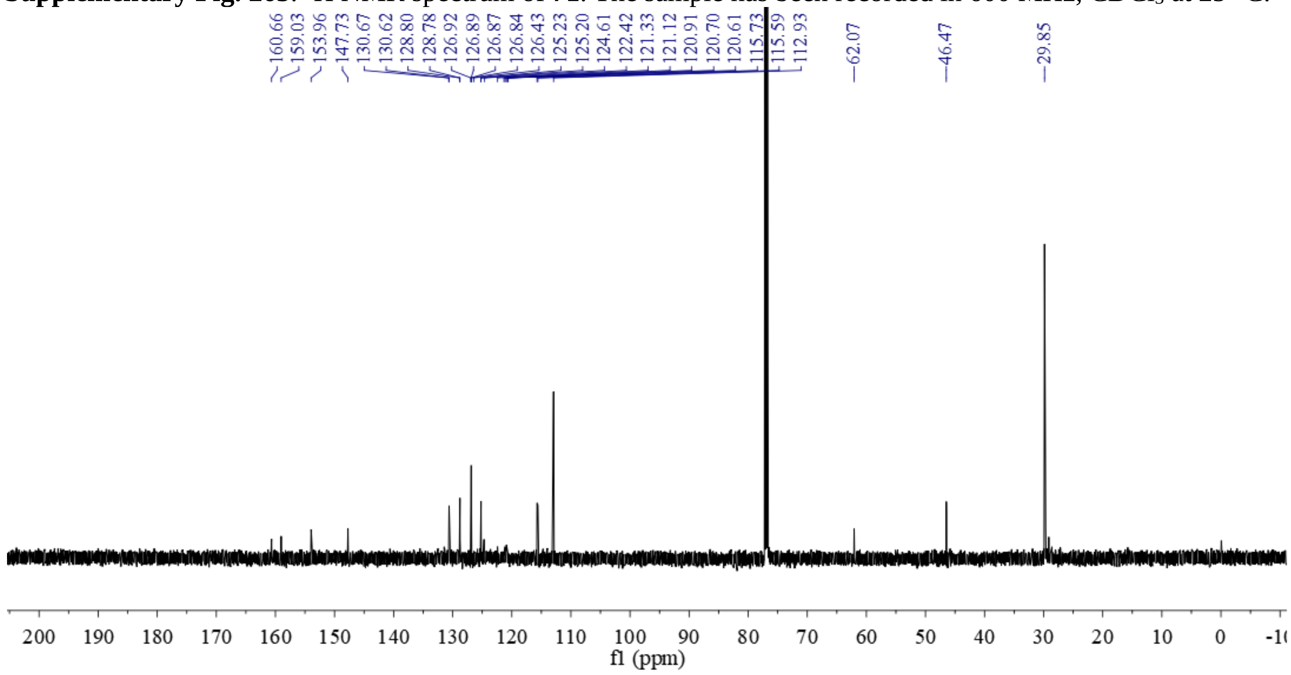

Supplementary Fig. 266. ^{13}C NMR spectrum of **71**. The sample has been recorded in 151 MHz, $CDCl_3$ at 25 °C.

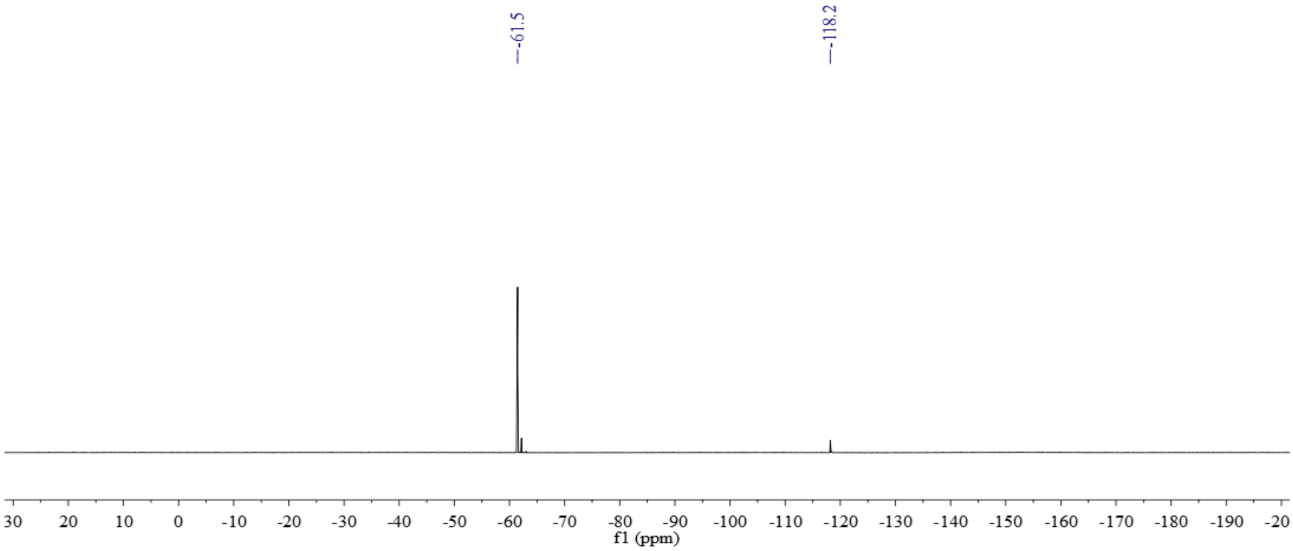

Supplementary Fig. 267. ^{31}F NMR spectrum of **71**. The sample has been recorded in 564 MHz, $CDCl_3$ at 25 °C.

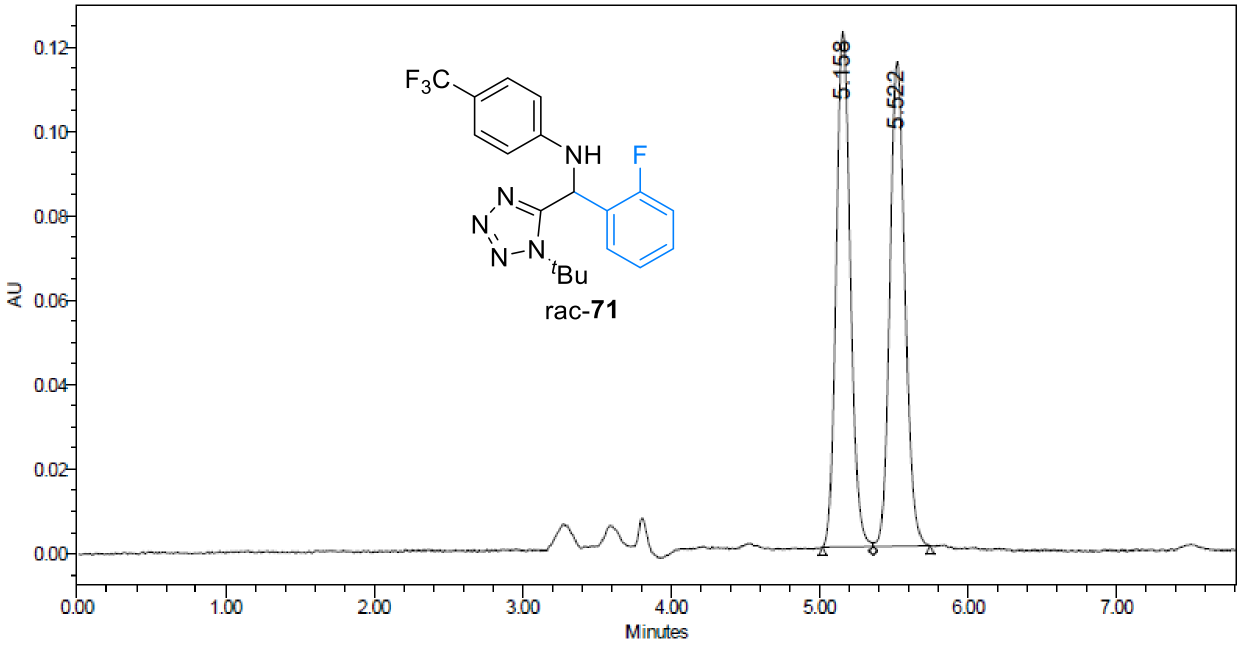

	RT (min)	Area (µV*sec)	% Area	Height (µV)	% Height
1	5.158	810826	49.96	122040	51.52
2	5.522	812238	50.04	114822	48.48

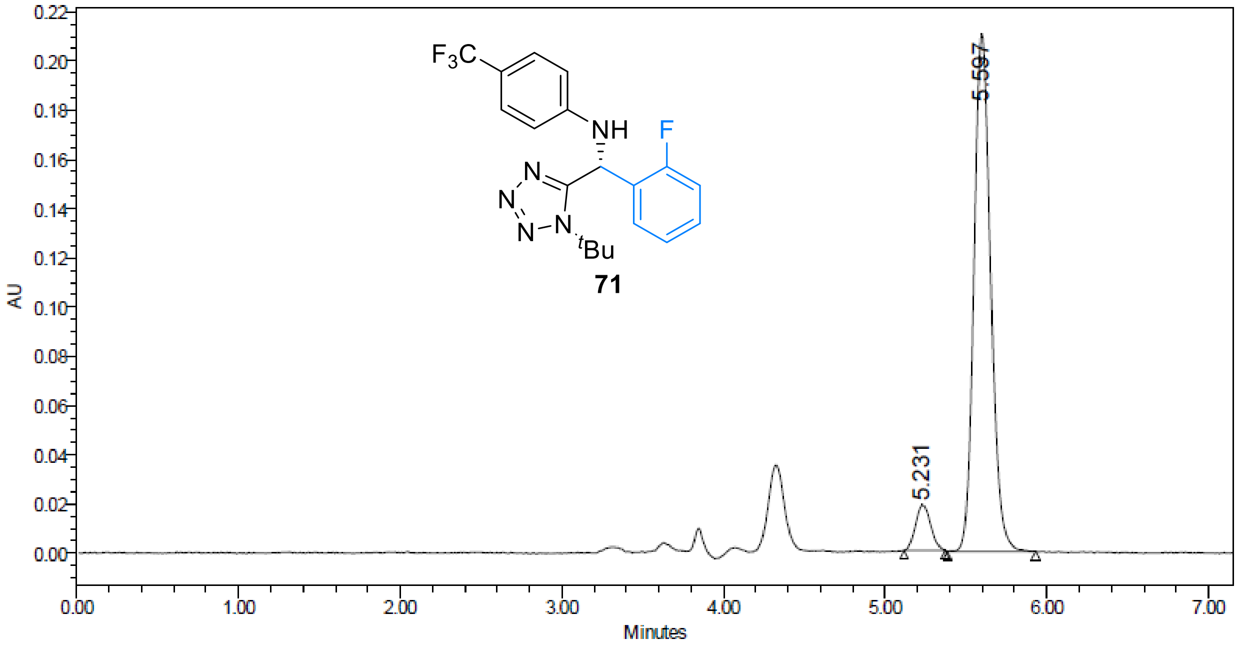

	RT (min)	Area (µV*sec)	% Area	Height (µV)	% Height
1	5.231	120183	7.37	18494	8.08
2	5.597	1510996	92.63	210395	91.92

Supplementary Fig. 268. HPLC of product **71**.

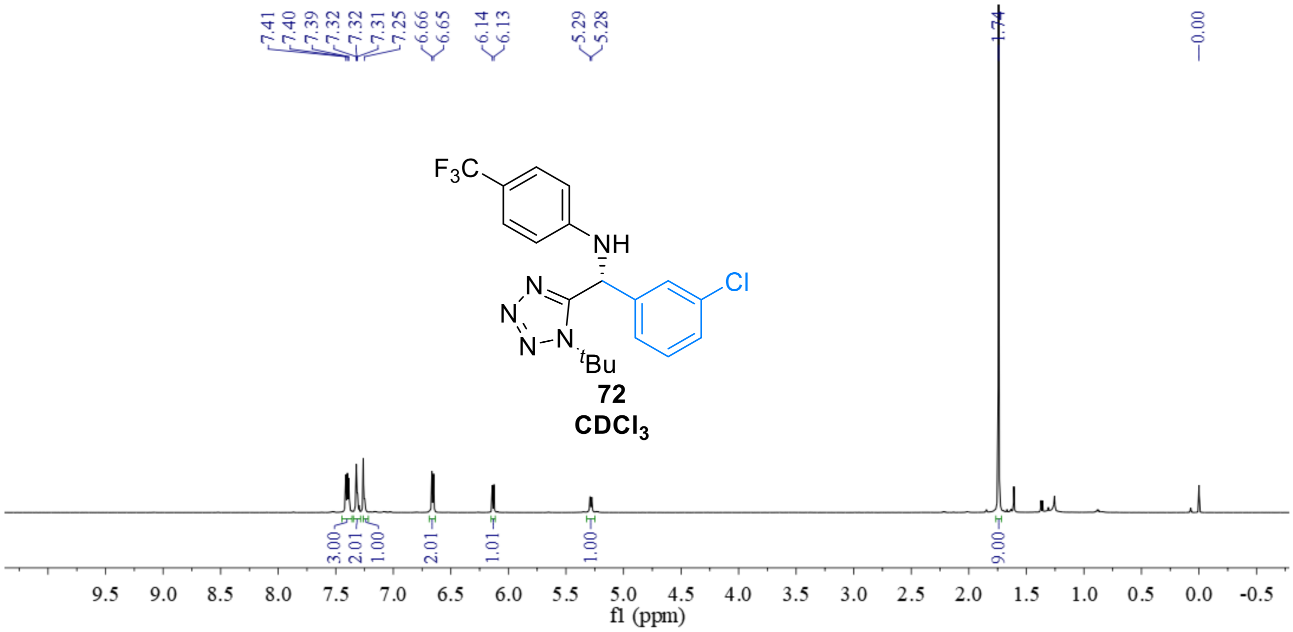

Supplementary Fig. 269. ^{1}H NMR spectrum of **72**. The sample has been recorded in 600 MHz, $CDCl_3$ at 25 °C.

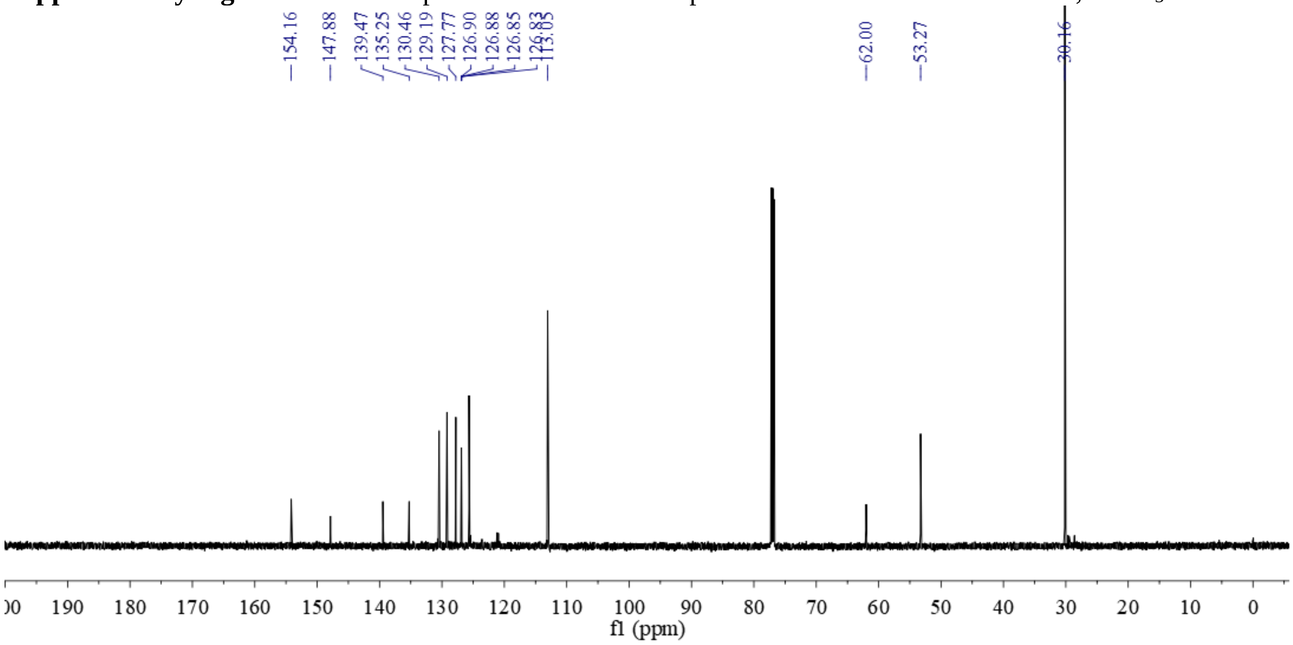

Supplementary Fig. 270. ^{13}C NMR spectrum of **72**. The sample has been recorded in 151 MHz, $CDCl_3$ at 25 °C.

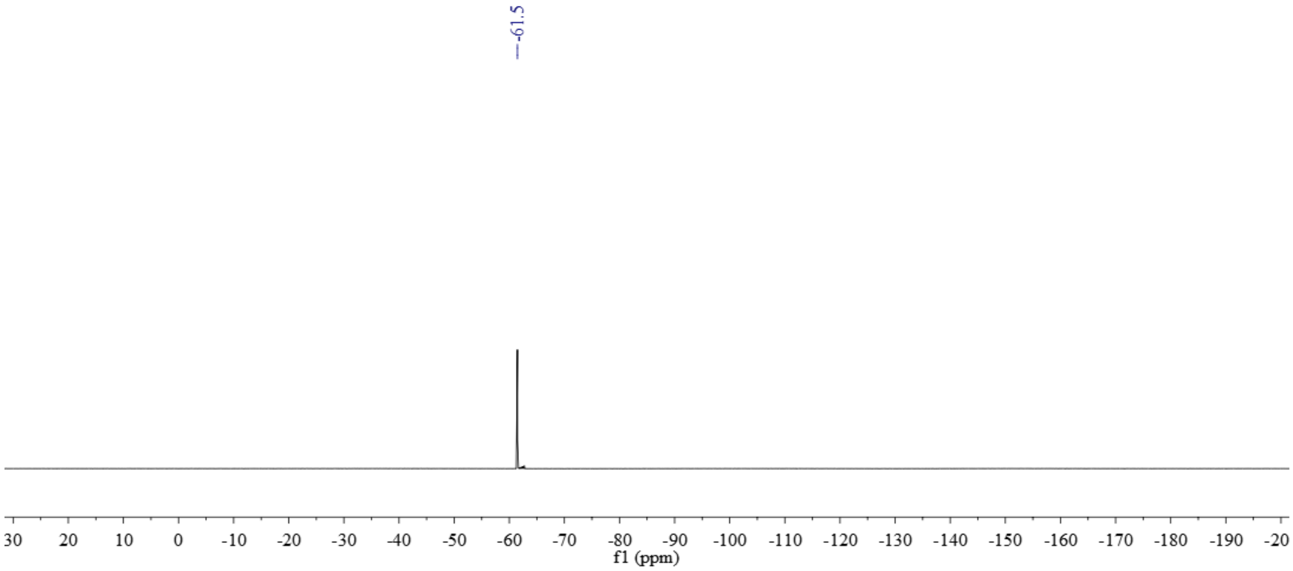

Supplementary Fig. 271. ^{31}F NMR spectrum of **72**. The sample has been recorded in 564 MHz, $CDCl_3$ at 25 °C.

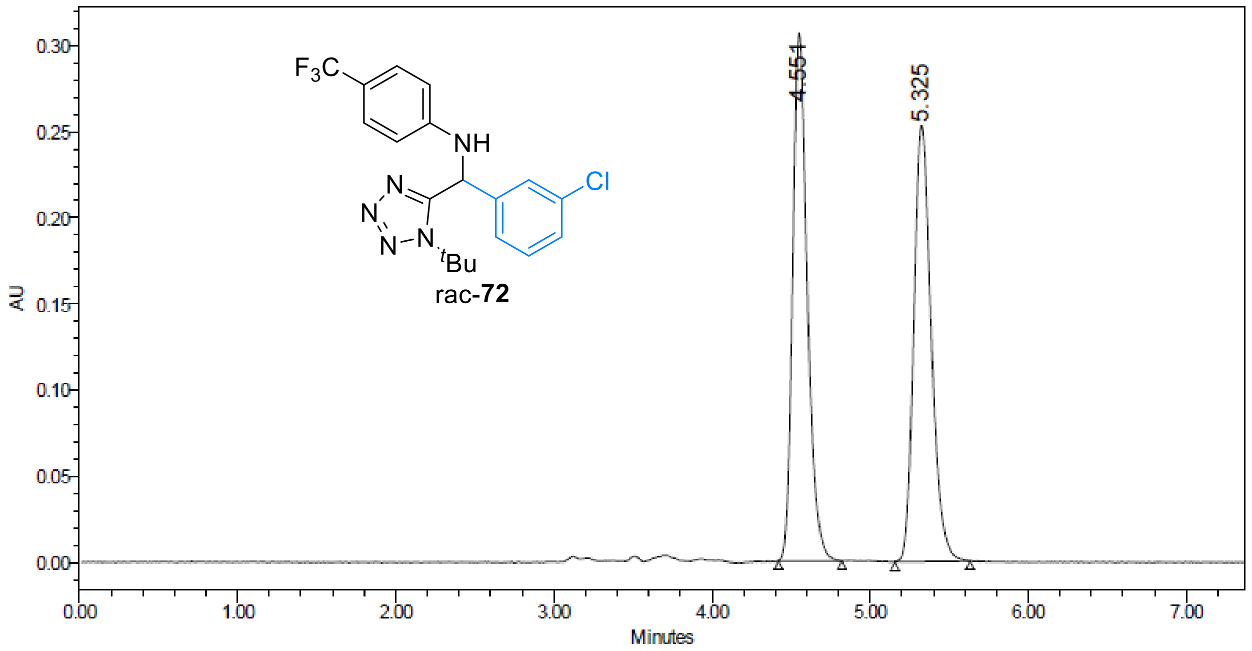

	RT (min)	Area (µV*sec)	% Area	Height (µV)	% Height
1	4.551	1977913	50.48	306663	54.80
2	5.325	1939955	49.52	252905	45.20

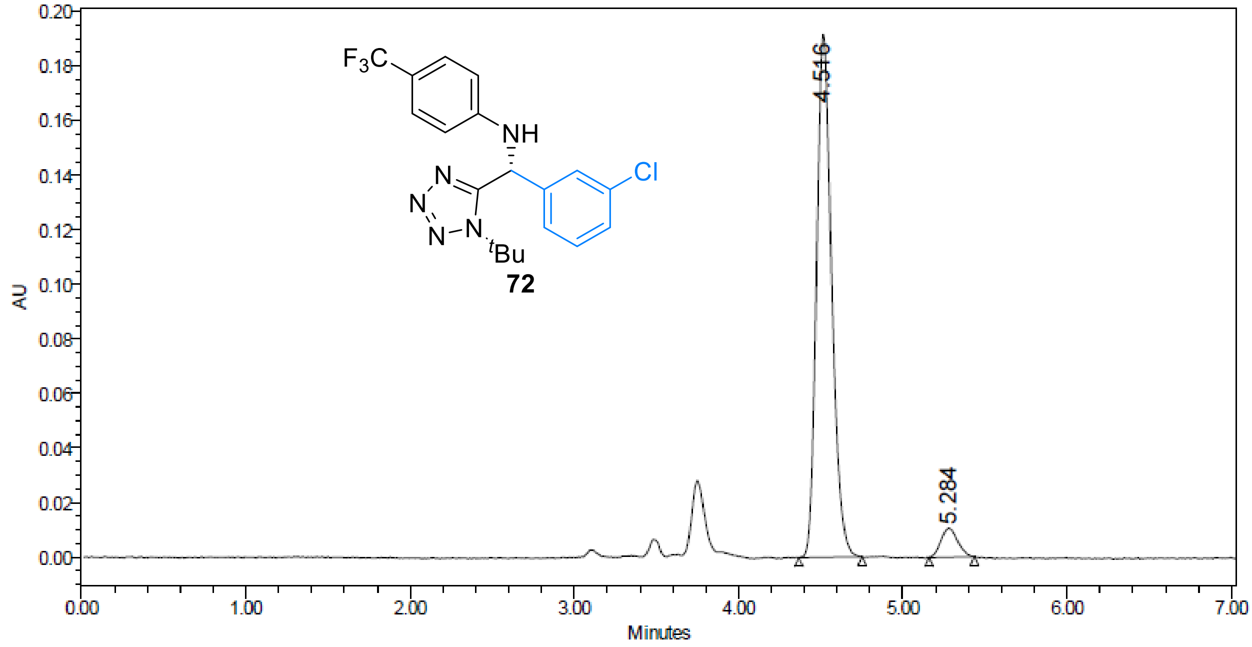

	RT (min)	Area (µV*sec)	% Area	Height (µV)	% Height
1	4.516	1226166	94.13	191728	94.76
2	5.284	76456	5.87	10593	5.24

Supplementary Fig. 272. HPLC of product **72**.

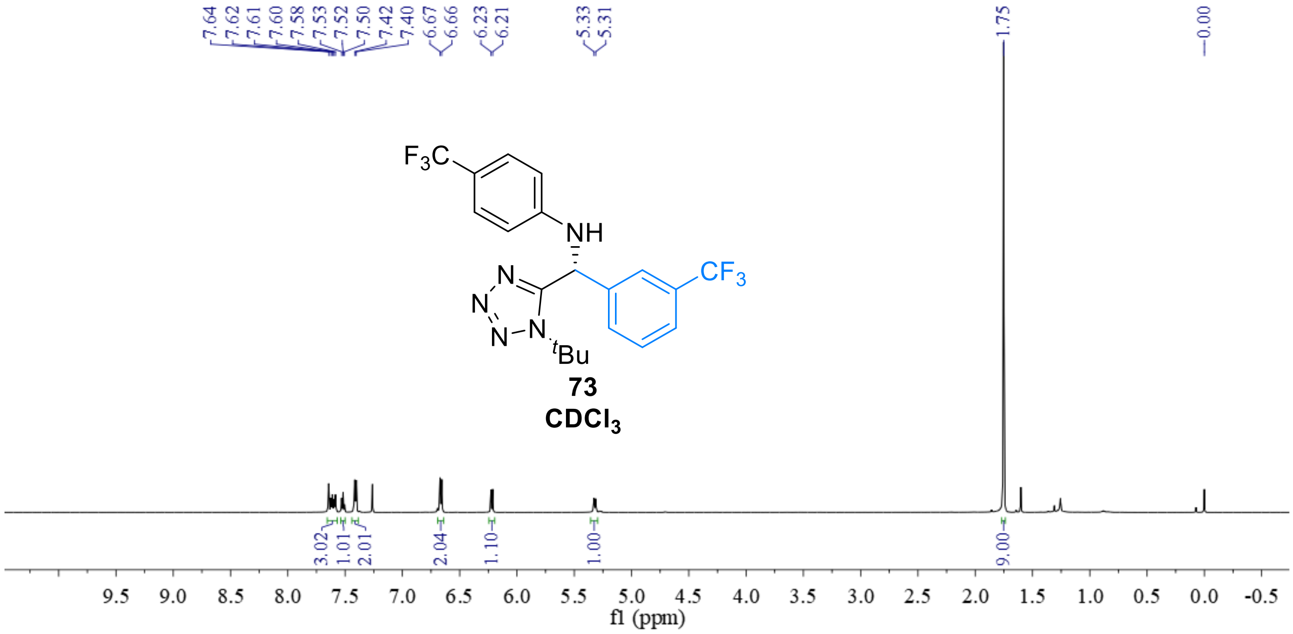

Supplementary Fig. 273. ^{1}H NMR spectrum of **73**. The sample has been recorded in 600 MHz, $CDCl_3$ at 25 °C.

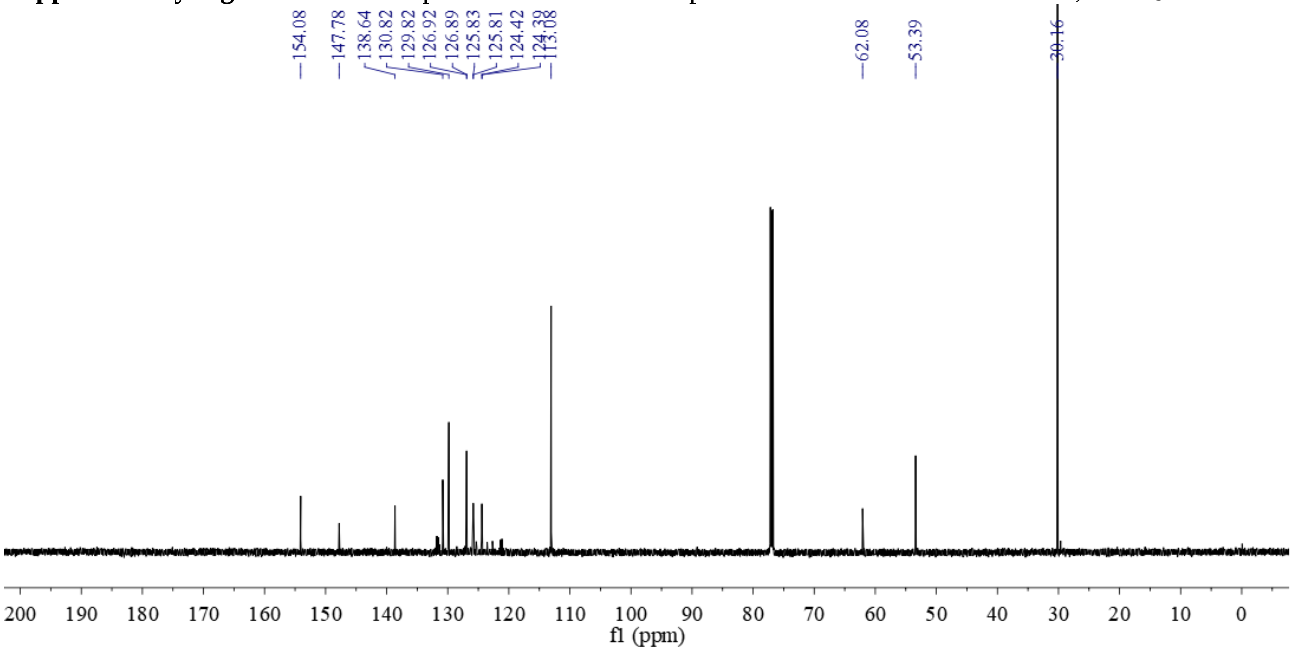

Supplementary Fig. 274. ^{13}C NMR spectrum of **73**. The sample has been recorded in 151 MHz, $CDCl_3$ at 25 °C.

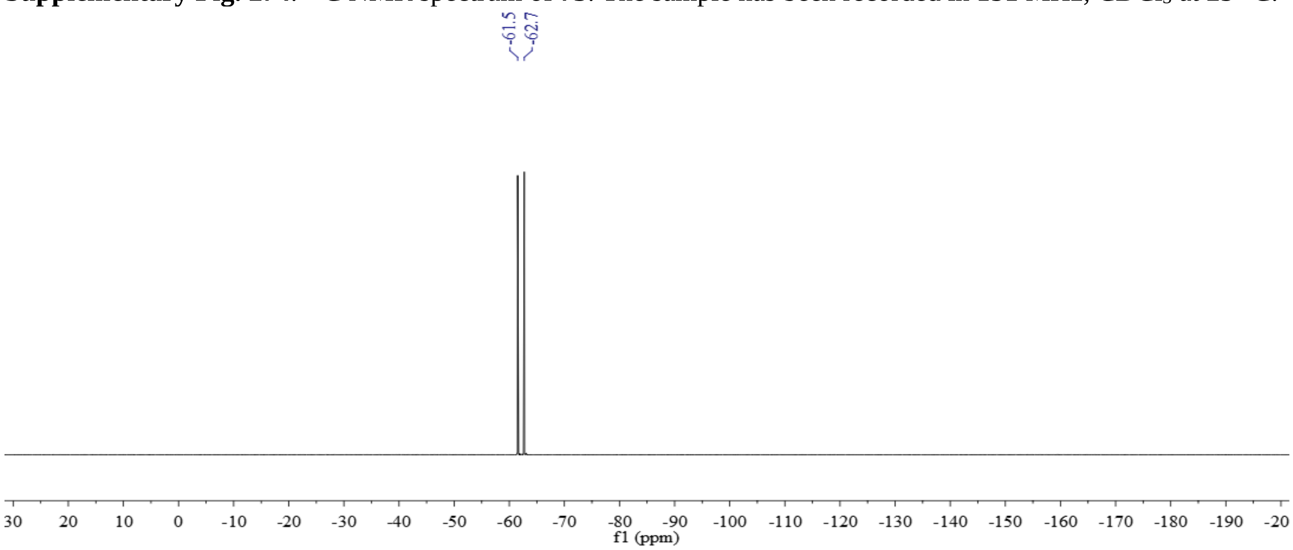

Supplementary Fig. 275. ^{31}F NMR spectrum of **73**. The sample has been recorded in 564 MHz, $CDCl_3$ at 25 °C.

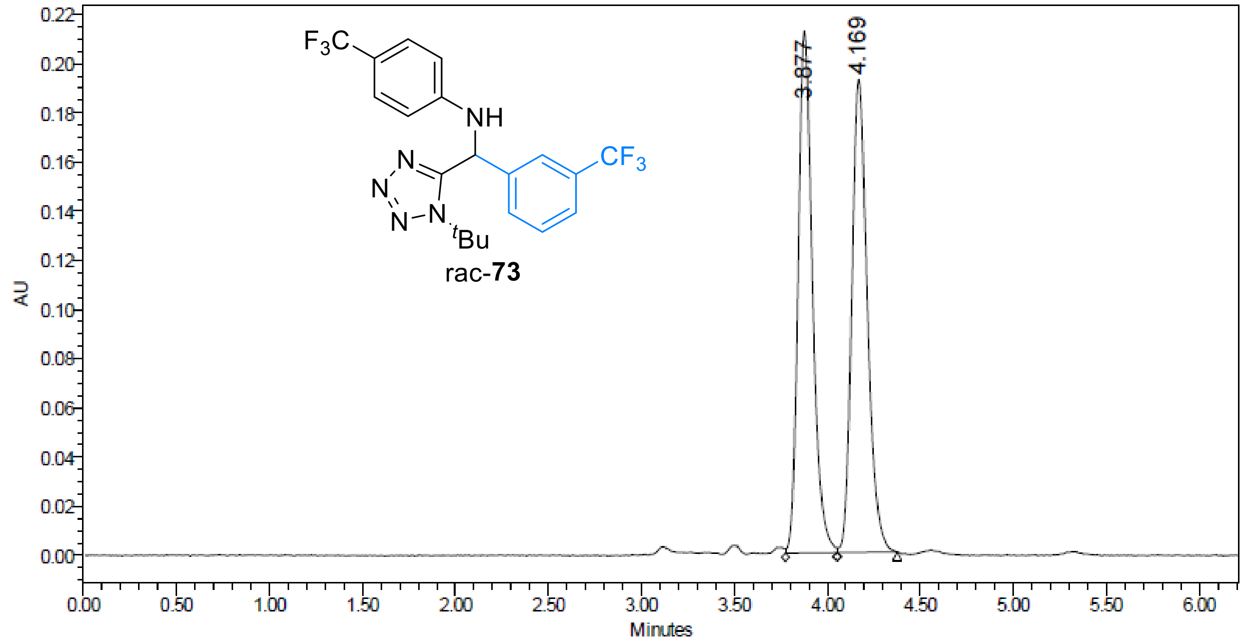

	RT (min)	Area (µV*sec)	% Area	Height (µV)	% Height
1	3.877	1135423	50.35	212200	52.44
2	4.169	1119564	49.65	192431	47.56

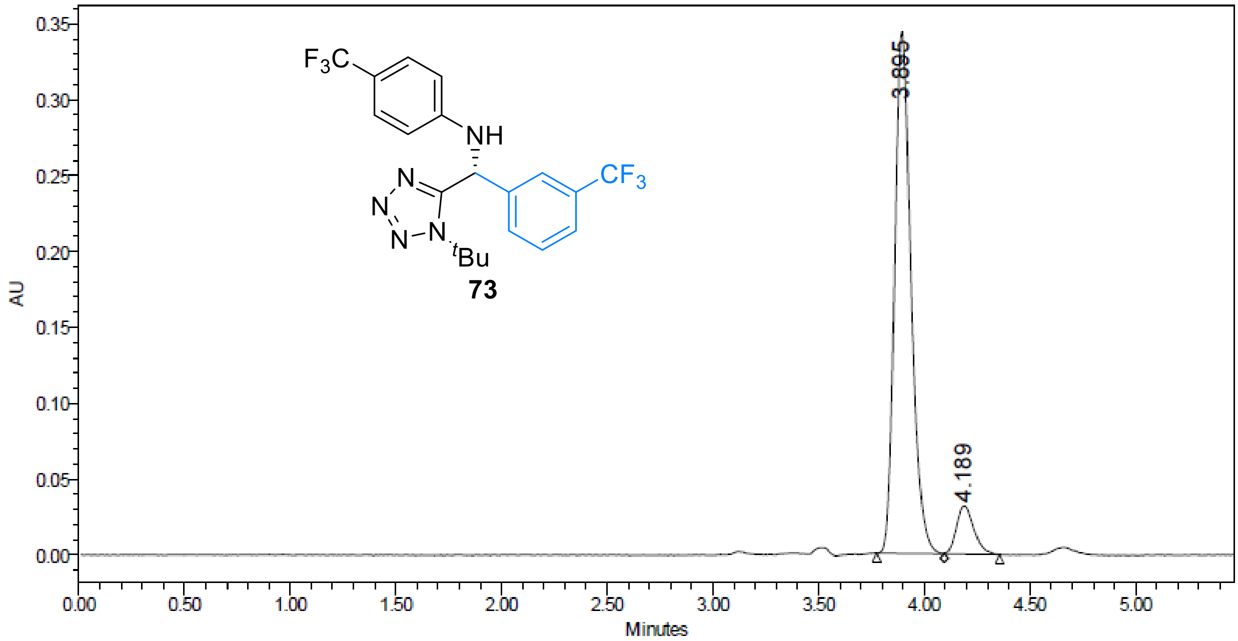

	RT (min)	Area (µV*sec)	% Area	Height (µV)	% Height
1	3.895	1850522	91.01	343904	91.56
2	4.189	182711	8.99	31712	8.44

Supplementary Fig. 276. HPLC of product **73**.

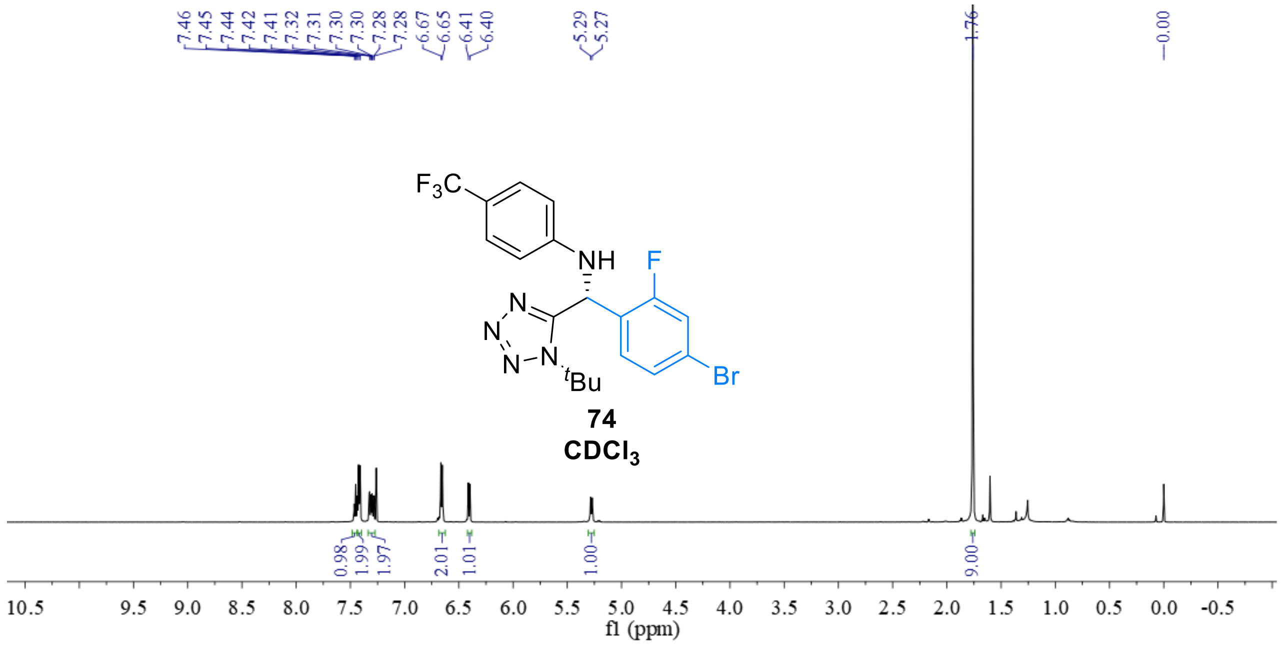

Supplementary Fig. 277. ^{1}H NMR spectrum of **74**. The sample has been recorded in 600 MHz, $CDCl_3$ at 25 °C.

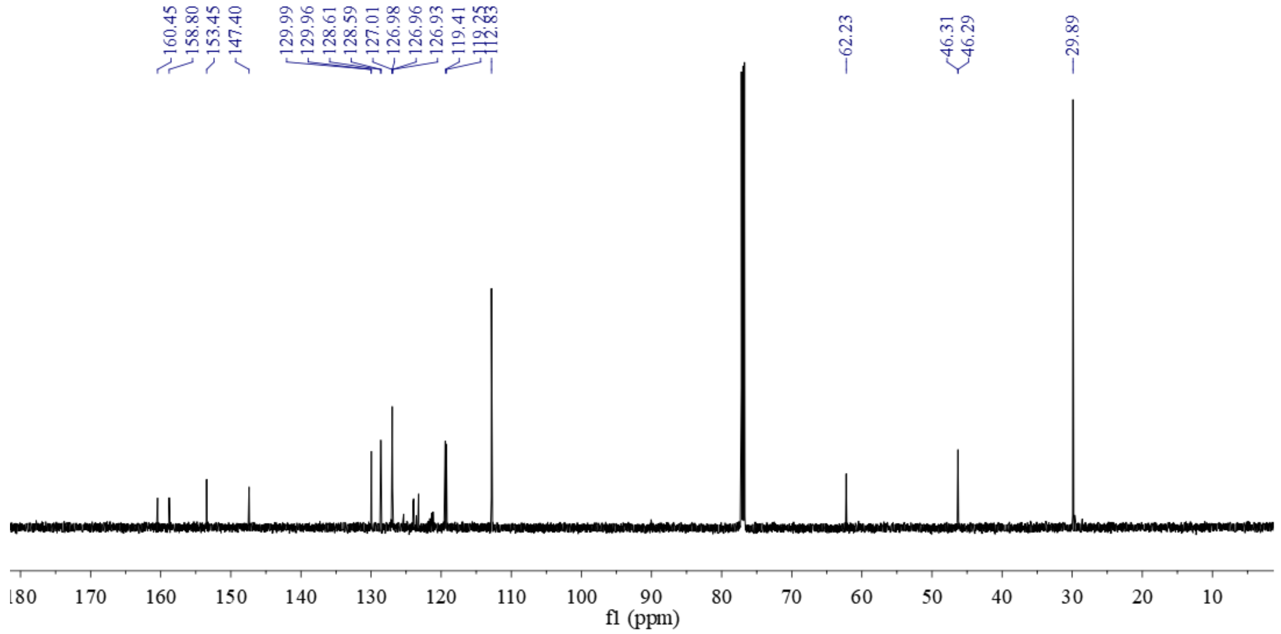

Supplementary Fig. 278. ^{13}C NMR spectrum of **74**. The sample has been recorded in 151 MHz, $CDCl_3$ at 25 °C.

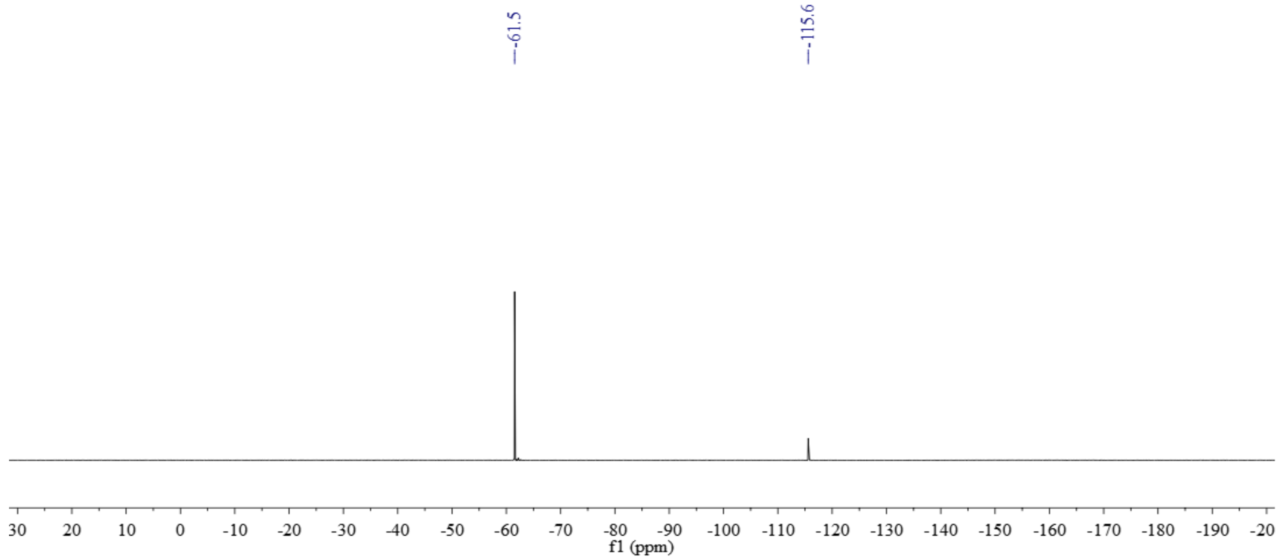

Supplementary Fig. 279. [31]F NMR spectrum of **74**. The sample has been recorded in 564 MHz, $CDCl_3$ at 25 °C.

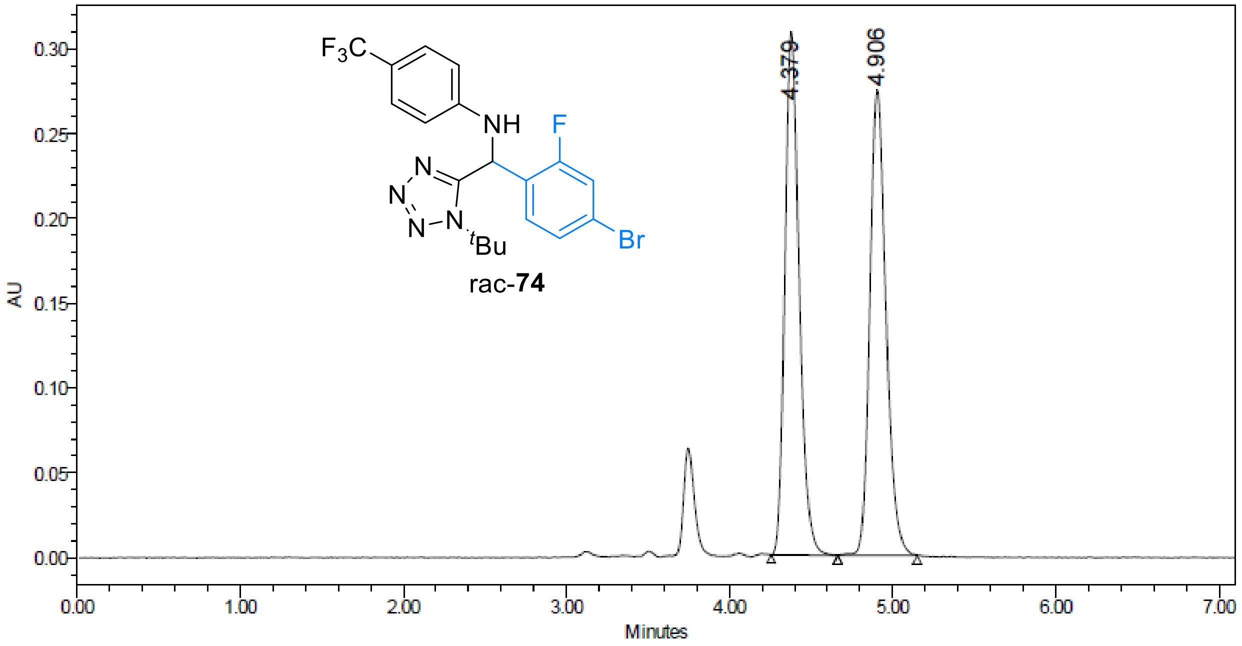

	RT (min)	Area (µV*sec)	% Area	Height (µV)	% Height
1	4.379	1888643	49.93	308783	52.93
2	4.906	1893825	50.07	274623	47.07

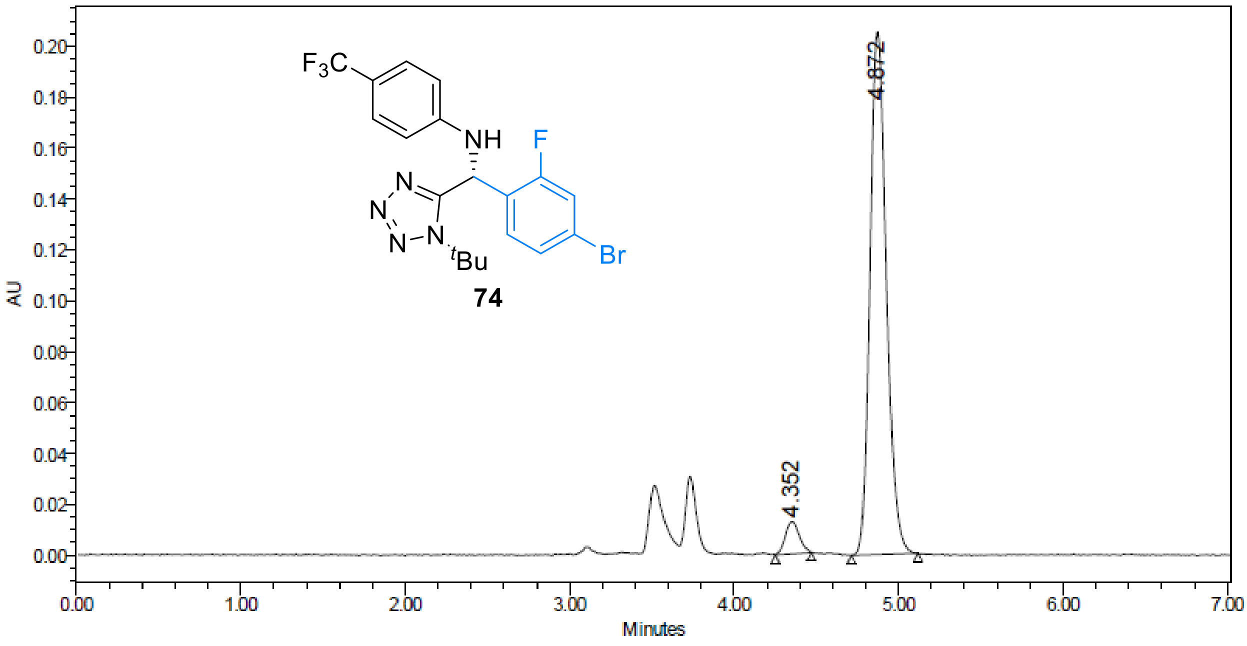

	RT (min)	Area (µV*sec)	% Area	Height (µV)	% Height
1	4.352	75330	5.04	12730	5.84
2	4.872	1417894	94.96	205329	94.16

Supplementary Fig. 280. HPLC of product **74**.

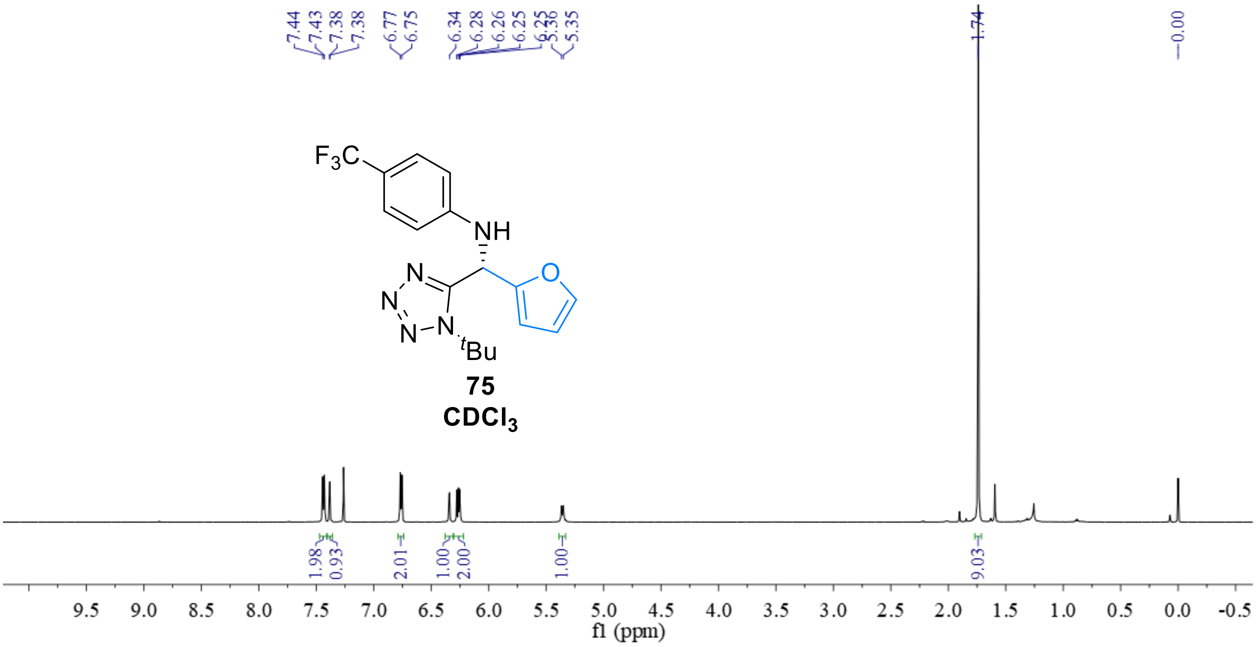

Supplementary Fig. 281. ^{1}H NMR spectrum of **75**. The sample has been recorded in 600 MHz, $CDCl_3$ at 25 °C.

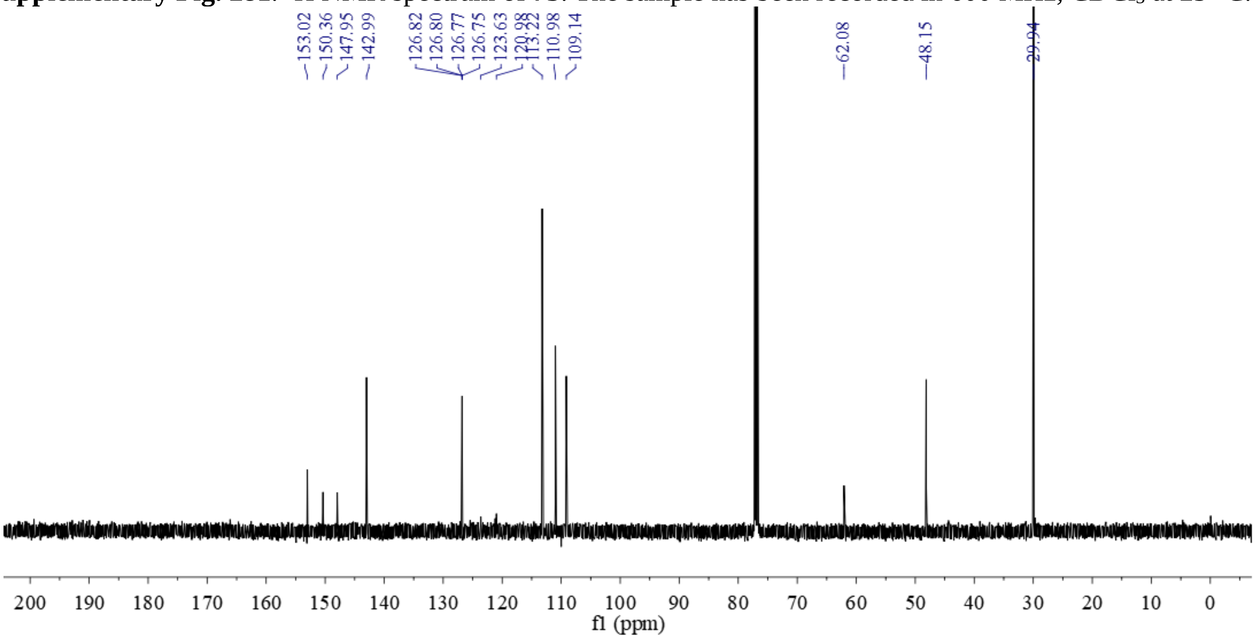

Supplementary Fig. 282. ^{13}C NMR spectrum of **75**. The sample has been recorded in 151 MHz, $CDCl_3$ at 25 °C.

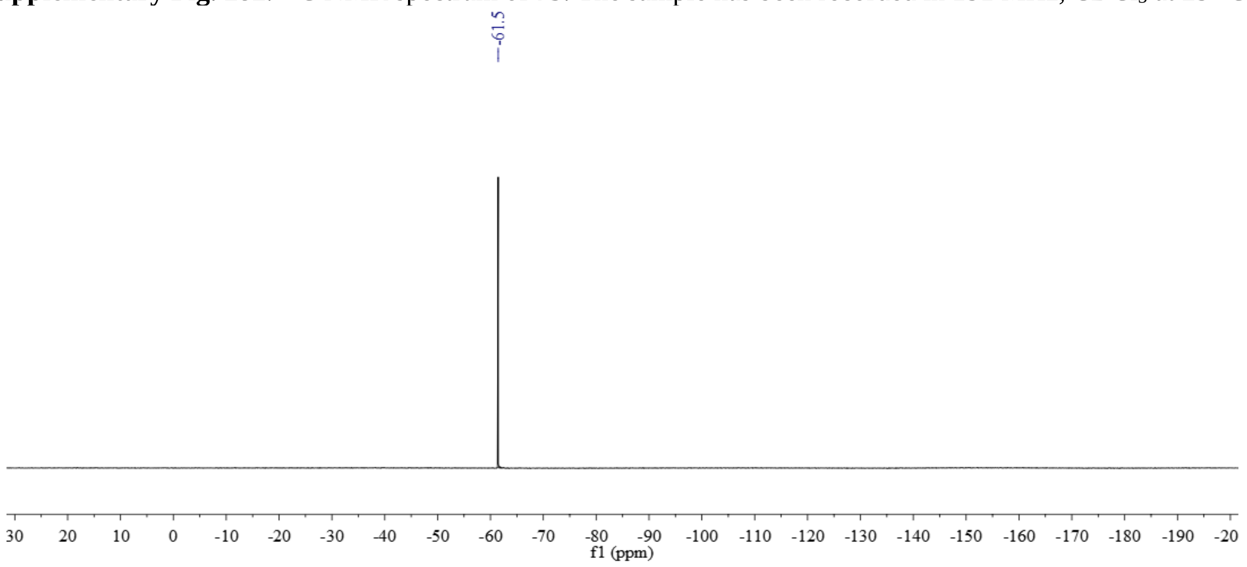

Supplementary Fig. 283. [31]F NMR spectrum of **75**. The sample has been recorded in 564 MHz, $CDCl_3$ at 25 °C.

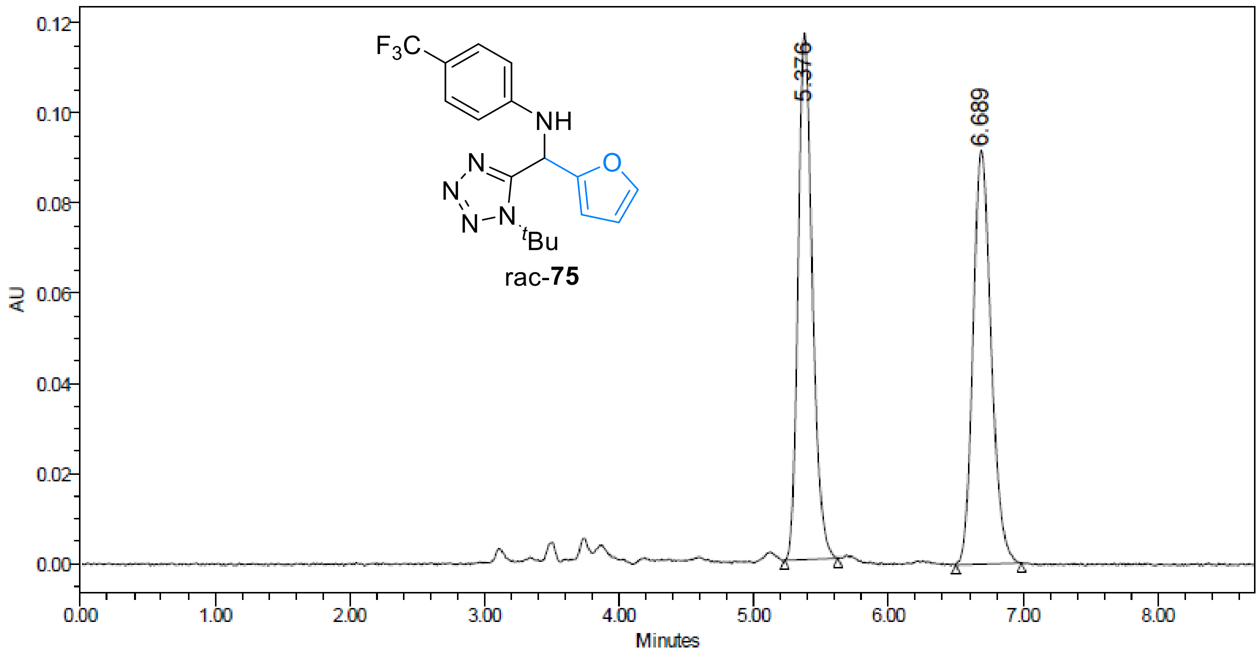

	RT (min)	Area (µV*sec)	% Area	Height (µV)	% Height
1	5.376	860317	50.04	116764	55.99
2	6.689	859036	49.96	91790	44.01

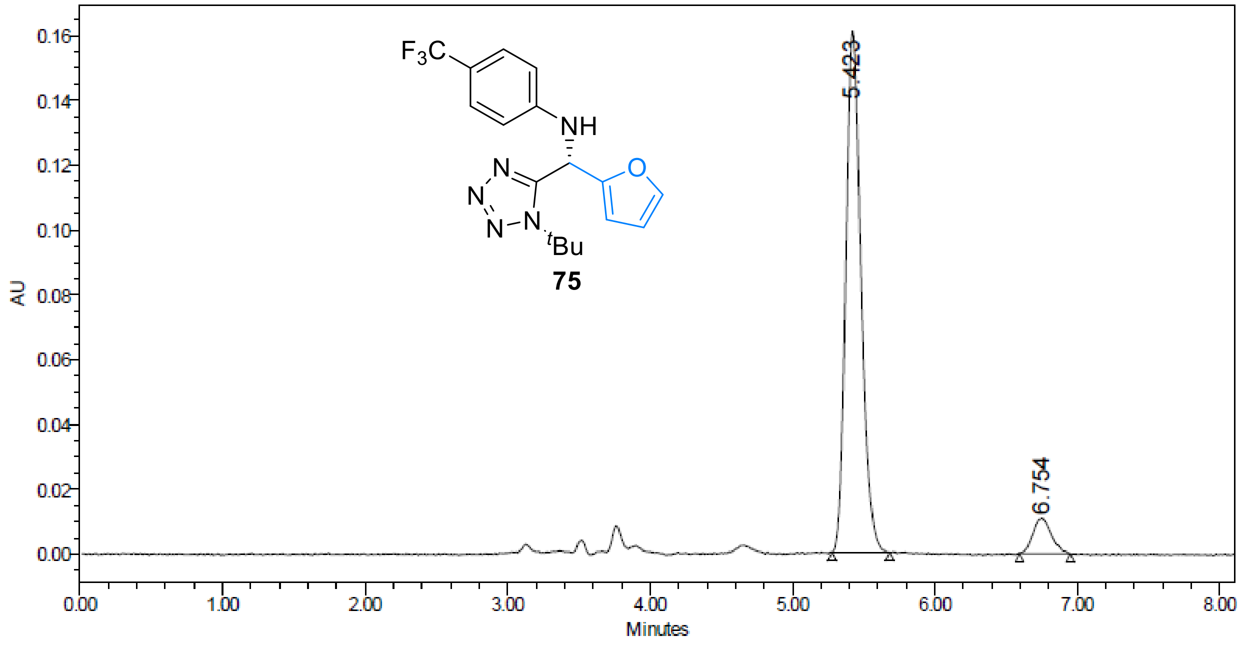

	RT (min)	Area (µV*sec)	% Area	Height (µV)	% Height
1	5.423	1194025	92.35	160866	93.64
2	6.754	98861	7.65	10931	6.36

Supplementary Fig. 284. HPLC of product **75**.

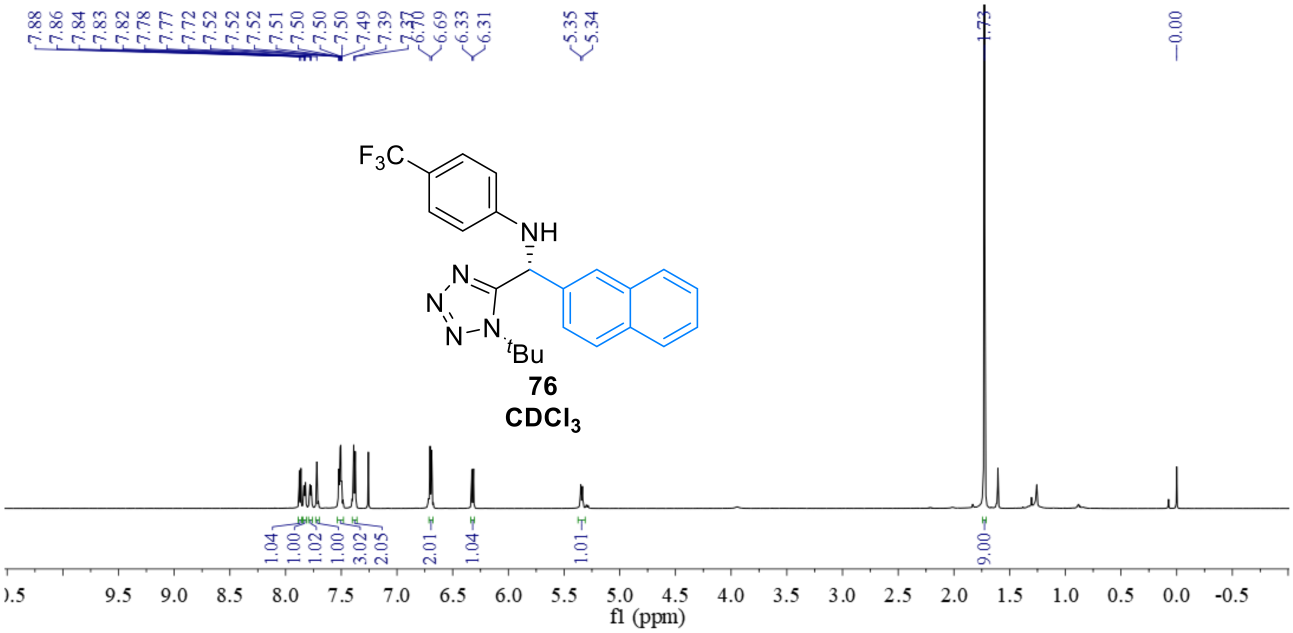

Supplementary Fig. 285. ^{1}H NMR spectrum of **76**. The sample has been recorded in 600 MHz, $CDCl_3$ at 25 °C.

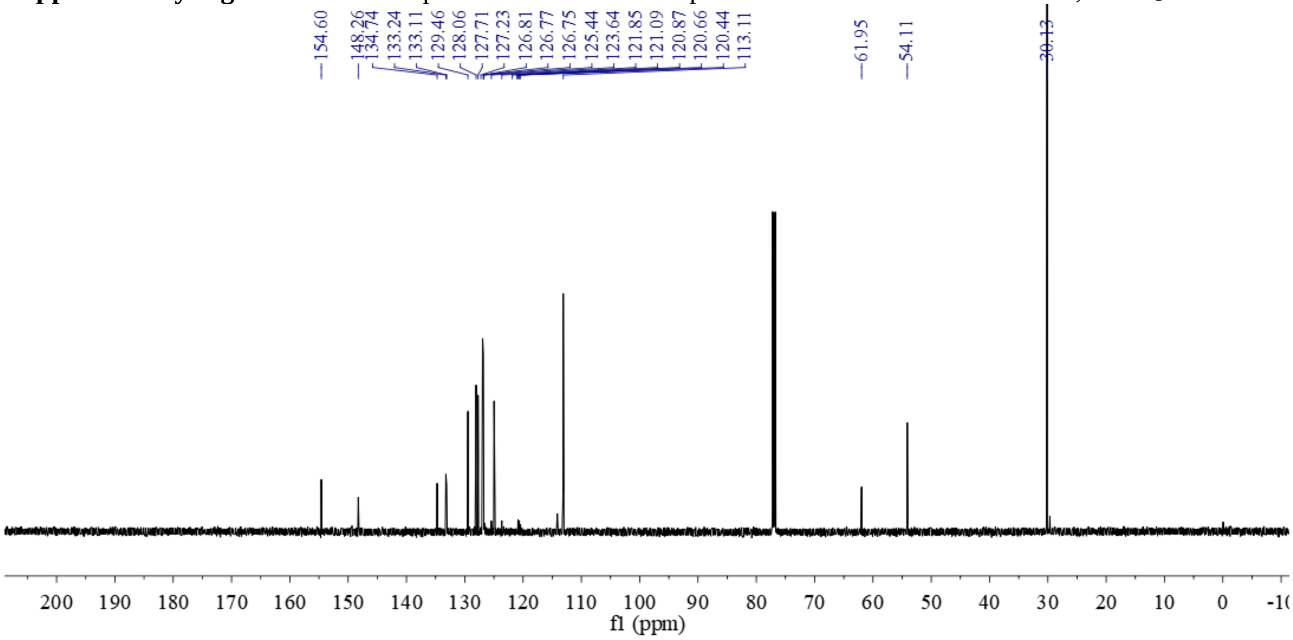

Supplementary Fig. 286. ^{13}C NMR spectrum of **76**. The sample has been recorded in 151 MHz, $CDCl_3$ at 25 °C.

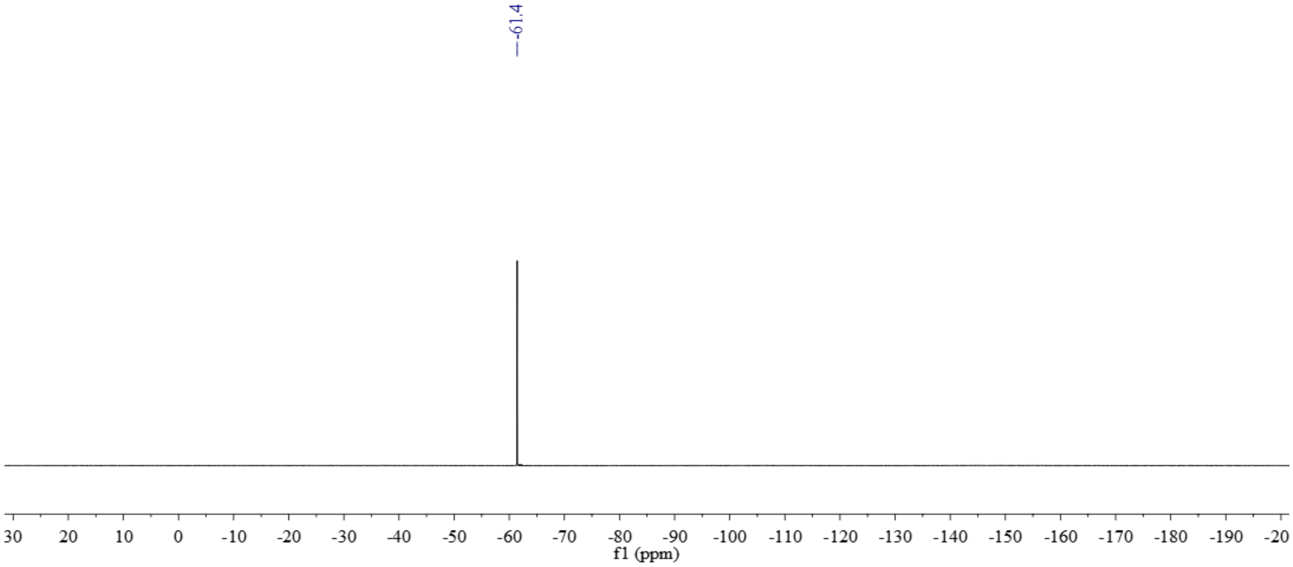

Supplementary Fig. 287. ^{31}F NMR spectrum of **76**. The sample has been recorded in 564 MHz, $CDCl_3$ at 25 °C.

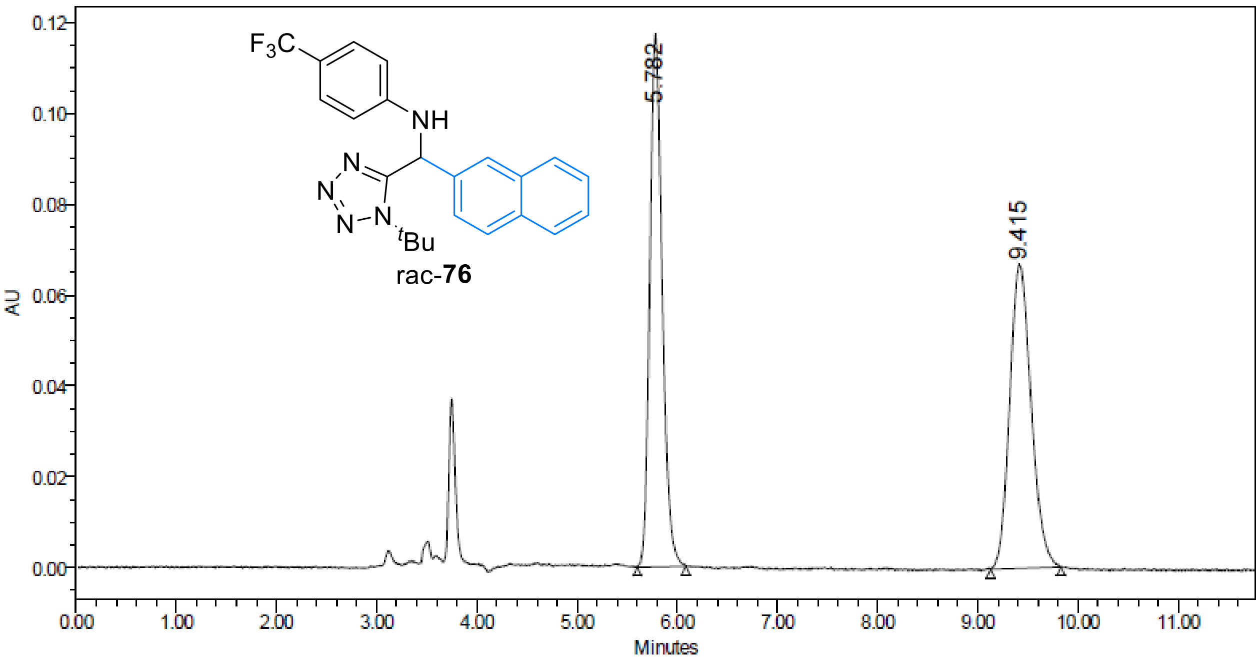

	RT (min)	Area (µV*sec)	% Area	Height (µV)	% Height
1	5.782	1014222	50.35	117474	63.67
2	9.415	1000130	49.65	67022	36.33

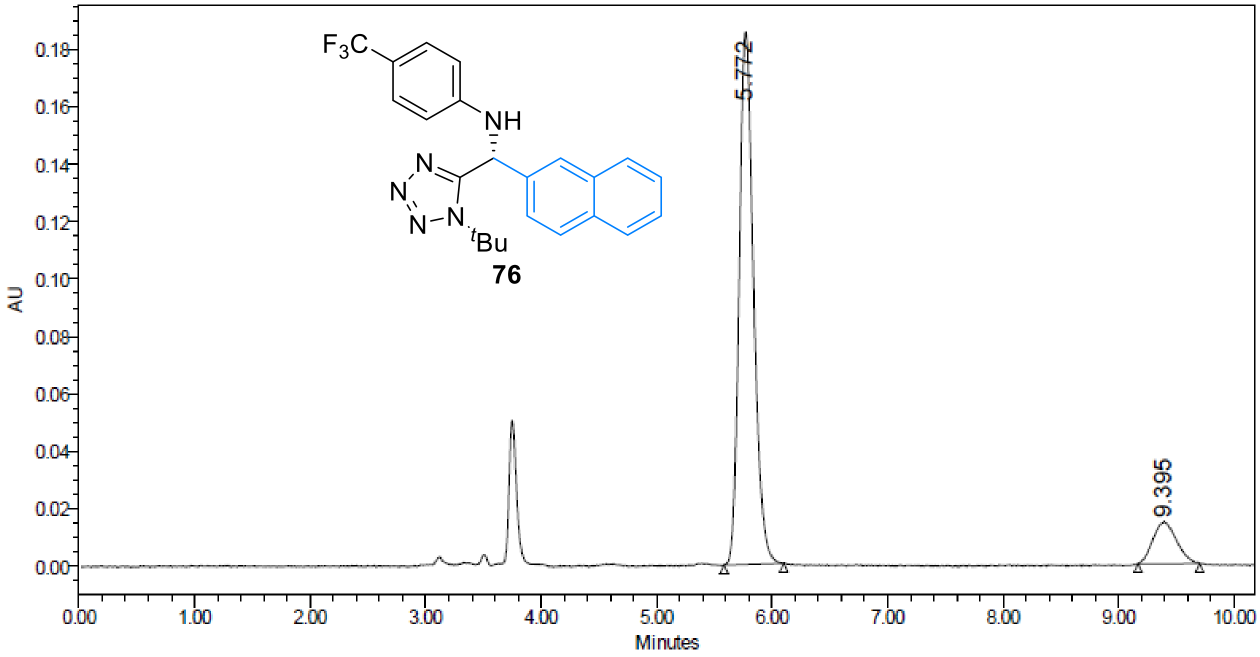

	RT (min)	Area (µV*sec)	% Area	Height (µV)	% Height
1	5.772	1605316	88.52	185525	92.70
2	9.395	208269	11.48	14617	7.30

Supplementary Fig. 288. HPLC of product **76**.

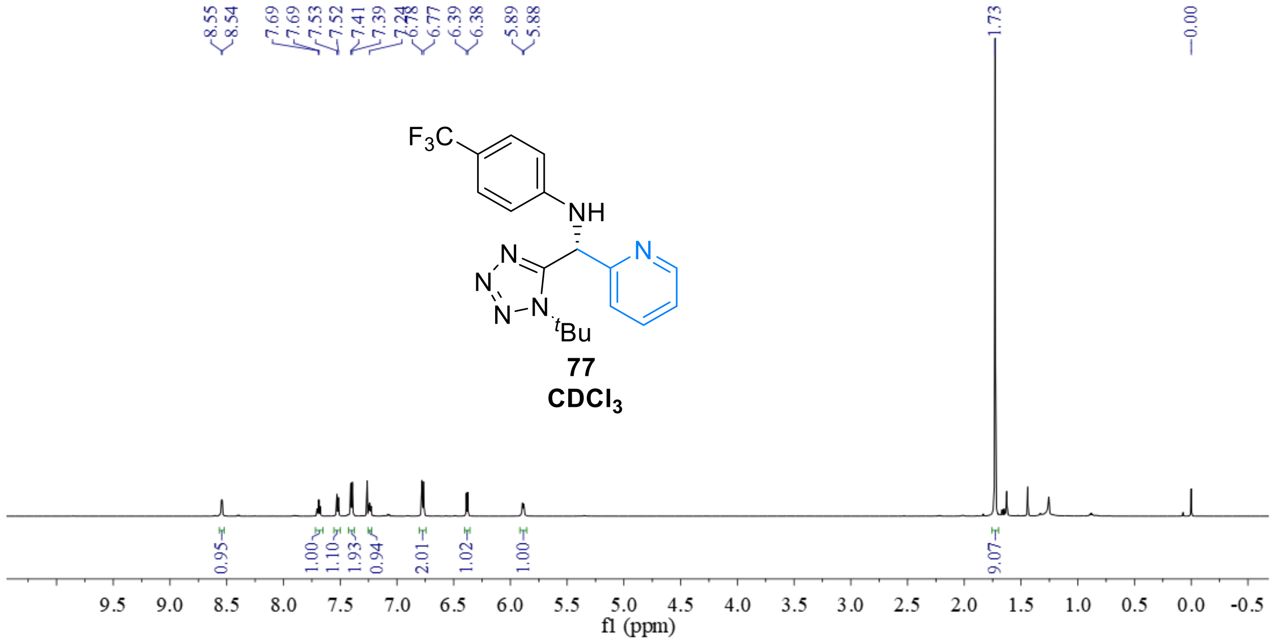

Supplementary Fig. 289. ^{1}H NMR spectrum of **77**. The sample has been recorded in 600 MHz, $CDCl_3$ at 25 °C.

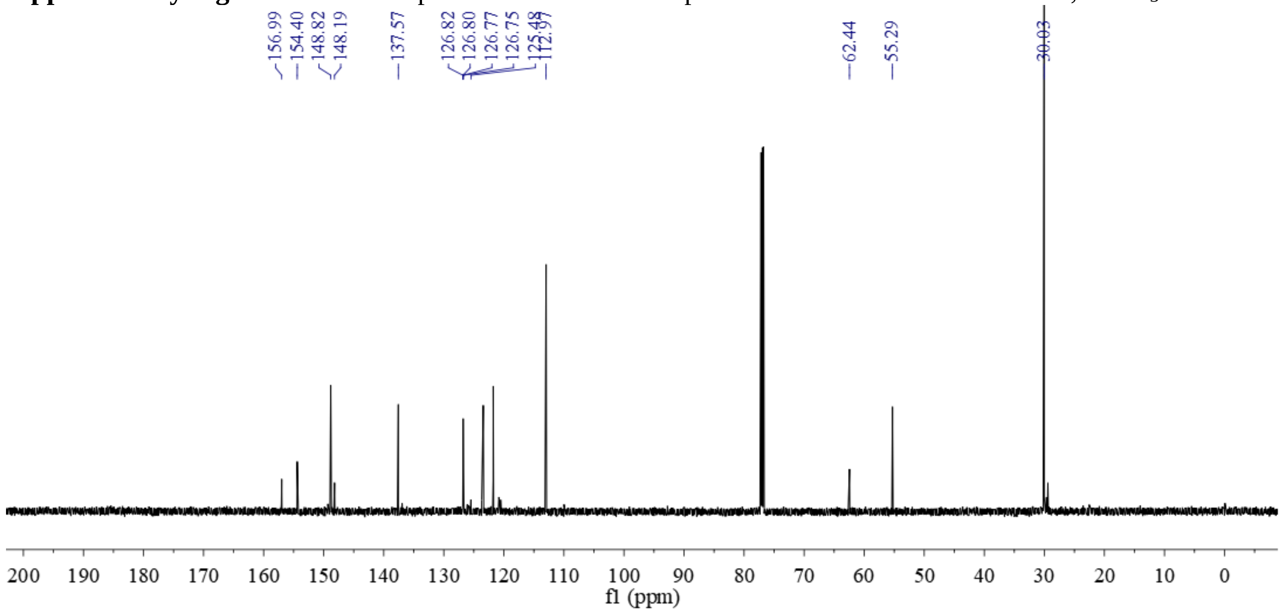

Supplementary Fig. 290. ^{13}C NMR spectrum of **77**. The sample has been recorded in 151 MHz, $CDCl_3$ at 25 °C.

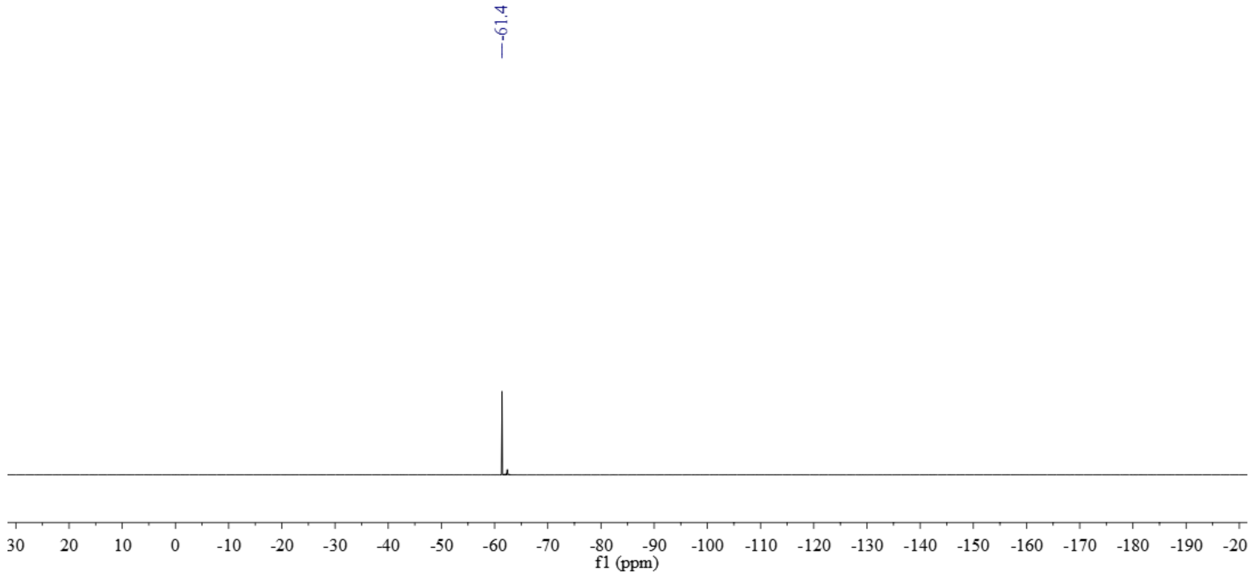

Supplementary Fig. 291. ^{31}F NMR spectrum of **77**. The sample has been recorded in 564 MHz, $CDCl_3$ at 25 °C.

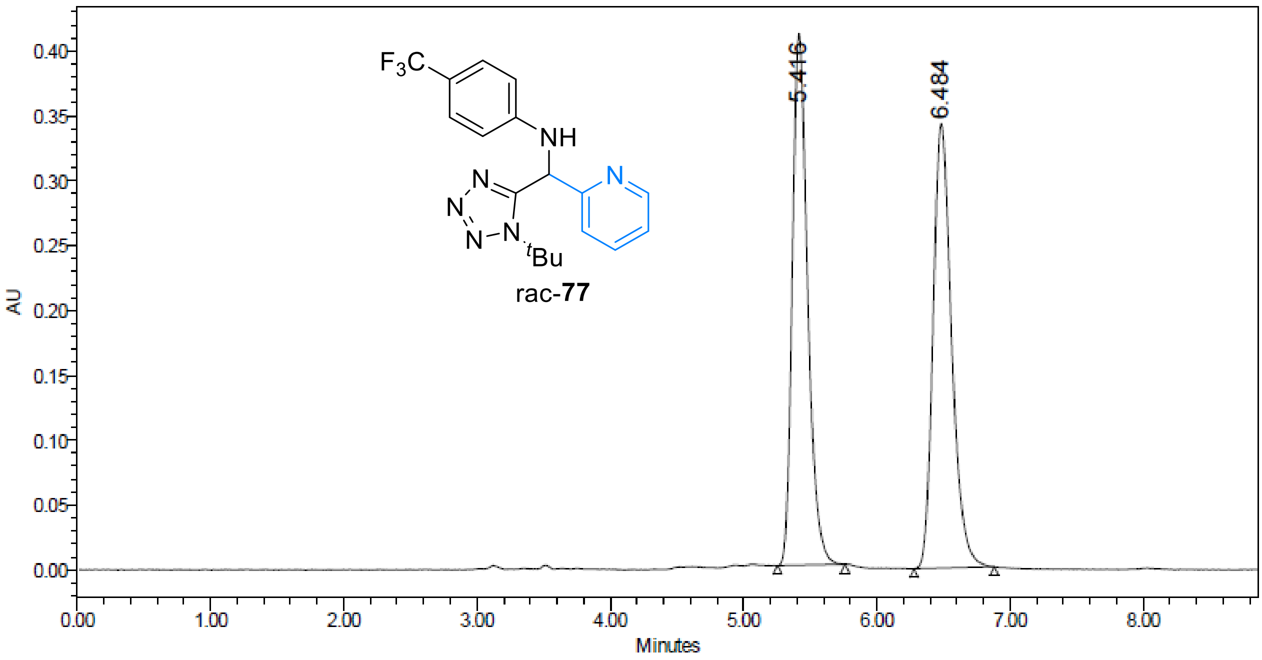

	RT (min)	Area (µV*sec)	% Area	Height (µV)	% Height
1	5.416	3260336	49.55	409807	54.47
2	6.484	3319174	50.45	342566	45.53

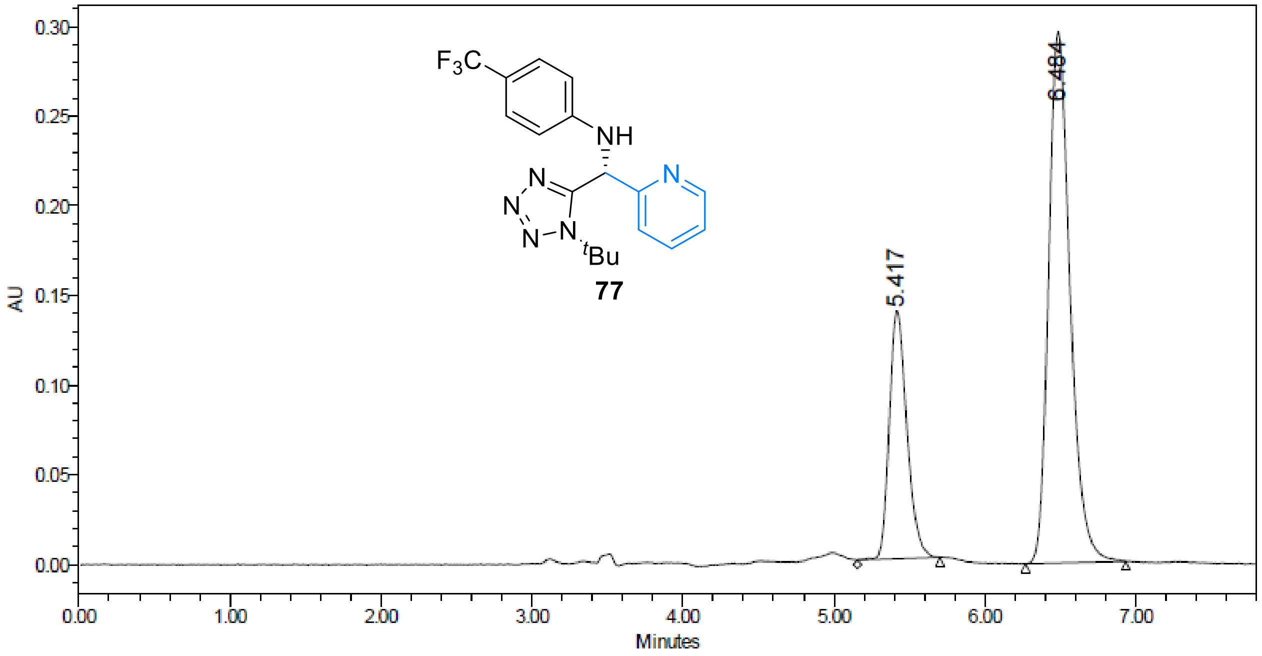

	RT (min)	Area (µV*sec)	% Area	Height (µV)	% Height
1	5.417	1120061	27.72	138018	31.78
2	6.484	2921087	72.28	296304	68.22

Supplementary Fig. 292. HPLC of product **77**.

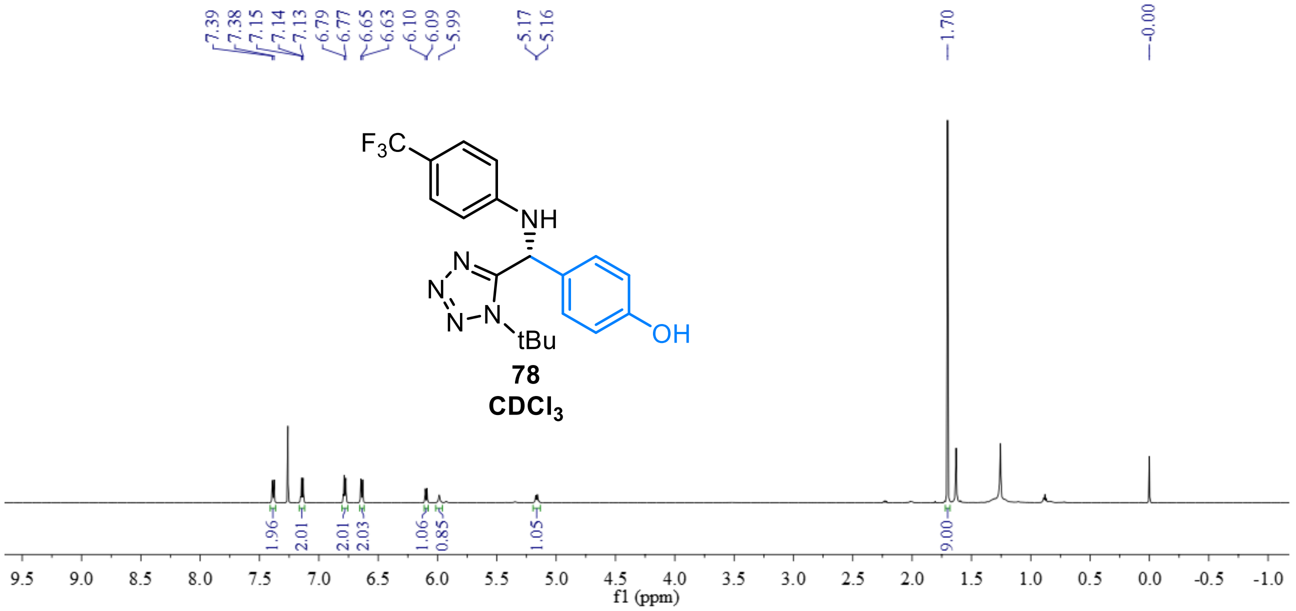

Supplementary Fig. 293. ^{1}H NMR spectrum of **78**. The sample has been recorded in 600 MHz, $CDCl_3$ at 25 °C.

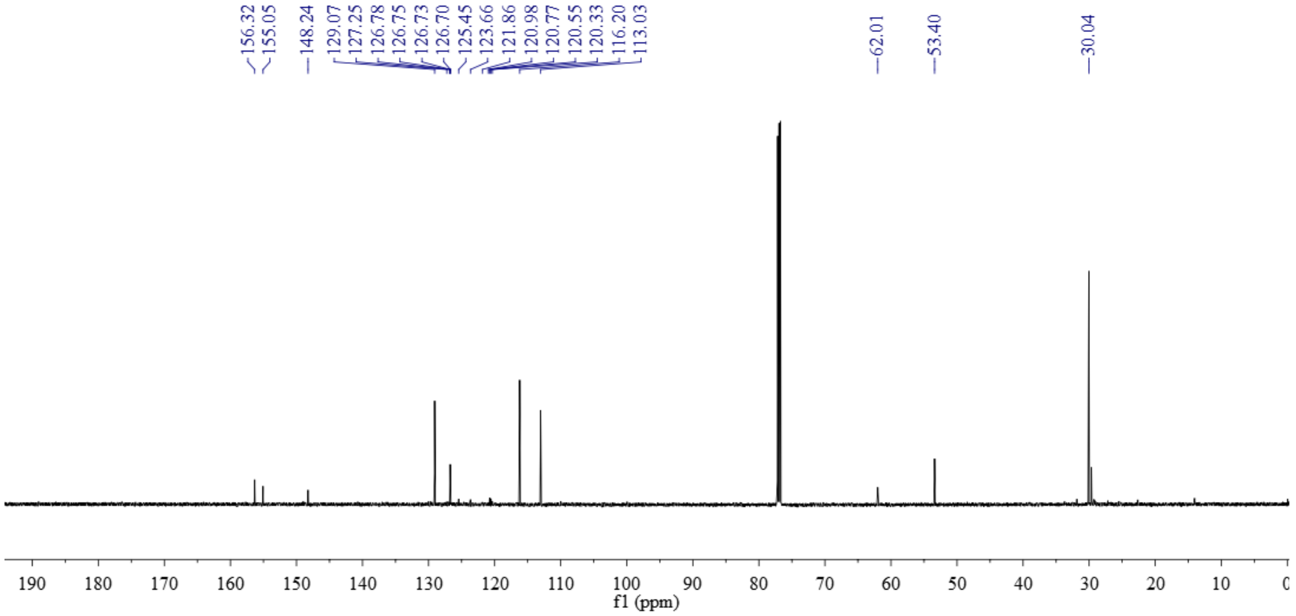

Supplementary Fig. 294. ^{13}C NMR spectrum of **78**. The sample has been recorded in 151 MHz, $CDCl_3$ at 25 °C.

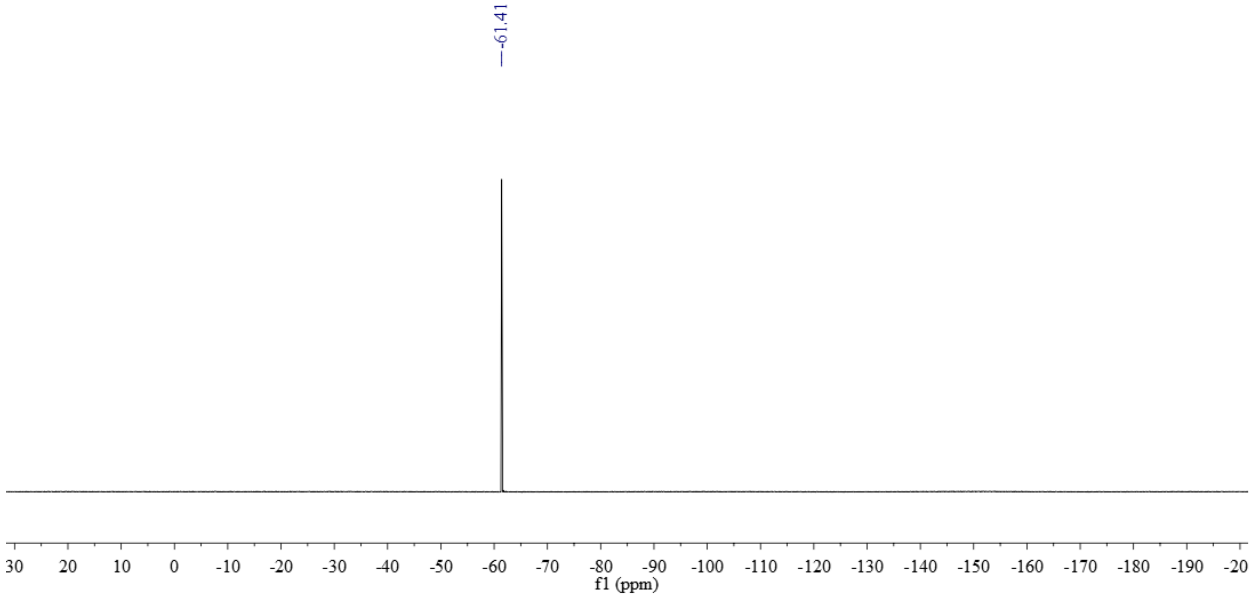

Supplementary Fig. 295. ^{31}F NMR spectrum of **78**. The sample has been recorded in 564 MHz, $CDCl_3$ at 25 °C.

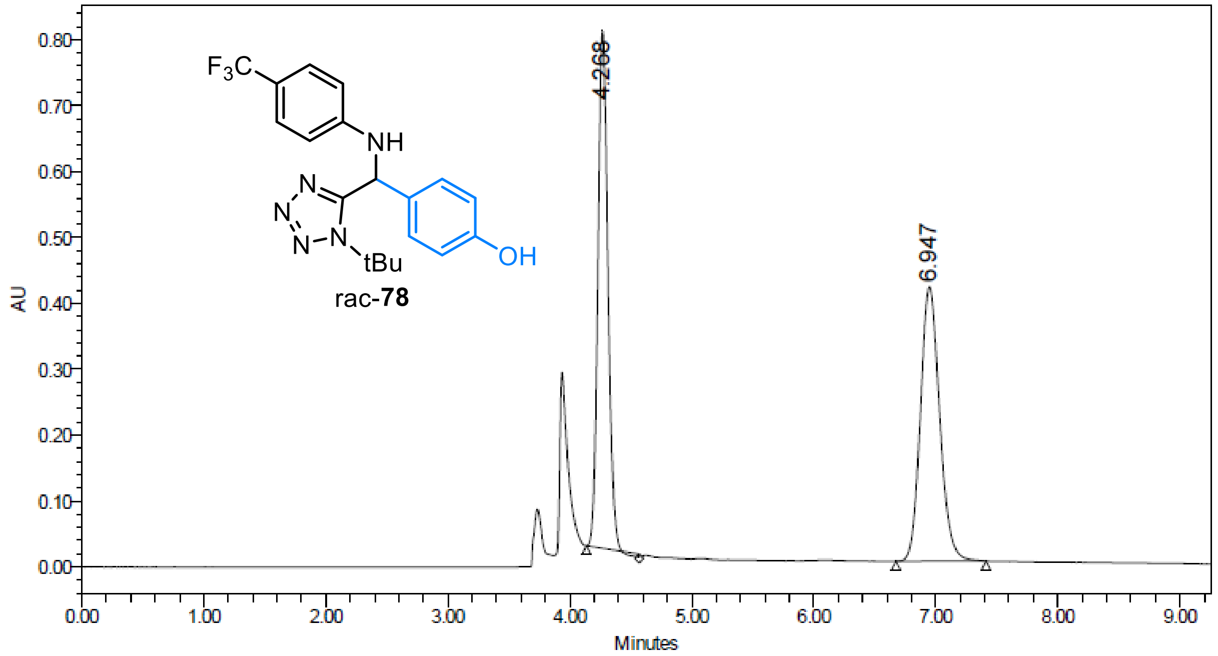

	RT (min)	Peak Type	Area (µV*sec)	% Area	Height (µV)	% Height	Integration Type	Points Across Peak	Start Time (min)	End Time (min)
1	4.268	Unknown	4505317	49.78	784805	65.34	bV	259	4.137	4.568
2	6.947	Unknown	4545541	50.22	416213	34.66	BB	443	6.673	7.412

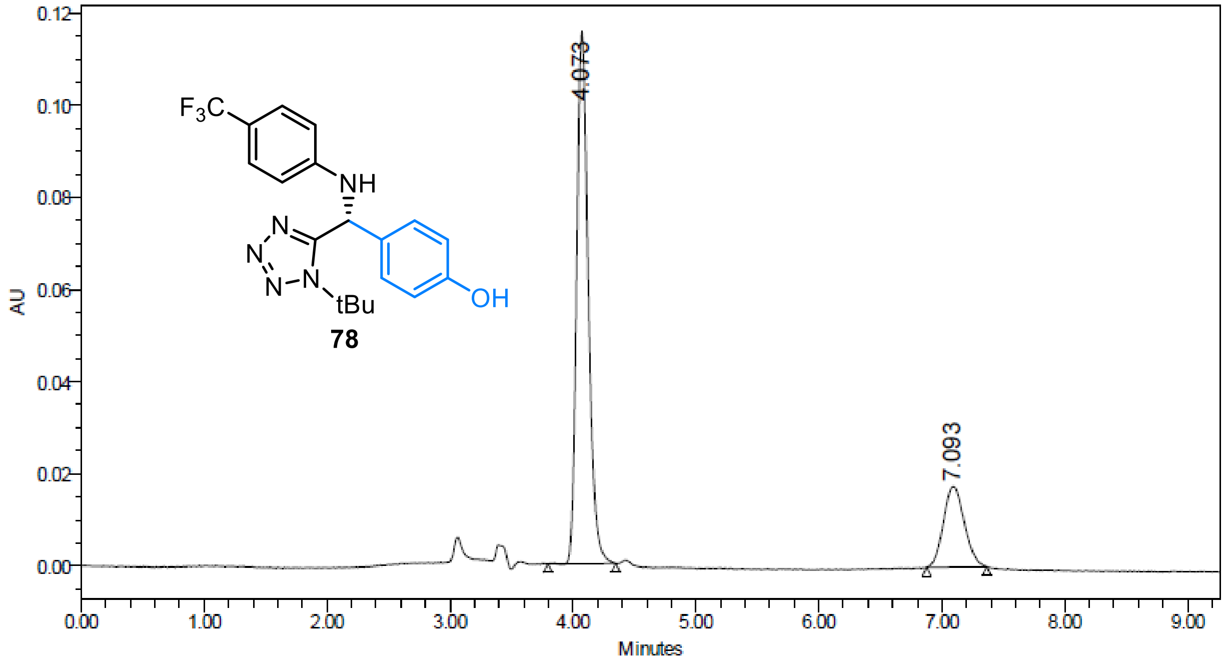

	RT (min)	Peak Type	Area (µV*sec)	% Area	Height (µV)	% Height	Integration Type	Points Across Peak	Start Time (min)	End Time (min)
1	4.073	Unknown	756039	78.50	115499	86.81	bB	331	3.797	4.348
2	7.093	Unknown	207078	21.50	17544	13.19	BB	292	6.878	7.365

Supplementary Fig. 296. HPLC of product **78**.

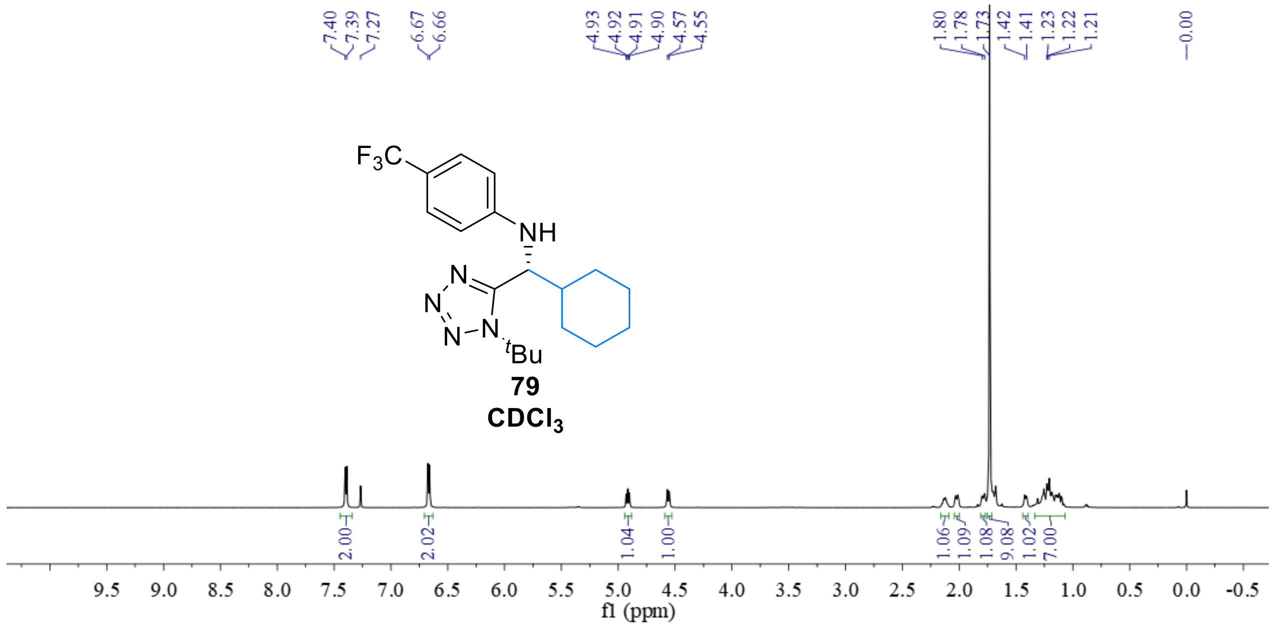

Supplementary Fig. 297. ^{1}H NMR spectrum of **79**. The sample has been recorded in 600 MHz, $CDCl_3$ at 25 °C.

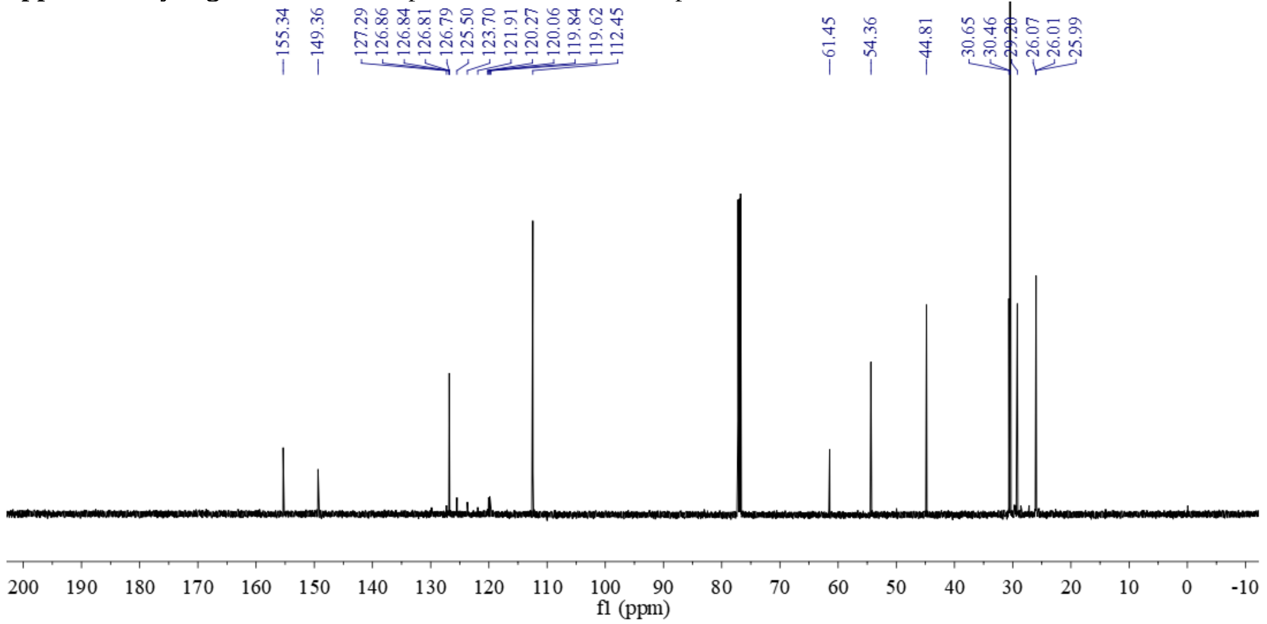

Supplementary Fig. 298. ^{13}C NMR spectrum of **79**. The sample has been recorded in 151 MHz, $CDCl_3$ at 25 °C.

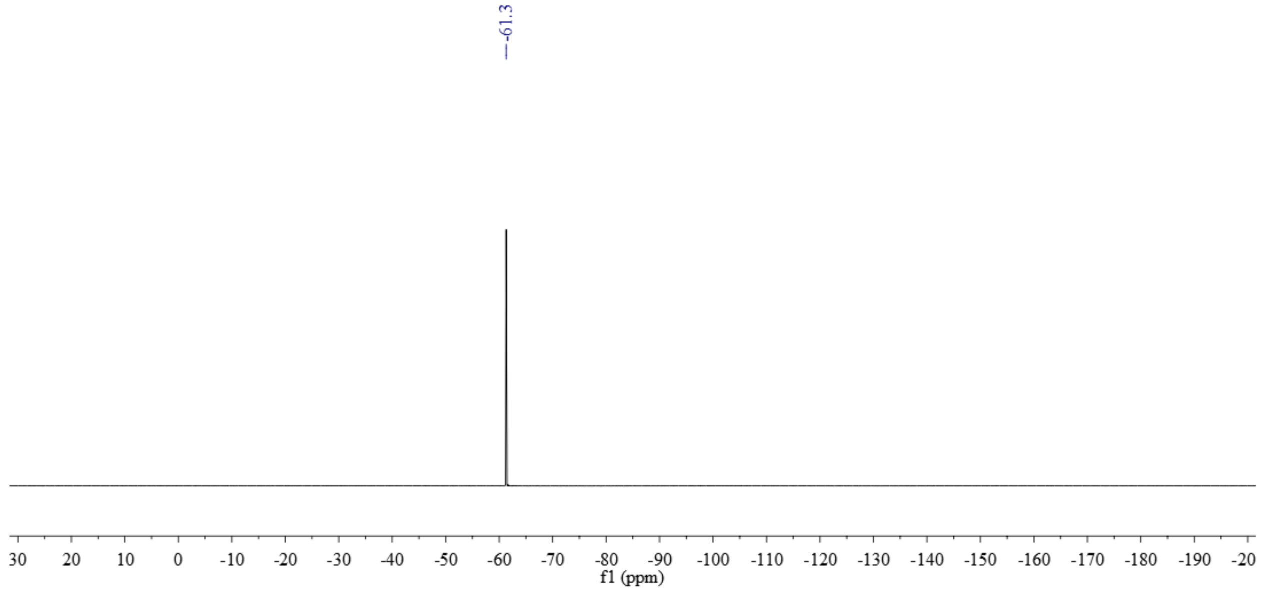

Supplementary Fig. 299. ^{31}F NMR spectrum of **79**. The sample has been recorded in 564 MHz, $CDCl_3$ at 25 °C.

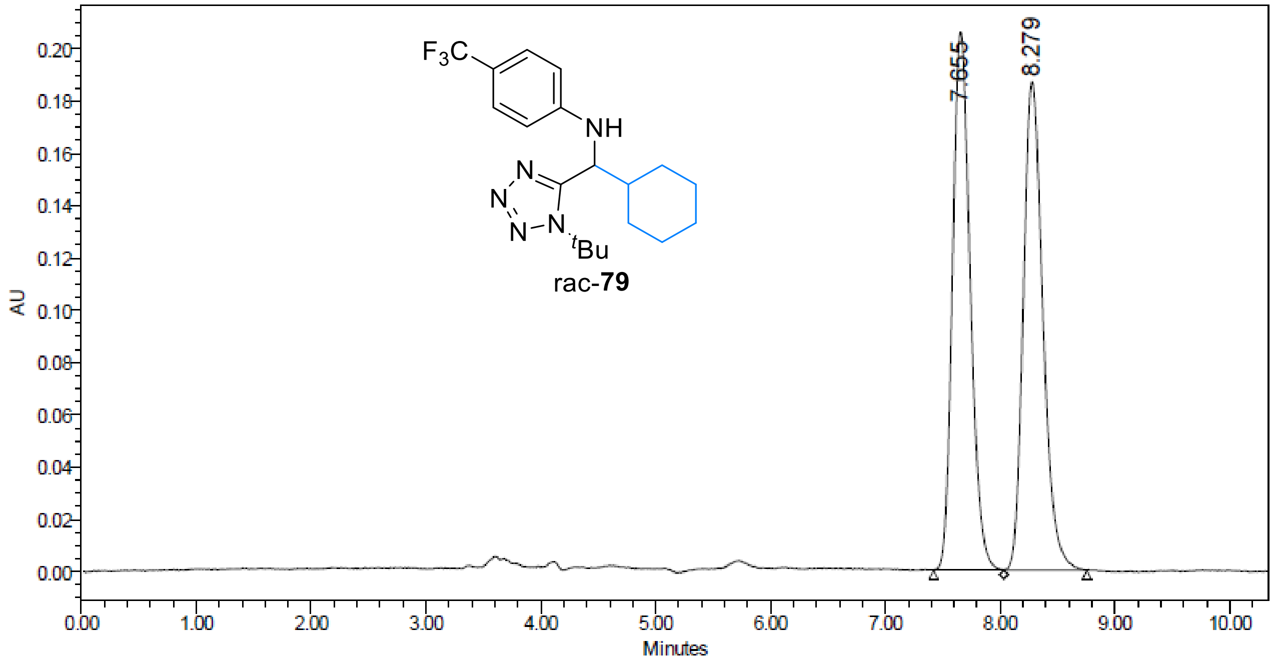

	RT (min)	Area (µV*sec)	% Area	Height (µV)	% Height
1	7.655	2203396	49.82	205924	52.40
2	8.279	2218897	50.18	187039	47.60

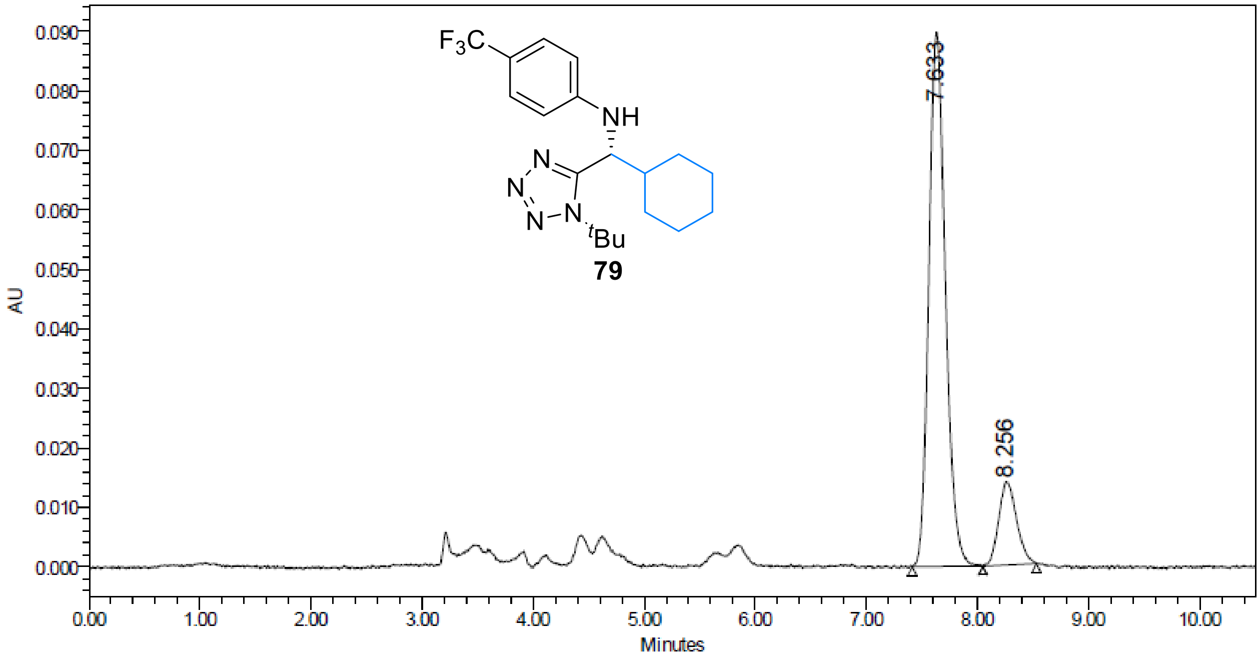

	RT (min)	Area (µV*sec)	% Area	Height (µV)	% Height
1	7.633	931280	85.56	89821	86.57
2	8.256	157209	14.44	13938	13.43

Supplementary Fig. 300. HPLC of product **79**.

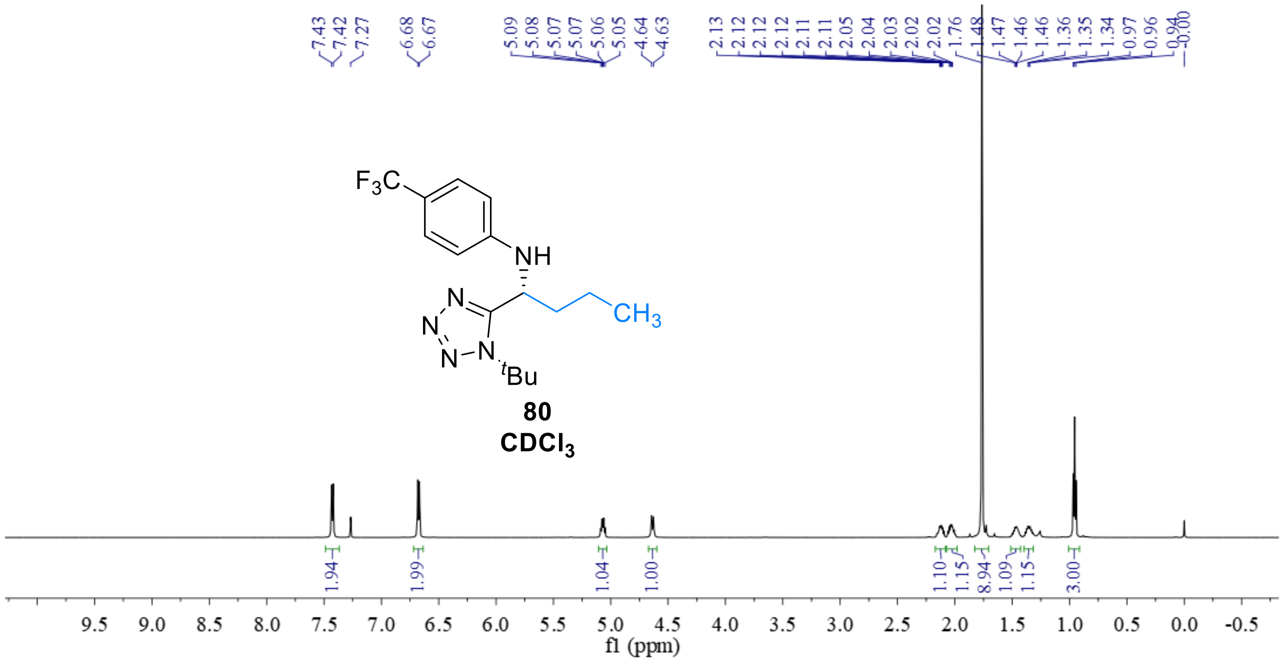

Supplementary Fig. 301. ^{1}H NMR spectrum of **80**. The sample has been recorded in 600 MHz, $CDCl_3$ at 25 °C.

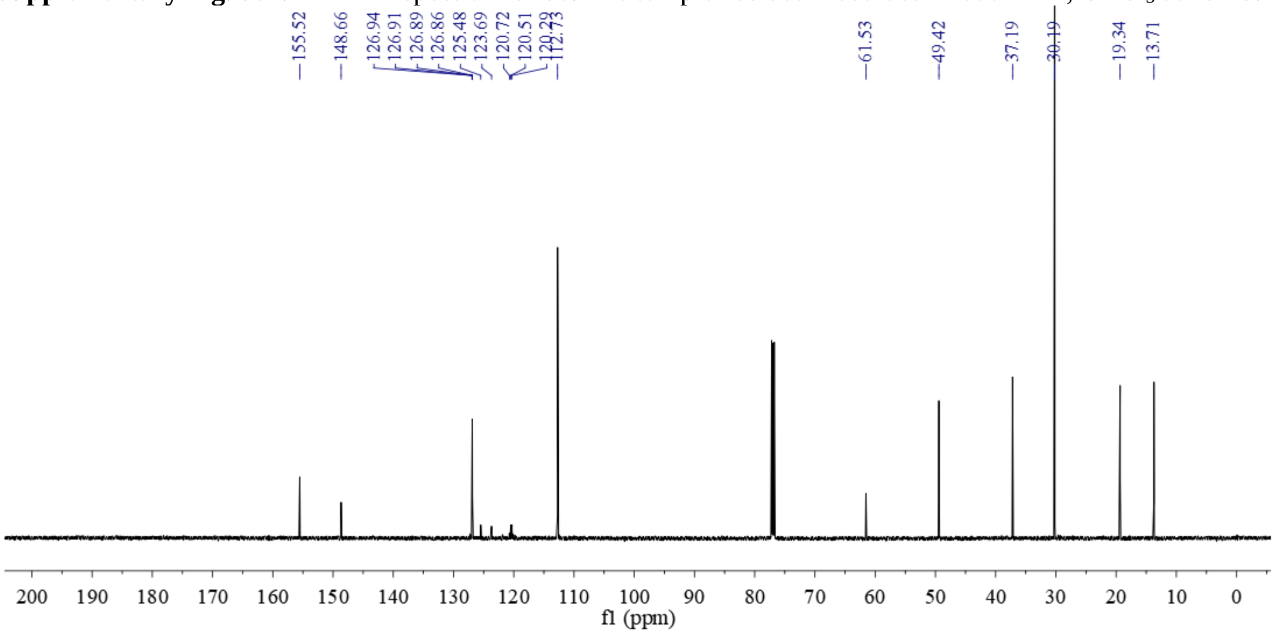

Supplementary Fig. 302. ^{13}C NMR spectrum of **80**. The sample has been recorded in 151 MHz, $CDCl_3$ at 25 °C.

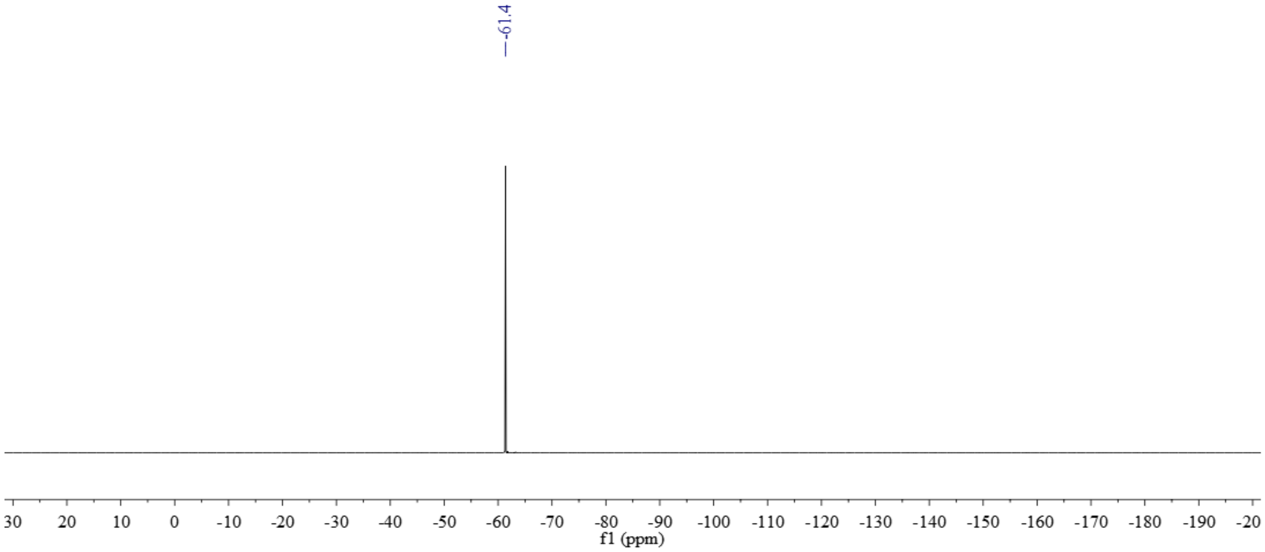

Supplementary Fig. 303. ^{31}F NMR spectrum of **80**. The sample has been recorded in 564 MHz, $CDCl_3$ at 25 °C.

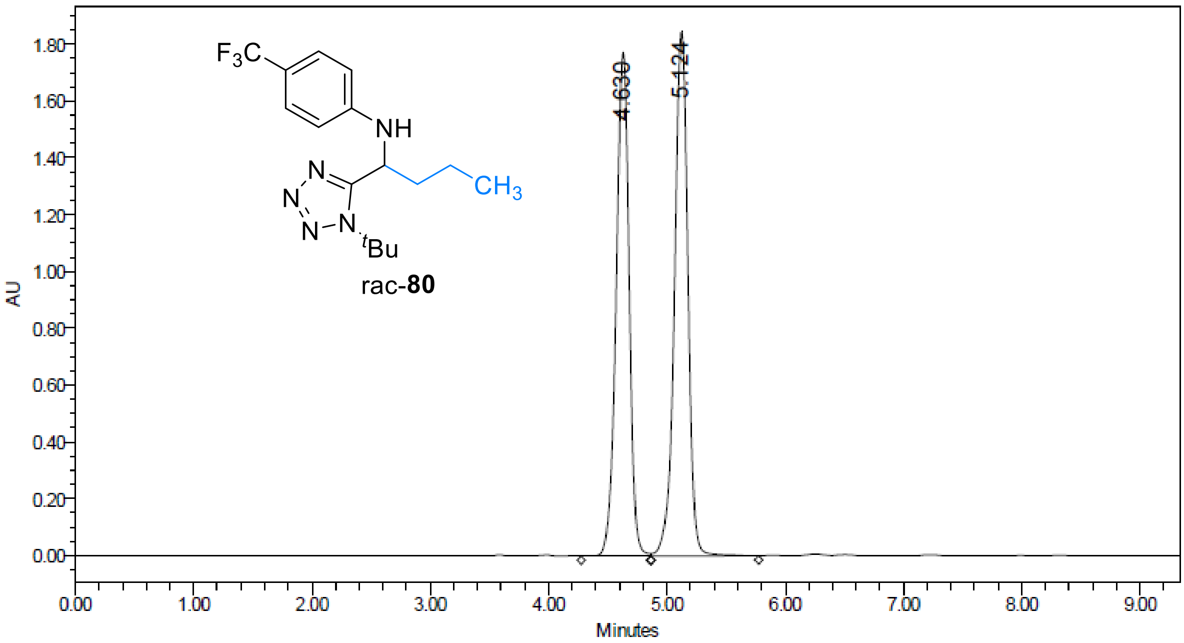

	RT (min)	Peak Type	Area (µV*sec)	% Area	Height (µV)	% Height	Integration Type	Points Across Peak	Start Time (min)	End Time (min)
1	4.630	Unknown	13485430	48.05	1771978	49.00	VV	353	4.277	4.865
2	5.124	Unknown	14580720	51.95	1844056	51.00	VV	547	4.865	5.777

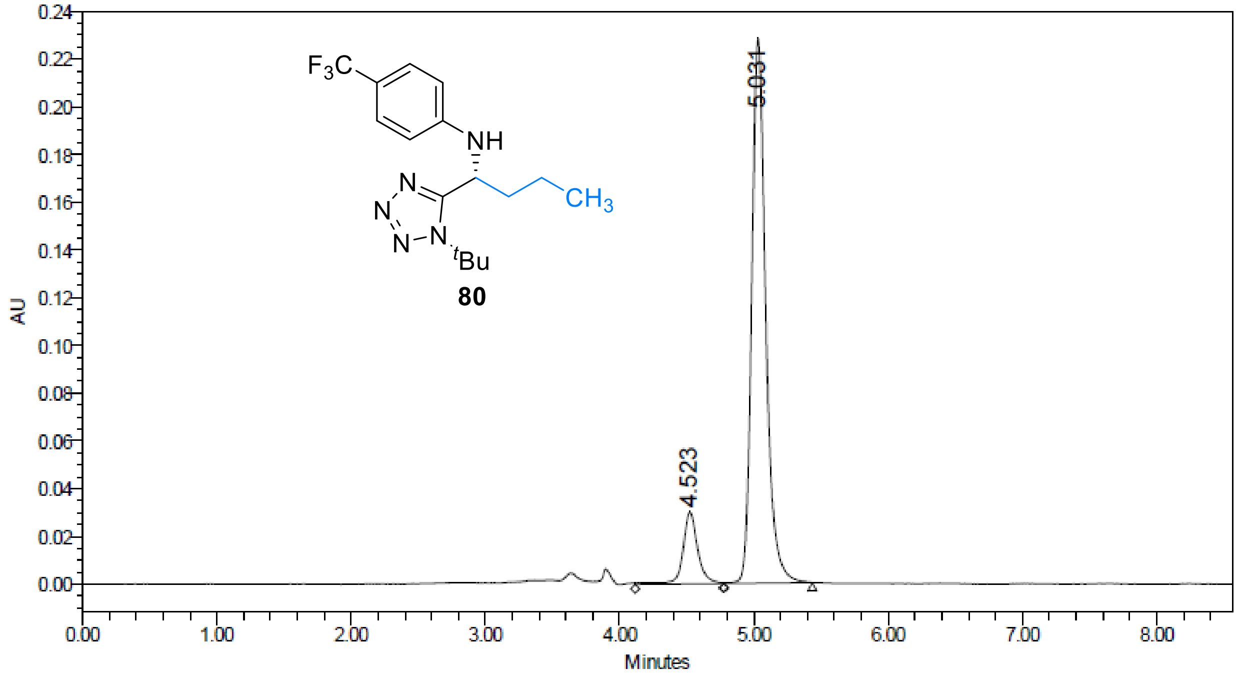

	RT (min)	Peak Type	Area (µV*sec)	% Area	Height (µV)	% Height	Integration Type	Points Across Peak	Start Time (min)	End Time (min)
1	4.523	Unknown	232303	12.36	30064	11.65	VV	395	4.117	4.775
2	5.031	Unknown	1647779	87.64	228105	88.35	VB	396	4.775	5.435

Supplementary Fig. 304. HPLC of product **80**.

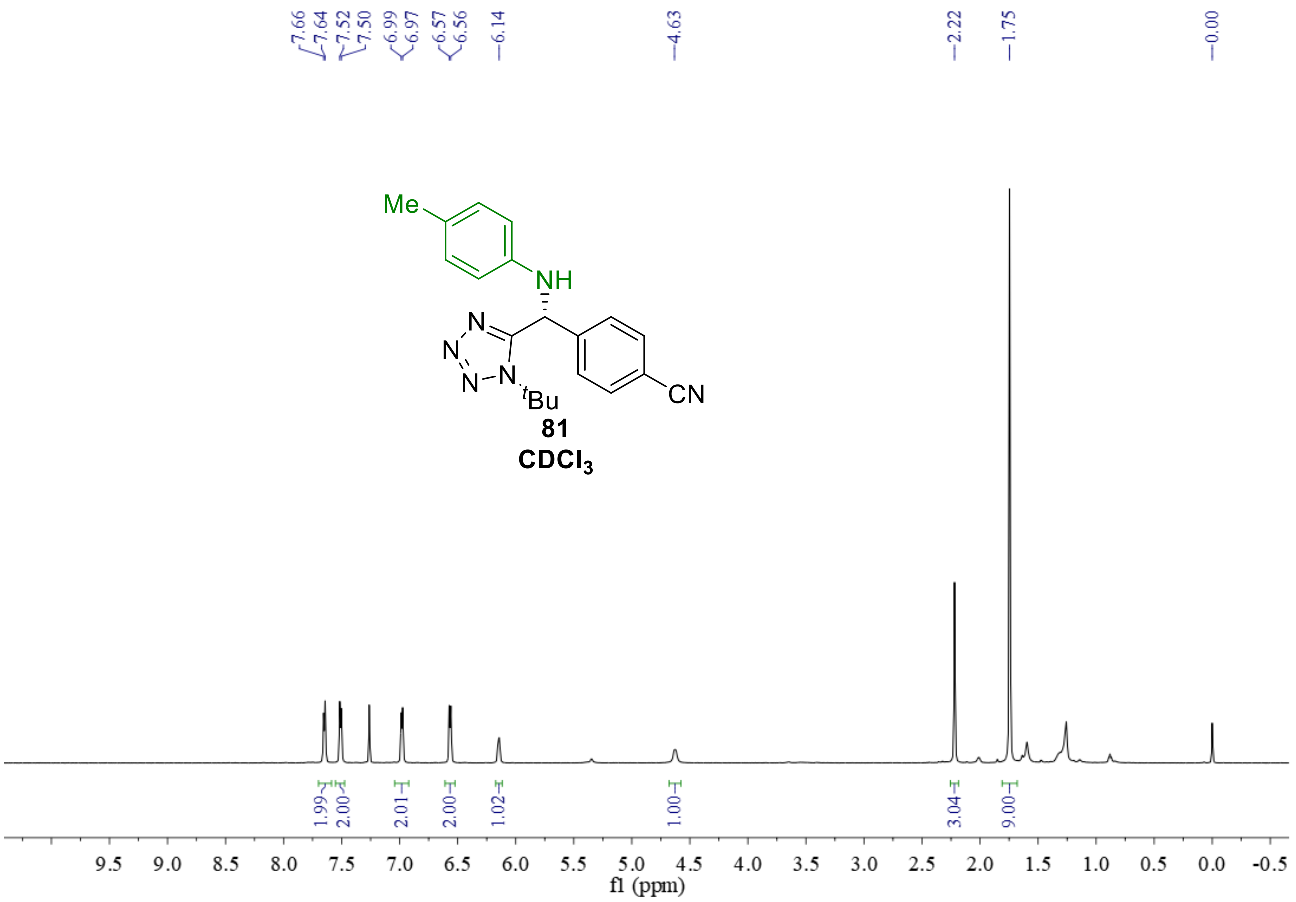

Supplementary Fig. 305. ^{1}H NMR spectrum of **81**. The sample has been recorded in 600 MHz, $CDCl_3$ at 25 °C.

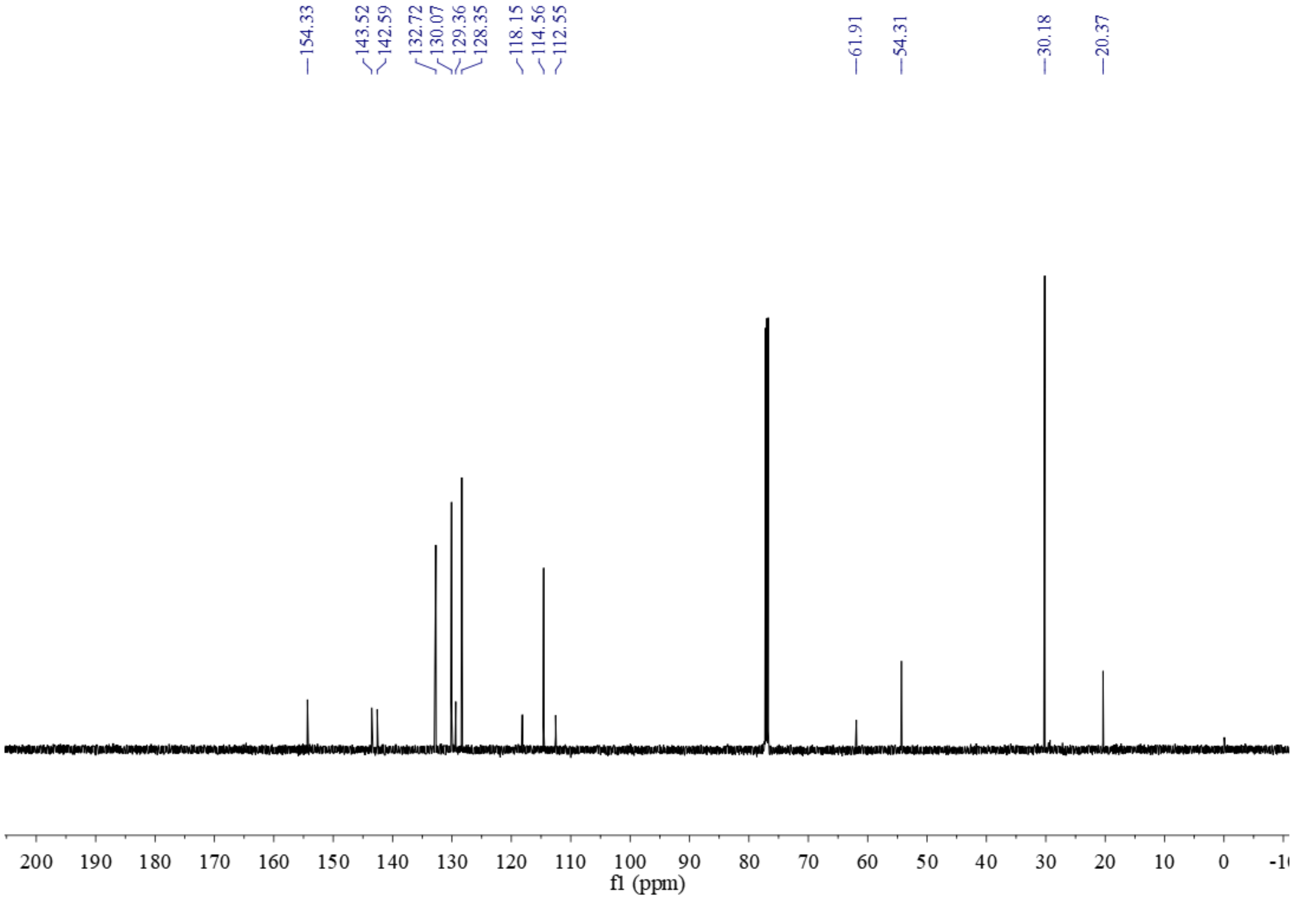

Supplementary Fig. 306. ^{13}C NMR spectrum of **81**. The sample has been recorded in 151 MHz, $CDCl_3$ at 25 °C.

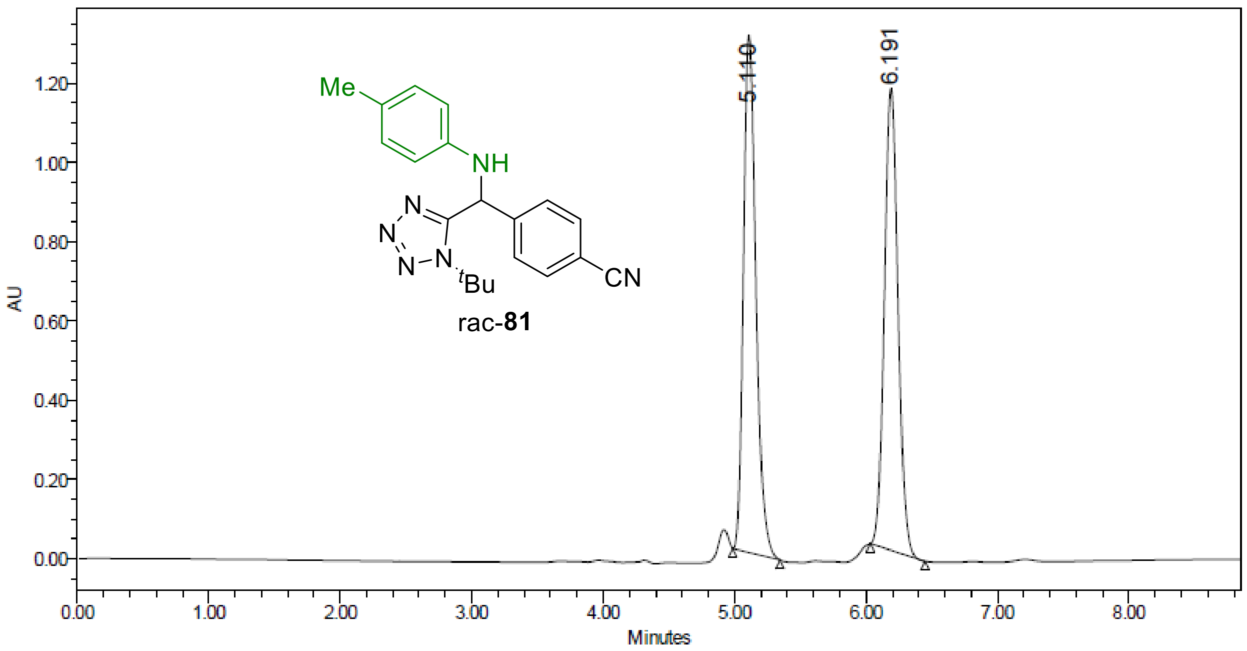

	RT (min)	Area (µV*sec)	% Area	Height (µV)	% Height
1	5.110	8578109	50.55	1306887	52.87
2	6.191	8392192	49.45	1165041	47.13

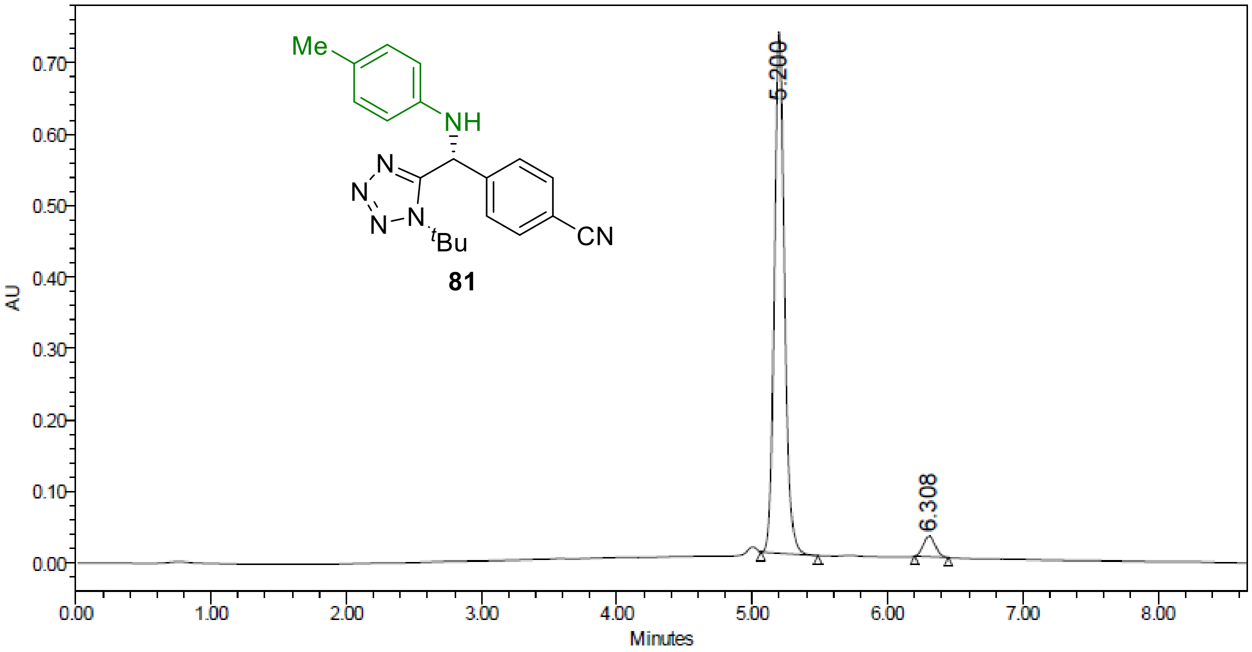

	RT (min)	Area (µV*sec)	% Area	Height (µV)	% Height
1	5.200	3779724	95.49	729503	96.23
2	6.308	178488	4.51	28617	3.77

Supplementary Fig. 307. HPLC of product **81**.

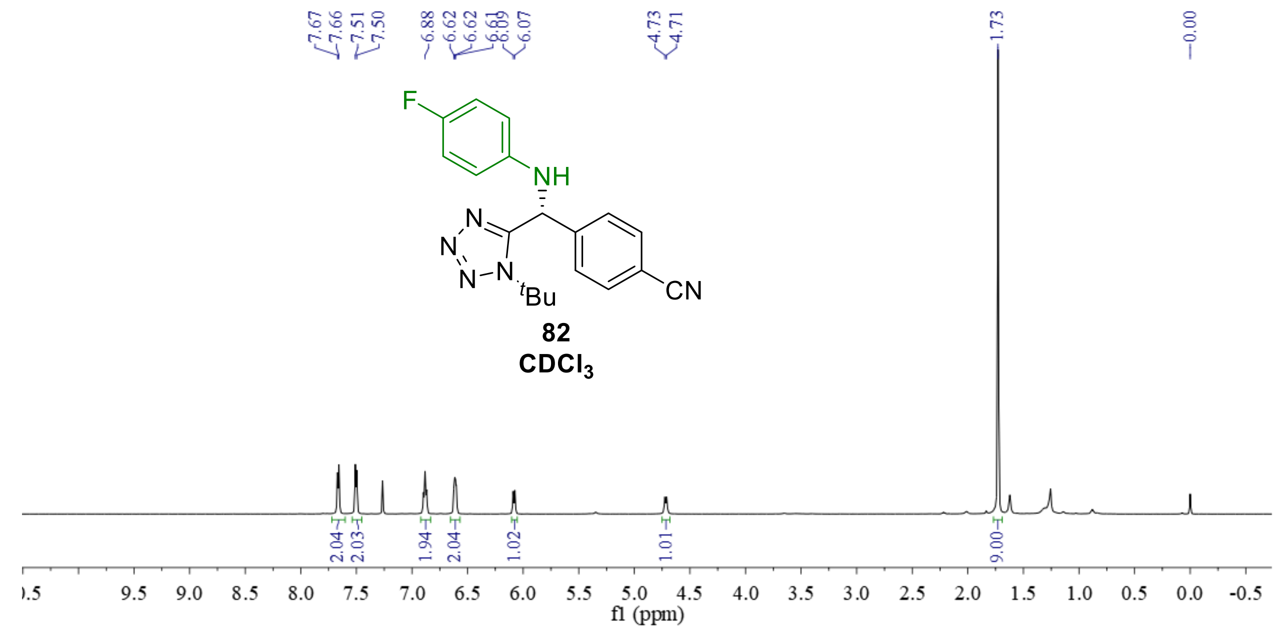

Supplementary Fig. 308. ^{1}H NMR spectrum of **82**. The sample has been recorded in 600 MHz, $CDCl_3$ at 25 °C.

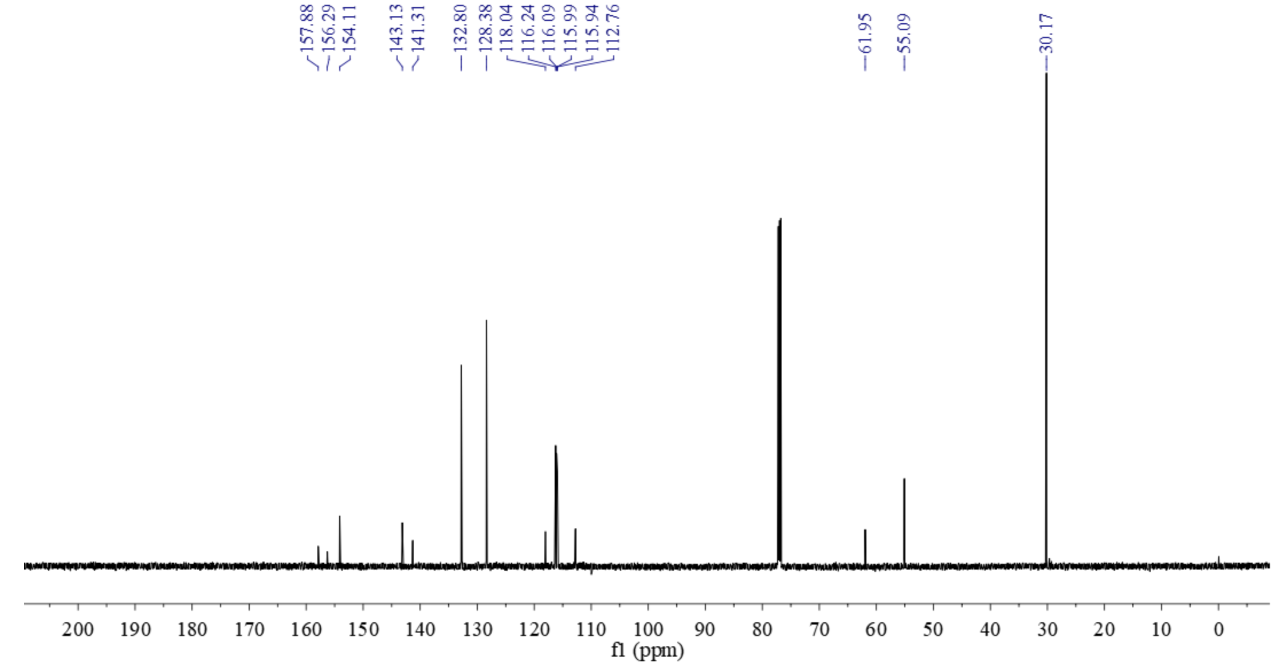

Supplementary Fig. 309. ^{13}C NMR spectrum of **82**. The sample has been recorded in 151 MHz, $CDCl_3$ at 25 °C.

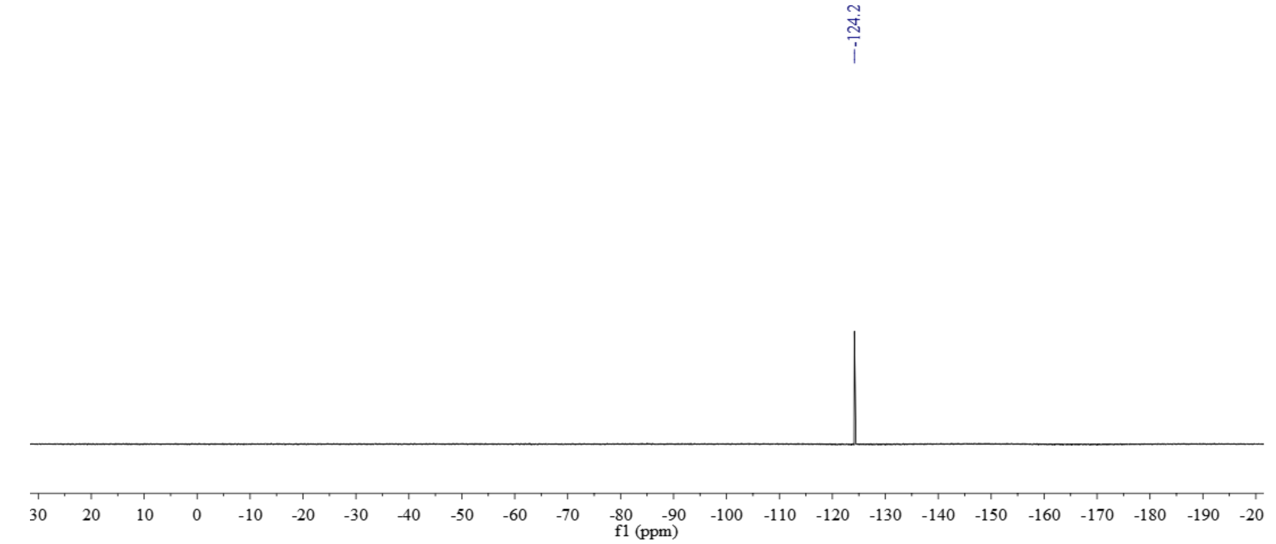

Supplementary Fig. 310. ^{31}F NMR spectrum of **82**. The sample has been recorded in 564 MHz, $CDCl_3$ at 25 °C.

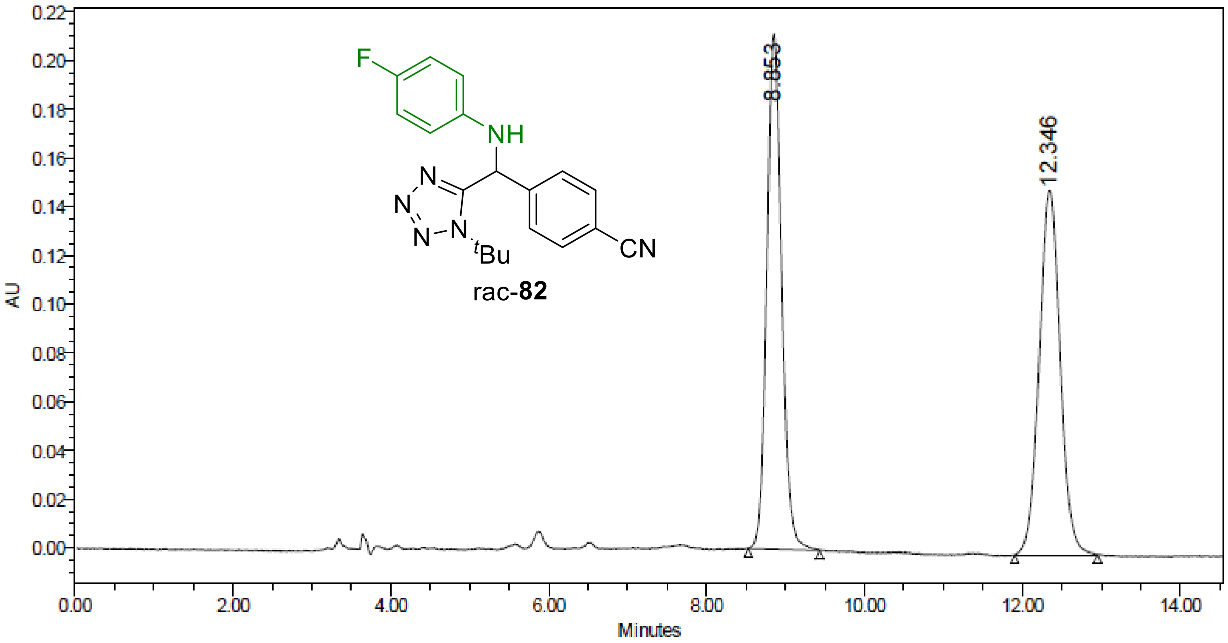

	RT (min)	Area (µV*sec)	% Area	Height (µV)	% Height
1	8.853	2768358	50.02	211309	58.57
2	12.346	2766350	49.98	149488	41.43

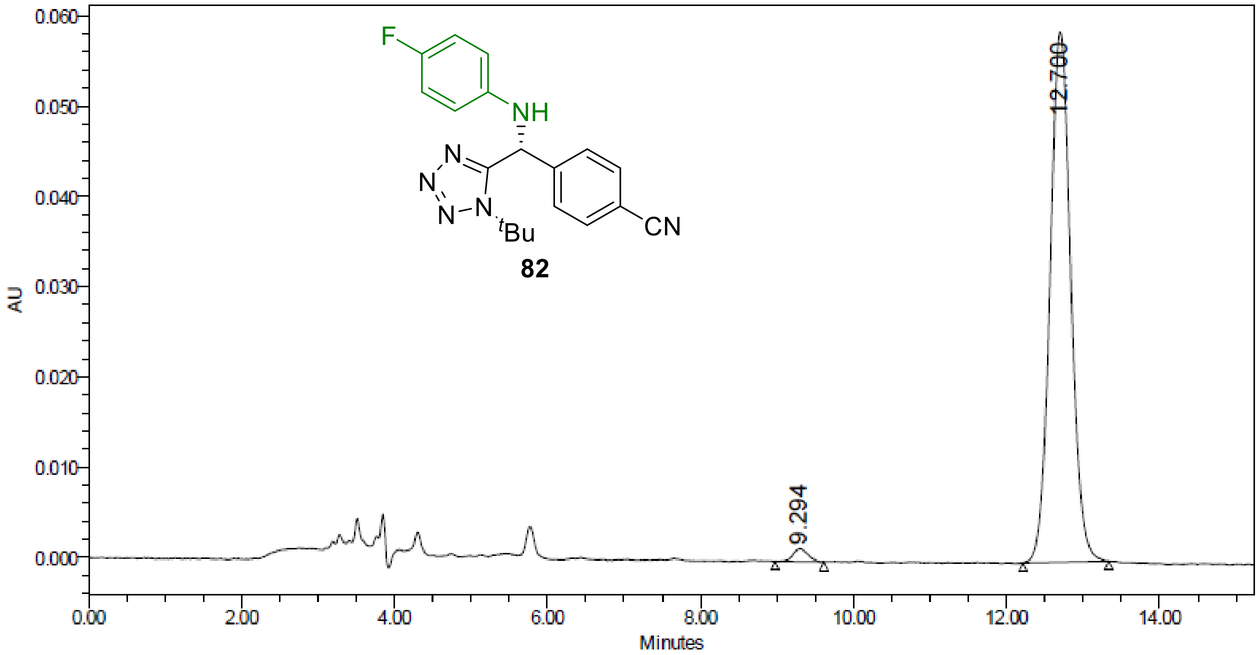

	RT (min)	Area (µV*sec)	% Area	Height (µV)	% Height
1	9.294	20291	1.80	1482	2.46
2	12.700	1107373	98.20	58778	97.54

Supplementary Fig. 311. HPLC of product **82**.

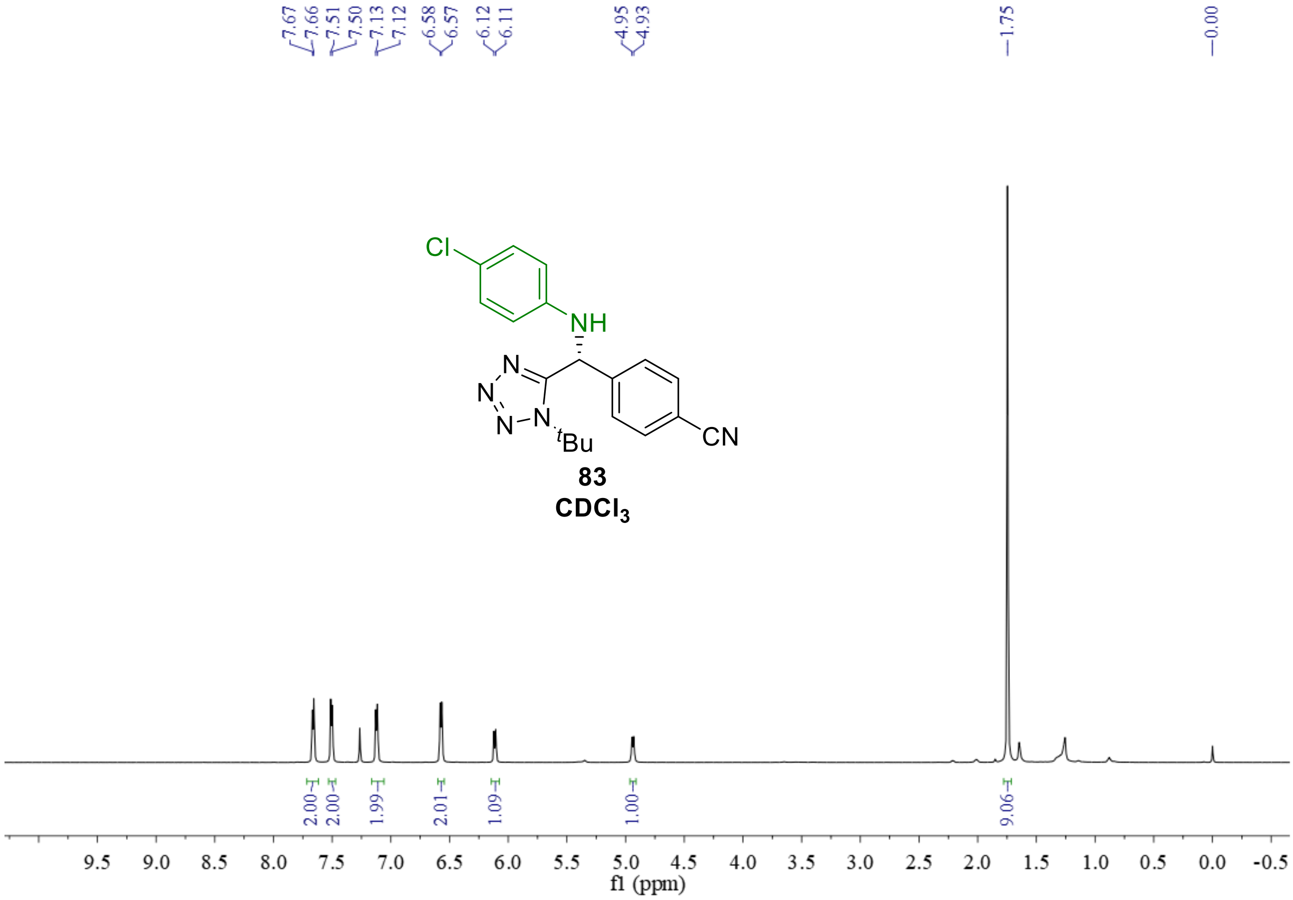

Supplementary Fig. 312. ^{1}H NMR spectrum of **83**. The sample has been recorded in 600 MHz, $CDCl_3$ at 25 °C.

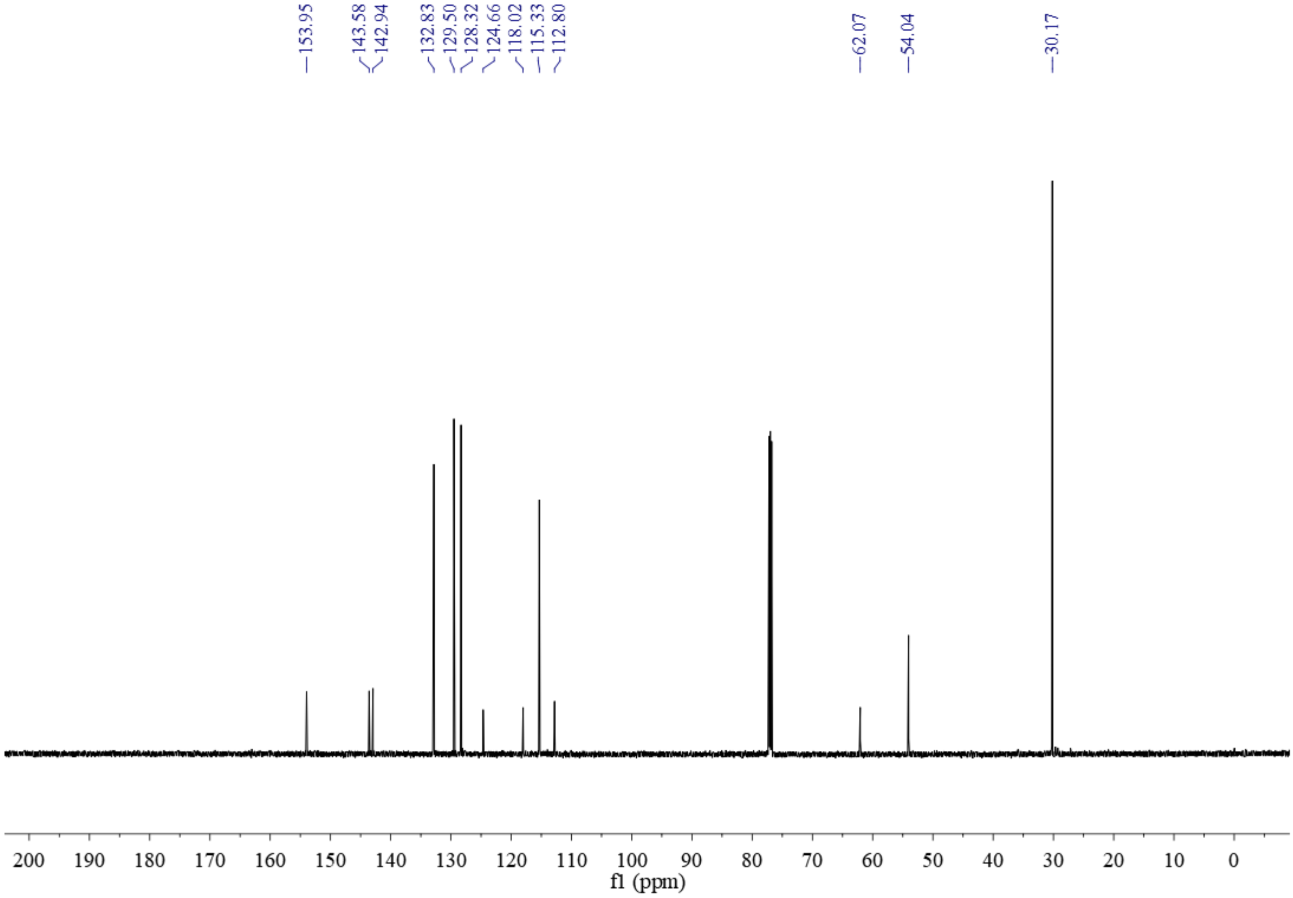

Supplementary Fig. 313. ^{13}C NMR spectrum of **83**. The sample has been recorded in 151 MHz, $CDCl_3$ at 25 °C.

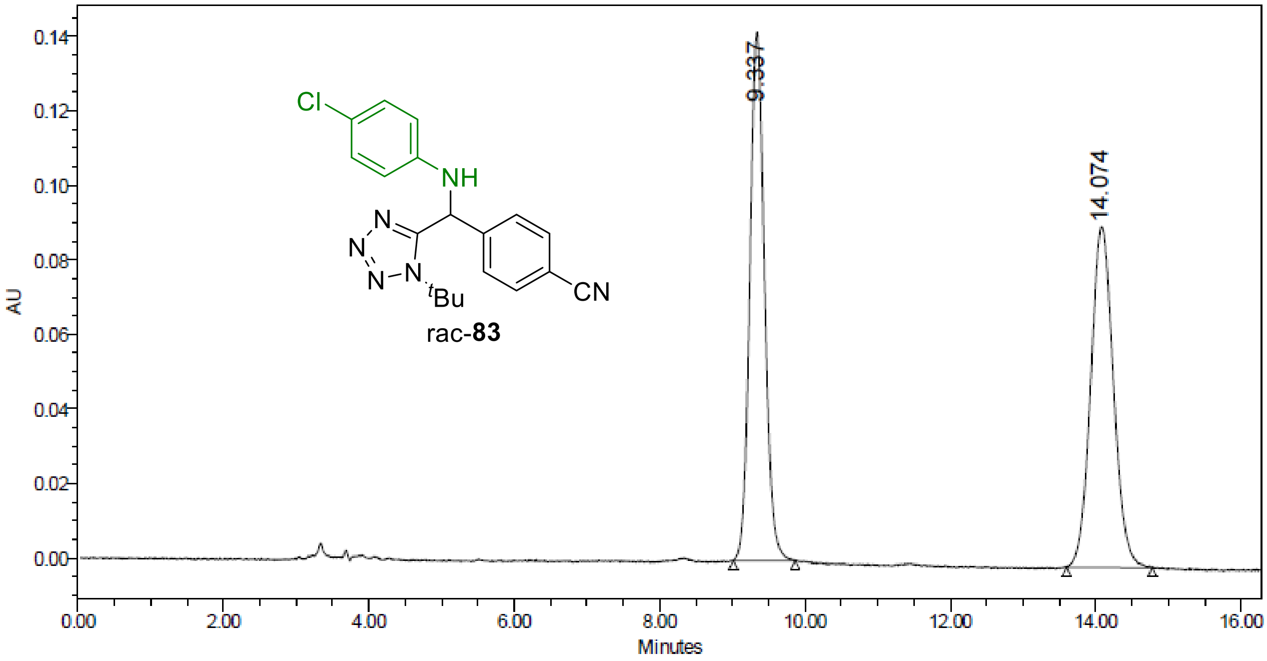

	RT (min)	Area (µV*sec)	% Area	Height (µV)	% Height
1	9.337	1986921	49.97	141768	60.79
2	14.074	1988998	50.03	91431	39.21

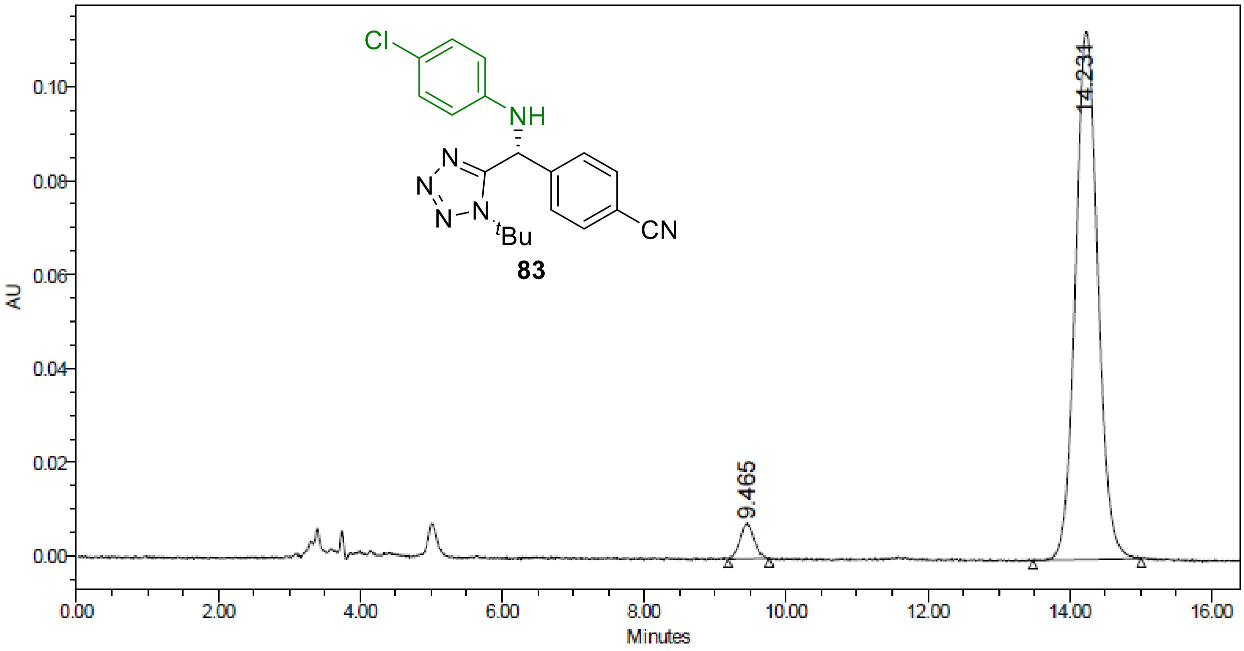

	RT (min)	Area (µV*sec)	% Area	Height (µV)	% Height
1	9.465	103201	4.00	7495	6.24
2	14.231	2475446	96.00	112559	93.76

Supplementary Fig. 314. HPLC of product **83**.

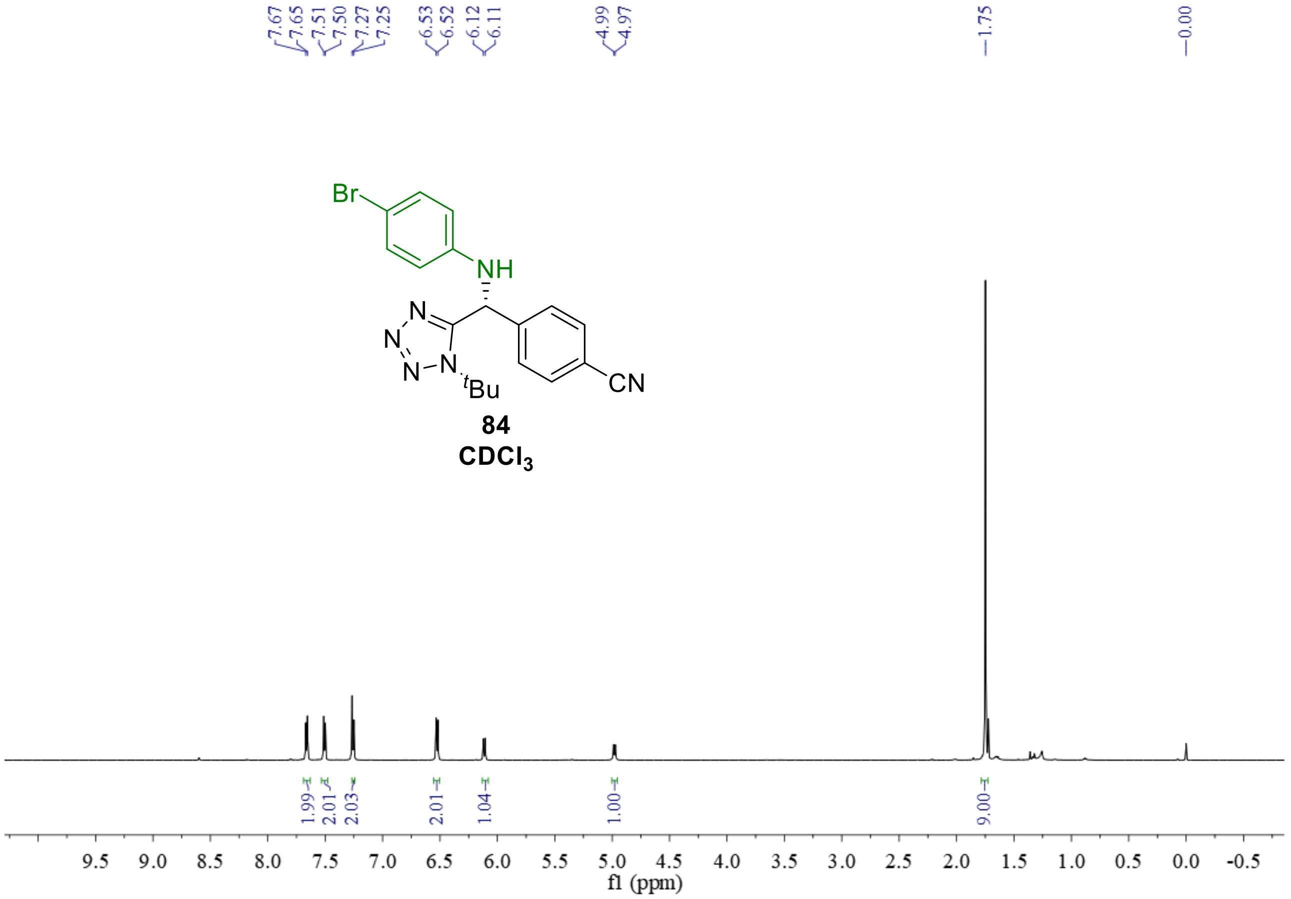

Supplementary Fig. 315. ^{1}H NMR spectrum of **84**. The sample has been recorded in 600 MHz, $CDCl_3$ at 25 °C.

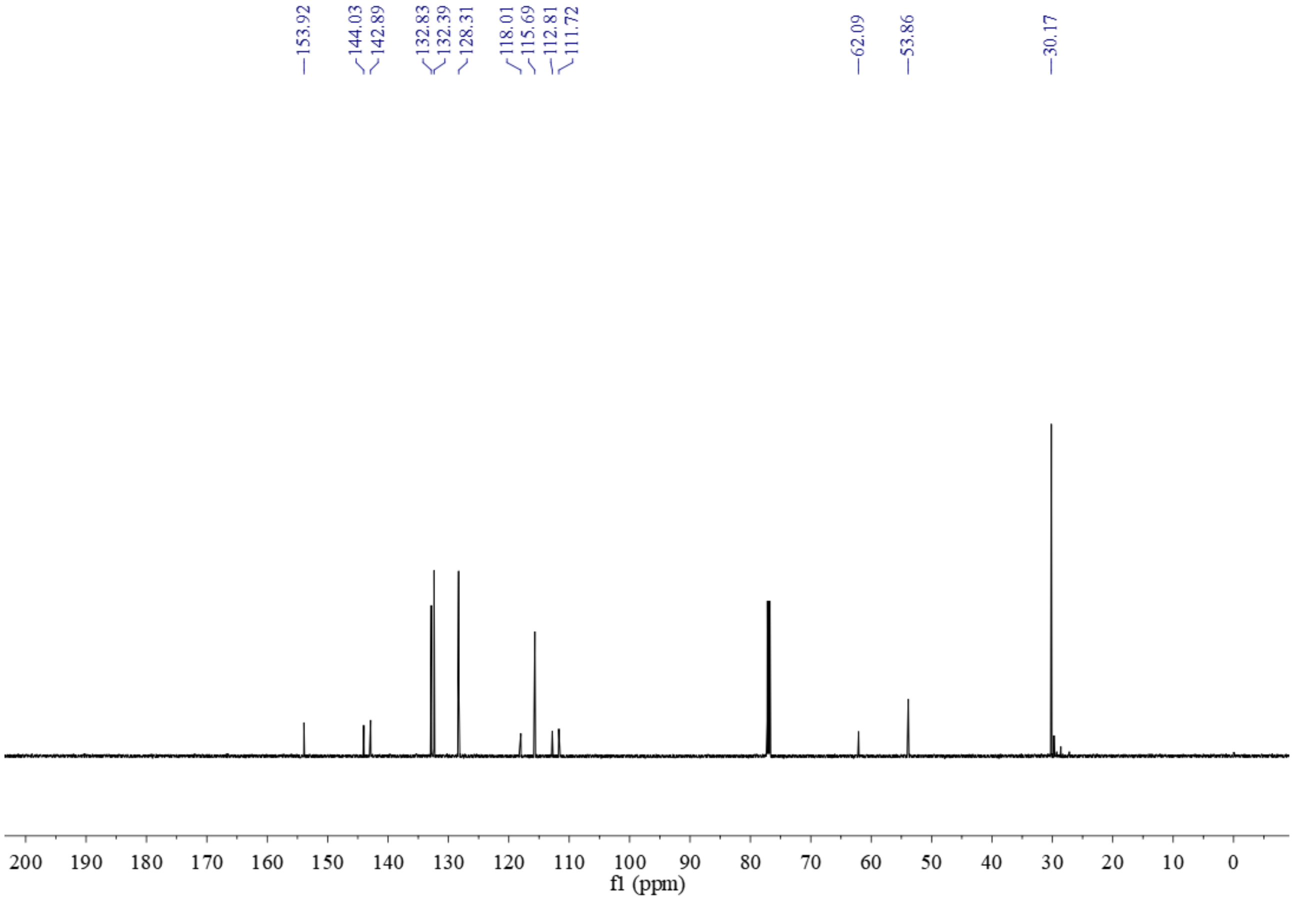

Supplementary Fig. 316. ^{13}C NMR spectrum of **84**. The sample has been recorded in 151 MHz, $CDCl_3$ at 25 °C.

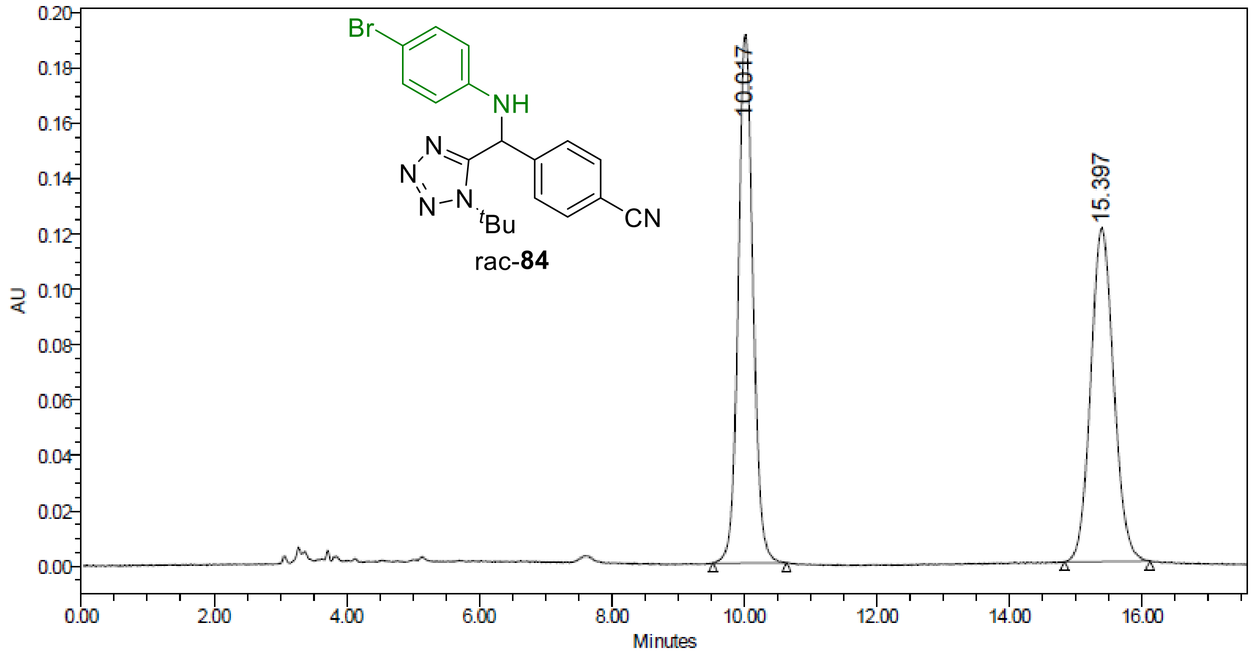

	RT (min)	Area (μV*sec)	% Area	Height (μV)	% Height
1	10.017	2971816	50.50	190995	61.32
2	15.397	2913266	49.50	120495	38.68

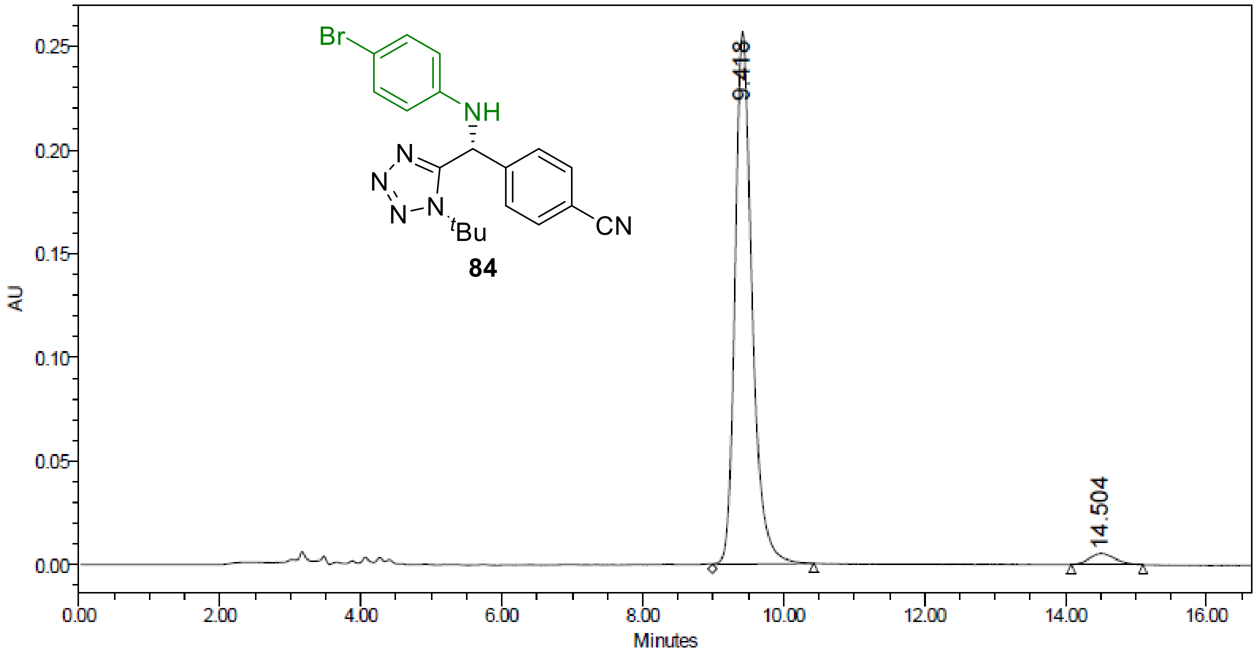

	RT (min)	Area (μV*sec)	% Area	Height (μV)	% Height
1	9.418	4325887	96.97	256799	97.93
2	14.504	135254	3.03	5435	2.07

Supplementary Fig. 317. HPLC of product **84**.

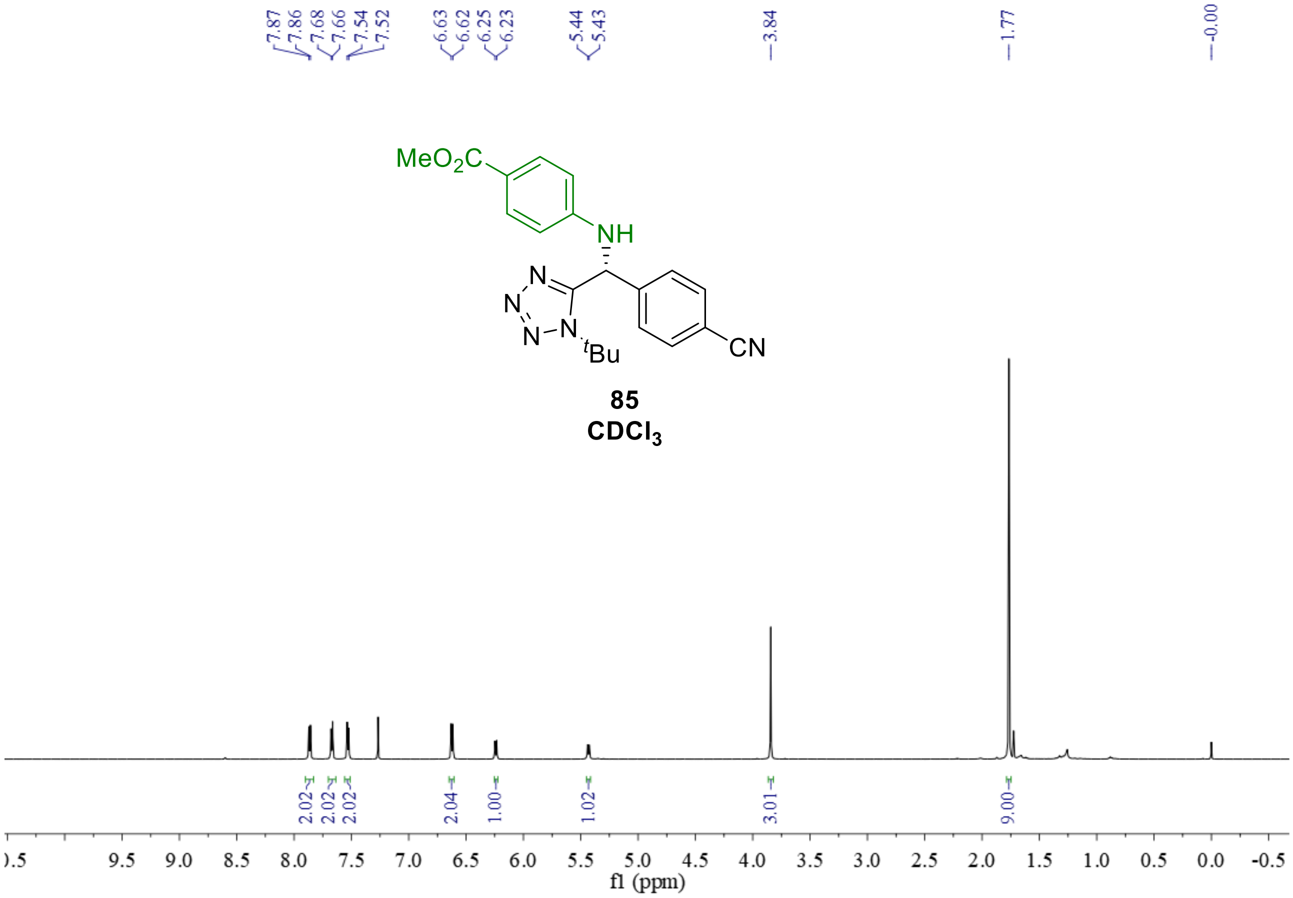

Supplementary Fig. 318. ^{1}H NMR spectrum of **85**. The sample has been recorded in 600 MHz, $CDCl_3$ at 25 °C.

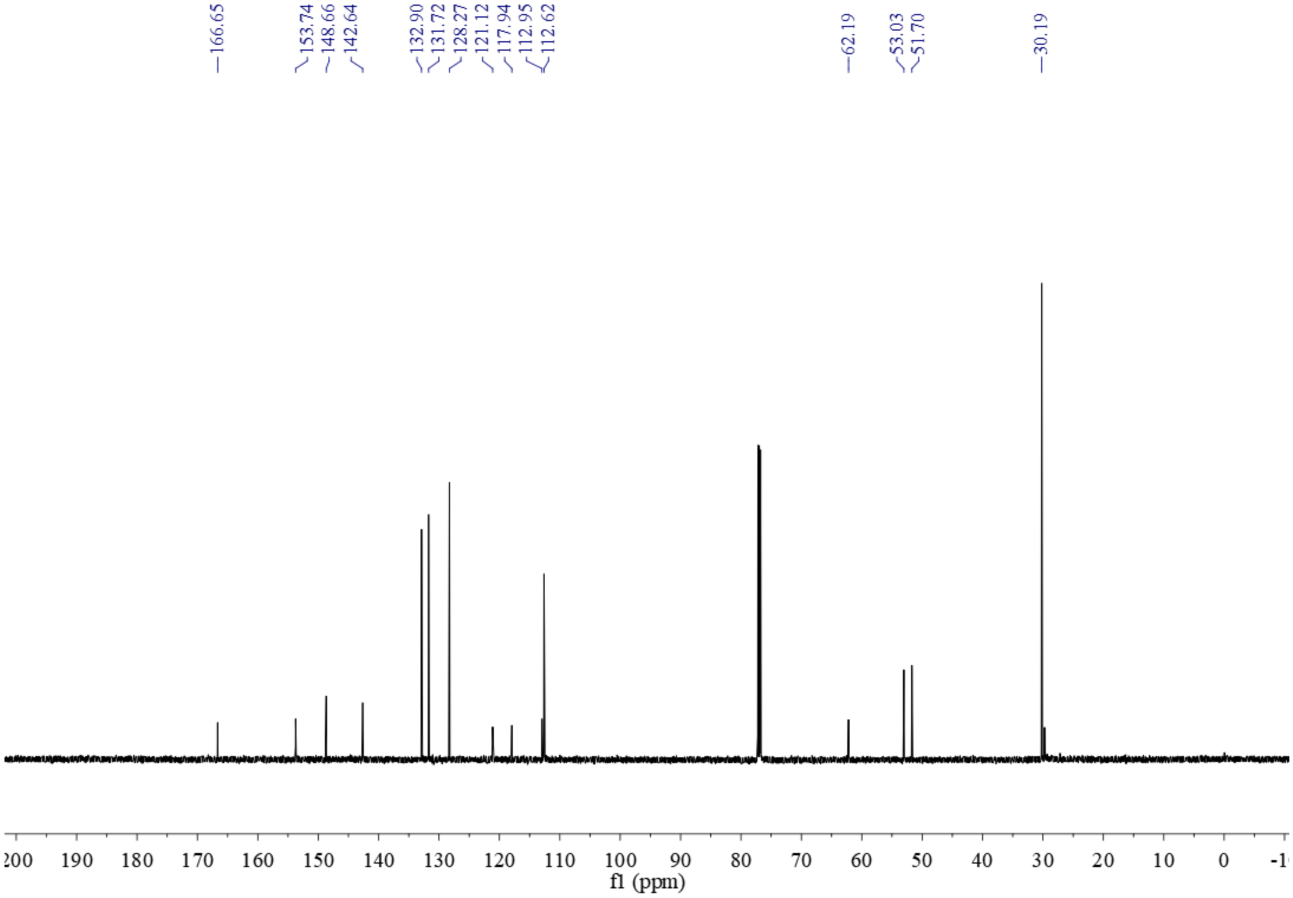

Supplementary Fig. 319. ^{13}C NMR spectrum of **85**. The sample has been recorded in 151 MHz, $CDCl_3$ at 25 °C.

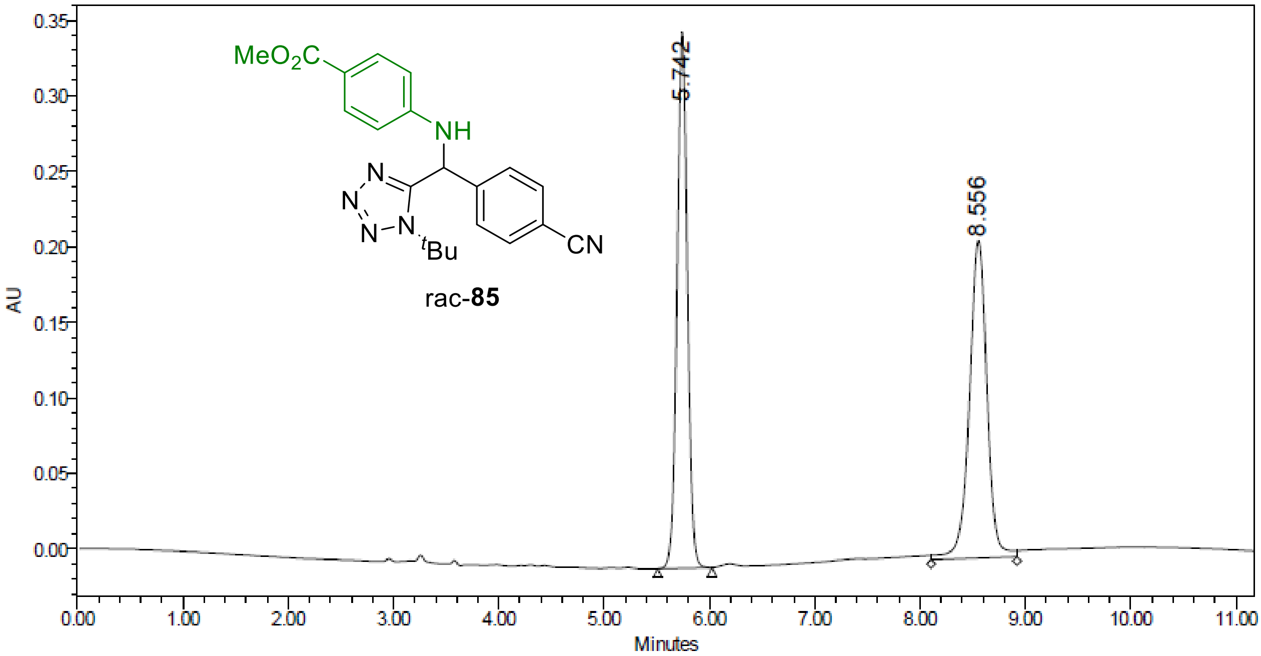

	RT (min)	Area (µV*sec)	% Area	Height (µV)	% Height
1	5.742	2418284	49.64	355133	62.81
2	8.556	2453225	50.36	210236	37.19

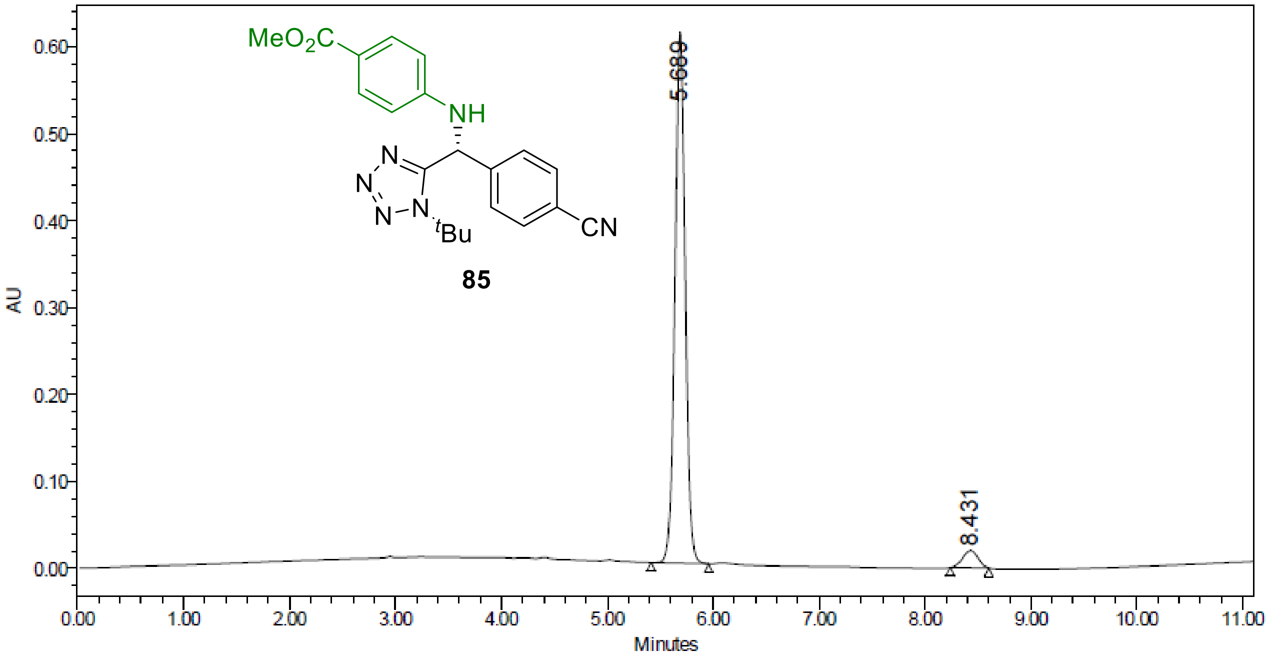

	RT (min)	Area (µV*sec)	% Area	Height (µV)	% Height
1	5.689	4104583	95.40	611095	96.85
2	8.431	197883	4.60	19859	3.15

Supplementary Fig. 320. HPLC of product **85**.

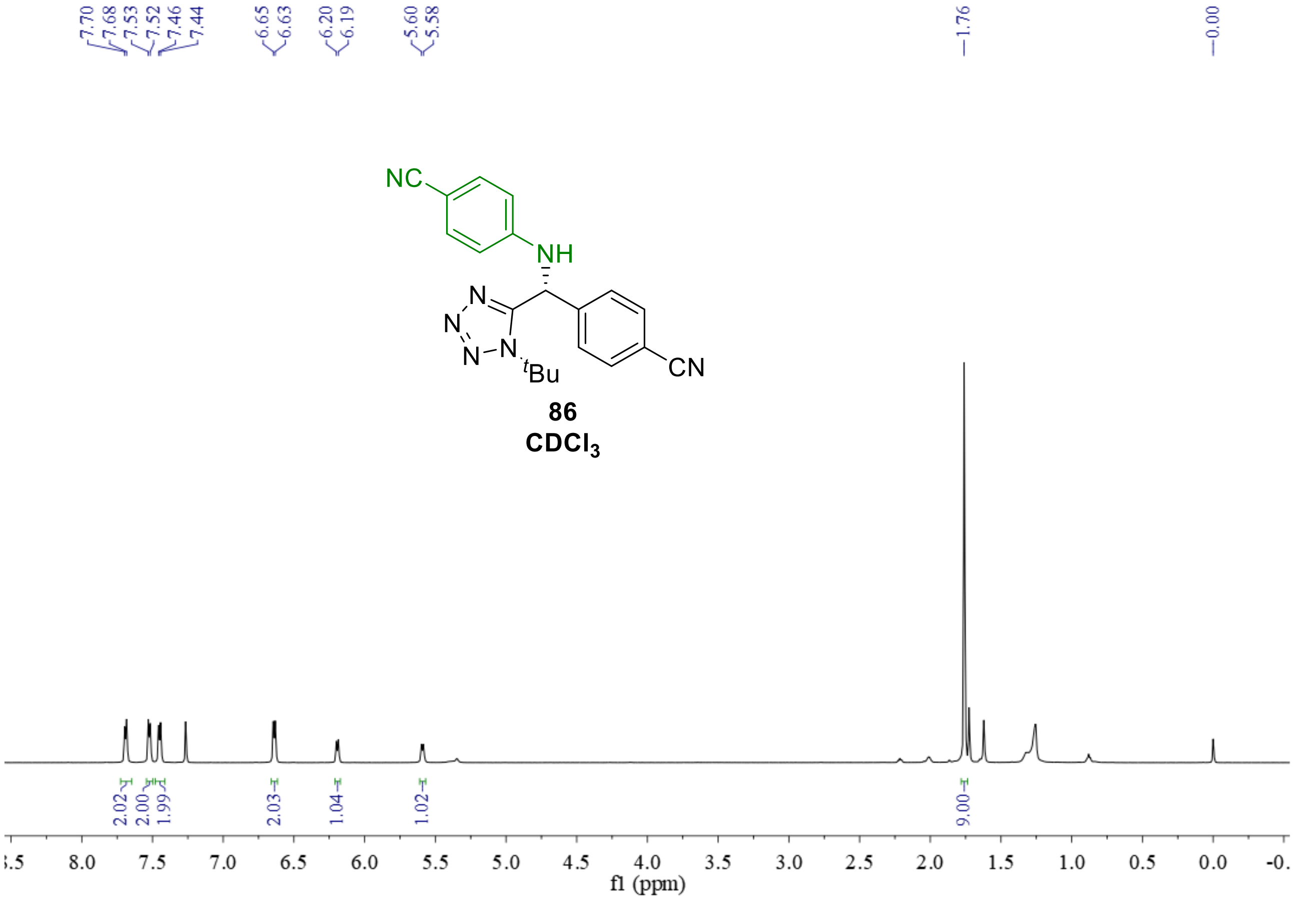

Supplementary Fig. 321. ^{1}H NMR spectrum of **86**. The sample has been recorded in 600 MHz, $CDCl_3$ at 25 °C.

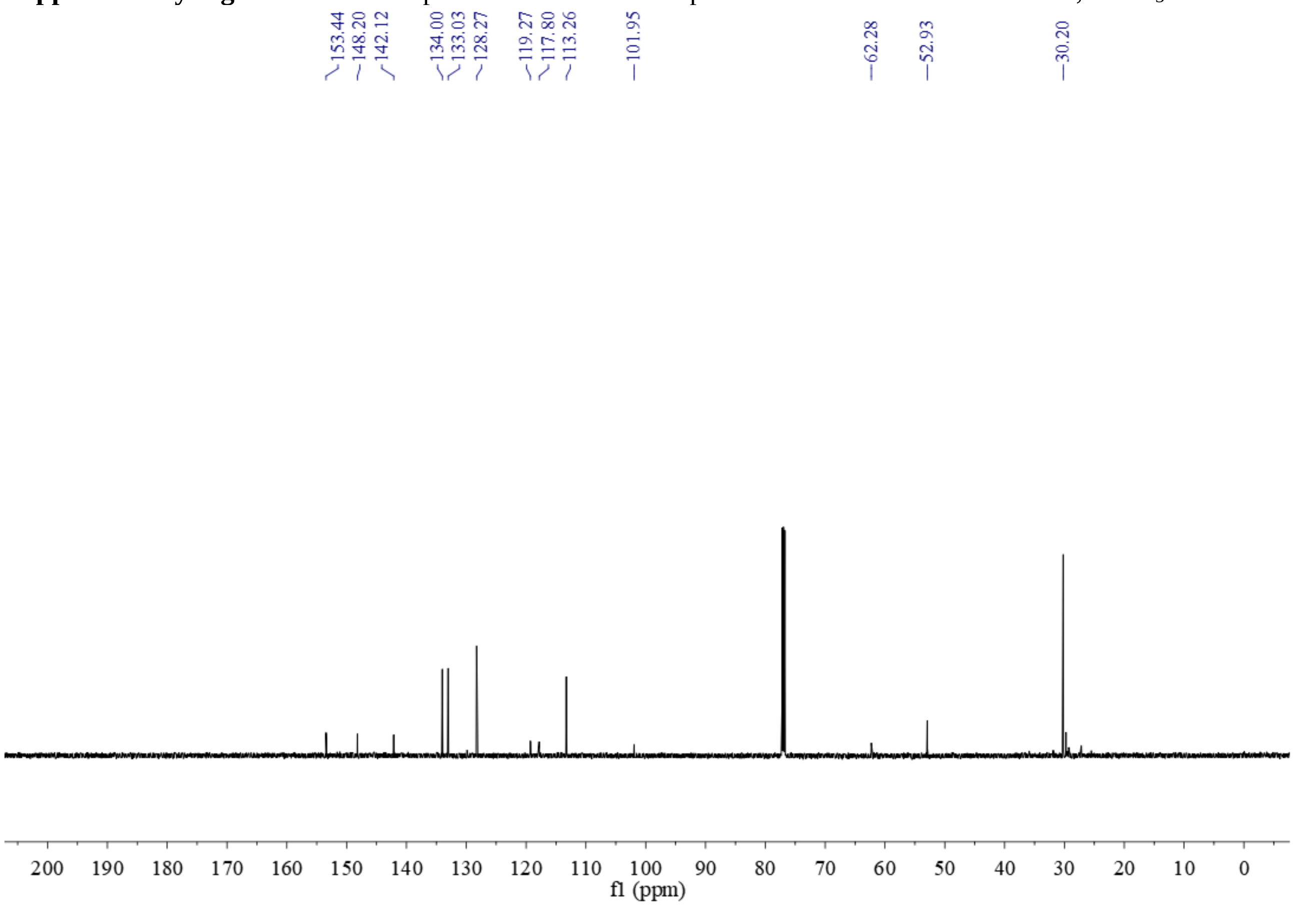

Supplementary Fig. 322. ^{13}C NMR spectrum of **86**. The sample has been recorded in 151 MHz, $CDCl_3$ at 25 °C.

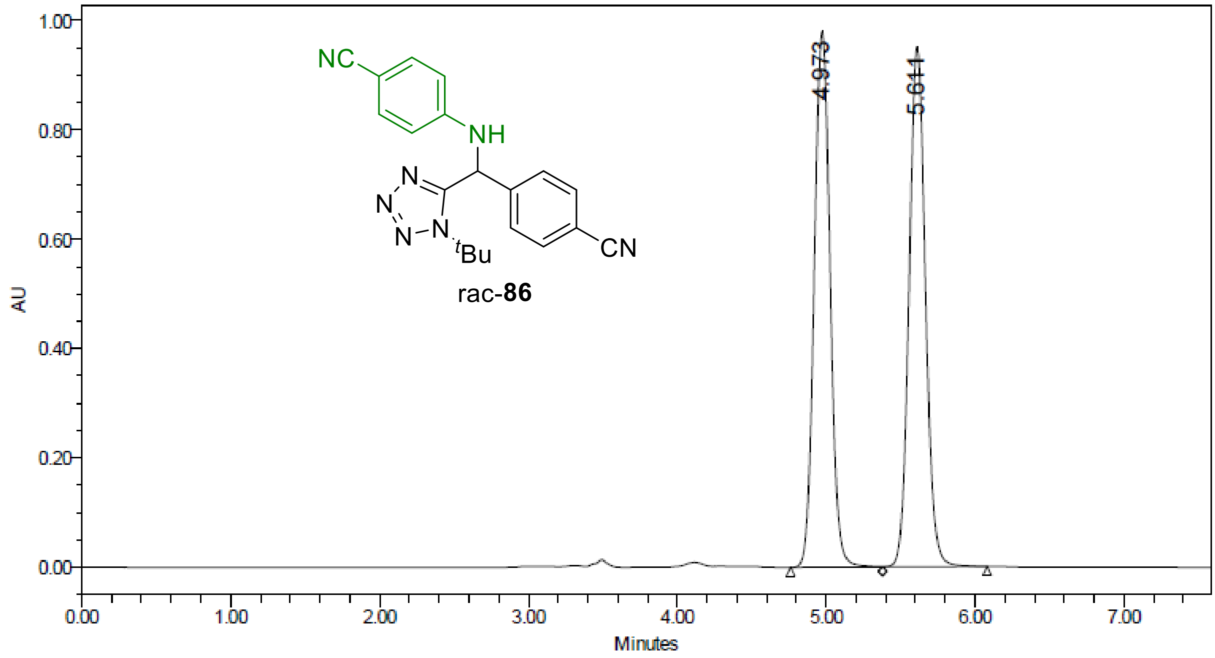

	RT (min)	Peak Type	Area (μV*sec)	% Area	Height (μV)	% Height	Integration Type	Points Across Peak	Start Time (min)	End Time (min)
1	4.973	Unknown	7243786	49.91	979767	50.74	BV	370	4.762	5.378
2	5.611	Unknown	7270493	50.09	951249	49.26	VB	422	5.378	6.082

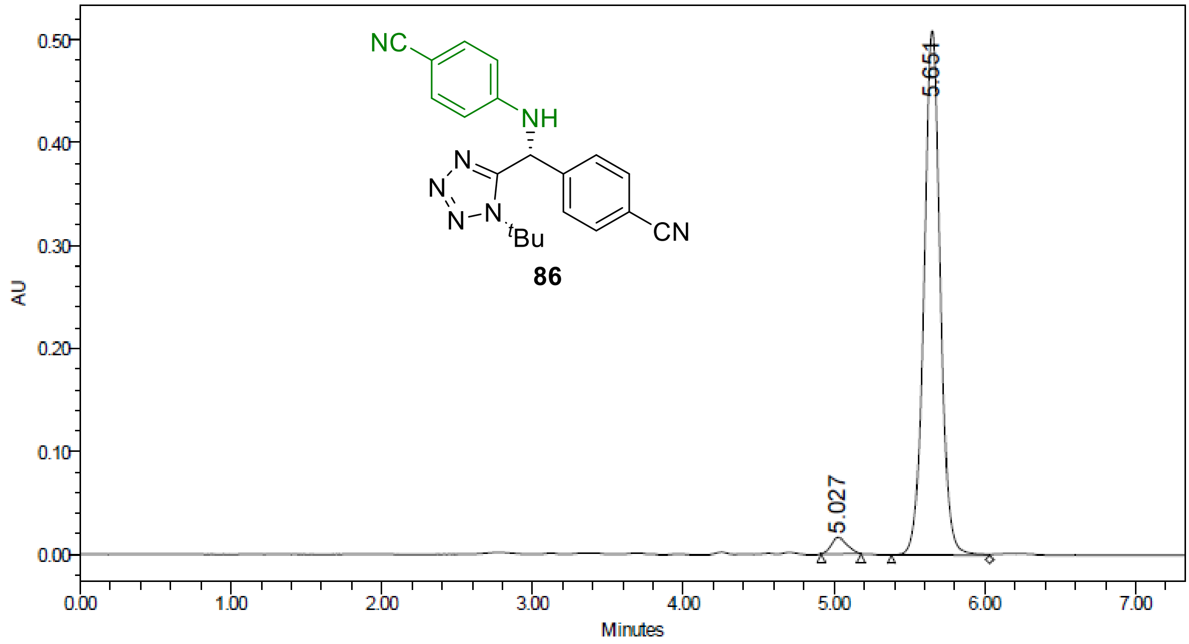

	RT (min)	Peak Type	Area (μV*sec)	% Area	Height (μV)	% Height	Integration Type	Points Across Peak	Start Time (min)	End Time (min)
1	5.027	Unknown	115124	2.93	15997	3.05	bb	158	4.915	5.178
2	5.651	Unknown	3815177	97.07	509300	96.95	bV	391	5.380	6.032

Supplementary Fig. 323. HPLC of product **86**.

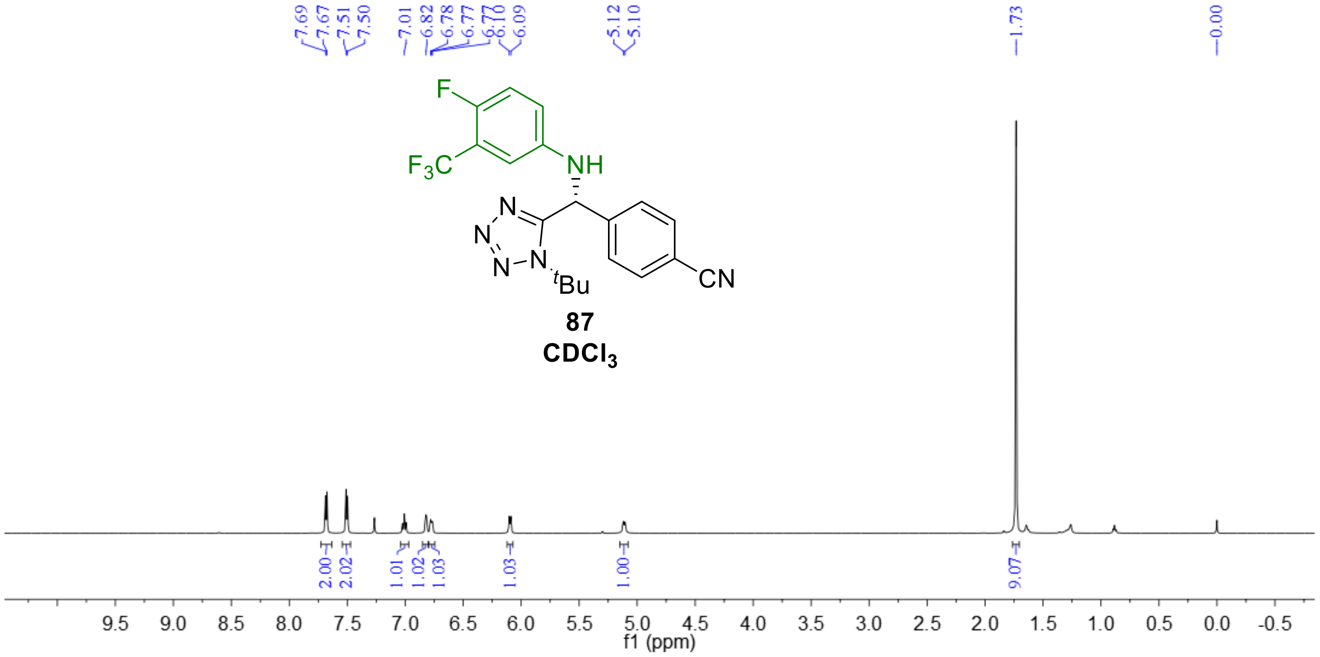

Supplementary Fig. 324. ^{1}H NMR spectrum of **87**. The sample has been recorded in 600 MHz, $CDCl_3$ at 25 °C.

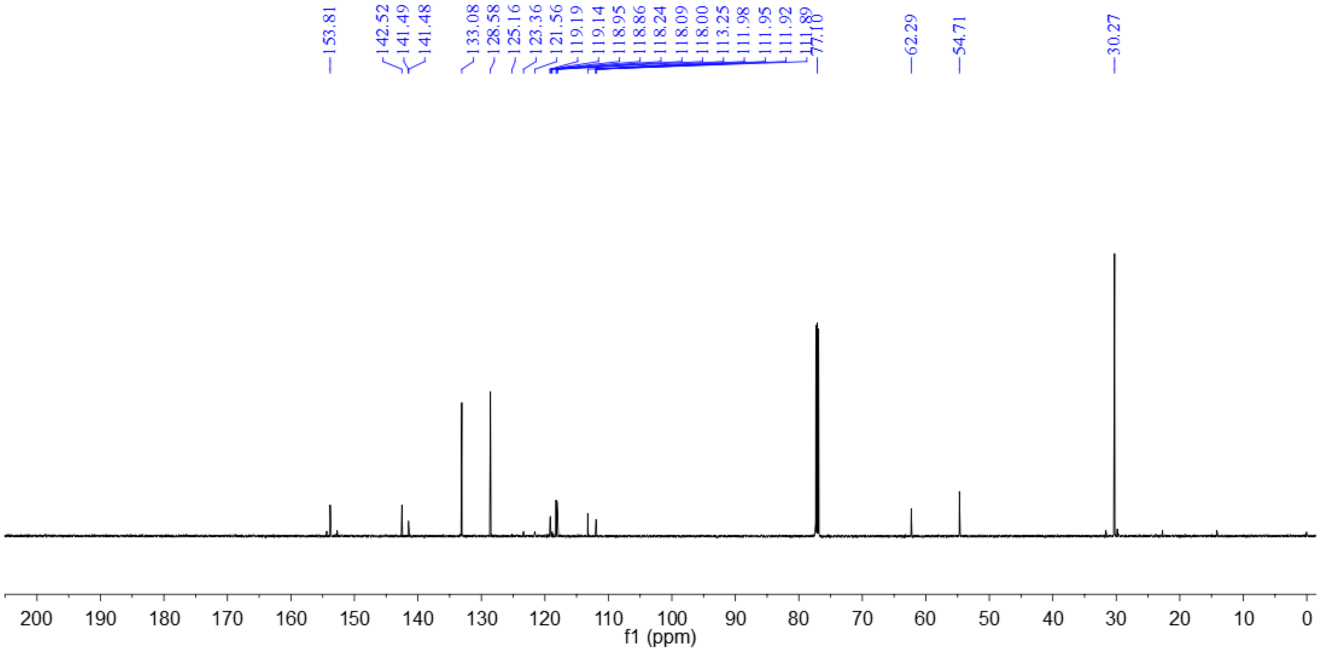

Supplementary Fig. 325. ^{13}C NMR spectrum of **87**. The sample has been recorded in 151 MHz, $CDCl_3$ at 25 °C.

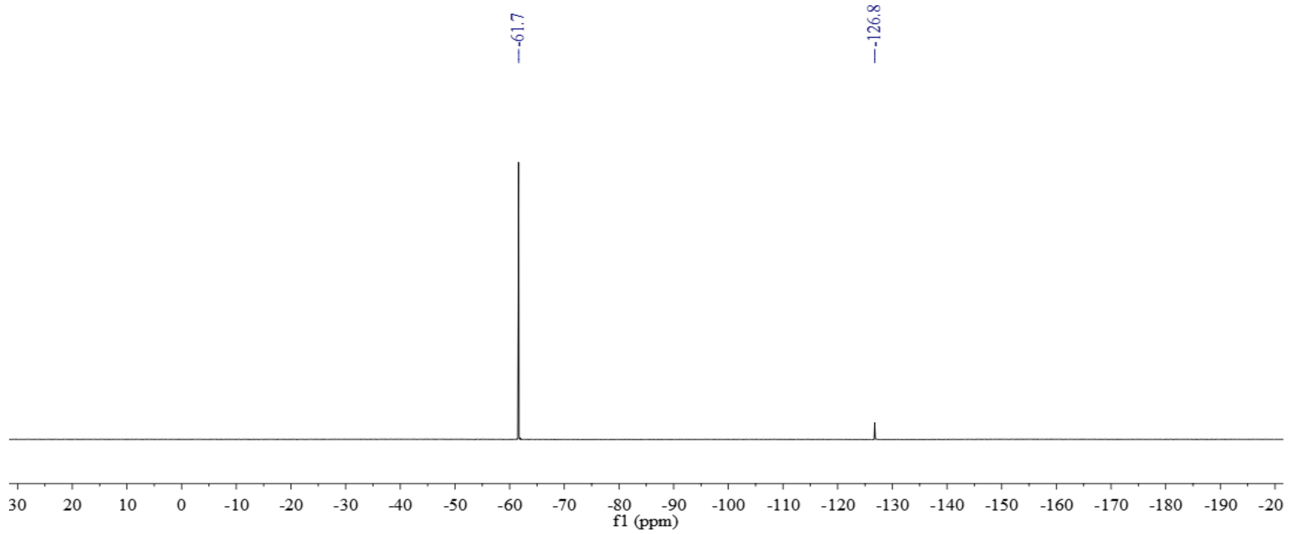

Supplementary Fig. 326. ^{31}F NMR spectrum of **87**. The sample has been recorded in 564 MHz, $CDCl_3$ at 25 °C.

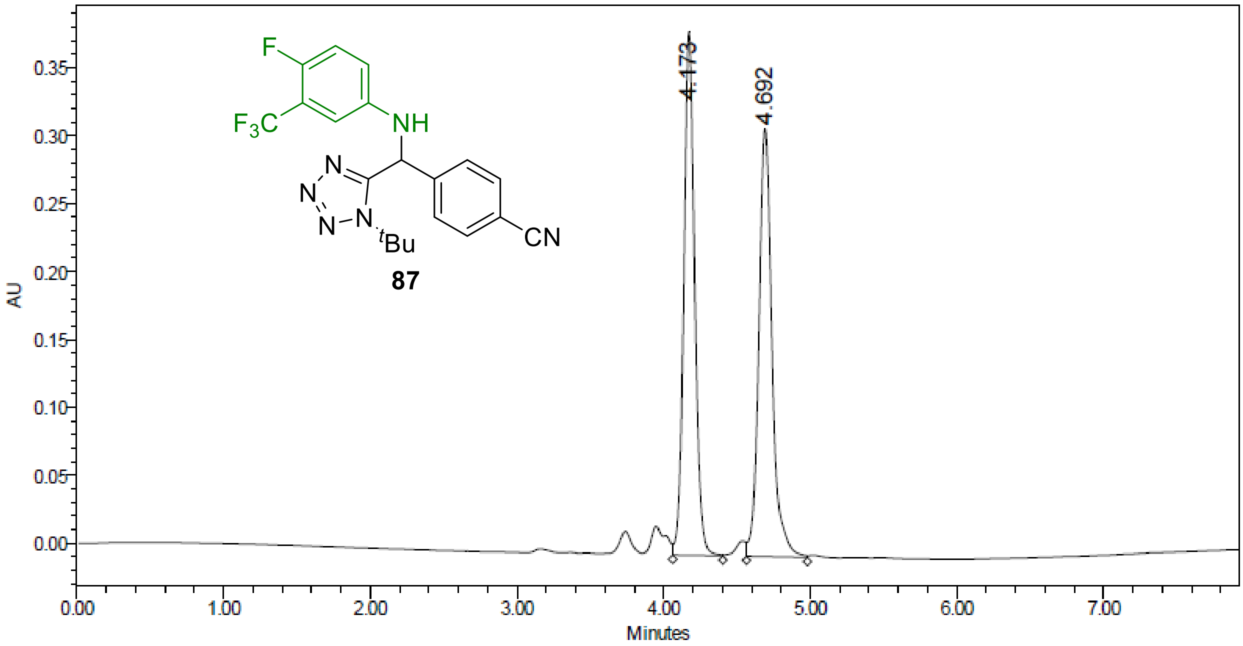

	RT (min)	Area (µV*sec)	% Area	Height (µV)	% Height
1	4.173	1992447	50.33	385729	55.04
2	4.692	1966545	49.67	315082	44.96

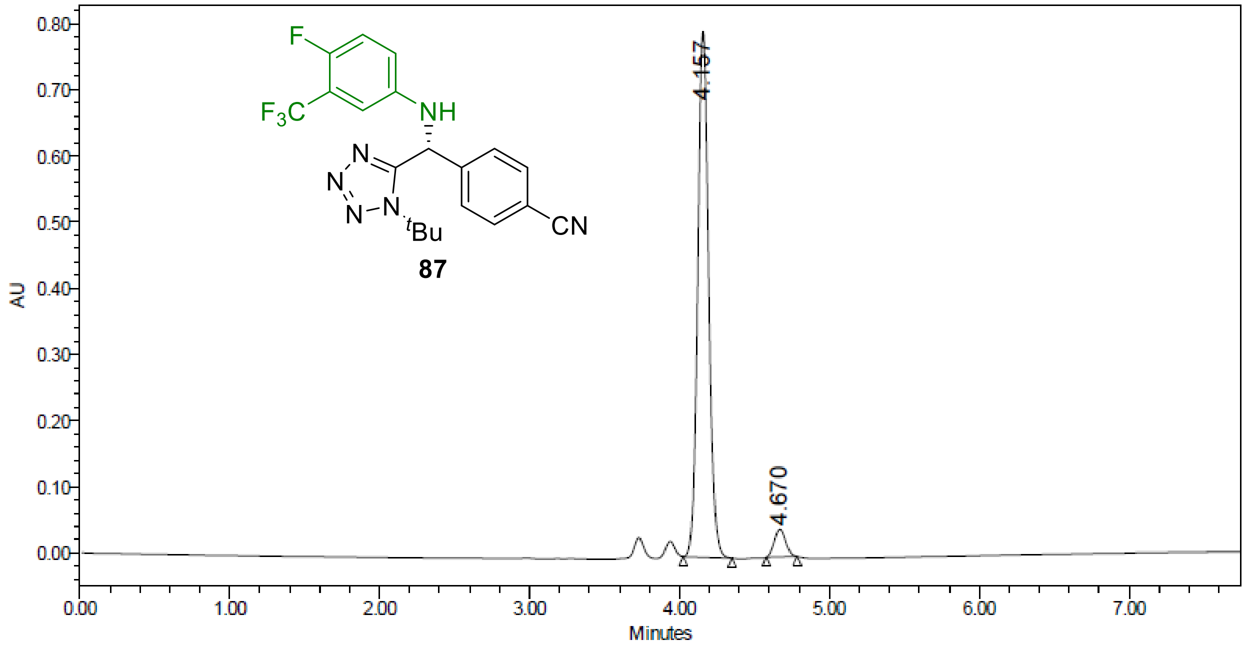

	RT (min)	Area (µV*sec)	% Area	Height (µV)	% Height
1	4.157	3992846	94.78	794648	94.99
2	4.670	219854	5.22	41888	5.01

Supplementary Fig. 327. HPLC of product **87**.

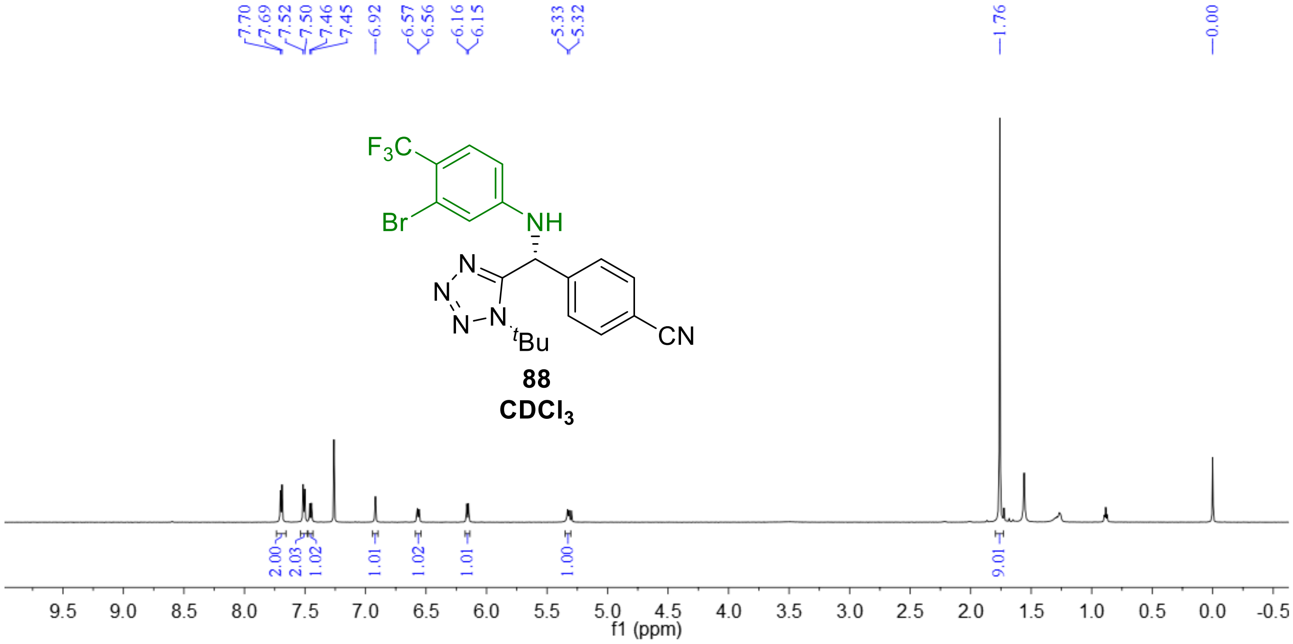

Supplementary Fig. 328. ^{1}H NMR spectrum of **88**. The sample has been recorded in 600 MHz, $CDCl_3$ at 25 °C.

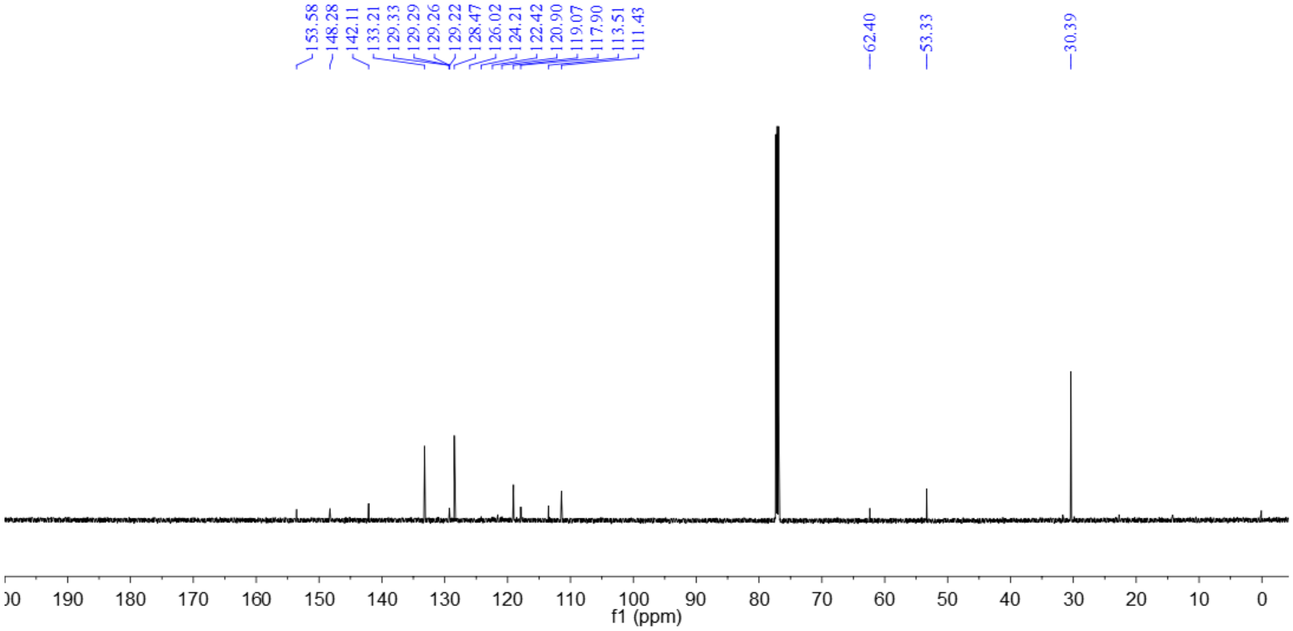

Supplementary Fig. 329. ^{13}C NMR spectrum of **88**. The sample has been recorded in 151 MHz, $CDCl_3$ at 25 °C.

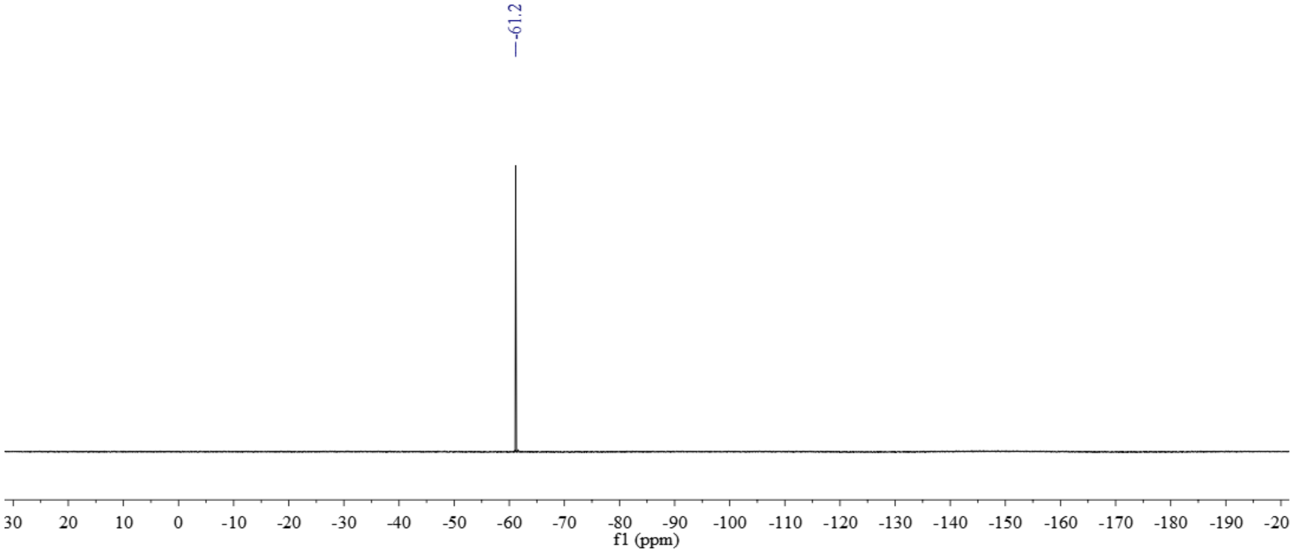

Supplementary Fig. 330. ^{31}F NMR spectrum of **88**. The sample has been recorded in 564 MHz, $CDCl_3$ at 25 °C.

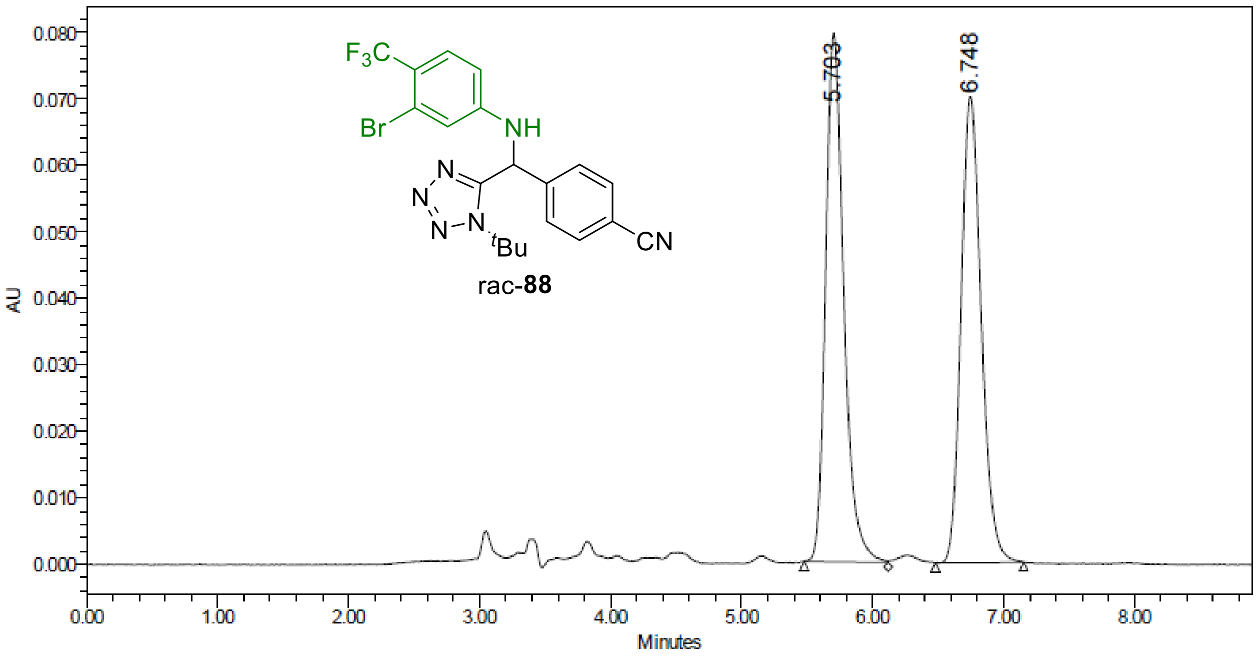

	RT (min)	Area (µV*sec)	% Area	Height (µV)	% Height
1	5.703	766007	50.46	79459	53.14
2	6.748	751921	49.54	70061	46.86

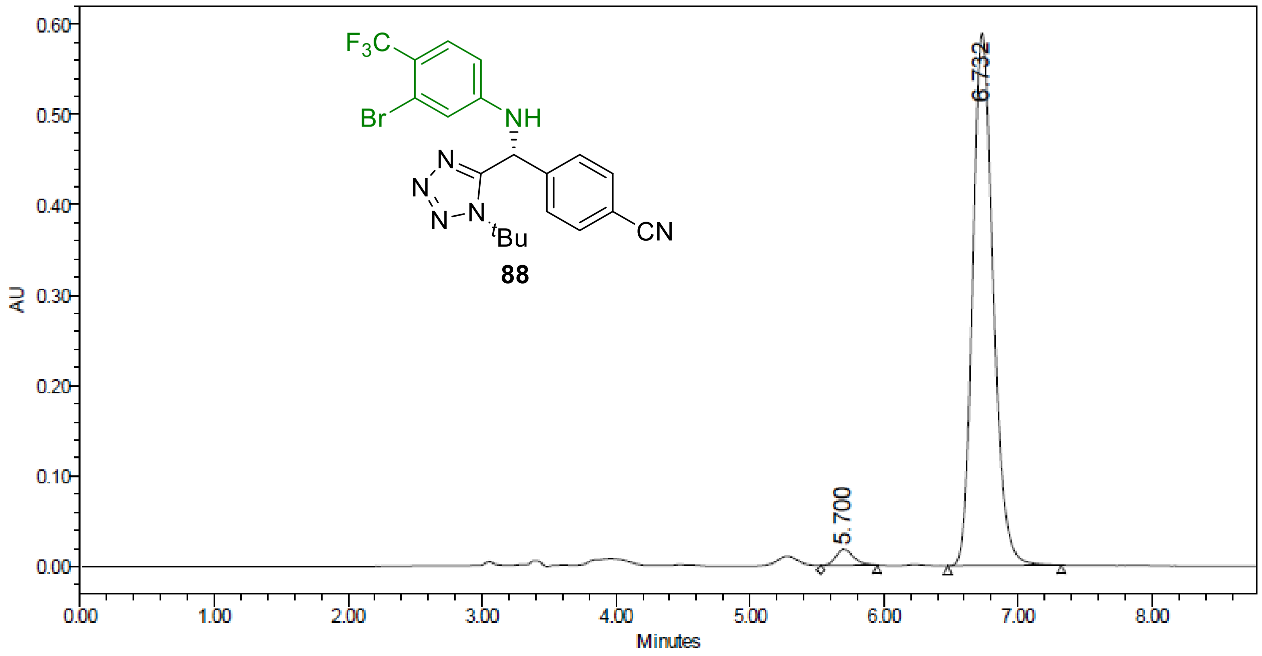

	RT (min)	Area (µV*sec)	% Area	Height (µV)	% Height
1	5.700	161103	2.51	17603	2.90
2	6.732	6266086	97.49	589191	97.10

Supplementary Fig. 331. HPLC of product **88**.

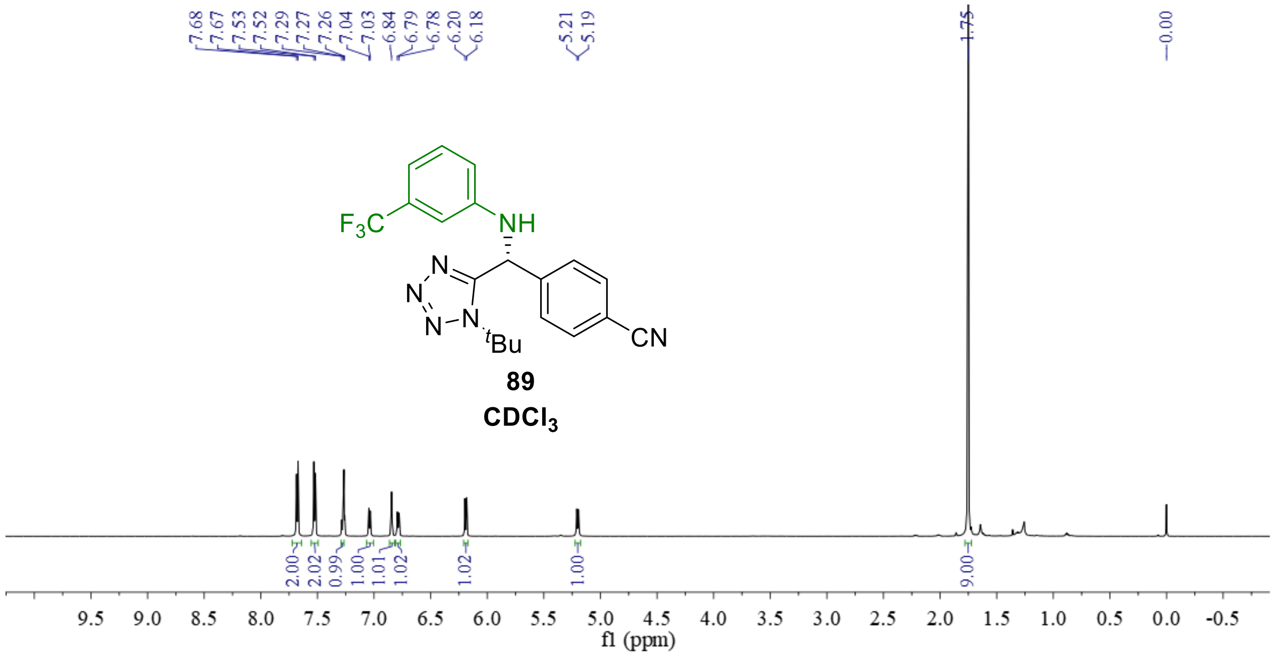

Supplementary Fig. 332. ^{1}H NMR spectrum of **89**. The sample has been recorded in 600 MHz, $CDCl_3$ at 25 °C.

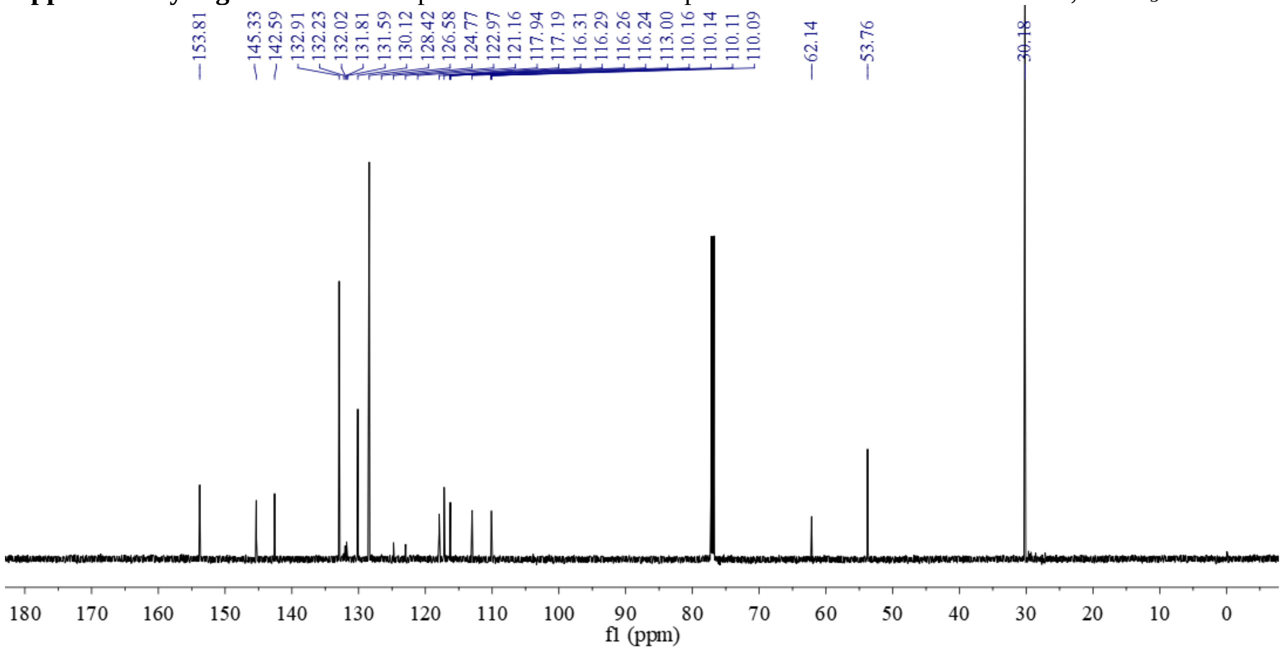

Supplementary Fig. 333. ^{13}C NMR spectrum of **89**. The sample has been recorded in 151 MHz, $CDCl_3$ at 25 °C.

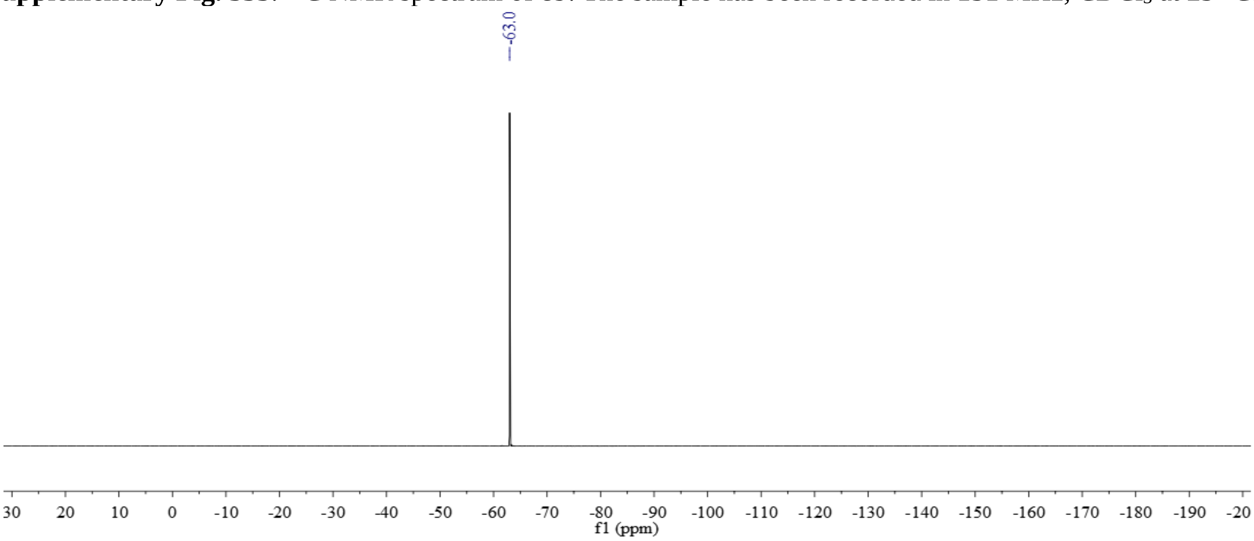

Supplementary Fig. 334. [31]F NMR spectrum of **89**. The sample has been recorded in 564 MHz, $CDCl_3$ at 25 °C.

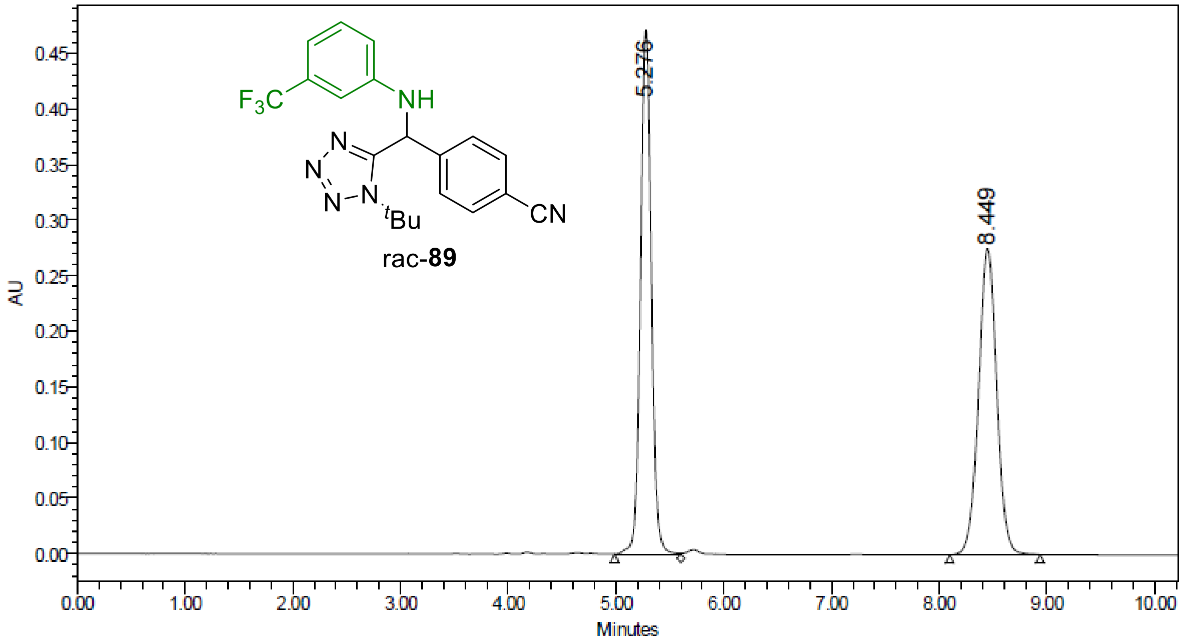

	RT (min)	Peak Type	Area (µV*sec)	% Area	Height (µV)	% Height	Integration Type	Points Across Peak	Start Time (min)	End Time (min)
1	5.276	Unknown	3277498	50.36	471014	63.13	BV	369	4.985	5.600
2	8.449	Unknown	3230418	49.64	275069	36.87	BB	503	8.097	8.935

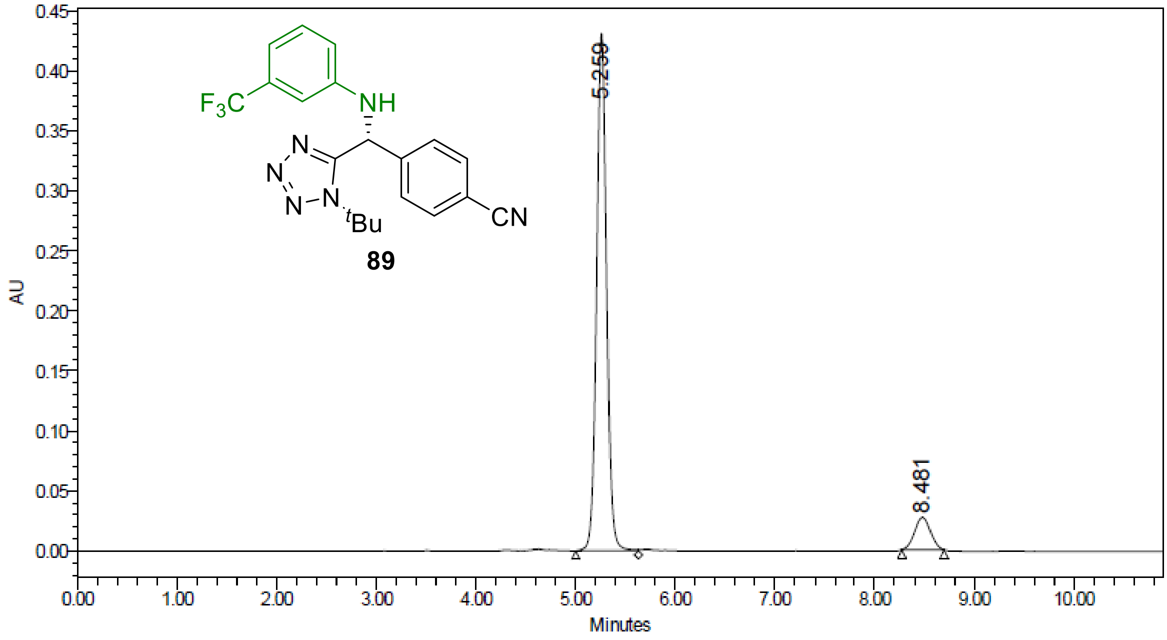

	RT (min)	Peak Type	Area (µV*sec)	% Area	Height (µV)	% Height	Integration Type	Points Across Peak	Start Time (min)	End Time (min)
1	5.259	Unknown	3005742	90.97	431226	94.12	BV	377	5.000	5.628
2	8.481	Unknown	298197	9.03	26937	5.88	bb	253	8.275	8.697

Supplementary Fig. 335. HPLC of product **89**.

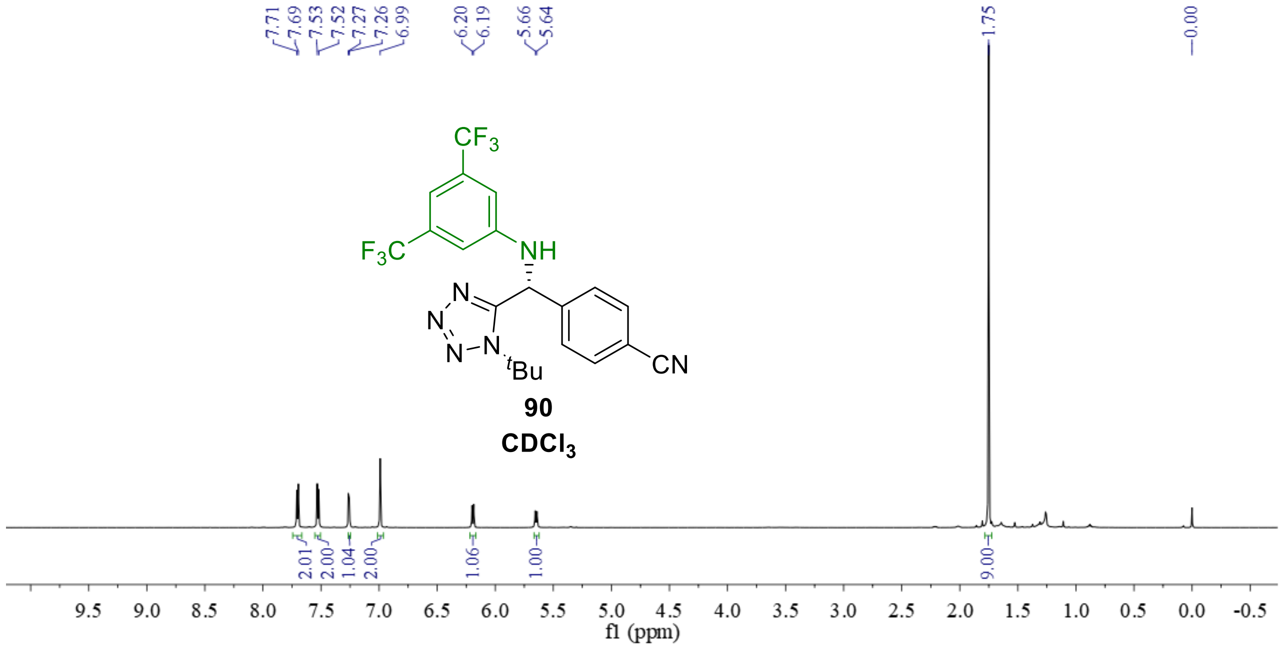

Supplementary Fig. 336. ^{1}H NMR spectrum of **90**. The sample has been recorded in 600 MHz, $CDCl_3$ at 25 °C.

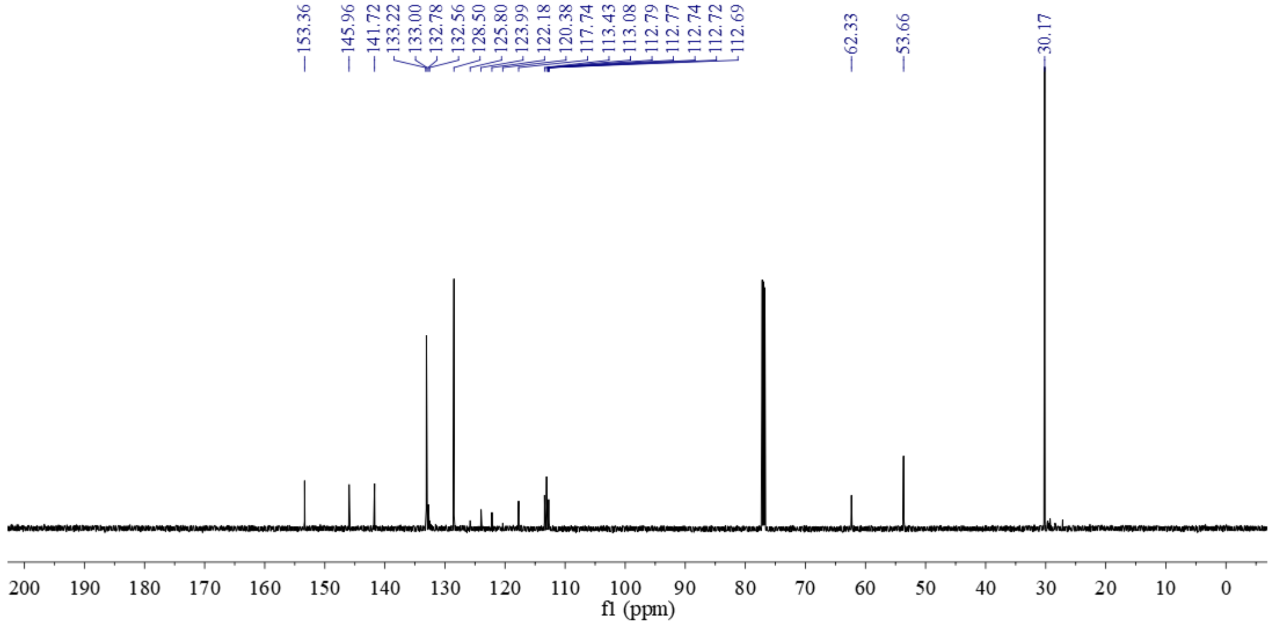

Supplementary Fig. 337. ^{13}C NMR spectrum of **90**. The sample has been recorded in 151 MHz, $CDCl_3$ at 25 °C.

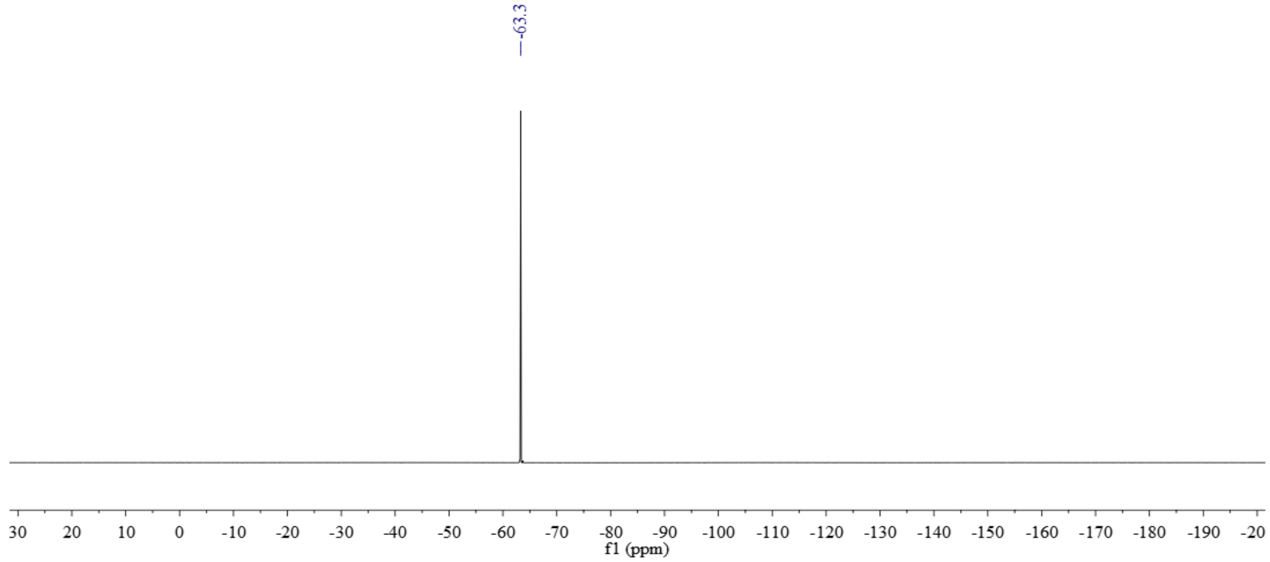

Supplementary Fig. 338. ^{31}F NMR spectrum of **90**. The sample has been recorded in 564 MHz, $CDCl_3$ at 25 °C.

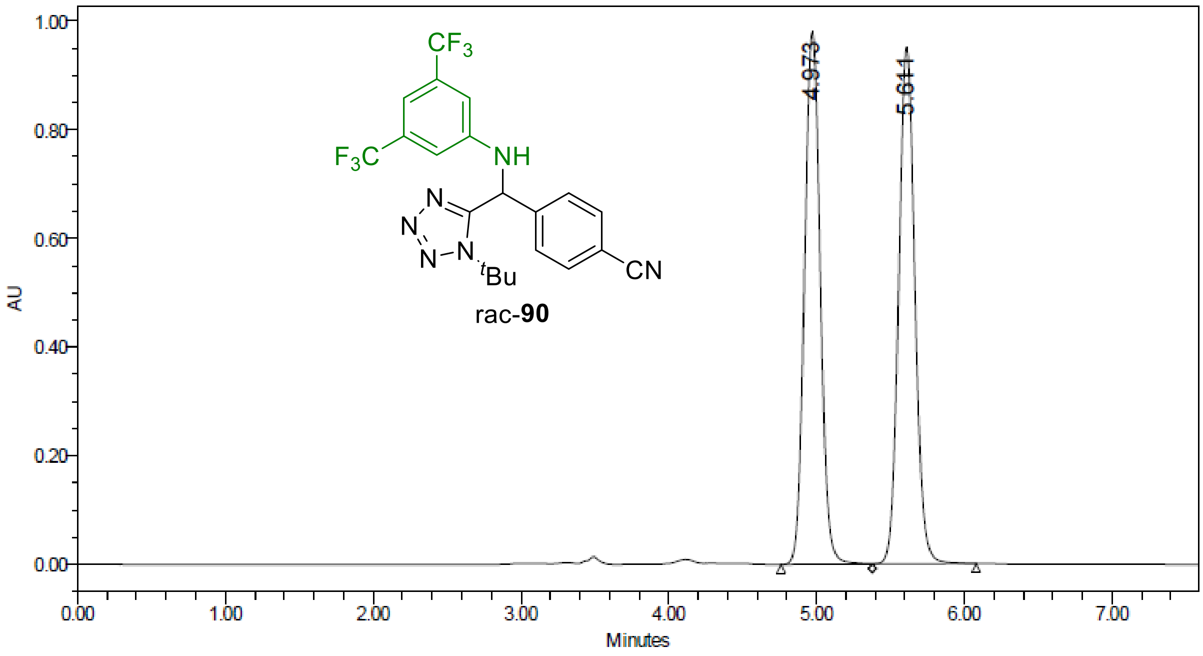

	RT (min)	Peak Type	Area (µV*sec)	% Area	Height (µV)	% Height	Integration Type	Points Across Peak	Start Time (min)	End Time (min)
1	4.973	Unknown	7243786	49.91	979767	50.74	BV	370	4.762	5.378
2	5.611	Unknown	7270493	50.09	951249	49.26	VB	422	5.378	6.082

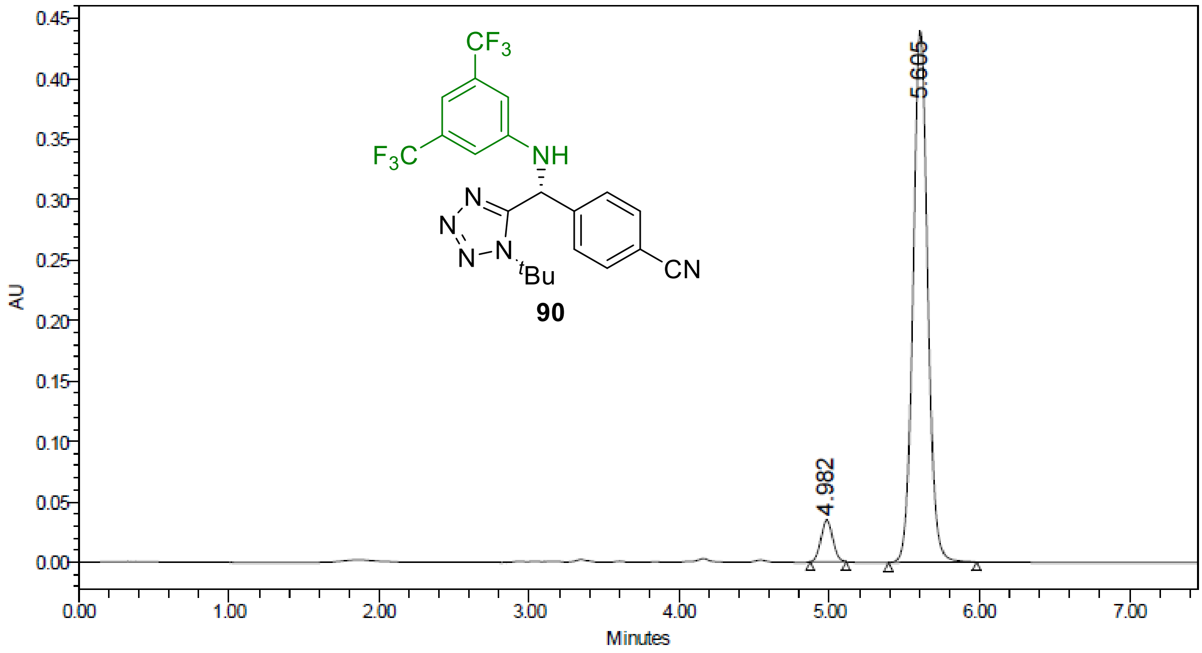

	RT (min)	Peak Type	Area (µV*sec)	% Area	Height (µV)	% Height	Integration Type	Points Across Peak	Start Time (min)	End Time (min)
1	4.982	Unknown	190250	6.00	34708	7.32	bb	143	4.872	5.110
2	5.605	Unknown	2981709	94.00	439275	92.68	BB	352	5.393	5.980

Supplementary Fig. 339. HPLC of product **90**.

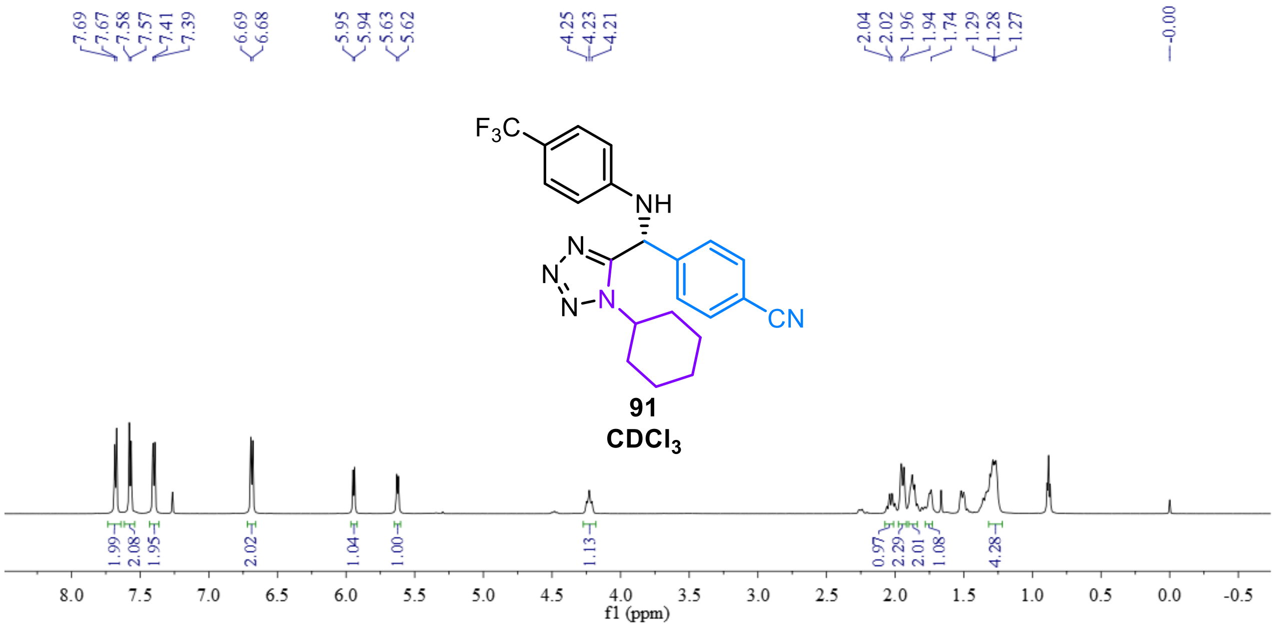

Supplementary Fig. 340. ^{1}H NMR spectrum of **91**. The sample has been recorded in 600 MHz, $CDCl_3$ at 25 °C.

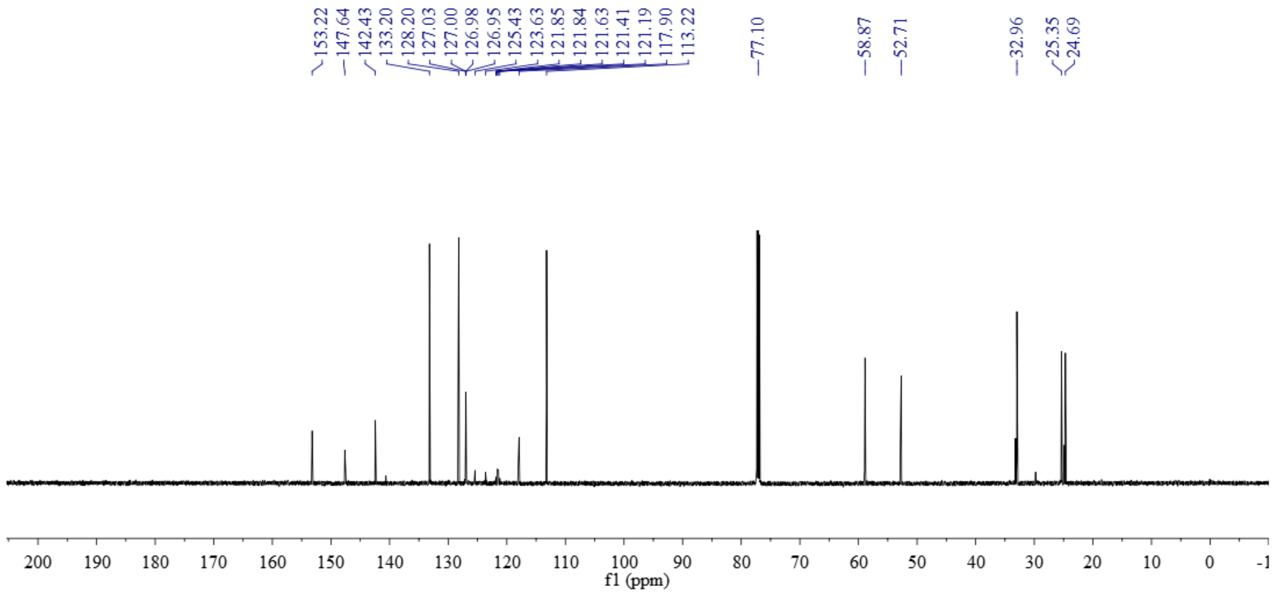

Supplementary Fig. 341. ^{13}C NMR spectrum of **91**. The sample has been recorded in 151 MHz, $CDCl_3$ at 25 °C.

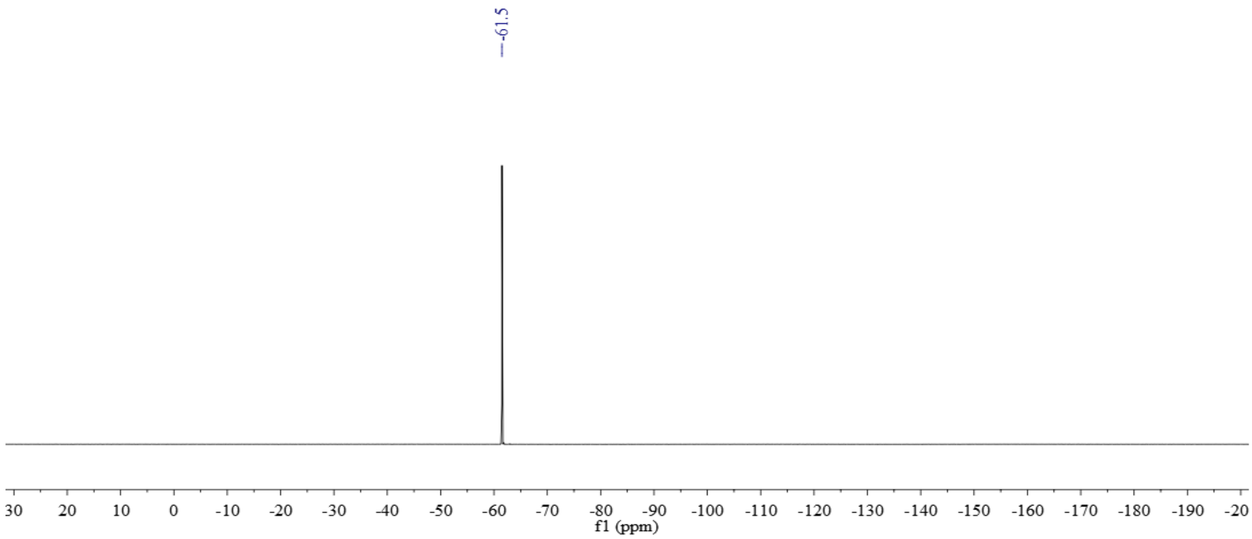

Supplementary Fig. 342. [31]F NMR spectrum of **91**. The sample has been recorded in 564 MHz, $CDCl_3$ at 25 °C.

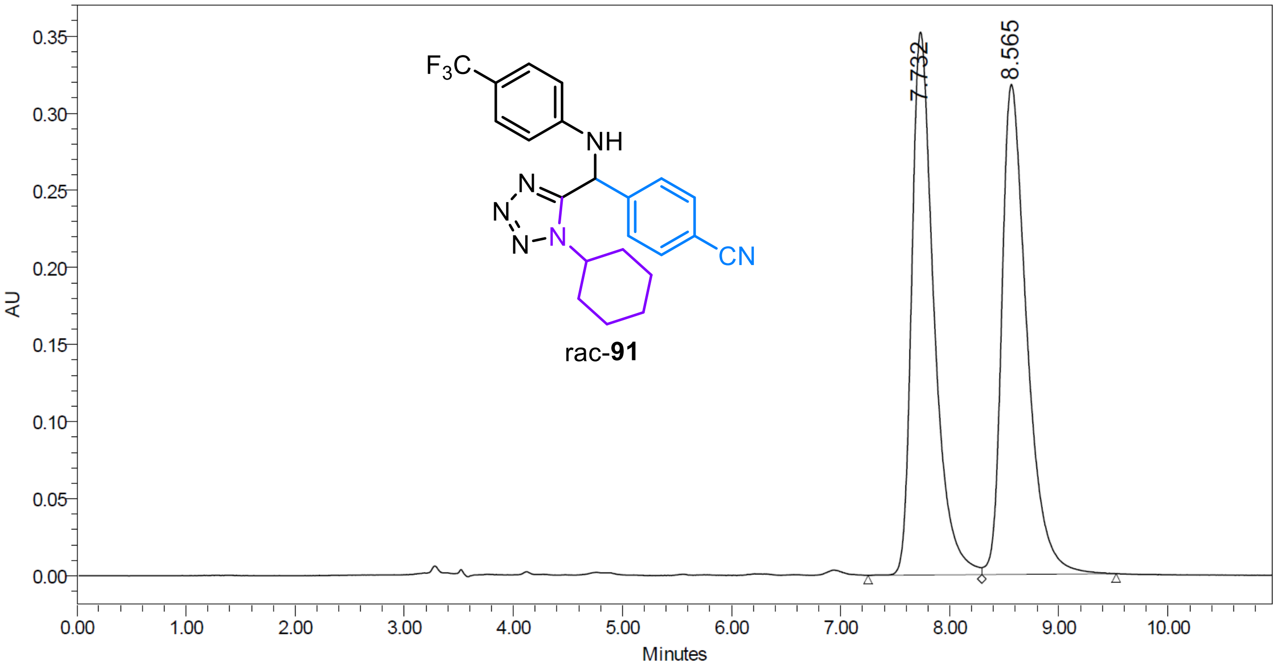

	RT (min)	Area (µV*sec)	% Area	Height (µV)	% Height
1	7.732	4974569	49.59	352047	52.56
2	8.565	5057143	50.41	317752	47.44

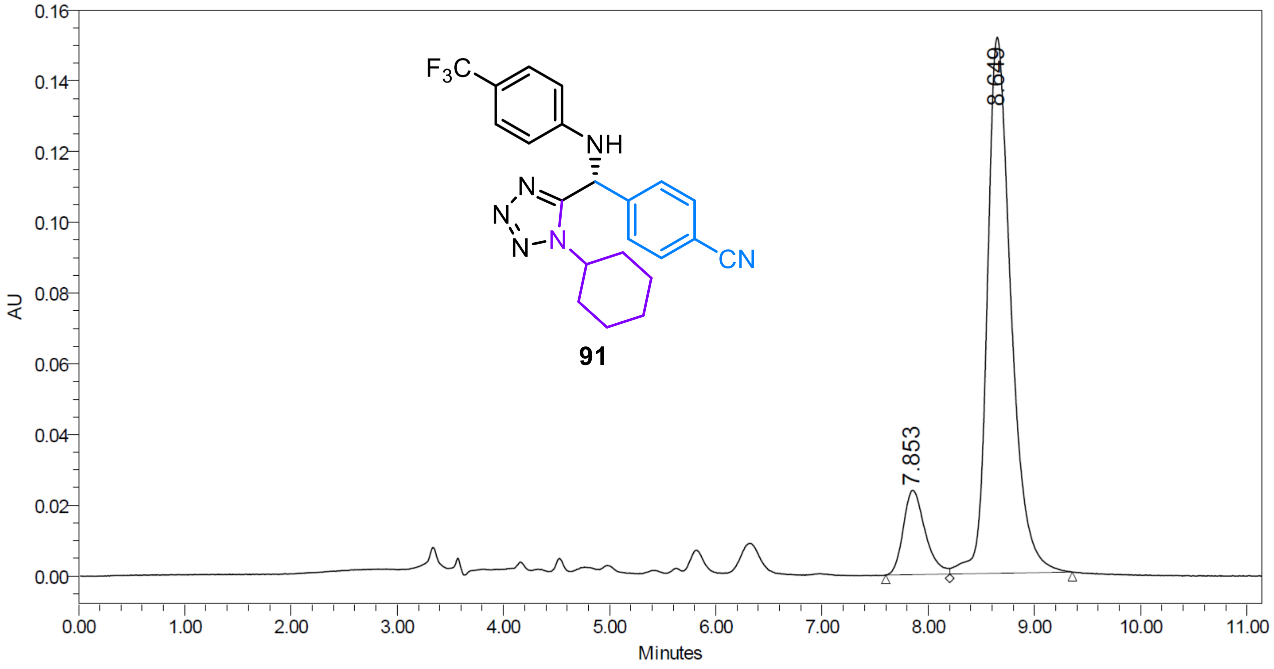

	RT (min)	Area (µV*sec)	% Area	Height (µV)	% Height
1	7.853	342216	12.69	23885	13.61
2	8.649	2354614	87.31	151595	86.39

Supplementary Fig. 343. HPLC of product **91**.

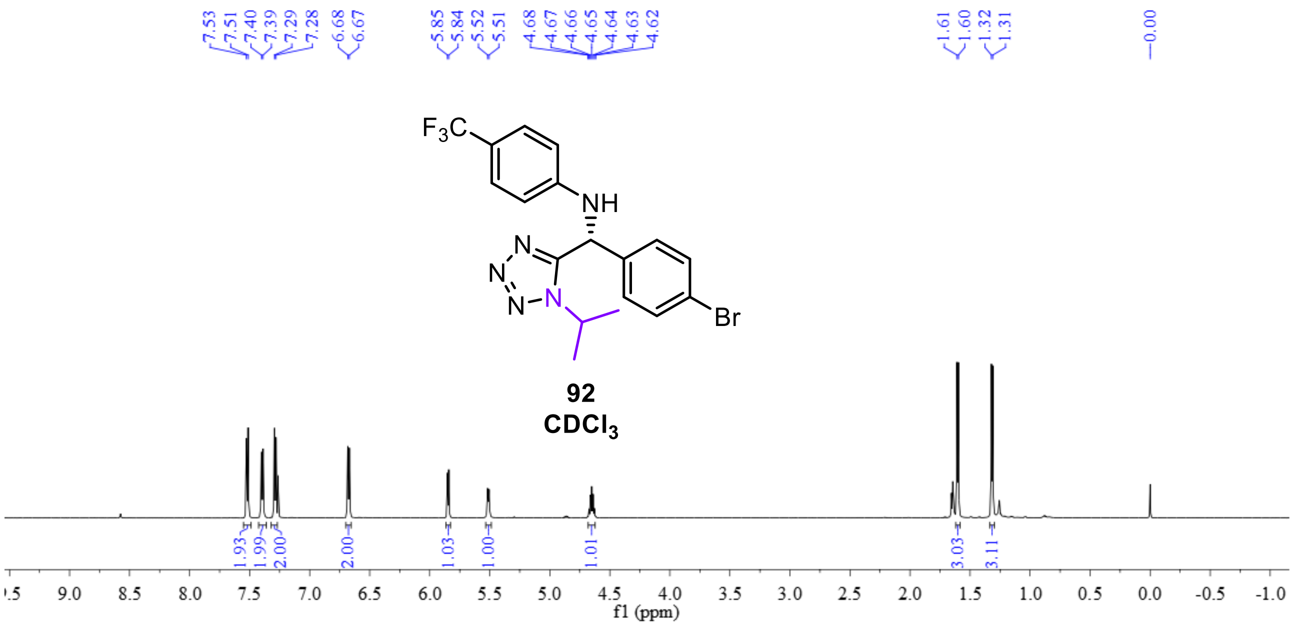

Supplementary Fig. 344. ^{1}H NMR spectrum of **92**. The sample has been recorded in 600 MHz, $CDCl_3$ at 25 °C.

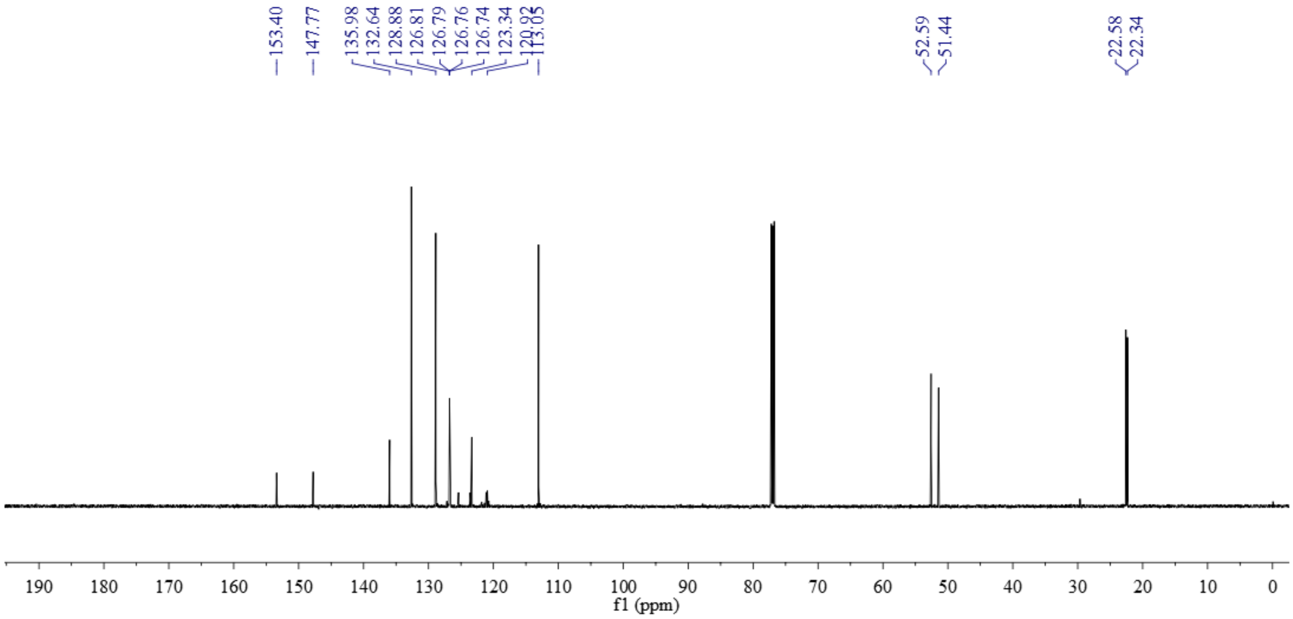

Supplementary Fig. 345. ^{13}C NMR spectrum of **92**. The sample has been recorded in 151 MHz, $CDCl_3$ at 25 °C.

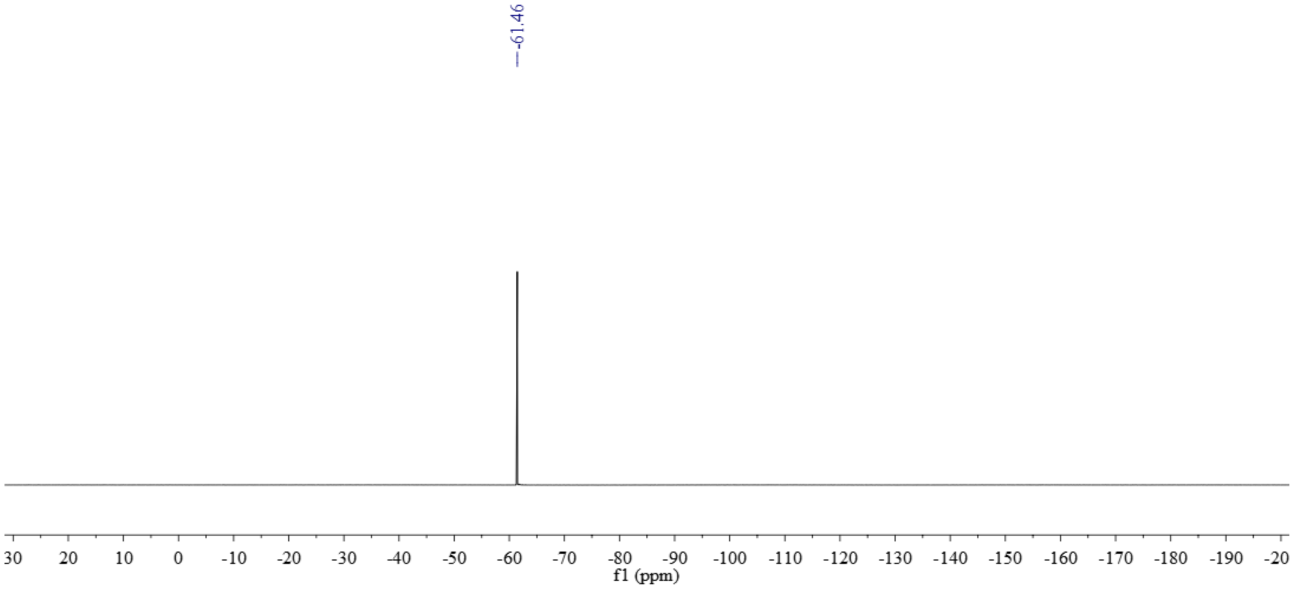

Supplementary Fig. 346. ^{31}F NMR spectrum of **92**. The sample has been recorded in 564 MHz, $CDCl_3$ at 25 °C.

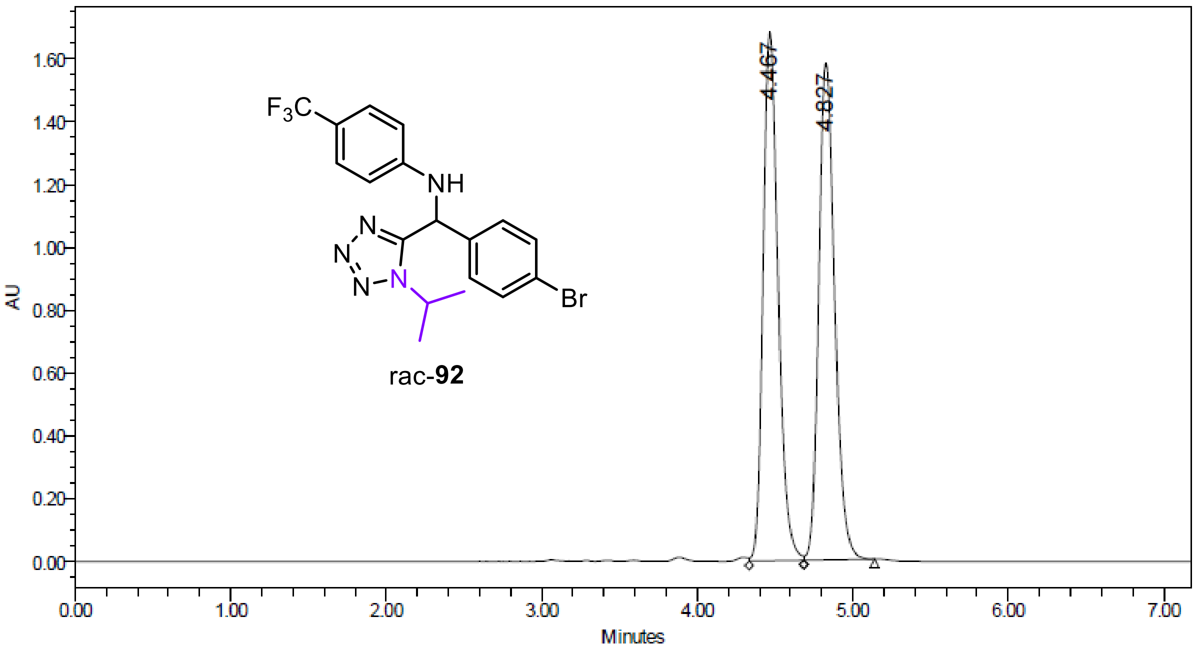

	RT (min)	Peak Type	Area (µV*sec)	% Area	Height (µV)	% Height	Integration Type	Points Across Peak	Start Time (min)	End Time (min)
1	4.467	Unknown	11254156	49.93	1681309	51.56	VV	211	4.333	4.685
2	4.827	Unknown	11287155	50.07	1579637	48.44	VB	273	4.685	5.140

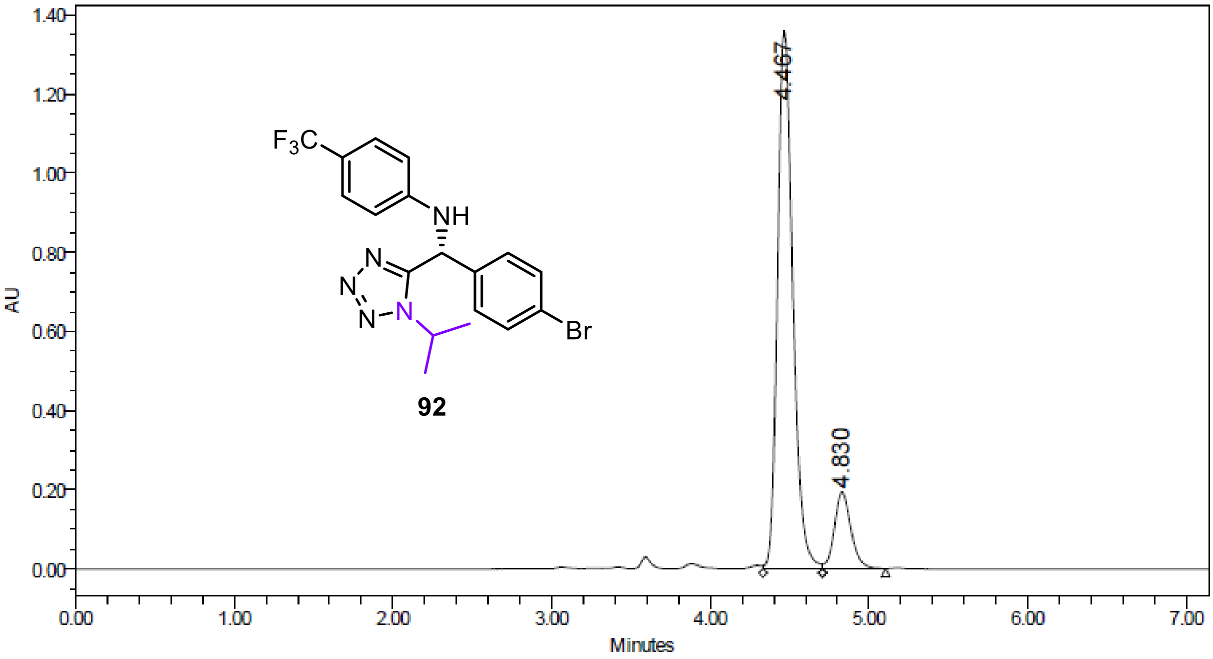

	RT (min)	Peak Type	Area (µV*sec)	% Area	Height (µV)	% Height	Integration Type	Points Across Peak	Start Time (min)	End Time (min)
1	4.467	Unknown	9075449	86.73	1356778	87.54	VV	225	4.332	4.707
2	4.830	Unknown	1388876	13.27	193038	12.46	VB	239	4.707	5.105

Supplementary Fig. 347. HPLC of product **92**.

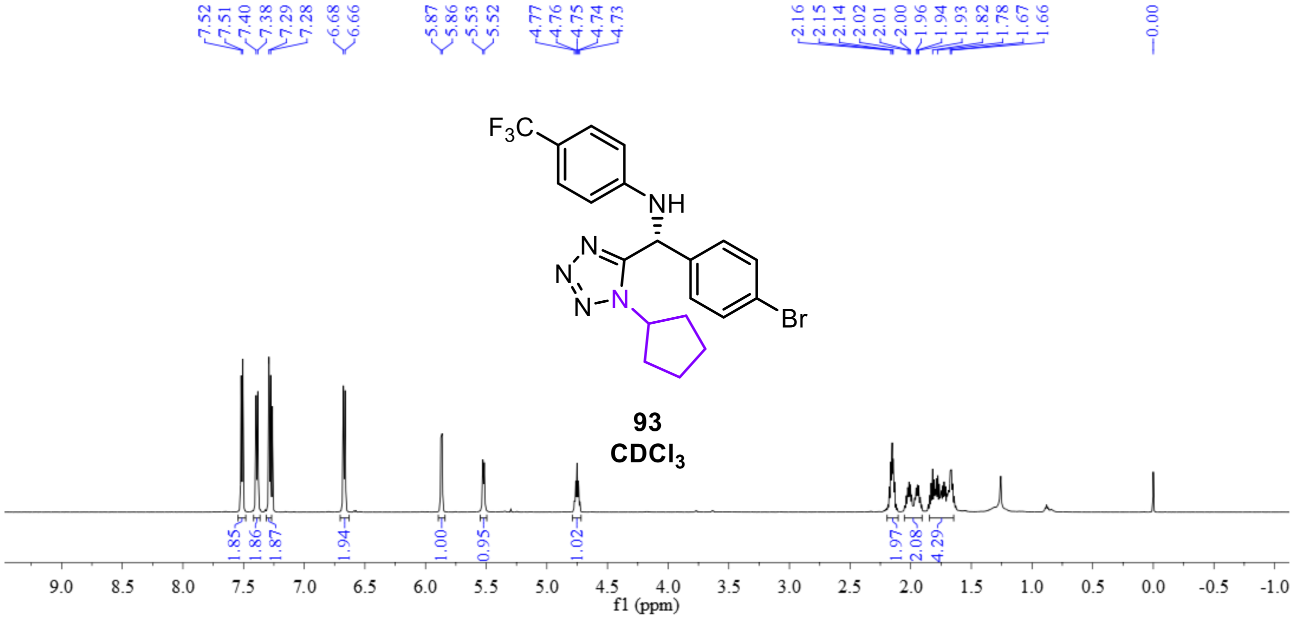

Supplementary Fig. 348. ^{1}H NMR spectrum of **93**. The sample has been recorded in 600 MHz, $CDCl_3$ at 25 °C.

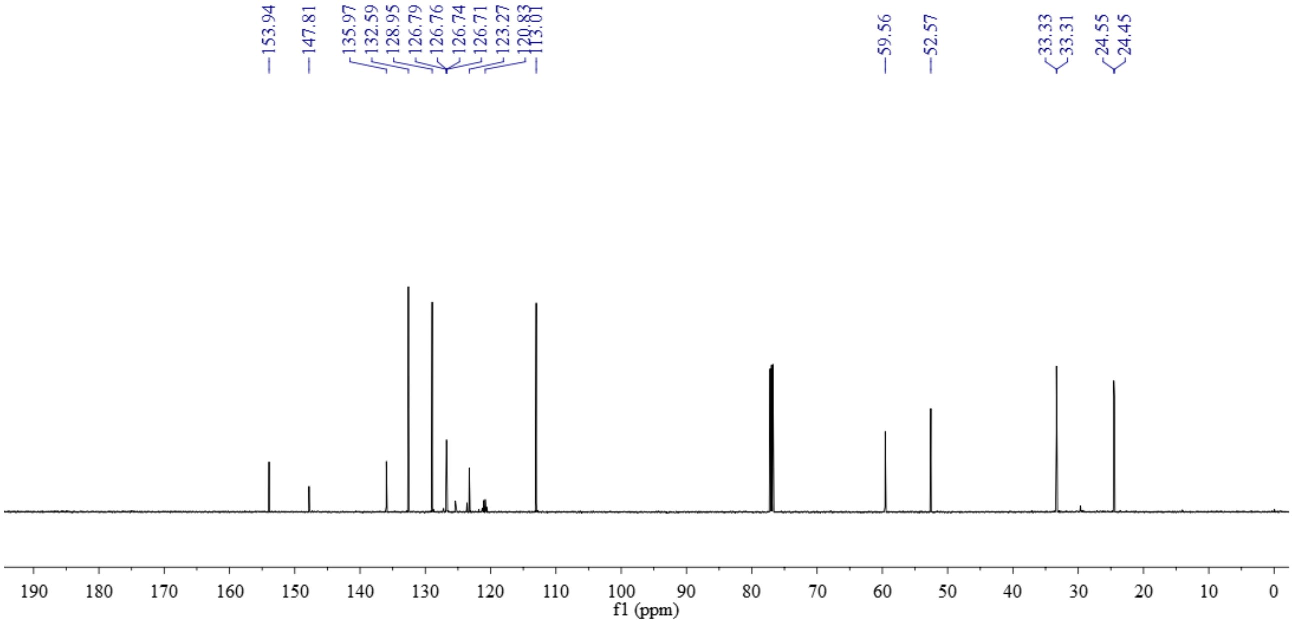

Supplementary Fig. 349. ^{13}C NMR spectrum of **93**. The sample has been recorded in 151 MHz, $CDCl_3$ at 25 °C.

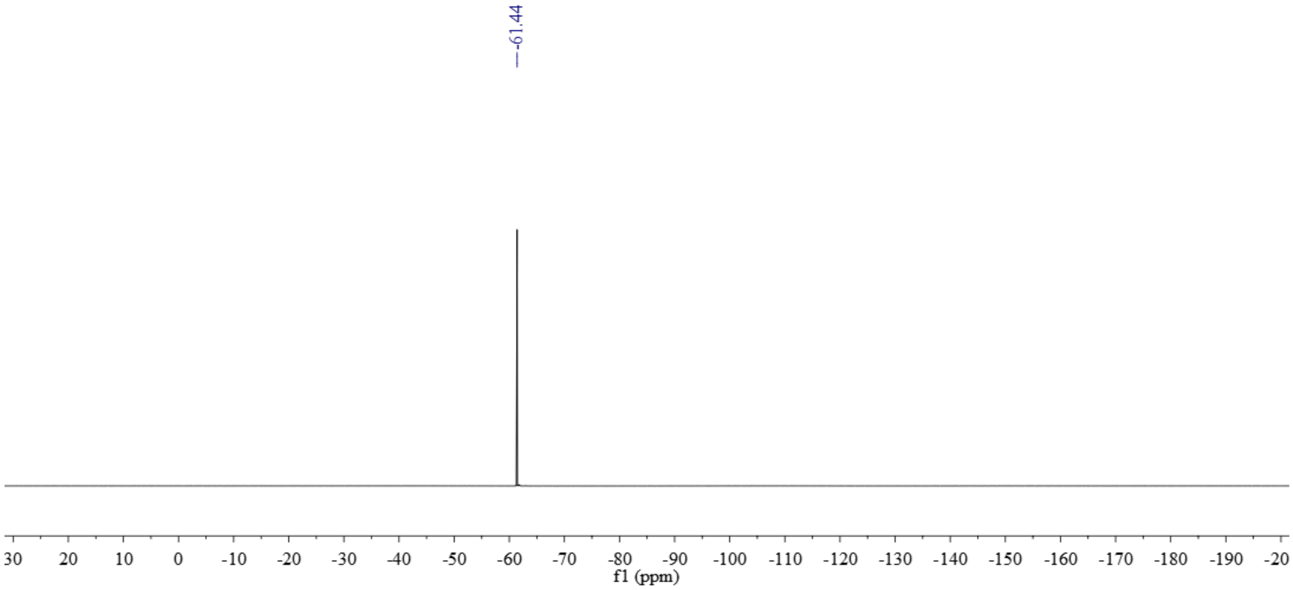

Supplementary Fig. 350. [31]F NMR spectrum of **93**. The sample has been recorded in 564 MHz, $CDCl_3$ at 25 °C.

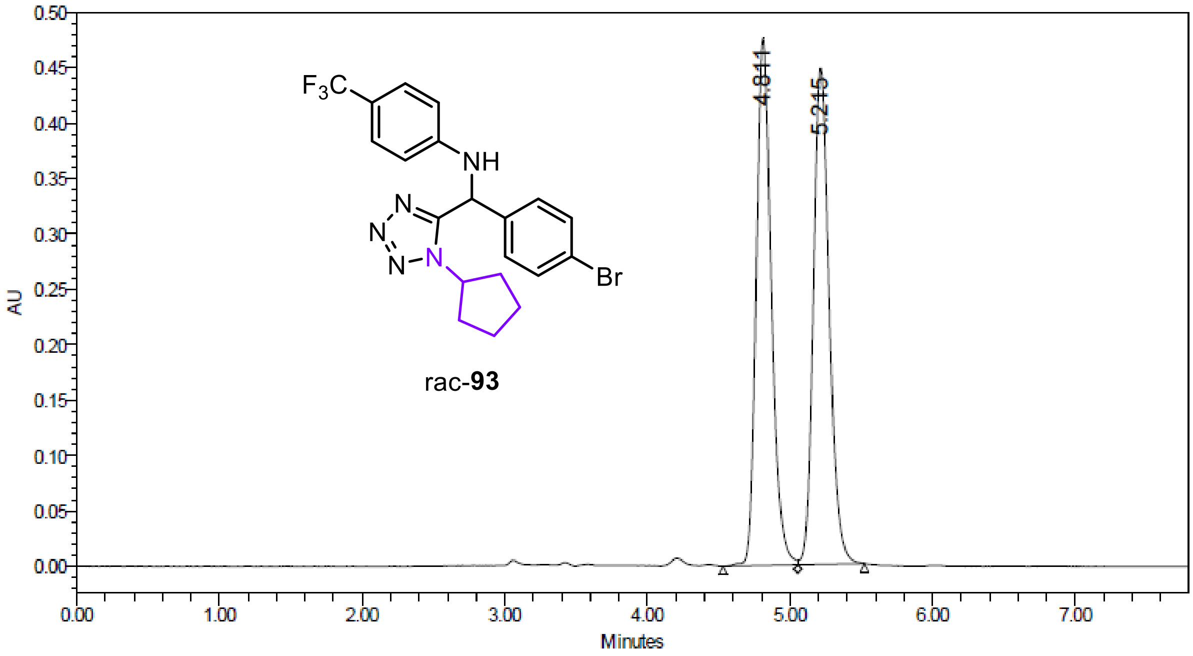

	RT (min)	Peak Type	Area (μV*sec)	% Area	Height (μV)	% Height	Integration Type	Points Across Peak	Start Time (min)	End Time (min)
1	4.811	Unknown	3497699	50.31	475560	51.51	BV	314	4.530	5.053
2	5.215	Unknown	3455129	49.69	447646	48.49	VB	281	5.053	5.522

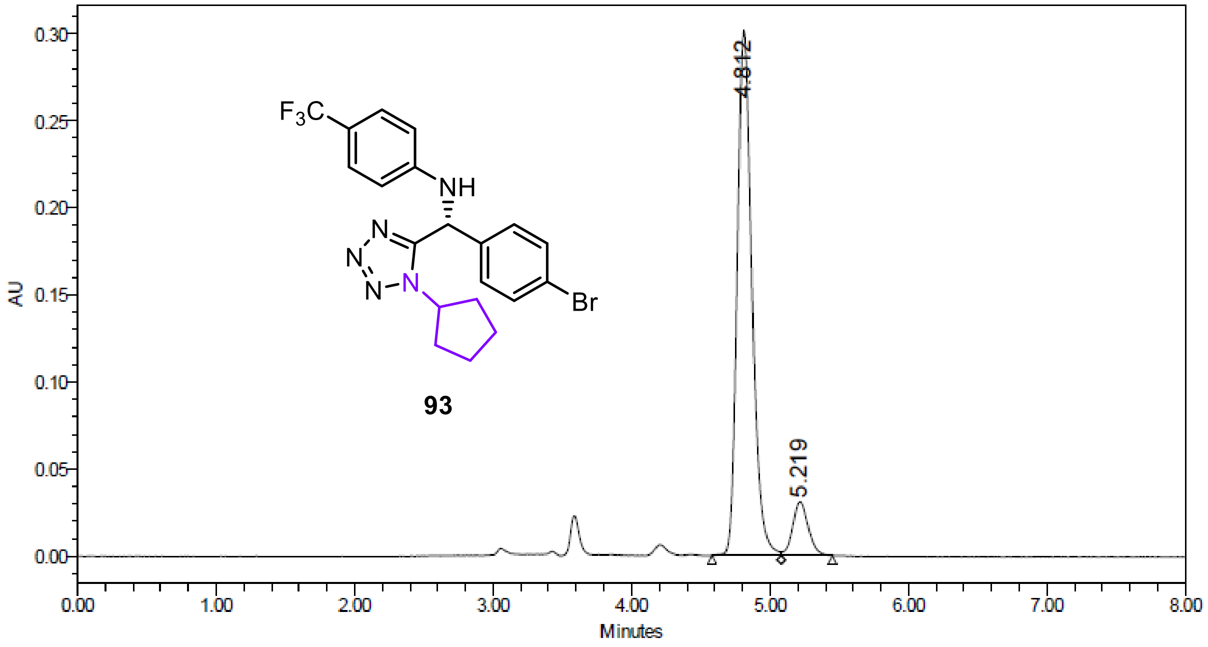

	RT (min)	Peak Type	Area (μV*sec)	% Area	Height (μV)	% Height	Integration Type	Points Across Peak	Start Time (min)	End Time (min)
1	4.812	Unknown	2223370	90.15	301408	90.68	BV	300	4.582	5.082
2	5.219	Unknown	242889	9.85	30979	9.32	VB	222	5.082	5.452

Supplementary Fig. 351. HPLC of product **93**.

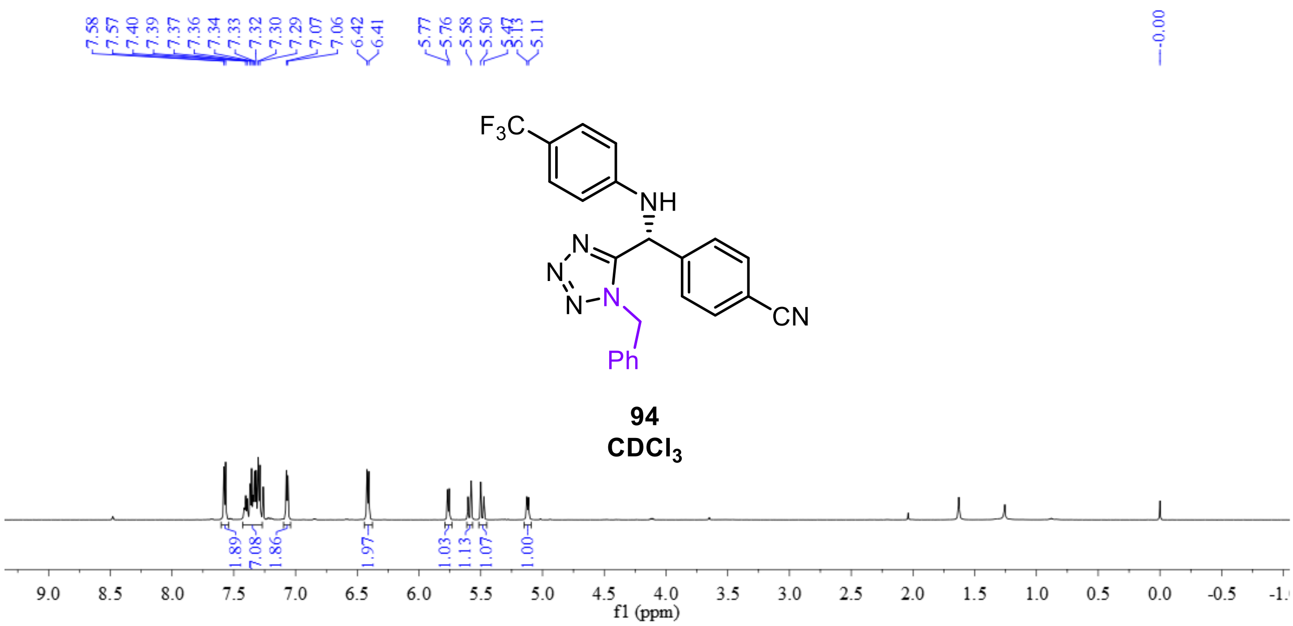

Supplementary Fig. 352. ^{1}H NMR spectrum of **94**. The sample has been recorded in 600 MHz, $CDCl_3$ at 25 °C.

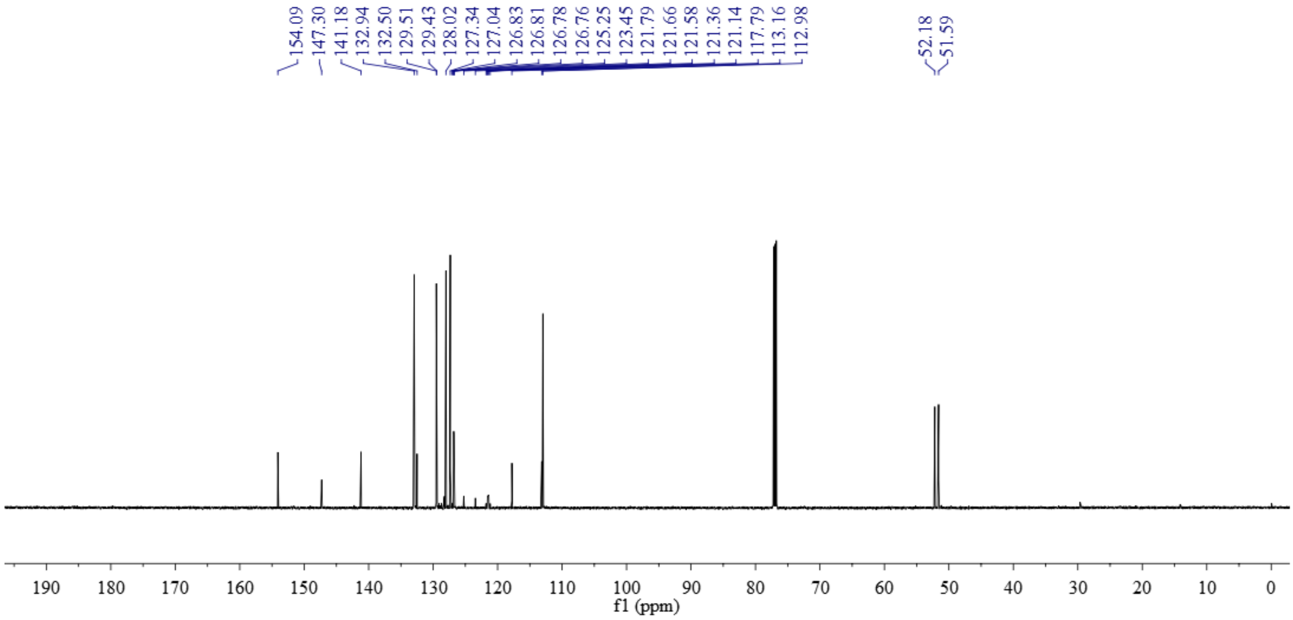

Supplementary Fig. 353. ^{13}C NMR spectrum of **94**. The sample has been recorded in 151 MHz, $CDCl_3$ at 25 °C.

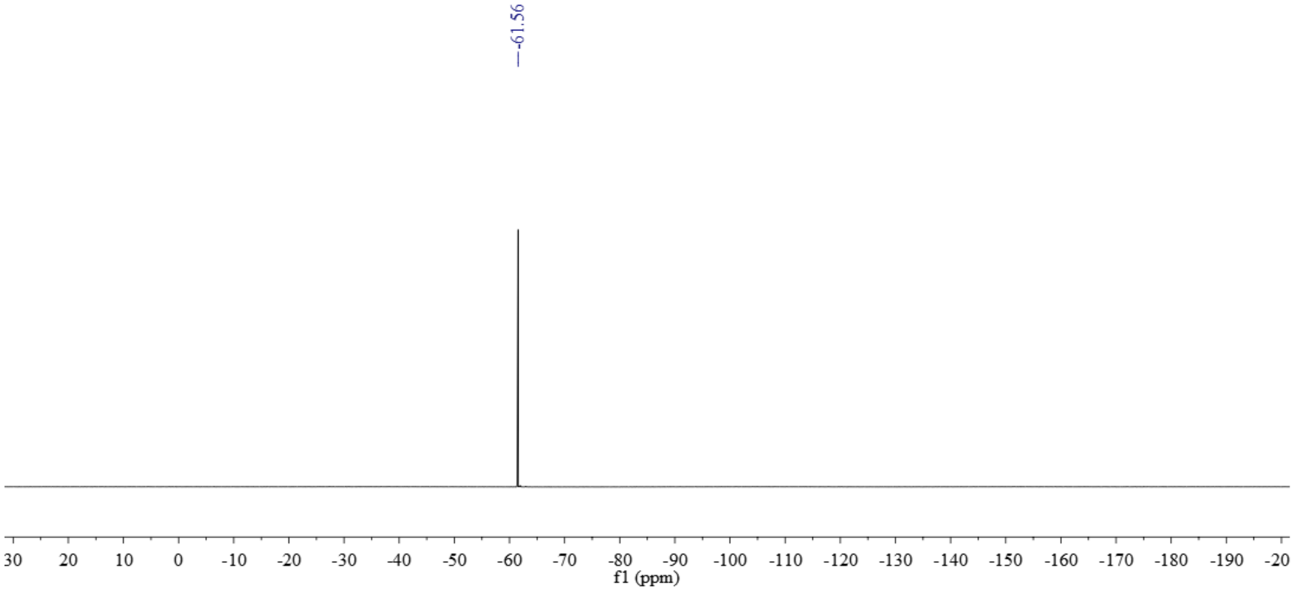

Supplementary Fig. 354. ^{31}F NMR spectrum of **94**. The sample has been recorded in 564 MHz, $CDCl_3$ at 25 °C.

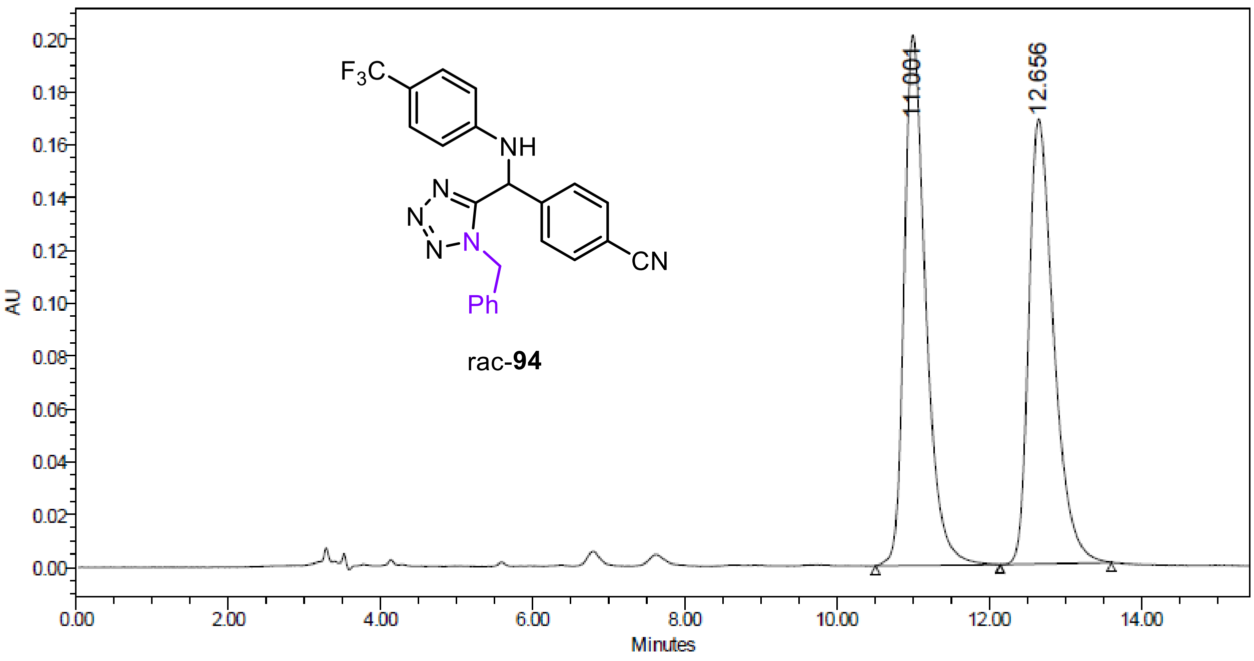

	RT (min)	Area (μV*sec)	% Area	Height (μV)	% Height
1	11.001	3956602	50.78	200747	54.37
2	12.656	3834675	49.22	168446	45.63

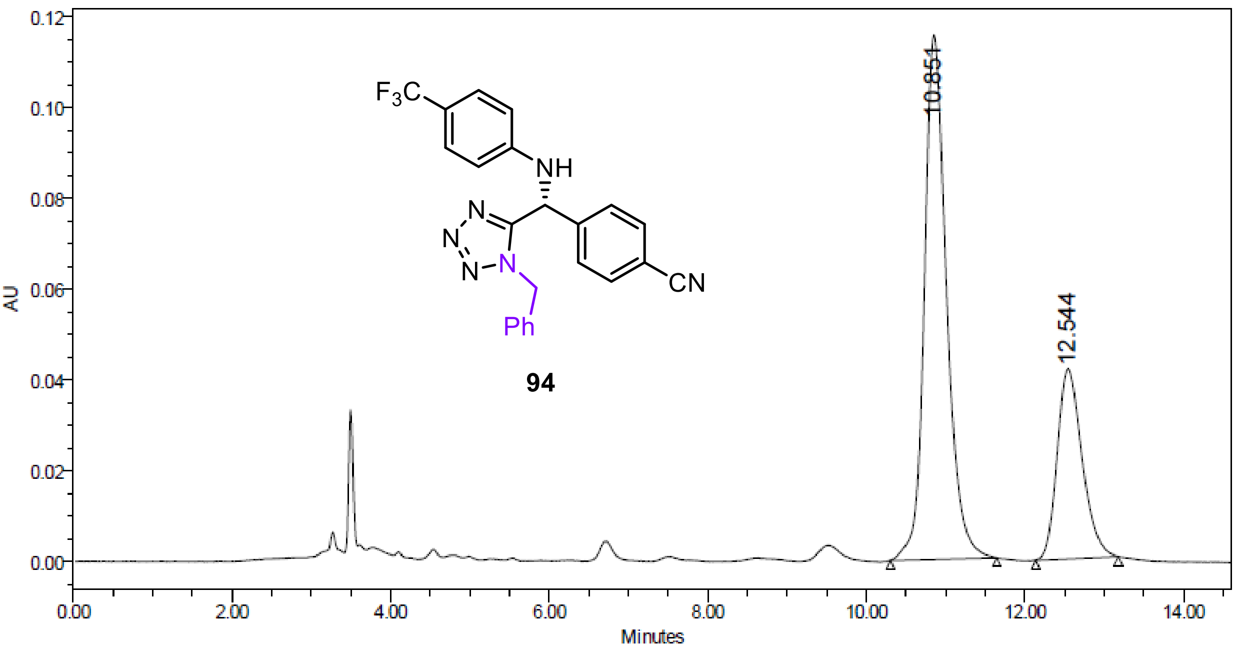

	RT (min)	Area (μV*sec)	% Area	Height (μV)	% Height
1	10.851	2241193	71.02	115551	73.42
2	12.544	914684	28.98	41840	26.58

Supplementary Fig. 355. HPLC of product **94**.

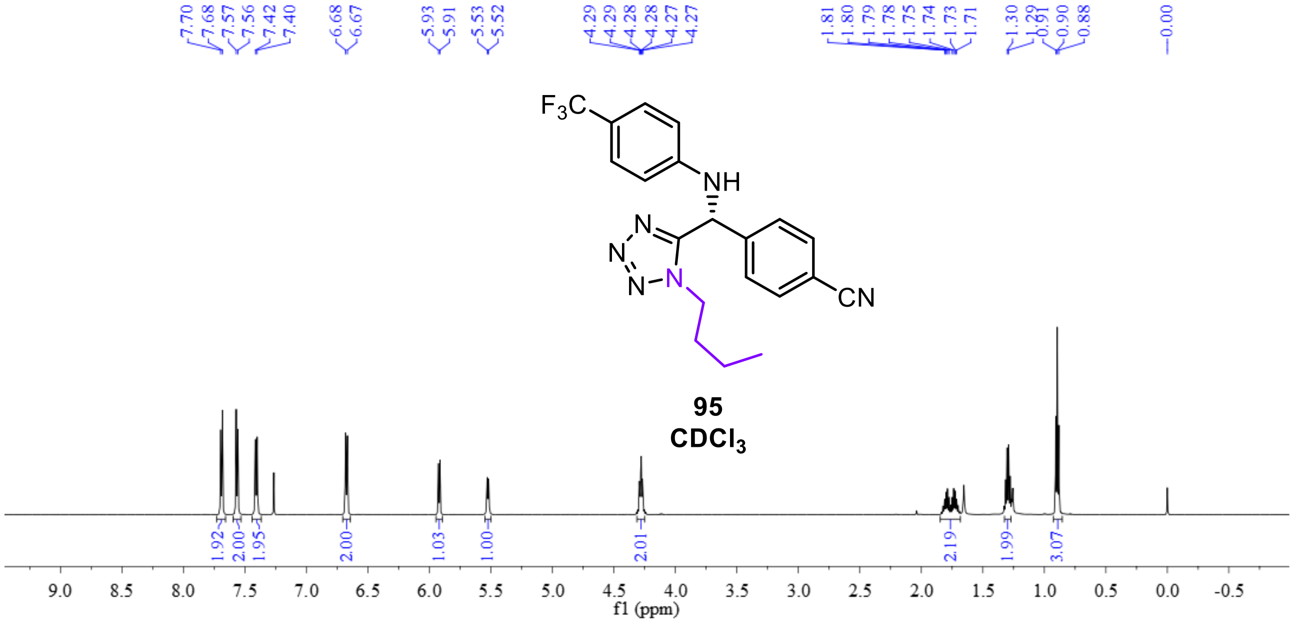

Supplementary Fig. 356. ^{1}H NMR spectrum of **95**. The sample has been recorded in 600 MHz, $CDCl_3$ at 25 °C.

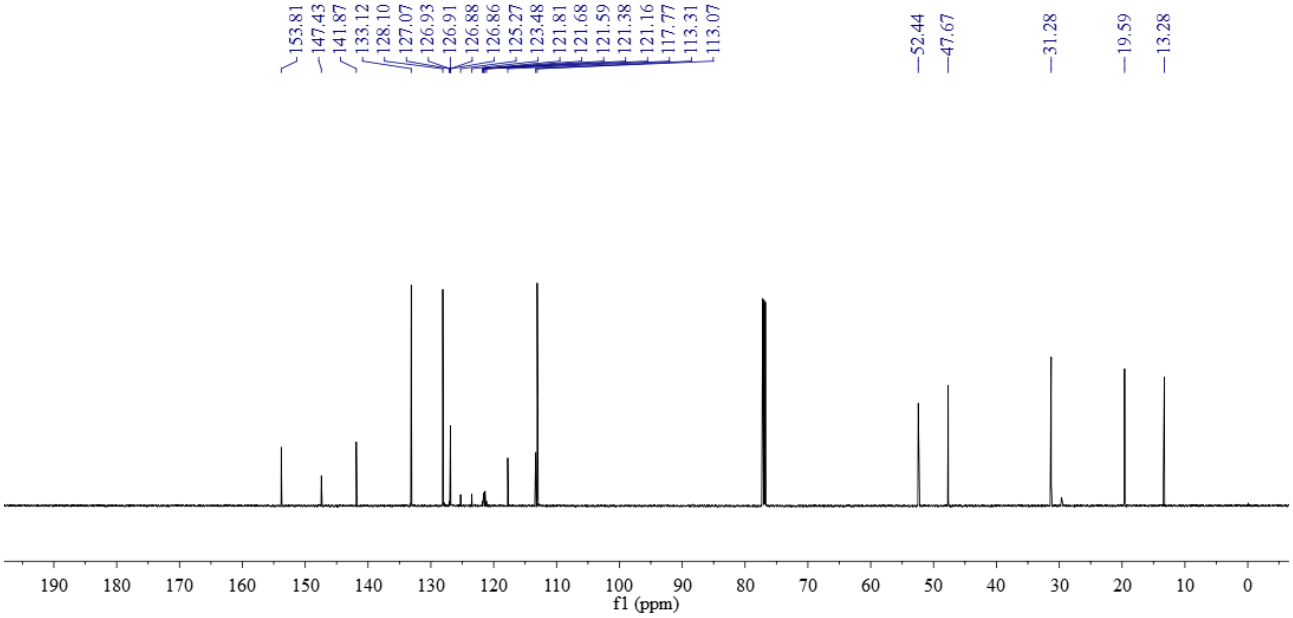

Supplementary Fig. 357. ^{13}C NMR spectrum of **95**. The sample has been recorded in 151 MHz, $CDCl_3$ at 25 °C.

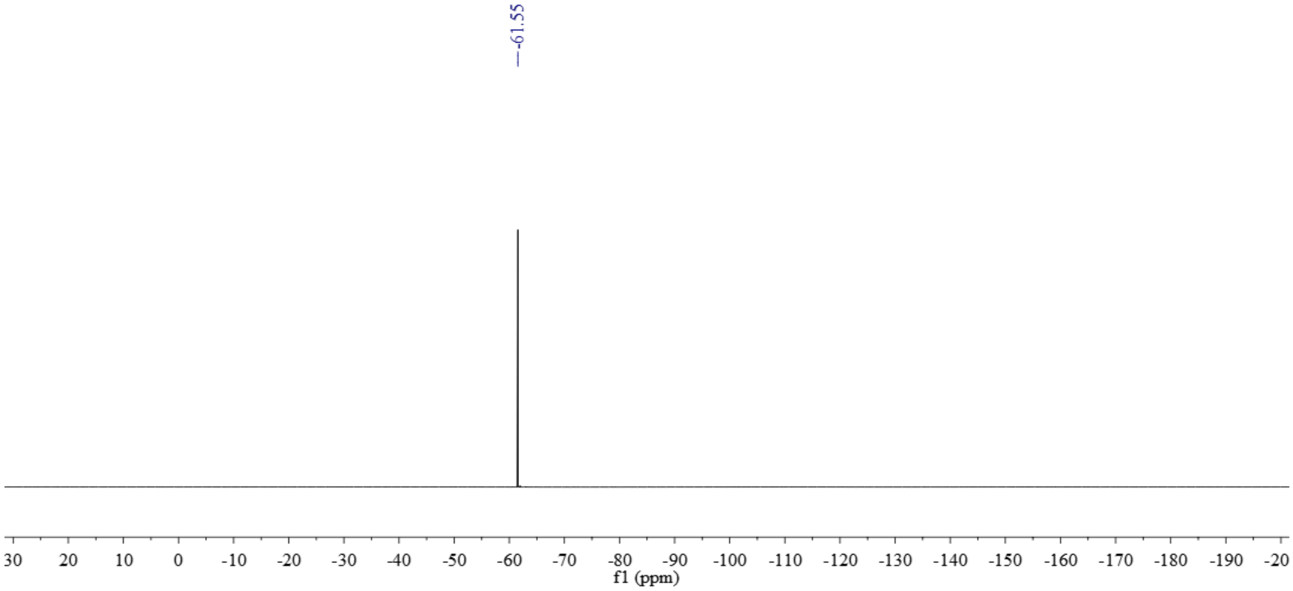

Supplementary Fig. 358. ^{31}F NMR spectrum of **95**. The sample has been recorded in 564 MHz, $CDCl_3$ at 25 °C.

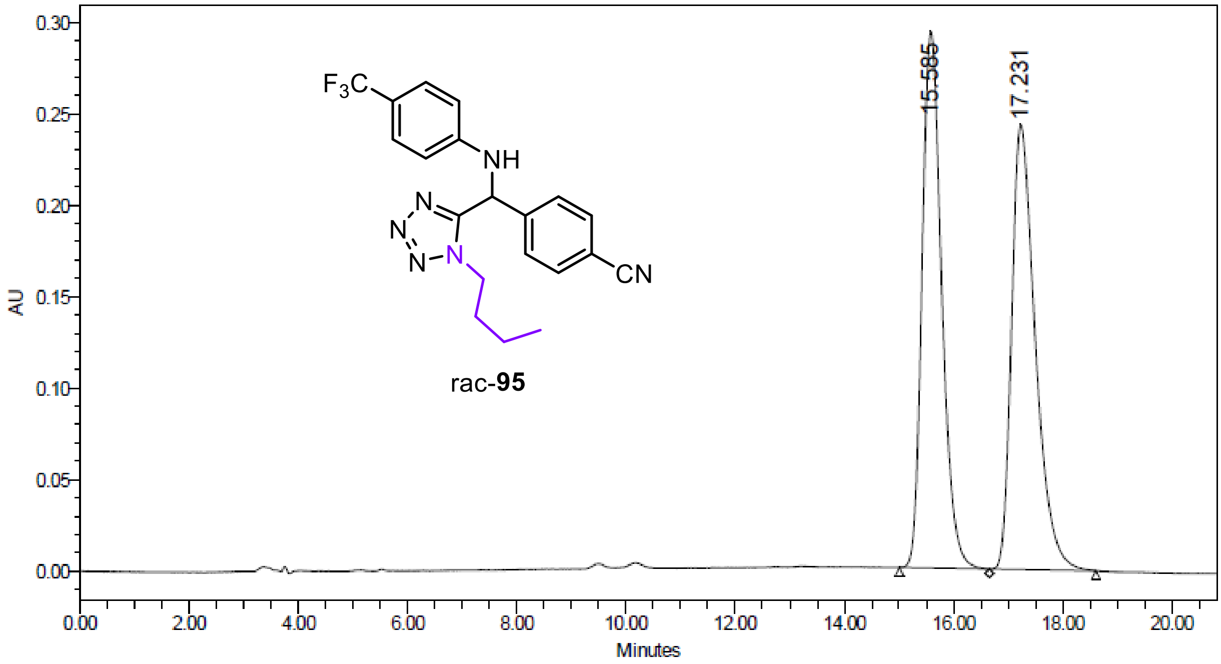

	RT (min)	Peak Type	Area (µV*sec)	% Area	Height (µV)	% Height	Integration Type	Points Across Peak	Start Time (min)	End Time (min)
1	15.585	Unknown	7302073	49.92	293172	54.67	BV	989	15.010	16.658
2	17.231	Unknown	7325933	50.08	243066	45.33	Vb	1169	16.658	18.607

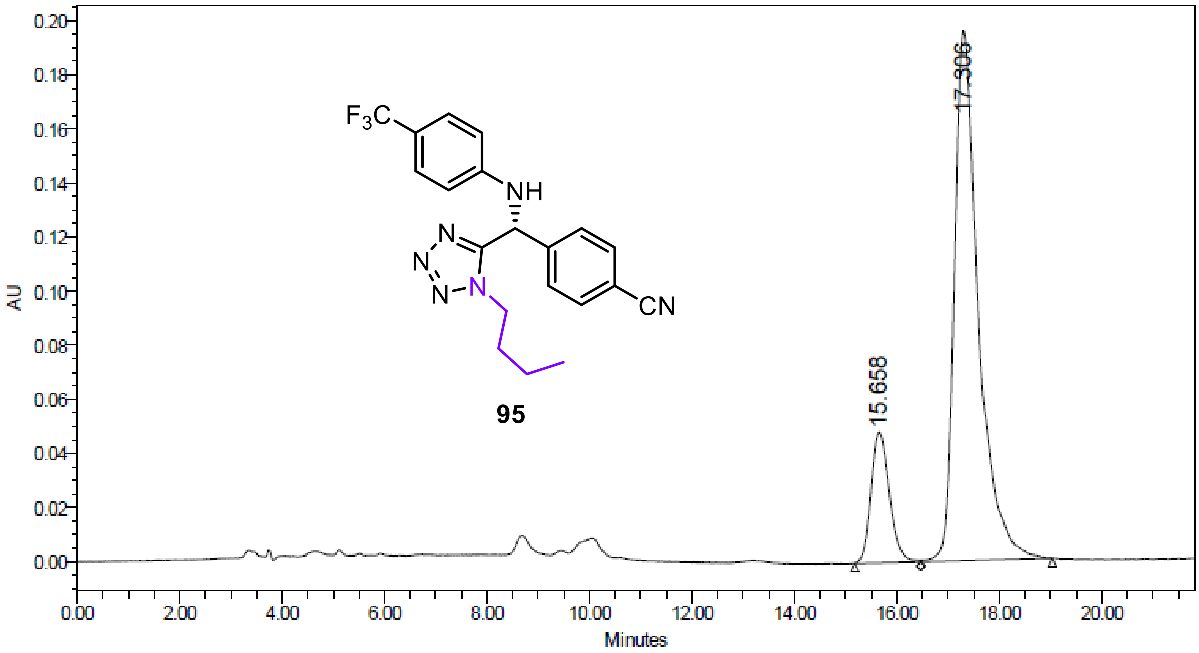

	RT (min)	Peak Type	Area (µV*sec)	% Area	Height (µV)	% Height	Integration Type	Points Across Peak	Start Time (min)	End Time (min)
1	15.658	Unknown	1204376	15.93	48273	19.78	BV	773	15.178	16.467
2	17.306	Unknown	6357299	84.07	195756	80.22	VB	1538	16.467	19.030

Supplementary Fig. 359. HPLC of product **95**.

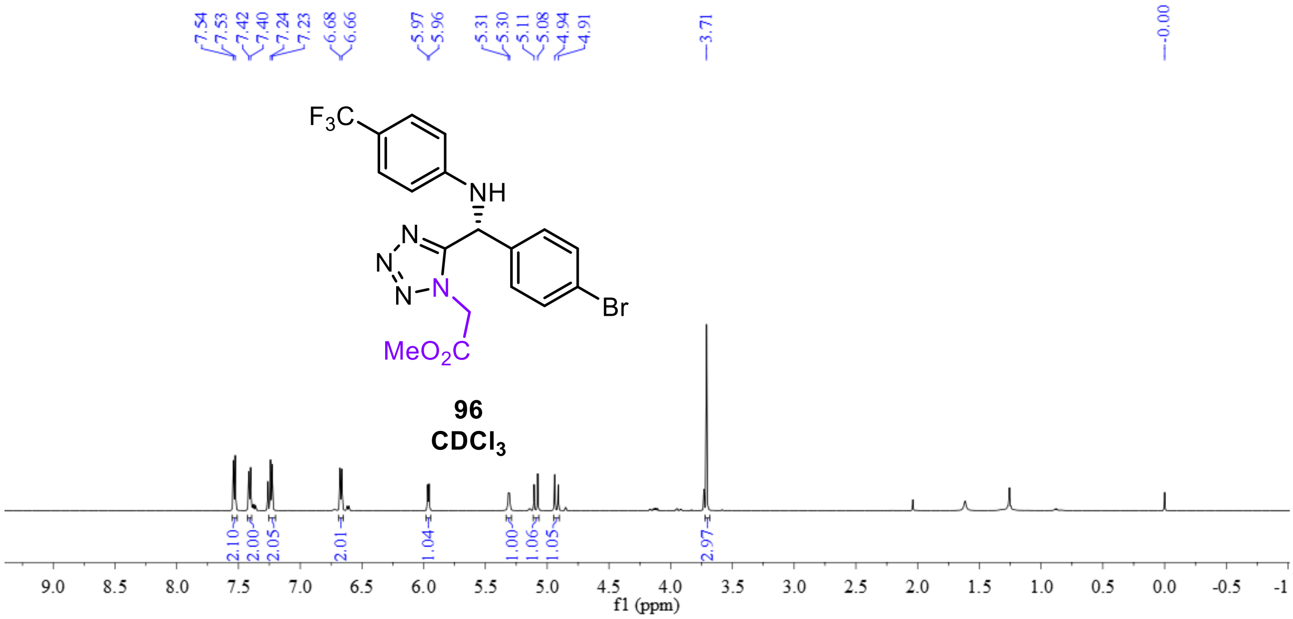

Supplementary Fig. 360. ^{1}H NMR spectrum of **96**. The sample has been recorded in 600 MHz, $CDCl_3$ at 25 °C.

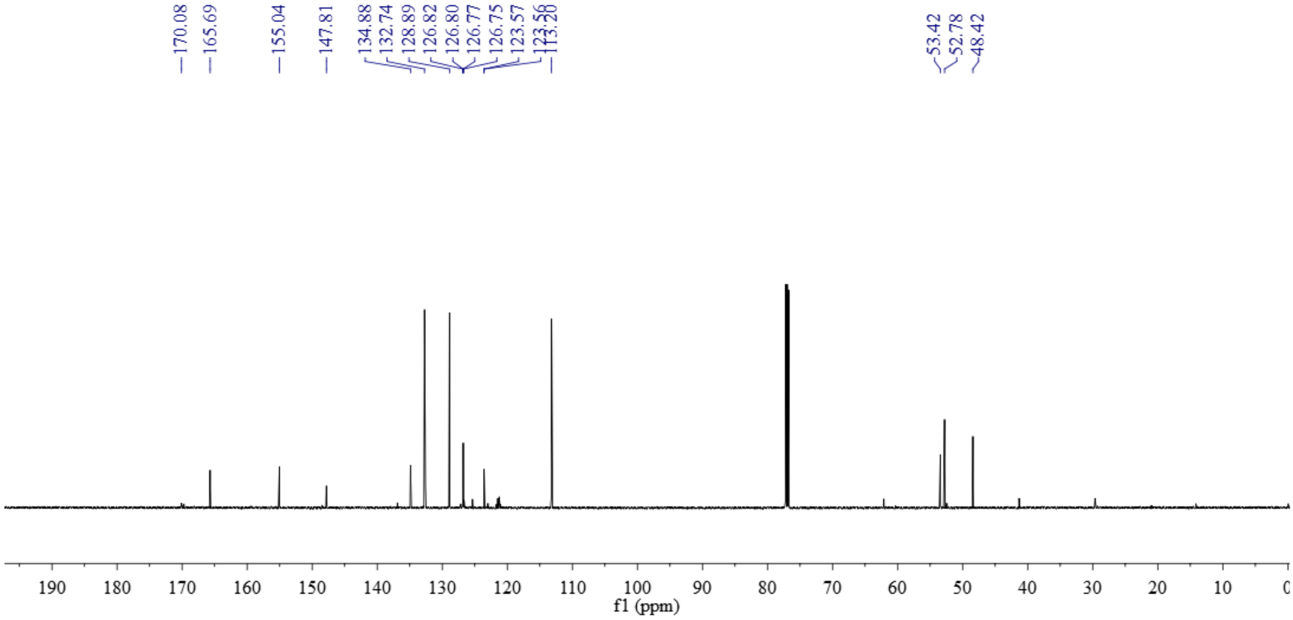

Supplementary Fig. 361. ^{13}C NMR spectrum of **96**. The sample has been recorded in 151 MHz, $CDCl_3$ at 25 °C.

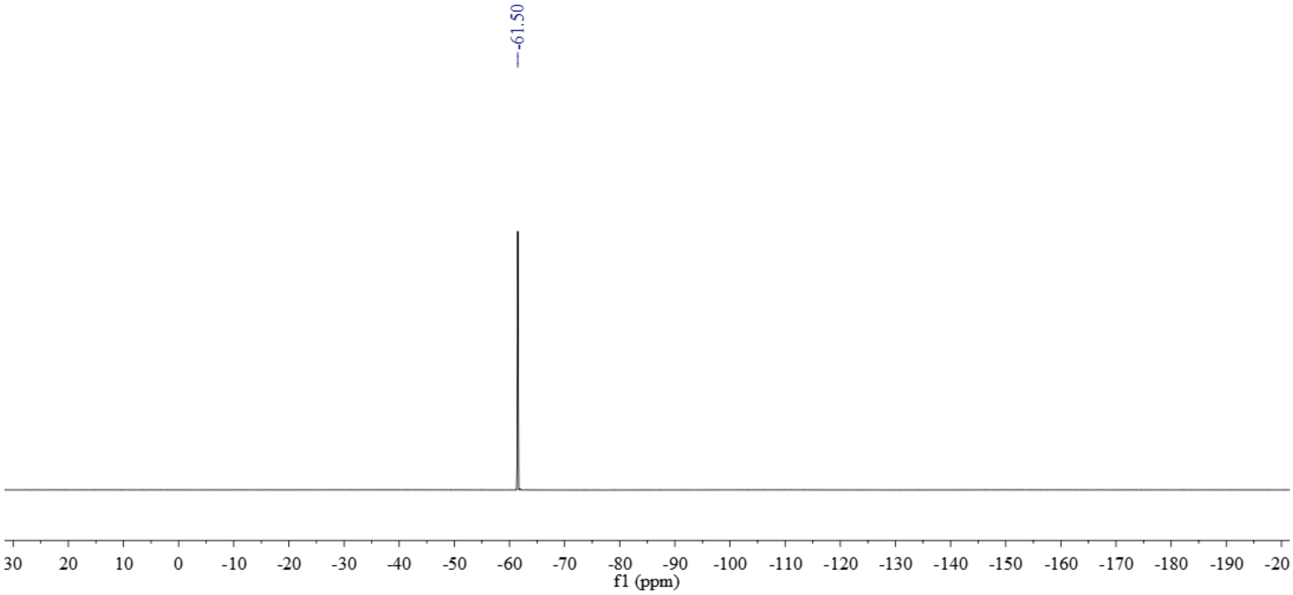

Supplementary Fig. 362. ^{31}F NMR spectrum of **96**. The sample has been recorded in 564 MHz, $CDCl_3$ at 25 °C.

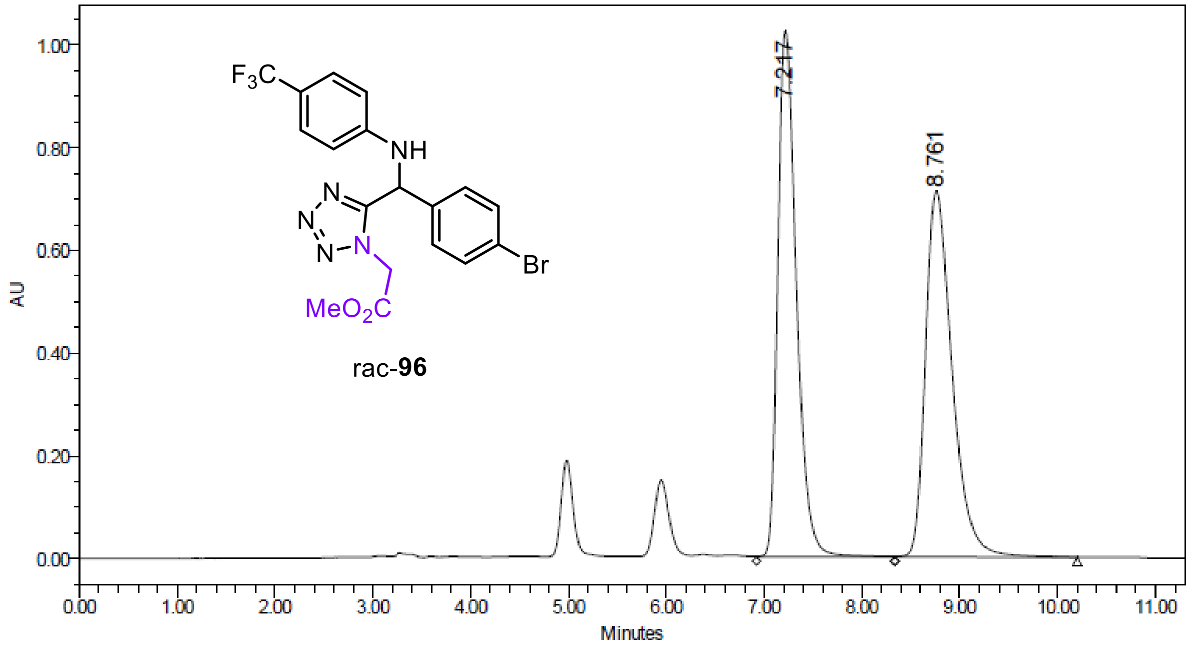

	RT (min)	Peak Type	Area (μV*sec)	% Area	Height (μV)	% Height	Integration Type	Points Across Peak	Start Time (min)	End Time (min)
1	7.217	Unknown	13528806	50.04	1024108	58.96	VV	848	6.923	8.337
2	8.761	Unknown	13507819	49.96	712707	41.04	VB	1119	8.337	10.202

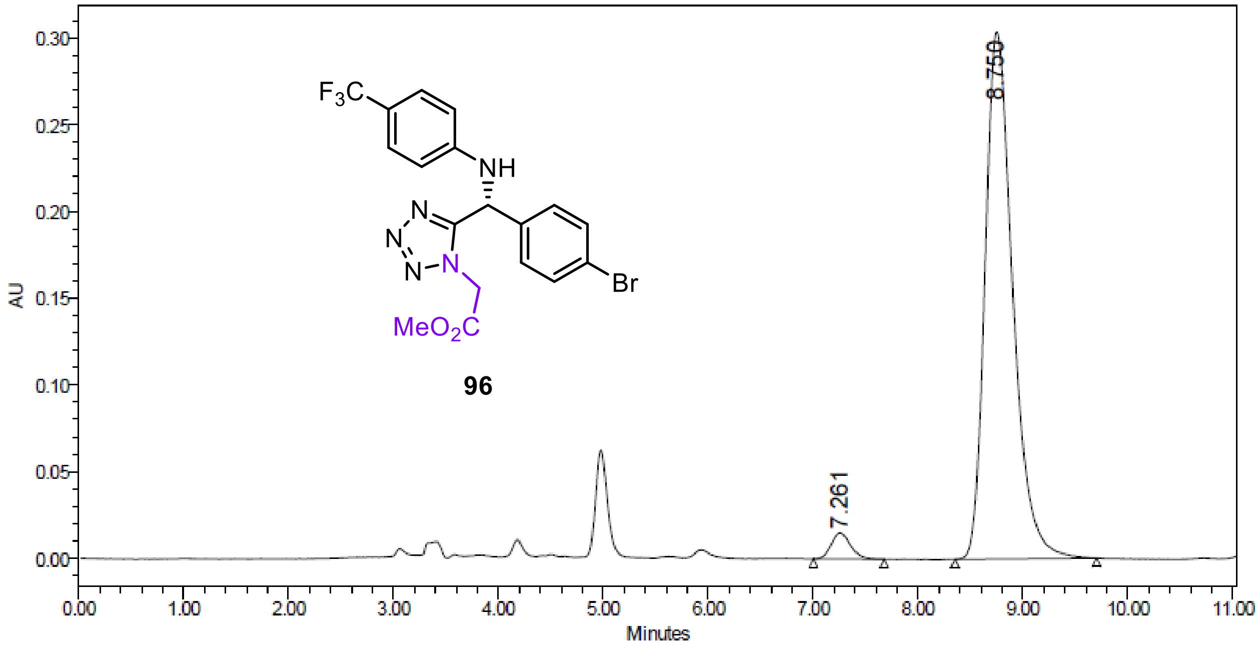

	RT (min)	Area (μV*sec)	% Area	Height (μV)	% Height
1	7.261	192390	3.34	15055	4.72
2	8.750	5560793	96.66	303915	95.28

Supplementary Fig. 363. HPLC of product **96**.

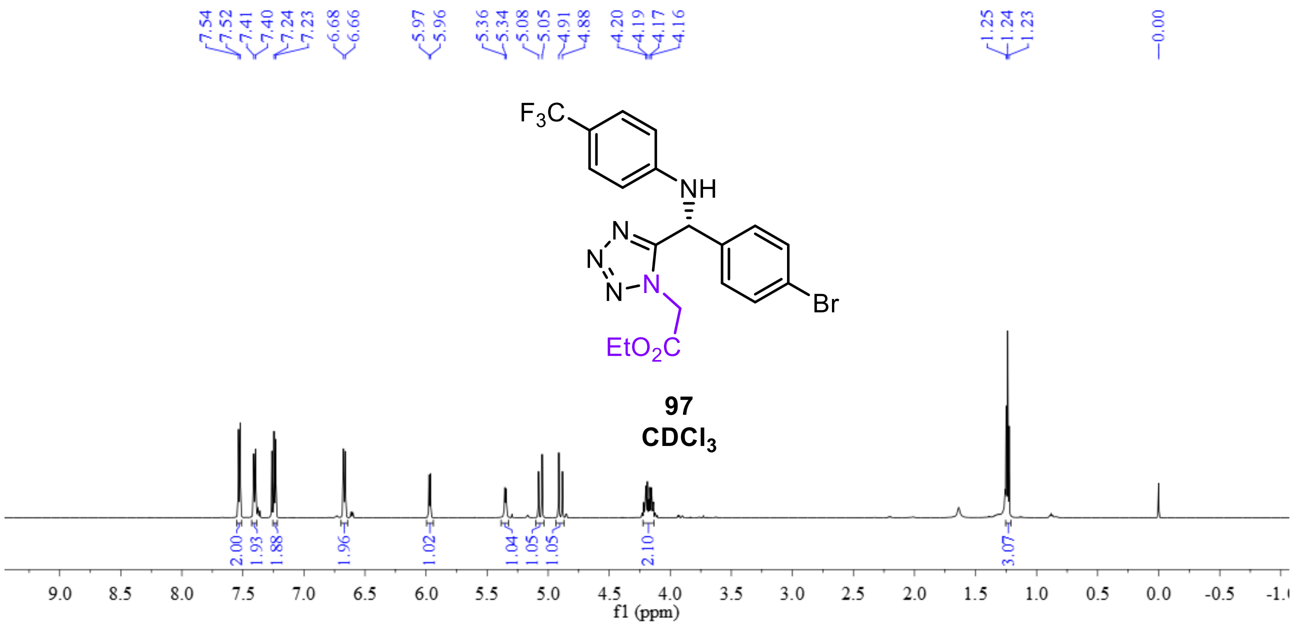

Supplementary Fig. 364. ^{1}H NMR spectrum of **97**. The sample has been recorded in 600 MHz, $CDCl_3$ at 25 °C.

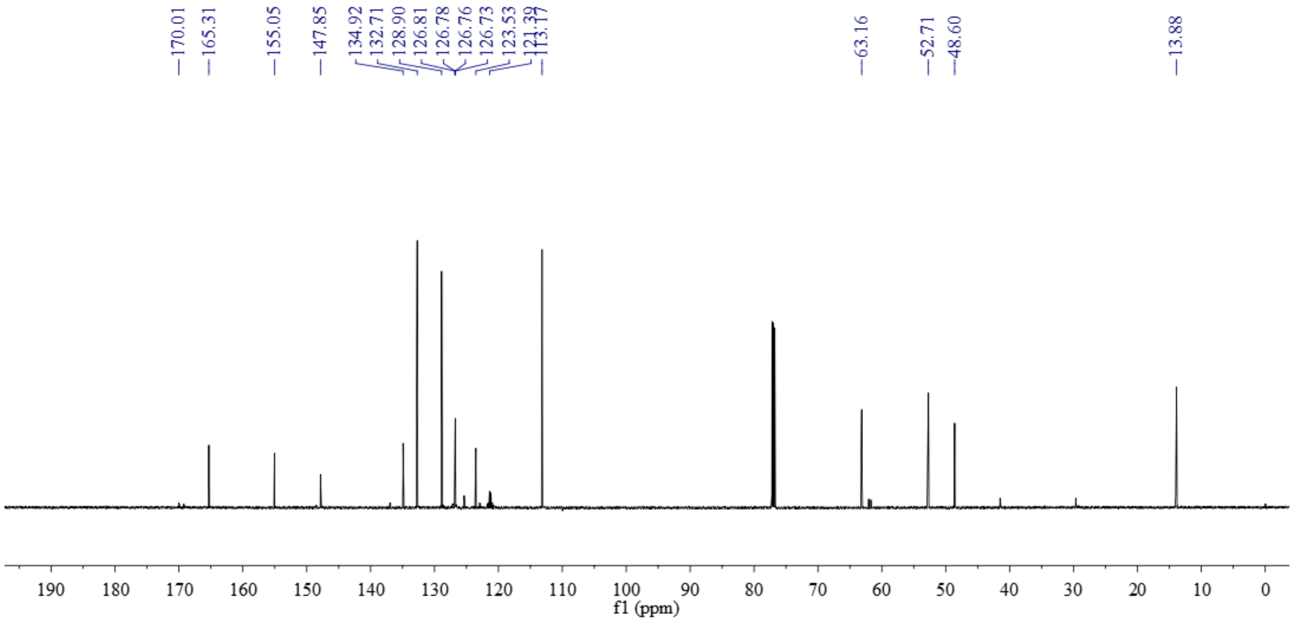

Supplementary Fig. 365. ^{13}C NMR spectrum of **97**. The sample has been recorded in 151 MHz, $CDCl_3$ at 25 °C.

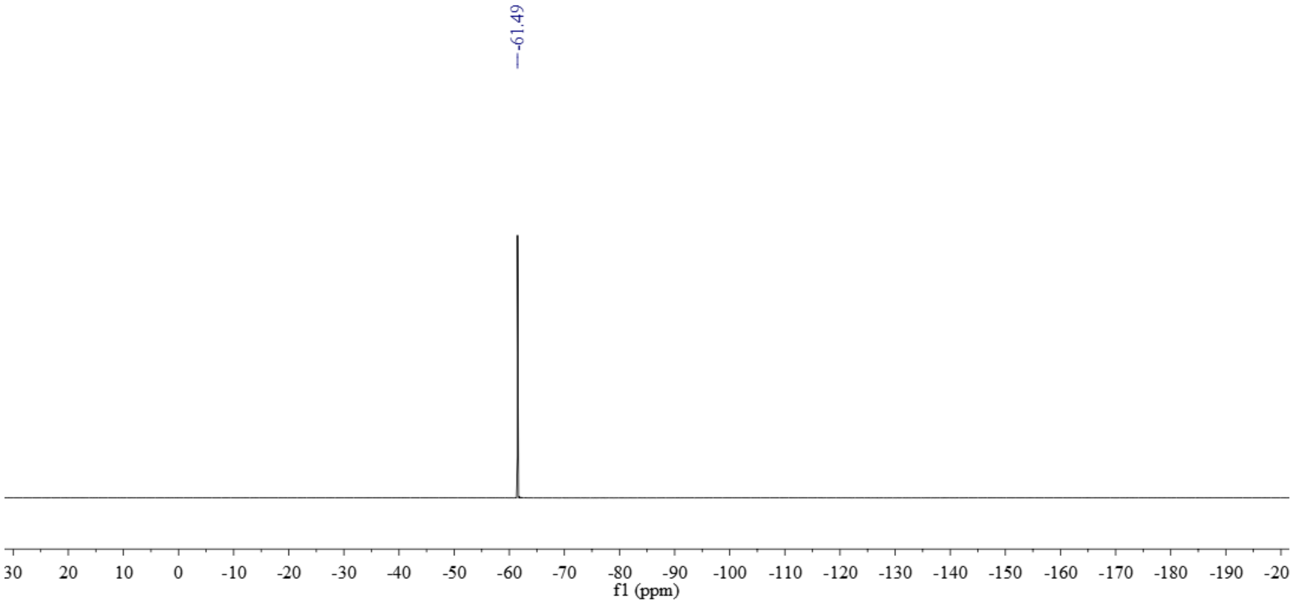

Supplementary Fig. 366. ^{31}F NMR spectrum of **97**. The sample has been recorded in 564 MHz, $CDCl_3$ at 25 °C.

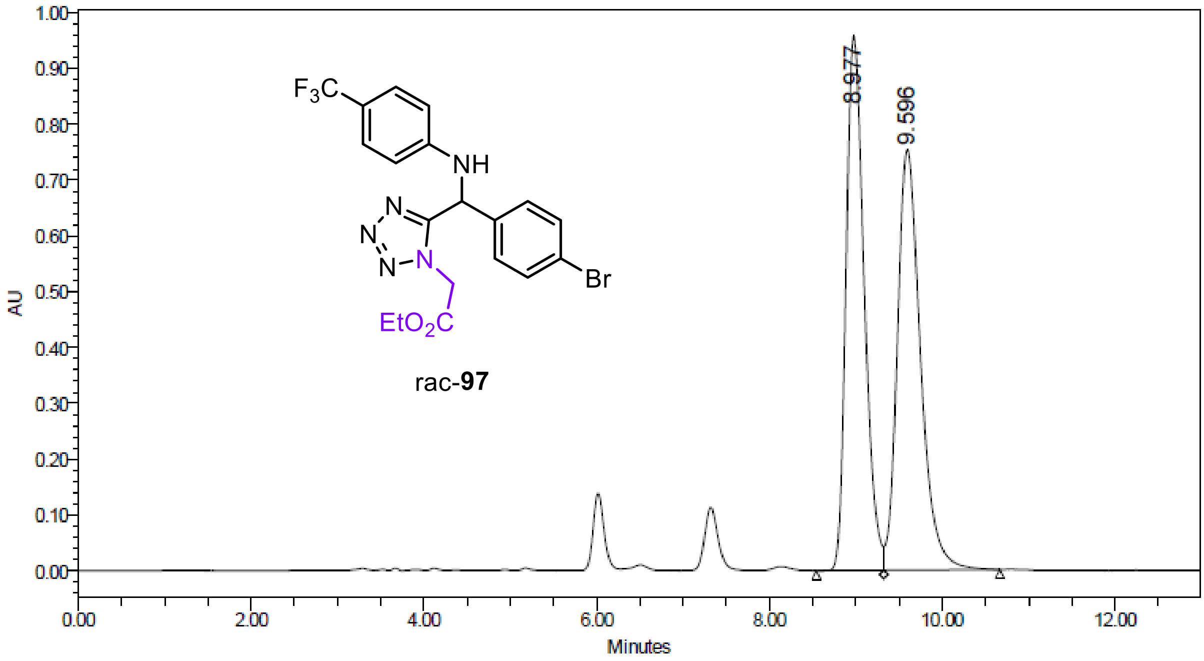

	RT (min)	Peak Type	Area (μV*sec)	% Area	Height (μV)	% Height	Integration Type	Points Across Peak	Start Time (min)	End Time (min)
1	8.977	Unknown	13960666	49.78	958128	55.95	BV	467	8.543	9.322
2	9.596	Unknown	14083163	50.22	754384	44.05	VB	806	9.322	10.665

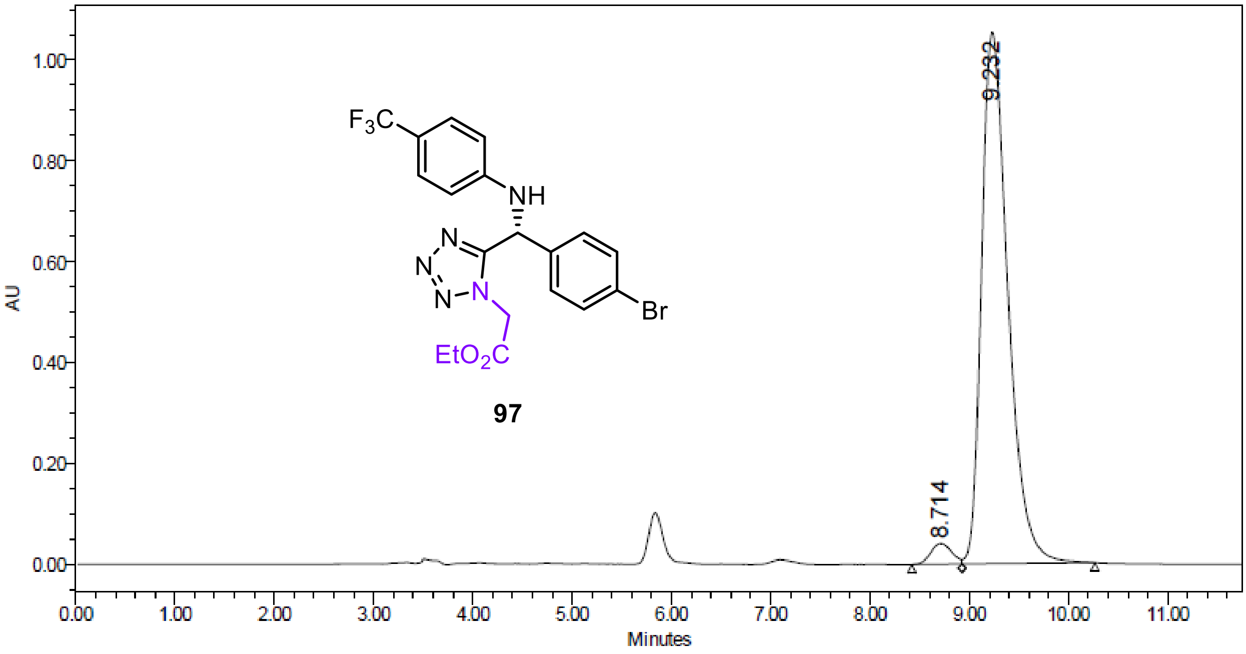

	RT (min)	Area (μV*sec)	% Area	Height (μV)	% Height
1	8.714	604155	3.02	41262	3.77
2	9.232	19409641	96.98	1054089	96.23

Supplementary Fig. 367. HPLC of product **97**.

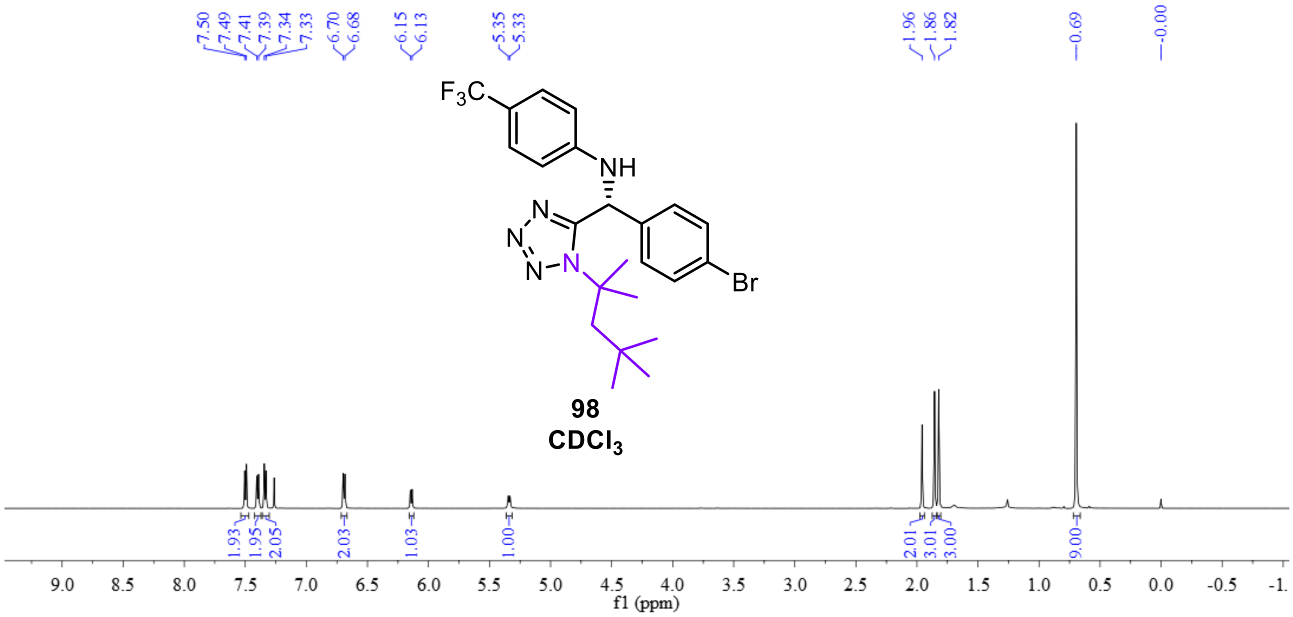

Supplementary Fig. 368. ^{1}H NMR spectrum of **98**. The sample has been recorded in 600 MHz, $CDCl_3$ at 25 °C.

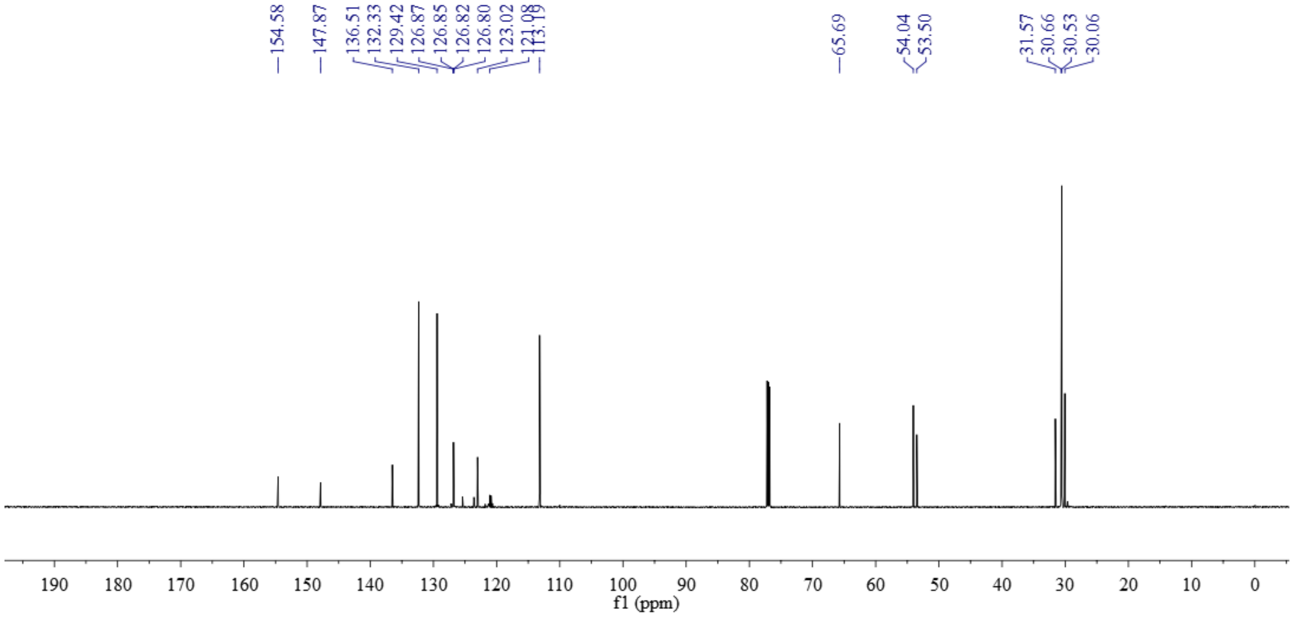

Supplementary Fig. 369. ^{13}C NMR spectrum of **98**. The sample has been recorded in 151 MHz, $CDCl_3$ at 25 °C.

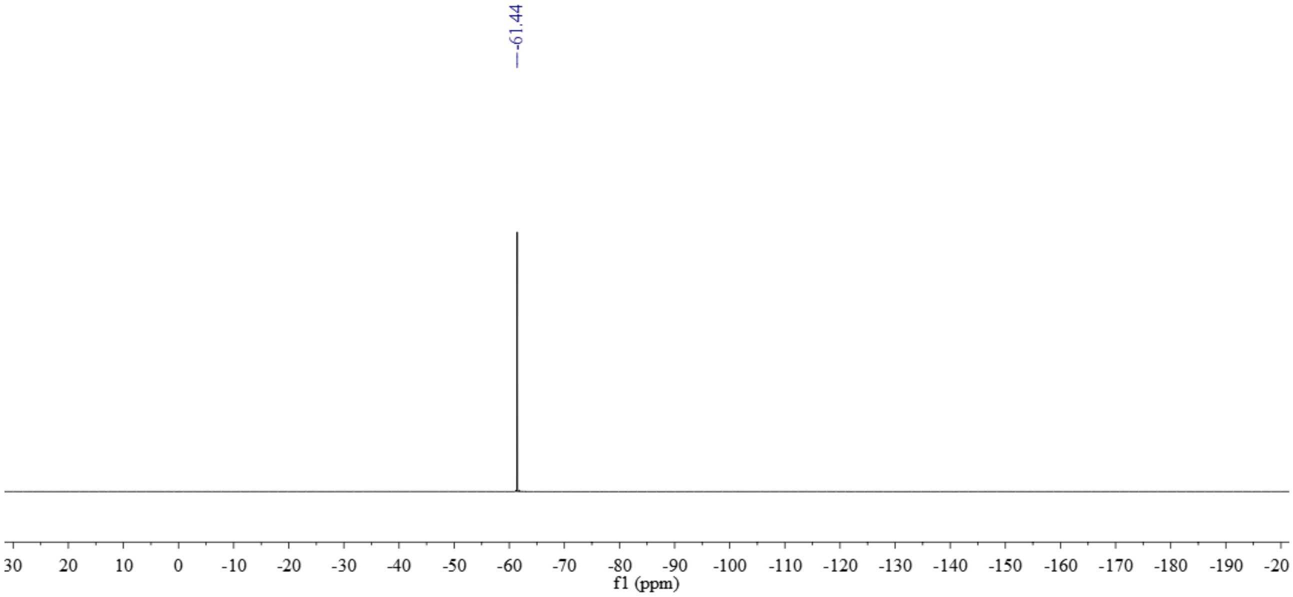

Supplementary Fig. 370. [31]F NMR spectrum of **98**. The sample has been recorded in 564 MHz, $CDCl_3$ at 25 °C.

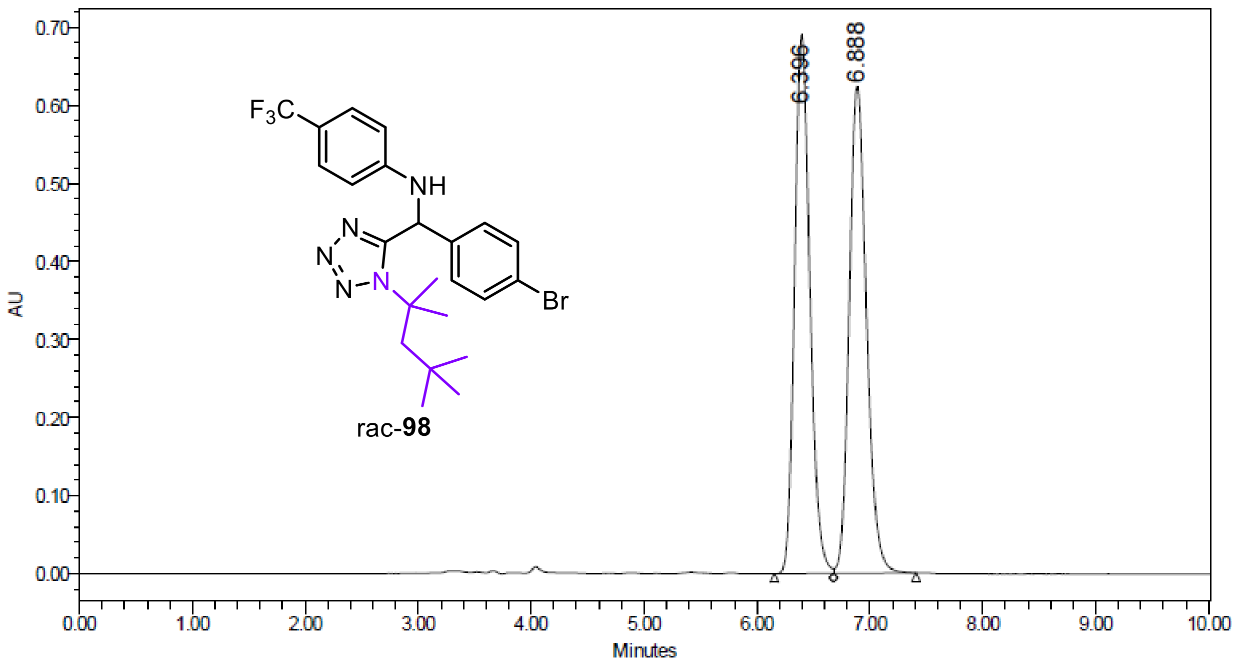

	RT (min)	Peak Type	Area (µV*sec)	% Area	Height (µV)	% Height	Integration Type	Points Across Peak	Start Time (min)	End Time (min)
1	6.396	Unknown	6520006	49.67	689884	52.53	BV	314	6.153	6.677
2	6.888	Unknown	6606968	50.33	623376	47.47	VB	439	6.677	7.408

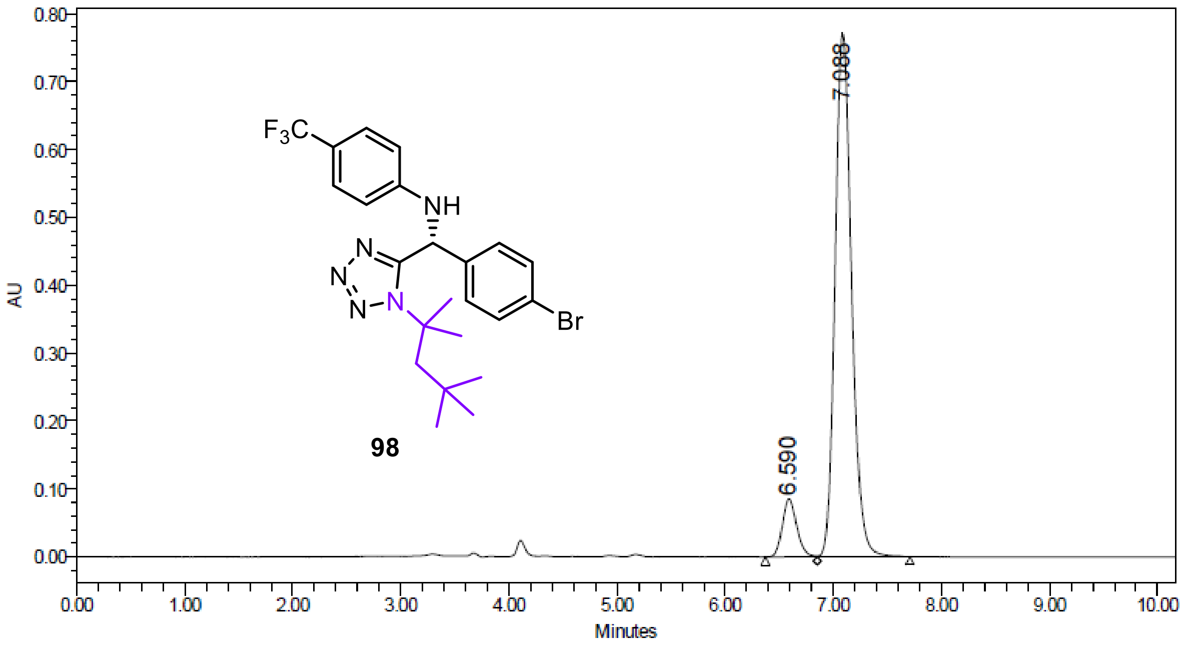

	RT (min)	Peak Type	Area (µV*sec)	% Area	Height (µV)	% Height	Integration Type	Points Across Peak	Start Time (min)	End Time (min)
1	6.590	Unknown	786902	8.81	85270	9.96	BV	287	6.372	6.850
2	7.088	Unknown	8149541	91.19	770562	90.04	VB	515	6.850	7.708

Supplementary Fig. 371. HPLC of product **98**.

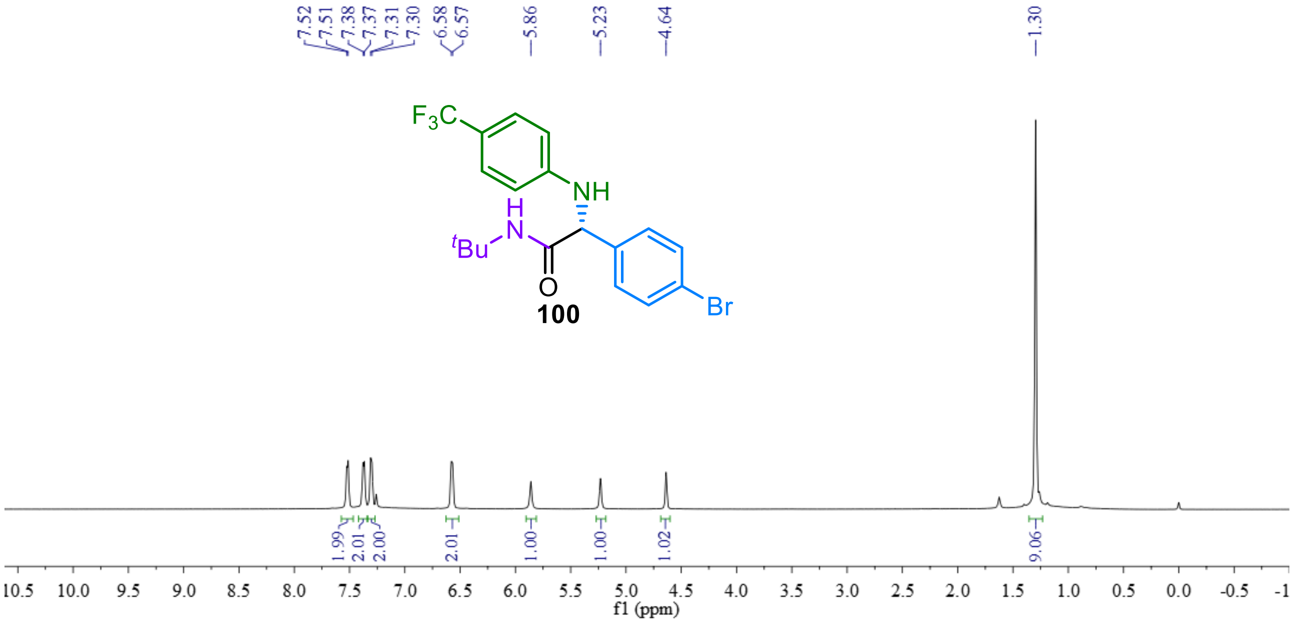

Supplementary Fig. 372. ¹H NMR spectrum of **100**. The sample has been recorded in 600 MHz, $CDCl_3$ at 25 °C.

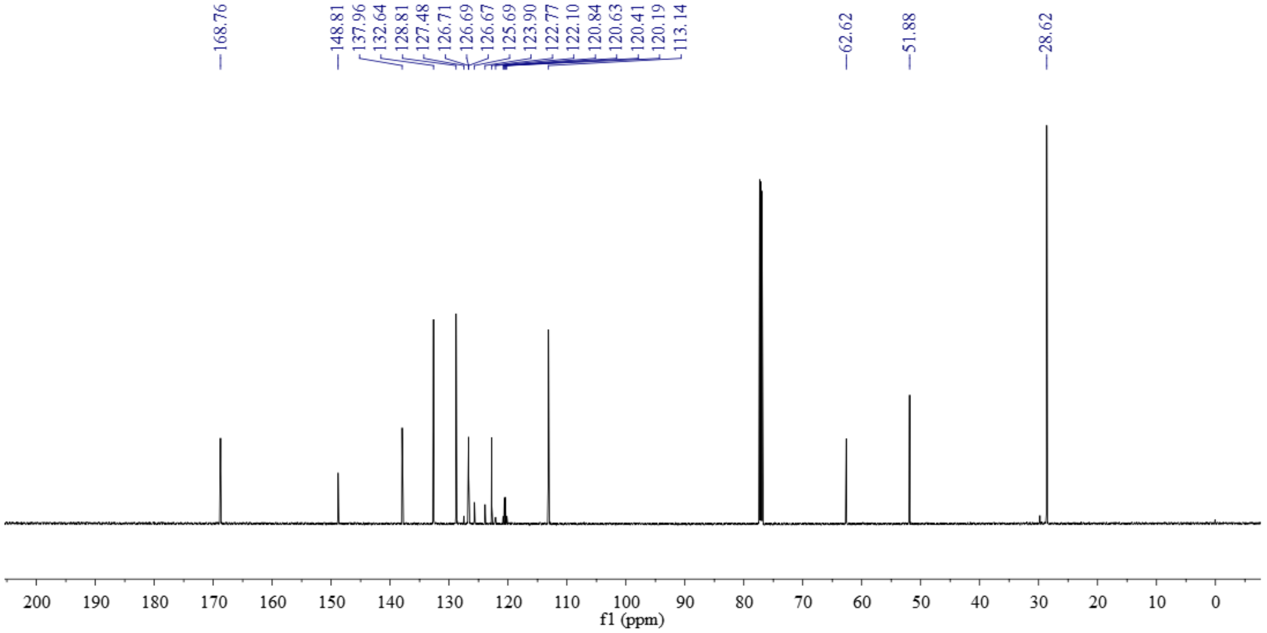

Supplementary Fig. 373. ¹³C NMR spectrum of **100**. The sample has been recorded in 151 MHz, $CDCl_3$ at 25 °C.

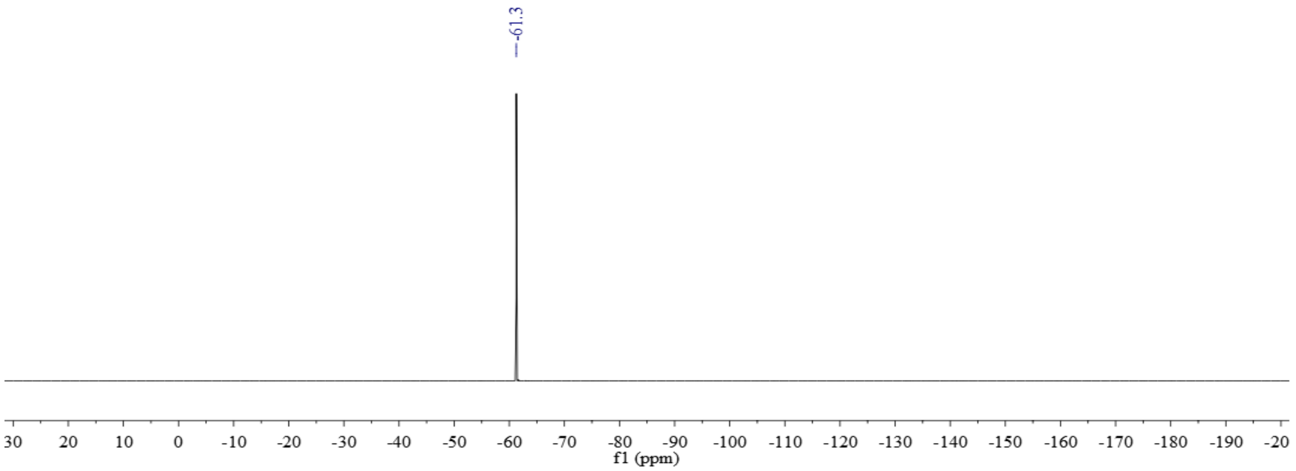

Supplementary Fig. 374. ³¹F NMR spectrum of **100**. The sample has been recorded in 564 MHz, $CDCl_3$ at 25 °C.

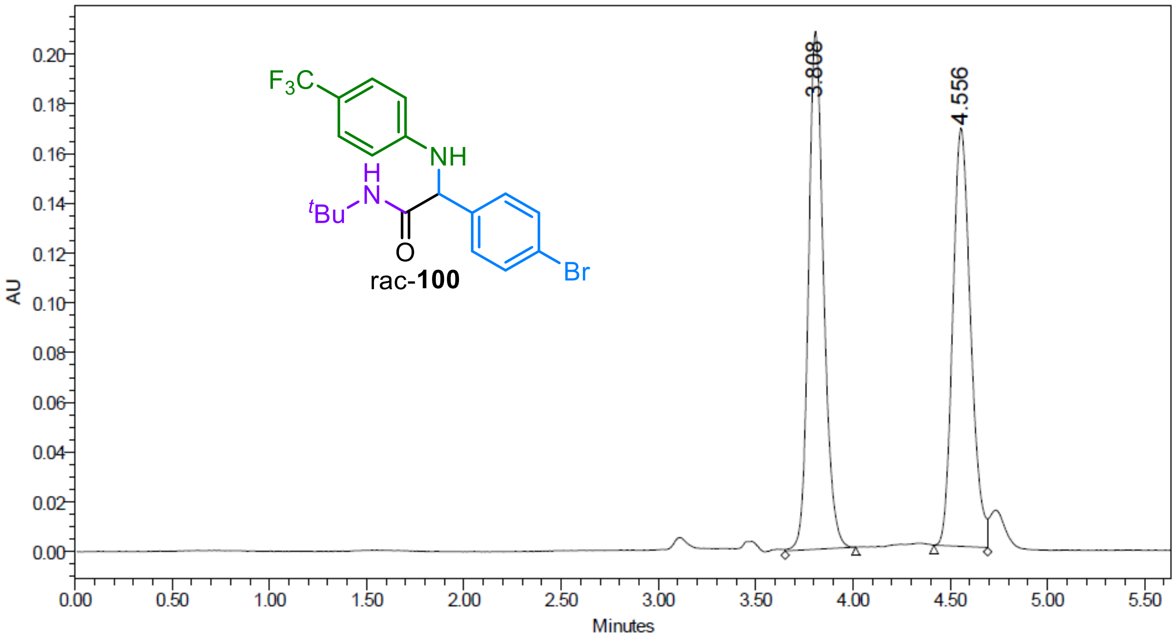

	RT (min)	Peak Type	Area (µV*sec)	% Area	Height (µV)	% Height	Integration Type	Points Across Peak	Start Time (min)	End Time (min)
1	3.808	Unknown	1123006	51.05	208274	55.36	VB	218	3.652	4.015
2	4.556	Unknown	1076670	48.95	167945	44.64	BV	166	4.417	4.693

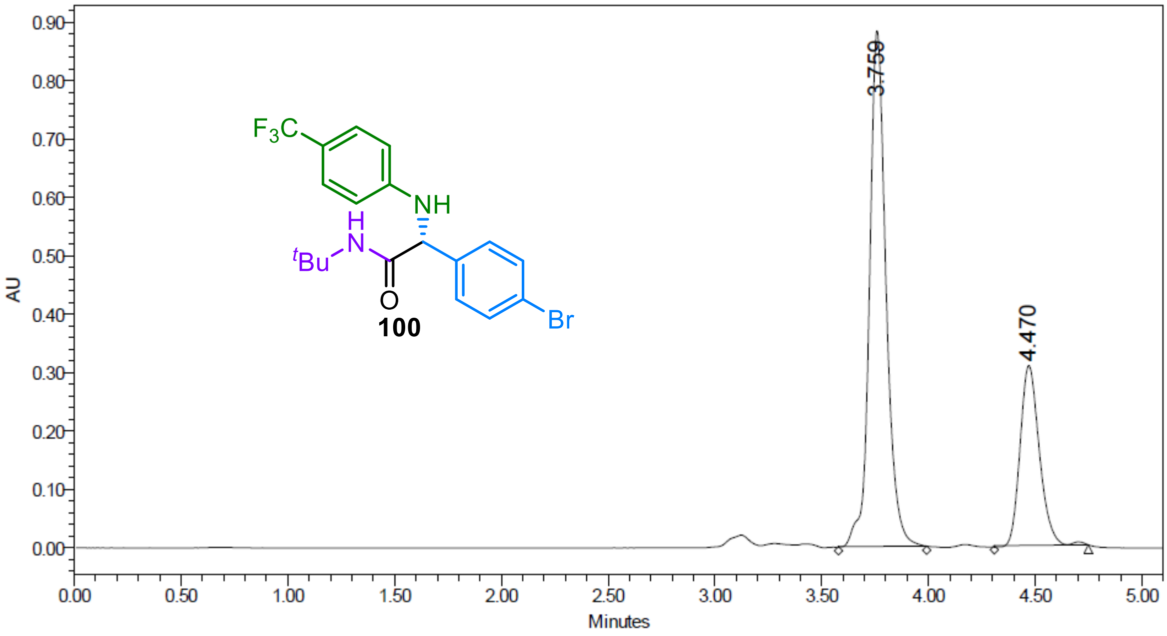

	RT (min)	Peak Type	Area (µV*sec)	% Area	Height (µV)	% Height	Integration Type	Points Across Peak	Start Time (min)	End Time (min)
1	3.759	Unknown	4768324	71.11	883221	74.16	Vv	248	3.578	3.992
2	4.470	Unknown	1937350	28.89	307791	25.84	vb	264	4.308	4.748

Supplementary Fig. 375. HPLC of product **100**.

4. Supplementary References

[1] Yu, J.; Jiang, H.-J.; Zhou, Y.; Luo, S.-W. & Gong, L.-Z. Sodium salts of anionic chiral cobalt(III) complexes as catalysts of the enantioselective Povarov reaction. *Angew. Chem. Int. Ed.* **54**, 11209−11213 (2015).

[2] Jiang, H.-J.; Liu, K.; Yu, J.; Zhang, L. & Gong, L.-Z. Switchable stereoselectivity in bromoaminocyclization of olefins: using Brønsted acids of anionic chiral cobalt(III) complexes. *Angew. Chem. Int. Ed.* **56**, 11931−11935 (2017).

[3] Jiang, H.-J.; Zhong, X.-M.; Yu, J.; Zhang, Y.; Zhang, X.; Wu, Y.-D. & Gong, L.-Z. Assembling a hybrid Pd catalyst from a chiral anionic Co(III) complex and ligand for asymmetric C(sp^3)–H functionalization. *Angew. Chem. Int. Ed.* **58**, 1803−1807 (2019).

[4] Wu, X.-B.; Gao, Q.; Fan, J.-J.; Zhao, Z.-Y.; Tu, X.-Q.; Cao, H.-Q. & Yu, J. Anionic chiral Co(III) complexes mediated asymmetric halocyclization—synthesis of 5-halomethyl pyrazolines and isoxazolines. *Org. Lett.* **23**, 9134−9139 (2021).
